# Supplementary material for: The ATG8 E3-like ligases sense lysosomal damage and initiate ESCRT-mediated membrane repair
Source: EMBO J. 2026 Jan 3;45(3):930–52. doi: 10.1038/s44318-025-00672-1 (PMC12865045; doi:10.1038/s44318-025-00672-1)
Supplement: Supplementary file 1 — Appendix [file 44318_2025_672_MOESM1_ESM.pdf]

APPENDIX

ATG8 E3-like ligases sense lysosomal damage and initiate ESCRT-mediated membrane repair

Dale P. Corkery*, Deerada Wijayatunga, Benedita K.L. Feron, Laura K. Herzog, Anastasia Knyazeva, Yao-Wen Wu*

*Corresponding author Email: yaowen.wu@umu.se; dale.corkery@umu.se

Table of Contents:

**Appendix Figure S1**	page 2
**Appendix Figure S2**	page 3
**Appendix Figure S3**	page 3
**Appendix Figure S4**	page 4
**Appendix Figure S5**	page 5
**Appendix Figure S6**	page 5

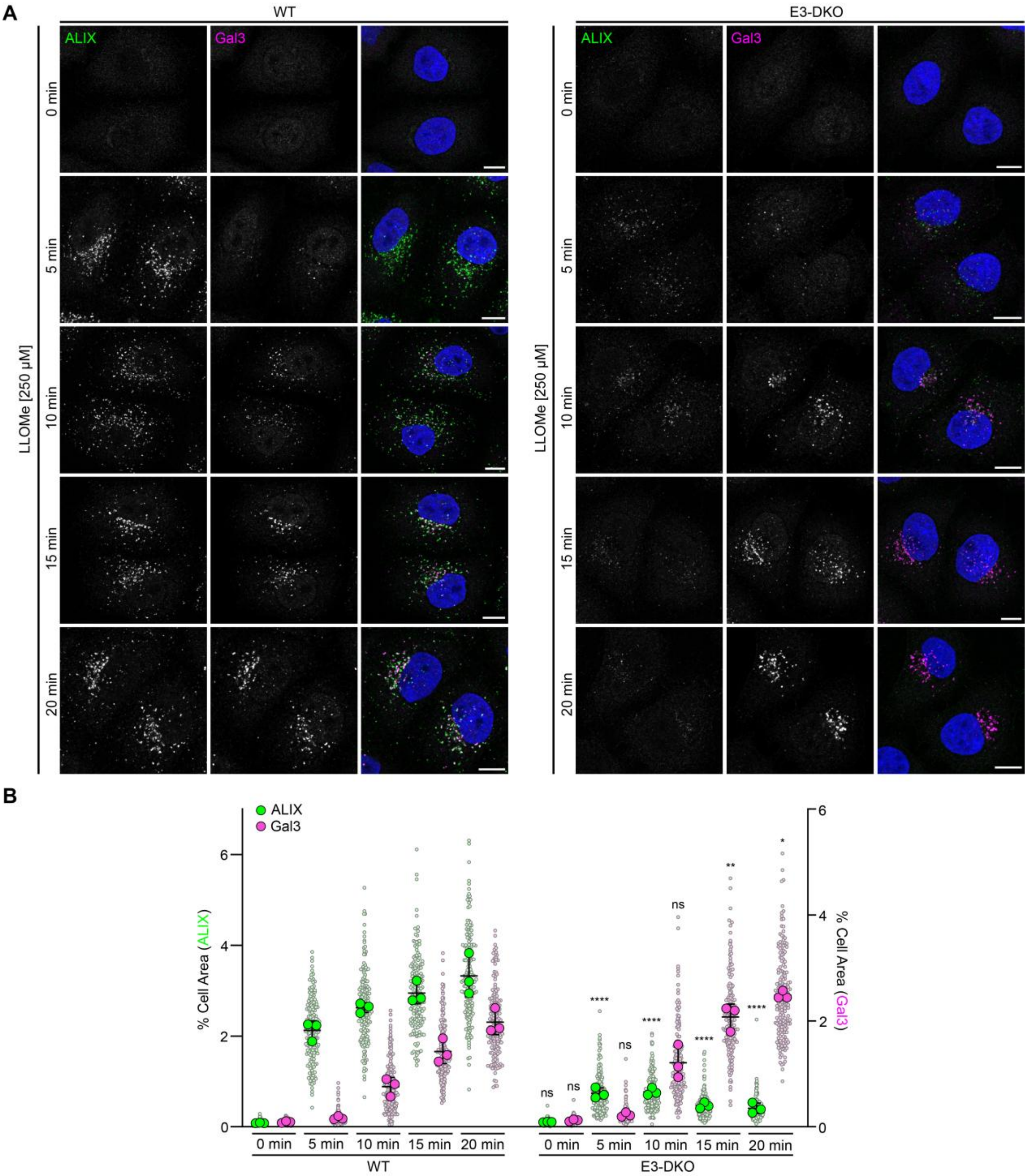

Appendix Figure S1. E3-DKO cells are more susceptible to lysosome rupture. (**A**) Confocal images of HeLa WT and E3-DKO cells treated with 250 µM LLOMe for the indicated time period. Scale bars = 10 µm. (**B**) Quantification of ALIX and Gal3 area from (A). Small points represent individual cells from three independent experiments. Large points represent the means of individual experiments (n = 60 cells per experiment). Bars represent the mean ± SD from the three experiments. Significance was determined from biological replicates using a one-way ANOVA with Tukey's multiple comparisons tests. Comparison of E3-DKO vs matched WT condition is shown. *ns* = not significant (0min ALIX, $p > 0.9999$; 0min Gal3, $p > 0.9999$; 5min Gal3, $p > 0.9999$; 10min Gal3, $p = 0.1056$), * $p = 0.0459$, ** $p = 0.0050$, **** p = < 0.0001.

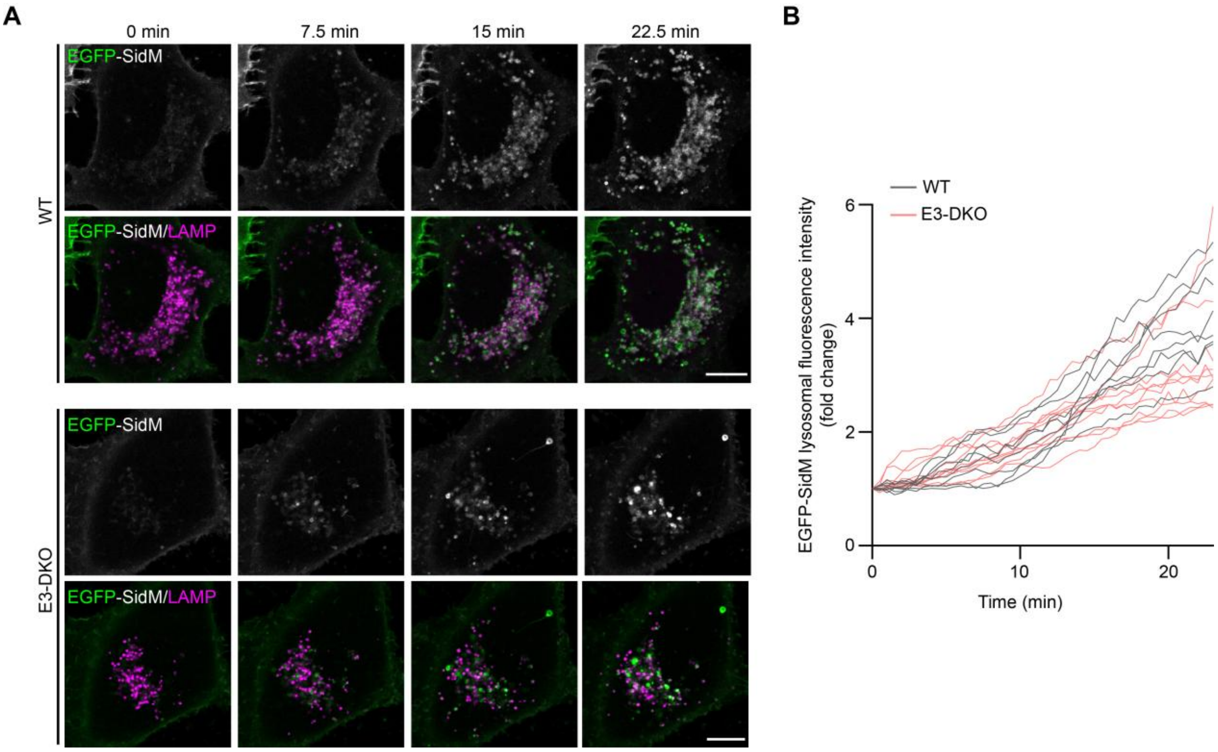

Appendix Figure S2. Damage-induced lysosomal PI4P accumulation is unaffected by the loss of the ATG8 E3 ligase complexes (**A**) Representative live-cell fluorescent images of HeLa WT and ATG16L1/TECPR1 DKO cells co-transfected with EGFP-SidM (PI4P biosensor) and LAMP1-mCherry and treated with 1 mM LLOMe for the indicated time. Scale bar = 10 µM. (**B**) Quantification of the fold change in EGFP-SidM lysosomal fluorescence from (A). Lines represent individual cells from three independent experiments.

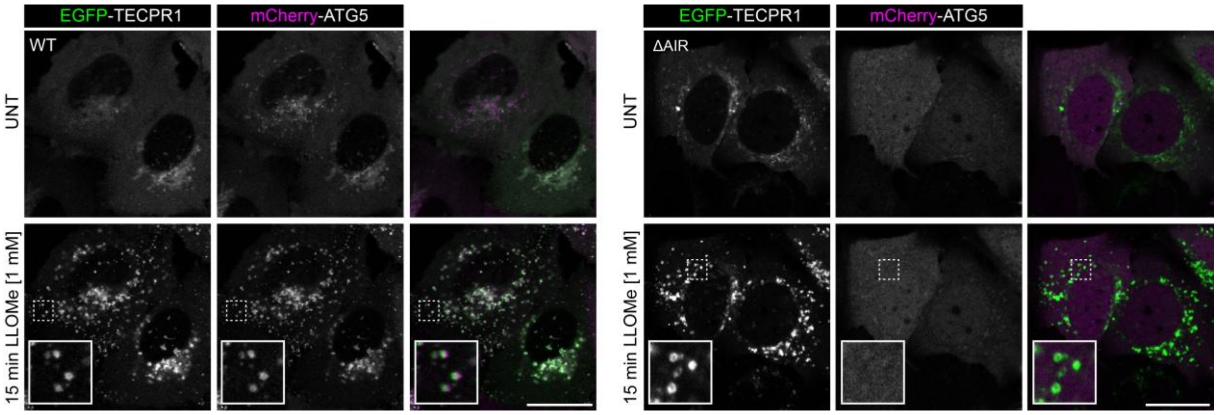

Appendix Figure S3. Deletion of the ATG5 interacting region of TECPR1 prevents ATG5 recruitment to lysosomes in response to damage. Representative live-cell fluorescent images of HeLa WT cells co-transfected with EGFP-TECPR1 and mCherry-ATG5 and treated with 1 mM LLOMe for 15 minutes. Scale bar = 20 µM.

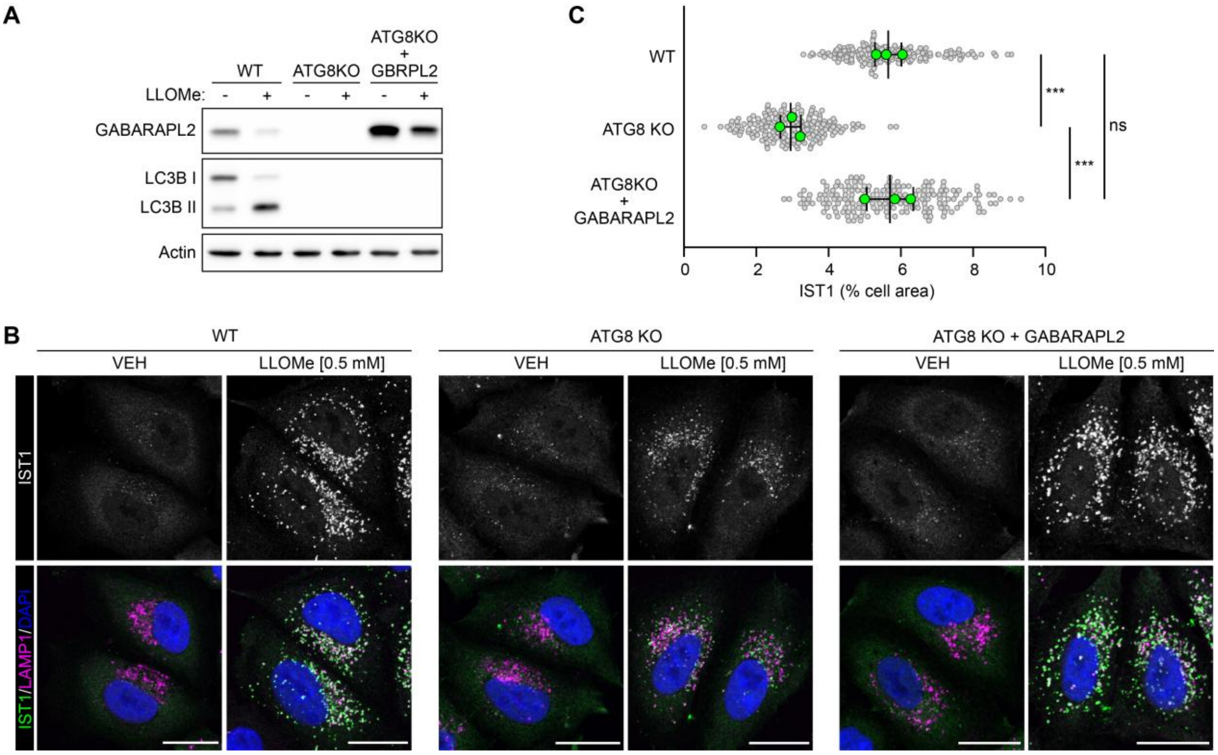

Appendix Figure S4. GABARAPL2 addback rescues ESCRT recruitment in ATG8KO cells. (**A**) Western blot analysis of GABARAPL2 addback cell line. (**B**) Confocal images of HeLa WT, ATG8KO and GABARAPL2 addback cells treated with 0.5 mM LLOMe (or vehicle) for 20 minutes. Scale bars = 20 µm. (**C**) Quantification of IST1 cell area from (B). Small points represent individual cells from three independent experiments. Large points represent the means of individual experiments (n = 60 cells per experiment). Bars represent the mean ± SD from the three experiments. Significance was determined from biological replicates using a one-way ANOVA with Tukey's multiple comparisons tests. *ns* = not significant (p = 0.9904), *** (WT vs 8KO) p = 0.0009, *** (8KO vs 8KO + GBRP) p = 0.0008.

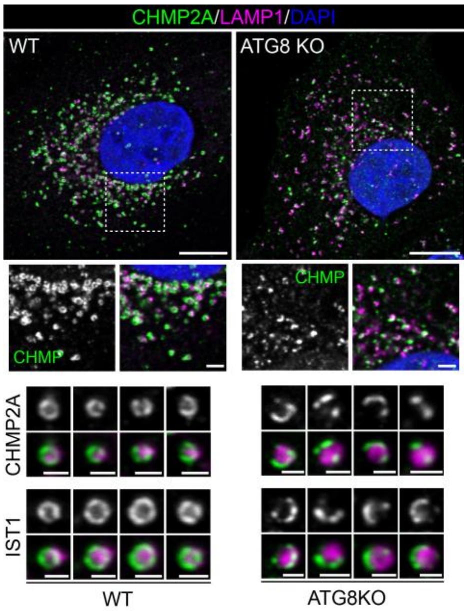

Appendix Figure S5. ESCRT recruitment to damaged lysosomes in the presence and absence of ATG8s. Confocal images of HeLa WT and ATG8 KO cells treated with 1 mM LLOMe for 20 minutes. Scale bars = 10 µm for whole cell images, 2 µm for insets, and 1 µm for individual lysosome images.

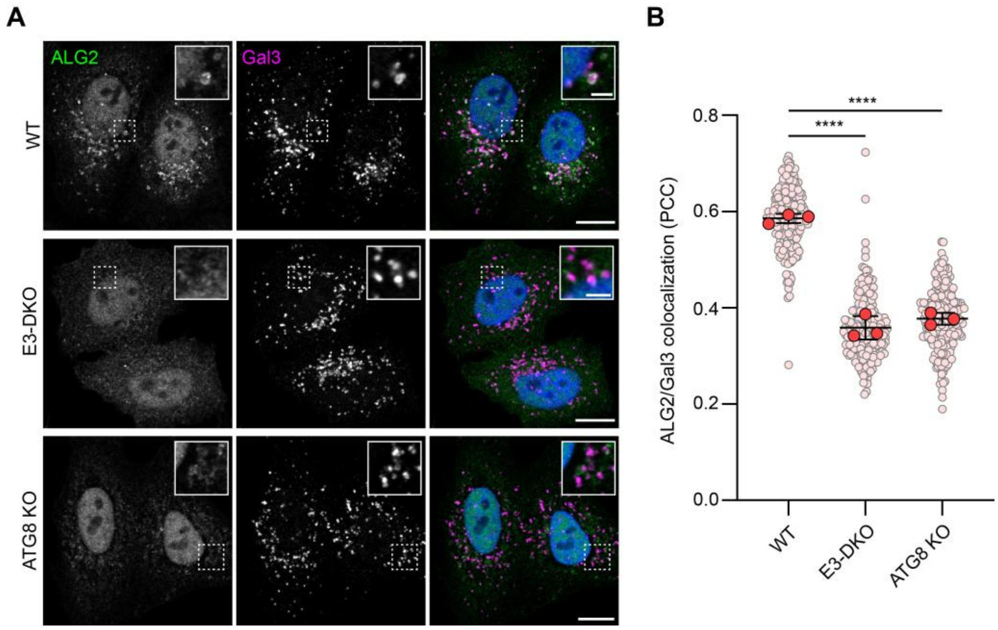

Appendix Fig. S6. ALG-2 recruitment to damaged lysosomes is dependent on ATG8 and the ATG8 E3-like ligases. (**A**) Confocal images of HeLa WT, E3-DKO and ATG8 KO cells treated with 1 mM LLOMe for 30 minutes. (**B**) Quantification of ALG-2/Gal3 colocalization from (A). Small points represent individual cells from three independent experiments. Large points represent the means of individual experiments (n > 50 cells per experiment). Bars represent the mean ± SD from the three experiments. Significance was determined from biological replicates using a one-way ANOVA with Tukey's multiple comparisons tests. **** p = < 0.0001.
