# Supplementary figures and images for: The ATG8 E3-like ligases sense lysosomal damage and initiate ESCRT-mediated membrane repair (part 2 of 7)
Source: EMBO J. 2026 Jan 3;45(3):930–52. doi: 10.1038/s44318-025-00672-1 (PMC12865045; doi:10.1038/s44318-025-00672-1)

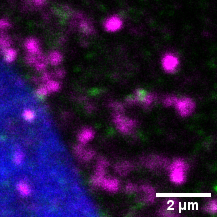

Supplement: Supplementary file 3 — Source data Fig. 1 [file 44318_2025_672_MOESM3_ESM.zip › Figure 1/1H/dAIR_scale_zoom.tif]

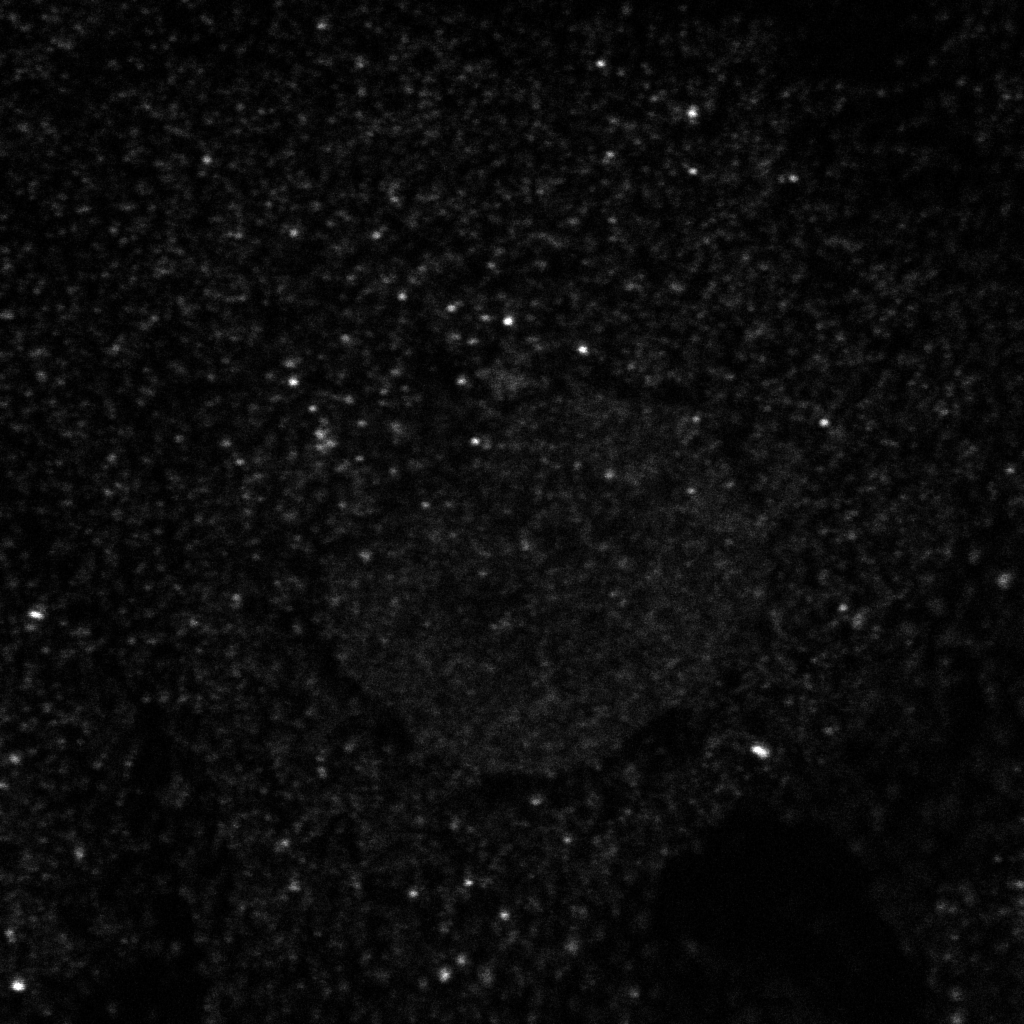

Supplement: Supplementary file 3 — Source data Fig. 1 [file 44318_2025_672_MOESM3_ESM.zip › Figure 1/1H/KO_ALIX.tif]

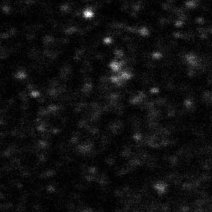

Supplement: Supplementary file 3 — Source data Fig. 1 [file 44318_2025_672_MOESM3_ESM.zip › Figure 1/1H/KO_ALIX_zoom.tif]

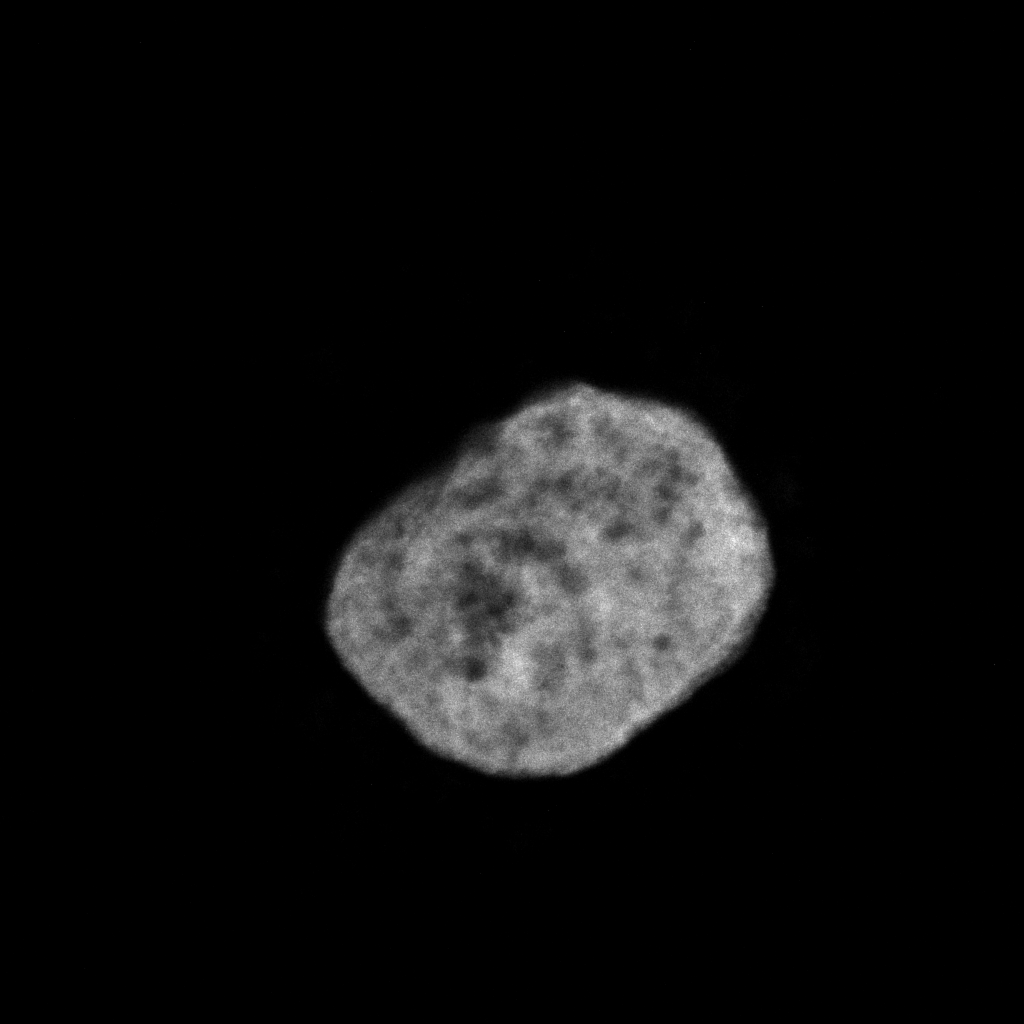

Supplement: Supplementary file 3 — Source data Fig. 1 [file 44318_2025_672_MOESM3_ESM.zip › Figure 1/1H/KO_DAPI.tif]

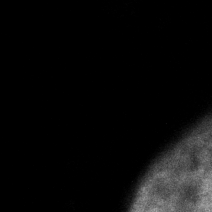

Supplement: Supplementary file 3 — Source data Fig. 1 [file 44318_2025_672_MOESM3_ESM.zip › Figure 1/1H/KO_DAPI_zoom.tif]

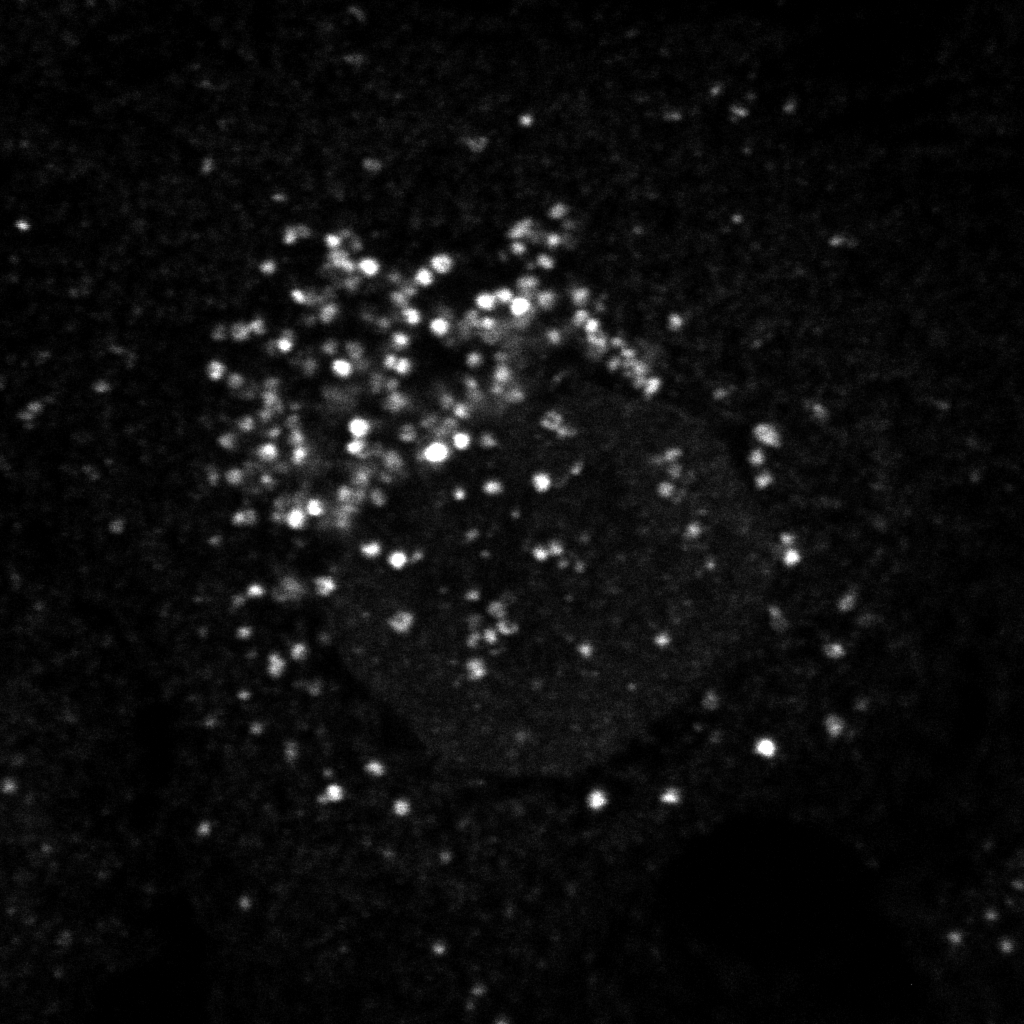

Supplement: Supplementary file 3 — Source data Fig. 1 [file 44318_2025_672_MOESM3_ESM.zip › Figure 1/1H/KO_Gal3.tif]

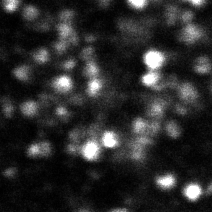

Supplement: Supplementary file 3 — Source data Fig. 1 [file 44318_2025_672_MOESM3_ESM.zip › Figure 1/1H/KO_Gal3_zoom.tif]

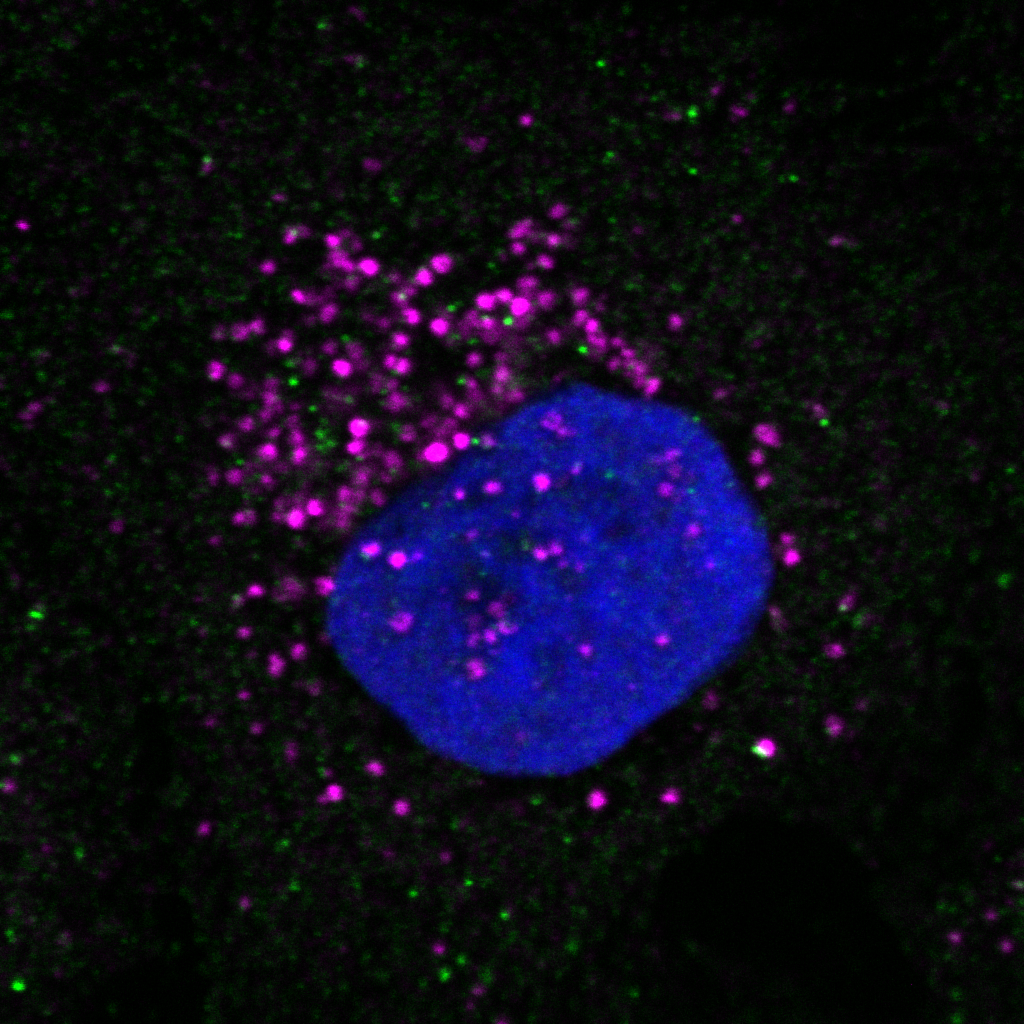

Supplement: Supplementary file 3 — Source data Fig. 1 [file 44318_2025_672_MOESM3_ESM.zip › Figure 1/1H/KO_merge.tif]

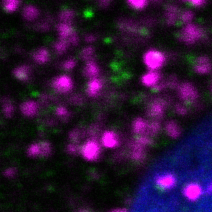

Supplement: Supplementary file 3 — Source data Fig. 1 [file 44318_2025_672_MOESM3_ESM.zip › Figure 1/1H/KO_merge_zoom.tif]

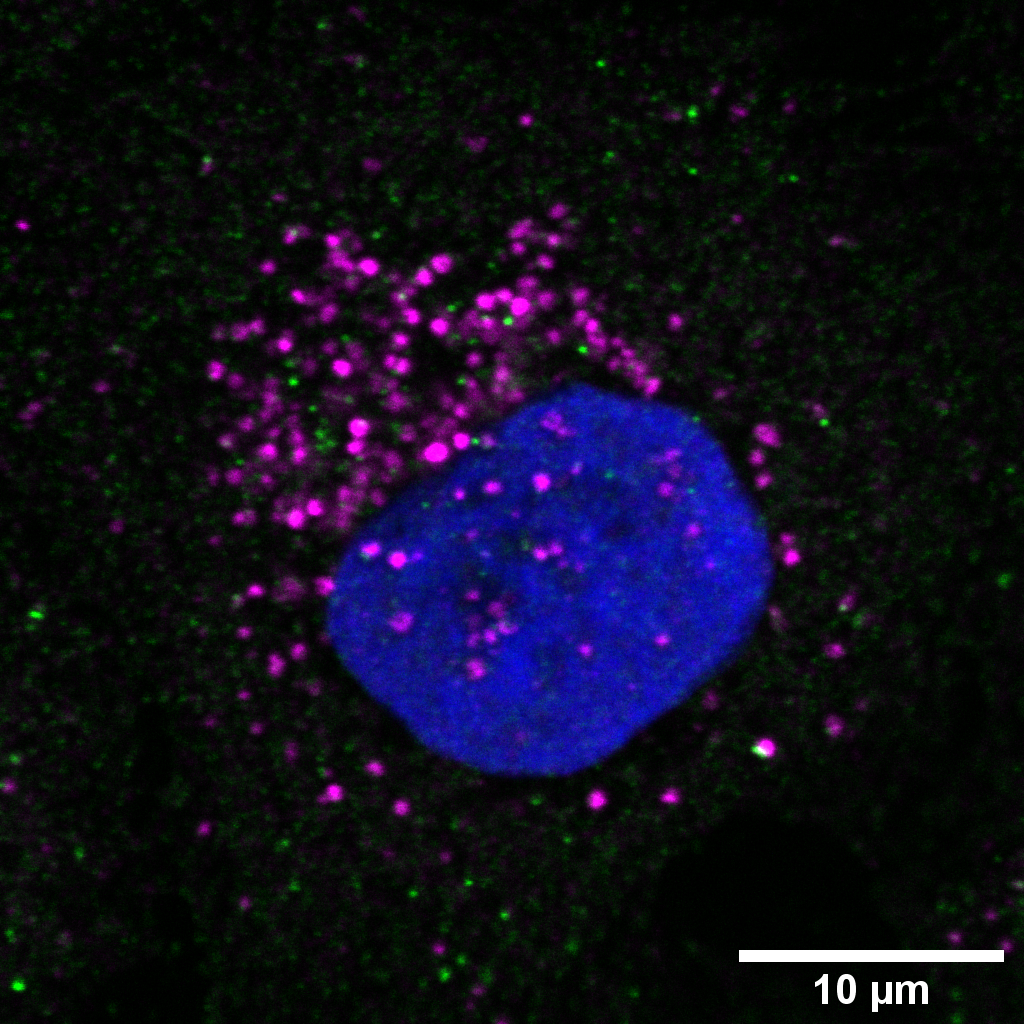

Supplement: Supplementary file 3 — Source data Fig. 1 [file 44318_2025_672_MOESM3_ESM.zip › Figure 1/1H/KO_scale.tif]

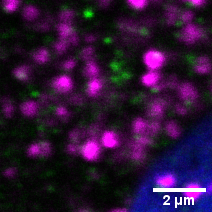

Supplement: Supplementary file 3 — Source data Fig. 1 [file 44318_2025_672_MOESM3_ESM.zip › Figure 1/1H/KO_scale_zoom.tif]

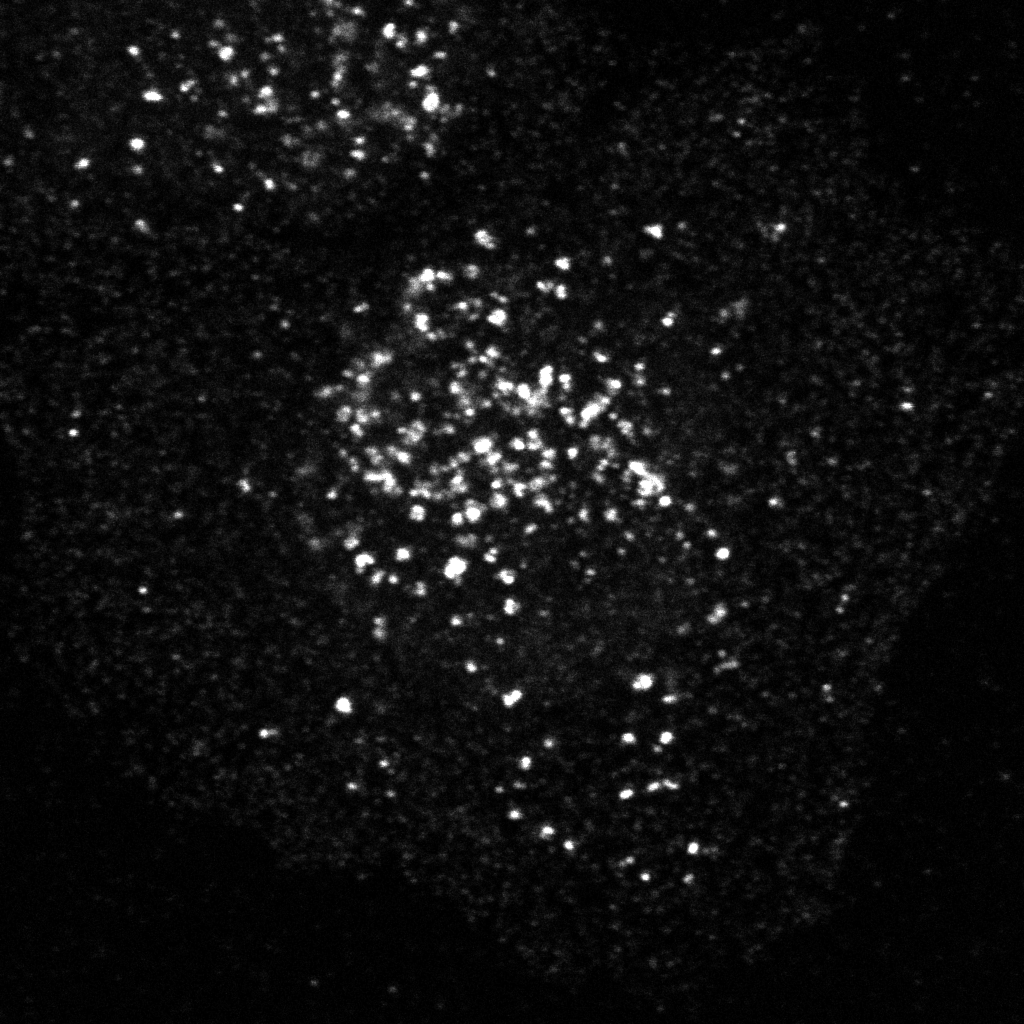

Supplement: Supplementary file 3 — Source data Fig. 1 [file 44318_2025_672_MOESM3_ESM.zip › Figure 1/1H/WT_ALIX.tif]

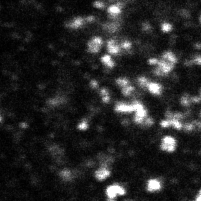

Supplement: Supplementary file 3 — Source data Fig. 1 [file 44318_2025_672_MOESM3_ESM.zip › Figure 1/1H/WT_ALIX_zoom.tif]

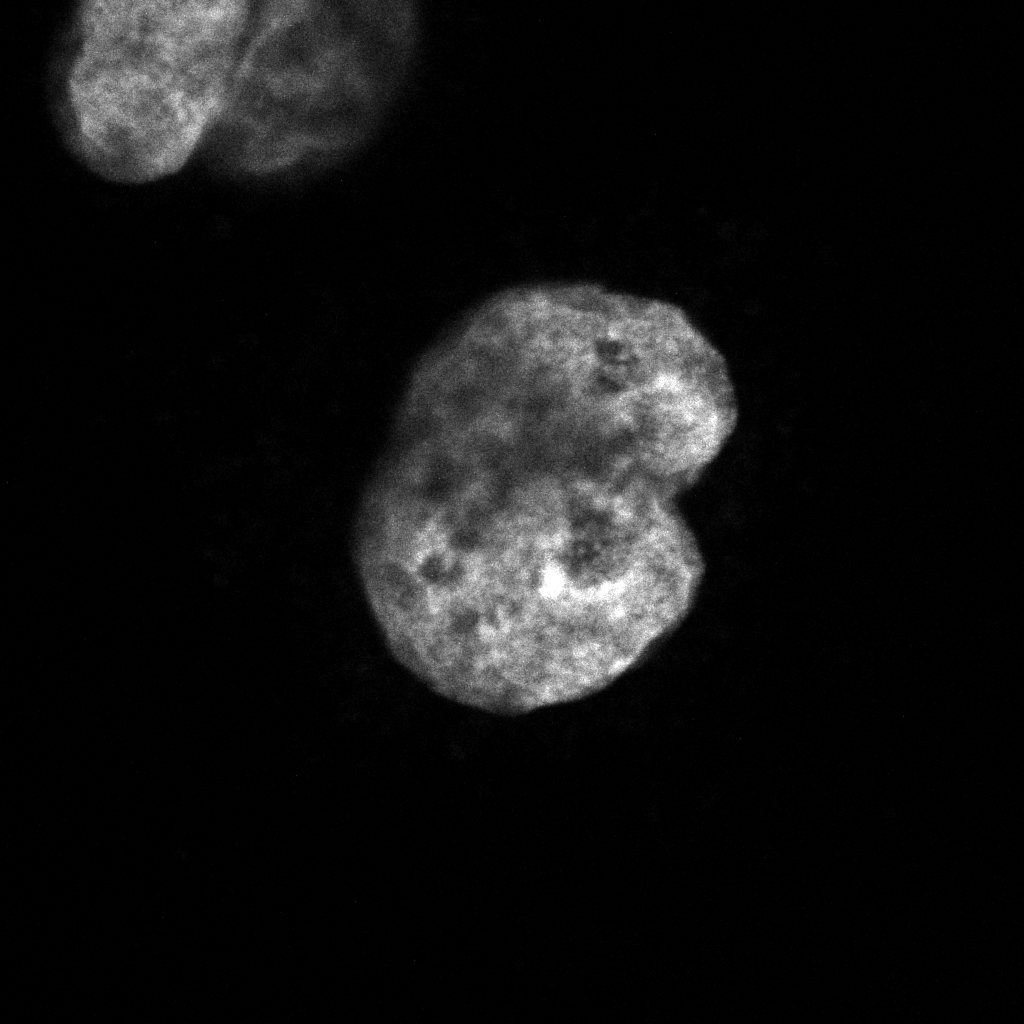

Supplement: Supplementary file 3 — Source data Fig. 1 [file 44318_2025_672_MOESM3_ESM.zip › Figure 1/1H/WT_DAPI.tif]

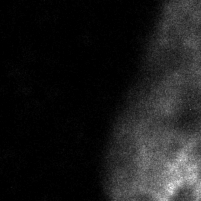

Supplement: Supplementary file 3 — Source data Fig. 1 [file 44318_2025_672_MOESM3_ESM.zip › Figure 1/1H/WT_DAPI_zoom.tif]

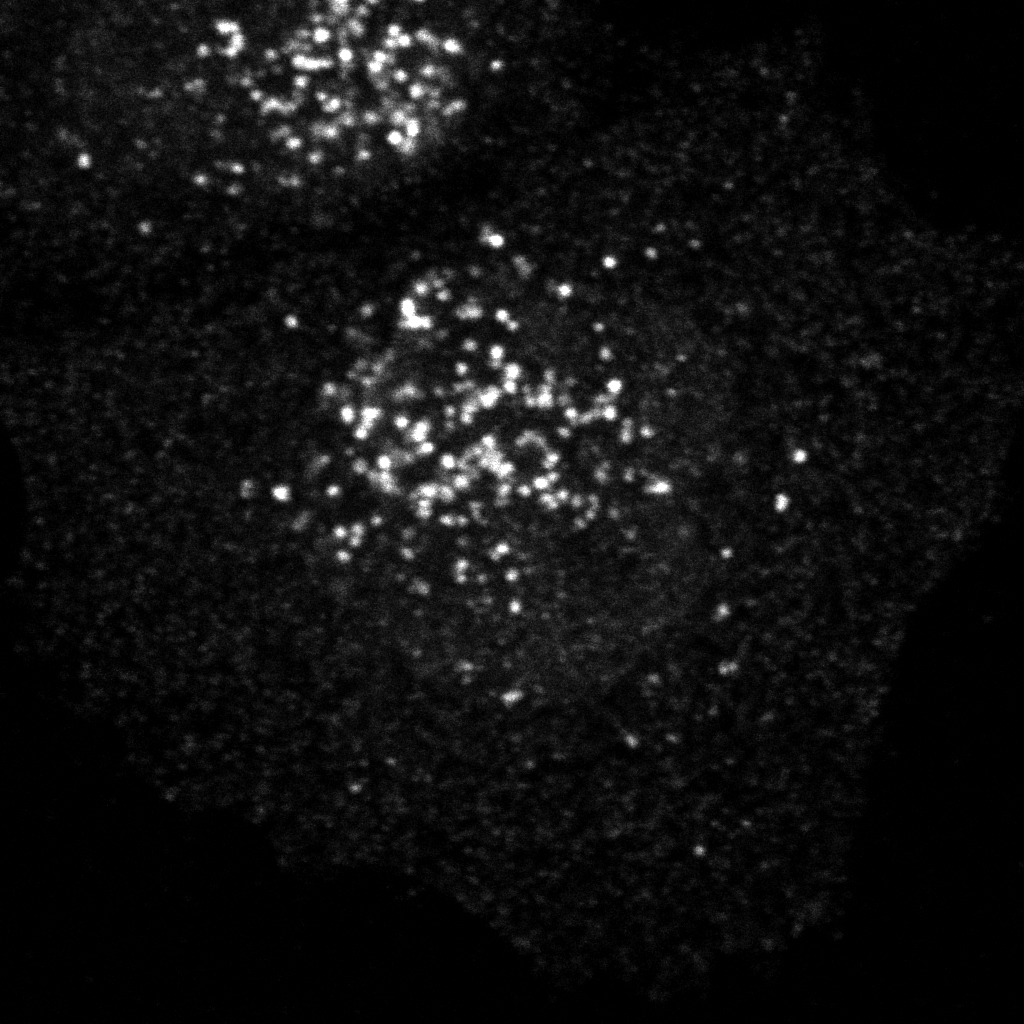

Supplement: Supplementary file 3 — Source data Fig. 1 [file 44318_2025_672_MOESM3_ESM.zip › Figure 1/1H/WT_Gal3.tif]

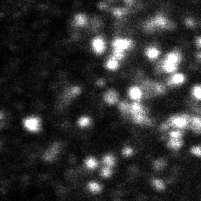

Supplement: Supplementary file 3 — Source data Fig. 1 [file 44318_2025_672_MOESM3_ESM.zip › Figure 1/1H/WT_Gal3_zoom.tif]

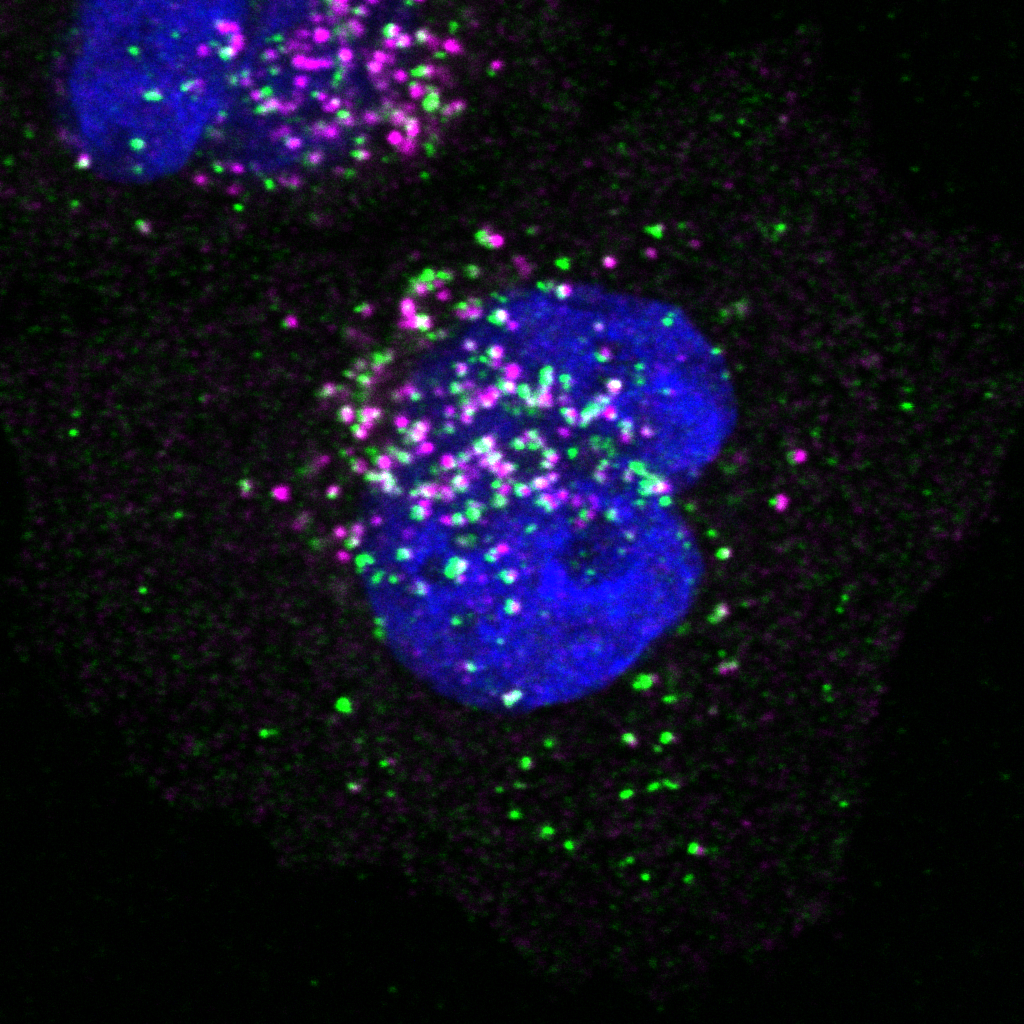

Supplement: Supplementary file 3 — Source data Fig. 1 [file 44318_2025_672_MOESM3_ESM.zip › Figure 1/1H/WT_merge.tif]

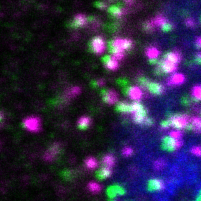

Supplement: Supplementary file 3 — Source data Fig. 1 [file 44318_2025_672_MOESM3_ESM.zip › Figure 1/1H/WT_merge_zoom.tif]

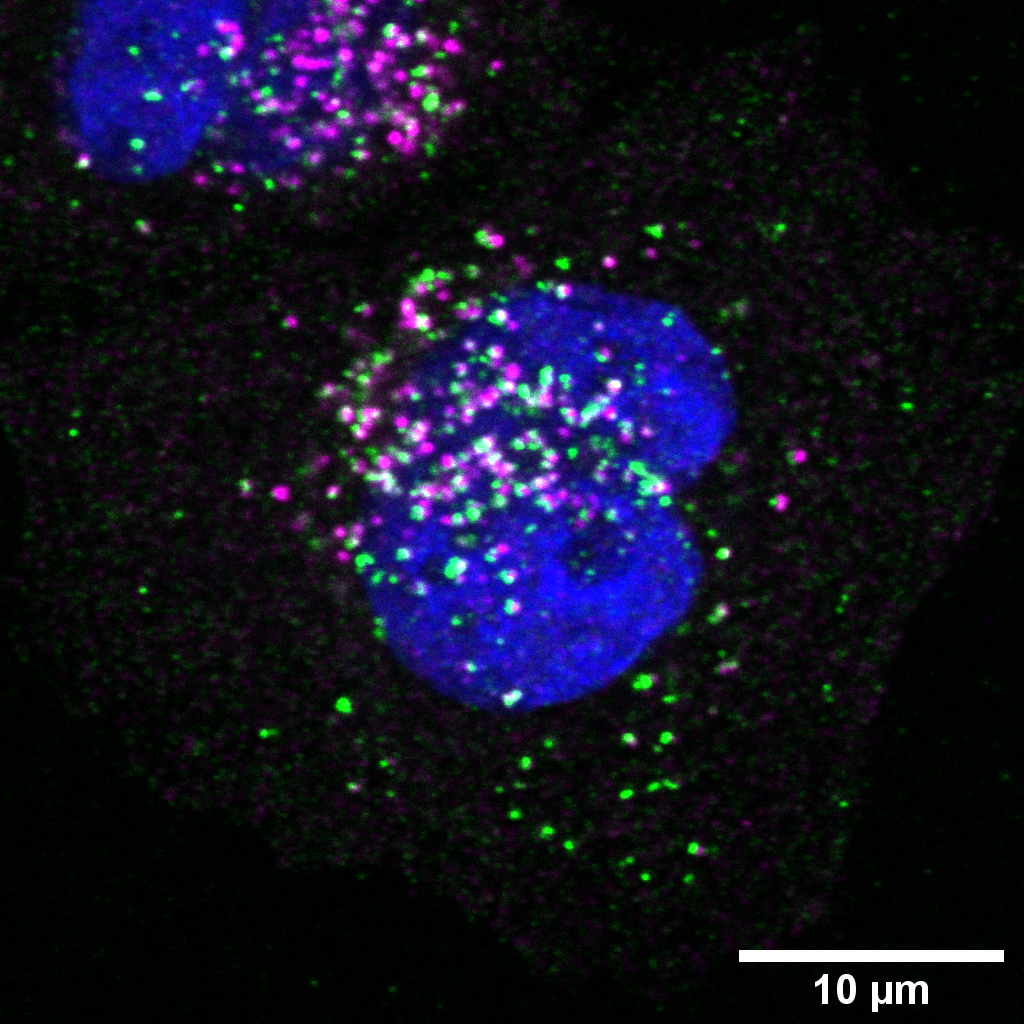

Supplement: Supplementary file 3 — Source data Fig. 1 [file 44318_2025_672_MOESM3_ESM.zip › Figure 1/1H/WT_scale.tif]

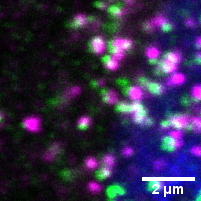

Supplement: Supplementary file 3 — Source data Fig. 1 [file 44318_2025_672_MOESM3_ESM.zip › Figure 1/1H/WT_scale_zoom.tif]

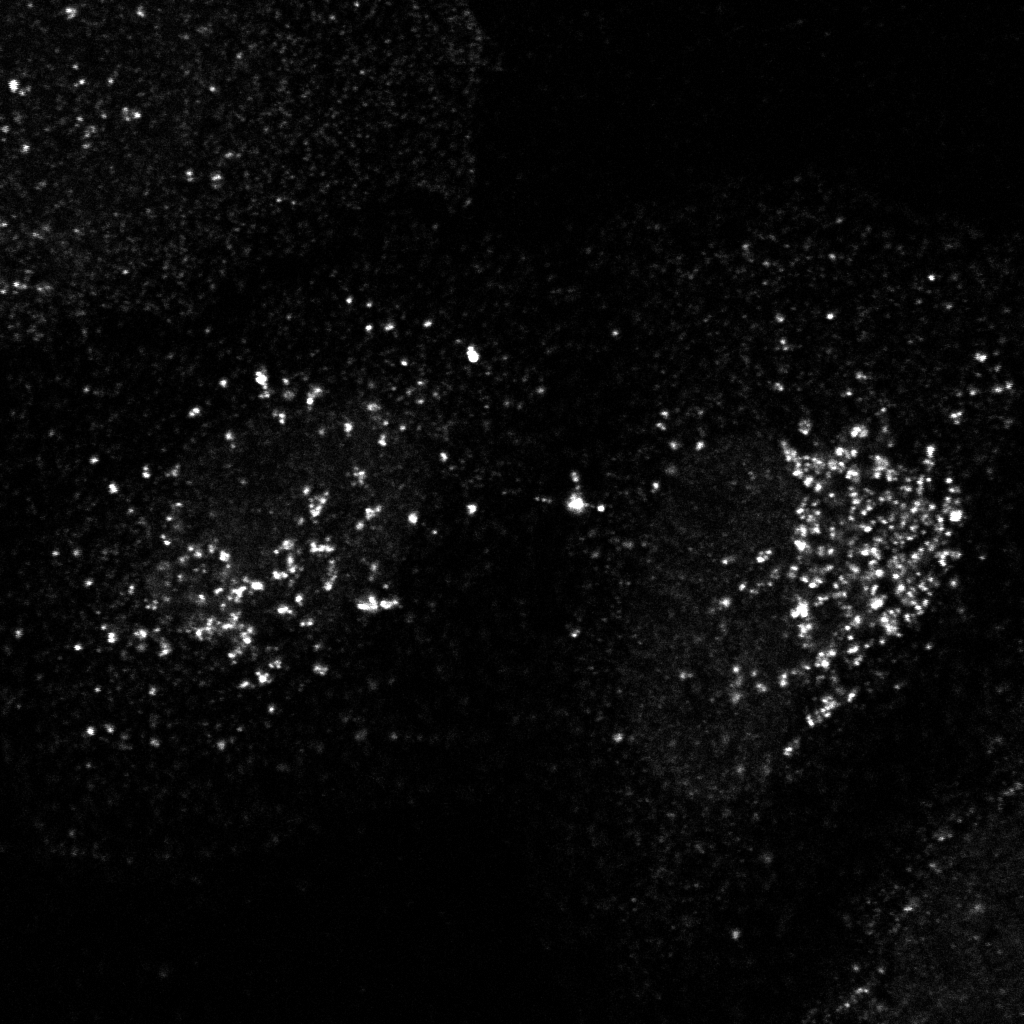

Supplement: Supplementary file 4 — Source data Fig. 2 [file 44318_2025_672_MOESM4_ESM.zip › Figure 2/2A/4KO_LLOMe_ALIX.tif]

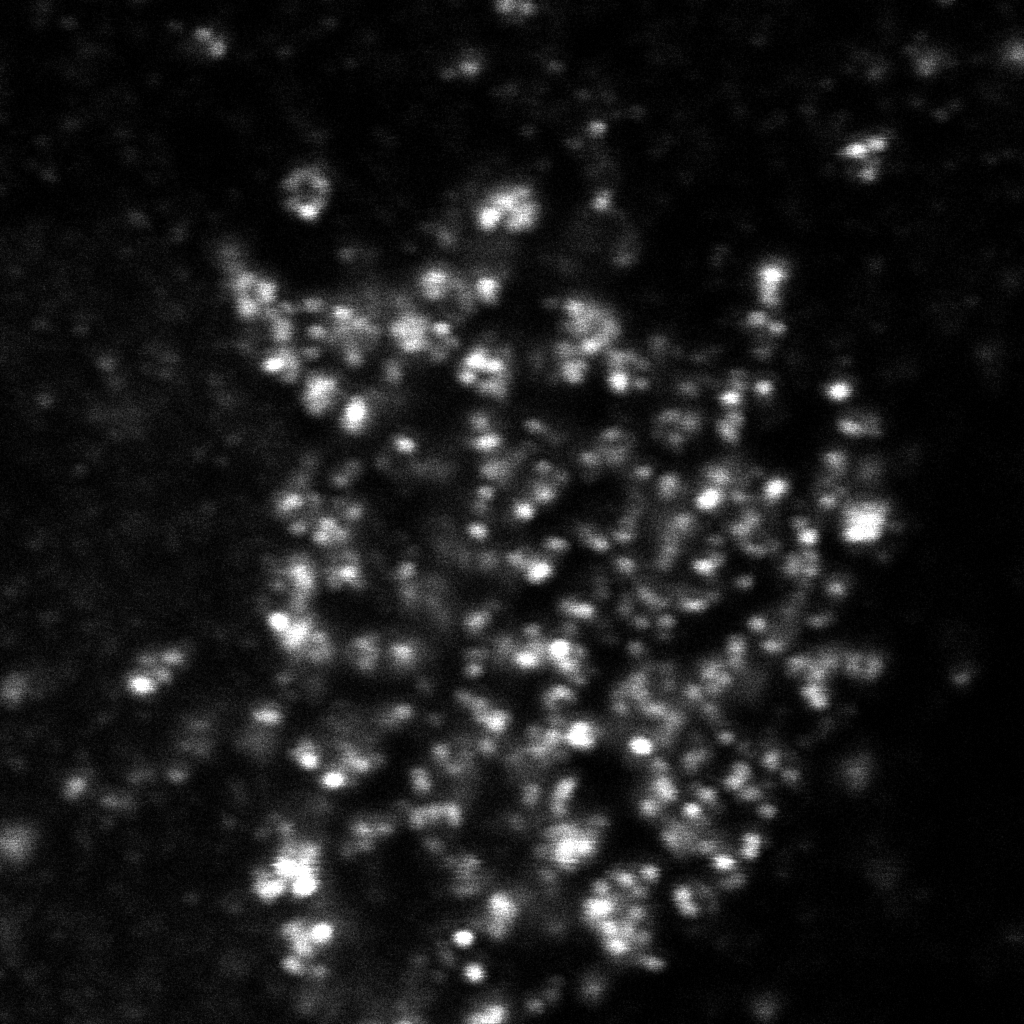

Supplement: Supplementary file 4 — Source data Fig. 2 [file 44318_2025_672_MOESM4_ESM.zip › Figure 2/2A/4KO_LLOMe_ALIX_zoom.tif]

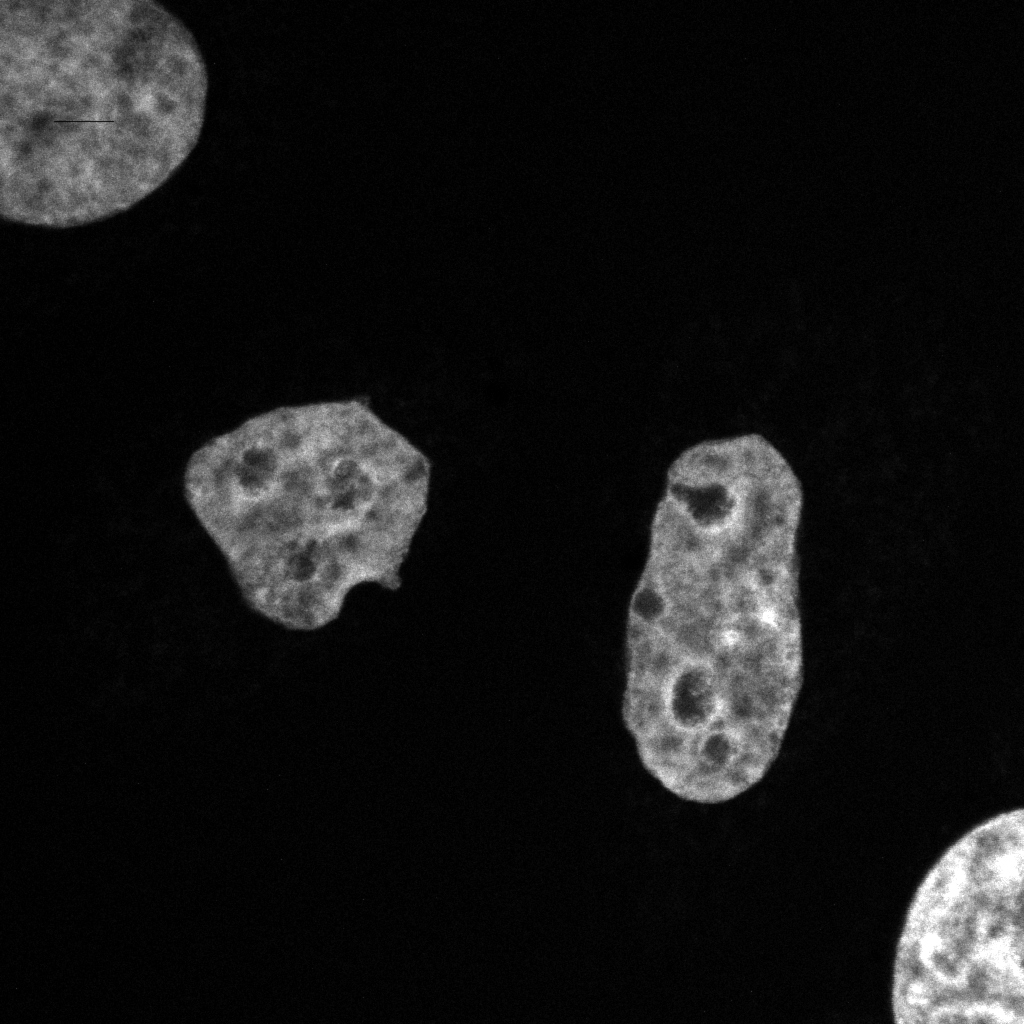

Supplement: Supplementary file 4 — Source data Fig. 2 [file 44318_2025_672_MOESM4_ESM.zip › Figure 2/2A/4KO_LLOMe_DAPI.tif]

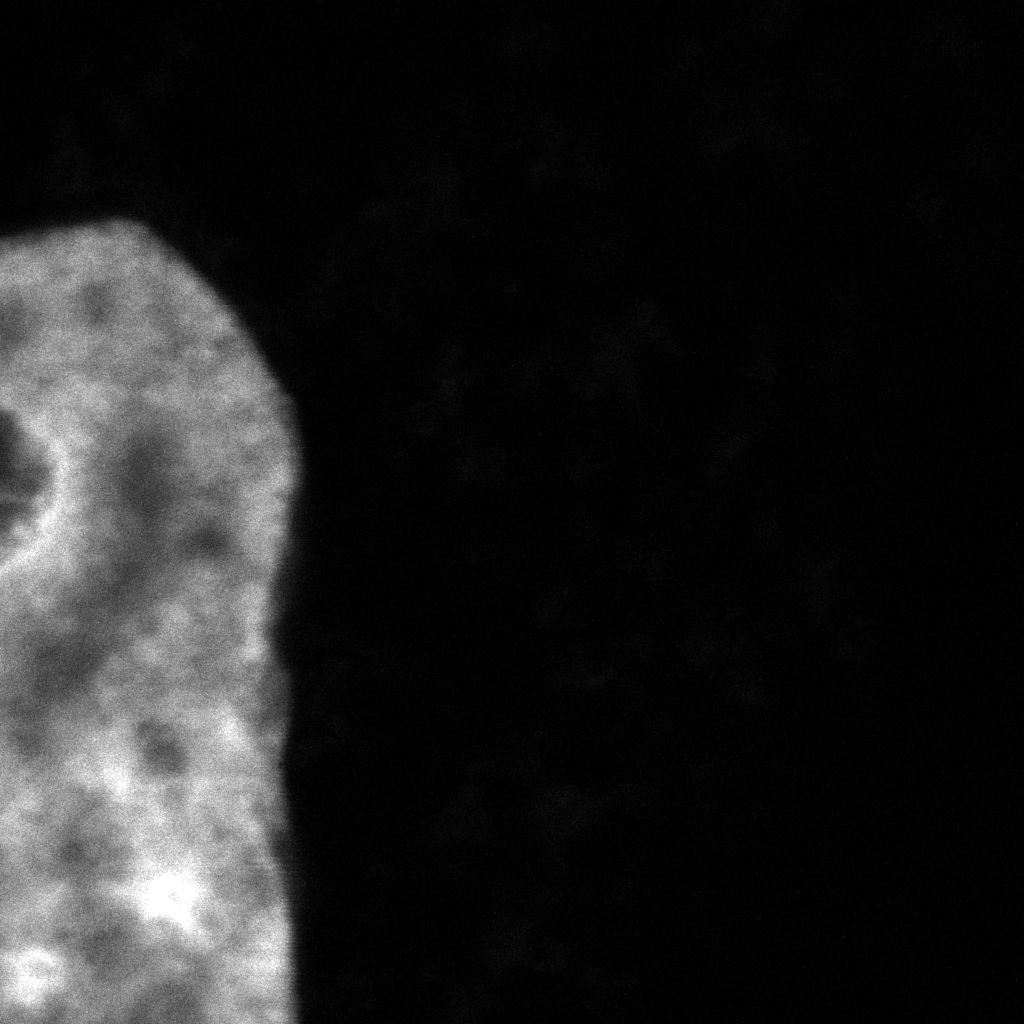

Supplement: Supplementary file 4 — Source data Fig. 2 [file 44318_2025_672_MOESM4_ESM.zip › Figure 2/2A/4KO_LLOMe_DAPI_zoom.tif]

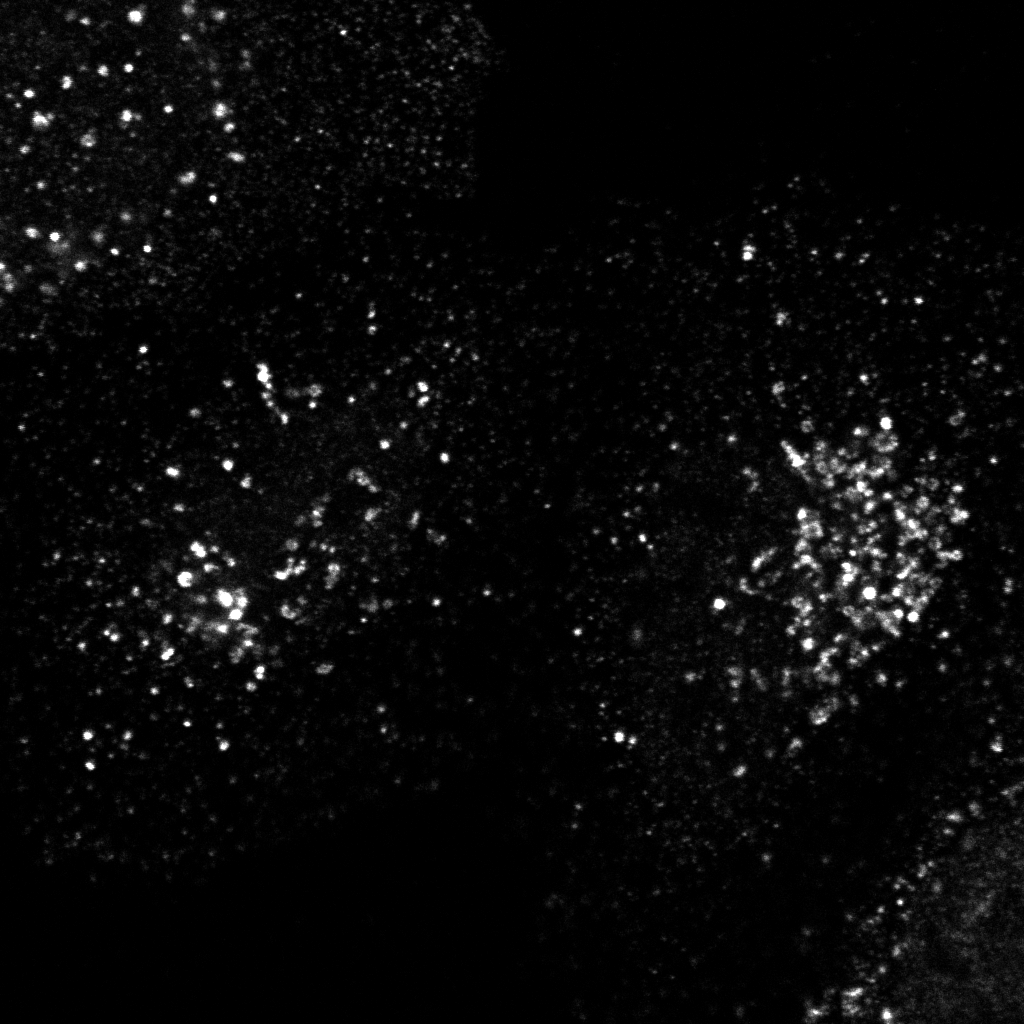

Supplement: Supplementary file 4 — Source data Fig. 2 [file 44318_2025_672_MOESM4_ESM.zip › Figure 2/2A/4KO_LLOMe_Gal3.tif]

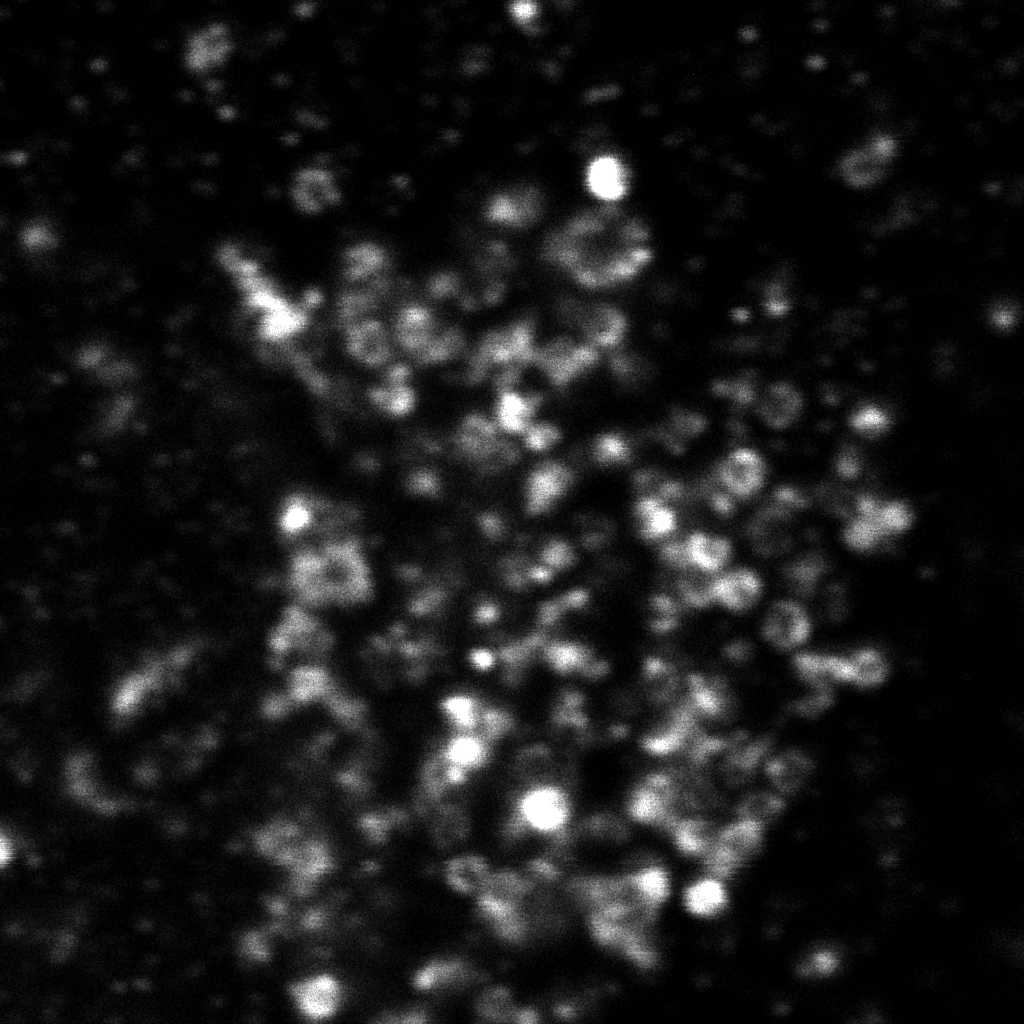

Supplement: Supplementary file 4 — Source data Fig. 2 [file 44318_2025_672_MOESM4_ESM.zip › Figure 2/2A/4KO_LLOMe_Gal3_zoom.tif]

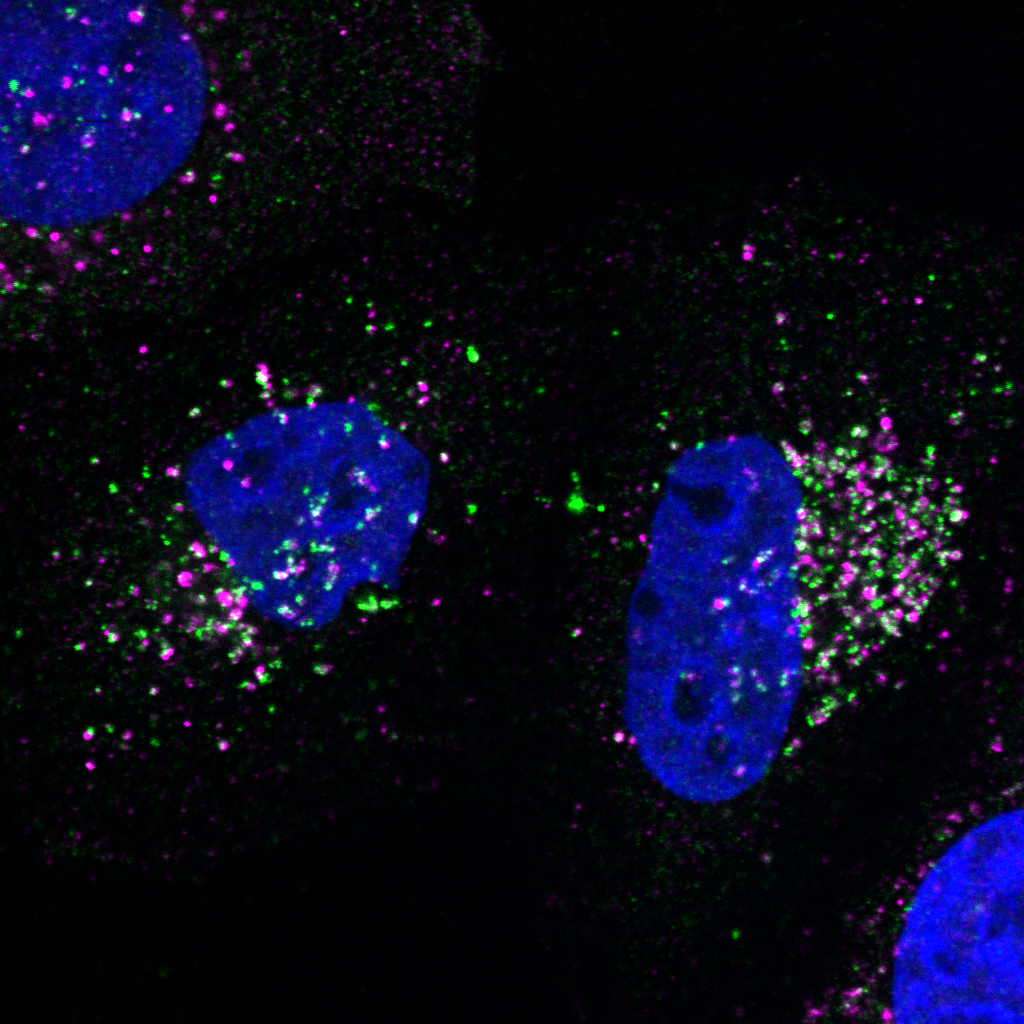

Supplement: Supplementary file 4 — Source data Fig. 2 [file 44318_2025_672_MOESM4_ESM.zip › Figure 2/2A/4KO_LLOMe_merge.tif]

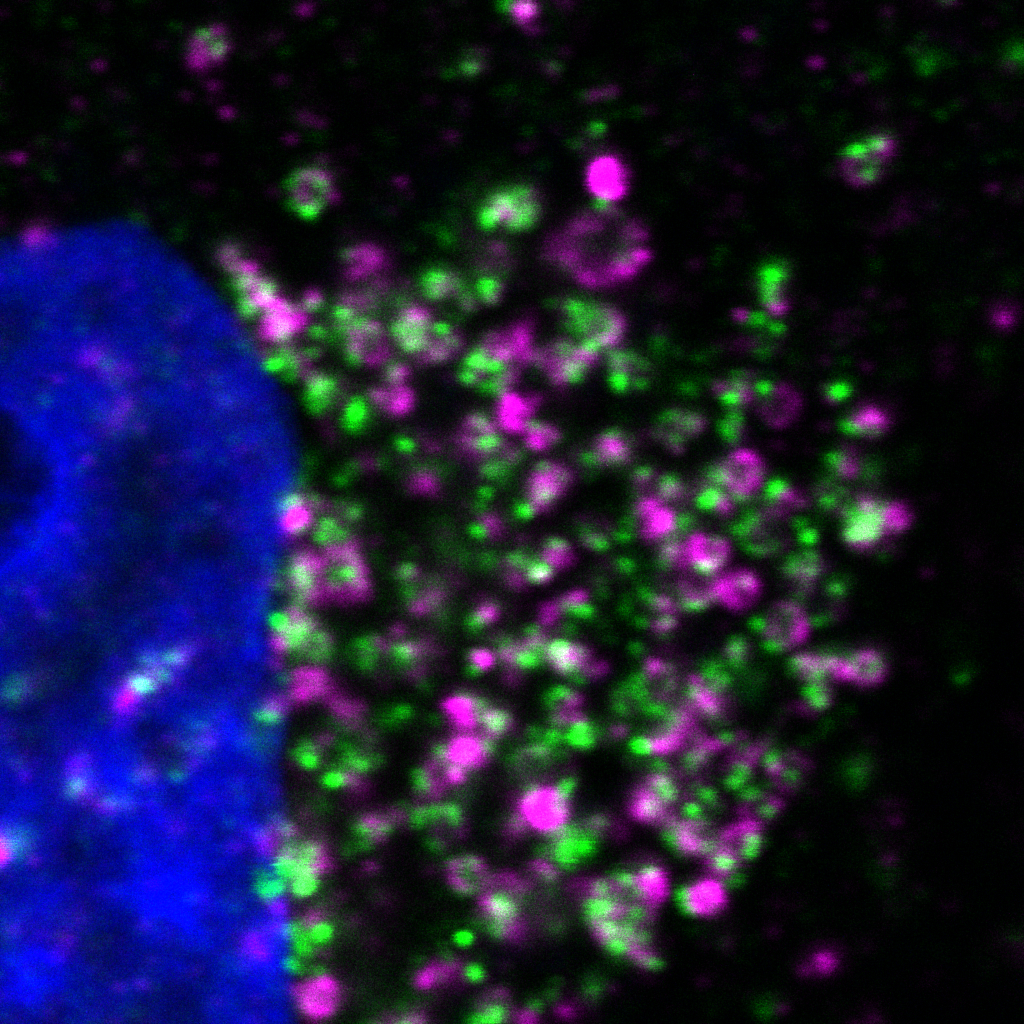

Supplement: Supplementary file 4 — Source data Fig. 2 [file 44318_2025_672_MOESM4_ESM.zip › Figure 2/2A/4KO_LLOMe_merge_zoom.tif]

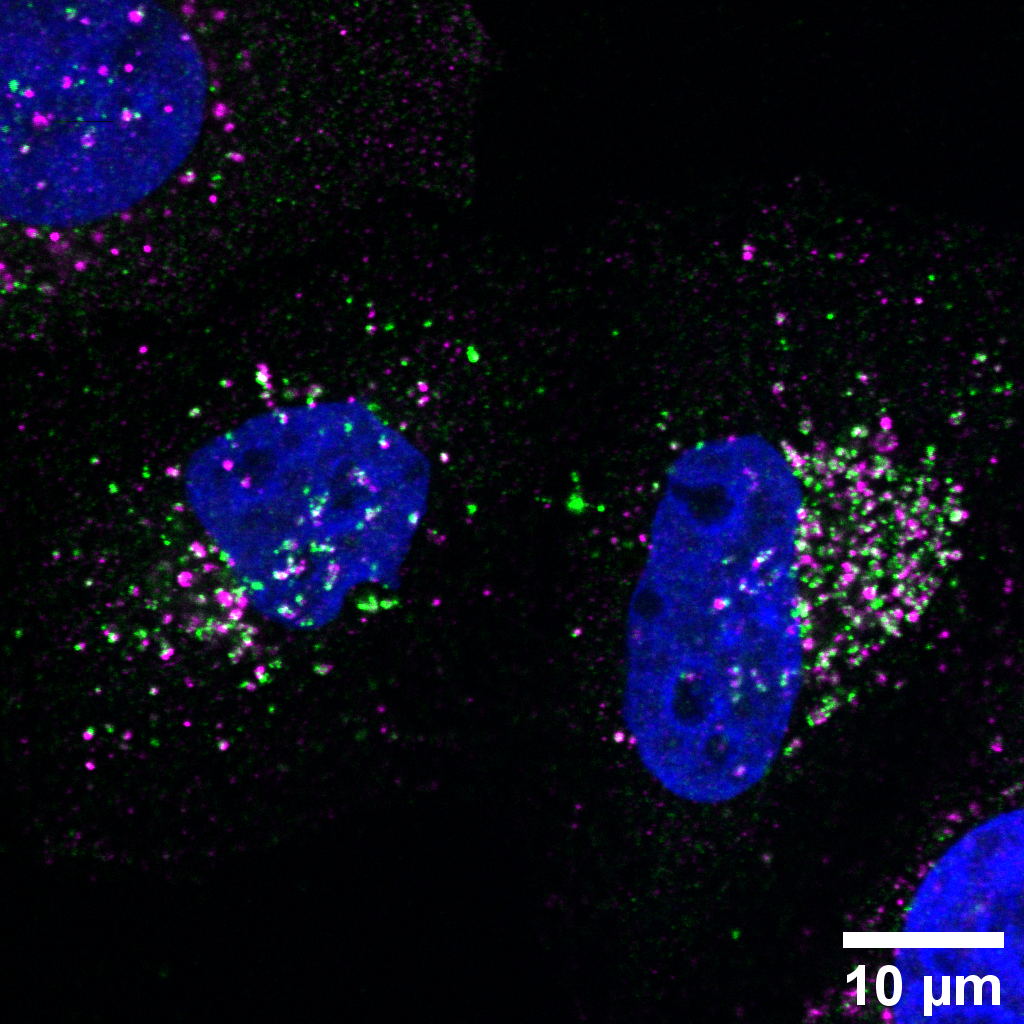

Supplement: Supplementary file 4 — Source data Fig. 2 [file 44318_2025_672_MOESM4_ESM.zip › Figure 2/2A/4KO_LLOMe_scale.tif]

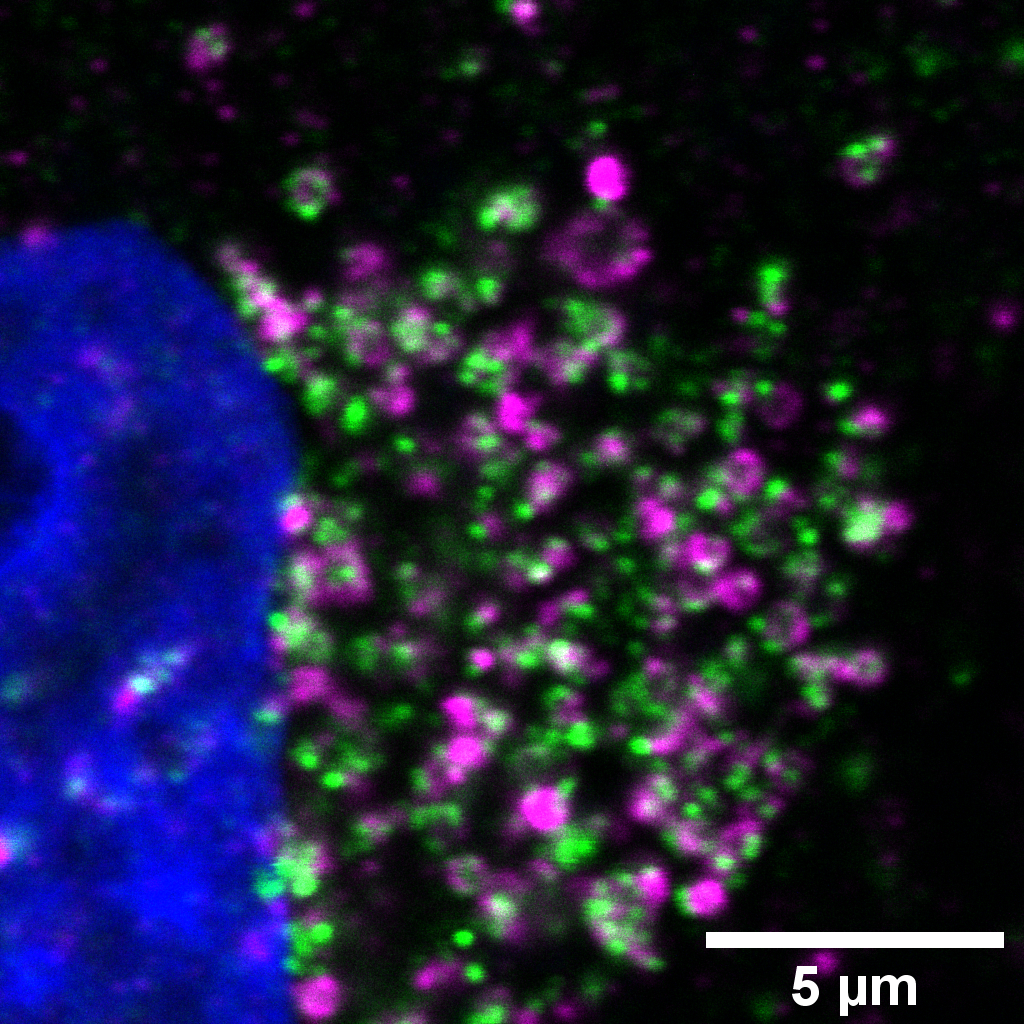

Supplement: Supplementary file 4 — Source data Fig. 2 [file 44318_2025_672_MOESM4_ESM.zip › Figure 2/2A/4KO_LLOMe_scale_zoom.tif]

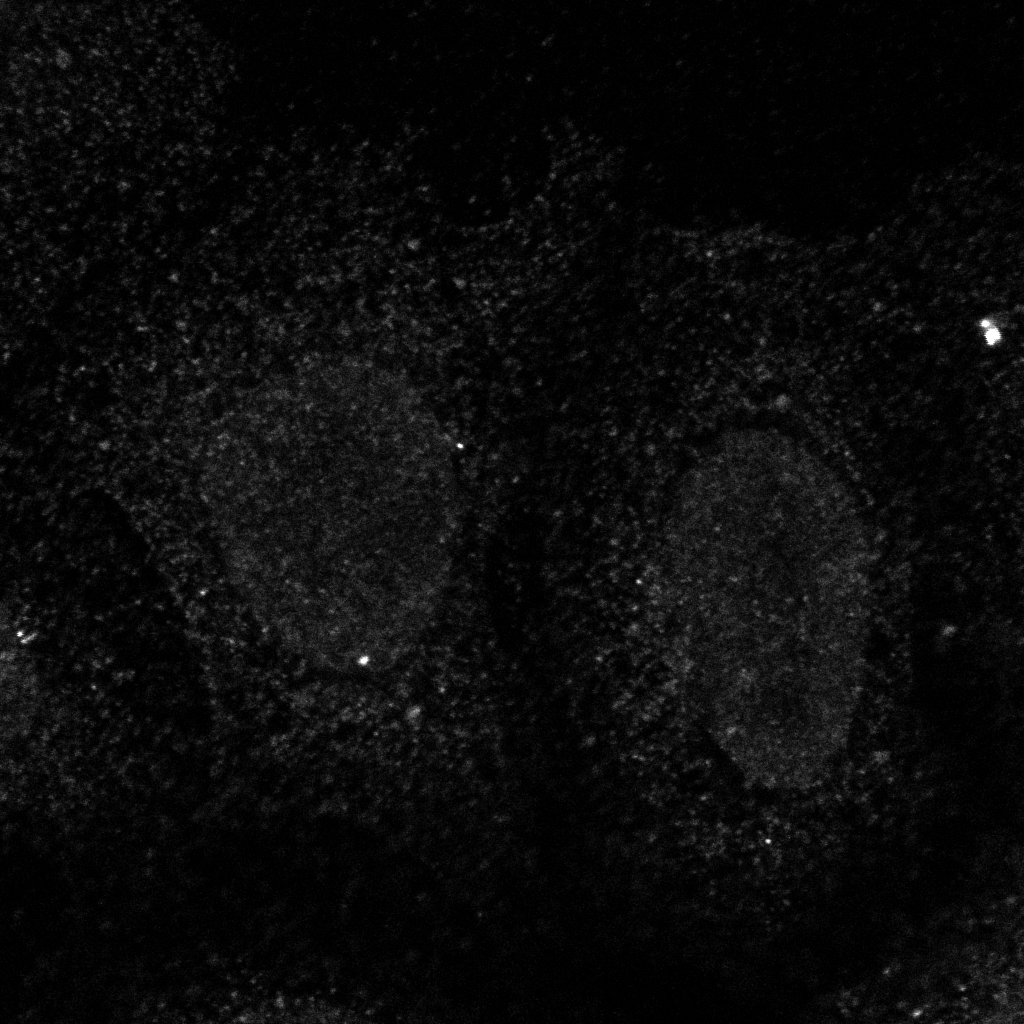

Supplement: Supplementary file 4 — Source data Fig. 2 [file 44318_2025_672_MOESM4_ESM.zip › Figure 2/2A/4KO_VEH_ALIX.tif]

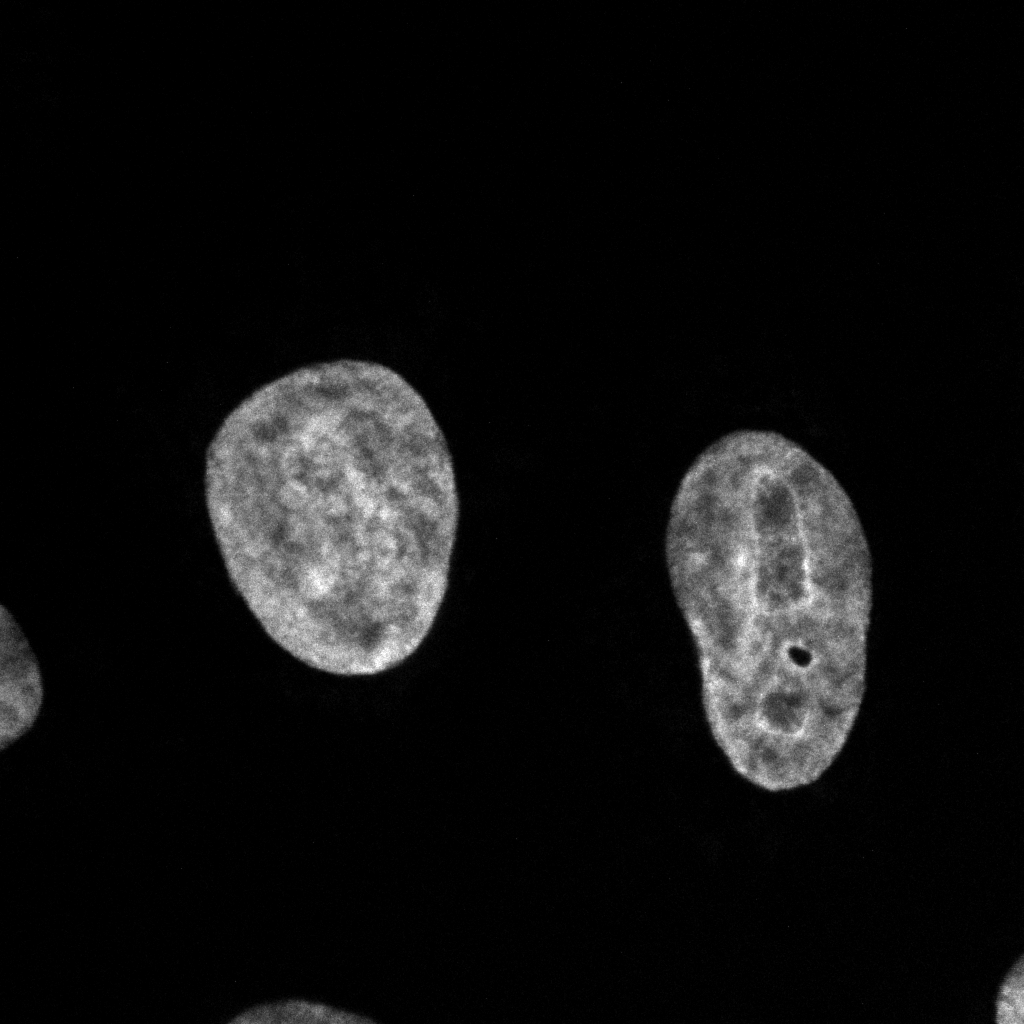

Supplement: Supplementary file 4 — Source data Fig. 2 [file 44318_2025_672_MOESM4_ESM.zip › Figure 2/2A/4KO_VEH_DAPI.tif]

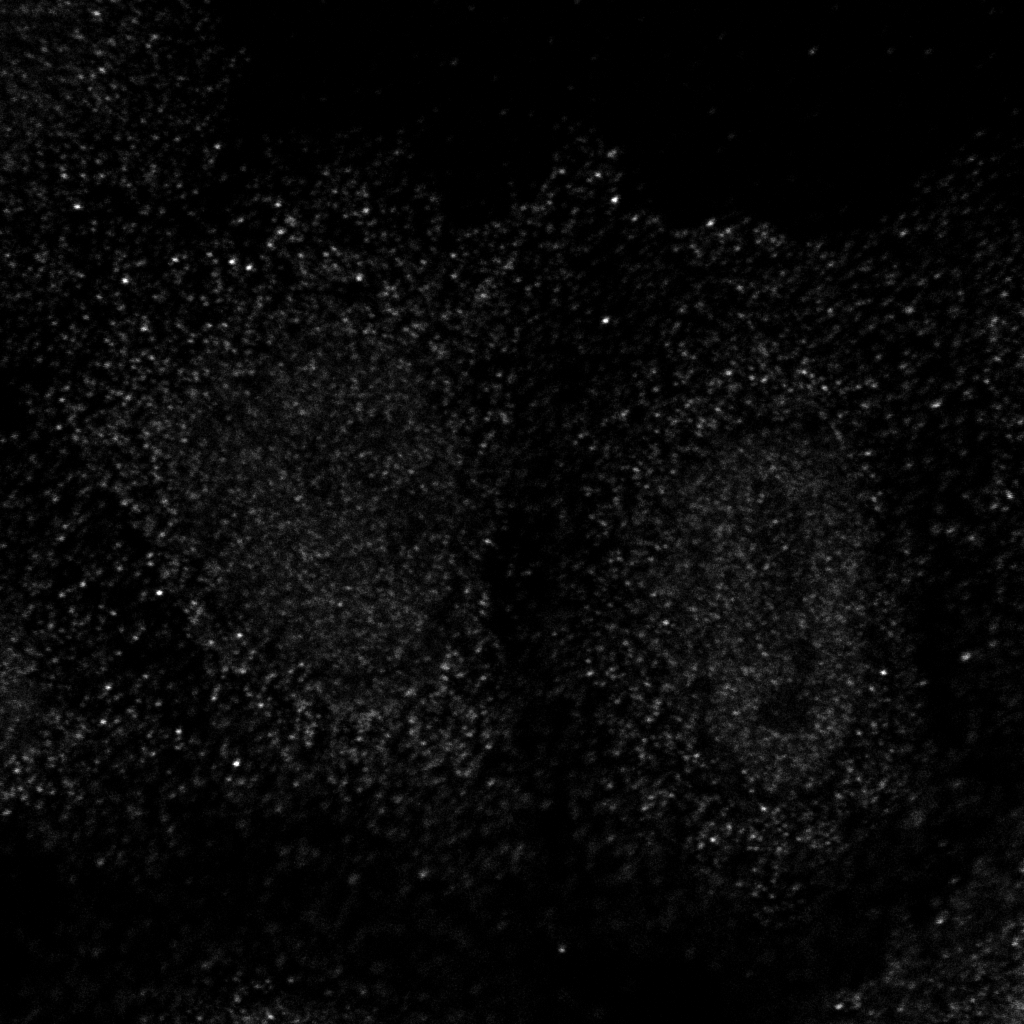

Supplement: Supplementary file 4 — Source data Fig. 2 [file 44318_2025_672_MOESM4_ESM.zip › Figure 2/2A/4KO_VEH_Gal3.tif]

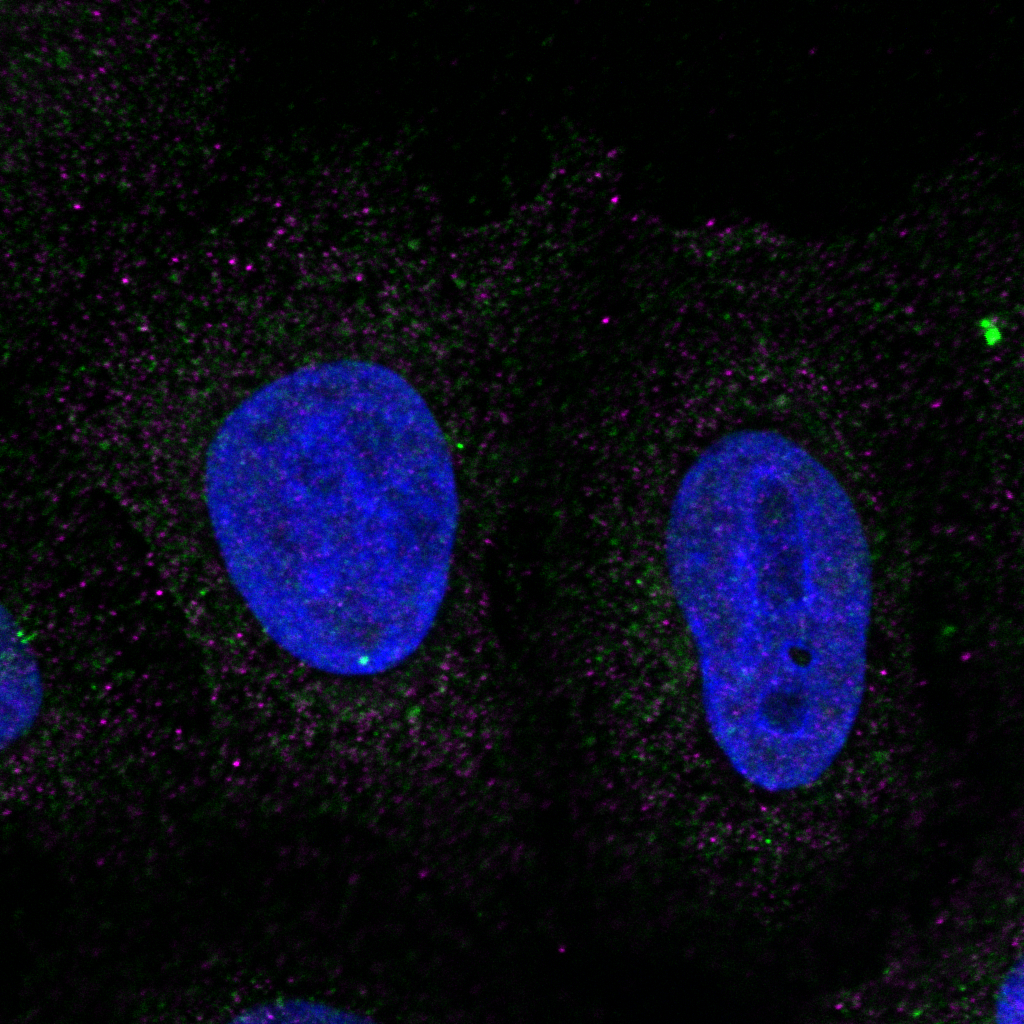

Supplement: Supplementary file 4 — Source data Fig. 2 [file 44318_2025_672_MOESM4_ESM.zip › Figure 2/2A/4KO_VEH_merge.tif]

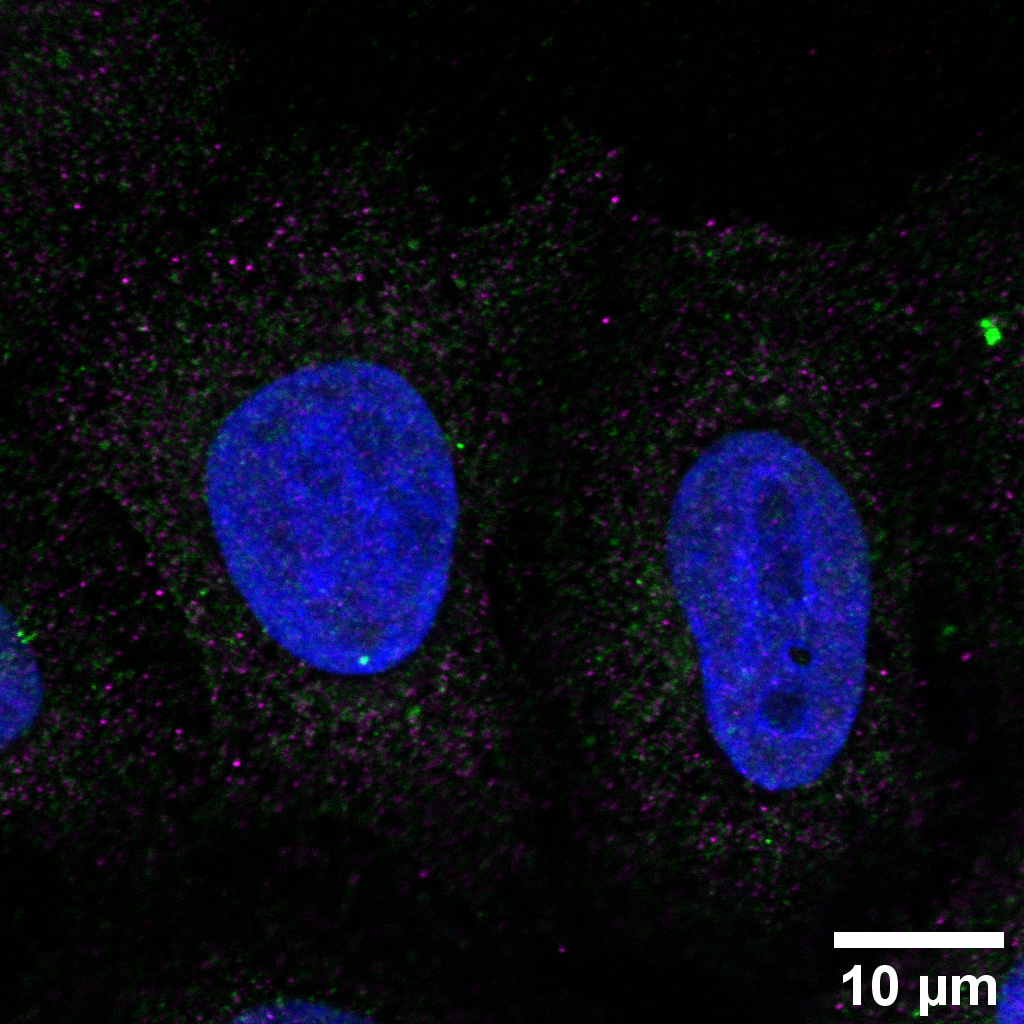

Supplement: Supplementary file 4 — Source data Fig. 2 [file 44318_2025_672_MOESM4_ESM.zip › Figure 2/2A/4KO_VEH_scale.tif]

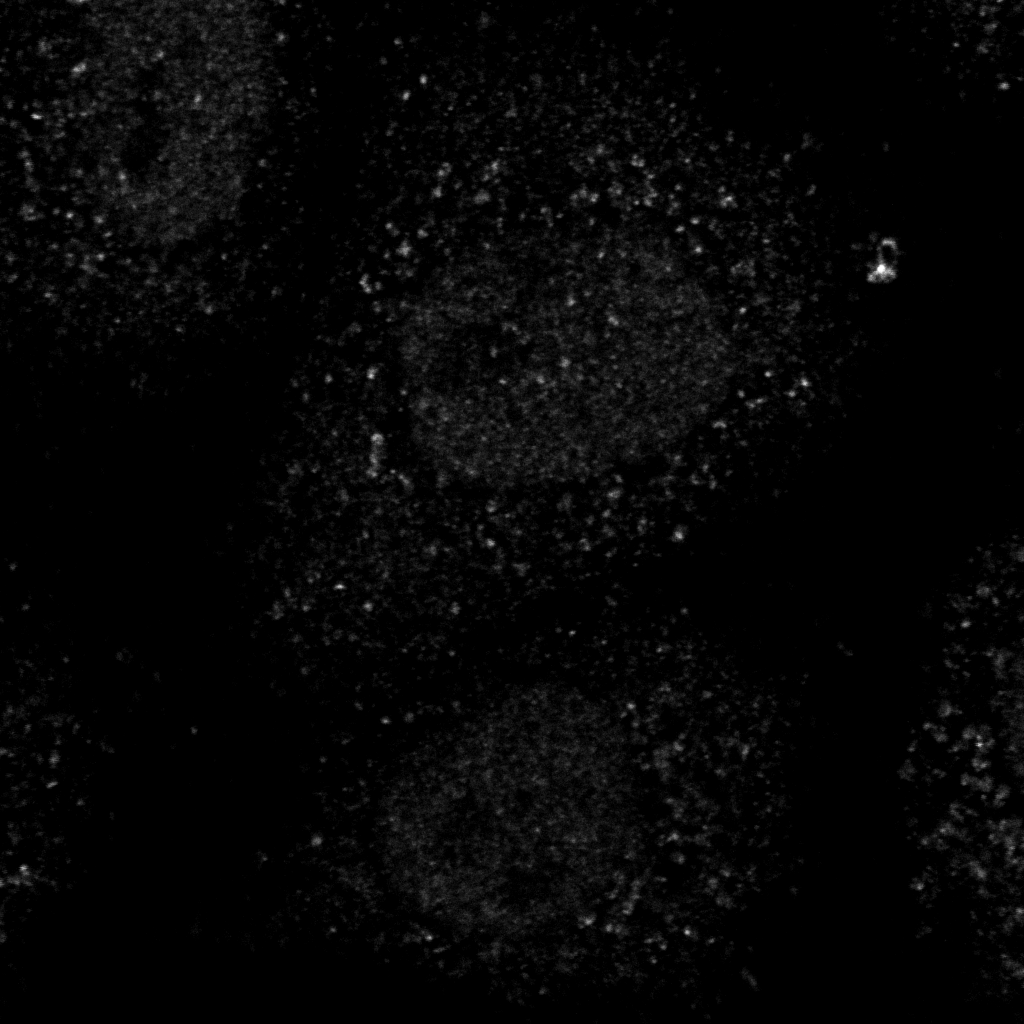

Supplement: Supplementary file 4 — Source data Fig. 2 [file 44318_2025_672_MOESM4_ESM.zip › Figure 2/2A/5KO_LLOMe_ALIX.tif]

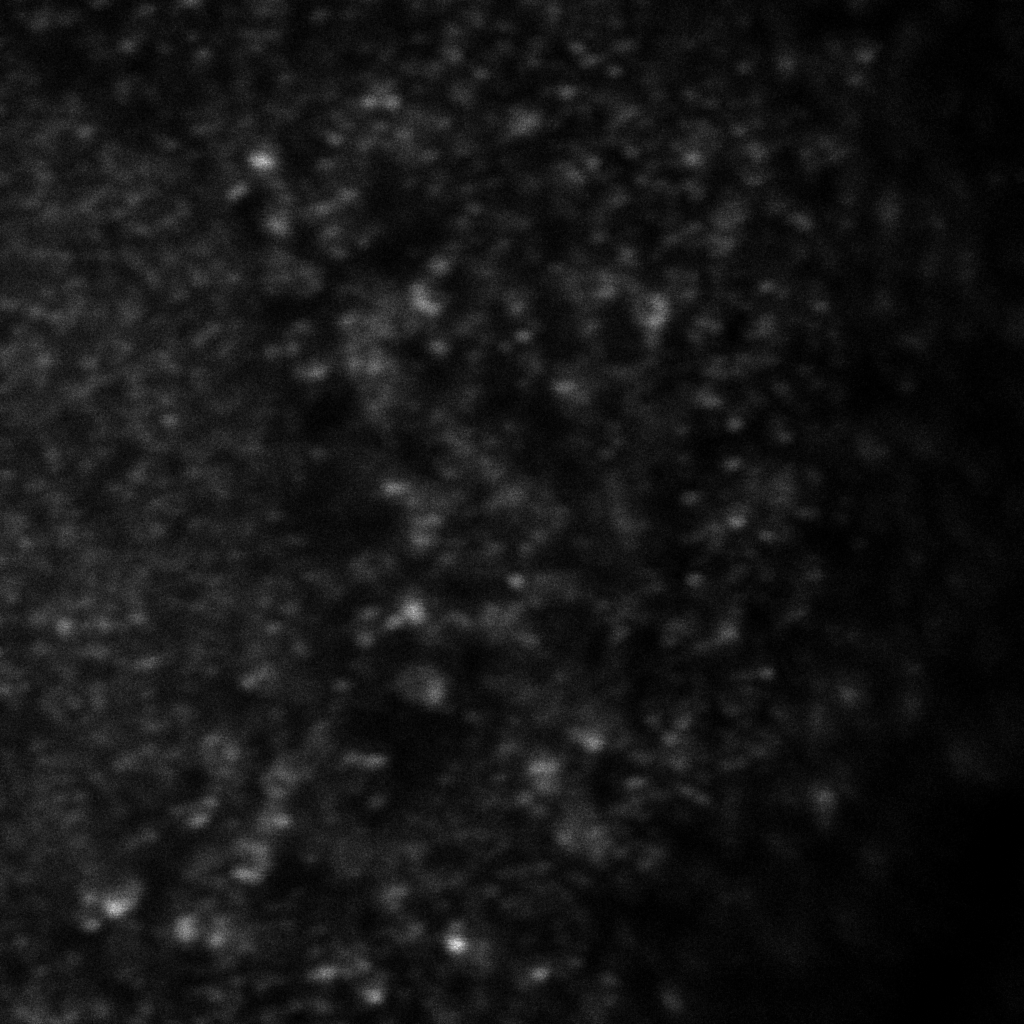

Supplement: Supplementary file 4 — Source data Fig. 2 [file 44318_2025_672_MOESM4_ESM.zip › Figure 2/2A/5KO_LLOMe_ALIX_zoom.tif]

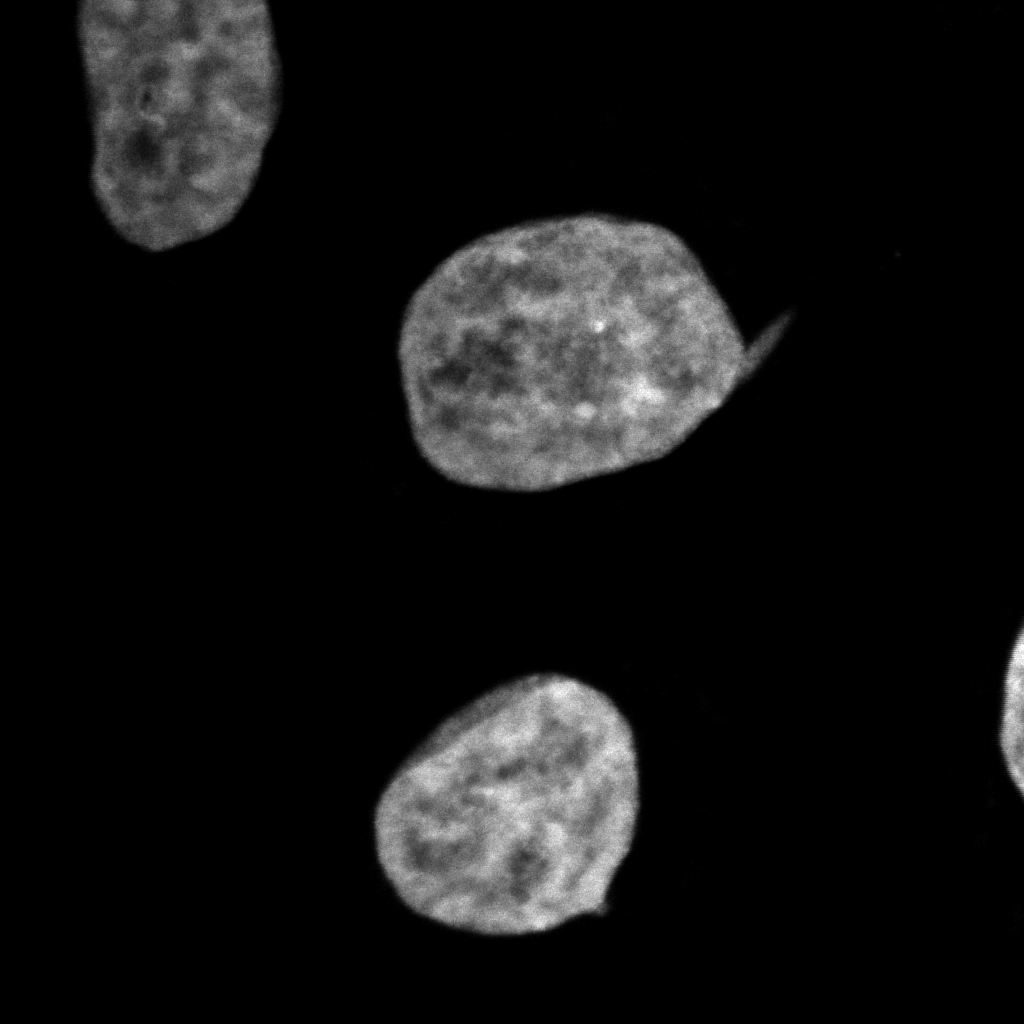

Supplement: Supplementary file 4 — Source data Fig. 2 [file 44318_2025_672_MOESM4_ESM.zip › Figure 2/2A/5KO_LLOMe_DAPI.tif]

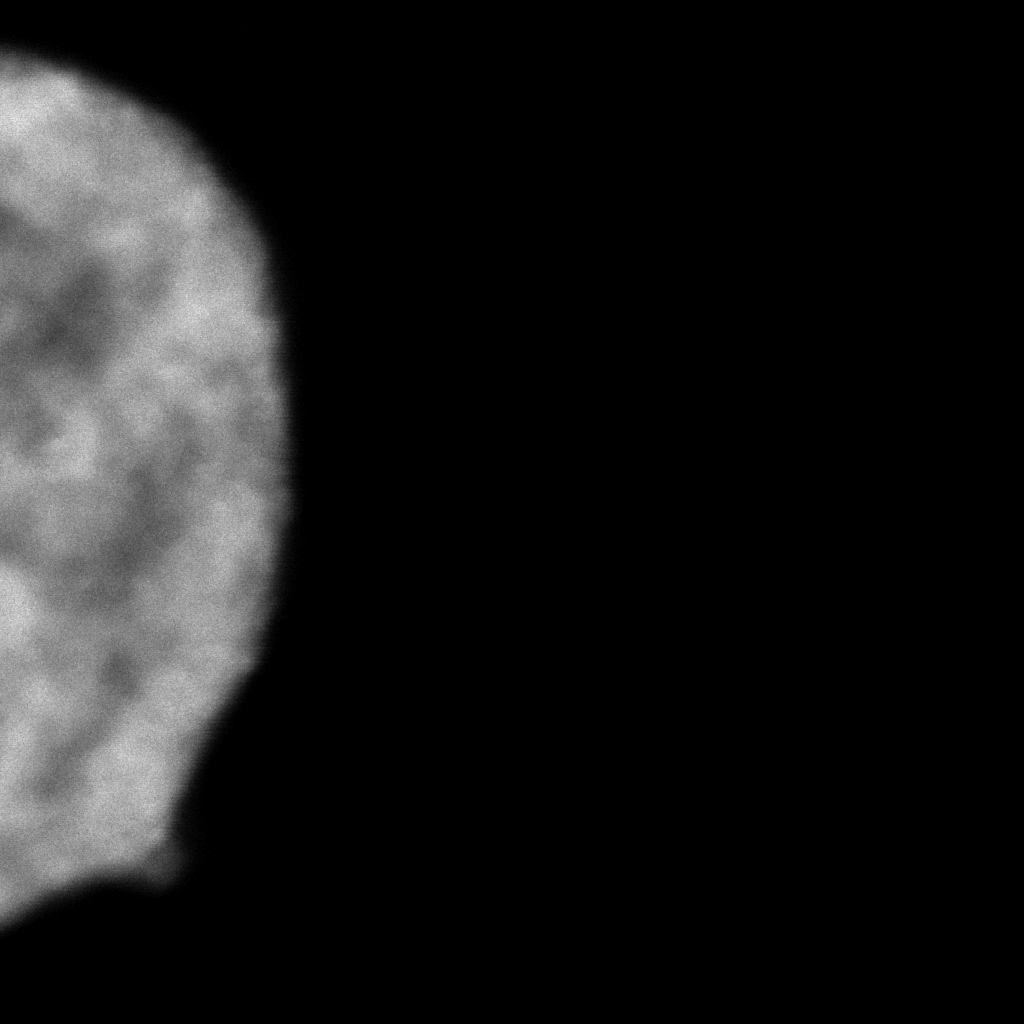

Supplement: Supplementary file 4 — Source data Fig. 2 [file 44318_2025_672_MOESM4_ESM.zip › Figure 2/2A/5KO_LLOMe_DAPI_zoom.tif]

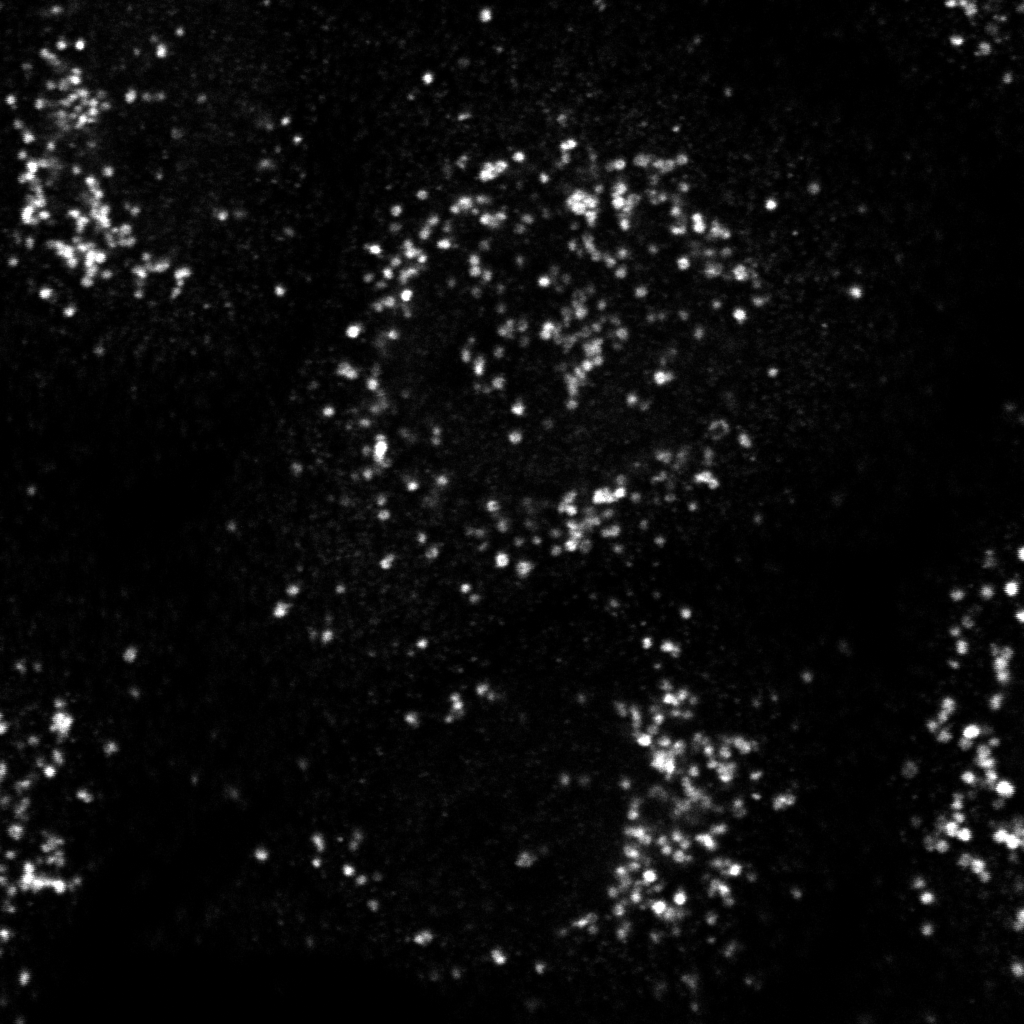

Supplement: Supplementary file 4 — Source data Fig. 2 [file 44318_2025_672_MOESM4_ESM.zip › Figure 2/2A/5KO_LLOMe_Gal3.tif]

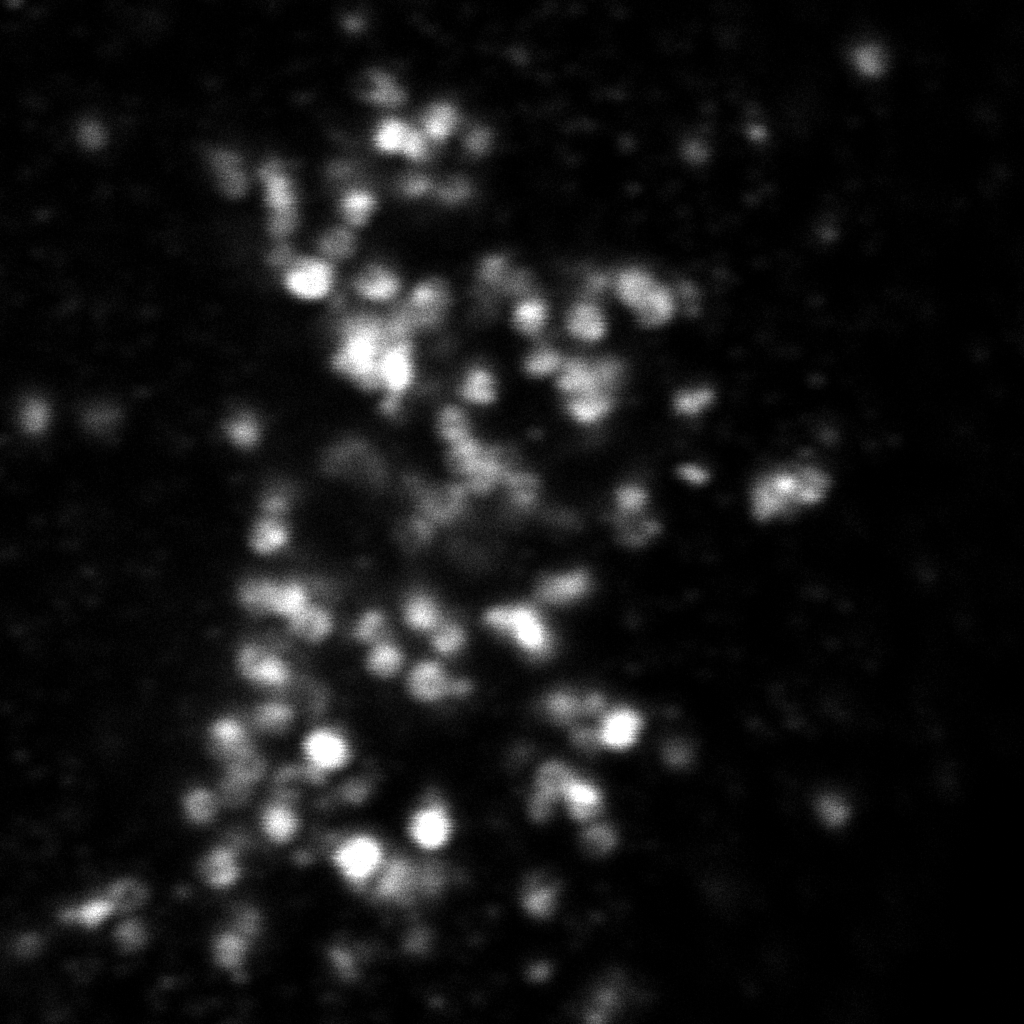

Supplement: Supplementary file 4 — Source data Fig. 2 [file 44318_2025_672_MOESM4_ESM.zip › Figure 2/2A/5KO_LLOMe_Gal3_zoom.tif]

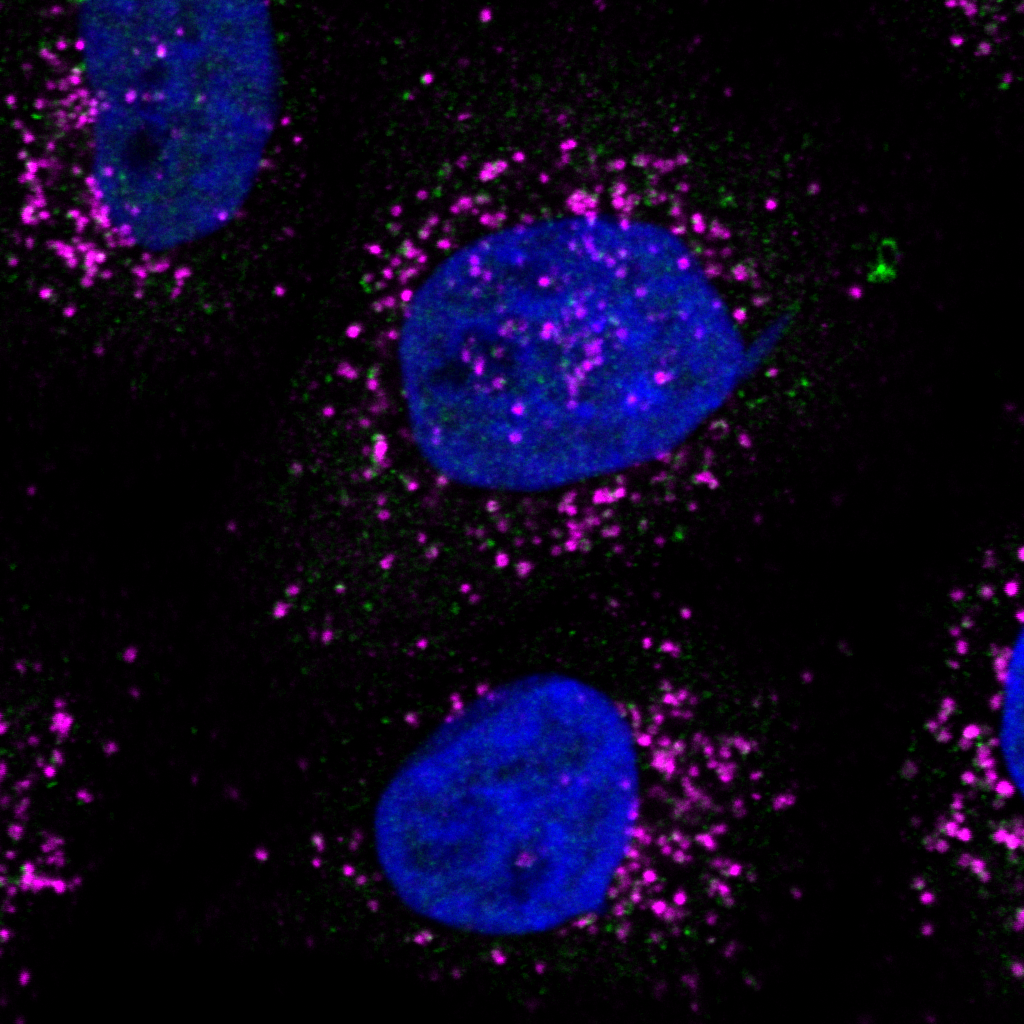

Supplement: Supplementary file 4 — Source data Fig. 2 [file 44318_2025_672_MOESM4_ESM.zip › Figure 2/2A/5KO_LLOMe_merge.tif]

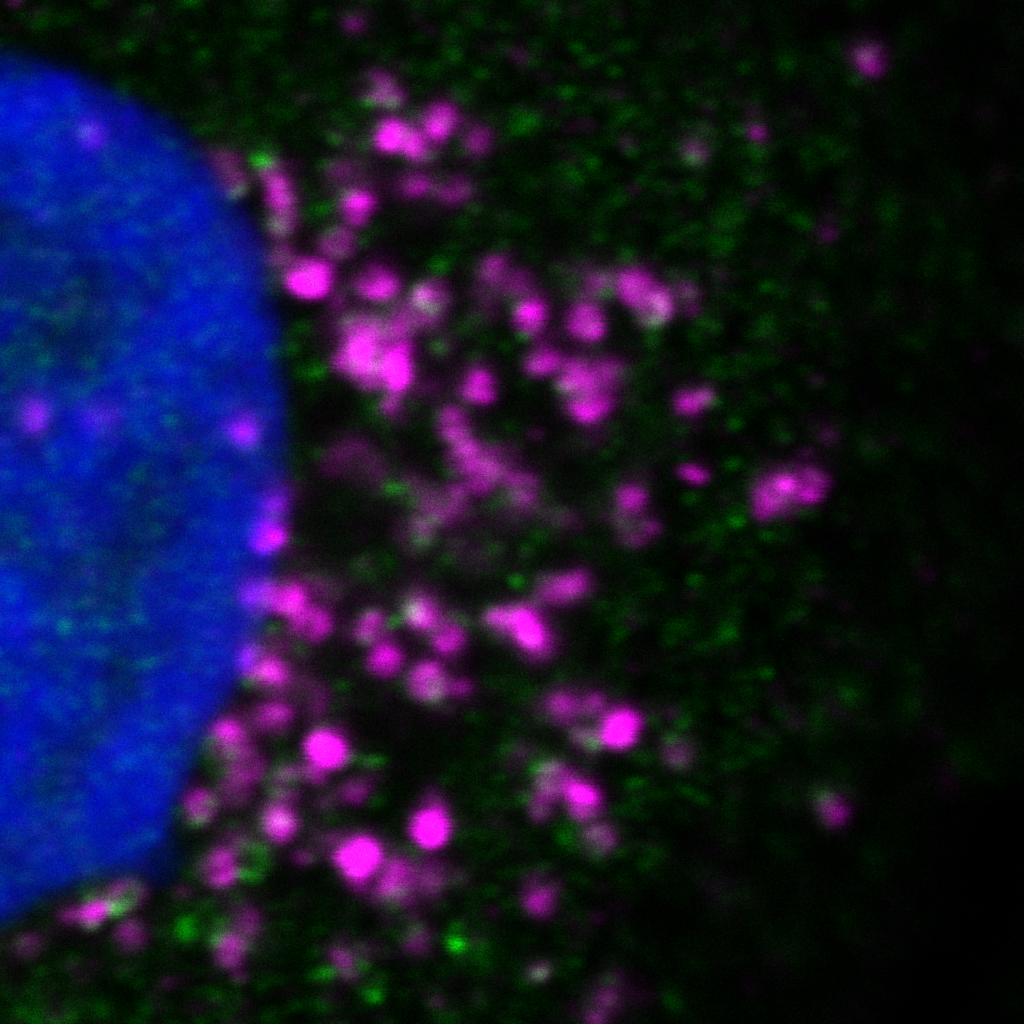

Supplement: Supplementary file 4 — Source data Fig. 2 [file 44318_2025_672_MOESM4_ESM.zip › Figure 2/2A/5KO_LLOMe_merge_zoom.tif]

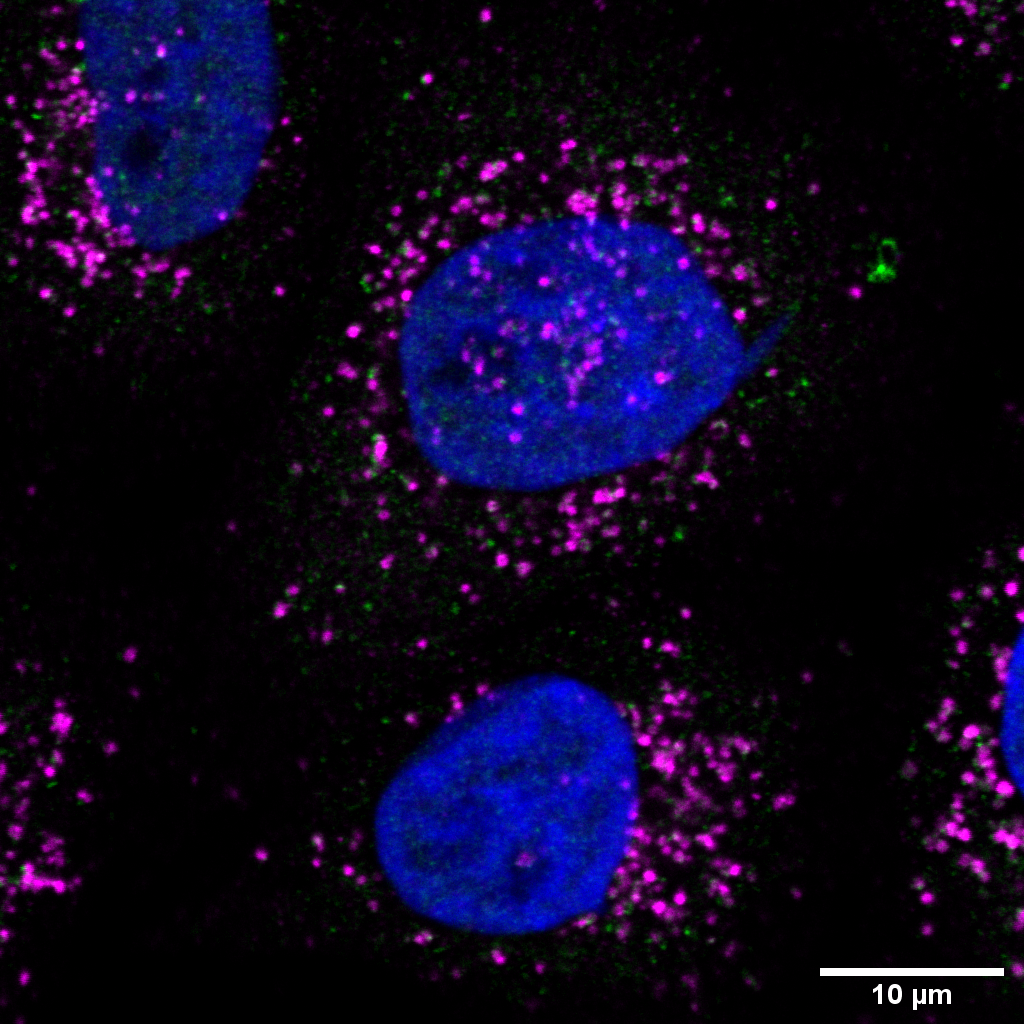

Supplement: Supplementary file 4 — Source data Fig. 2 [file 44318_2025_672_MOESM4_ESM.zip › Figure 2/2A/5KO_LLOMe_scale.tif]

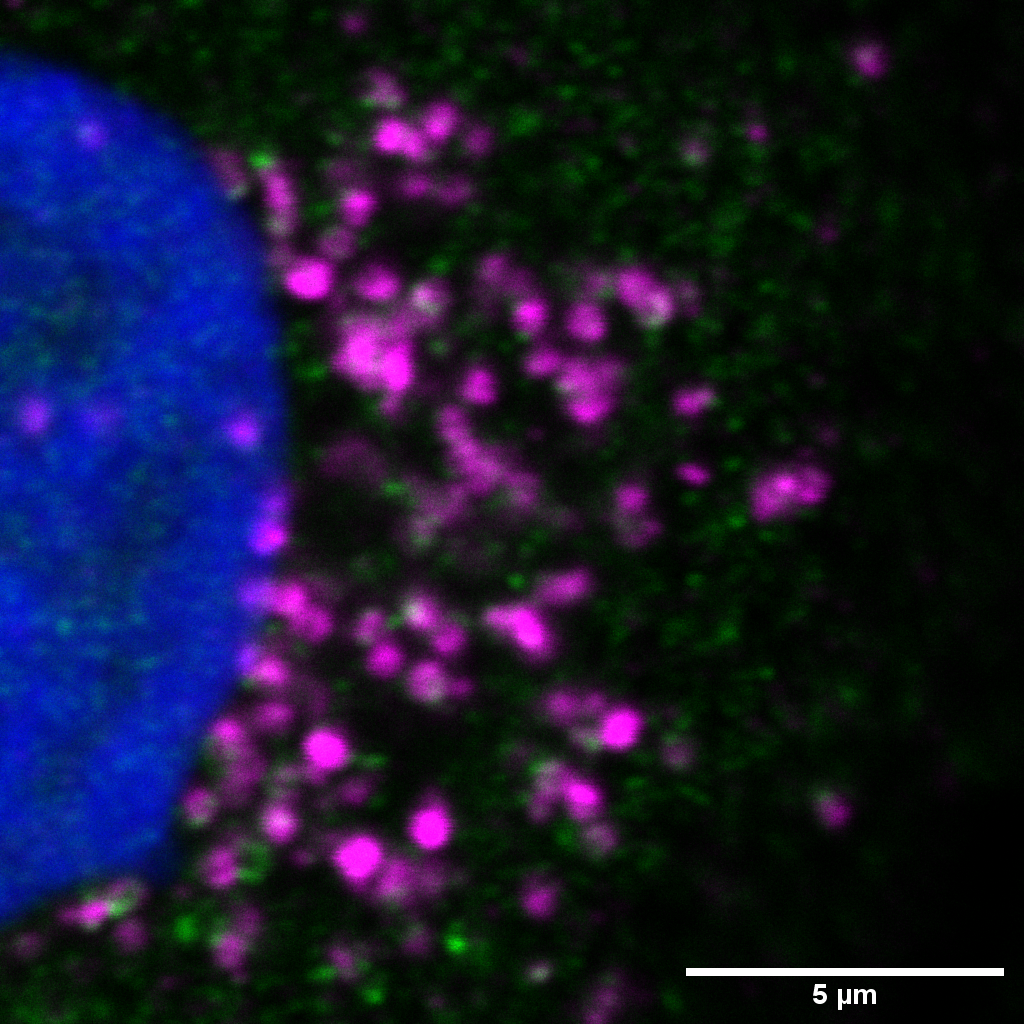

Supplement: Supplementary file 4 — Source data Fig. 2 [file 44318_2025_672_MOESM4_ESM.zip › Figure 2/2A/5KO_LLOMe_scale_zoom.tif]

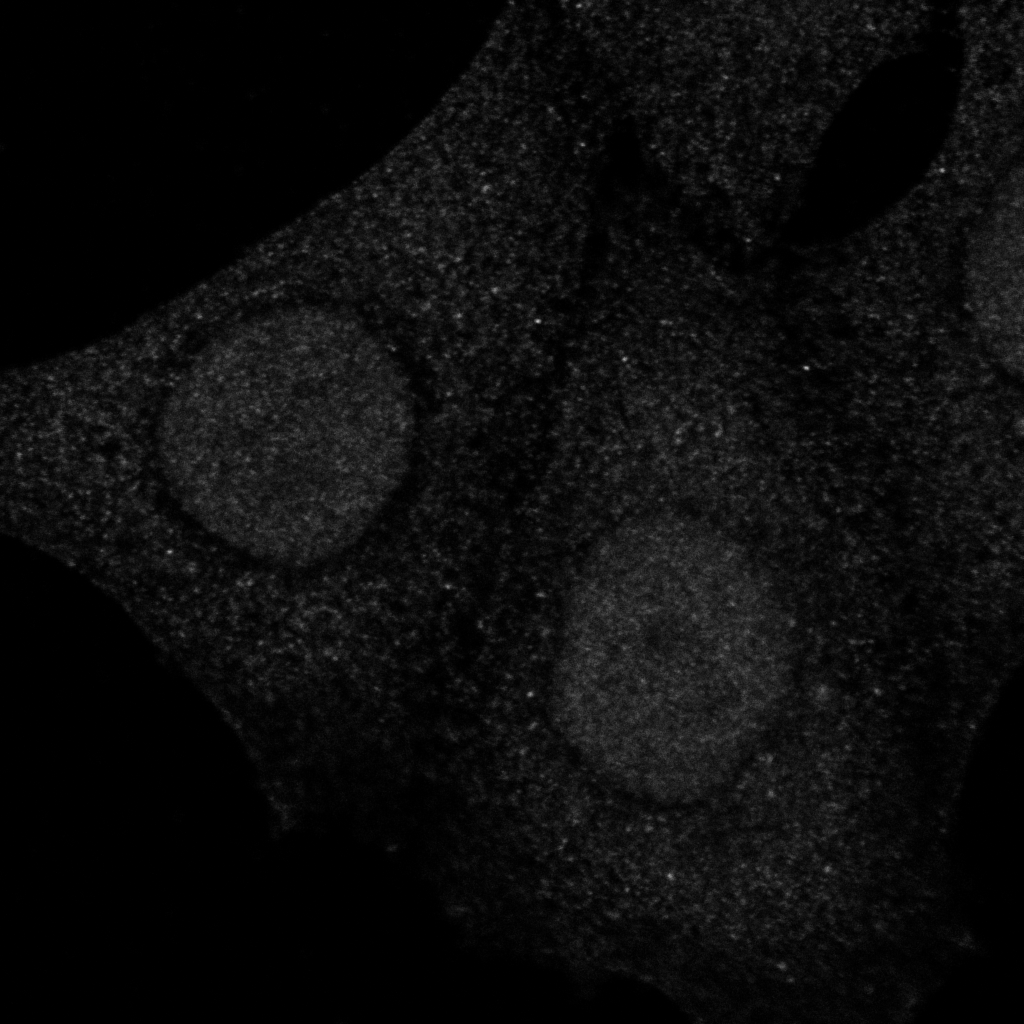

Supplement: Supplementary file 4 — Source data Fig. 2 [file 44318_2025_672_MOESM4_ESM.zip › Figure 2/2A/5KO_VEH_ALIX.tif]

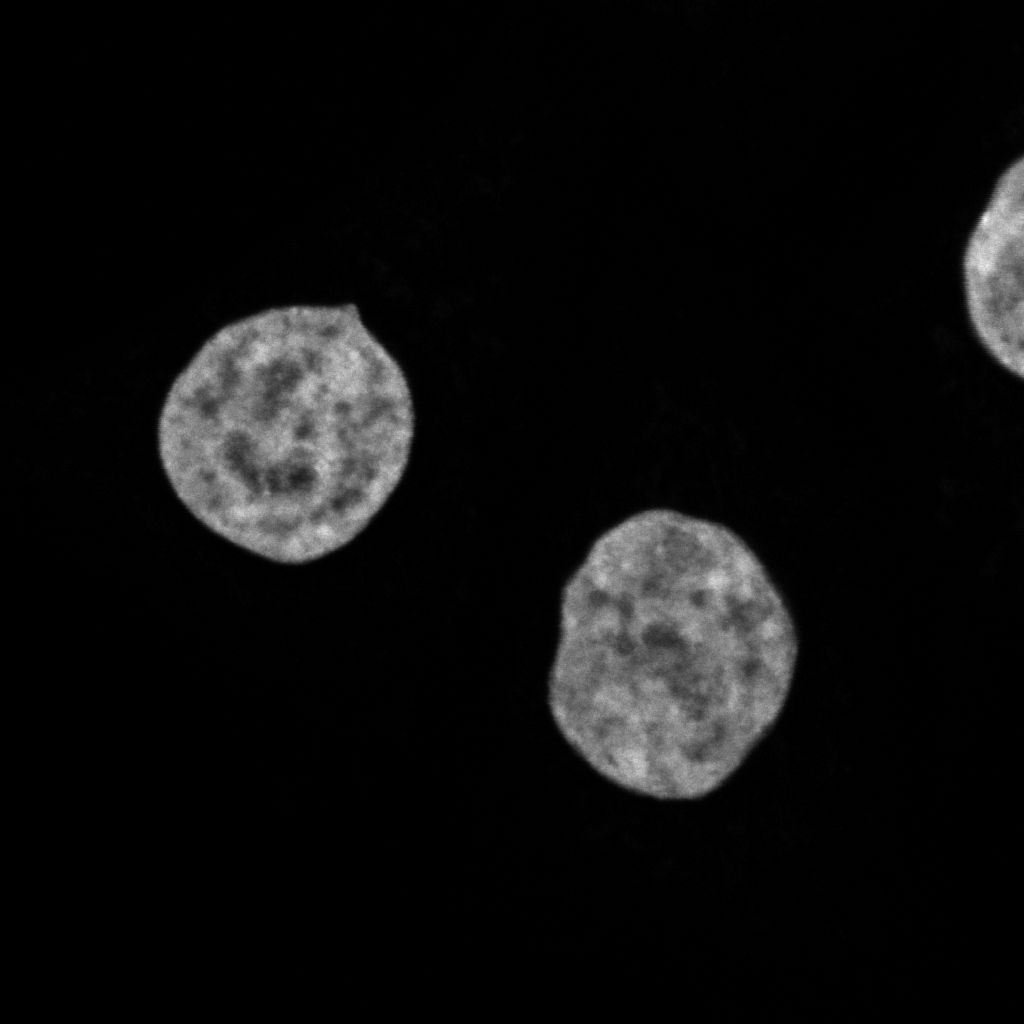

Supplement: Supplementary file 4 — Source data Fig. 2 [file 44318_2025_672_MOESM4_ESM.zip › Figure 2/2A/5KO_VEH_DAPI.tif]

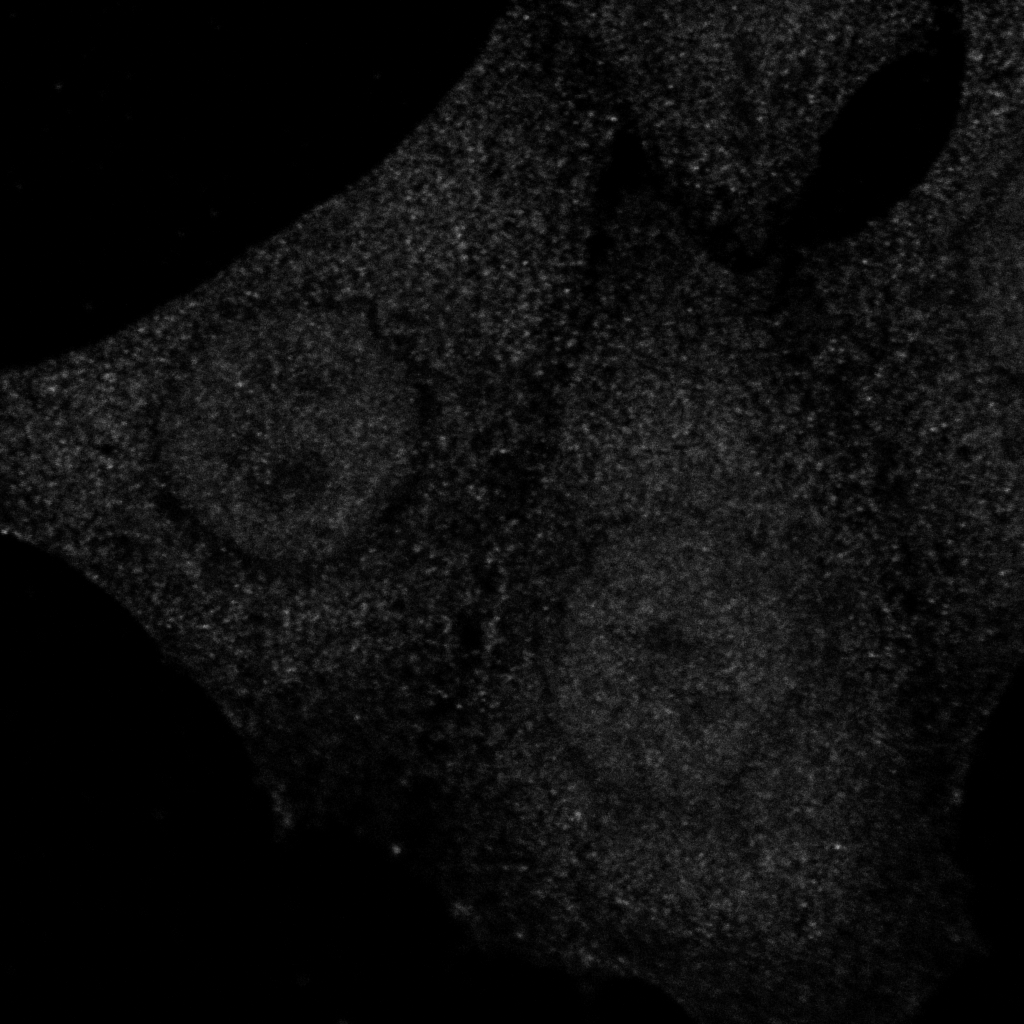

Supplement: Supplementary file 4 — Source data Fig. 2 [file 44318_2025_672_MOESM4_ESM.zip › Figure 2/2A/5KO_VEH_Gal3.tif]

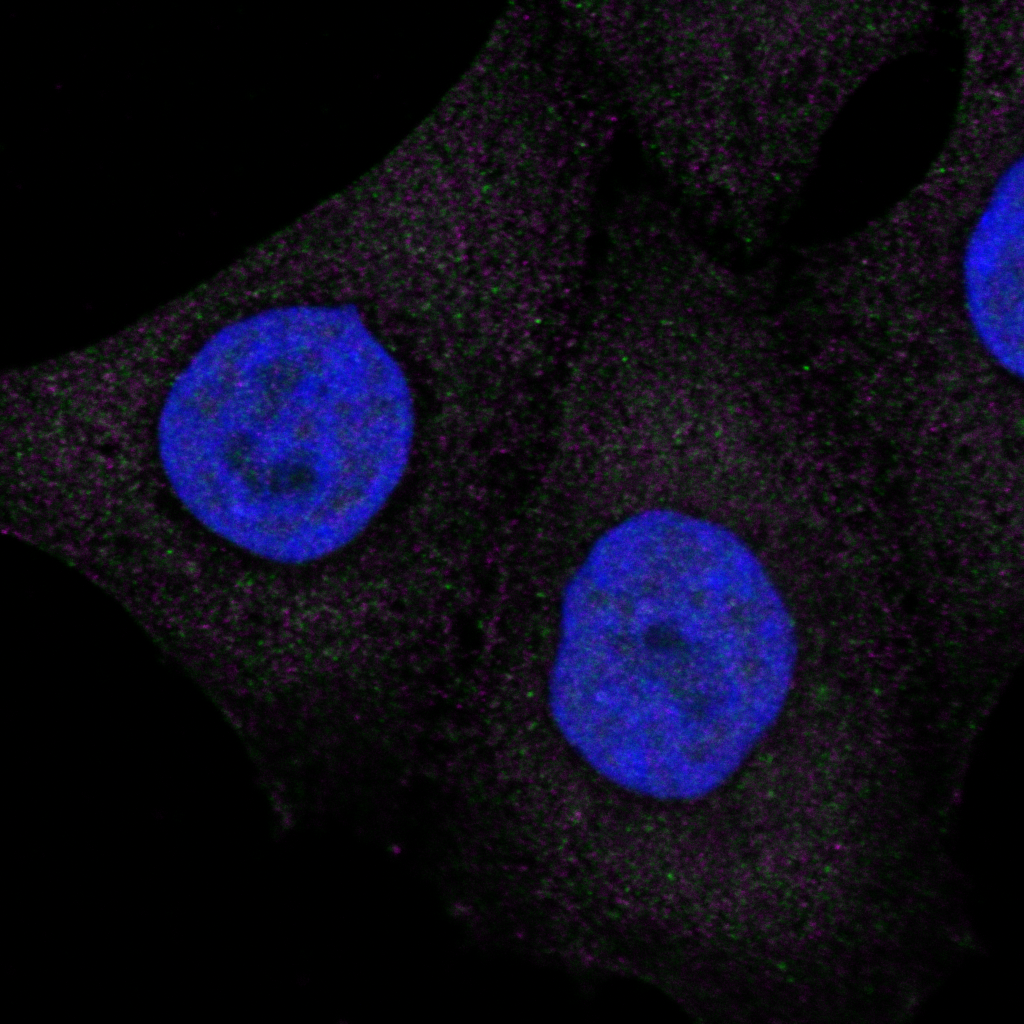

Supplement: Supplementary file 4 — Source data Fig. 2 [file 44318_2025_672_MOESM4_ESM.zip › Figure 2/2A/5KO_VEH_merge.tif]

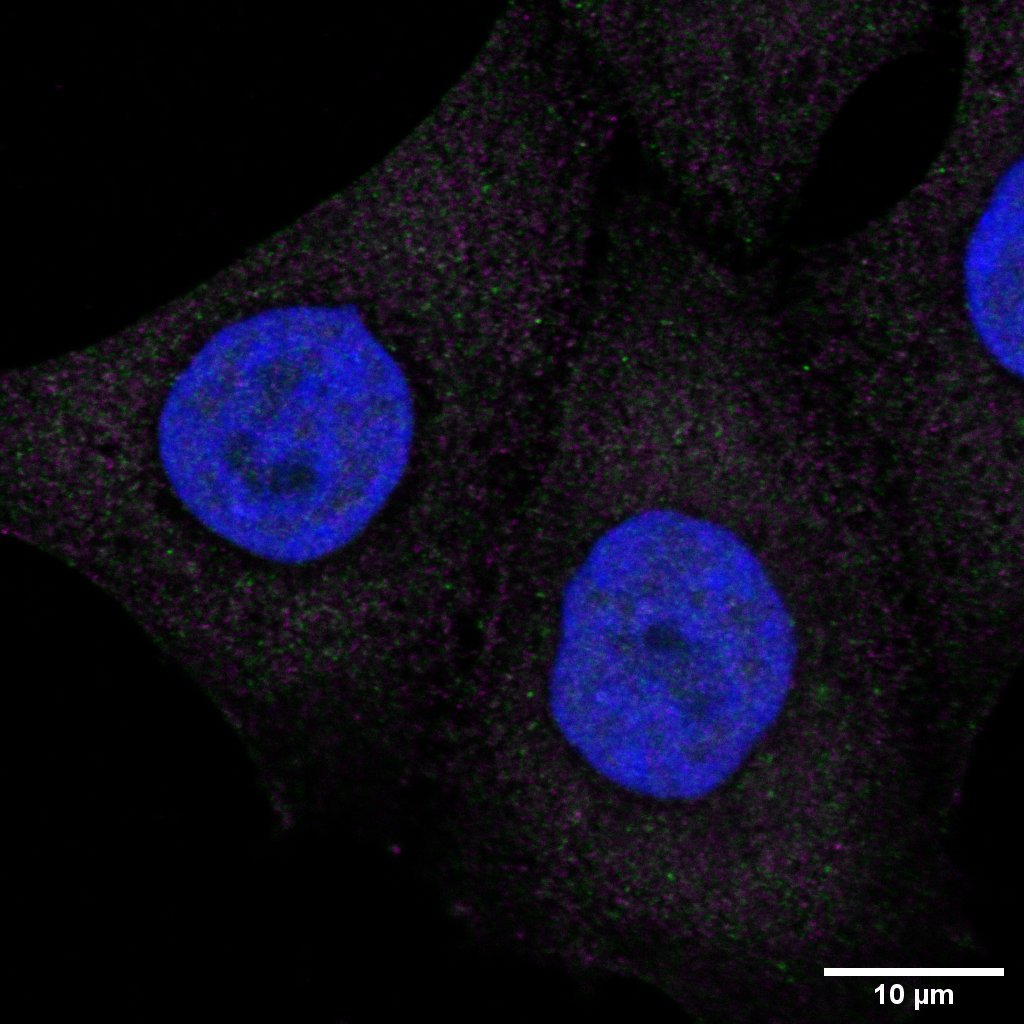

Supplement: Supplementary file 4 — Source data Fig. 2 [file 44318_2025_672_MOESM4_ESM.zip › Figure 2/2A/5KO_VEH_scale.tif]

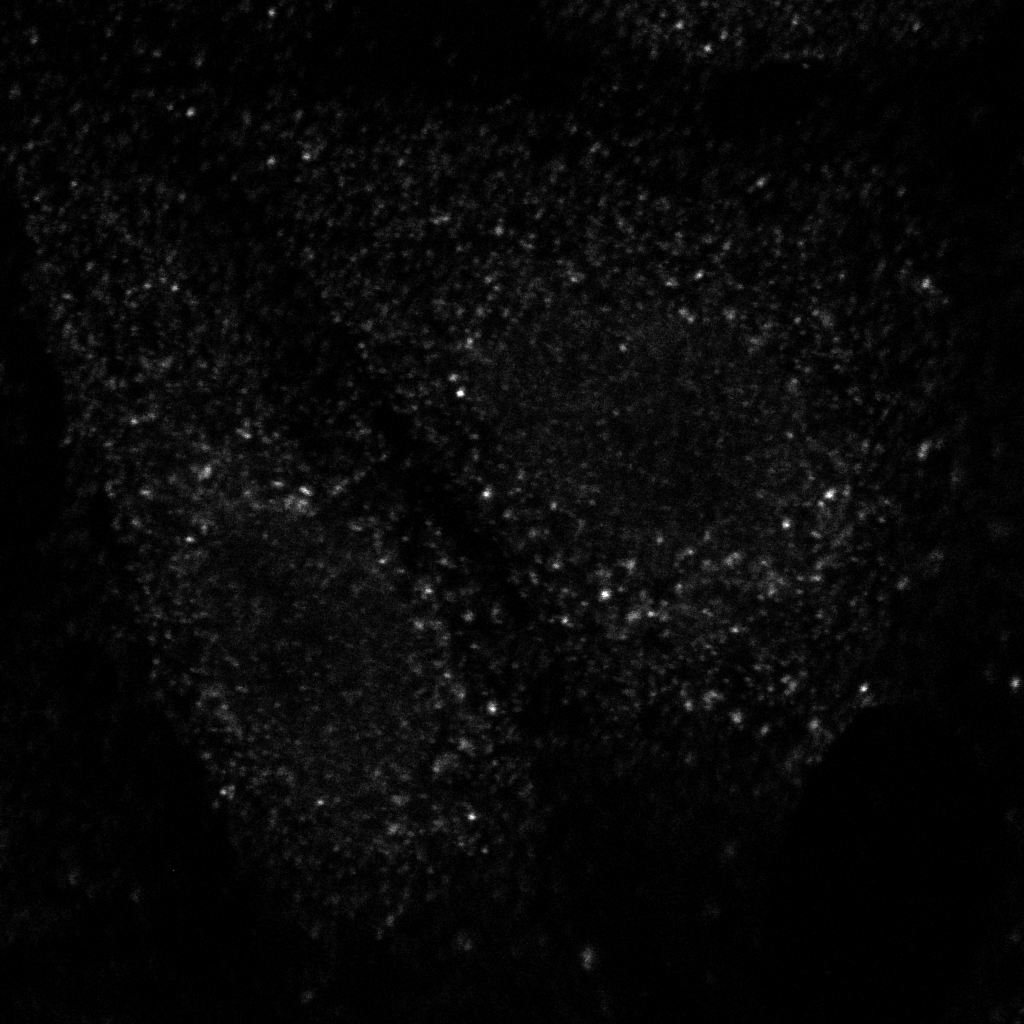

Supplement: Supplementary file 4 — Source data Fig. 2 [file 44318_2025_672_MOESM4_ESM.zip › Figure 2/2A/7KO_LLOMe_ALIX.tif]

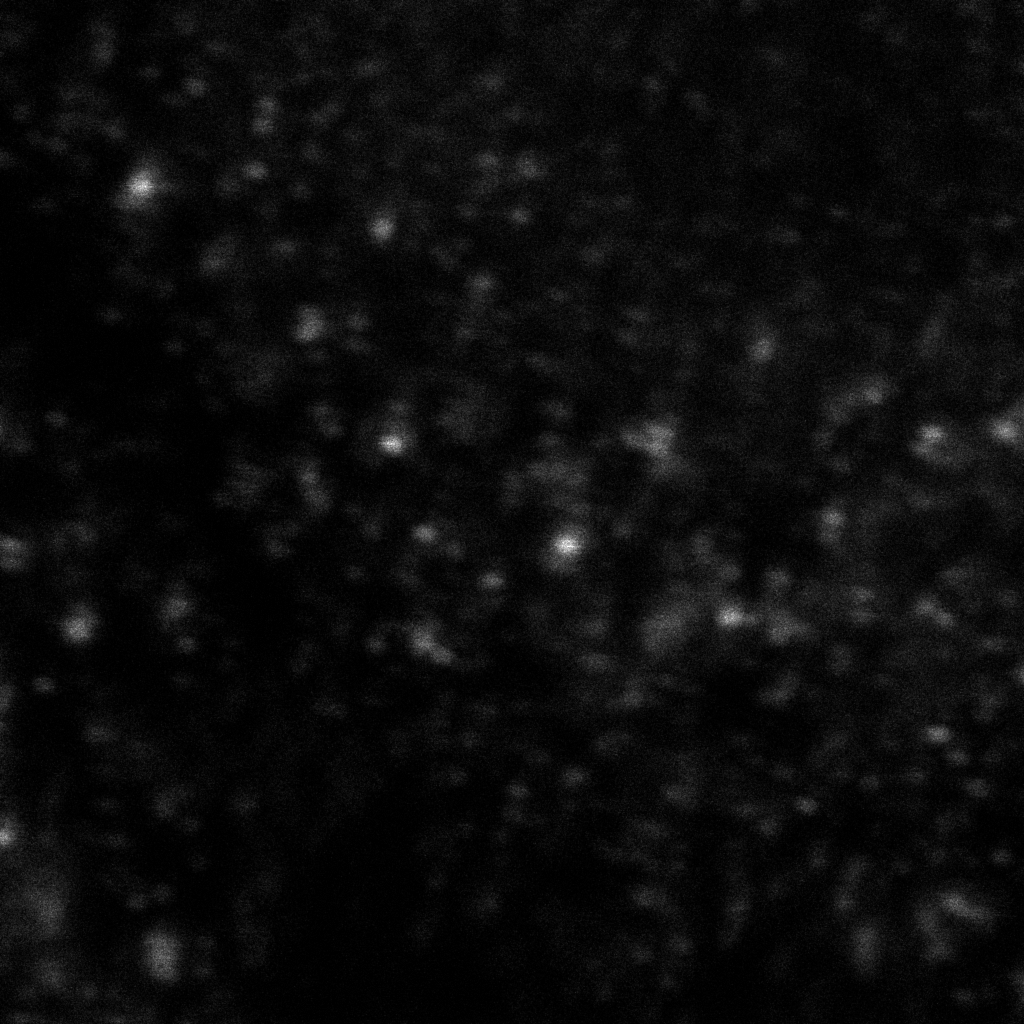

Supplement: Supplementary file 4 — Source data Fig. 2 [file 44318_2025_672_MOESM4_ESM.zip › Figure 2/2A/7KO_LLOMe_ALIX_zoom.tif]

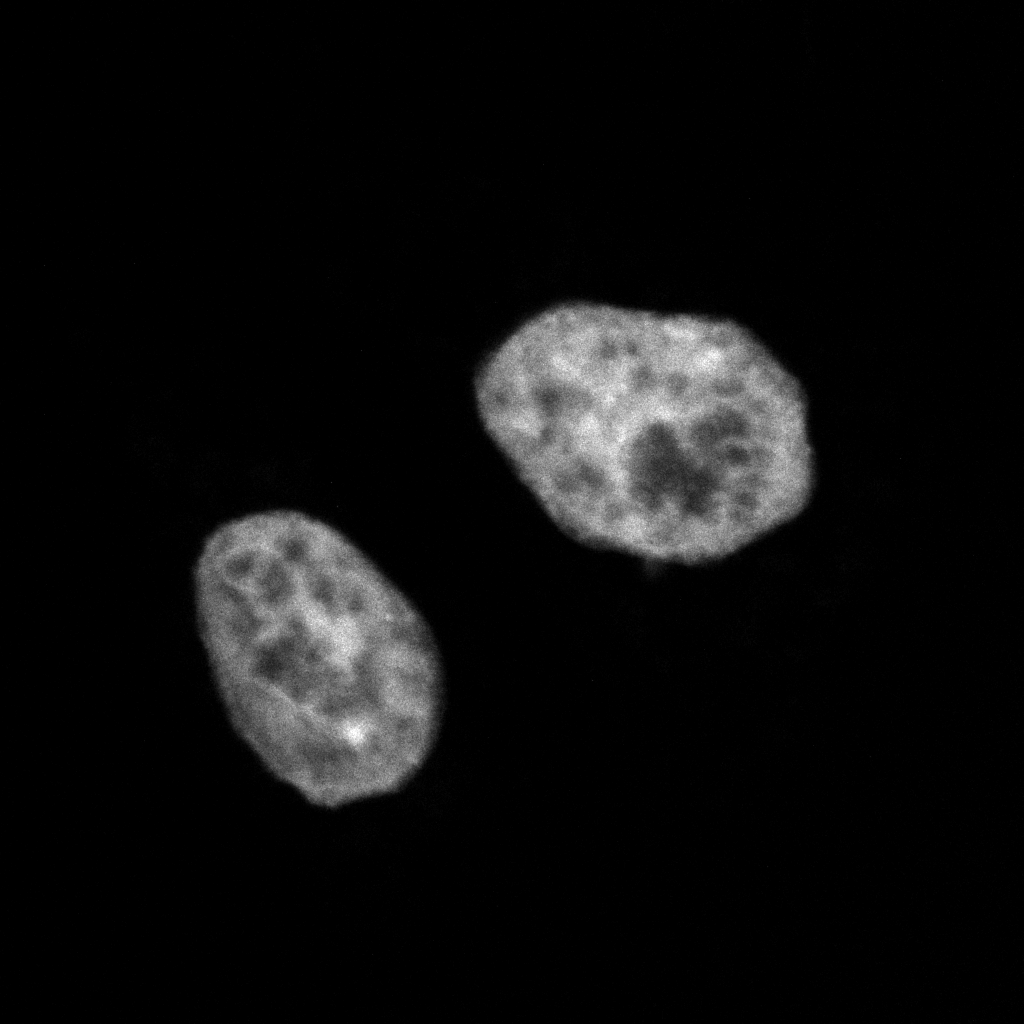

Supplement: Supplementary file 4 — Source data Fig. 2 [file 44318_2025_672_MOESM4_ESM.zip › Figure 2/2A/7KO_LLOMe_DAPI.tif]

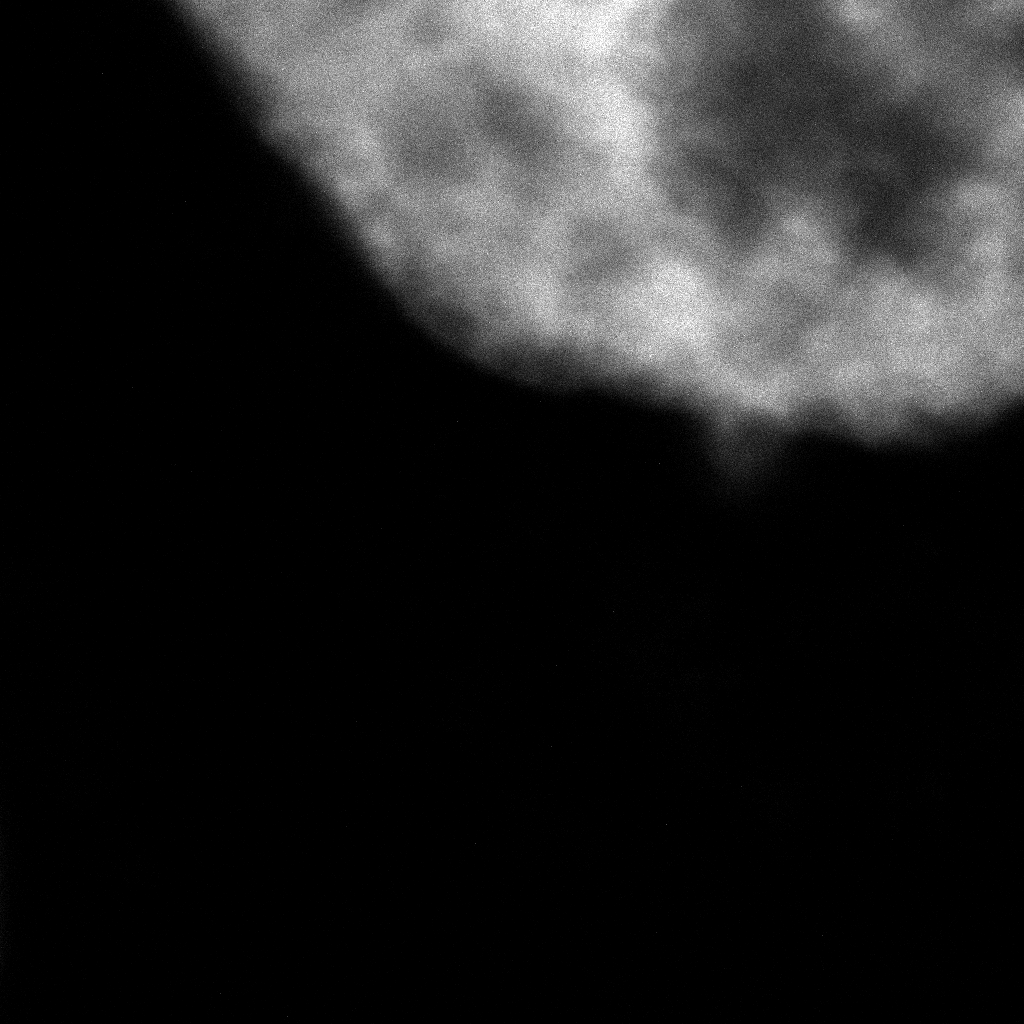

Supplement: Supplementary file 4 — Source data Fig. 2 [file 44318_2025_672_MOESM4_ESM.zip › Figure 2/2A/7KO_LLOMe_DAPI_zoom.tif]

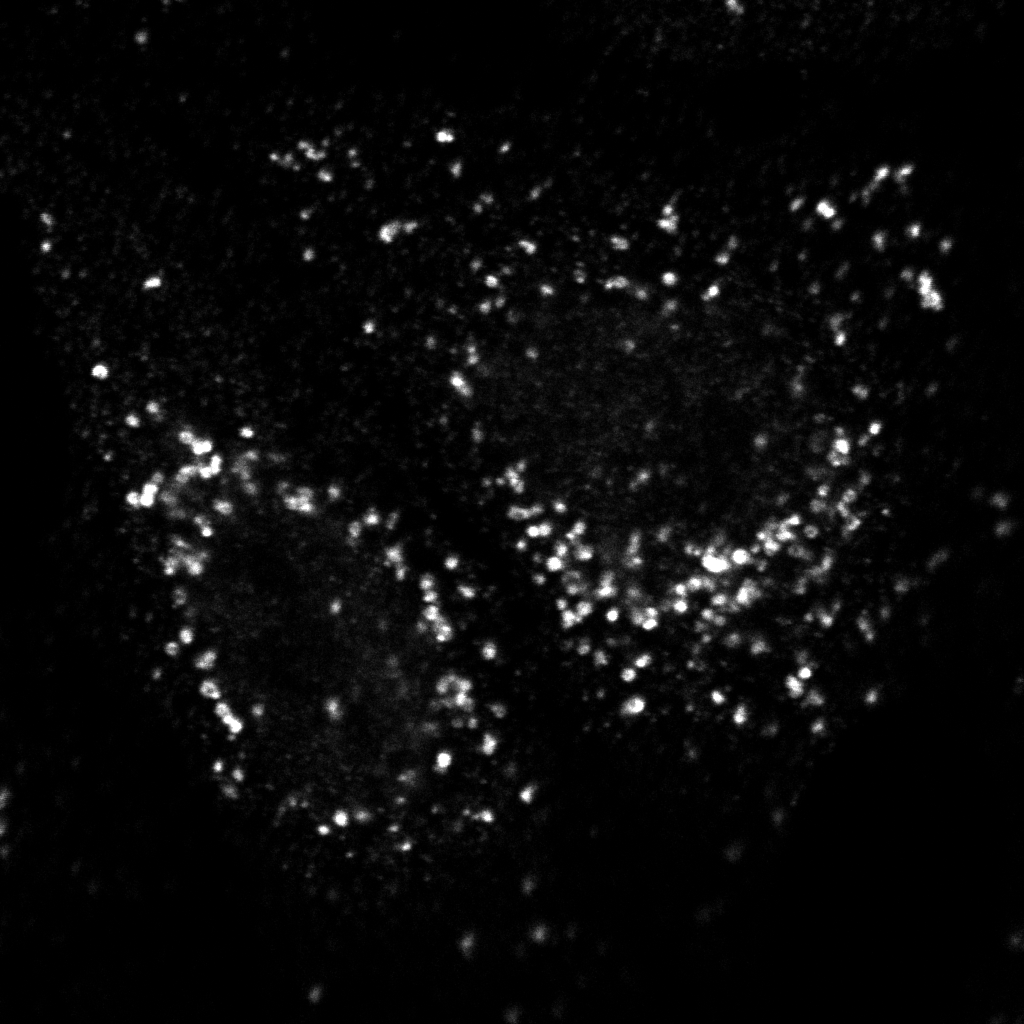

Supplement: Supplementary file 4 — Source data Fig. 2 [file 44318_2025_672_MOESM4_ESM.zip › Figure 2/2A/7KO_LLOMe_Gal3.tif]

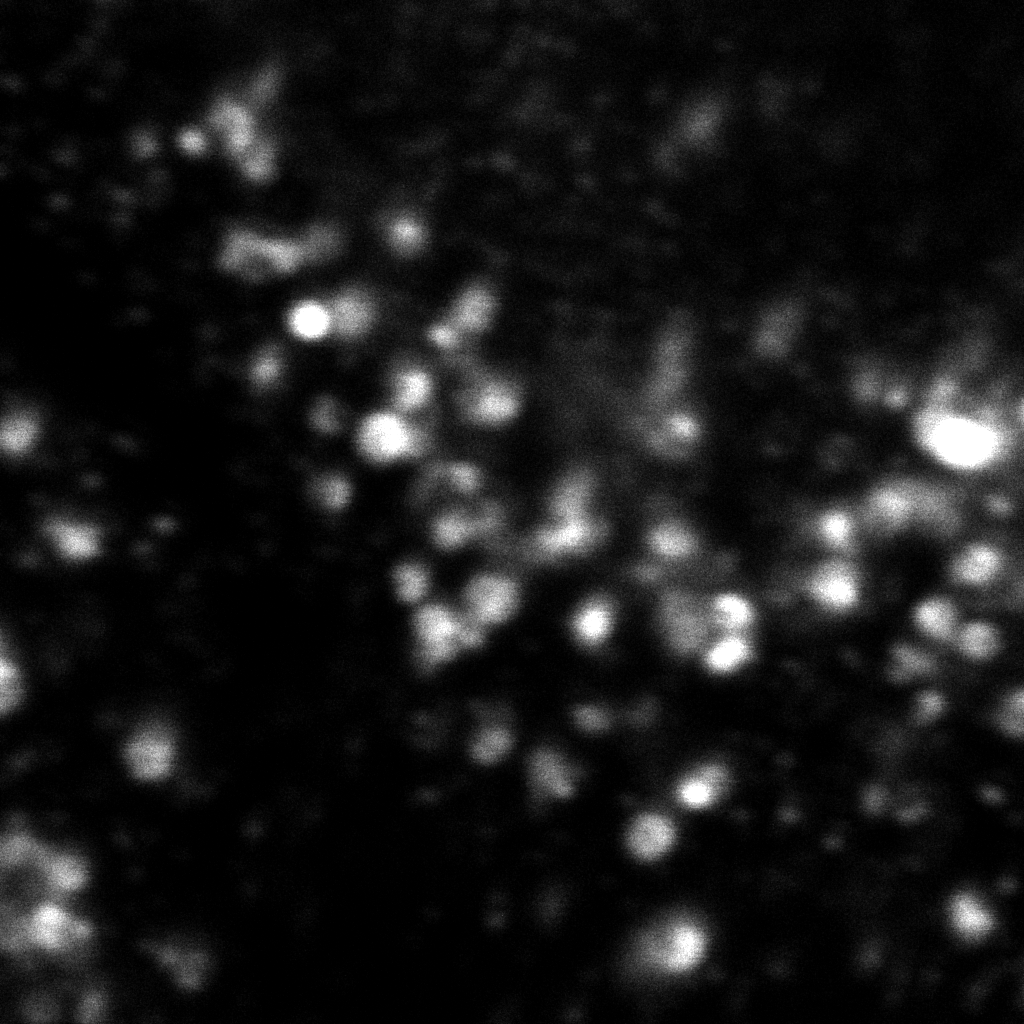

Supplement: Supplementary file 4 — Source data Fig. 2 [file 44318_2025_672_MOESM4_ESM.zip › Figure 2/2A/7KO_LLOMe_Gal3_zoom.tif]

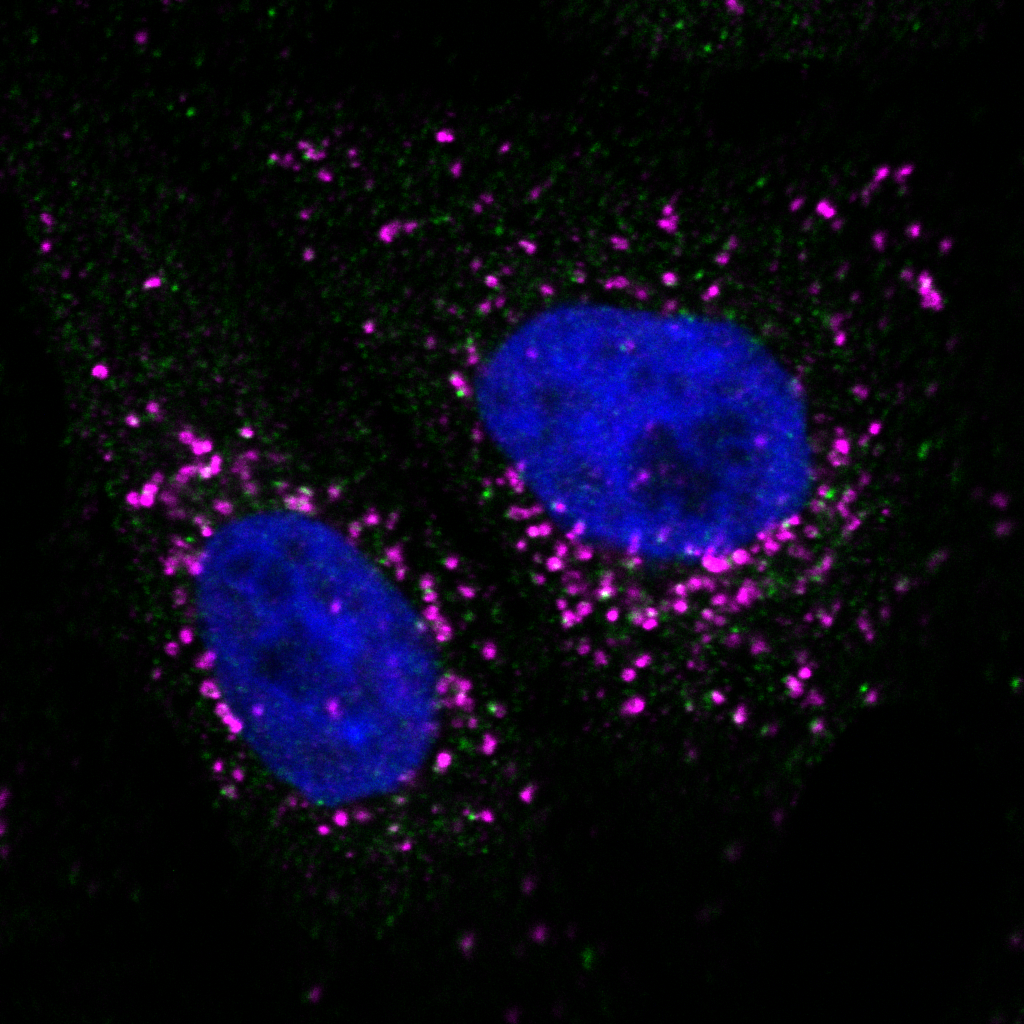

Supplement: Supplementary file 4 — Source data Fig. 2 [file 44318_2025_672_MOESM4_ESM.zip › Figure 2/2A/7KO_LLOMe_merge.tif]

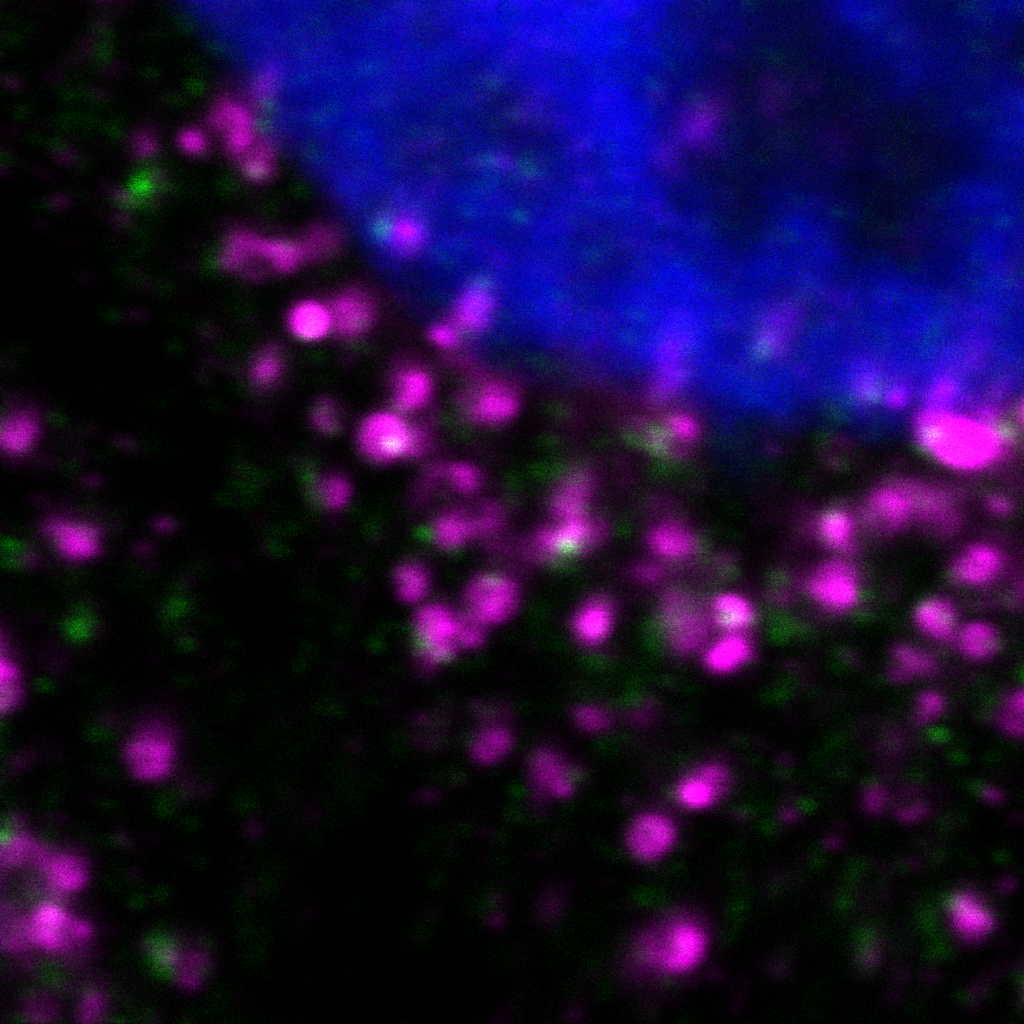

Supplement: Supplementary file 4 — Source data Fig. 2 [file 44318_2025_672_MOESM4_ESM.zip › Figure 2/2A/7KO_LLOMe_merge_zoom.tif]

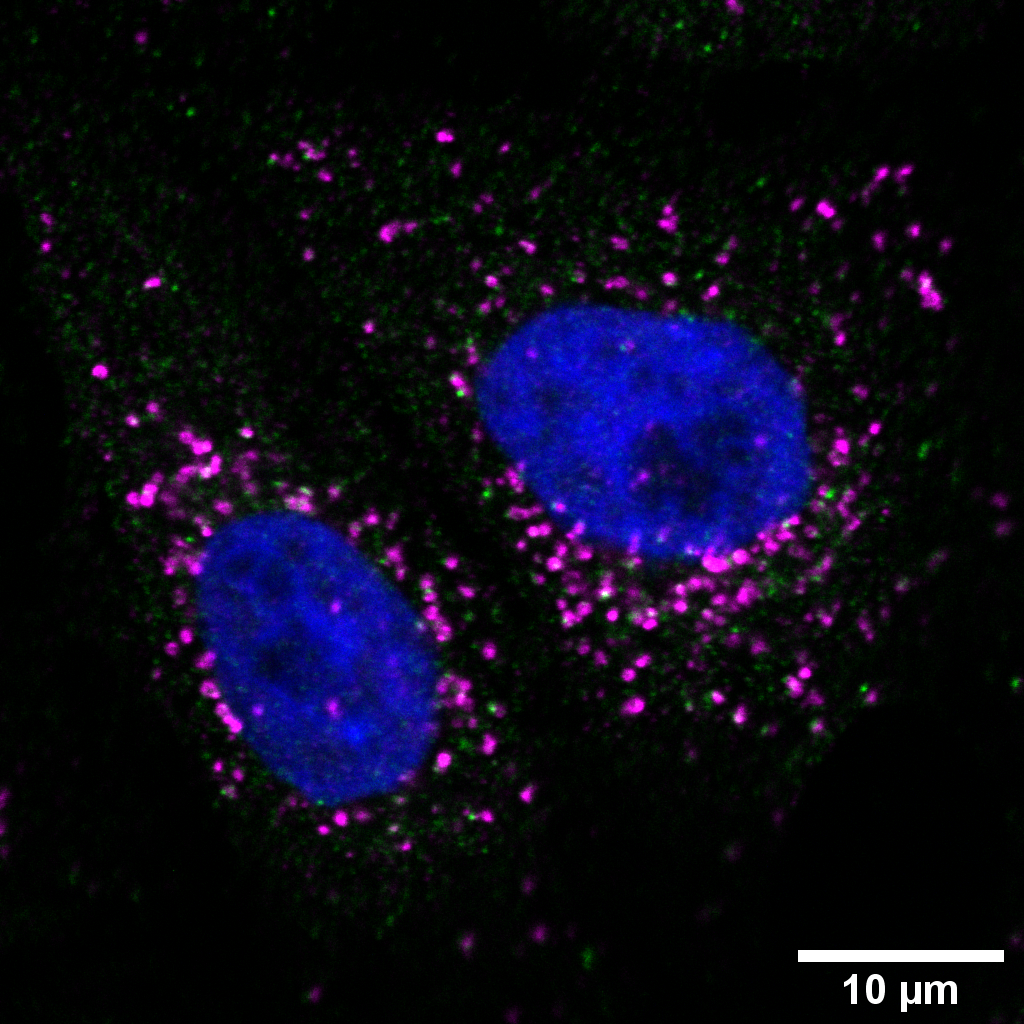

Supplement: Supplementary file 4 — Source data Fig. 2 [file 44318_2025_672_MOESM4_ESM.zip › Figure 2/2A/7KO_LLOMe_scale.tif]

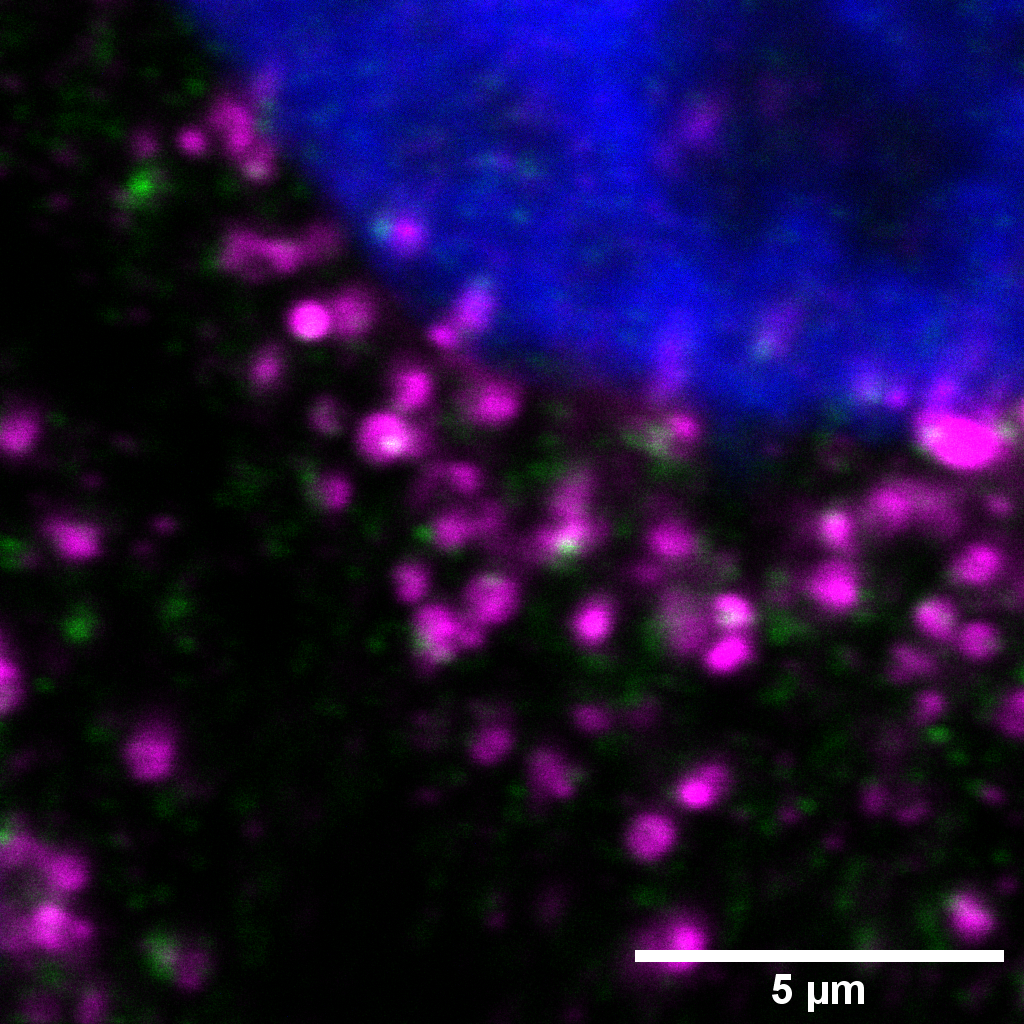

Supplement: Supplementary file 4 — Source data Fig. 2 [file 44318_2025_672_MOESM4_ESM.zip › Figure 2/2A/7KO_LLOMe_scale_zoom.tif]

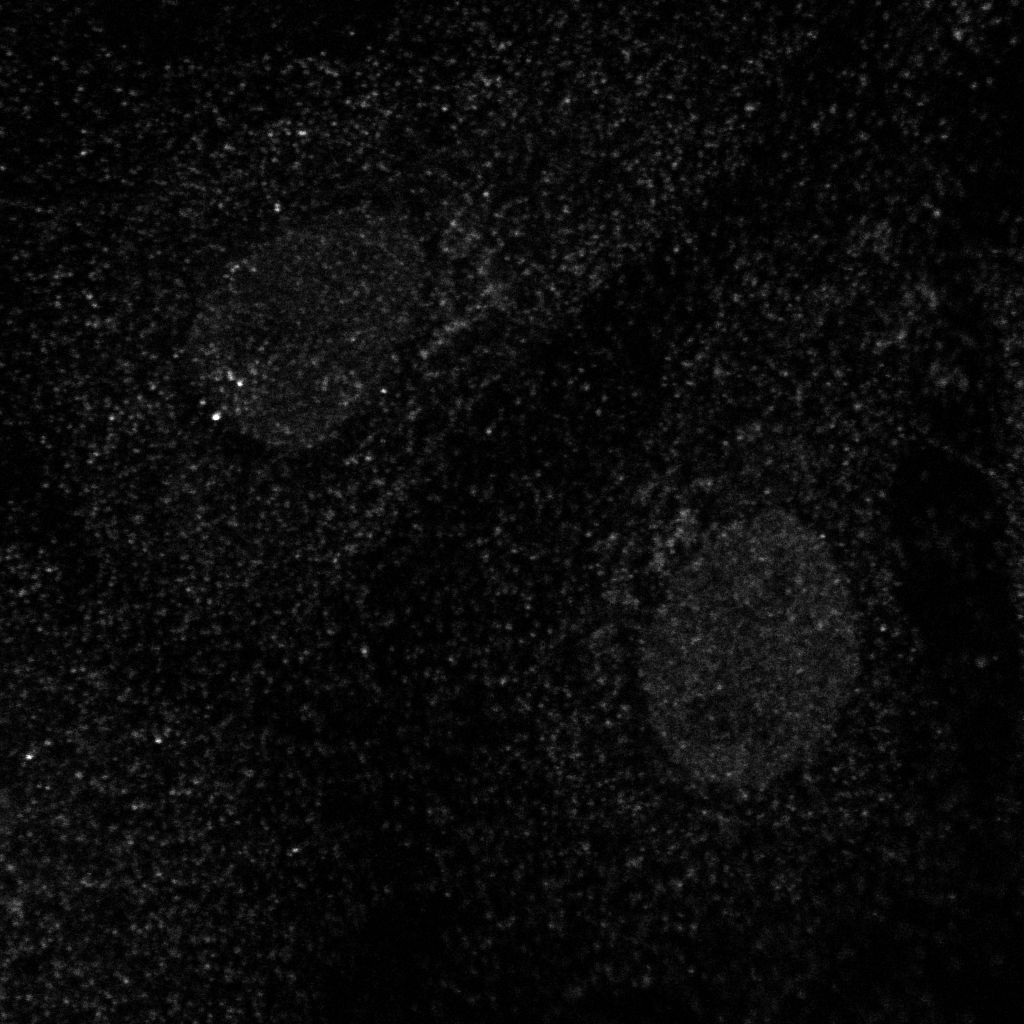

Supplement: Supplementary file 4 — Source data Fig. 2 [file 44318_2025_672_MOESM4_ESM.zip › Figure 2/2A/7KO_VEH_ALIX.tif]

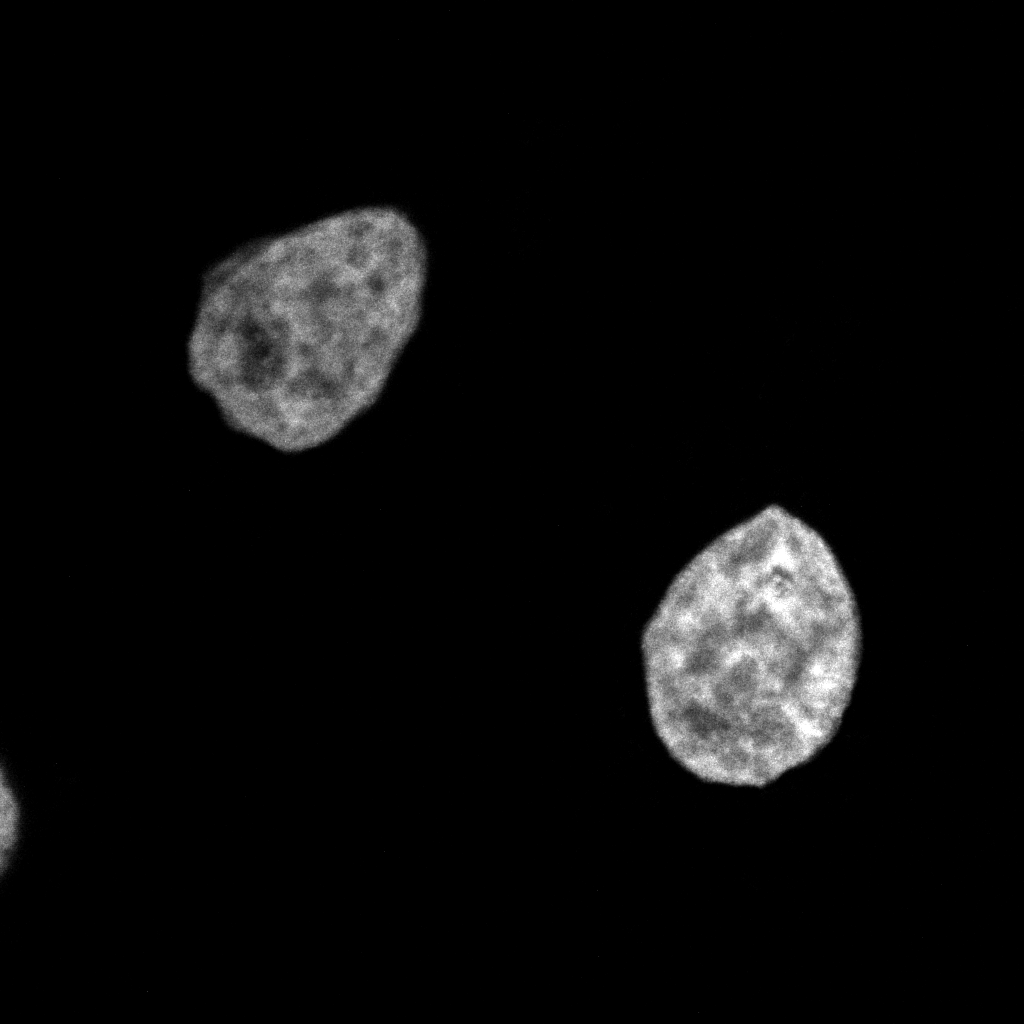

Supplement: Supplementary file 4 — Source data Fig. 2 [file 44318_2025_672_MOESM4_ESM.zip › Figure 2/2A/7KO_VEH_DAPI.tif]

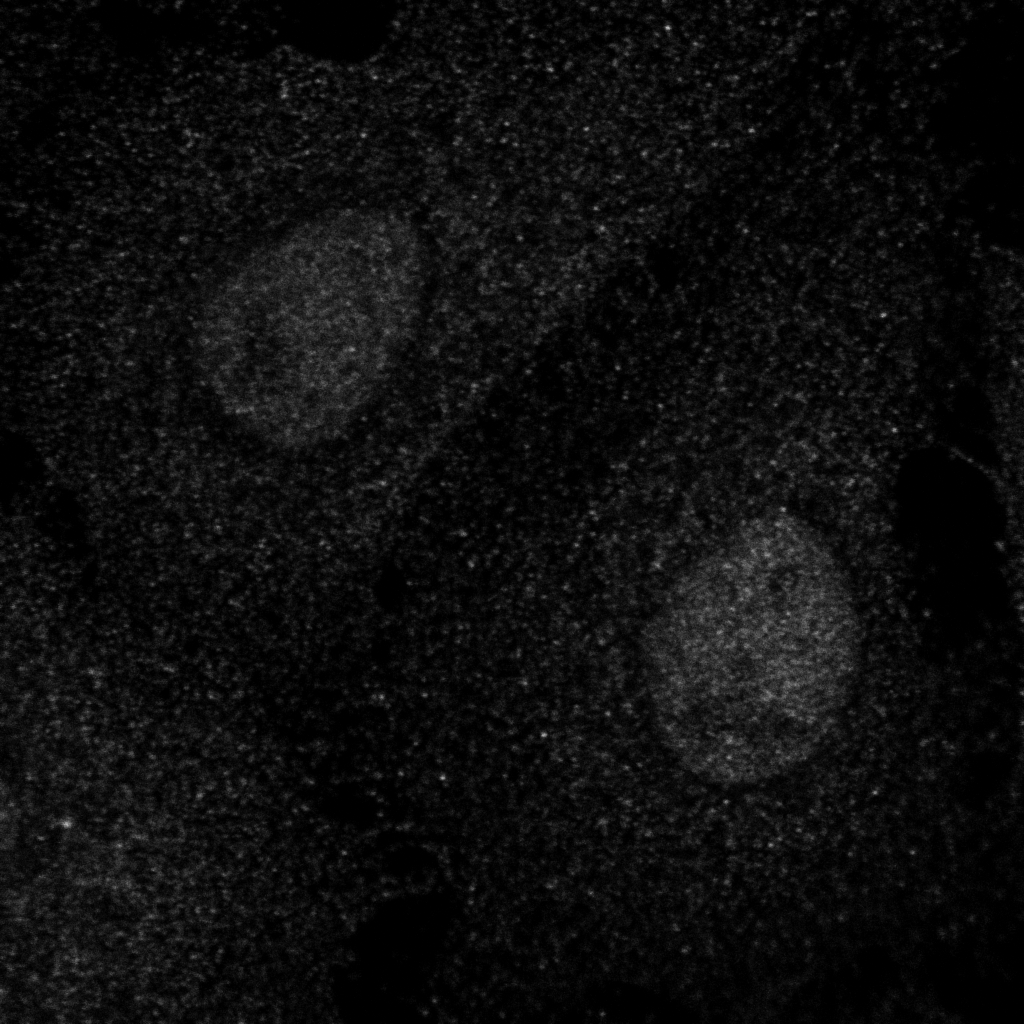

Supplement: Supplementary file 4 — Source data Fig. 2 [file 44318_2025_672_MOESM4_ESM.zip › Figure 2/2A/7KO_VEH_Gal3.tif]

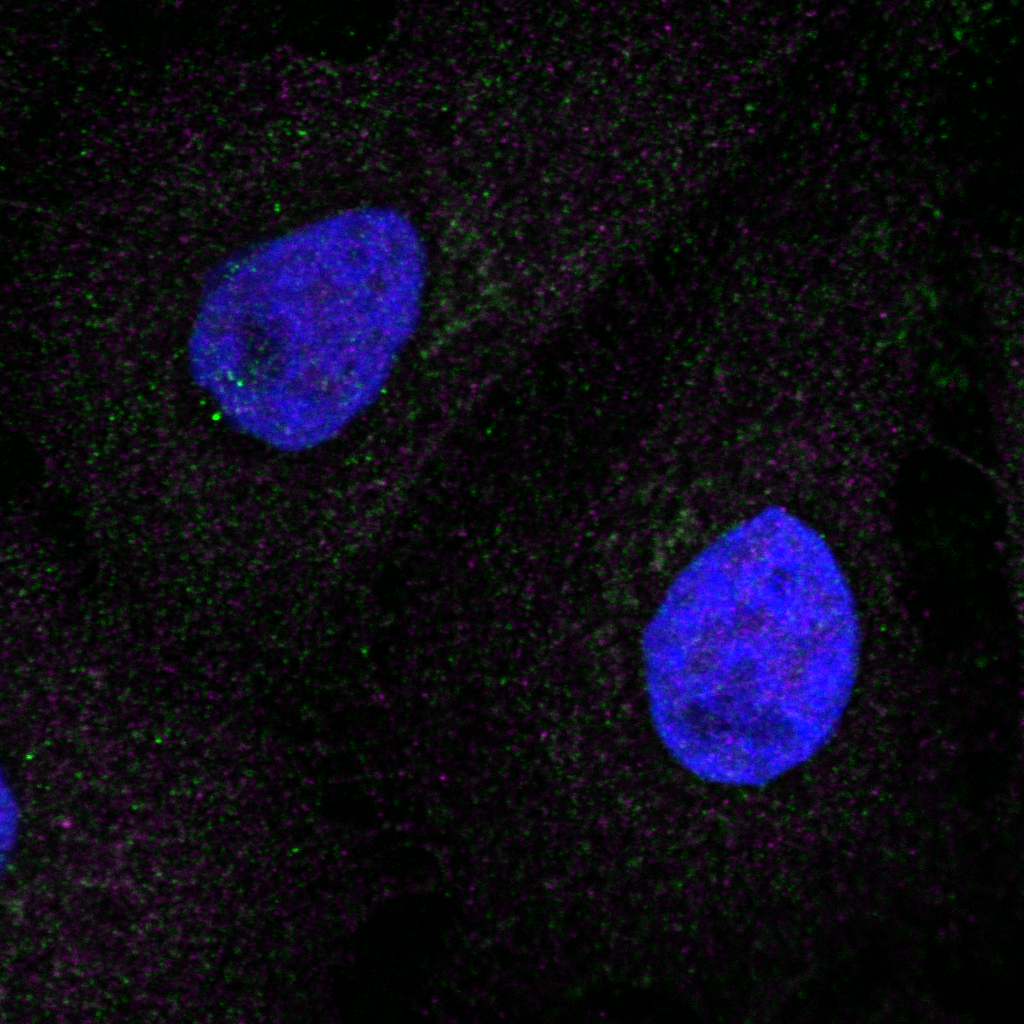

Supplement: Supplementary file 4 — Source data Fig. 2 [file 44318_2025_672_MOESM4_ESM.zip › Figure 2/2A/7KO_VEH_merge.tif]

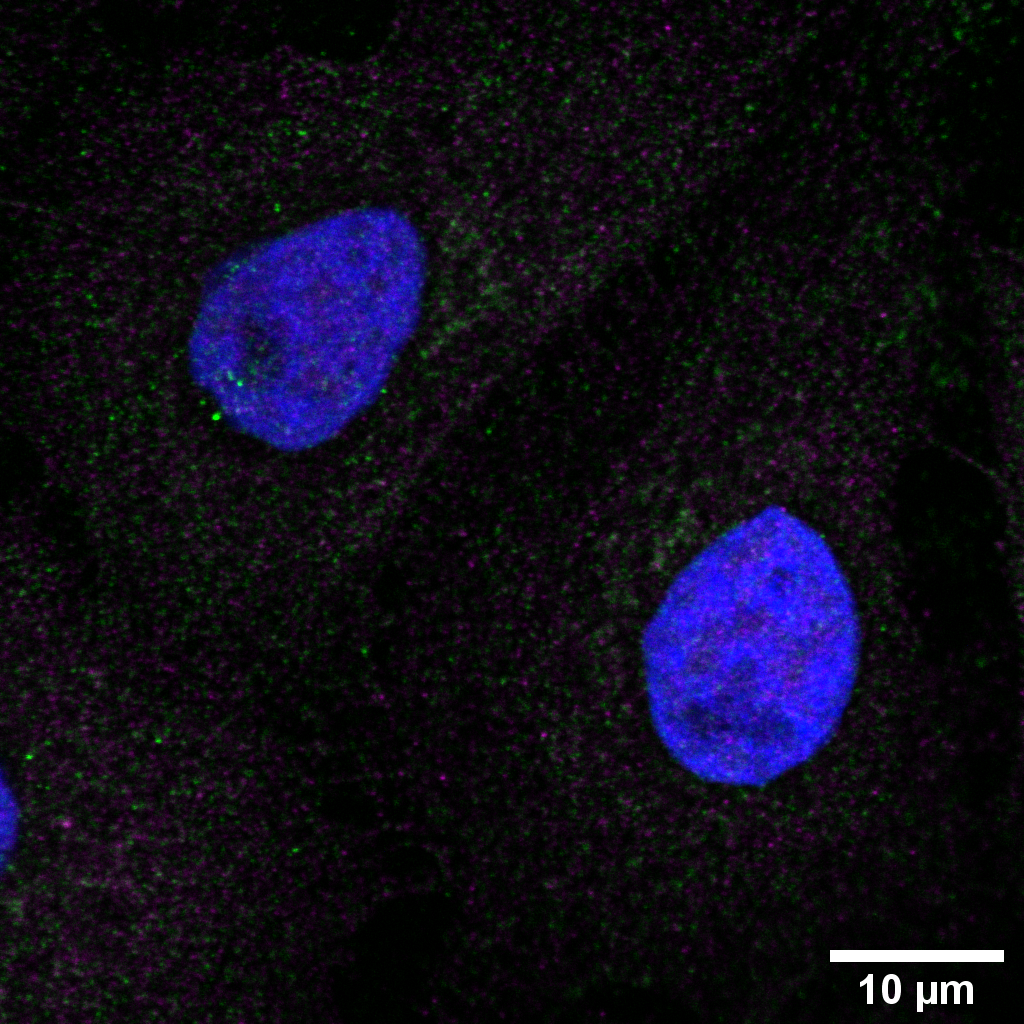

Supplement: Supplementary file 4 — Source data Fig. 2 [file 44318_2025_672_MOESM4_ESM.zip › Figure 2/2A/7KO_VEH_scale.tif]

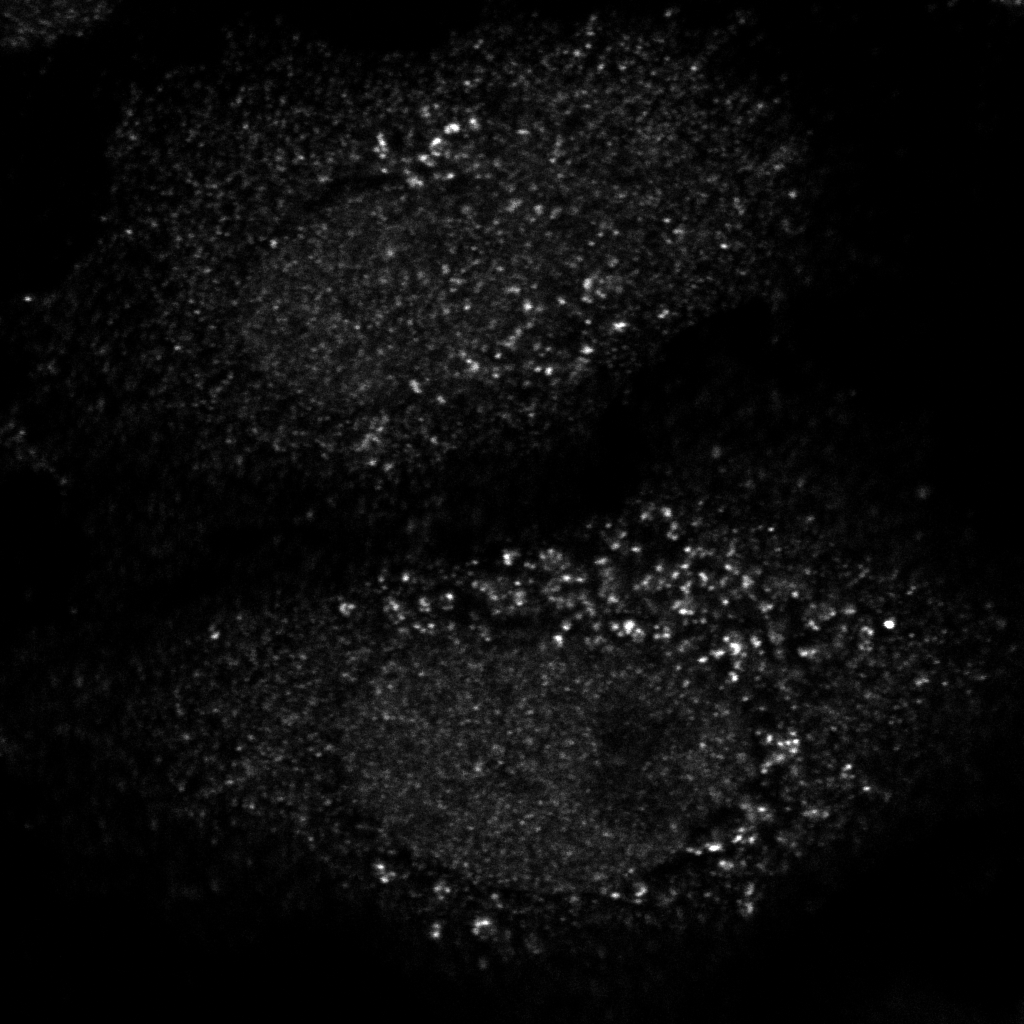

Supplement: Supplementary file 4 — Source data Fig. 2 [file 44318_2025_672_MOESM4_ESM.zip › Figure 2/2A/8KO_LLOMe_ALIX.tif]

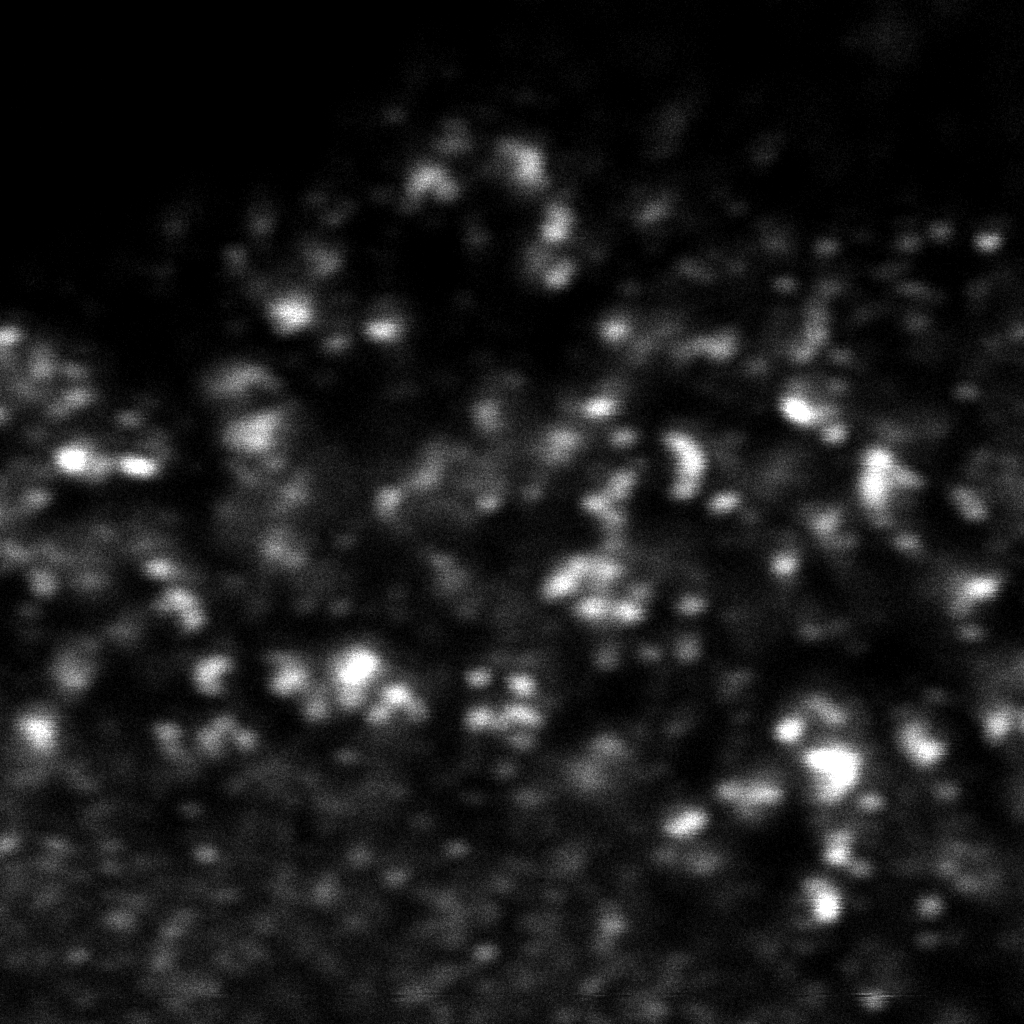

Supplement: Supplementary file 4 — Source data Fig. 2 [file 44318_2025_672_MOESM4_ESM.zip › Figure 2/2A/8KO_LLOME_ALIX_zoom.tif]

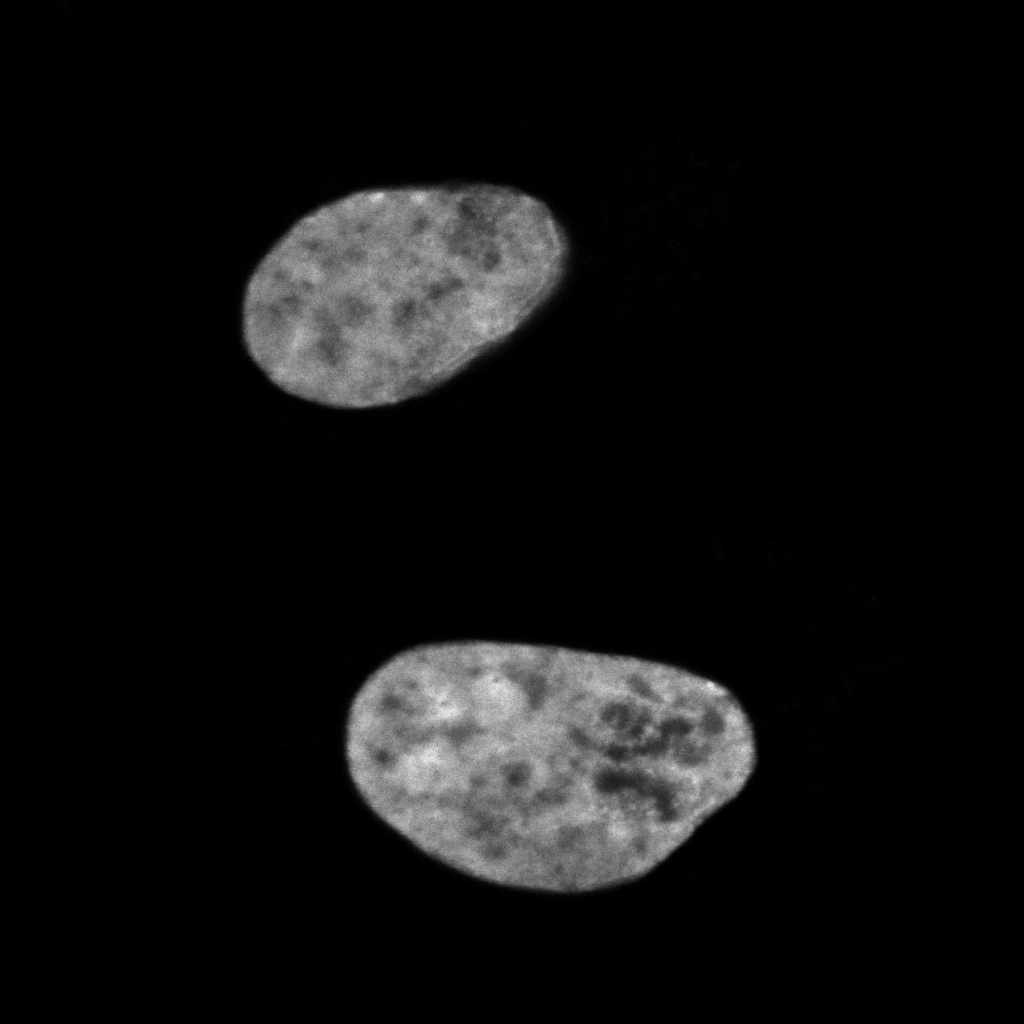

Supplement: Supplementary file 4 — Source data Fig. 2 [file 44318_2025_672_MOESM4_ESM.zip › Figure 2/2A/8KO_LLOMe_DAPI.tif]

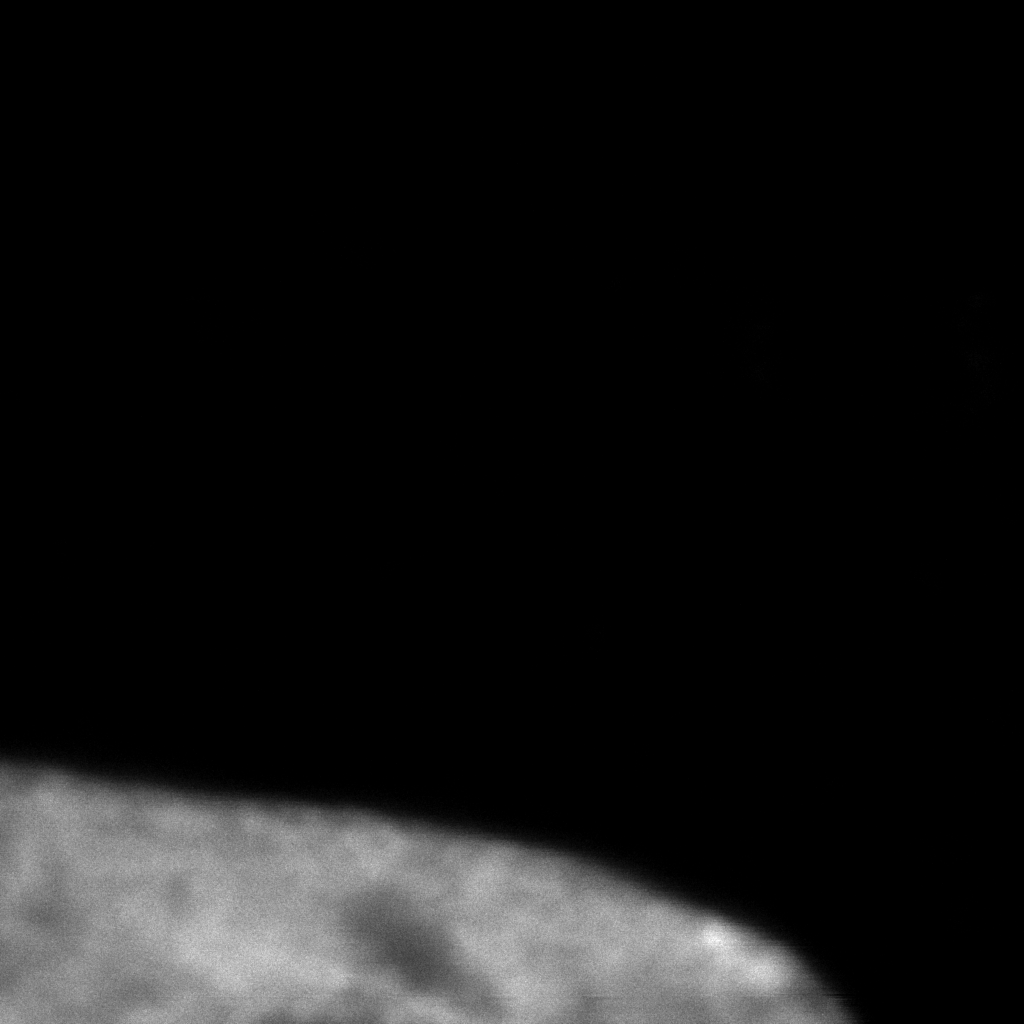

Supplement: Supplementary file 4 — Source data Fig. 2 [file 44318_2025_672_MOESM4_ESM.zip › Figure 2/2A/8KO_LLOMe_DAPI_zoom.tif]

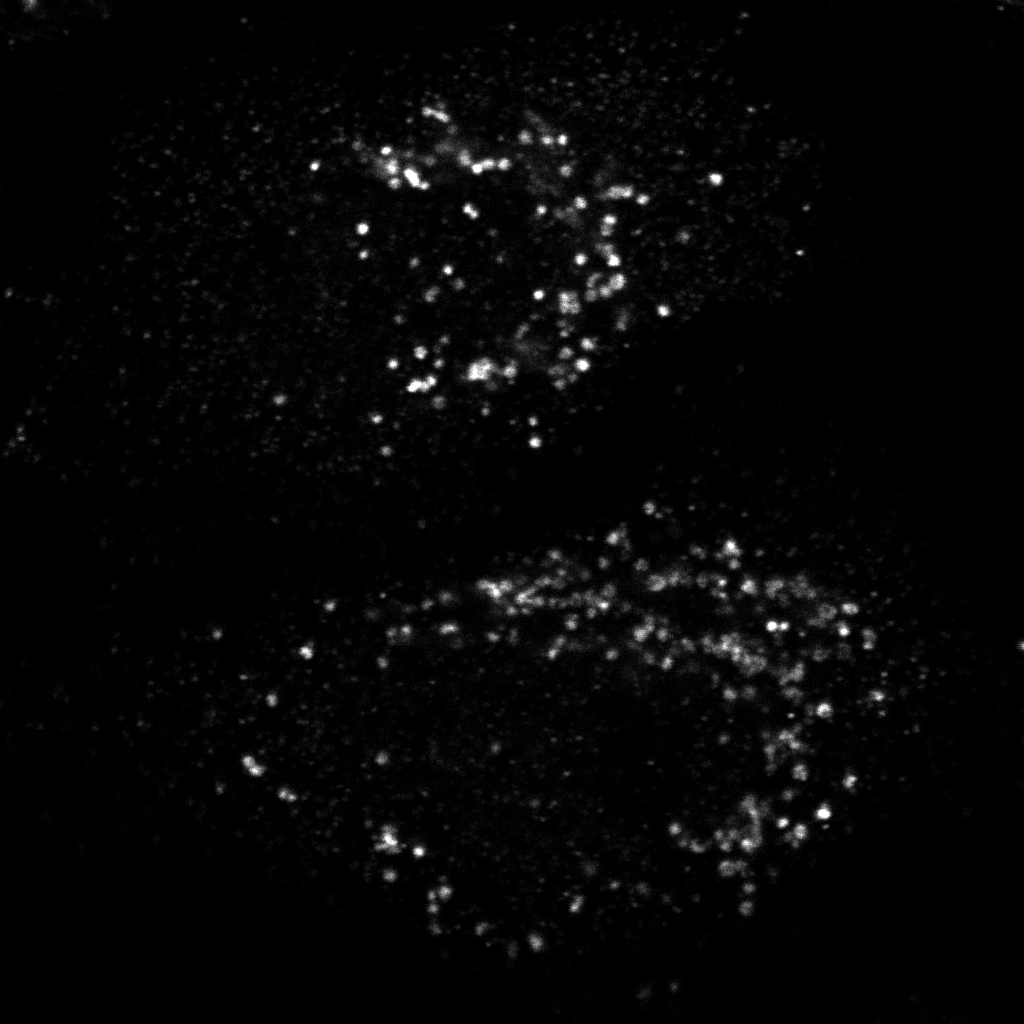

Supplement: Supplementary file 4 — Source data Fig. 2 [file 44318_2025_672_MOESM4_ESM.zip › Figure 2/2A/8KO_LLOMe_Gal3.tif]

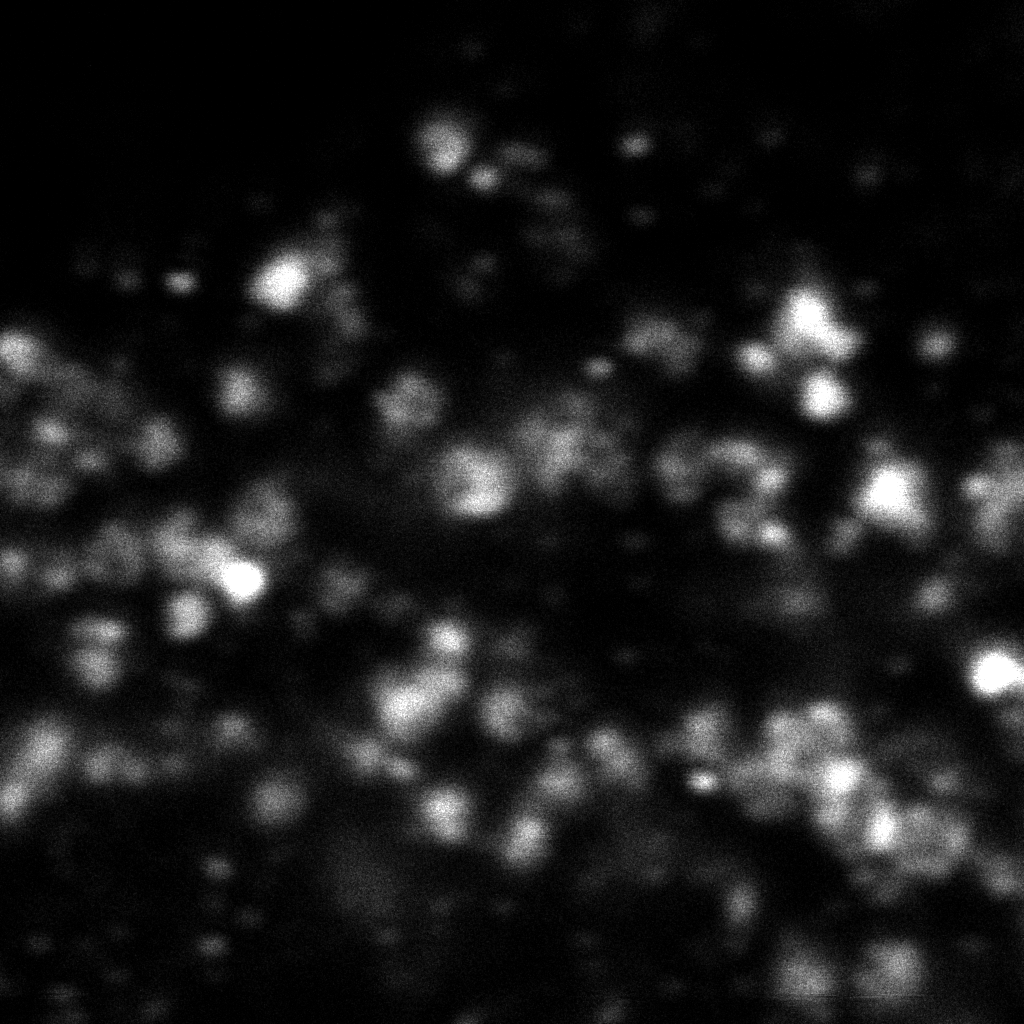

Supplement: Supplementary file 4 — Source data Fig. 2 [file 44318_2025_672_MOESM4_ESM.zip › Figure 2/2A/8KO_LLOMe_Gal3_zoom.tif]

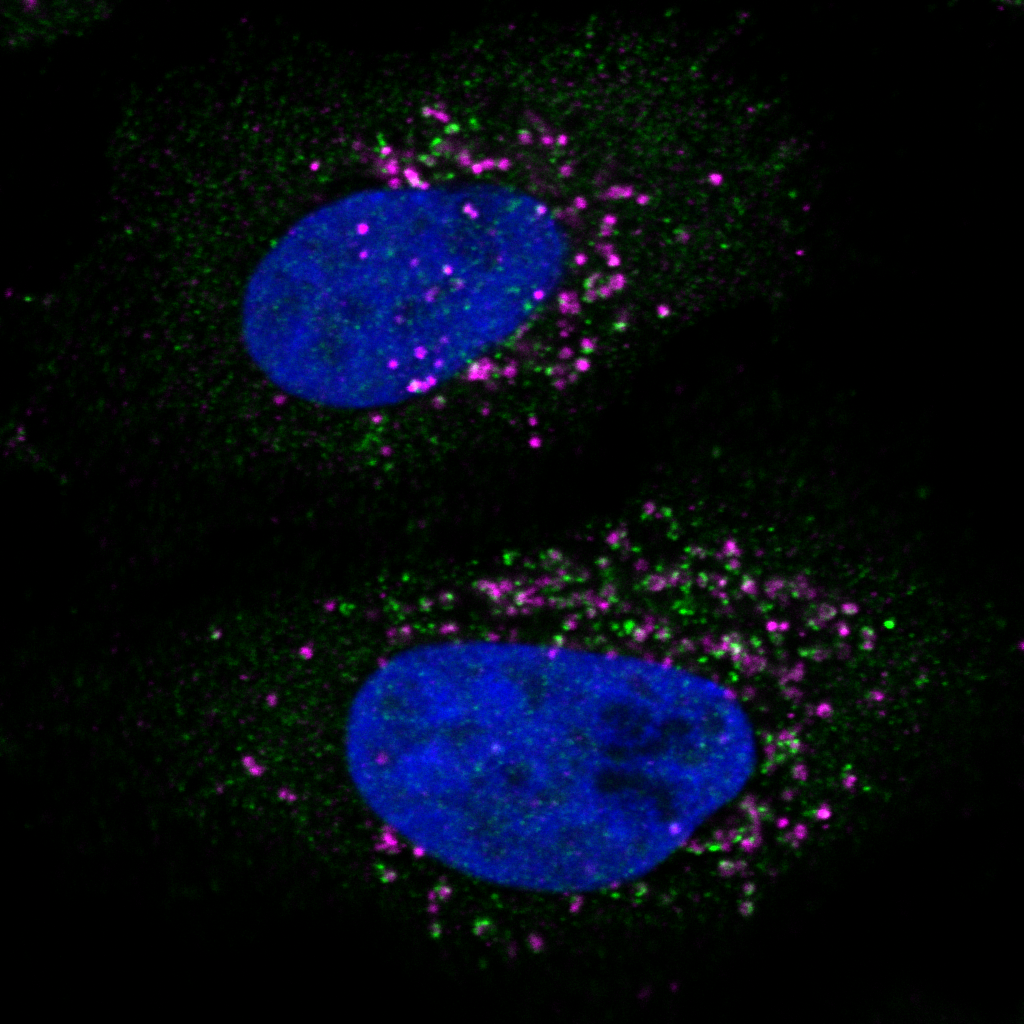

Supplement: Supplementary file 4 — Source data Fig. 2 [file 44318_2025_672_MOESM4_ESM.zip › Figure 2/2A/8KO_LLOMe_merge.tif]

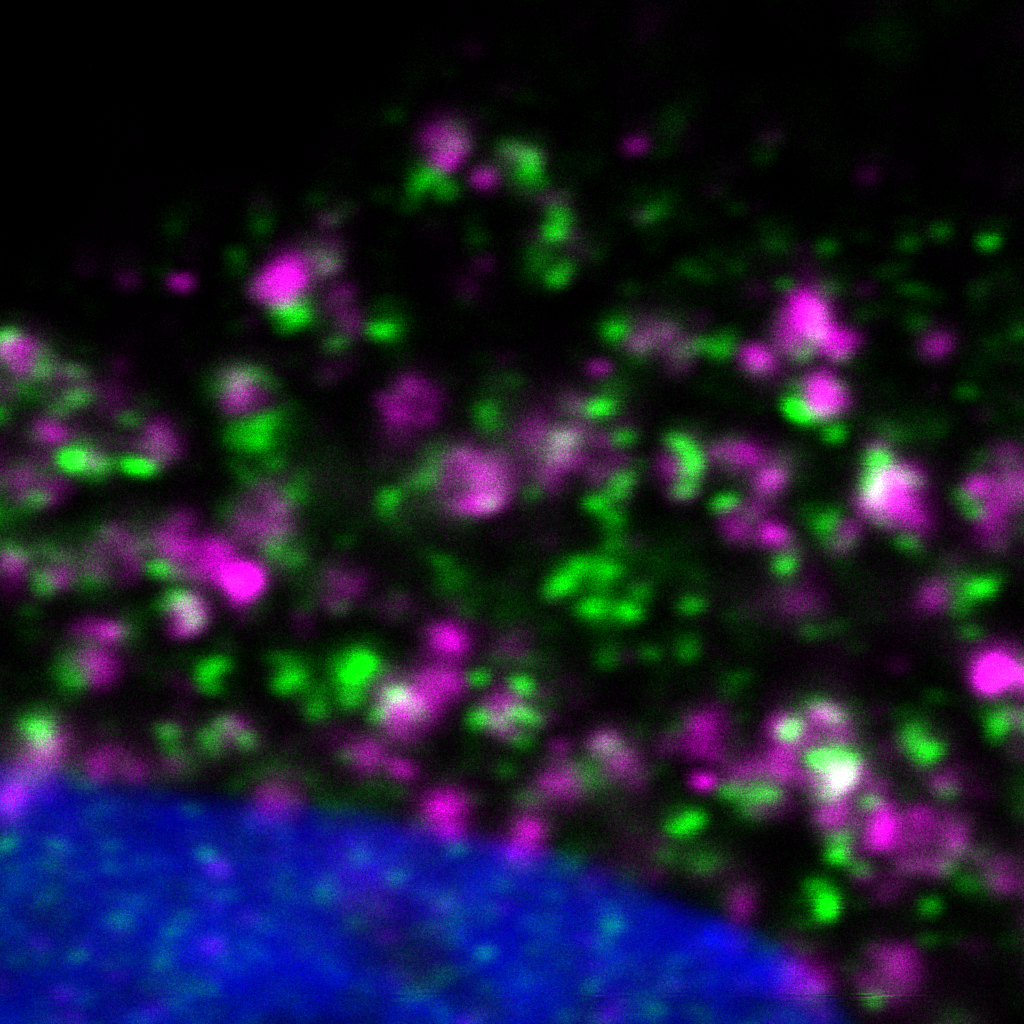

Supplement: Supplementary file 4 — Source data Fig. 2 [file 44318_2025_672_MOESM4_ESM.zip › Figure 2/2A/8KO_LLOMe_merge_zoom.tif]

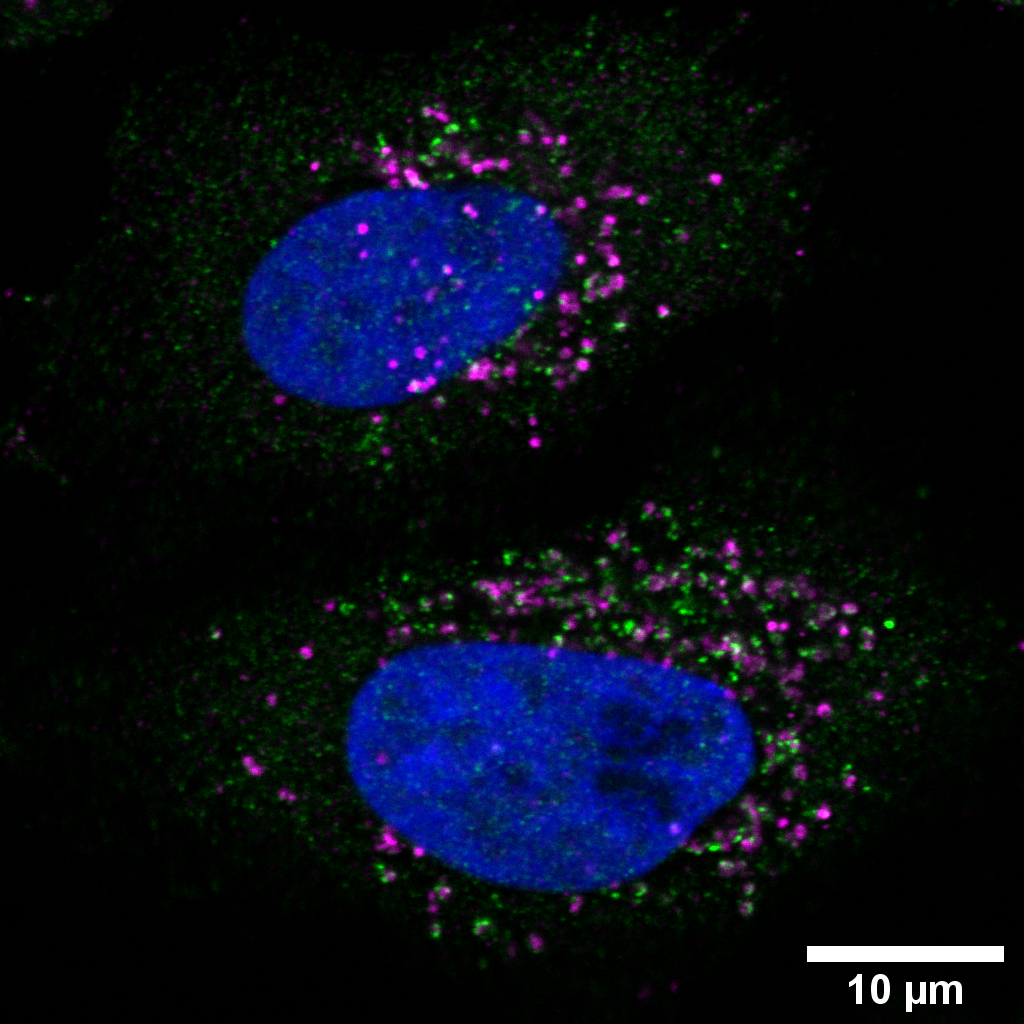

Supplement: Supplementary file 4 — Source data Fig. 2 [file 44318_2025_672_MOESM4_ESM.zip › Figure 2/2A/8KO_LLOMe_scale.tif]

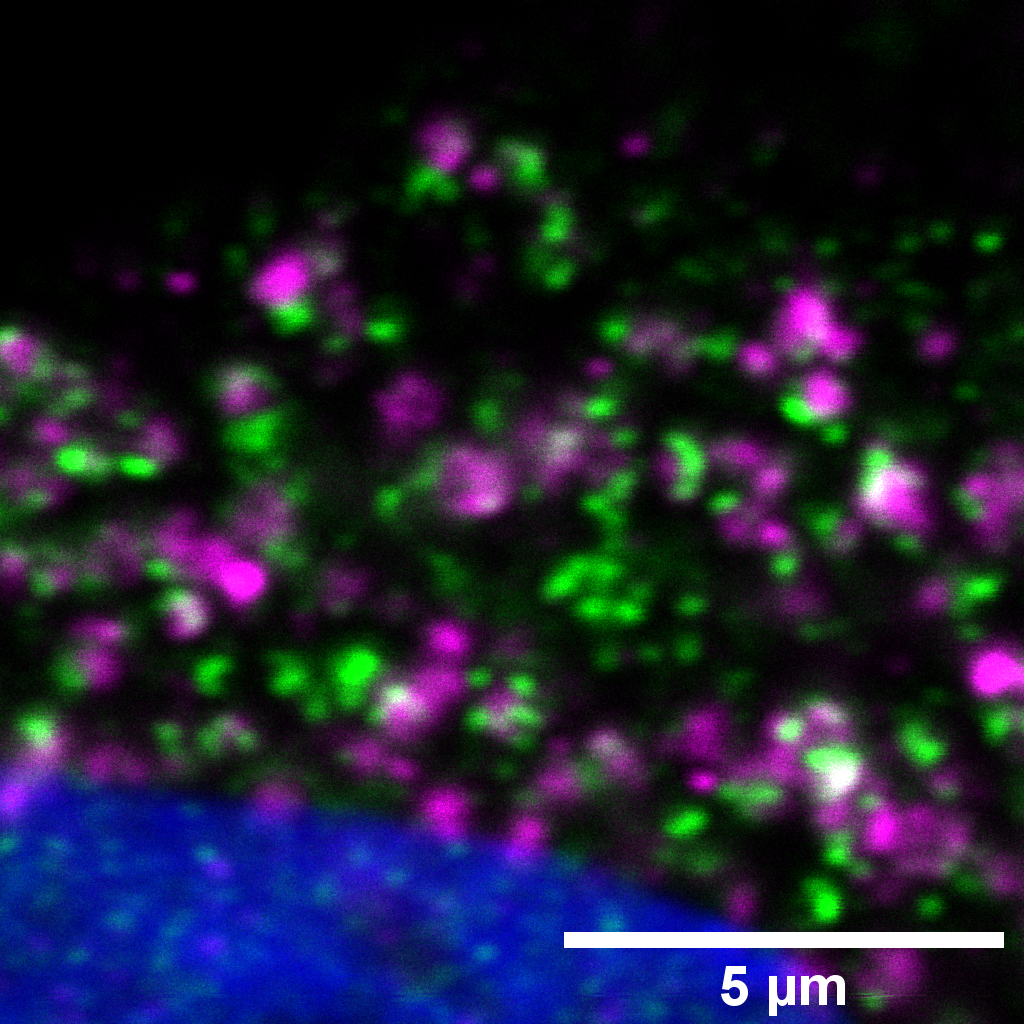

Supplement: Supplementary file 4 — Source data Fig. 2 [file 44318_2025_672_MOESM4_ESM.zip › Figure 2/2A/8KO_LLOMe_scale_zoom.tif]

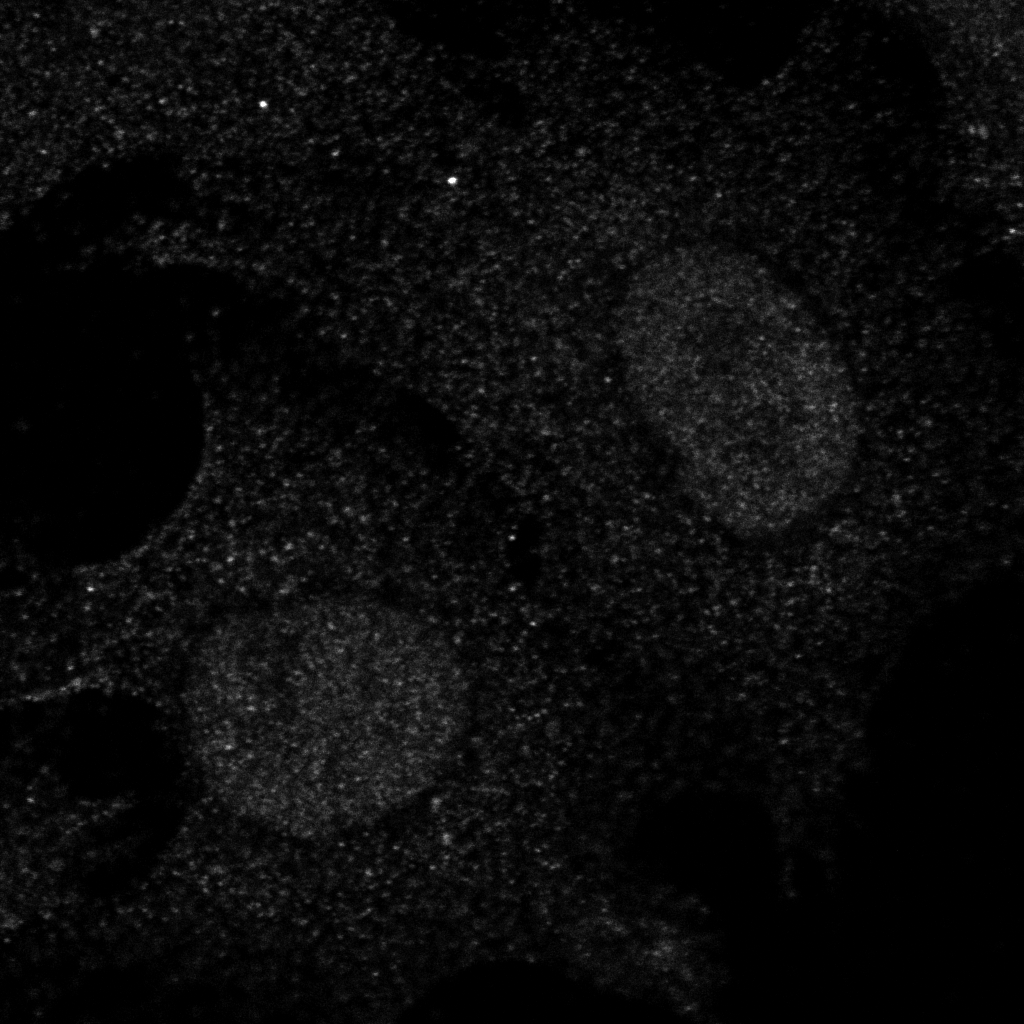

Supplement: Supplementary file 4 — Source data Fig. 2 [file 44318_2025_672_MOESM4_ESM.zip › Figure 2/2A/8KO_VEH_ALIX.tif]

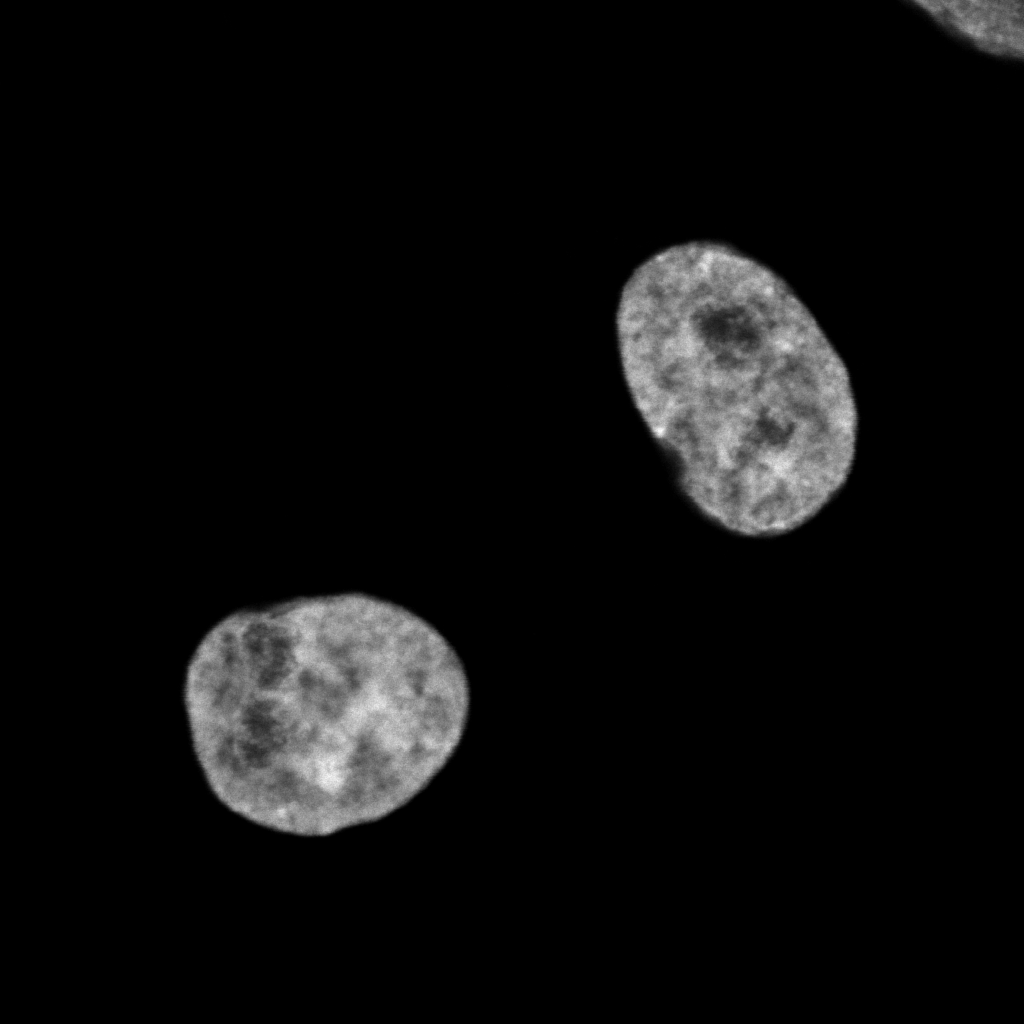

Supplement: Supplementary file 4 — Source data Fig. 2 [file 44318_2025_672_MOESM4_ESM.zip › Figure 2/2A/8KO_VEH_DAPI.tif]

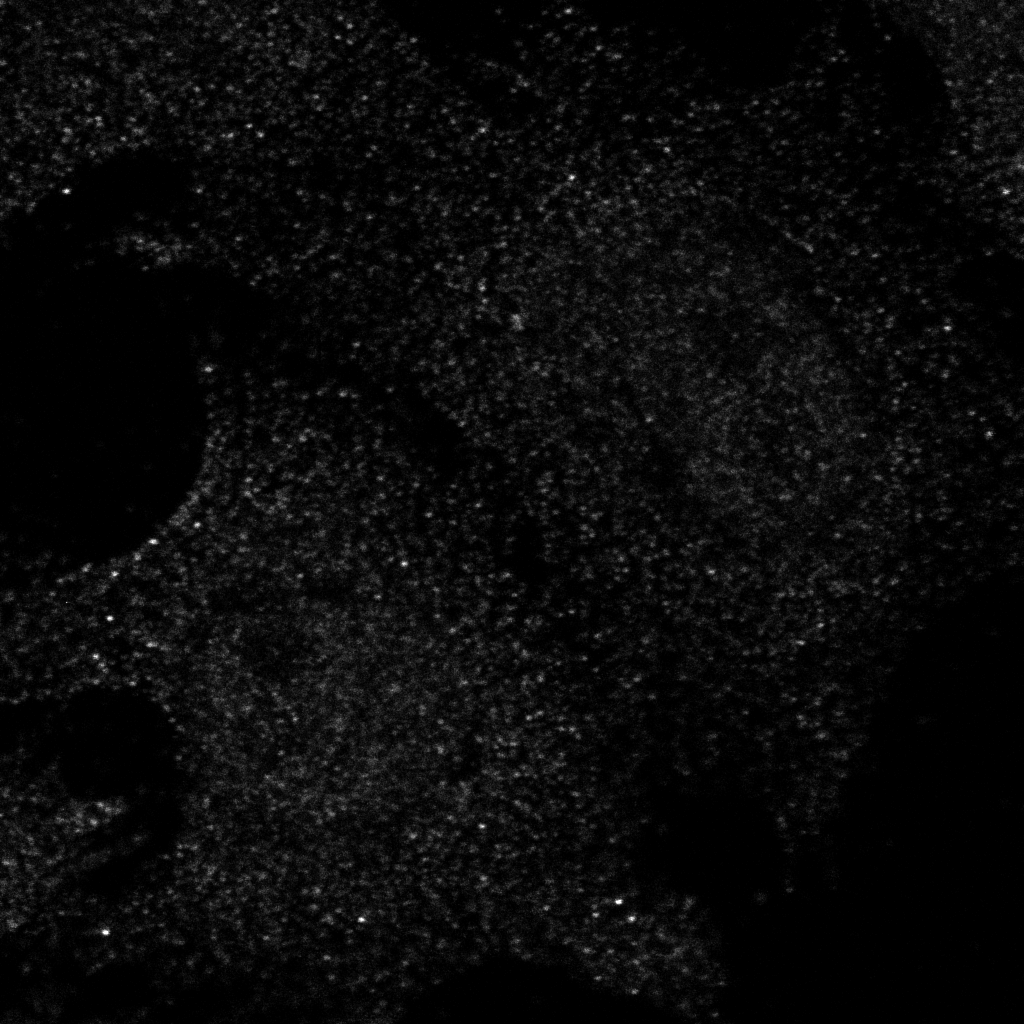

Supplement: Supplementary file 4 — Source data Fig. 2 [file 44318_2025_672_MOESM4_ESM.zip › Figure 2/2A/8KO_VEH_Gal3.tif]

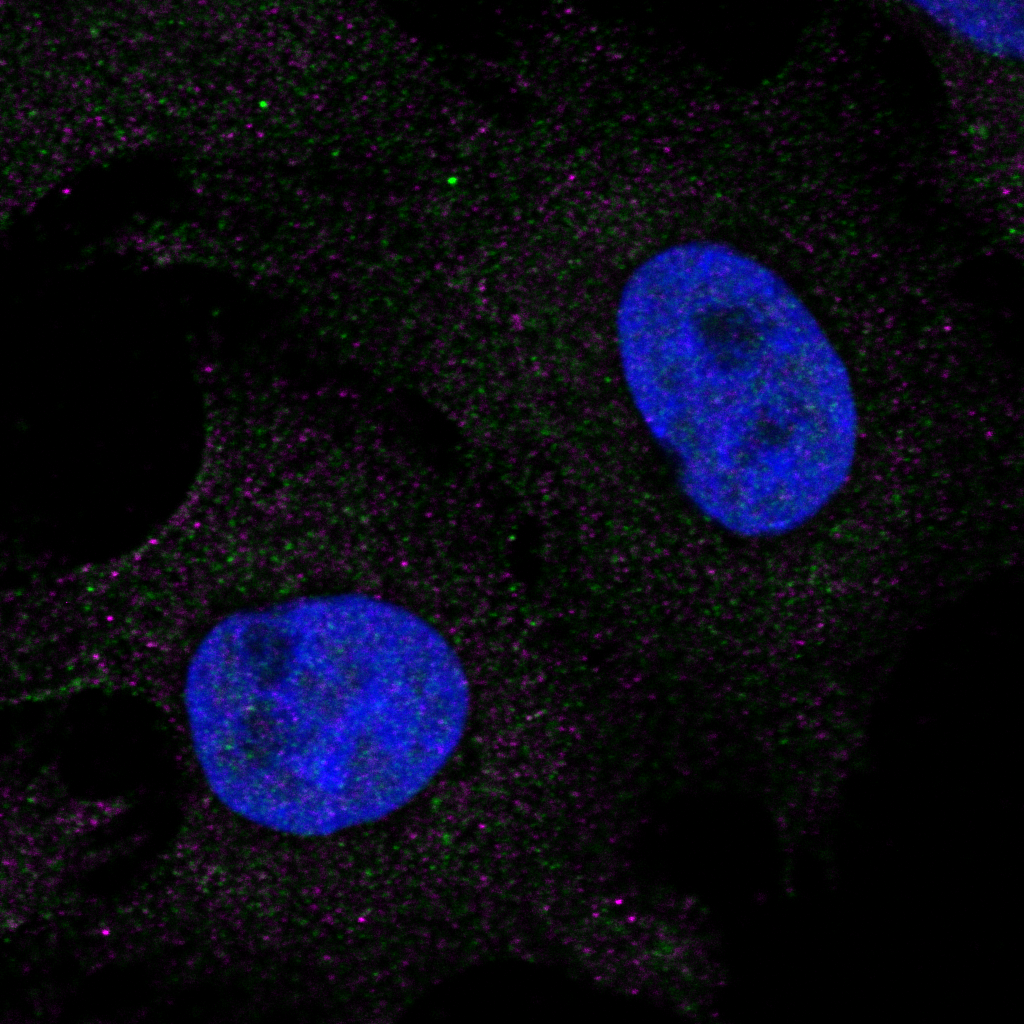

Supplement: Supplementary file 4 — Source data Fig. 2 [file 44318_2025_672_MOESM4_ESM.zip › Figure 2/2A/8KO_VEH_merge.tif]

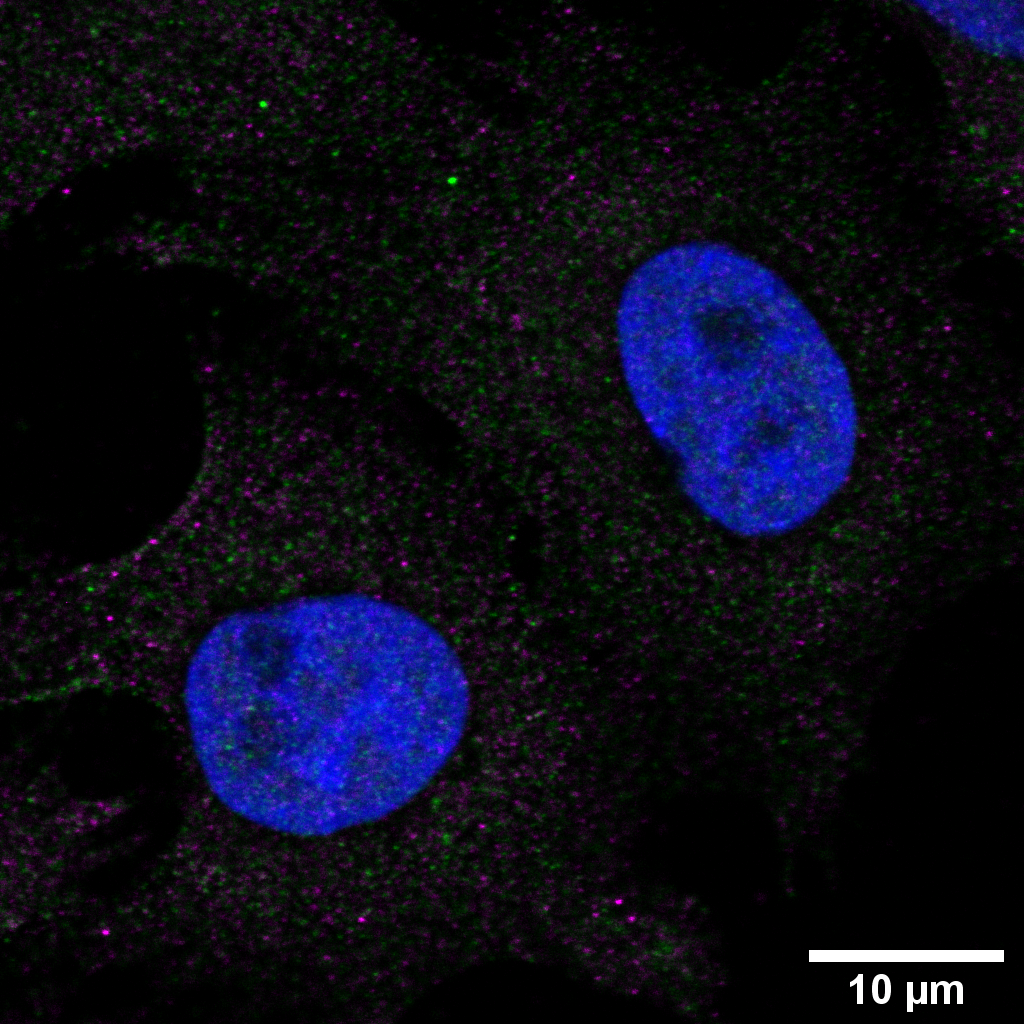

Supplement: Supplementary file 4 — Source data Fig. 2 [file 44318_2025_672_MOESM4_ESM.zip › Figure 2/2A/8KO_VEH_scale.tif]

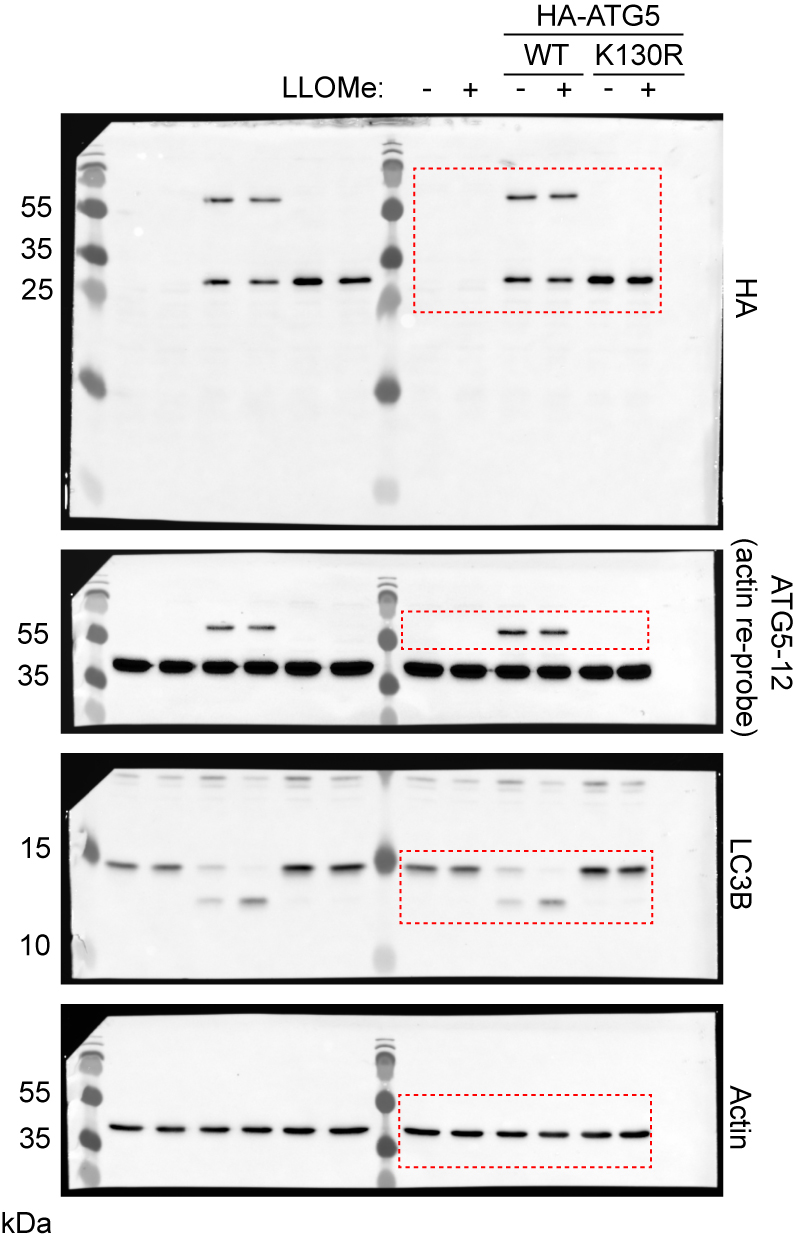

Supplement: Supplementary file 4 — Source data Fig. 2 [file 44318_2025_672_MOESM4_ESM.zip › Figure 2/2C/HA-ATG5_uncropped blots.jpg]

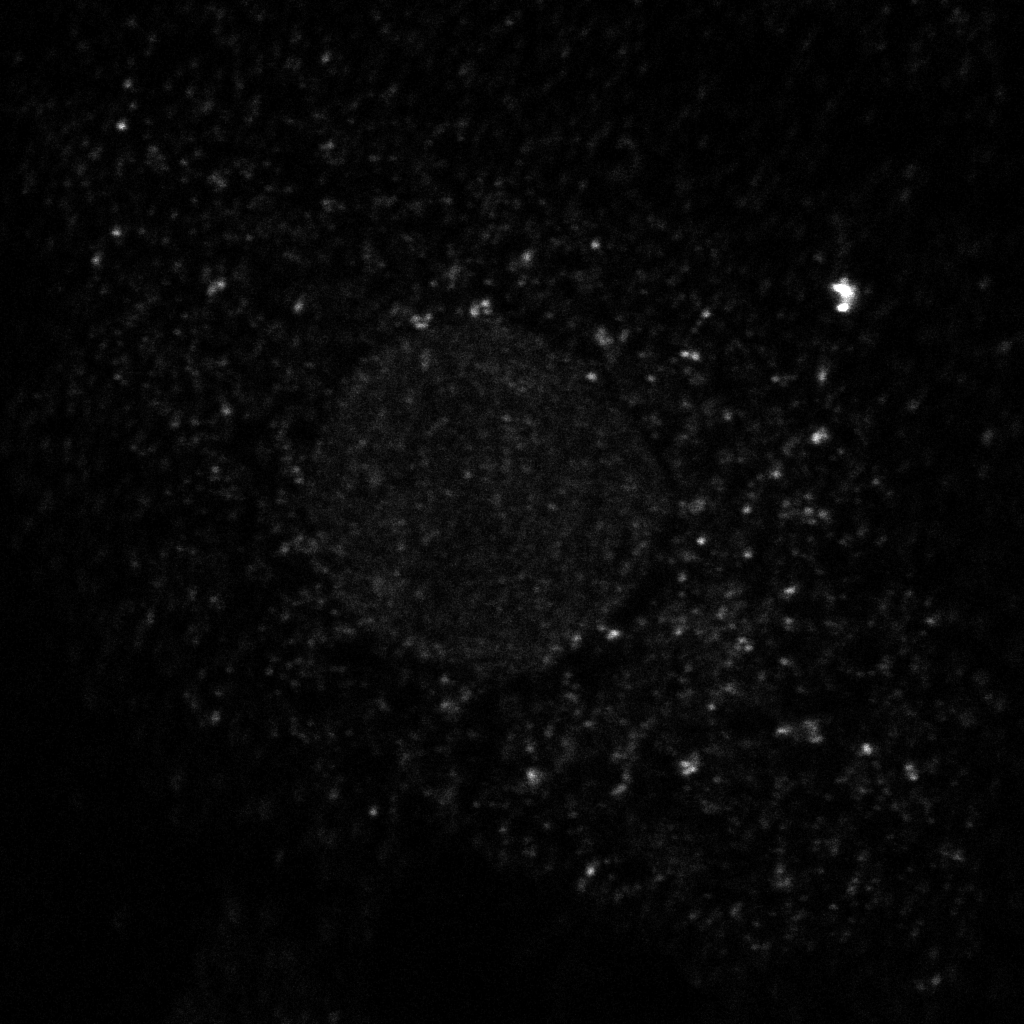

Supplement: Supplementary file 4 — Source data Fig. 2 [file 44318_2025_672_MOESM4_ESM.zip › Figure 2/2D/5KO_ALIX.tif]

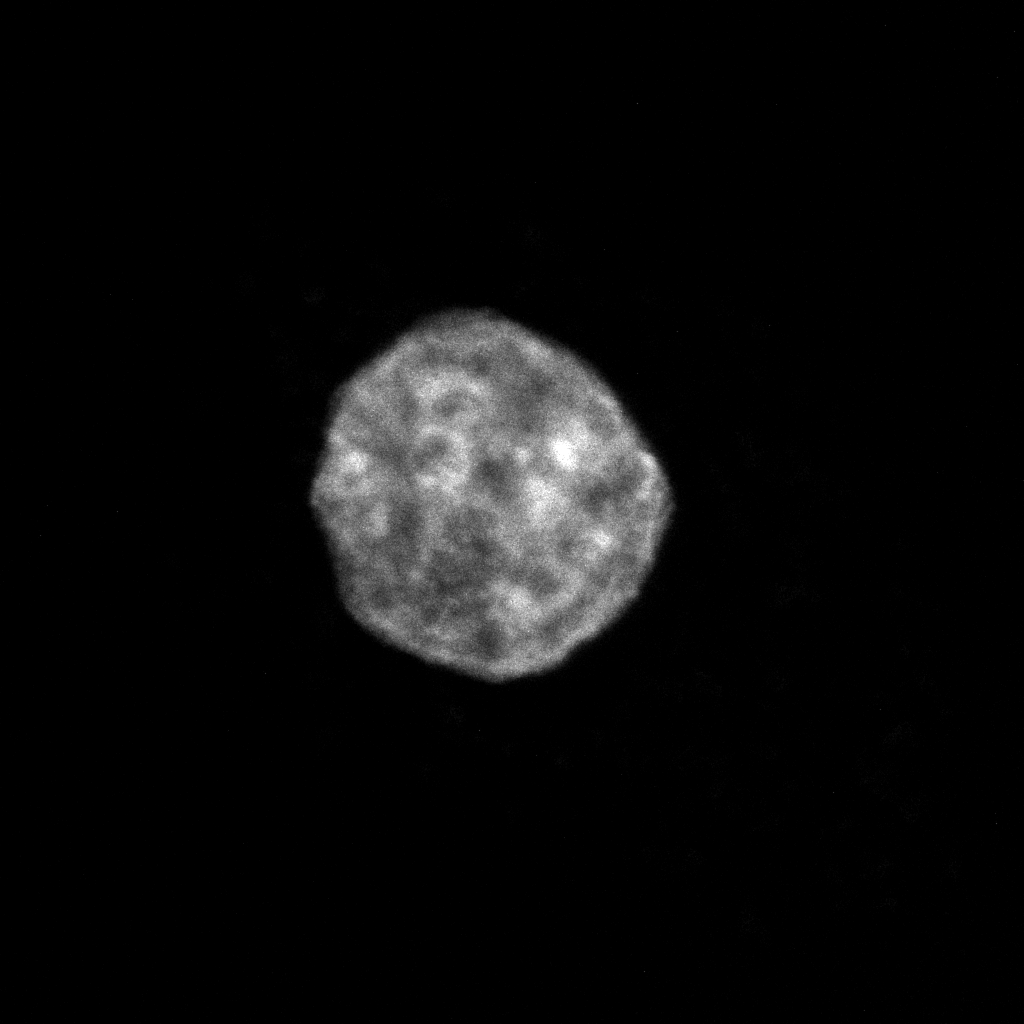

Supplement: Supplementary file 4 — Source data Fig. 2 [file 44318_2025_672_MOESM4_ESM.zip › Figure 2/2D/5KO_DAPI.tif]

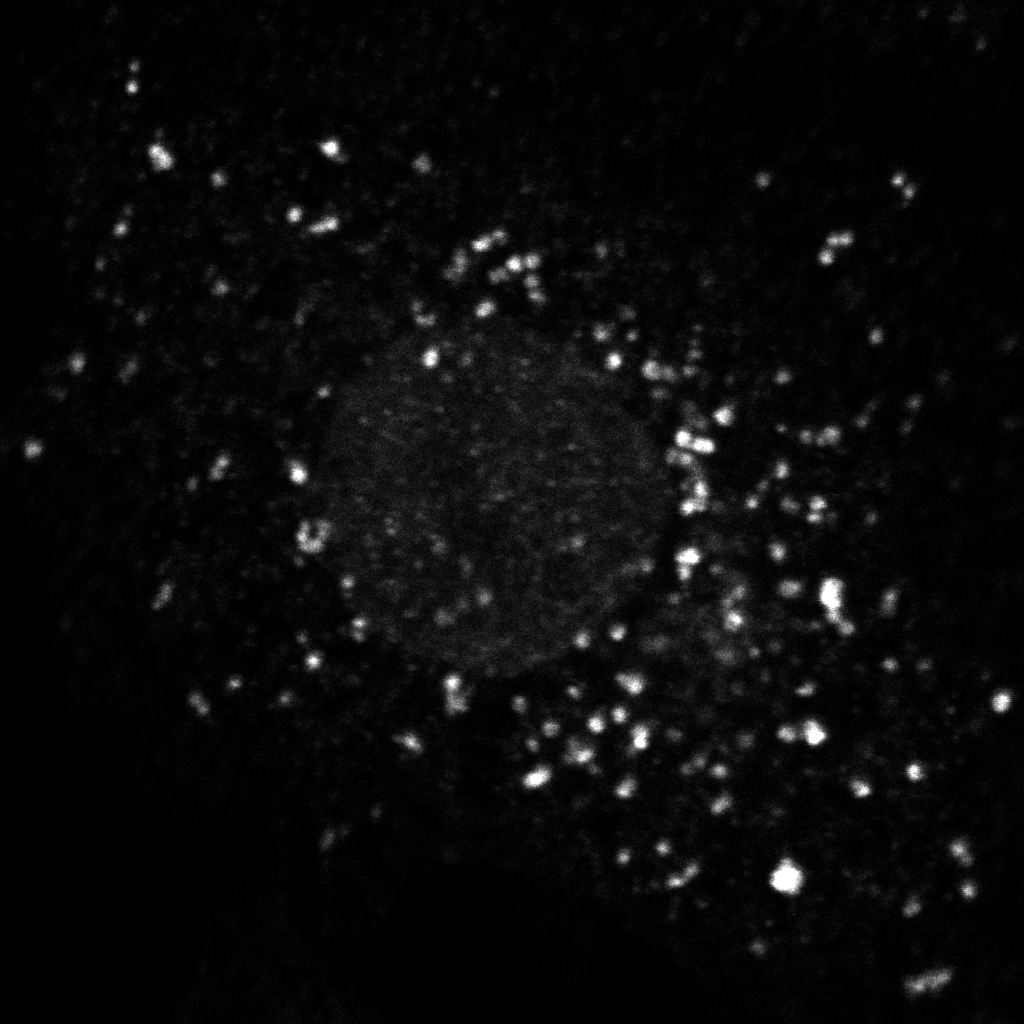

Supplement: Supplementary file 4 — Source data Fig. 2 [file 44318_2025_672_MOESM4_ESM.zip › Figure 2/2D/5KO_Gal3.tif]

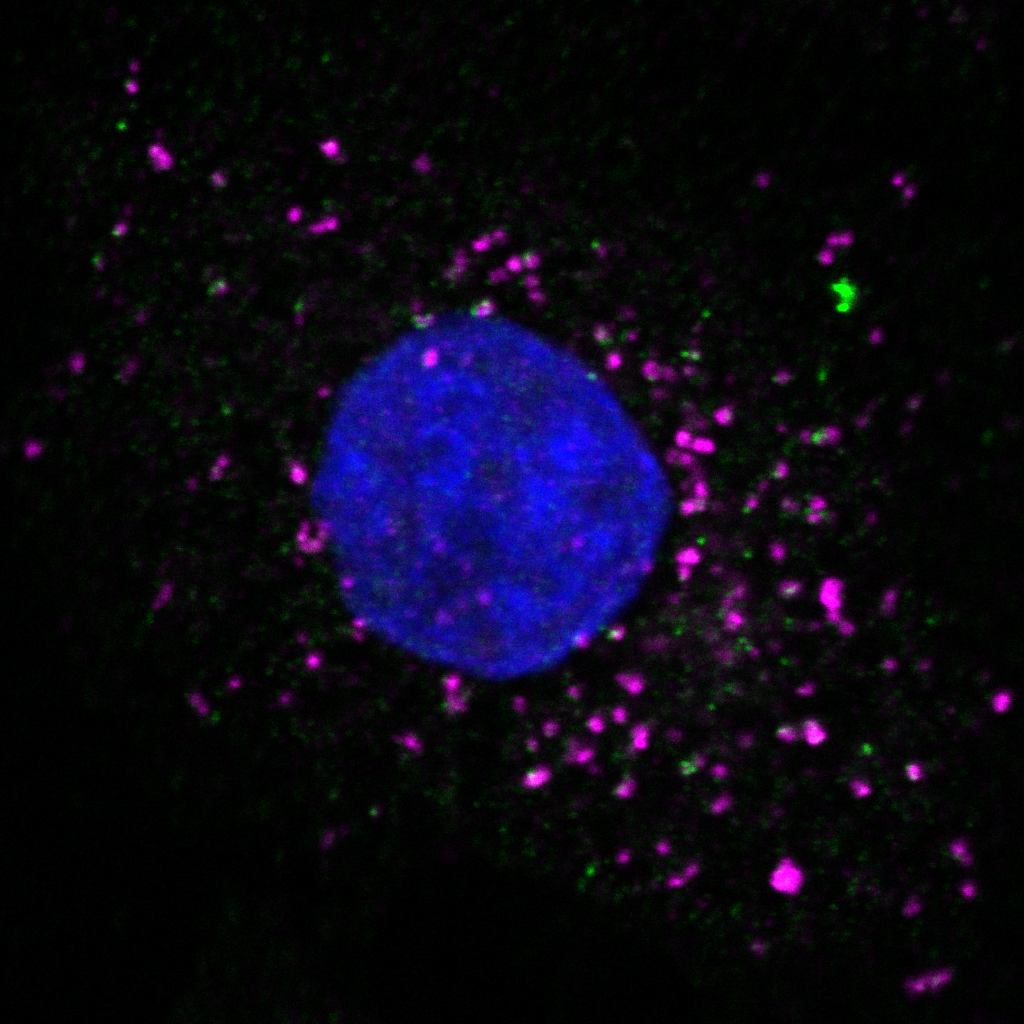

Supplement: Supplementary file 4 — Source data Fig. 2 [file 44318_2025_672_MOESM4_ESM.zip › Figure 2/2D/5KO_merge.tif]

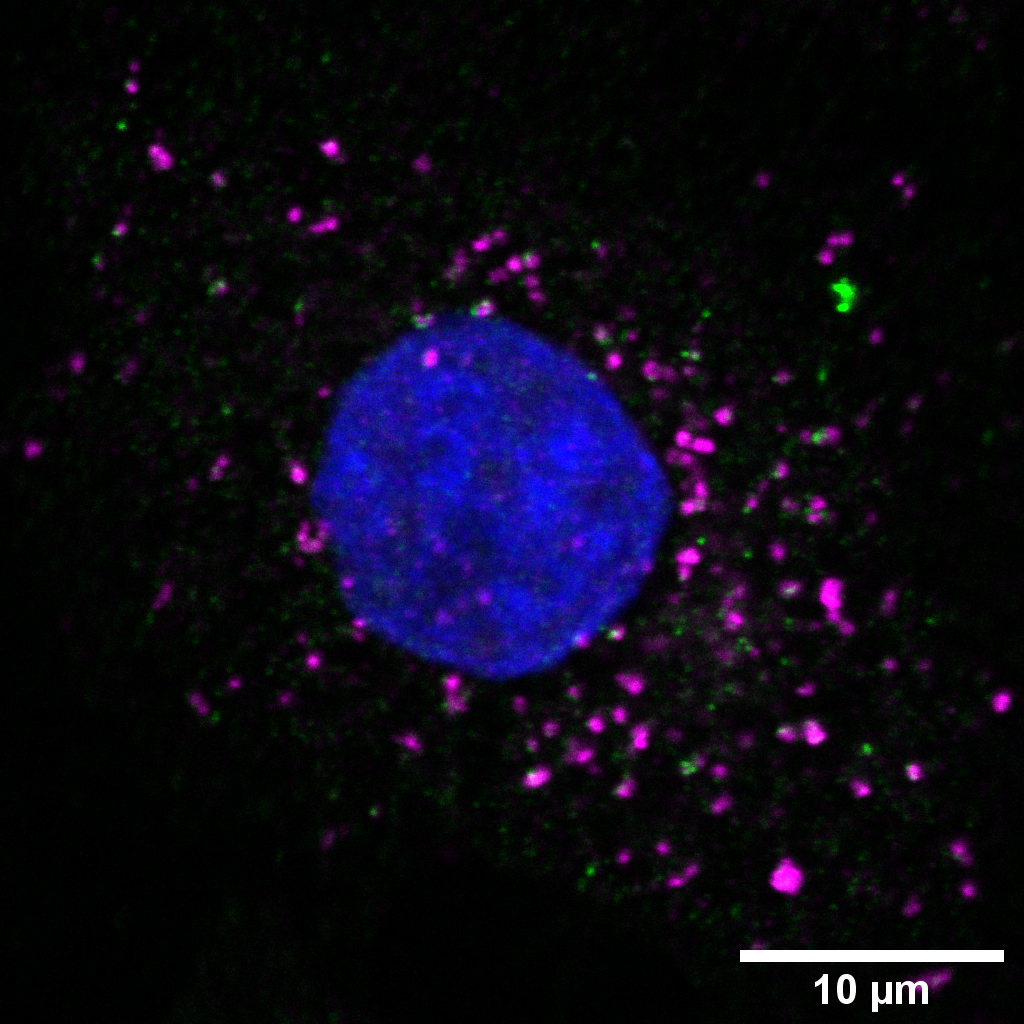

Supplement: Supplementary file 4 — Source data Fig. 2 [file 44318_2025_672_MOESM4_ESM.zip › Figure 2/2D/5KO_scale.tif]

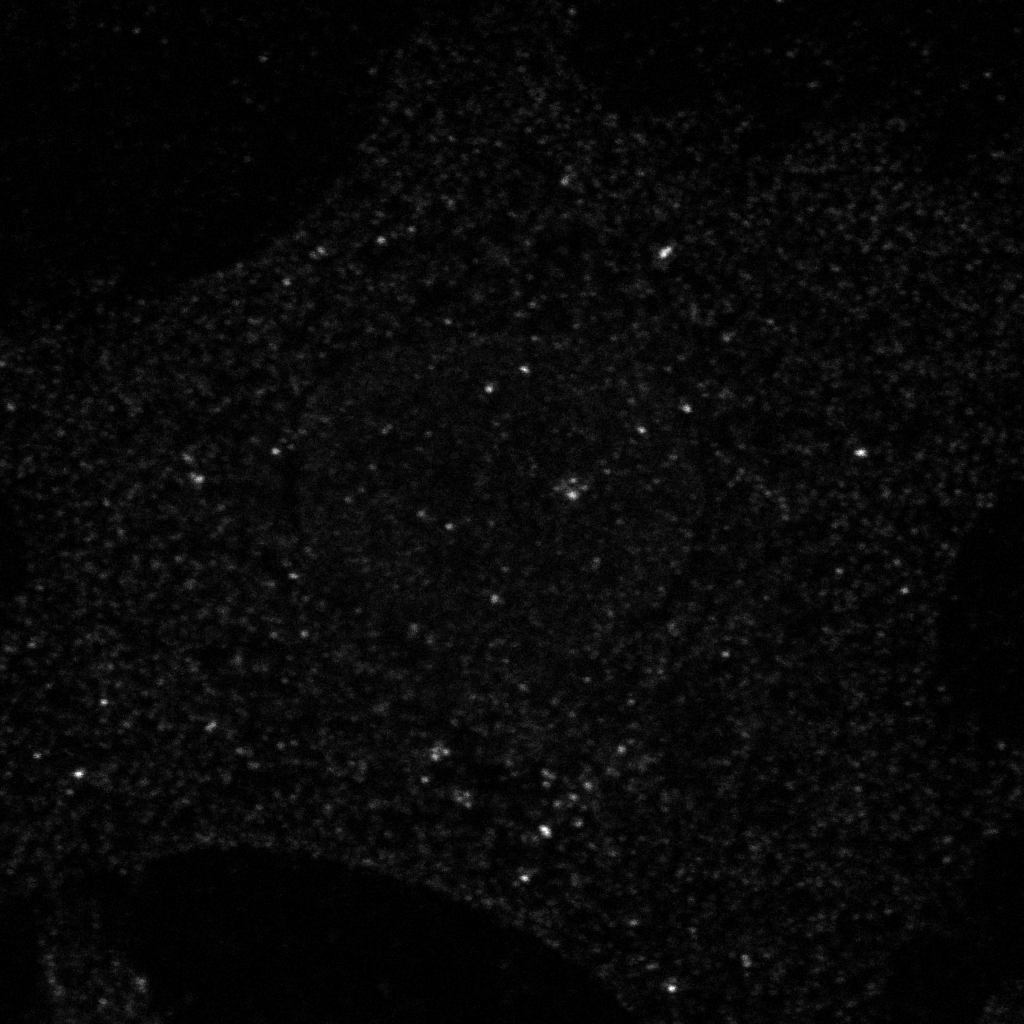

Supplement: Supplementary file 4 — Source data Fig. 2 [file 44318_2025_672_MOESM4_ESM.zip › Figure 2/2D/K130R addback_ALIX.tif]

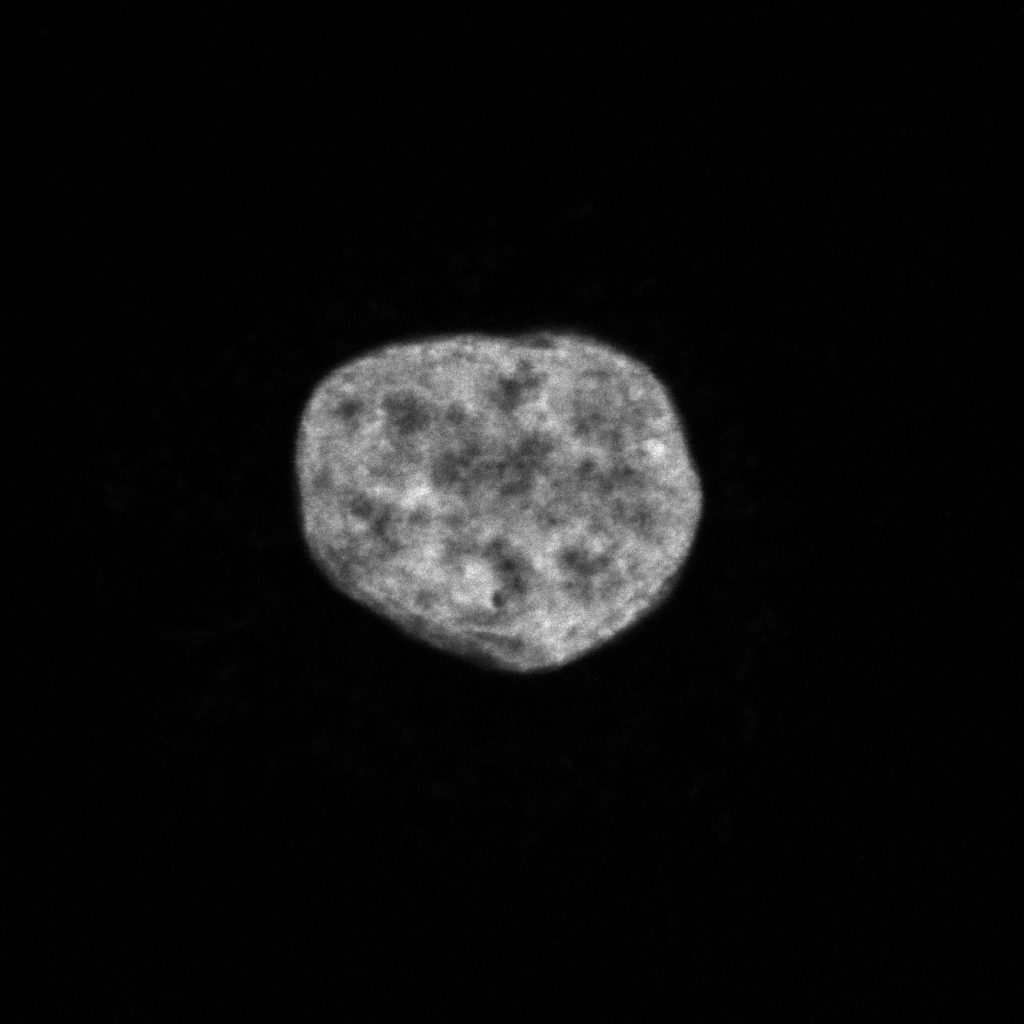

Supplement: Supplementary file 4 — Source data Fig. 2 [file 44318_2025_672_MOESM4_ESM.zip › Figure 2/2D/K130R addback_DAPI.tif]

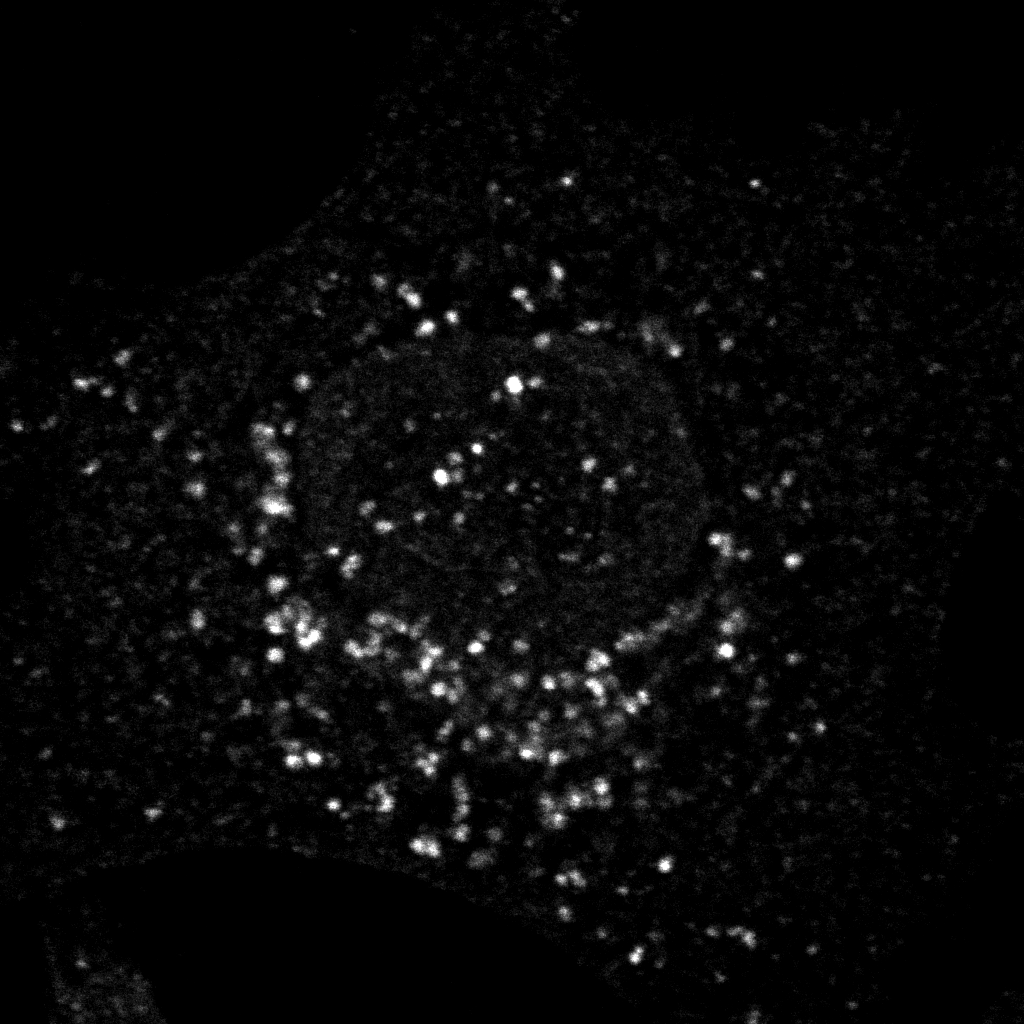

Supplement: Supplementary file 4 — Source data Fig. 2 [file 44318_2025_672_MOESM4_ESM.zip › Figure 2/2D/K130R addback_Gal3.tif]

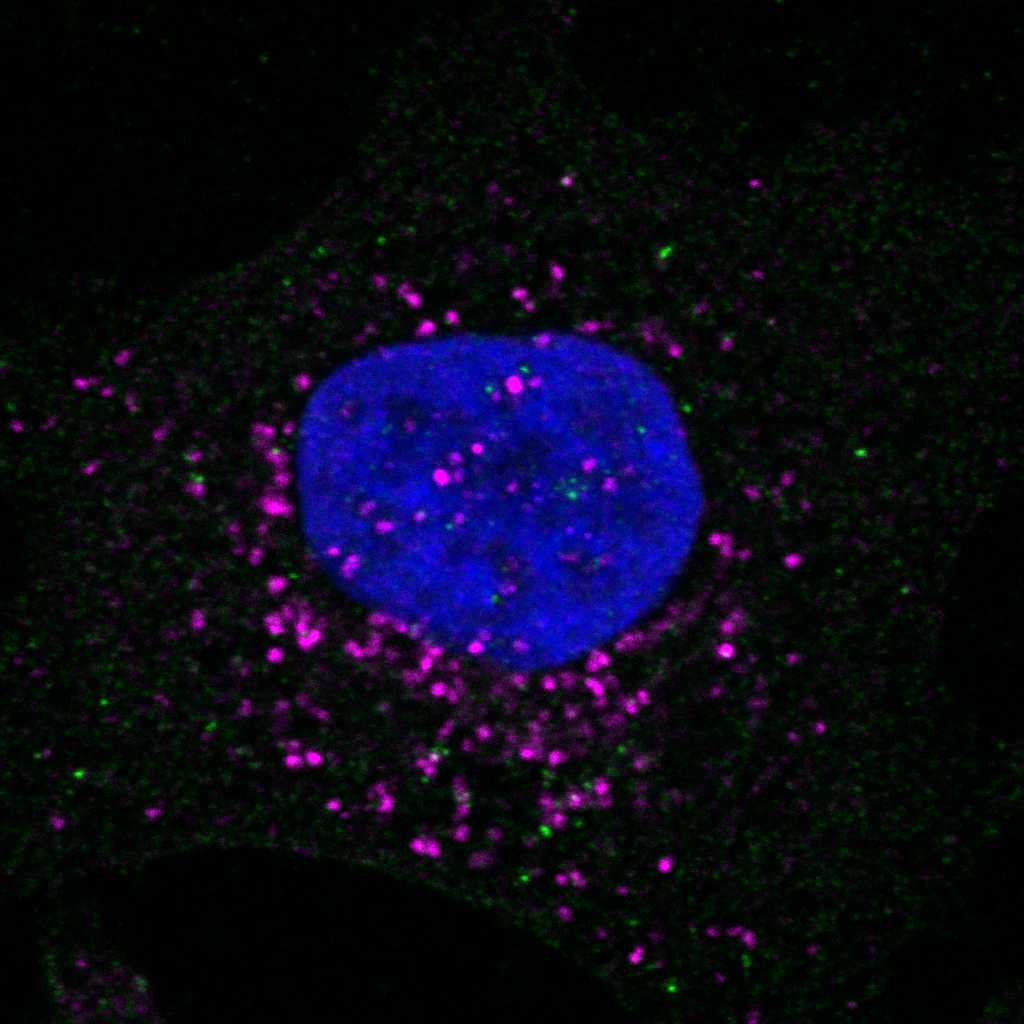

Supplement: Supplementary file 4 — Source data Fig. 2 [file 44318_2025_672_MOESM4_ESM.zip › Figure 2/2D/K130R addback_merge.tif]

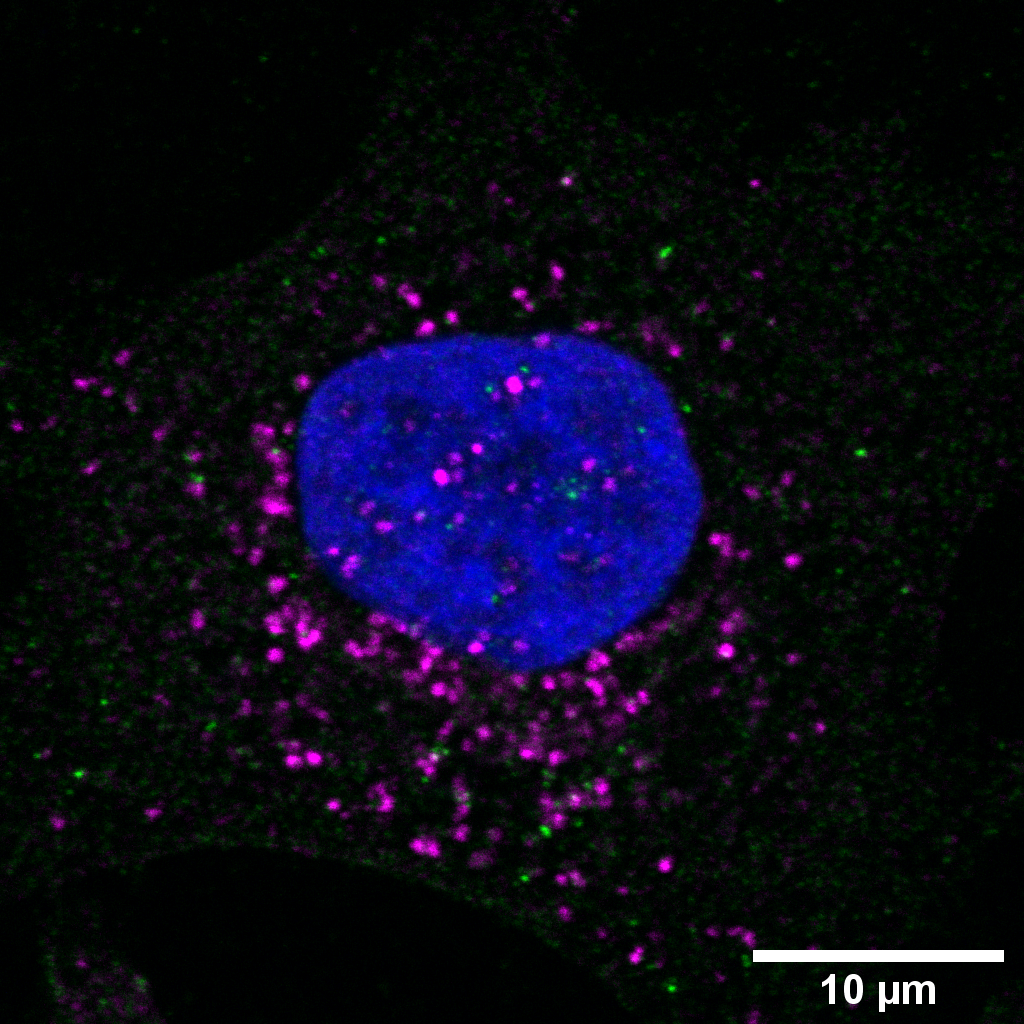

Supplement: Supplementary file 4 — Source data Fig. 2 [file 44318_2025_672_MOESM4_ESM.zip › Figure 2/2D/K130R addback_scale.tif]

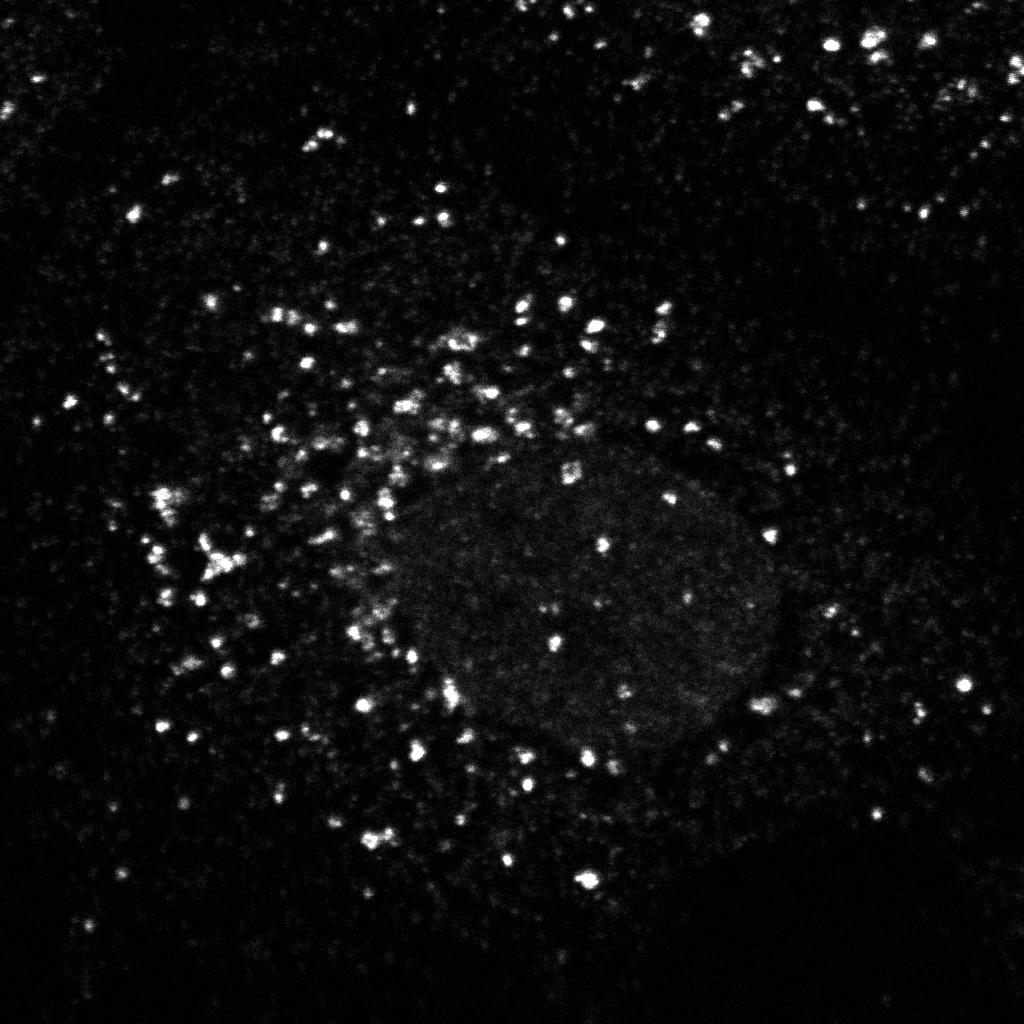

Supplement: Supplementary file 4 — Source data Fig. 2 [file 44318_2025_672_MOESM4_ESM.zip › Figure 2/2D/WT addback_ALIX.tif]

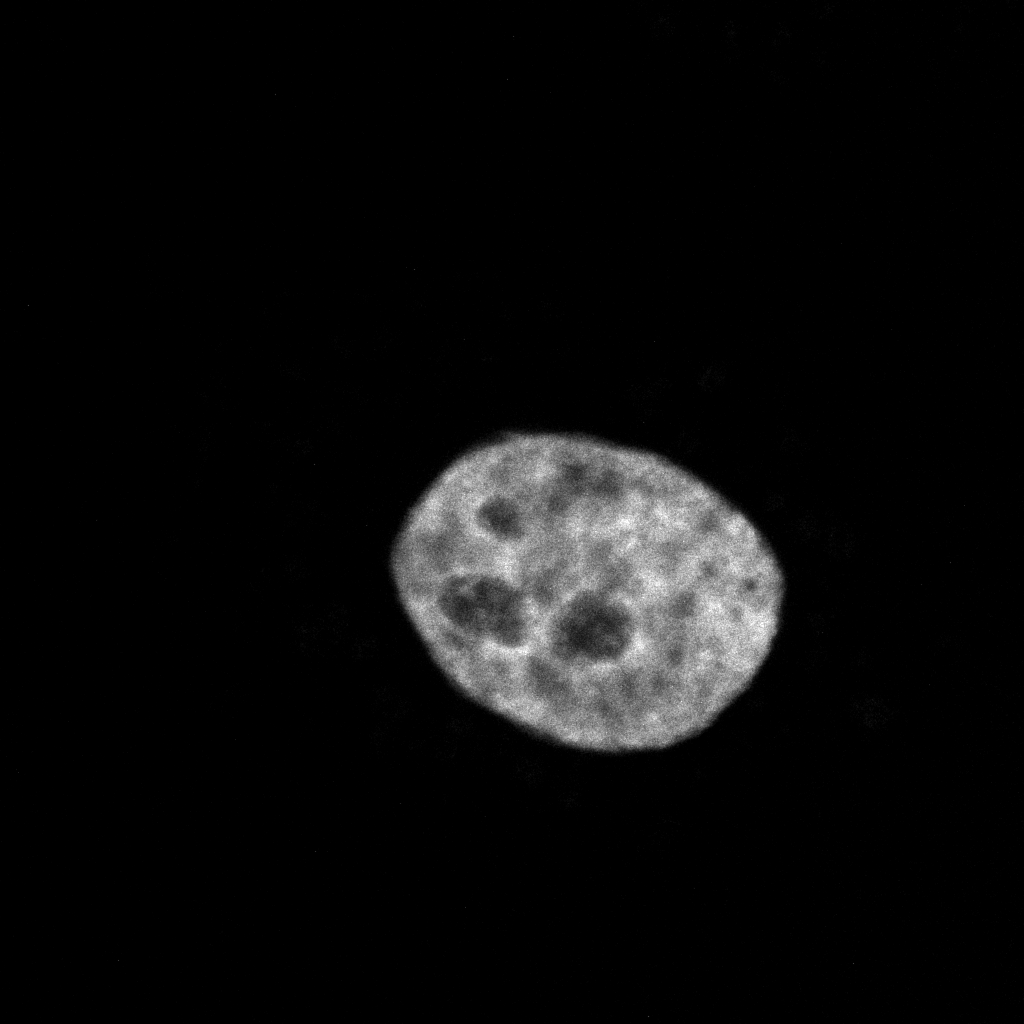

Supplement: Supplementary file 4 — Source data Fig. 2 [file 44318_2025_672_MOESM4_ESM.zip › Figure 2/2D/WT addback_DAPI.tif]

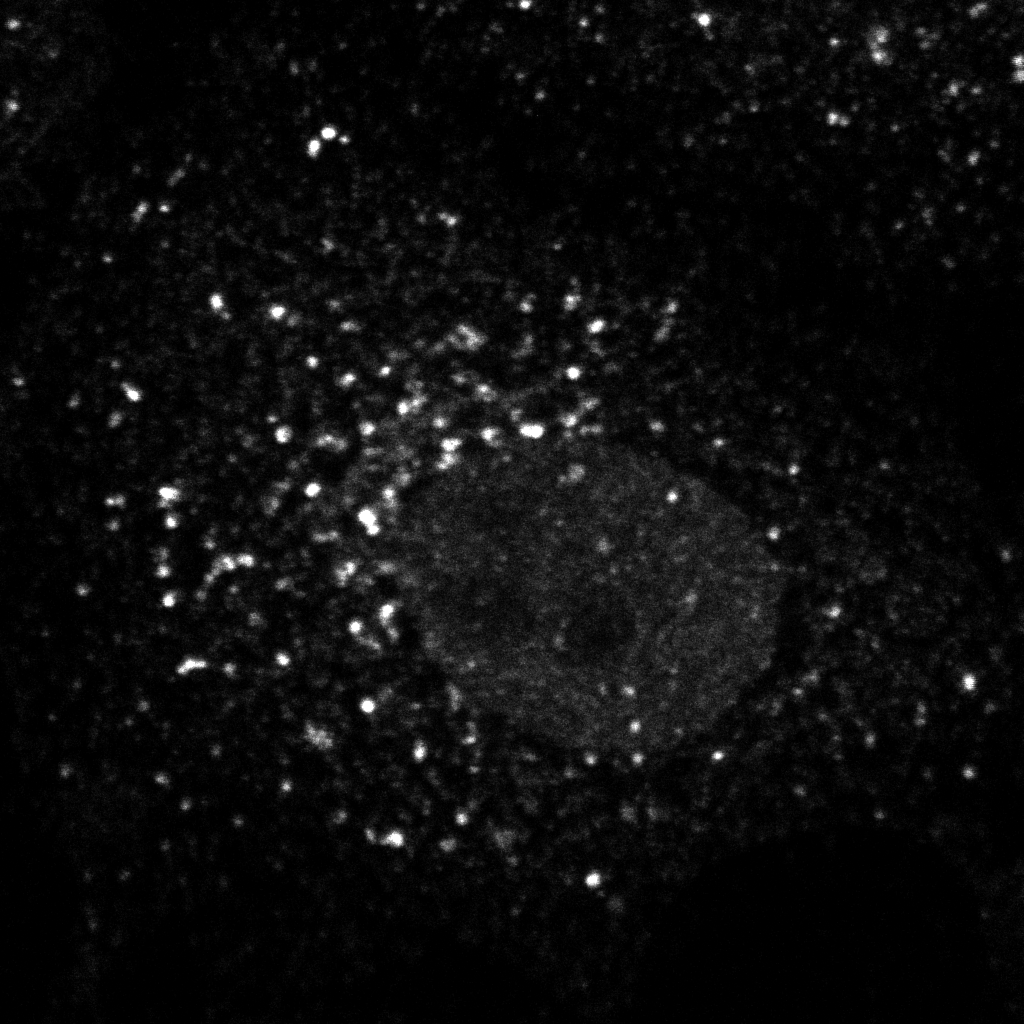

Supplement: Supplementary file 4 — Source data Fig. 2 [file 44318_2025_672_MOESM4_ESM.zip › Figure 2/2D/WT addback_Gal3.tif]

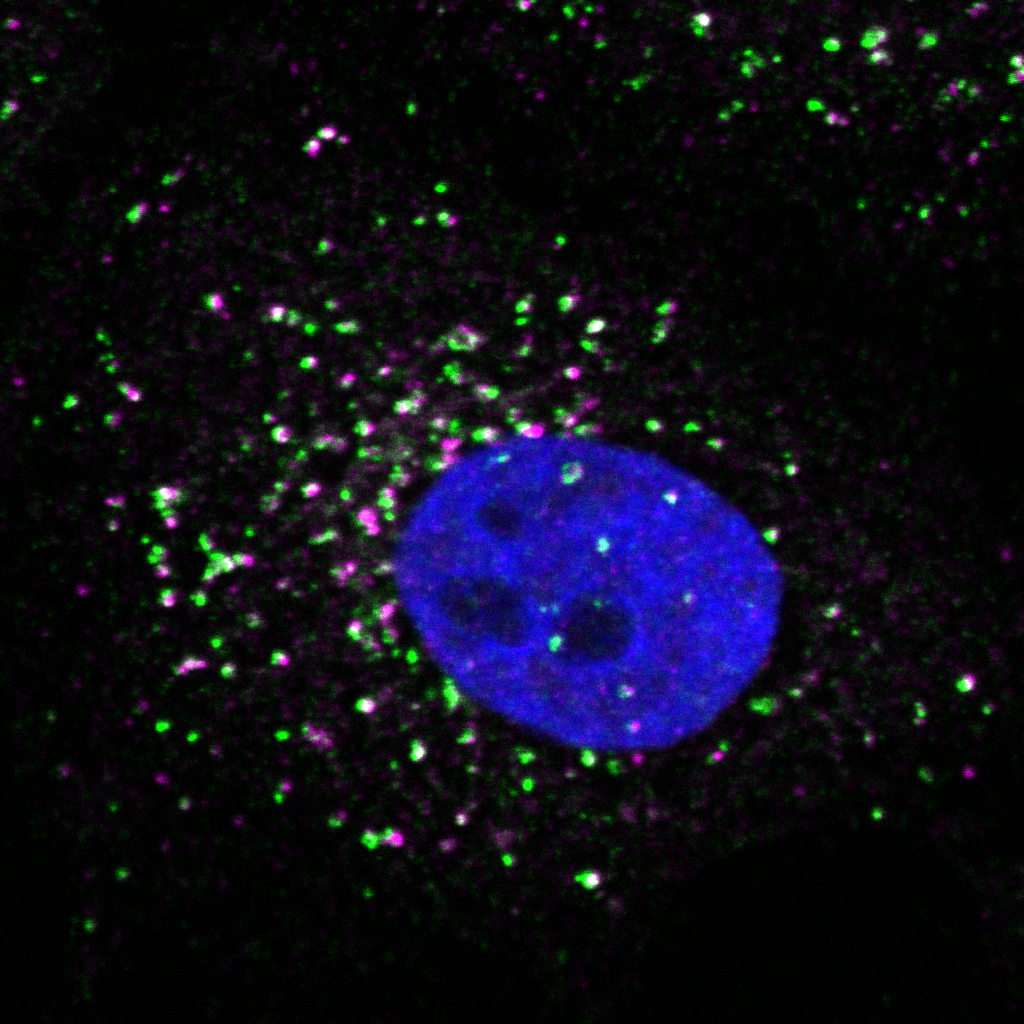

Supplement: Supplementary file 4 — Source data Fig. 2 [file 44318_2025_672_MOESM4_ESM.zip › Figure 2/2D/WT addback_merge.tif]

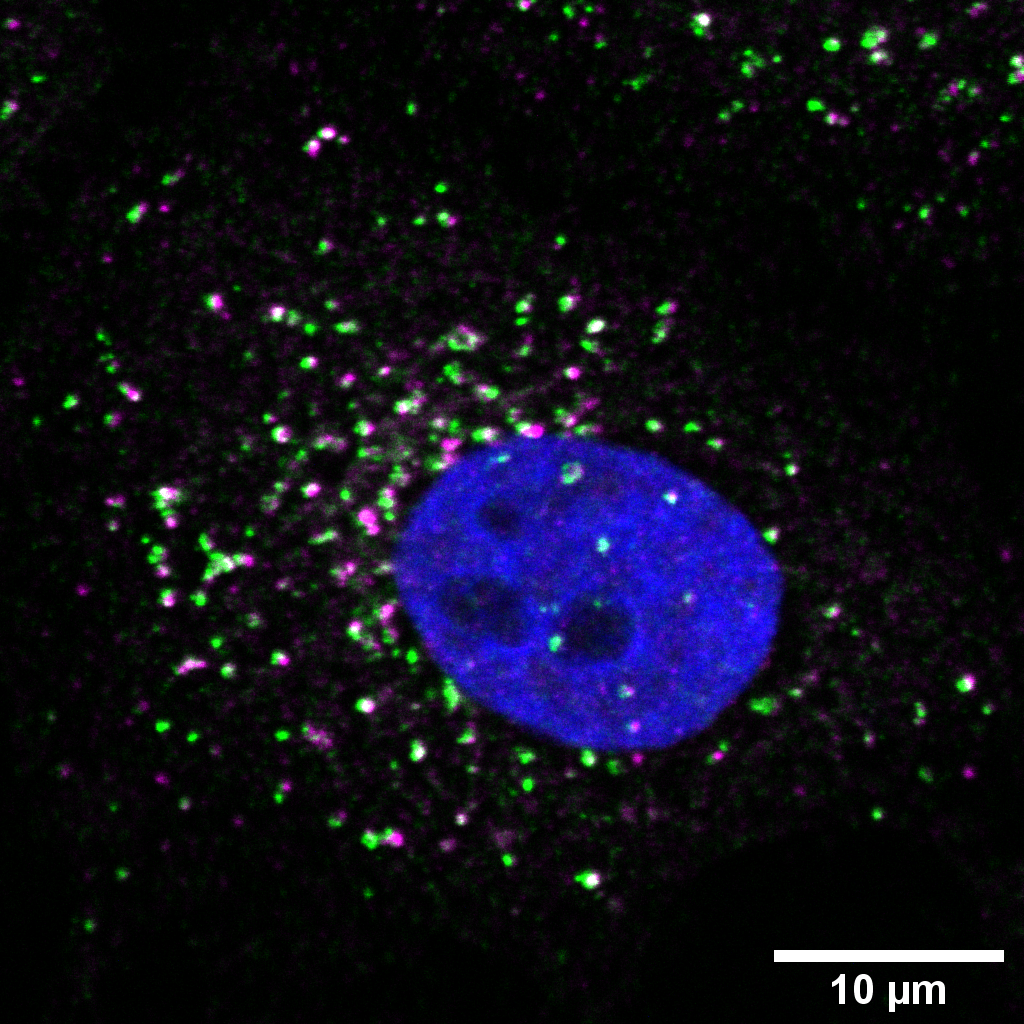

Supplement: Supplementary file 4 — Source data Fig. 2 [file 44318_2025_672_MOESM4_ESM.zip › Figure 2/2D/WT addback_scale.tif]

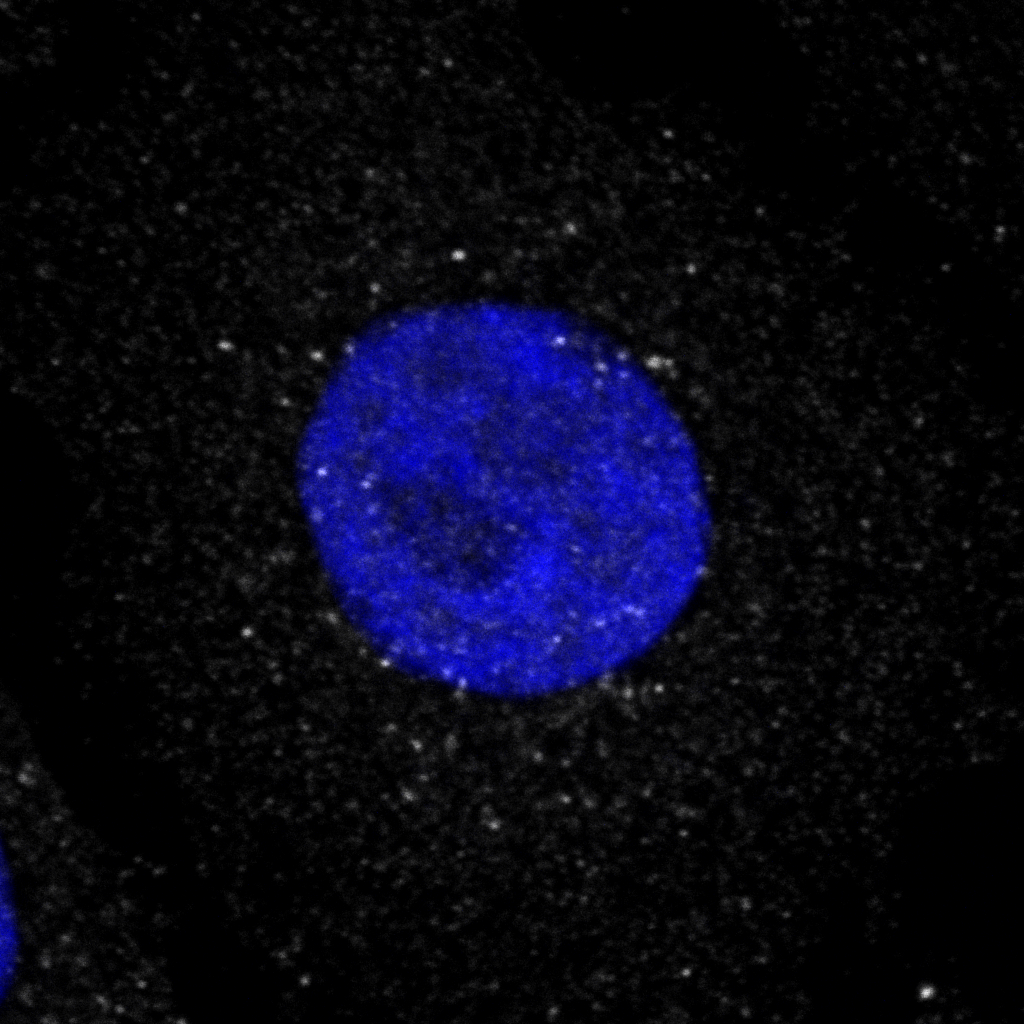

Supplement: Supplementary file 5 — Source data Fig. 3 [file 44318_2025_672_MOESM5_ESM.zip › Figure 3/3A/5KO_CHMP.tif]

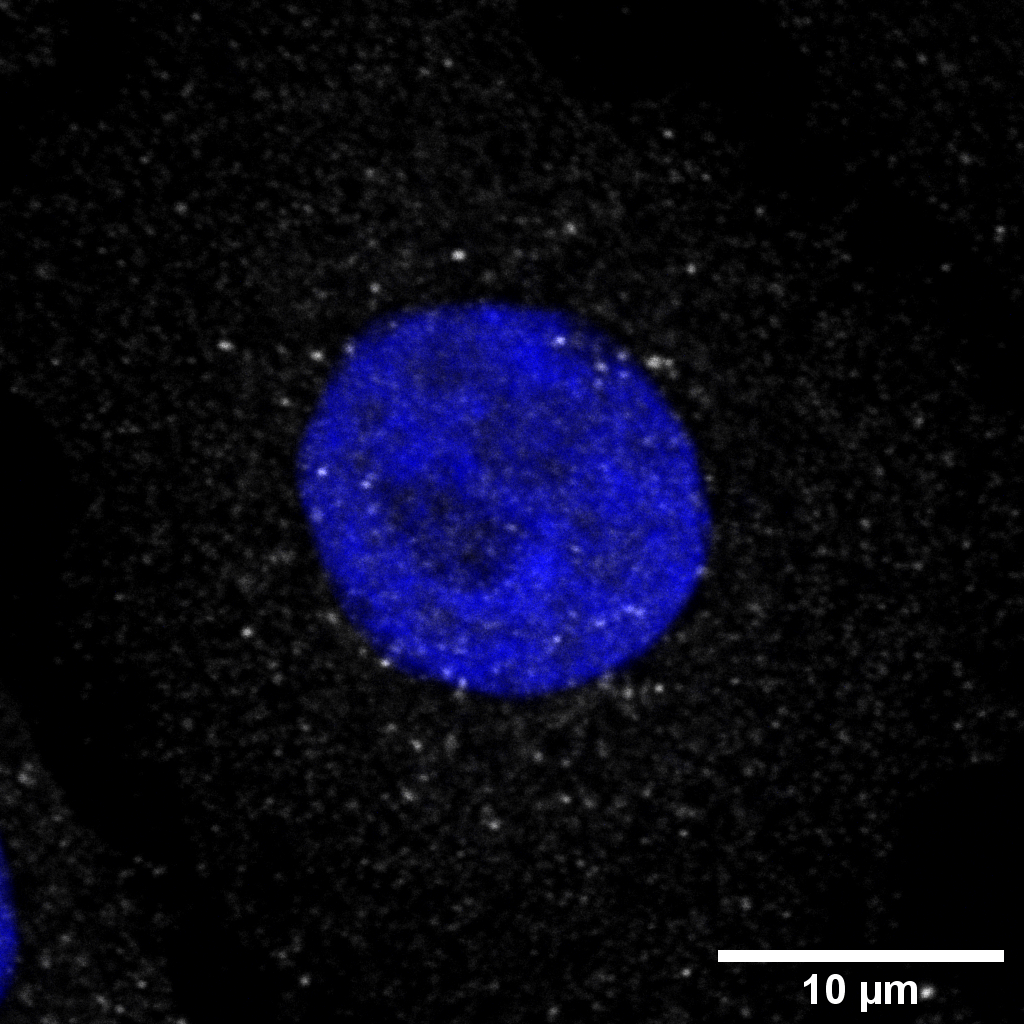

Supplement: Supplementary file 5 — Source data Fig. 3 [file 44318_2025_672_MOESM5_ESM.zip › Figure 3/3A/5KO_CHMP_scale.tif]

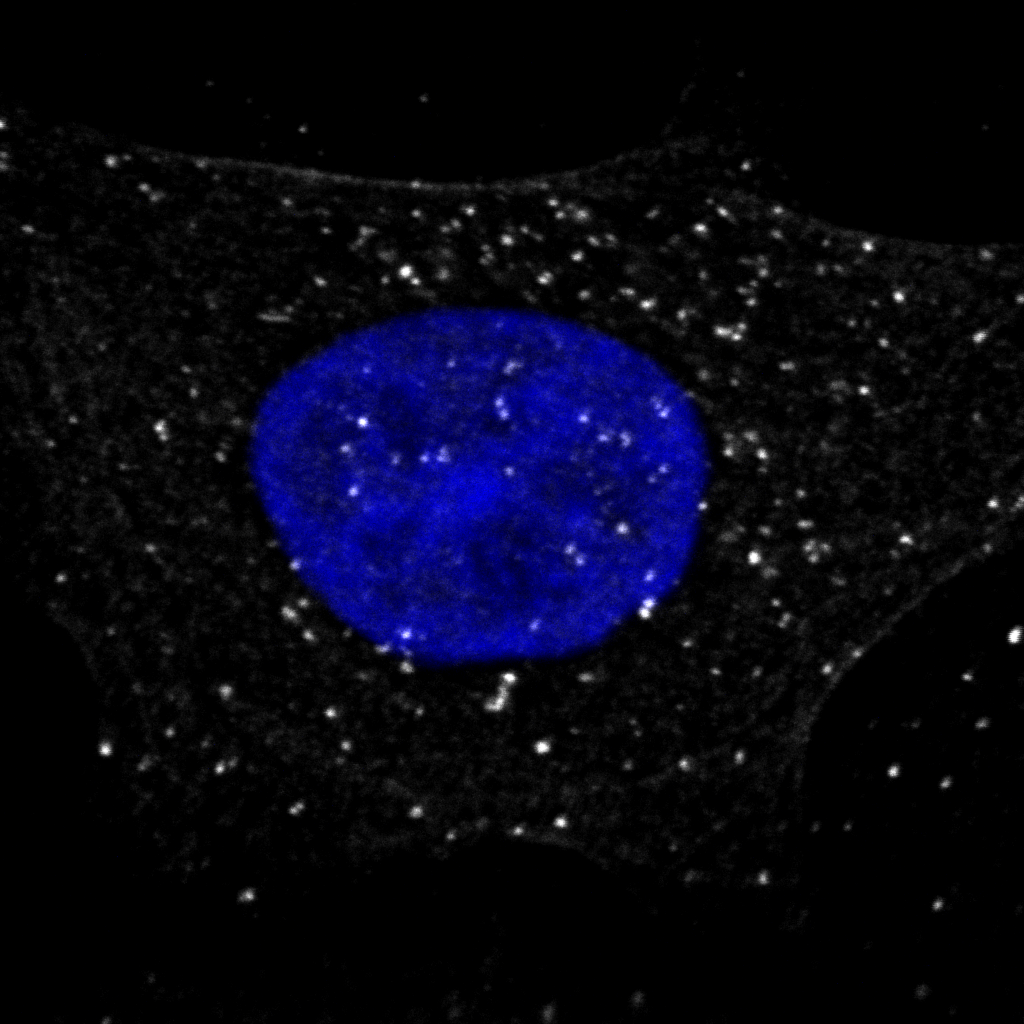

Supplement: Supplementary file 5 — Source data Fig. 3 [file 44318_2025_672_MOESM5_ESM.zip › Figure 3/3A/5KO_IST1.tif]
