# Supplementary figures and images for: The ATG8 E3-like ligases sense lysosomal damage and initiate ESCRT-mediated membrane repair (part 1 of 7)
Source: EMBO J. 2026 Jan 3;45(3):930–52. doi: 10.1038/s44318-025-00672-1 (PMC12865045; doi:10.1038/s44318-025-00672-1)

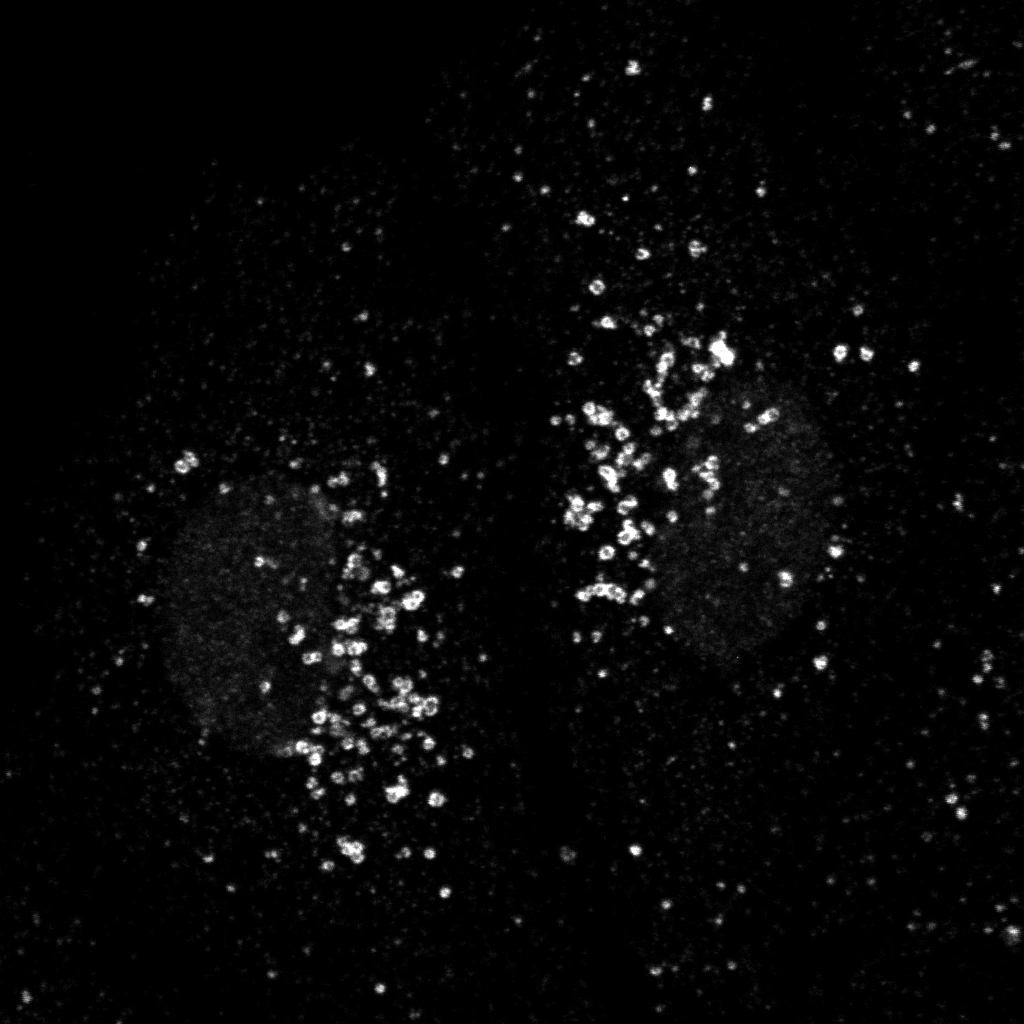

Supplement: Supplementary file 3 — Source data Fig. 1 [file 44318_2025_672_MOESM3_ESM.zip › Figure 1/1A/16KO_ALIX.tif]

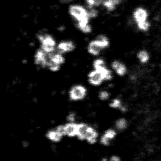

Supplement: Supplementary file 3 — Source data Fig. 1 [file 44318_2025_672_MOESM3_ESM.zip › Figure 1/1A/16KO_ALIX_zoom.tif]

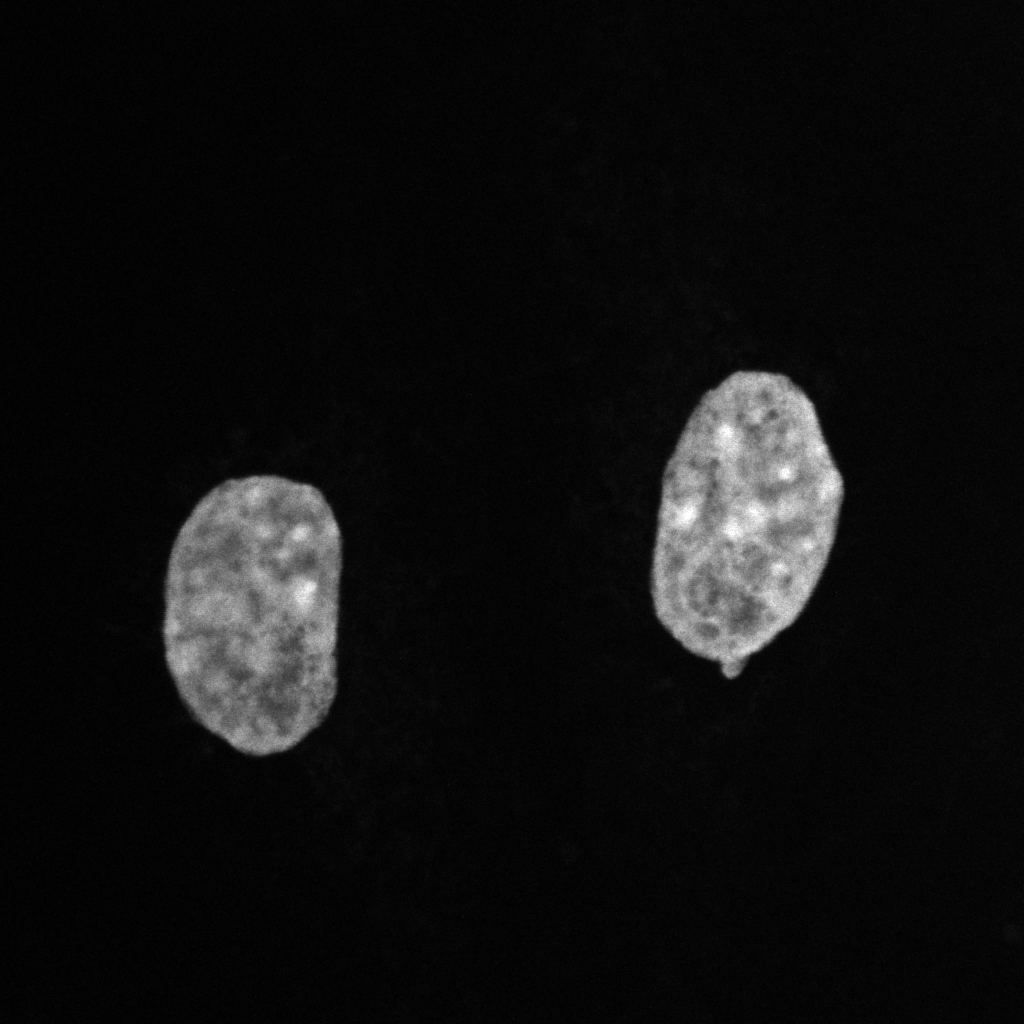

Supplement: Supplementary file 3 — Source data Fig. 1 [file 44318_2025_672_MOESM3_ESM.zip › Figure 1/1A/16KO_DAPI.tif]

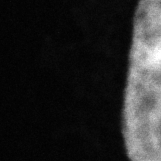

Supplement: Supplementary file 3 — Source data Fig. 1 [file 44318_2025_672_MOESM3_ESM.zip › Figure 1/1A/16KO_DAPI_zoom.tif]

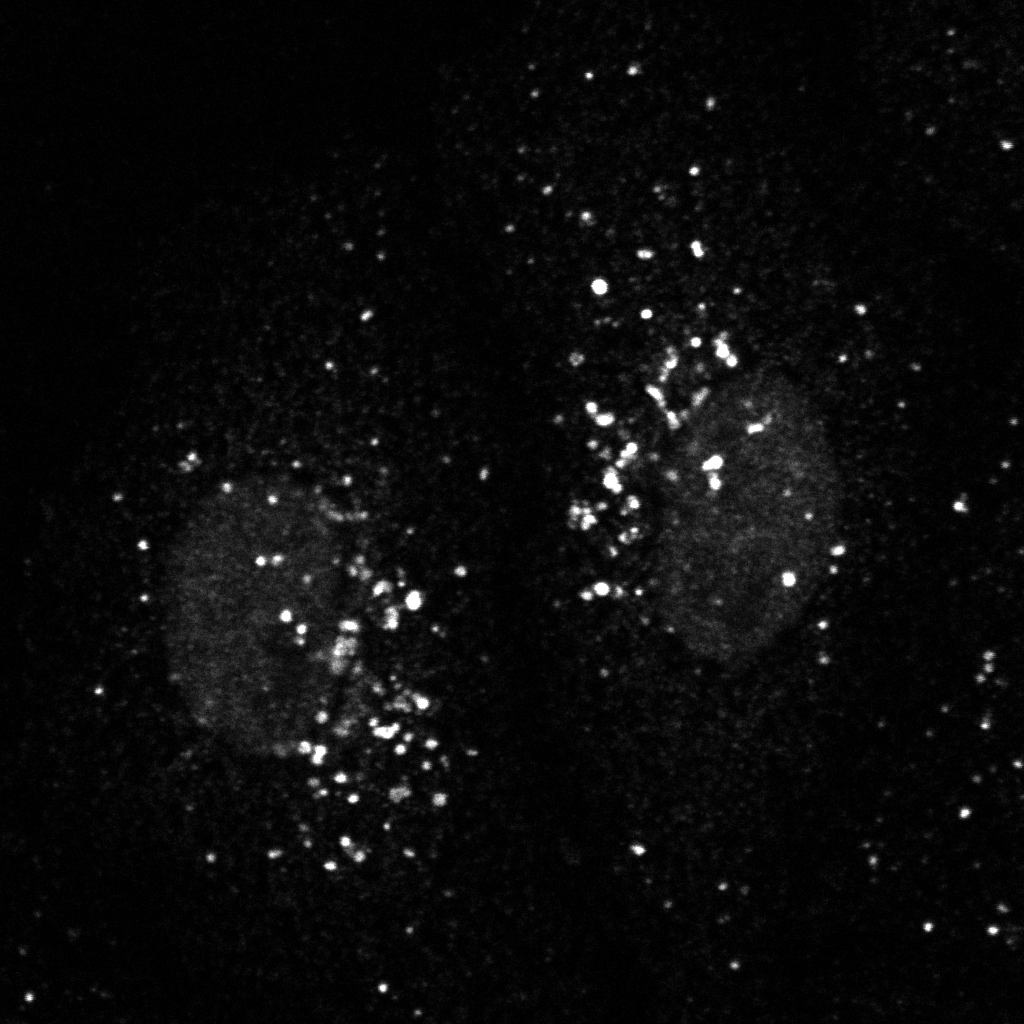

Supplement: Supplementary file 3 — Source data Fig. 1 [file 44318_2025_672_MOESM3_ESM.zip › Figure 1/1A/16KO_GAL3.tif]

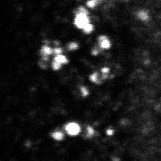

Supplement: Supplementary file 3 — Source data Fig. 1 [file 44318_2025_672_MOESM3_ESM.zip › Figure 1/1A/16KO_GAL3_zoom.tif]

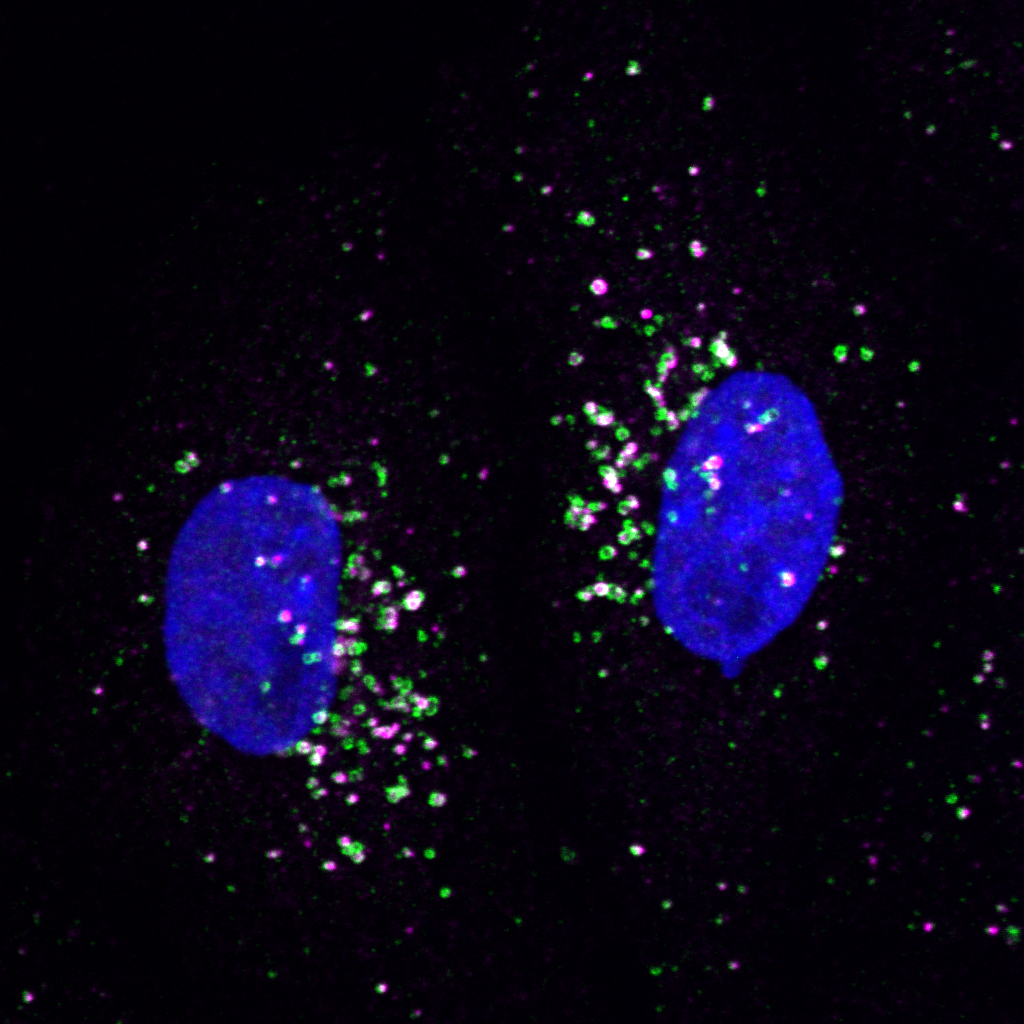

Supplement: Supplementary file 3 — Source data Fig. 1 [file 44318_2025_672_MOESM3_ESM.zip › Figure 1/1A/16KO_merge.tif]

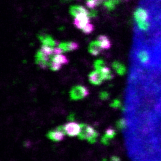

Supplement: Supplementary file 3 — Source data Fig. 1 [file 44318_2025_672_MOESM3_ESM.zip › Figure 1/1A/16KO_zoom.tif]

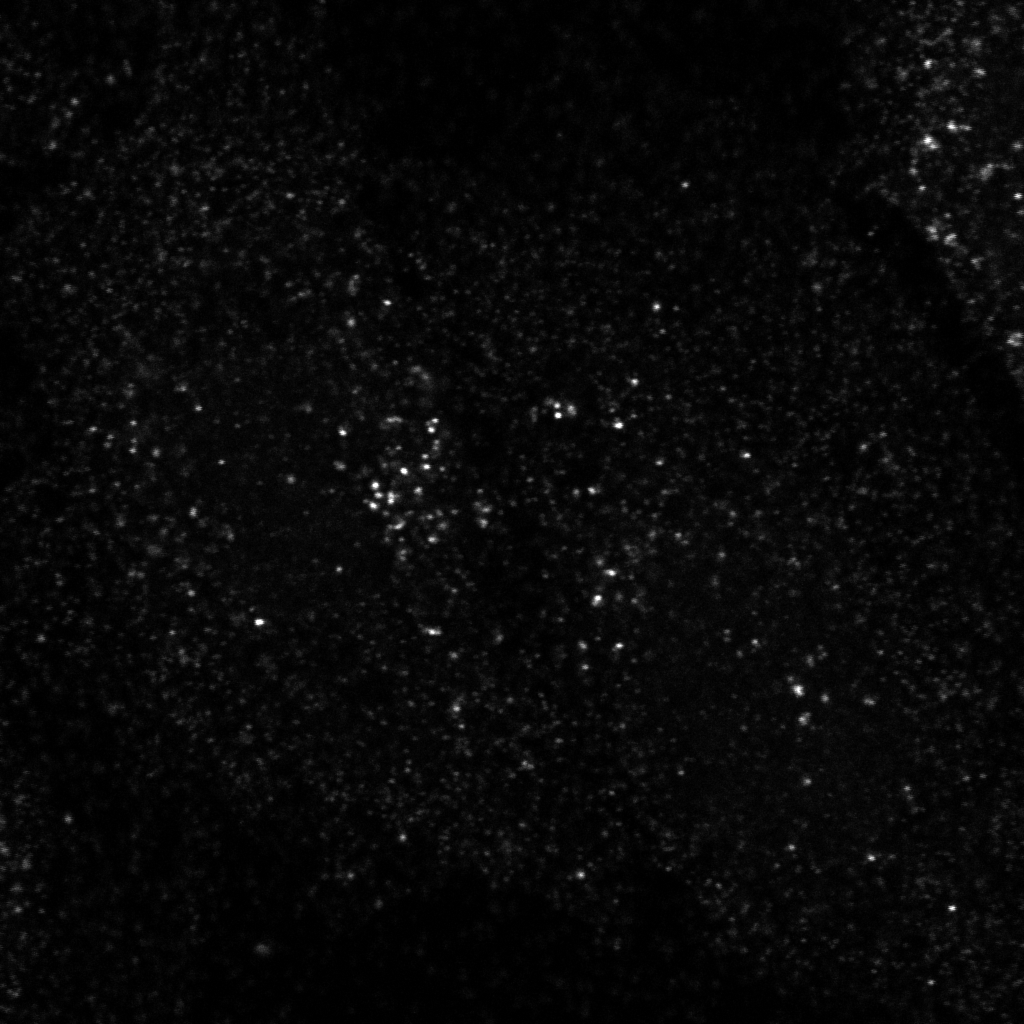

Supplement: Supplementary file 3 — Source data Fig. 1 [file 44318_2025_672_MOESM3_ESM.zip › Figure 1/1A/DKO_LLOMe_ALIX.tif]

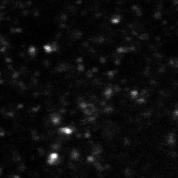

Supplement: Supplementary file 3 — Source data Fig. 1 [file 44318_2025_672_MOESM3_ESM.zip › Figure 1/1A/DKO_LLOMe_ALIX_zoom.tif]

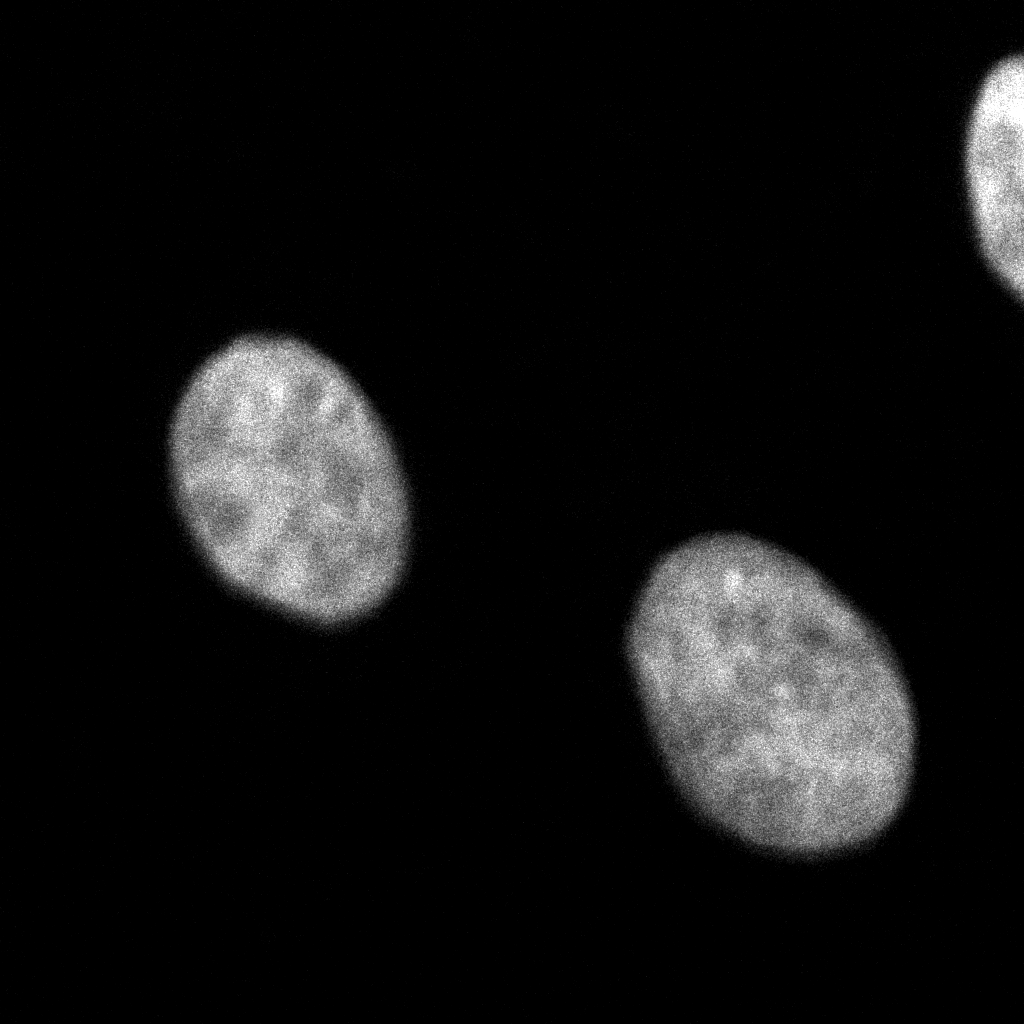

Supplement: Supplementary file 3 — Source data Fig. 1 [file 44318_2025_672_MOESM3_ESM.zip › Figure 1/1A/DKO_LLOMe_DAPI.tif]

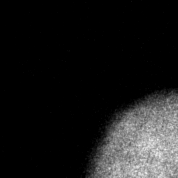

Supplement: Supplementary file 3 — Source data Fig. 1 [file 44318_2025_672_MOESM3_ESM.zip › Figure 1/1A/DKO_LLOMe_DAPI_zoom.tif]

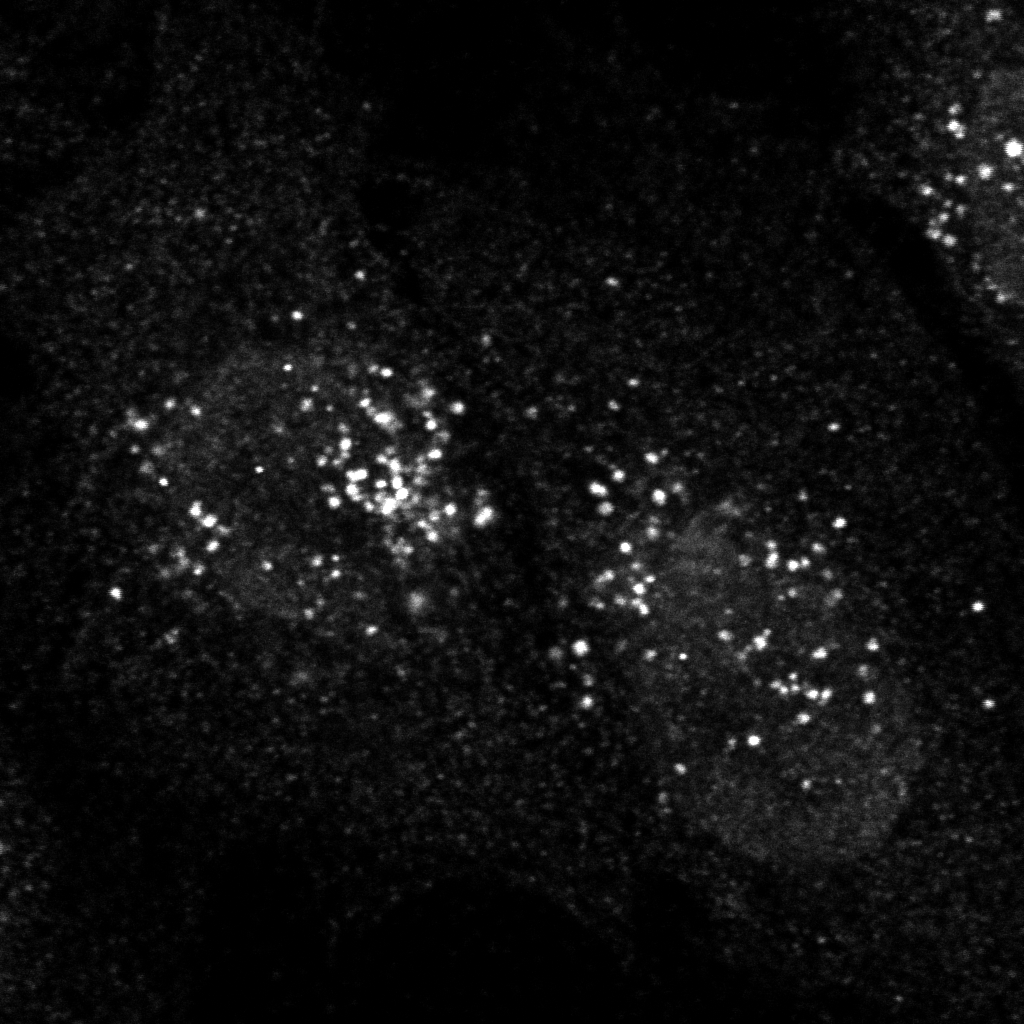

Supplement: Supplementary file 3 — Source data Fig. 1 [file 44318_2025_672_MOESM3_ESM.zip › Figure 1/1A/DKO_LLOMe_Gal3.tif]

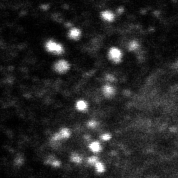

Supplement: Supplementary file 3 — Source data Fig. 1 [file 44318_2025_672_MOESM3_ESM.zip › Figure 1/1A/DKO_LLOMe_Gal3_zoom.tif]

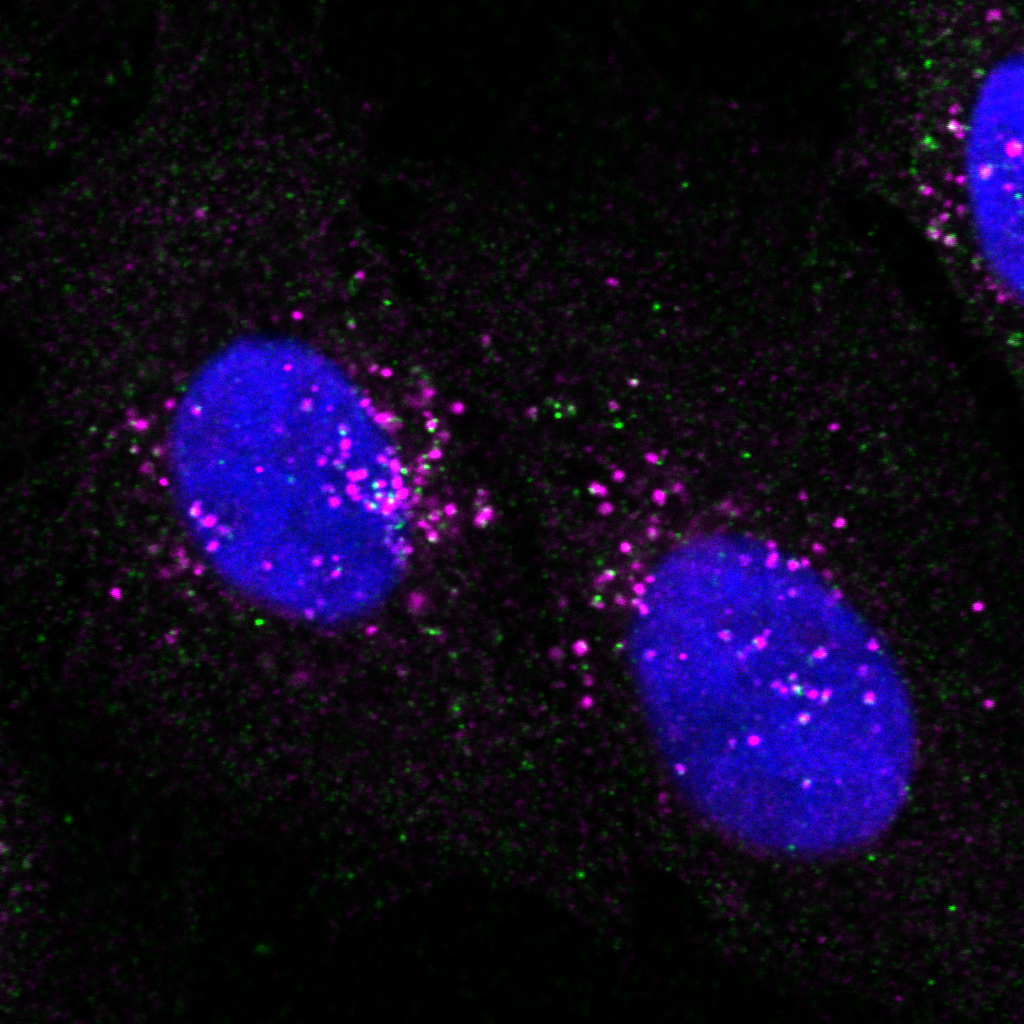

Supplement: Supplementary file 3 — Source data Fig. 1 [file 44318_2025_672_MOESM3_ESM.zip › Figure 1/1A/DKO_LLOMe_Merge.tif]

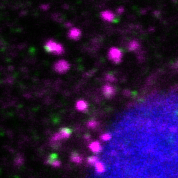

Supplement: Supplementary file 3 — Source data Fig. 1 [file 44318_2025_672_MOESM3_ESM.zip › Figure 1/1A/DKO_LLOMe_Merge_zoom.tif]

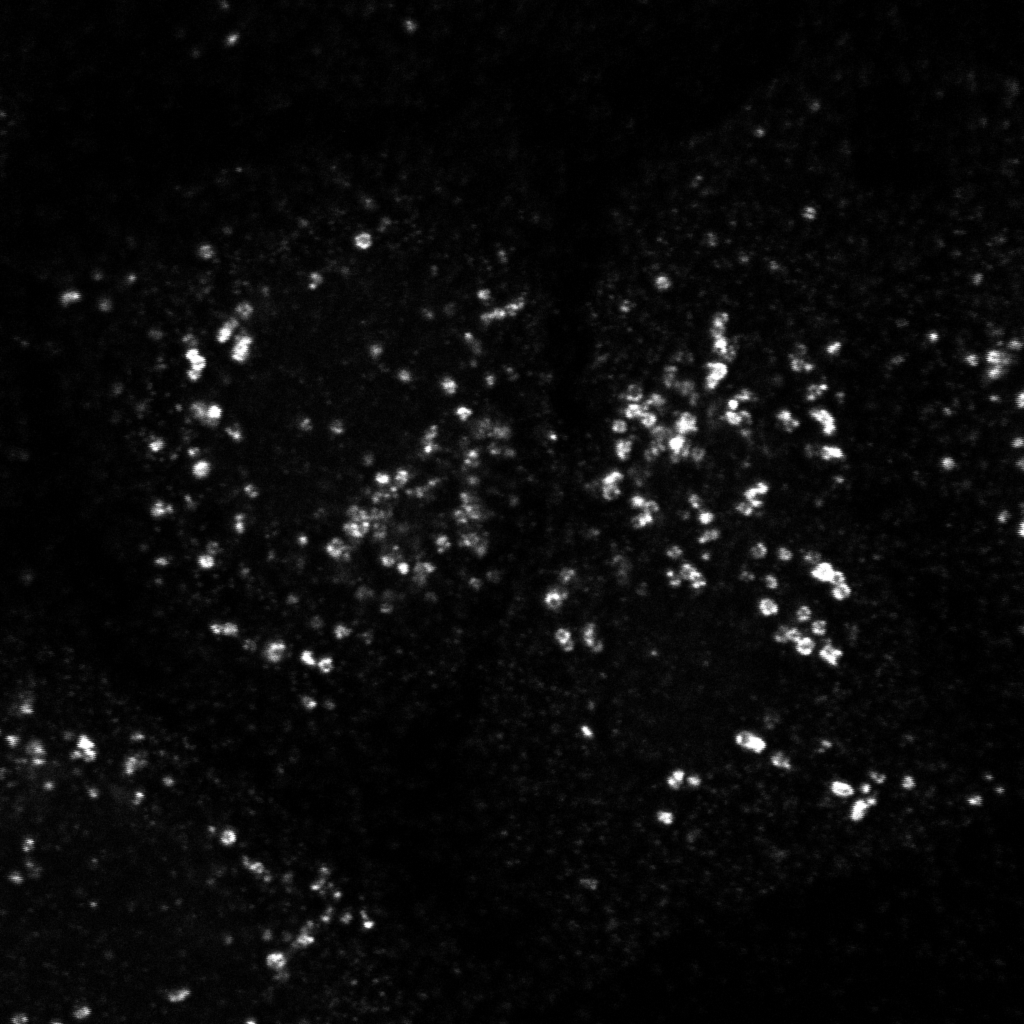

Supplement: Supplementary file 3 — Source data Fig. 1 [file 44318_2025_672_MOESM3_ESM.zip › Figure 1/1A/TECPRKO_LLOMe_ALIX.tif]

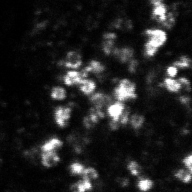

Supplement: Supplementary file 3 — Source data Fig. 1 [file 44318_2025_672_MOESM3_ESM.zip › Figure 1/1A/TECPRKO_LLOMe_ALIX_zoom.tif]

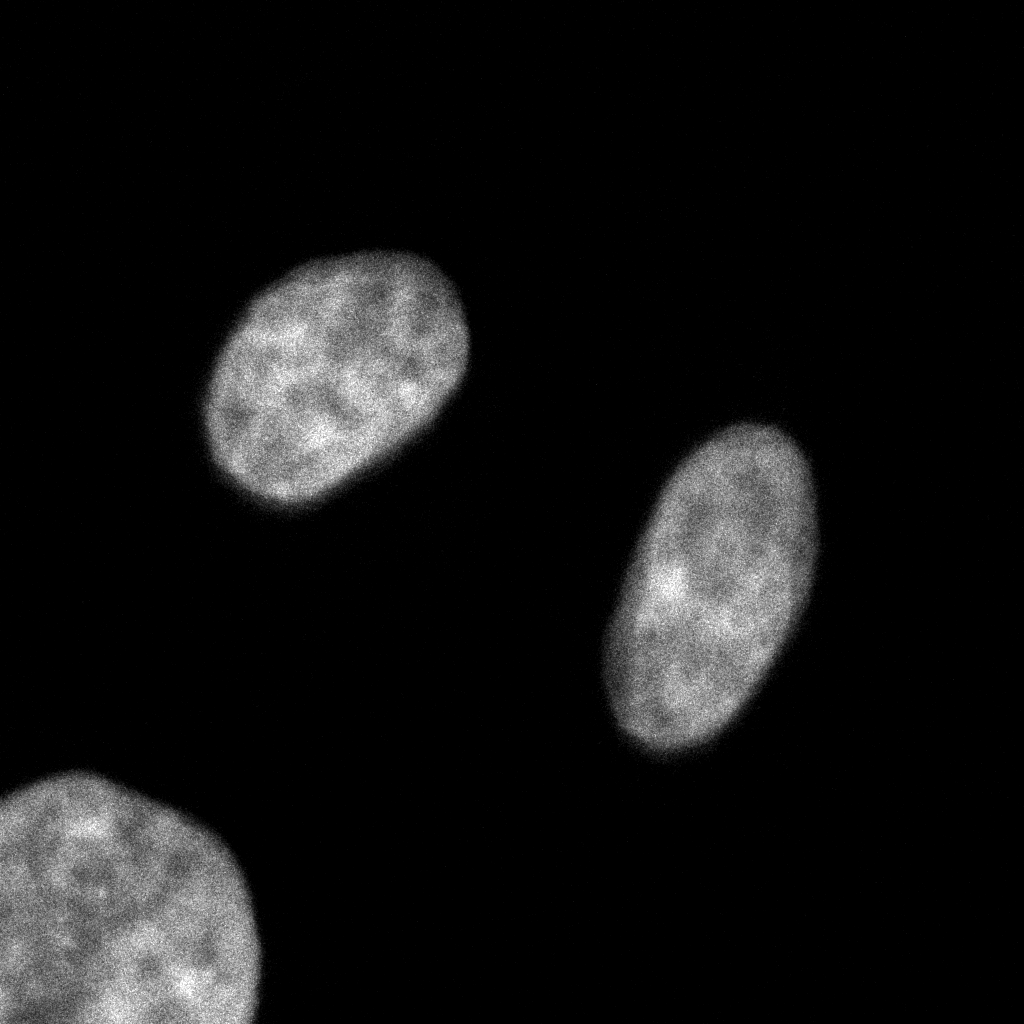

Supplement: Supplementary file 3 — Source data Fig. 1 [file 44318_2025_672_MOESM3_ESM.zip › Figure 1/1A/TECPRKO_LLOMe_DAPI.tif]

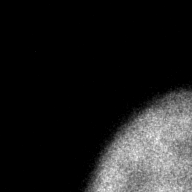

Supplement: Supplementary file 3 — Source data Fig. 1 [file 44318_2025_672_MOESM3_ESM.zip › Figure 1/1A/TECPRKO_LLOMe_DAPI_zoom.tif]

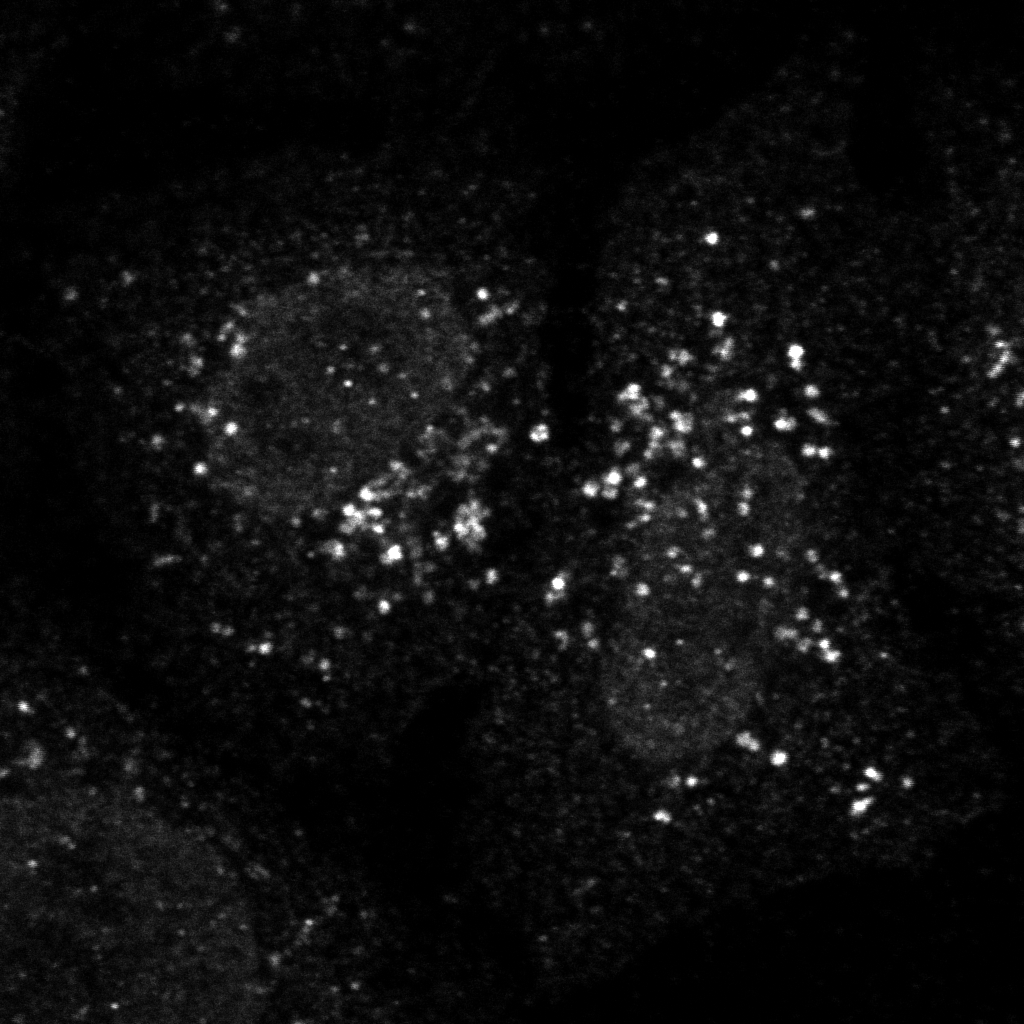

Supplement: Supplementary file 3 — Source data Fig. 1 [file 44318_2025_672_MOESM3_ESM.zip › Figure 1/1A/TECPRKO_LLOMe_Gal3.tif]

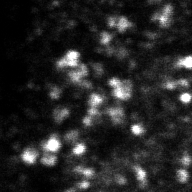

Supplement: Supplementary file 3 — Source data Fig. 1 [file 44318_2025_672_MOESM3_ESM.zip › Figure 1/1A/TECPRKO_LLOMe_Gal3_zoom.tif]

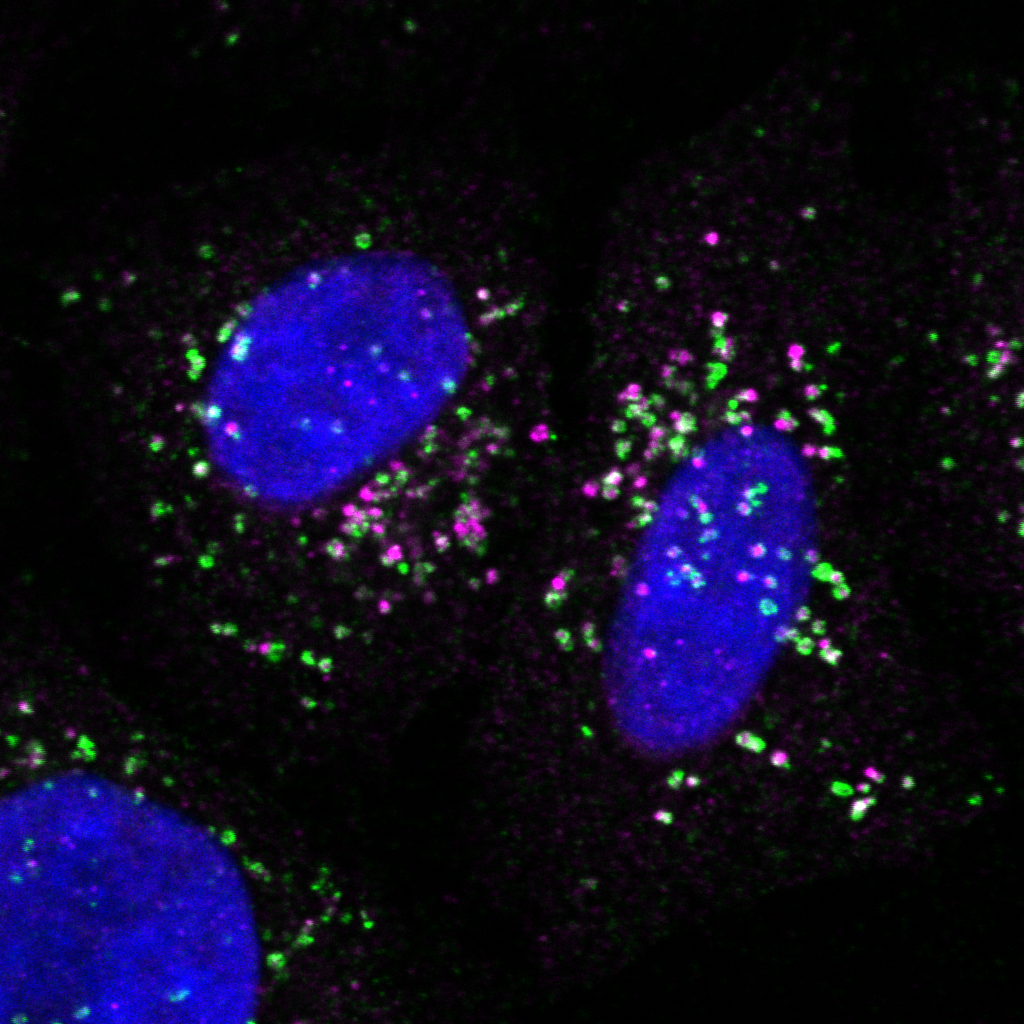

Supplement: Supplementary file 3 — Source data Fig. 1 [file 44318_2025_672_MOESM3_ESM.zip › Figure 1/1A/TECPRKO_LLOMe_Merge.tif]

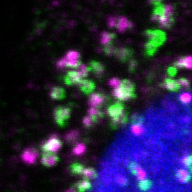

Supplement: Supplementary file 3 — Source data Fig. 1 [file 44318_2025_672_MOESM3_ESM.zip › Figure 1/1A/TECPRKO_LLOMe_Merge_zoom.tif]

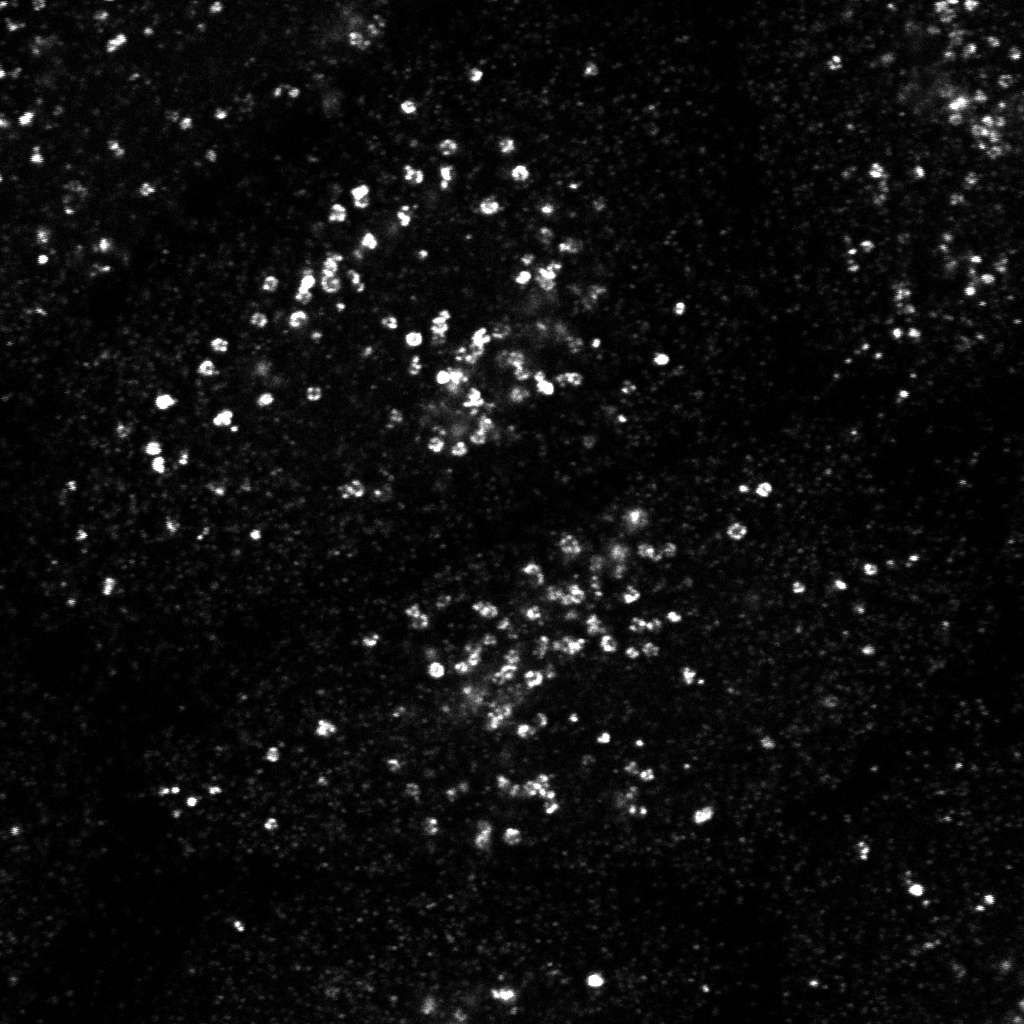

Supplement: Supplementary file 3 — Source data Fig. 1 [file 44318_2025_672_MOESM3_ESM.zip › Figure 1/1A/WT_LLOMe_ALIX.tif]

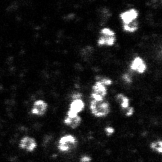

Supplement: Supplementary file 3 — Source data Fig. 1 [file 44318_2025_672_MOESM3_ESM.zip › Figure 1/1A/WT_LLOMe_ALIX_zoom.tif]

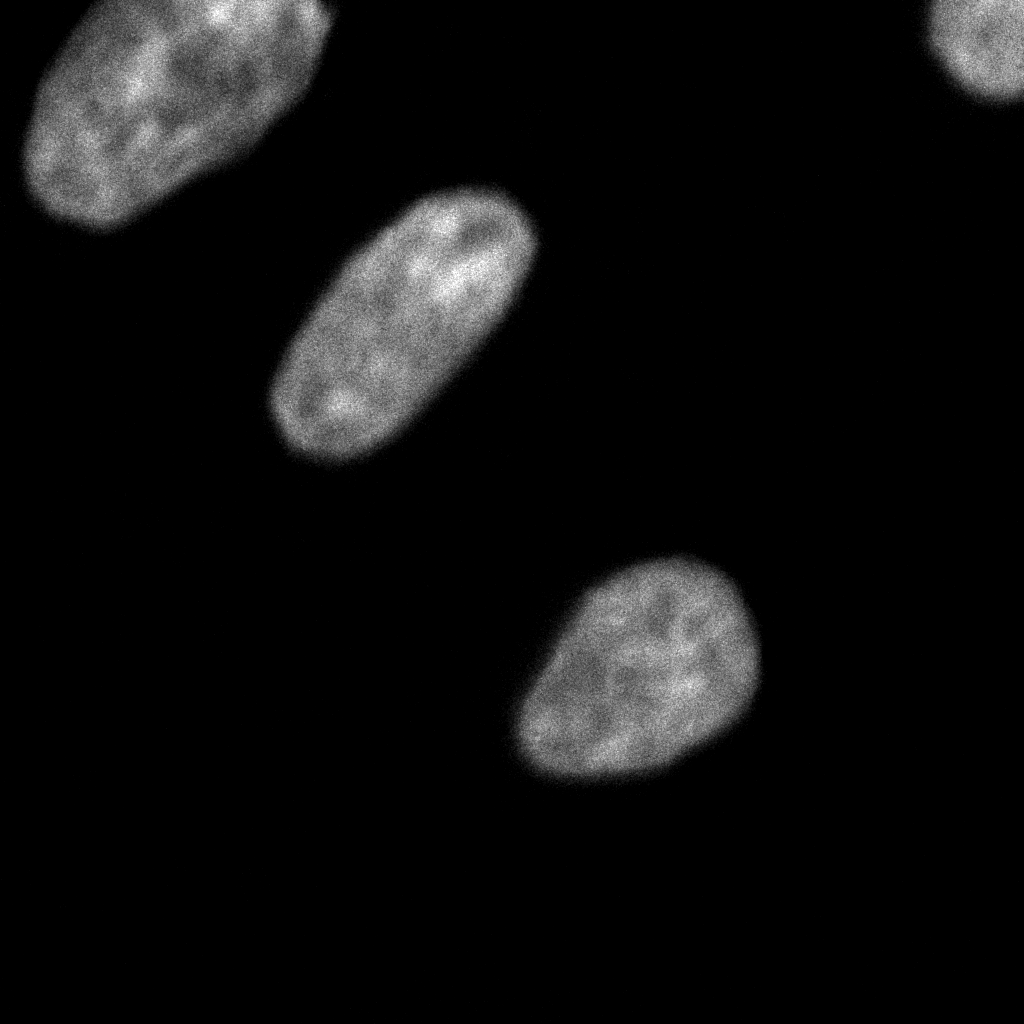

Supplement: Supplementary file 3 — Source data Fig. 1 [file 44318_2025_672_MOESM3_ESM.zip › Figure 1/1A/WT_LLOMe_DAPI.tif]

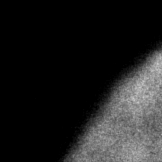

Supplement: Supplementary file 3 — Source data Fig. 1 [file 44318_2025_672_MOESM3_ESM.zip › Figure 1/1A/WT_LLOMe_DAPI_zoom.tif]

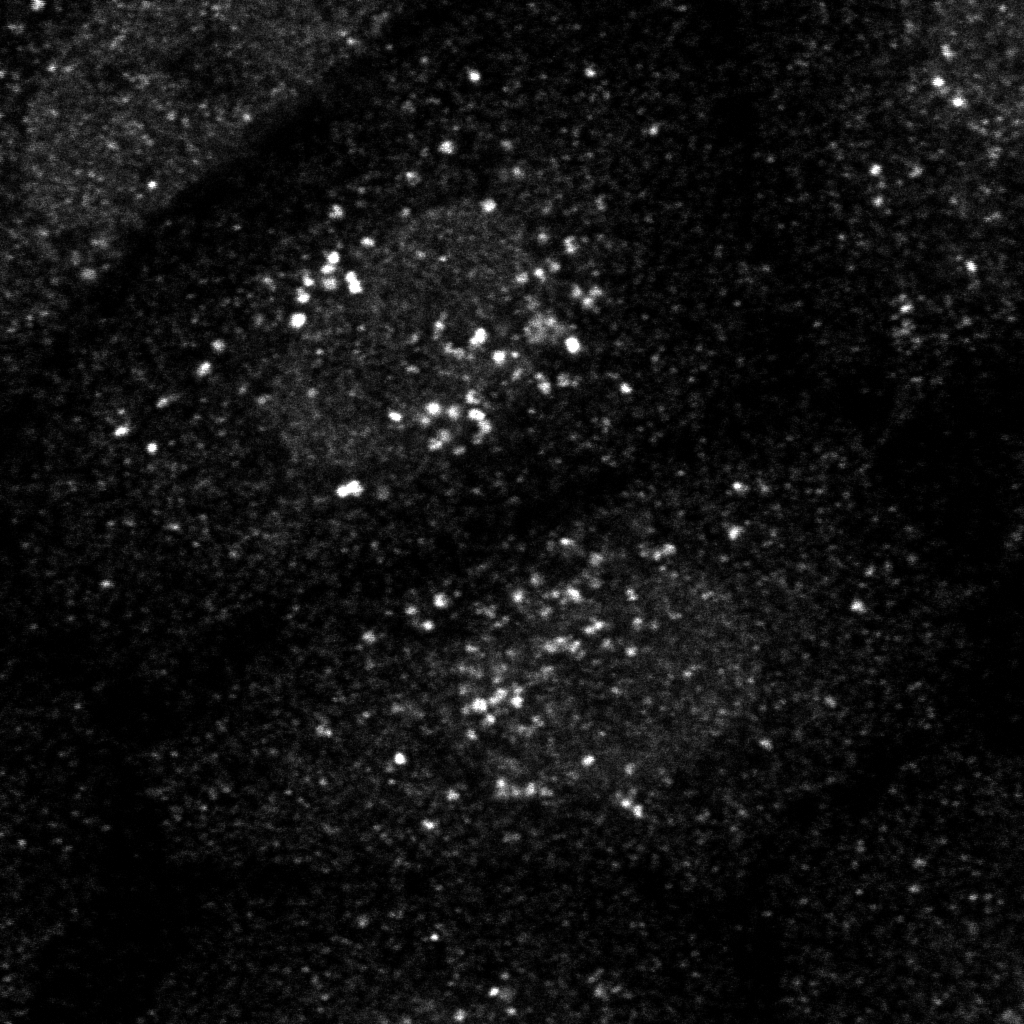

Supplement: Supplementary file 3 — Source data Fig. 1 [file 44318_2025_672_MOESM3_ESM.zip › Figure 1/1A/WT_LLOMe_Gal3.tif]

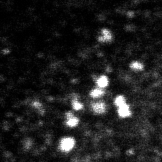

Supplement: Supplementary file 3 — Source data Fig. 1 [file 44318_2025_672_MOESM3_ESM.zip › Figure 1/1A/WT_LLOMe_Gal3_zoom.tif]

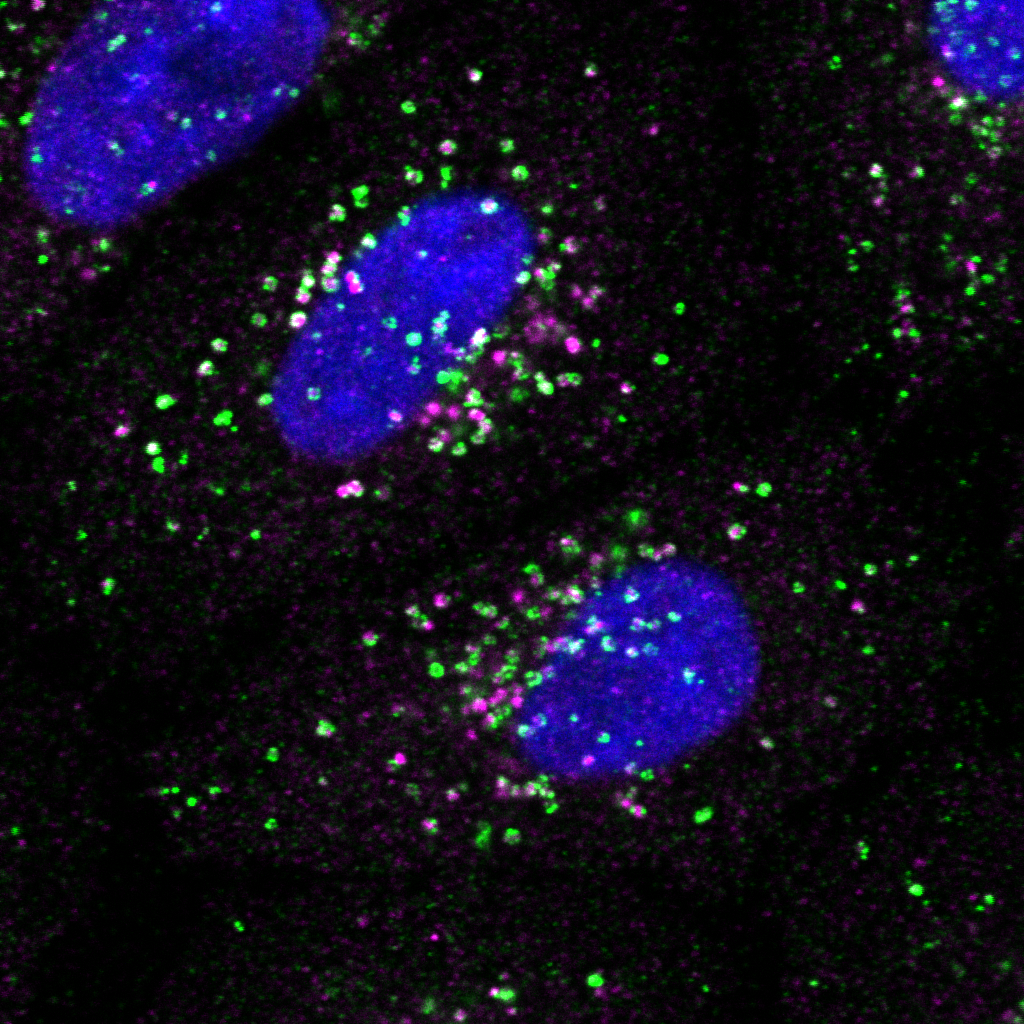

Supplement: Supplementary file 3 — Source data Fig. 1 [file 44318_2025_672_MOESM3_ESM.zip › Figure 1/1A/WT_LLOMe_Merge.tif]

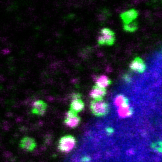

Supplement: Supplementary file 3 — Source data Fig. 1 [file 44318_2025_672_MOESM3_ESM.zip › Figure 1/1A/WT_LLOMe_Merge_zoom.tif]

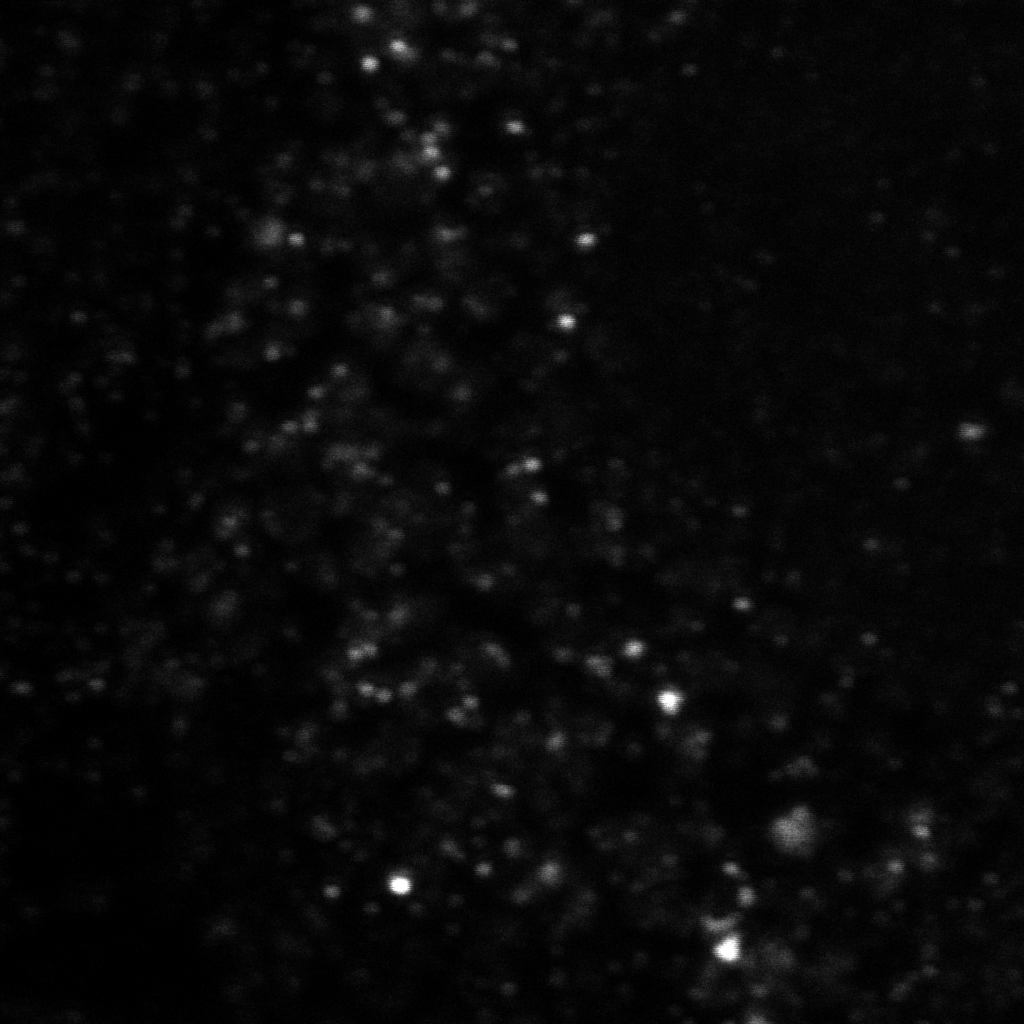

Supplement: Supplementary file 3 — Source data Fig. 1 [file 44318_2025_672_MOESM3_ESM.zip › Figure 1/1D/DKO_ALIX_ALIX.tif]

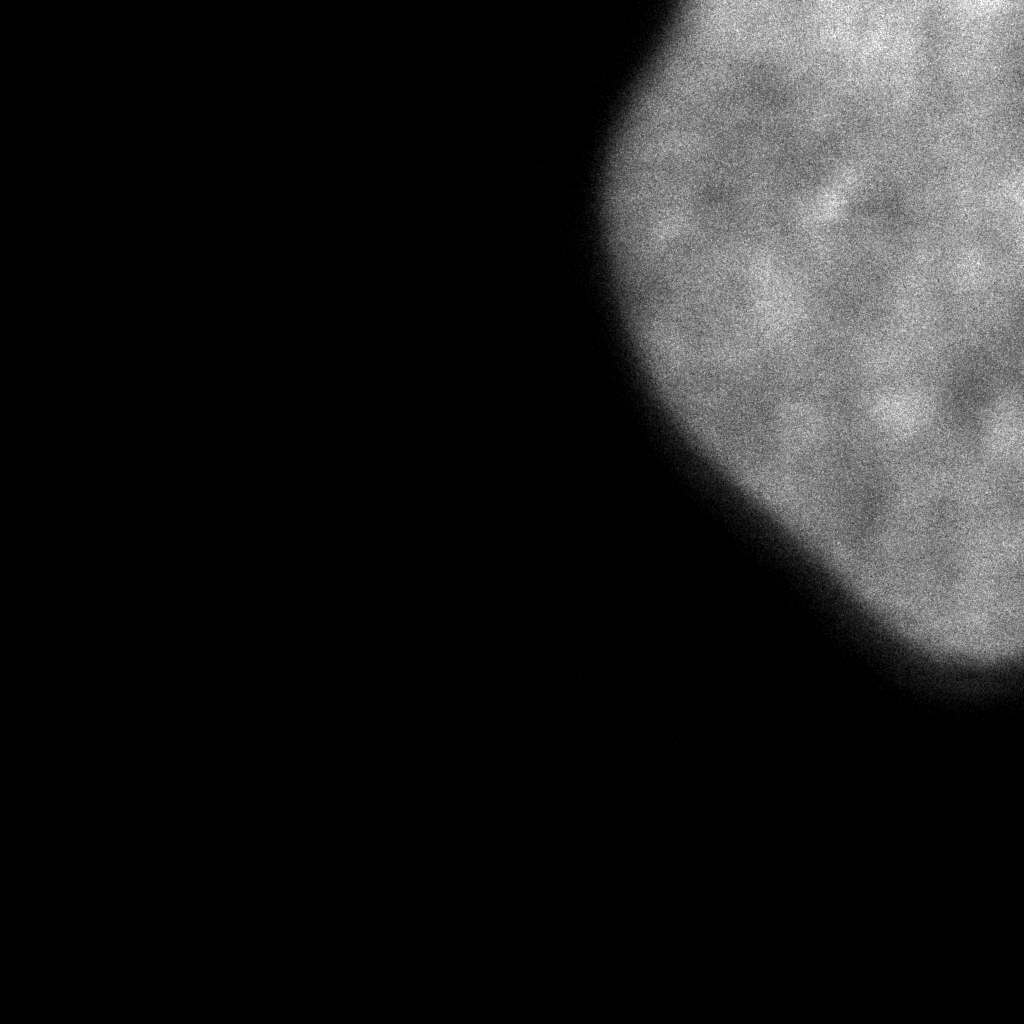

Supplement: Supplementary file 3 — Source data Fig. 1 [file 44318_2025_672_MOESM3_ESM.zip › Figure 1/1D/DKO_ALIX_DAPI.tif]

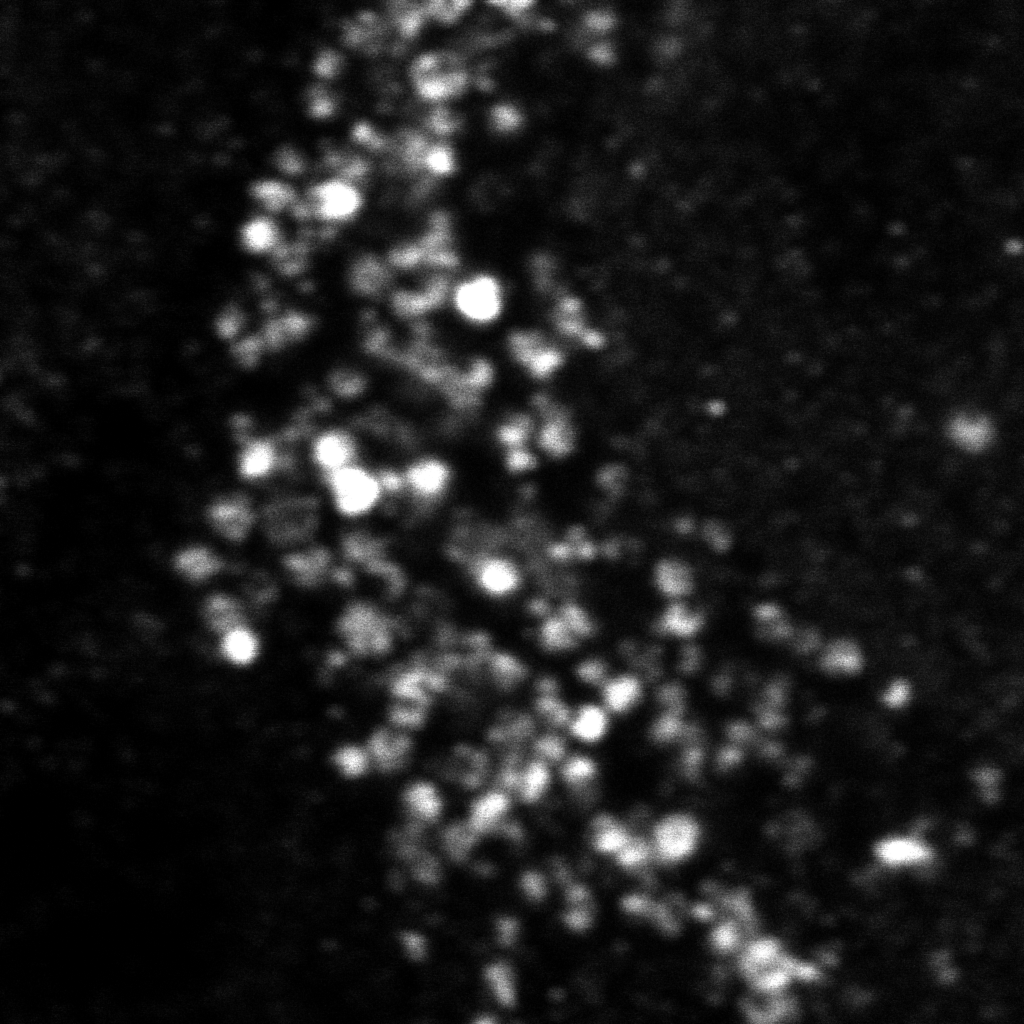

Supplement: Supplementary file 3 — Source data Fig. 1 [file 44318_2025_672_MOESM3_ESM.zip › Figure 1/1D/DKO_ALIX_Gal3.tif]

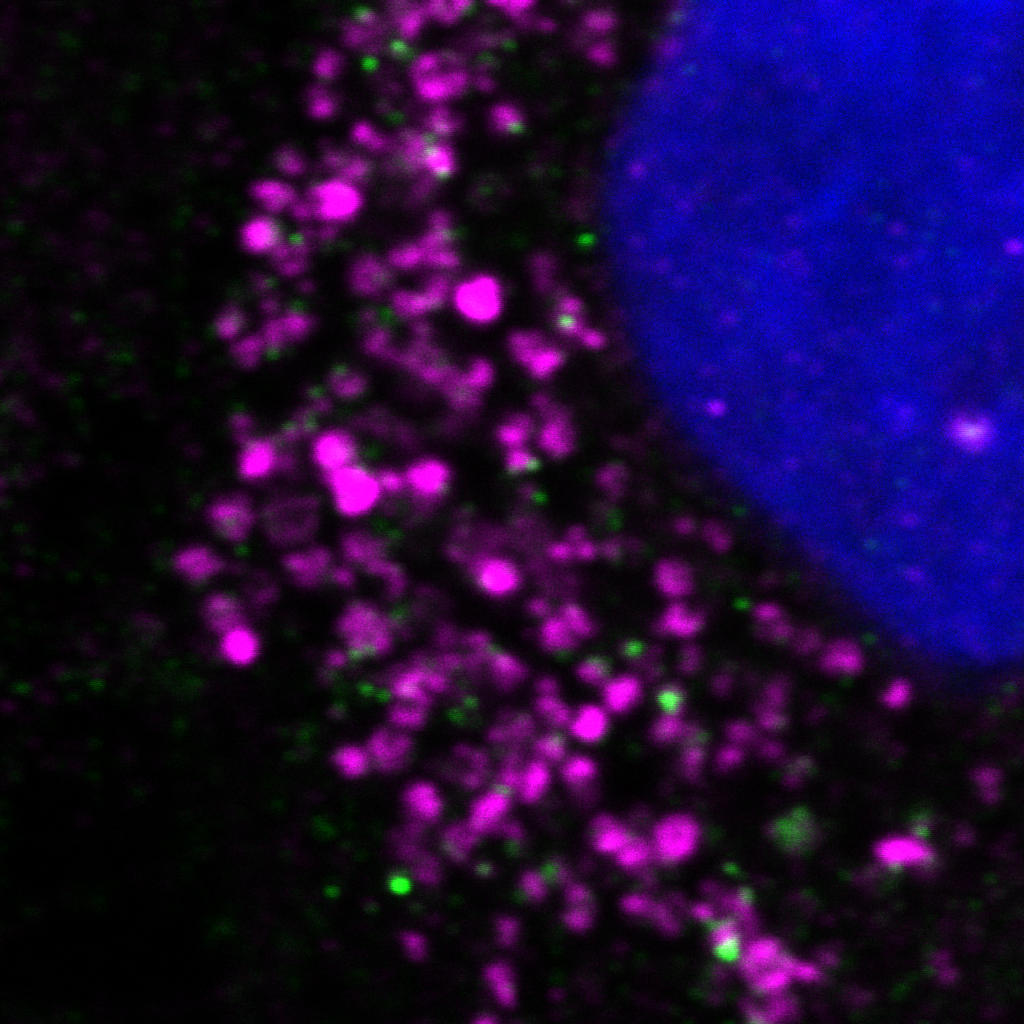

Supplement: Supplementary file 3 — Source data Fig. 1 [file 44318_2025_672_MOESM3_ESM.zip › Figure 1/1D/DKO_ALIX_Merge.tif]

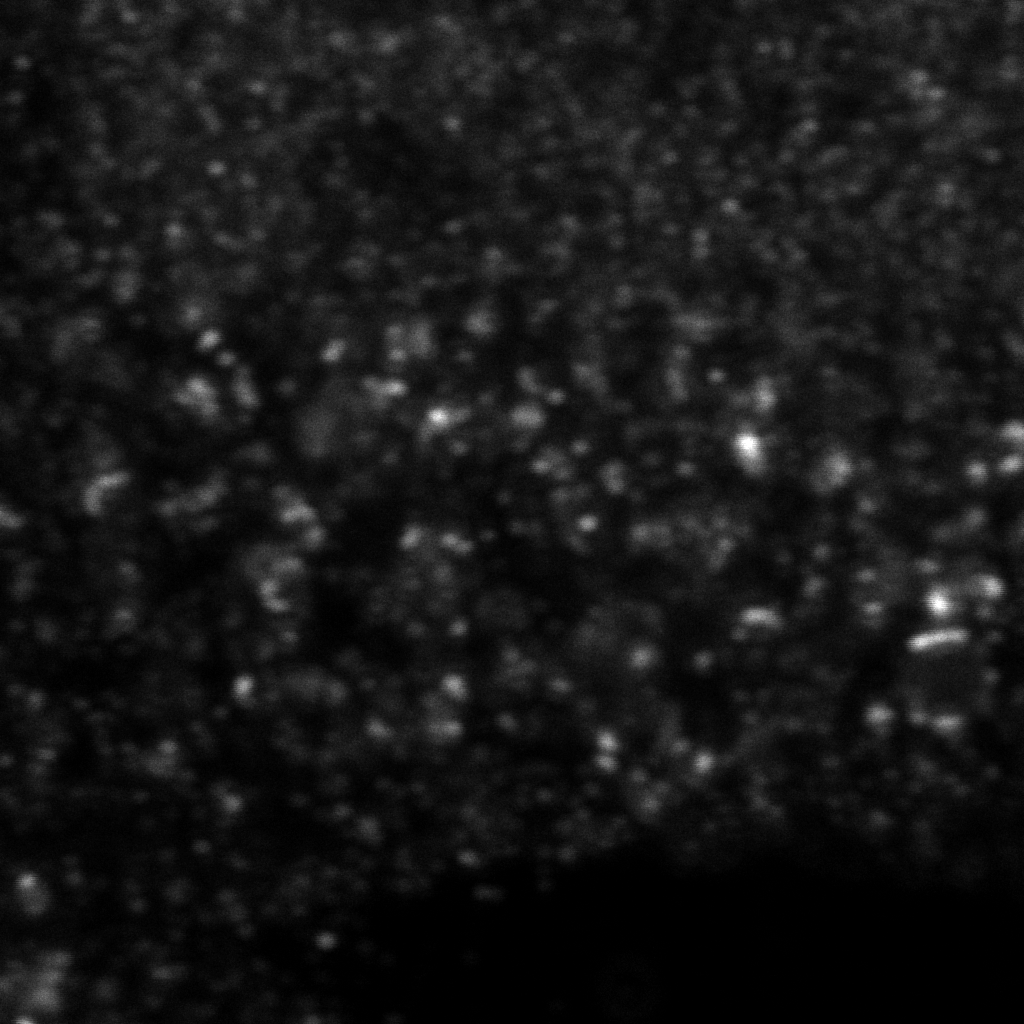

Supplement: Supplementary file 3 — Source data Fig. 1 [file 44318_2025_672_MOESM3_ESM.zip › Figure 1/1D/DKO_CHMP2A_CHMP2A.tif]

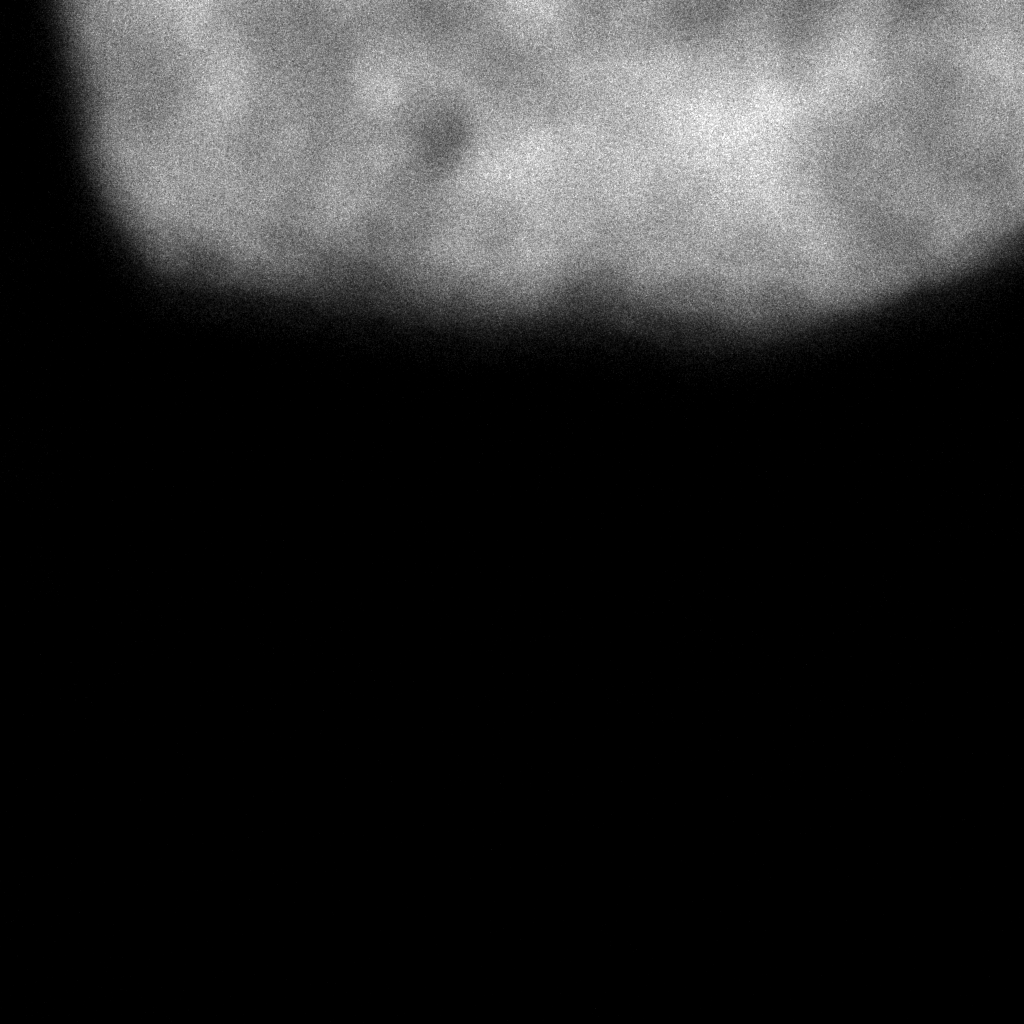

Supplement: Supplementary file 3 — Source data Fig. 1 [file 44318_2025_672_MOESM3_ESM.zip › Figure 1/1D/DKO_CHMP2A_DAPI.tif]

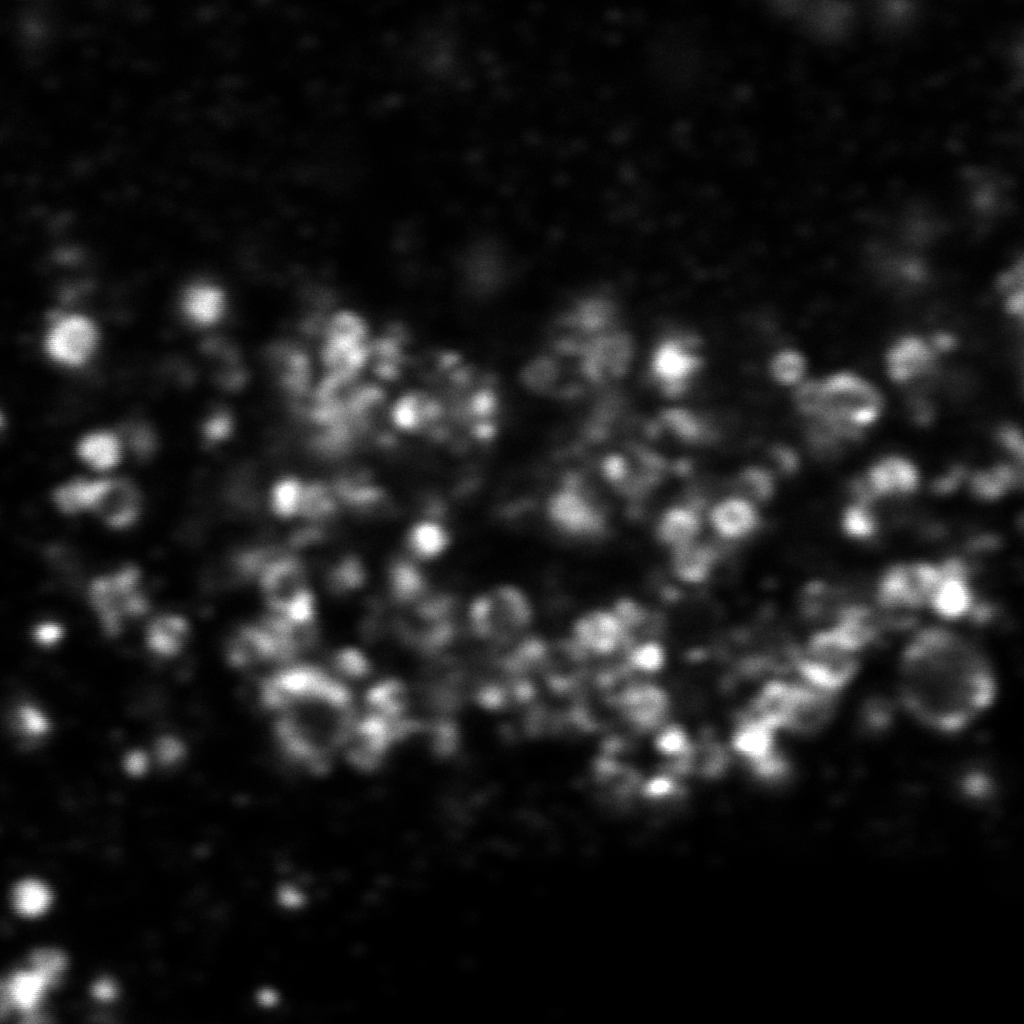

Supplement: Supplementary file 3 — Source data Fig. 1 [file 44318_2025_672_MOESM3_ESM.zip › Figure 1/1D/DKO_CHMP2A_Gal3.tif]

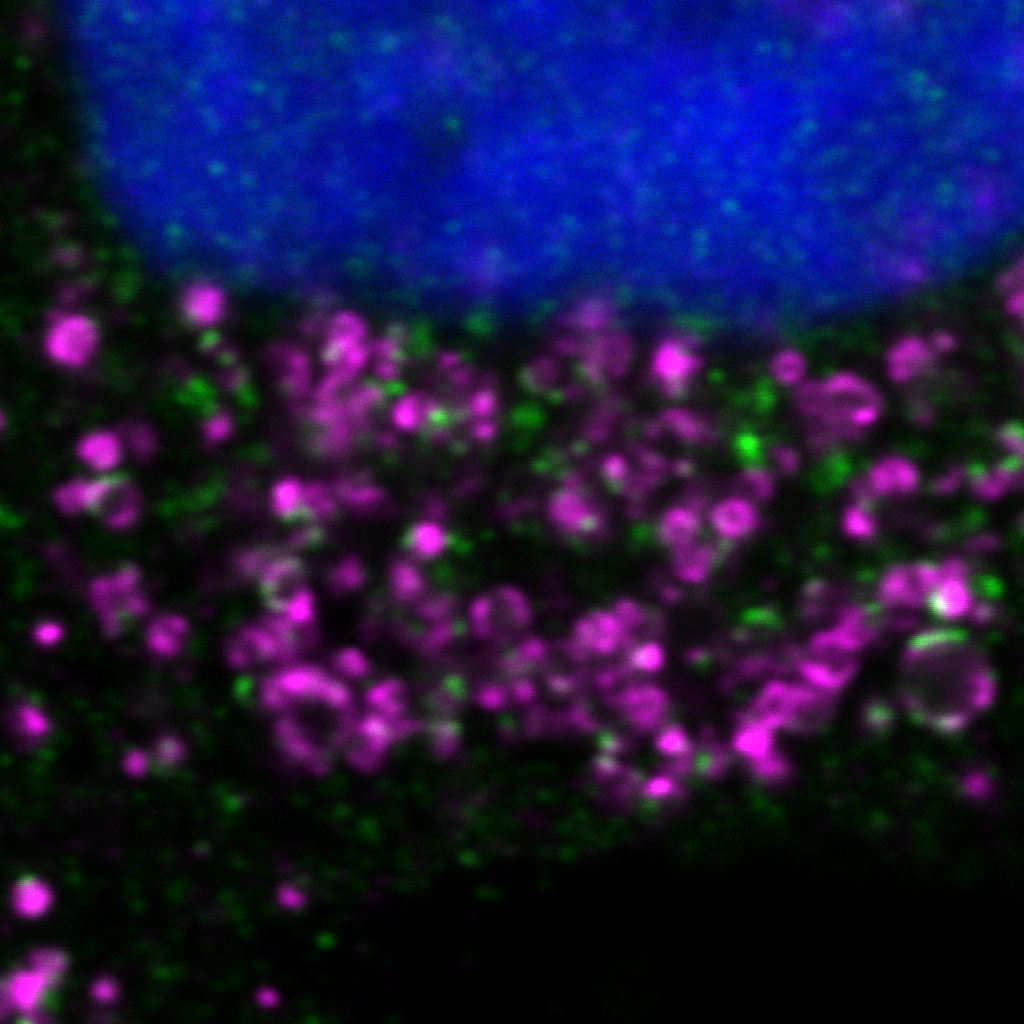

Supplement: Supplementary file 3 — Source data Fig. 1 [file 44318_2025_672_MOESM3_ESM.zip › Figure 1/1D/DKO_CHMP2A_merge.tif]

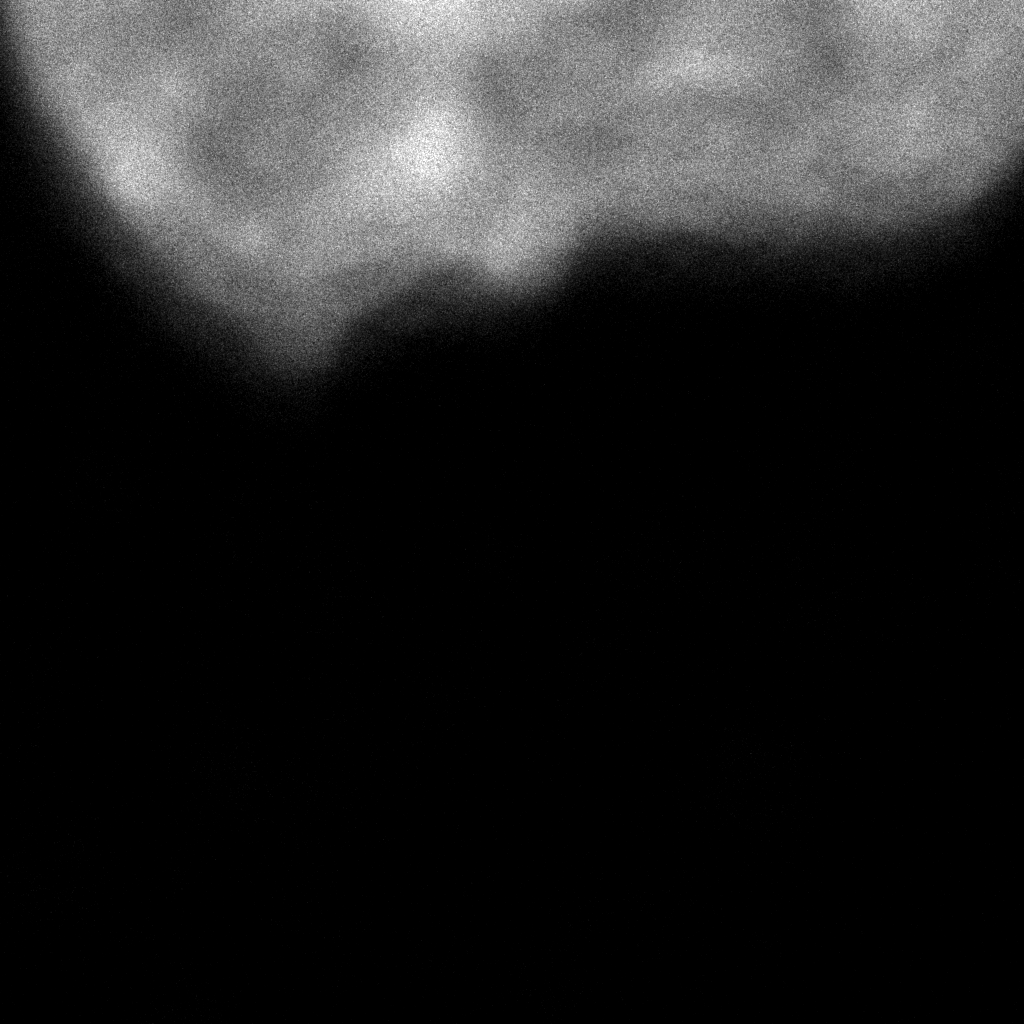

Supplement: Supplementary file 3 — Source data Fig. 1 [file 44318_2025_672_MOESM3_ESM.zip › Figure 1/1D/DKO_IST1_DAPI.tif]

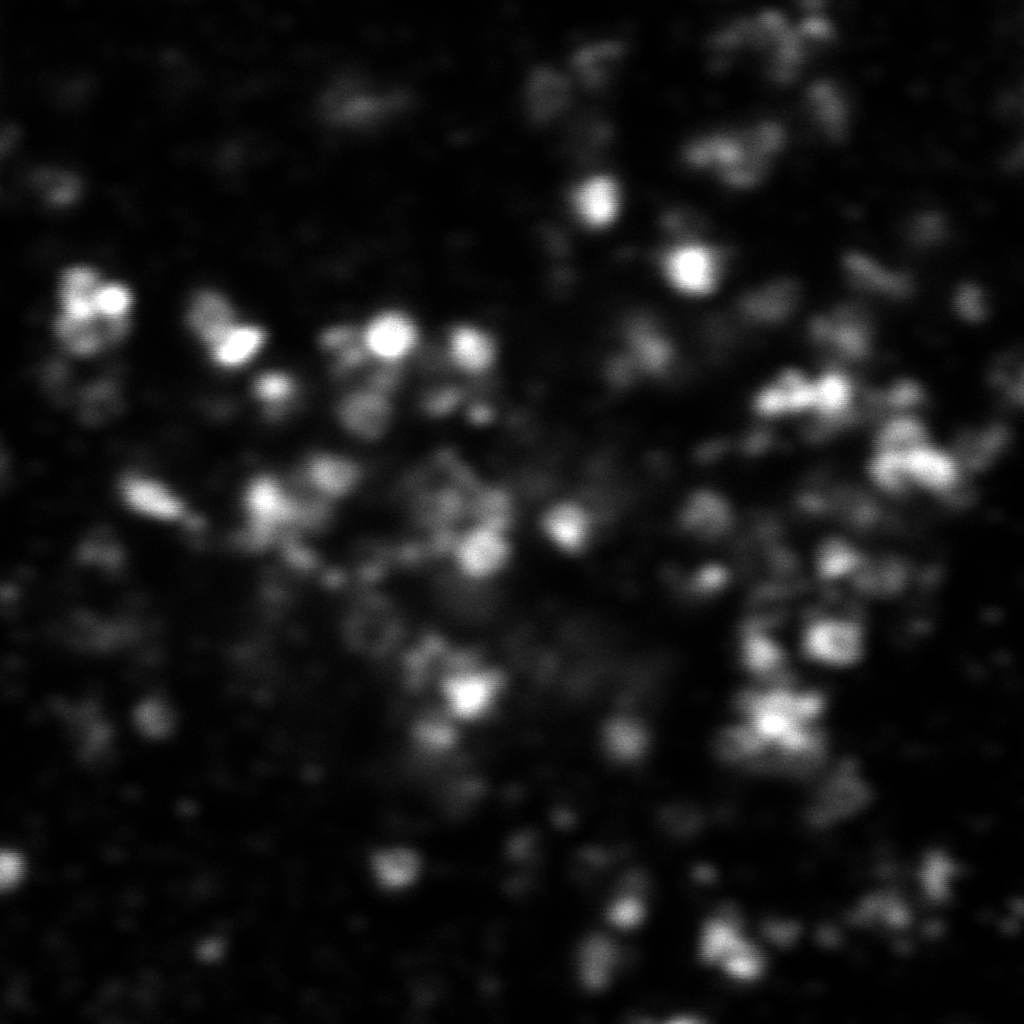

Supplement: Supplementary file 3 — Source data Fig. 1 [file 44318_2025_672_MOESM3_ESM.zip › Figure 1/1D/DKO_IST1_Gal3.tif]

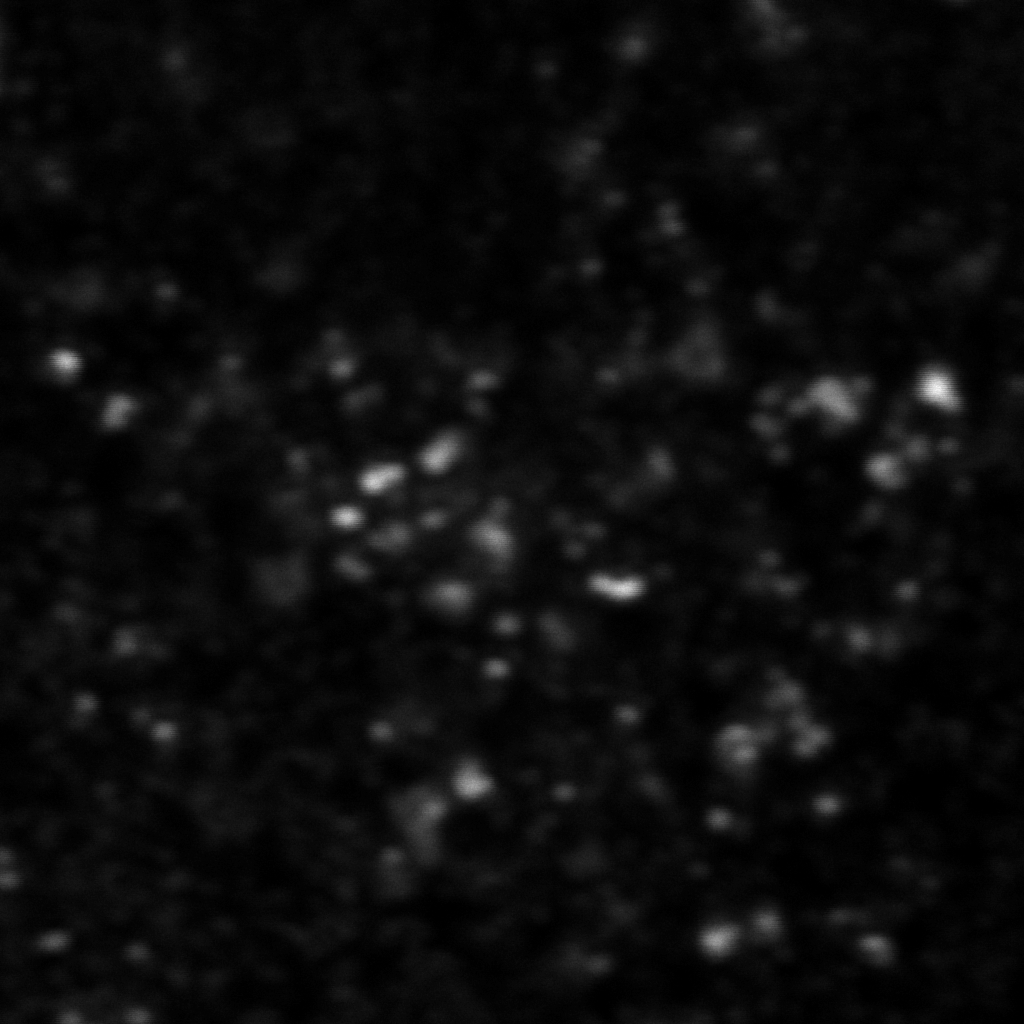

Supplement: Supplementary file 3 — Source data Fig. 1 [file 44318_2025_672_MOESM3_ESM.zip › Figure 1/1D/DKO_IST1_IST1.tif]

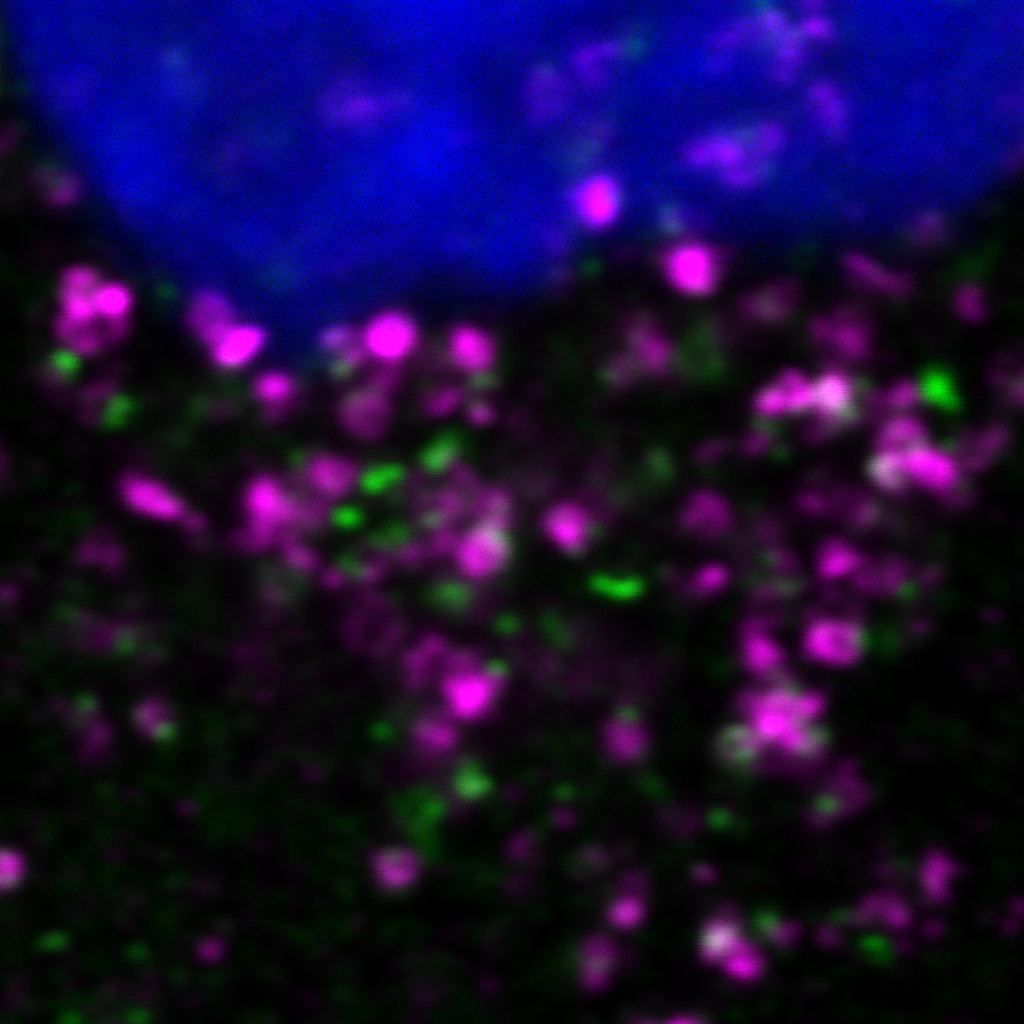

Supplement: Supplementary file 3 — Source data Fig. 1 [file 44318_2025_672_MOESM3_ESM.zip › Figure 1/1D/DKO_IST1_merge.tif]

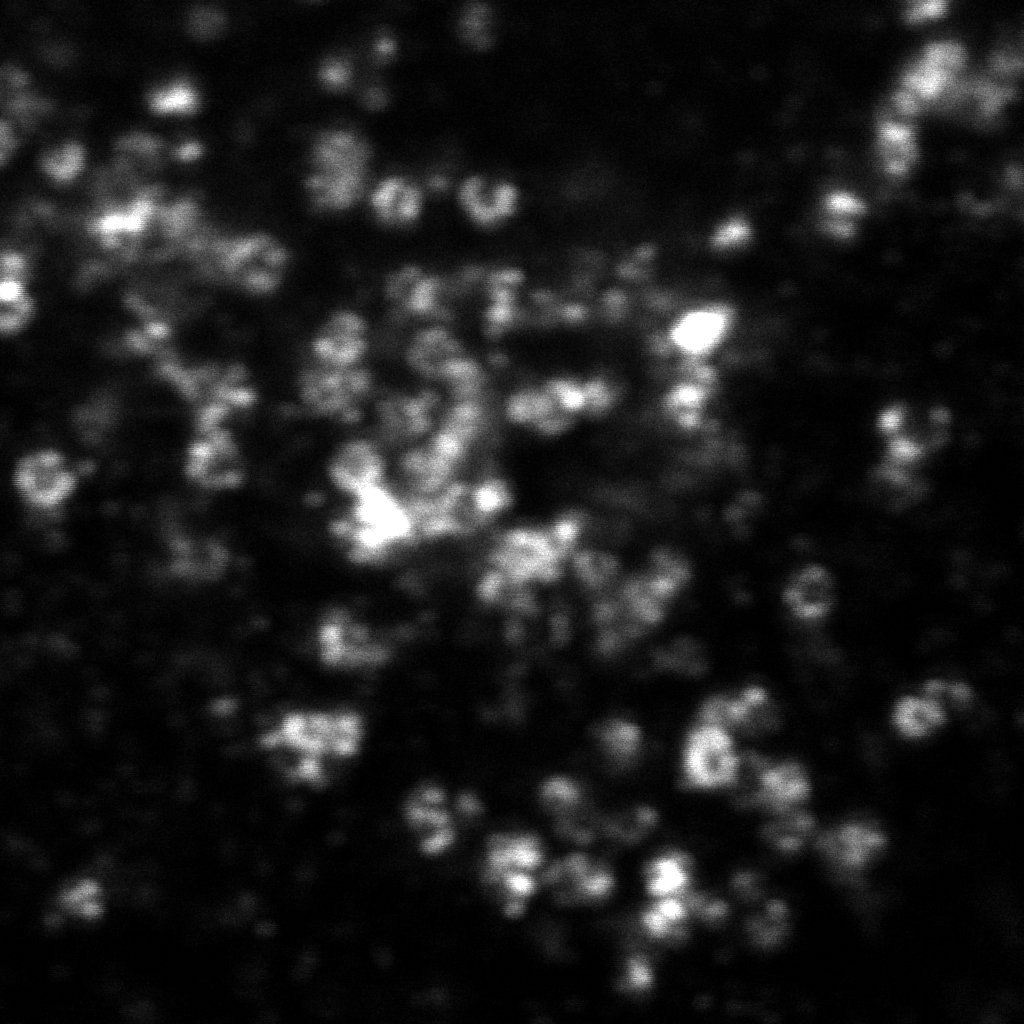

Supplement: Supplementary file 3 — Source data Fig. 1 [file 44318_2025_672_MOESM3_ESM.zip › Figure 1/1D/WT_ALIX_ALIX.tif]

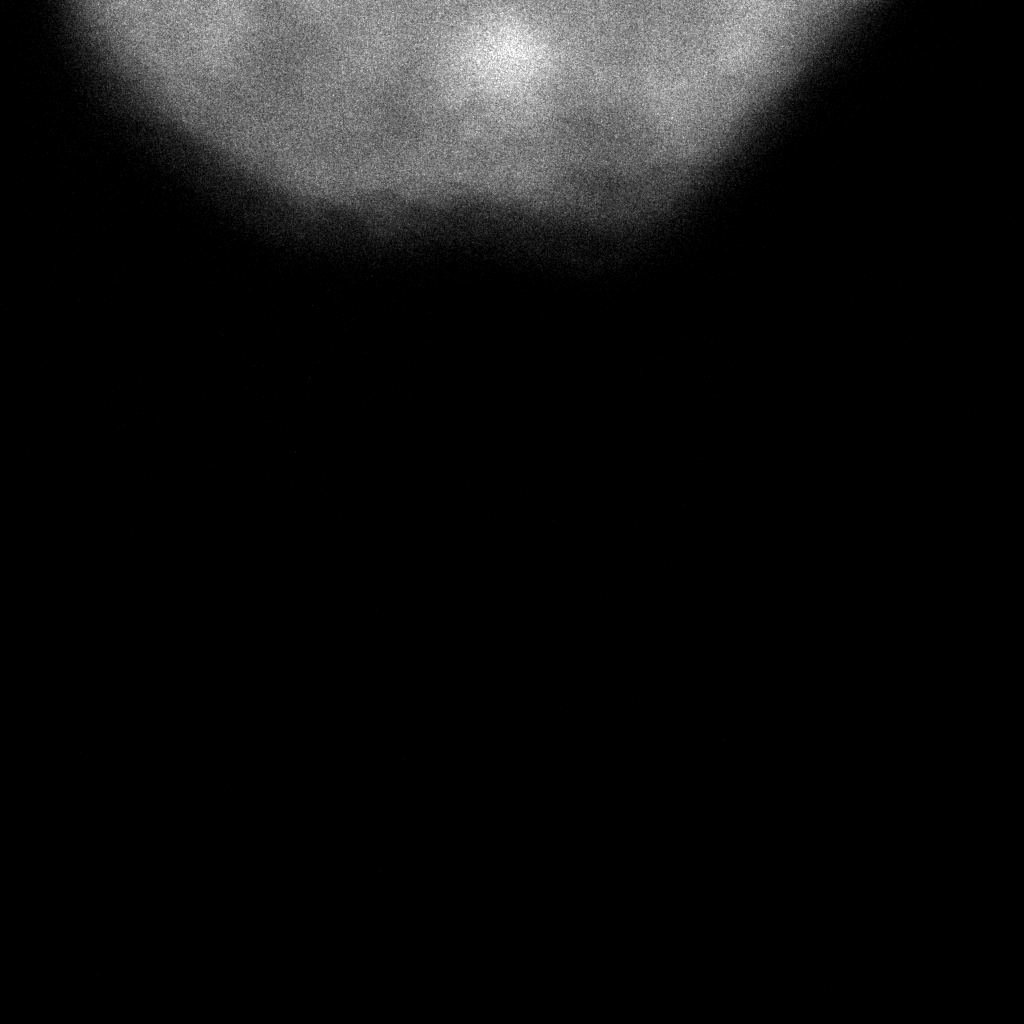

Supplement: Supplementary file 3 — Source data Fig. 1 [file 44318_2025_672_MOESM3_ESM.zip › Figure 1/1D/WT_ALIX_DAPI.tif]

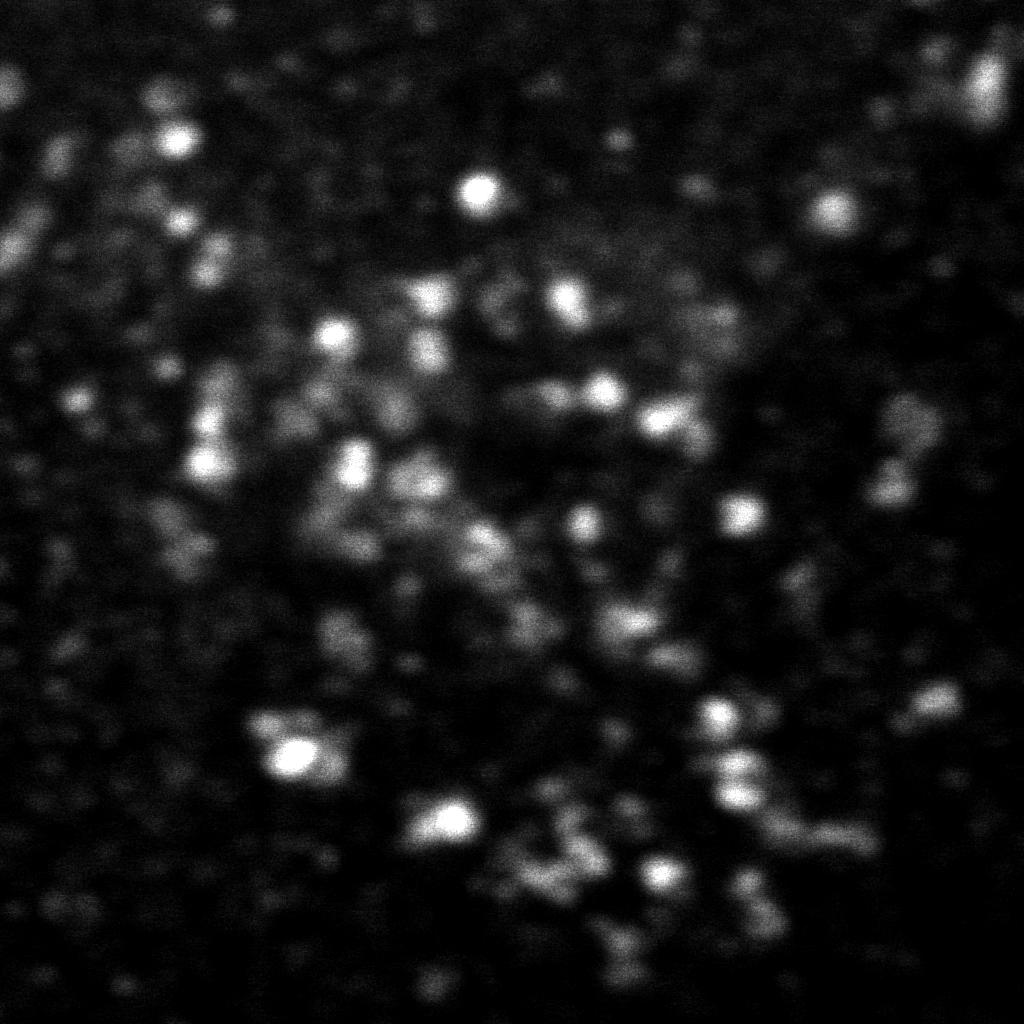

Supplement: Supplementary file 3 — Source data Fig. 1 [file 44318_2025_672_MOESM3_ESM.zip › Figure 1/1D/WT_ALIX_Gal3.tif]

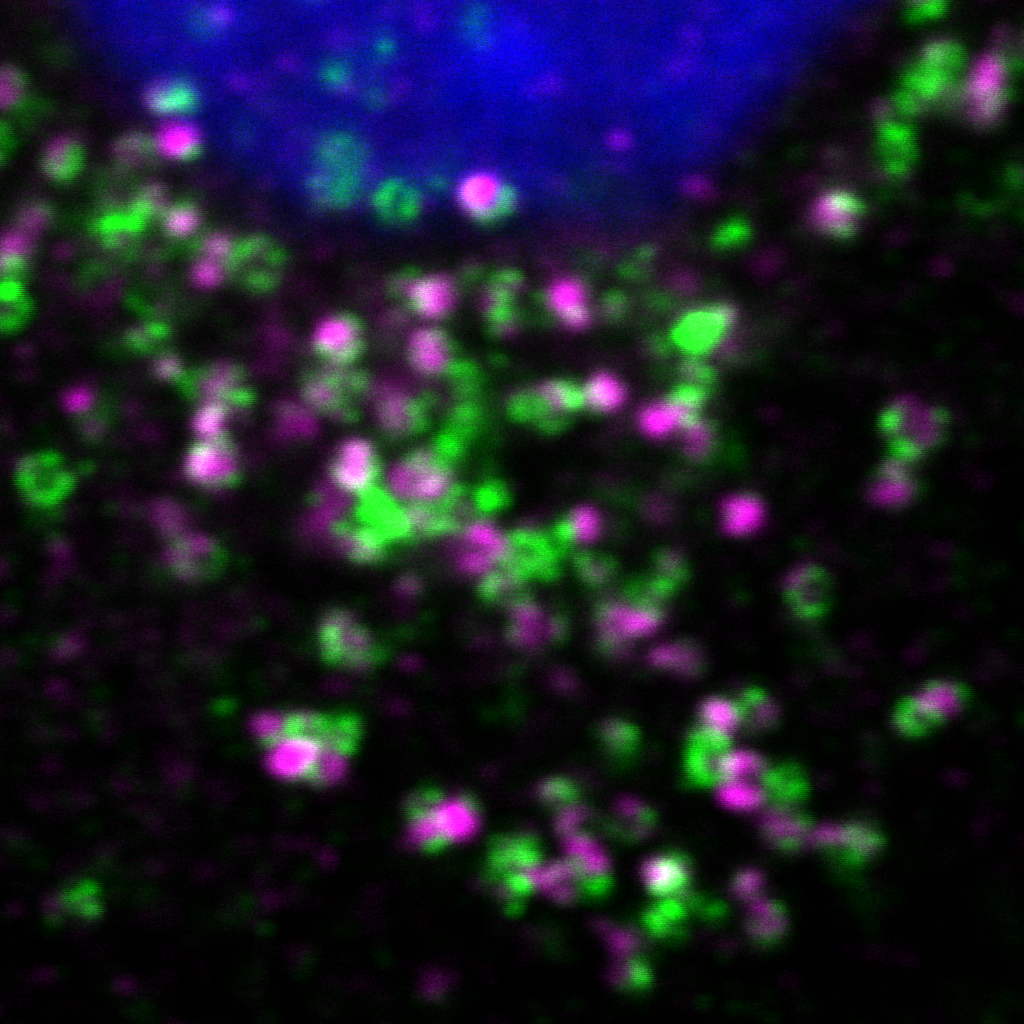

Supplement: Supplementary file 3 — Source data Fig. 1 [file 44318_2025_672_MOESM3_ESM.zip › Figure 1/1D/WT_ALIX_Merge.tif]

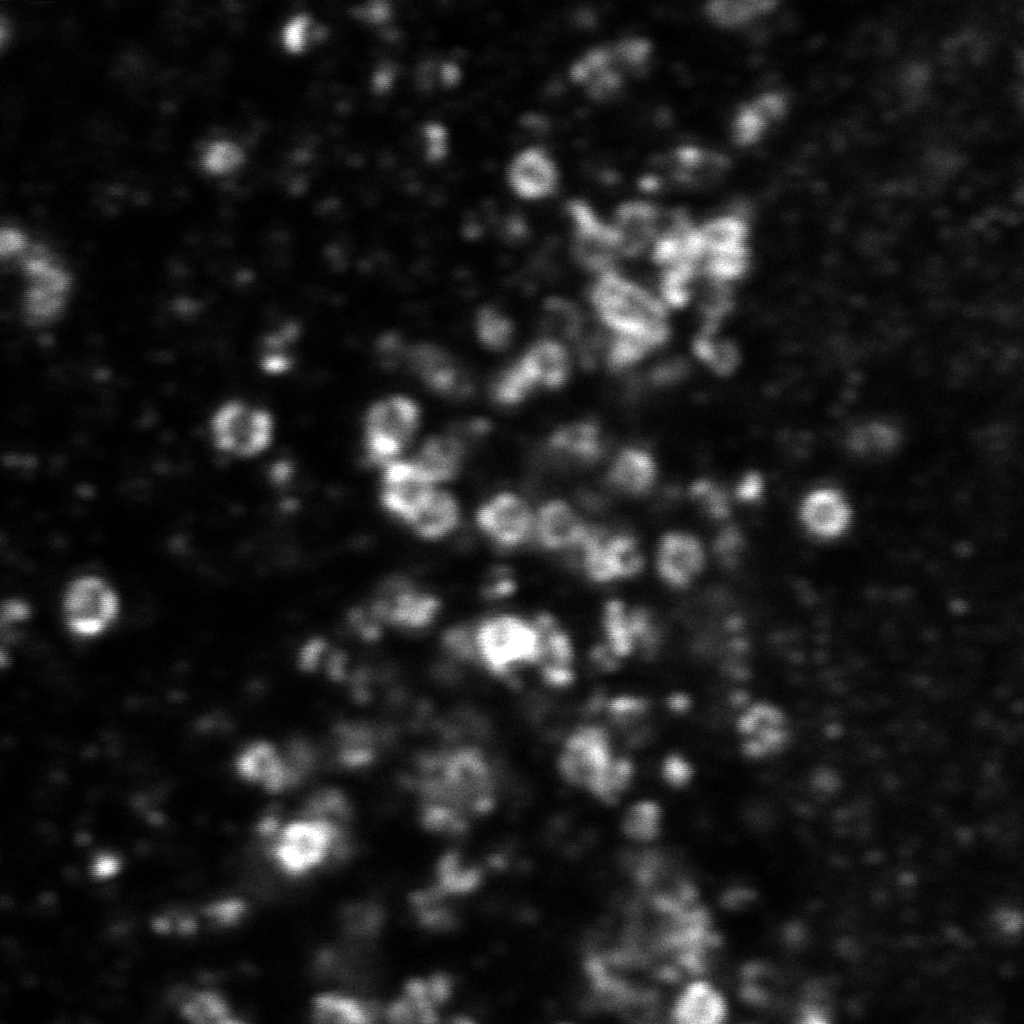

Supplement: Supplementary file 3 — Source data Fig. 1 [file 44318_2025_672_MOESM3_ESM.zip › Figure 1/1D/WT_CHMP2A_CHMP2A.tif]

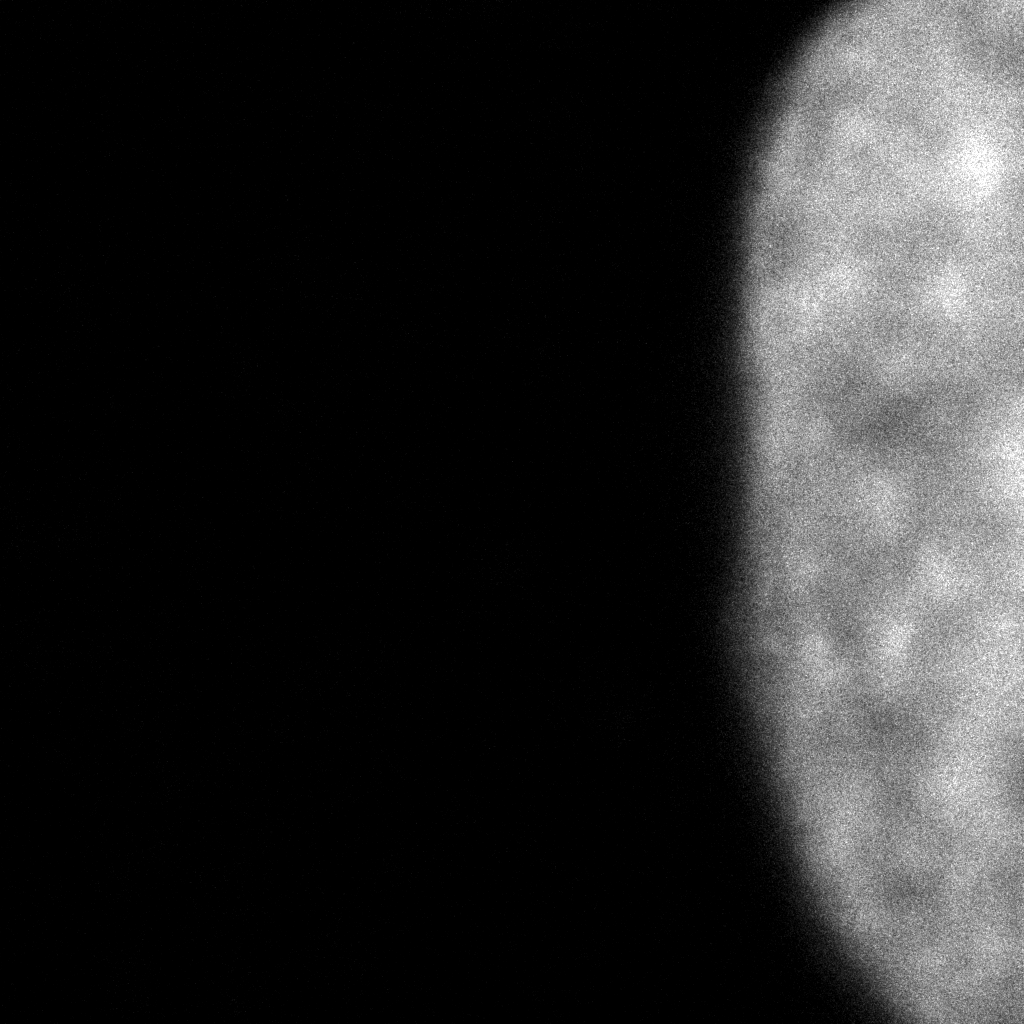

Supplement: Supplementary file 3 — Source data Fig. 1 [file 44318_2025_672_MOESM3_ESM.zip › Figure 1/1D/WT_CHMP2A_DAPI.tif]

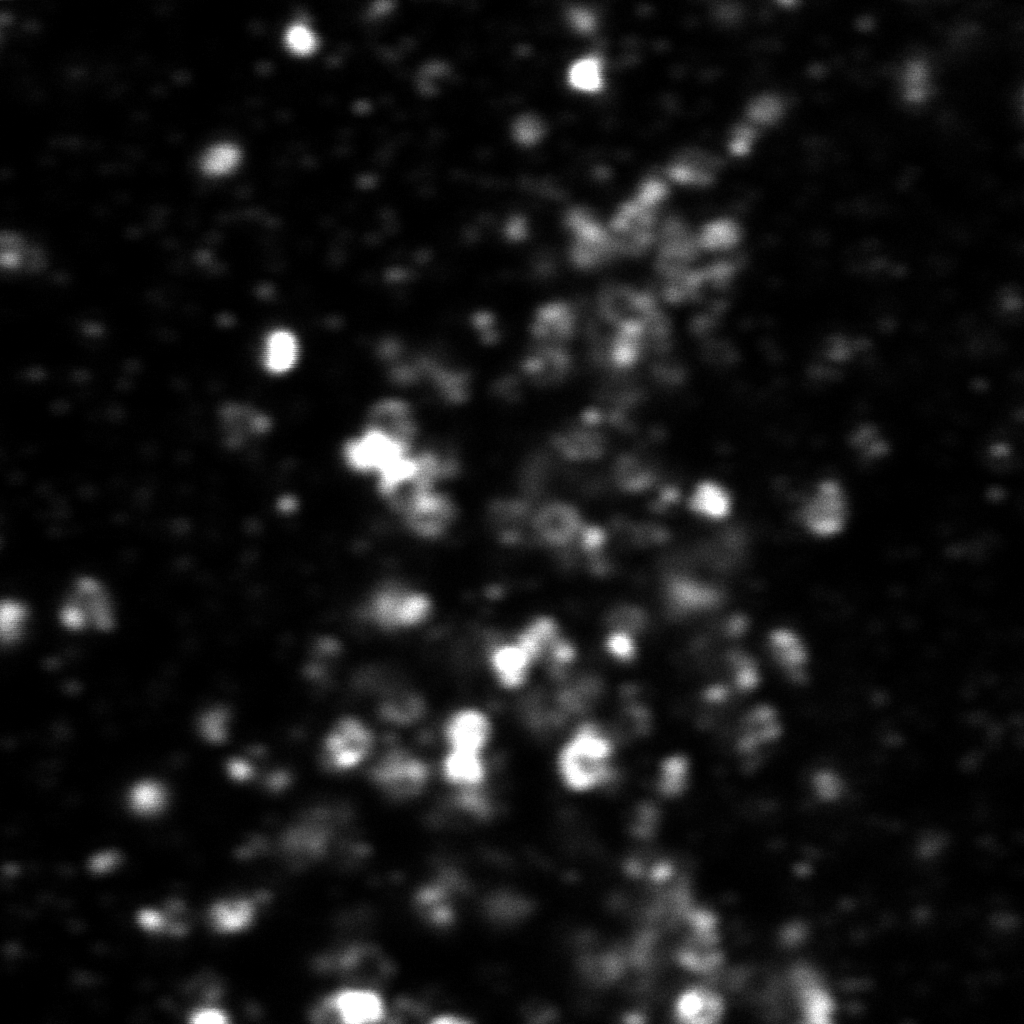

Supplement: Supplementary file 3 — Source data Fig. 1 [file 44318_2025_672_MOESM3_ESM.zip › Figure 1/1D/WT_CHMP2A_Gal3.tif]

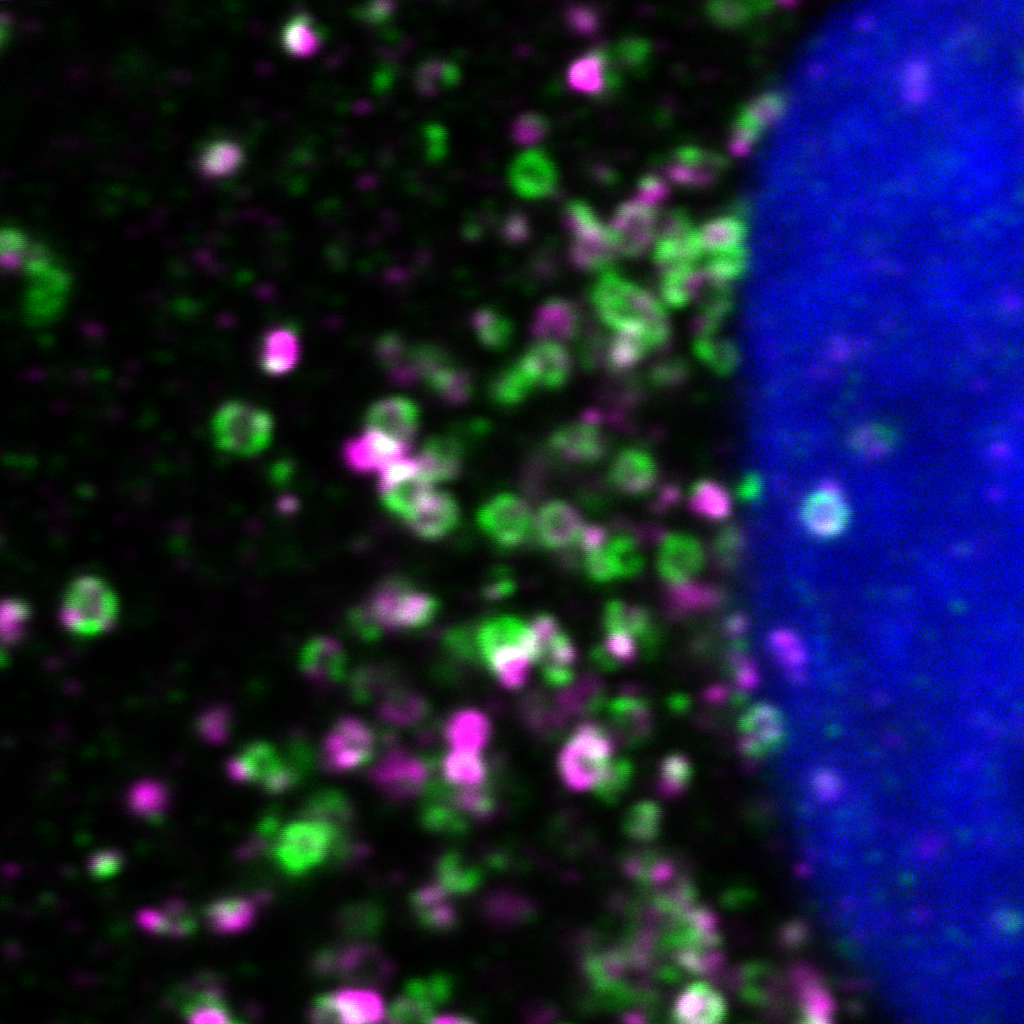

Supplement: Supplementary file 3 — Source data Fig. 1 [file 44318_2025_672_MOESM3_ESM.zip › Figure 1/1D/WT_CHMP2A_merge.tif]

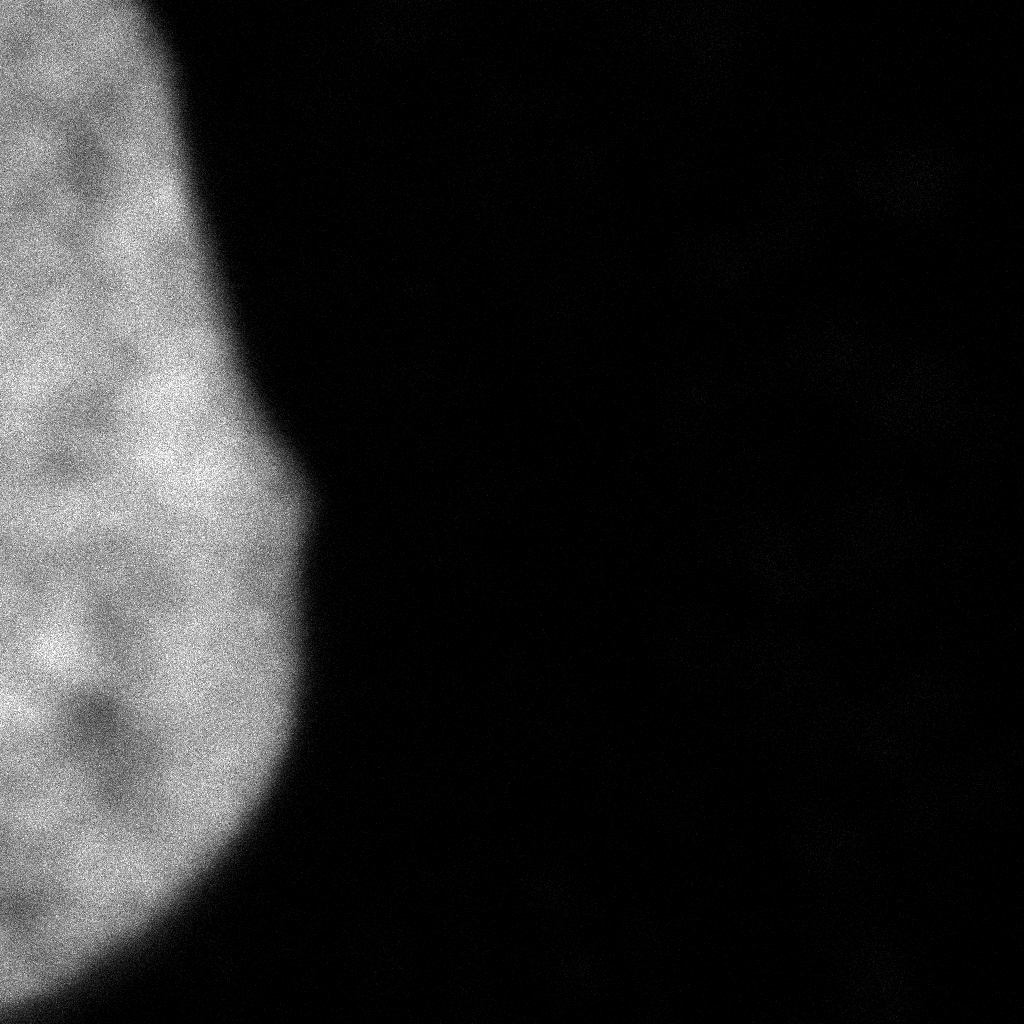

Supplement: Supplementary file 3 — Source data Fig. 1 [file 44318_2025_672_MOESM3_ESM.zip › Figure 1/1D/WT_IST1_DAPI.tif]

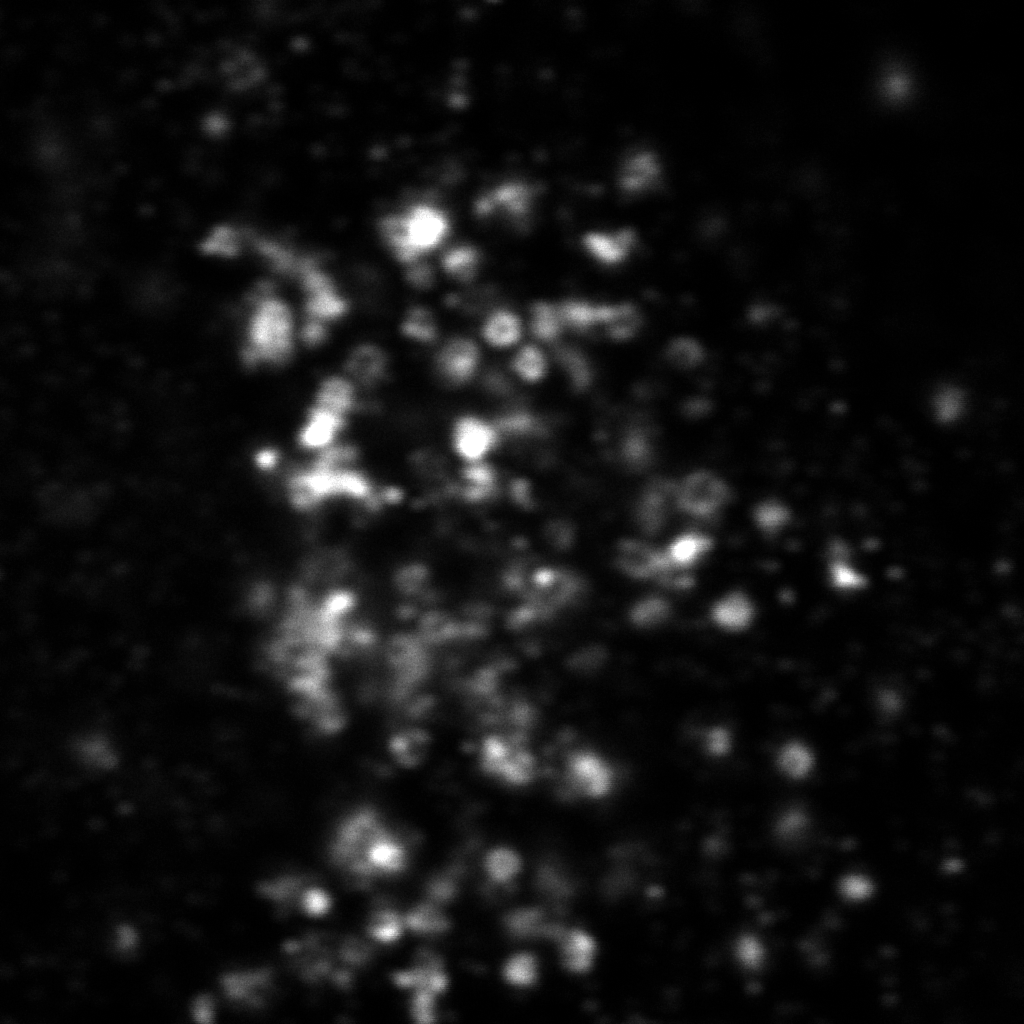

Supplement: Supplementary file 3 — Source data Fig. 1 [file 44318_2025_672_MOESM3_ESM.zip › Figure 1/1D/WT_IST1_Gal3.tif]

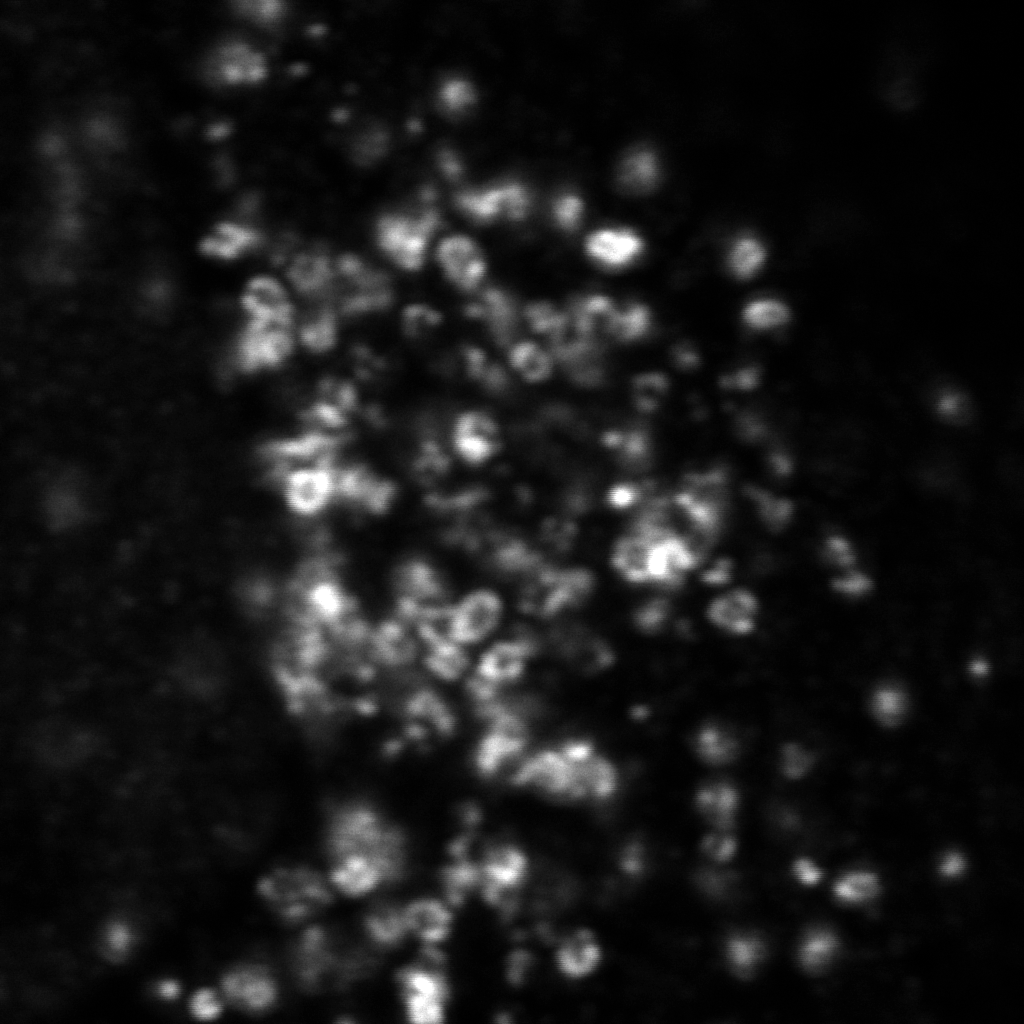

Supplement: Supplementary file 3 — Source data Fig. 1 [file 44318_2025_672_MOESM3_ESM.zip › Figure 1/1D/WT_IST1_IST1.tif]

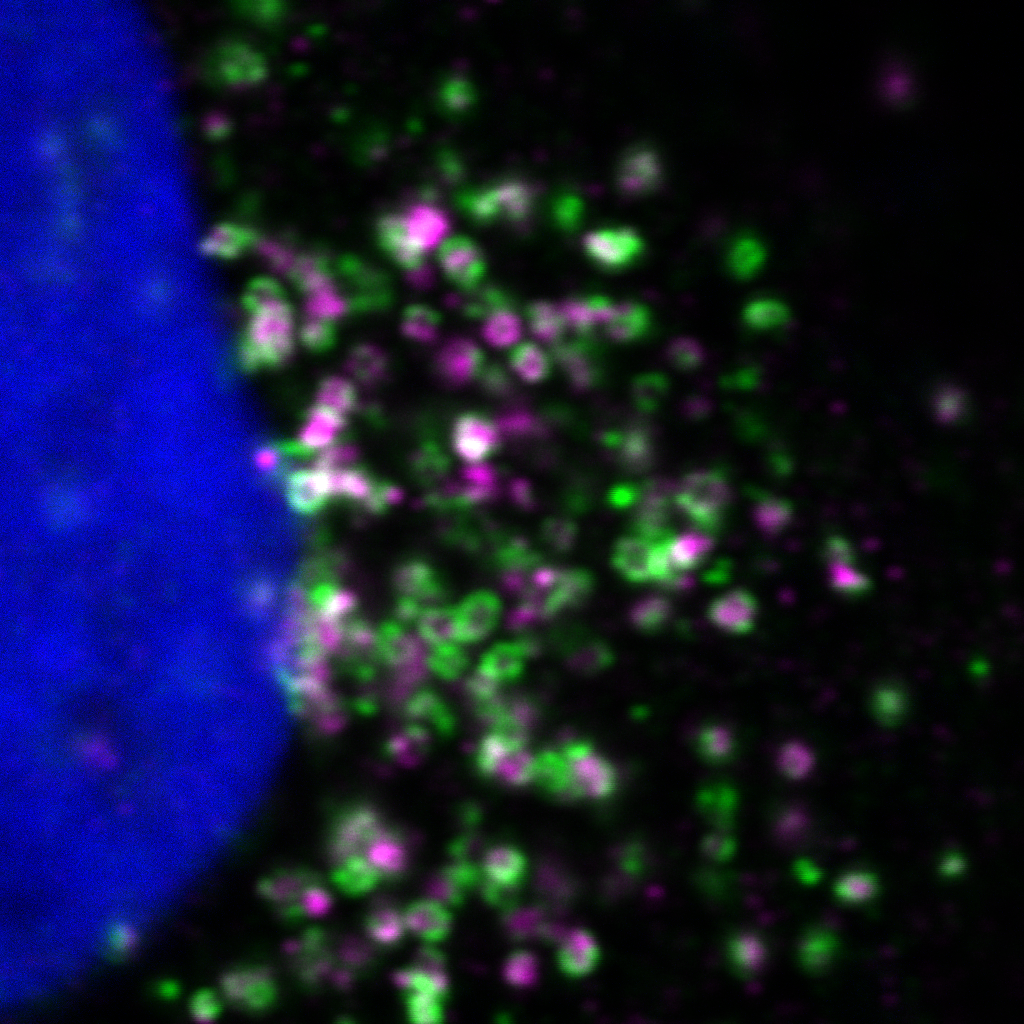

Supplement: Supplementary file 3 — Source data Fig. 1 [file 44318_2025_672_MOESM3_ESM.zip › Figure 1/1D/WT_IST1_merge.tif]

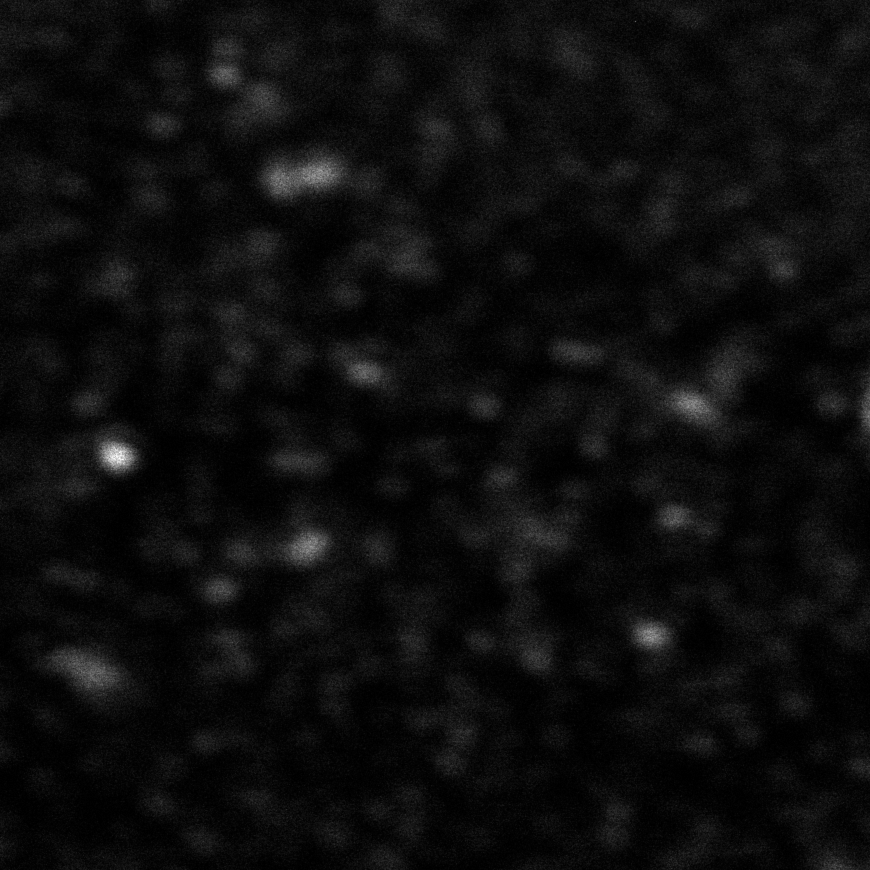

Supplement: Supplementary file 3 — Source data Fig. 1 [file 44318_2025_672_MOESM3_ESM.zip › Figure 1/1F/d1-377_ALIX.tif]

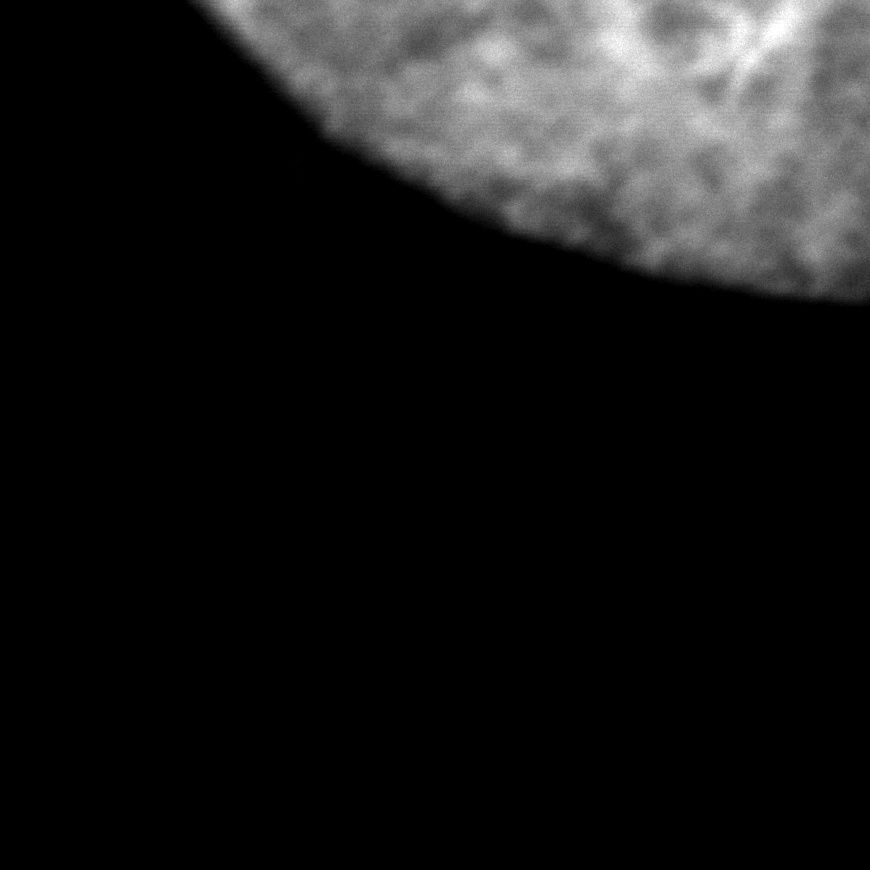

Supplement: Supplementary file 3 — Source data Fig. 1 [file 44318_2025_672_MOESM3_ESM.zip › Figure 1/1F/d1-377_DAPI.tif]

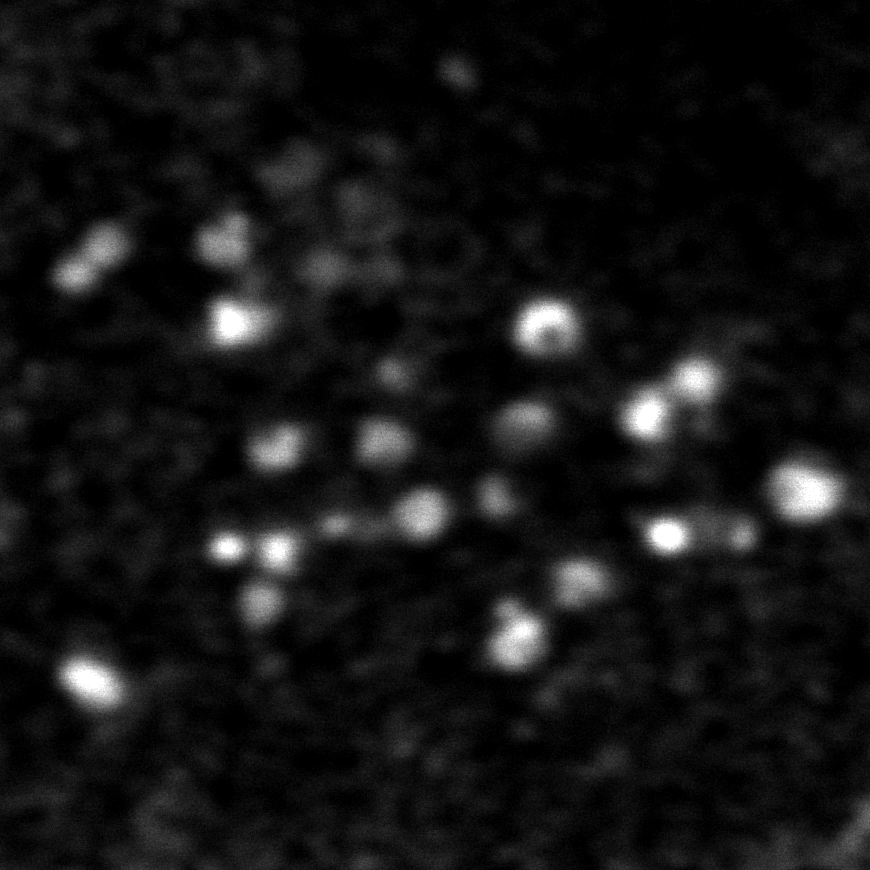

Supplement: Supplementary file 3 — Source data Fig. 1 [file 44318_2025_672_MOESM3_ESM.zip › Figure 1/1F/d1-377_LAMP.tif]

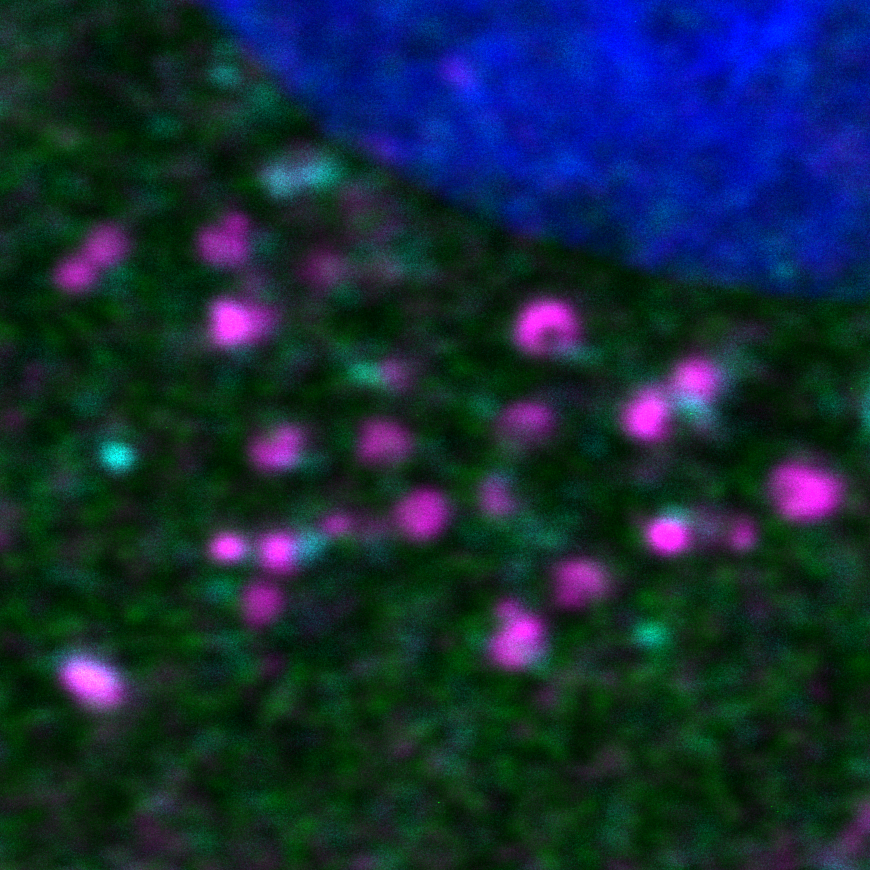

Supplement: Supplementary file 3 — Source data Fig. 1 [file 44318_2025_672_MOESM3_ESM.zip › Figure 1/1F/d1-377_merge.tif]

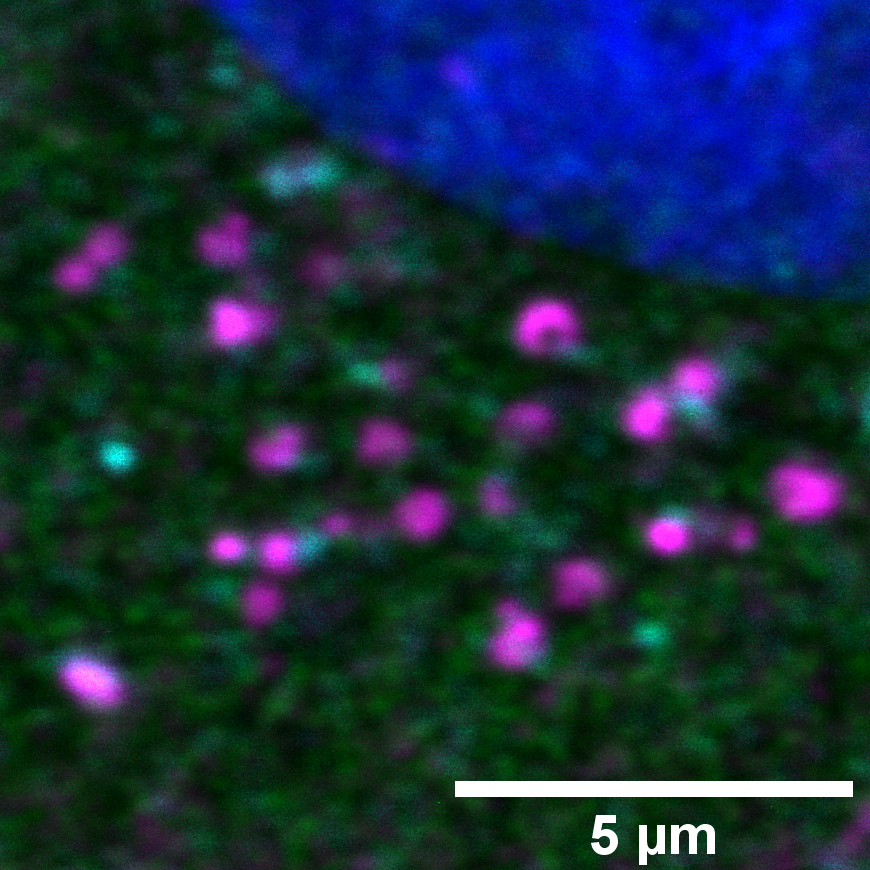

Supplement: Supplementary file 3 — Source data Fig. 1 [file 44318_2025_672_MOESM3_ESM.zip › Figure 1/1F/d1-377_scale.tif]

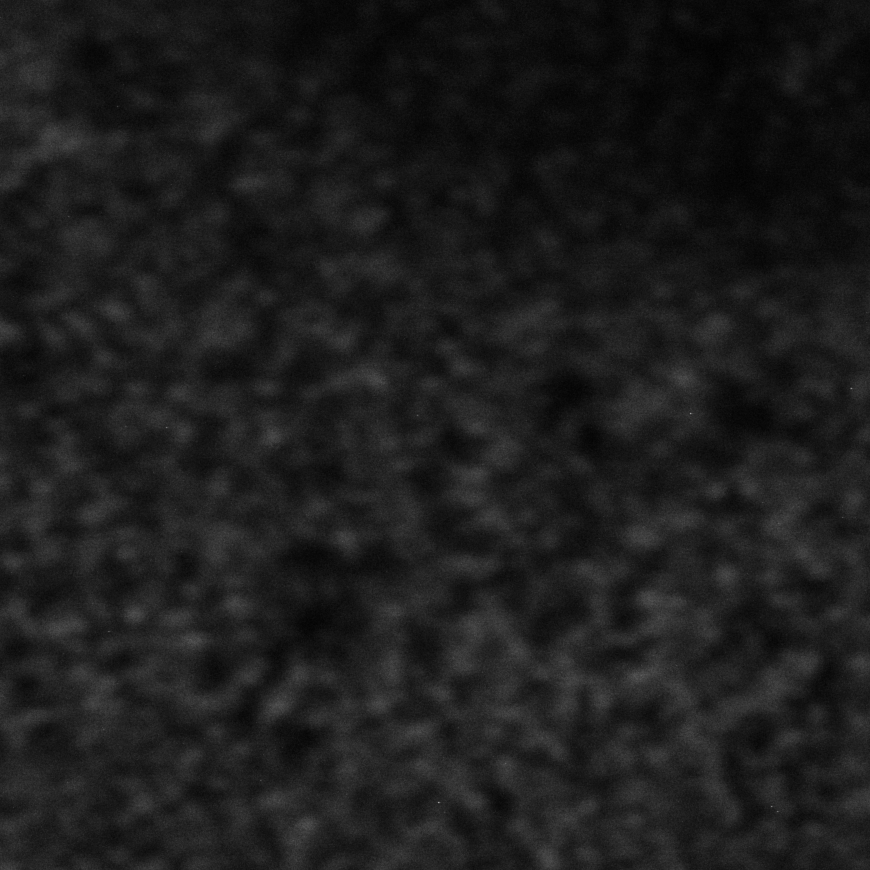

Supplement: Supplementary file 3 — Source data Fig. 1 [file 44318_2025_672_MOESM3_ESM.zip › Figure 1/1F/d1-377_TECPR1.tif]

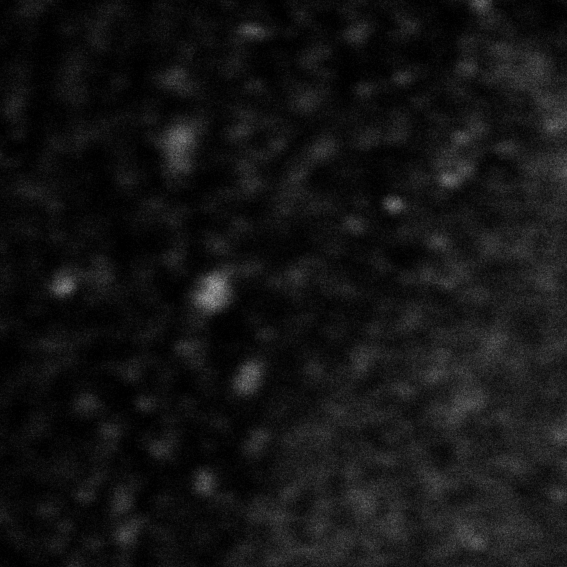

Supplement: Supplementary file 3 — Source data Fig. 1 [file 44318_2025_672_MOESM3_ESM.zip › Figure 1/1F/dAIR_ALIX.tif]

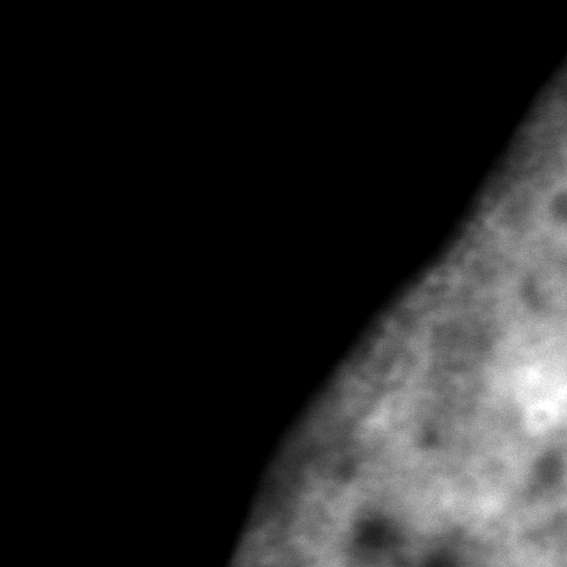

Supplement: Supplementary file 3 — Source data Fig. 1 [file 44318_2025_672_MOESM3_ESM.zip › Figure 1/1F/dAIR_DAPI.tif]

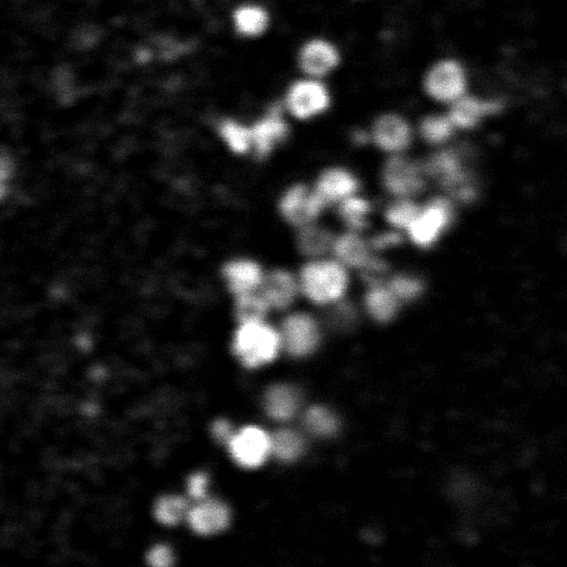

Supplement: Supplementary file 3 — Source data Fig. 1 [file 44318_2025_672_MOESM3_ESM.zip › Figure 1/1F/dAIR_LAMP.tif]

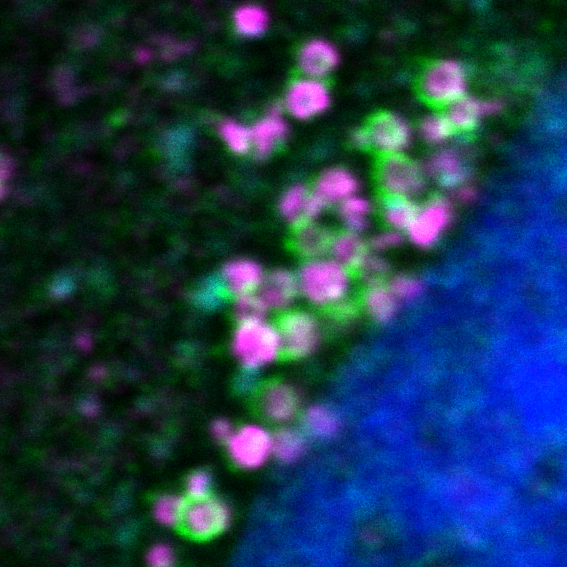

Supplement: Supplementary file 3 — Source data Fig. 1 [file 44318_2025_672_MOESM3_ESM.zip › Figure 1/1F/dAIR_merge.tif]

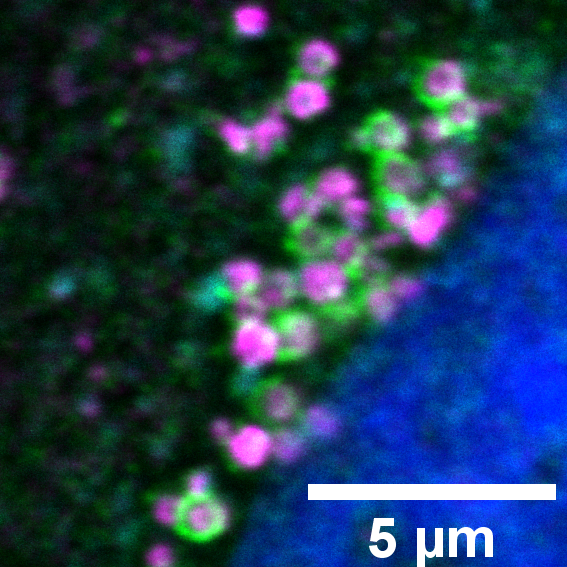

Supplement: Supplementary file 3 — Source data Fig. 1 [file 44318_2025_672_MOESM3_ESM.zip › Figure 1/1F/dAIR_scale.tif]

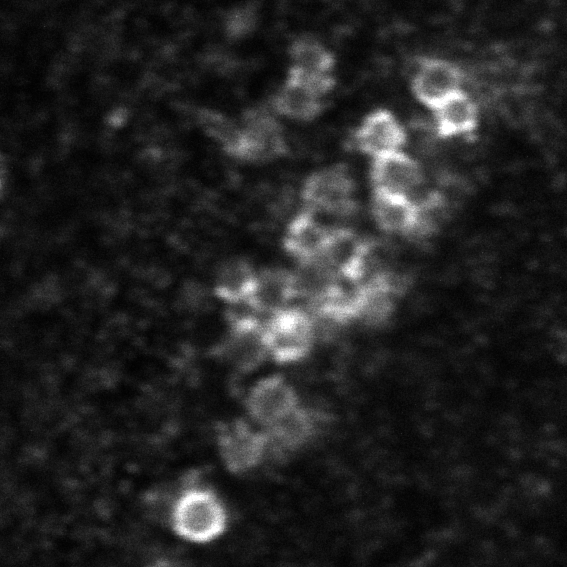

Supplement: Supplementary file 3 — Source data Fig. 1 [file 44318_2025_672_MOESM3_ESM.zip › Figure 1/1F/dAIR_TECPR1.tif]

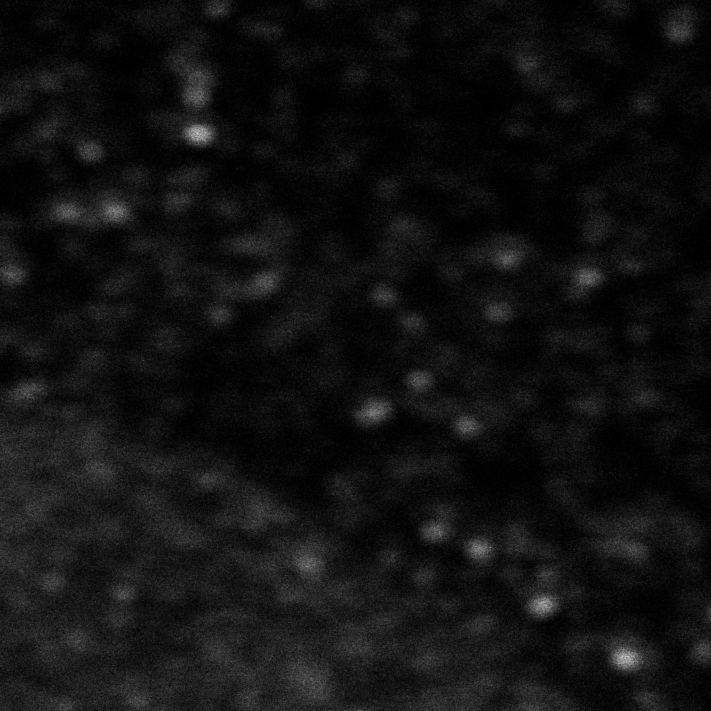

Supplement: Supplementary file 3 — Source data Fig. 1 [file 44318_2025_672_MOESM3_ESM.zip › Figure 1/1F/W145G_ALIX.tif]

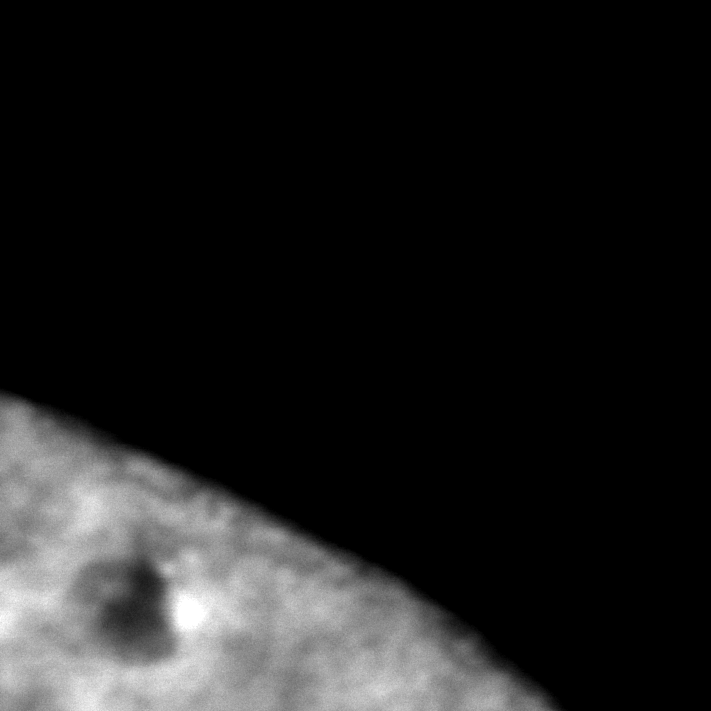

Supplement: Supplementary file 3 — Source data Fig. 1 [file 44318_2025_672_MOESM3_ESM.zip › Figure 1/1F/W145G_DAPI.tif]

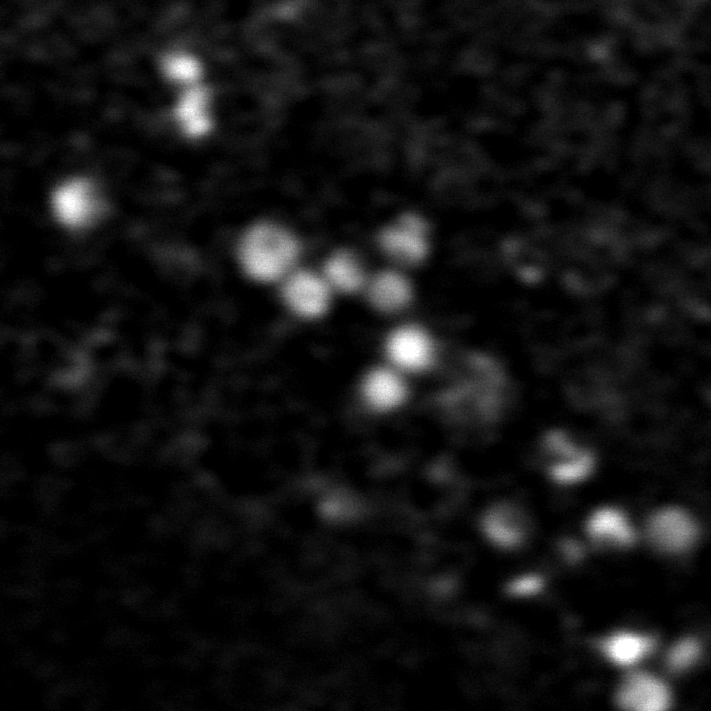

Supplement: Supplementary file 3 — Source data Fig. 1 [file 44318_2025_672_MOESM3_ESM.zip › Figure 1/1F/W145G_LAMP.tif]

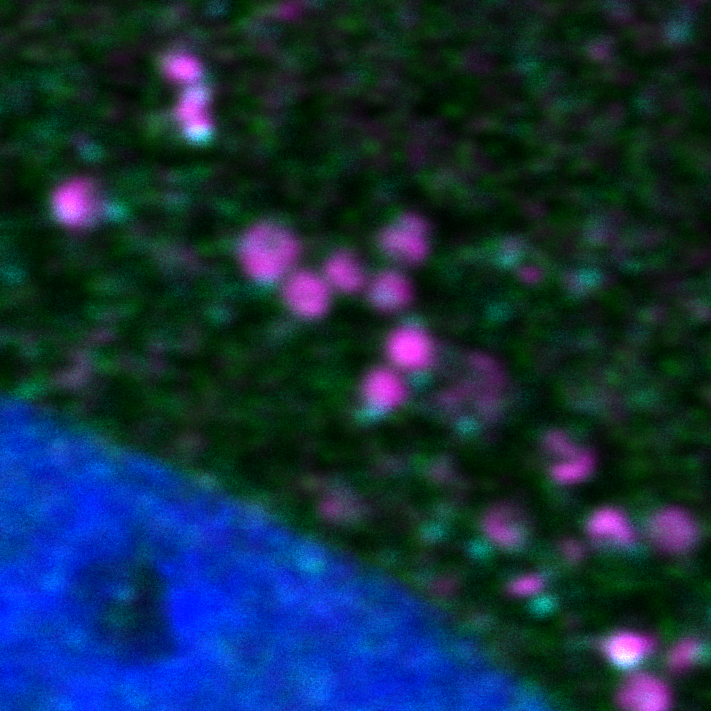

Supplement: Supplementary file 3 — Source data Fig. 1 [file 44318_2025_672_MOESM3_ESM.zip › Figure 1/1F/W145G_merge.tif]

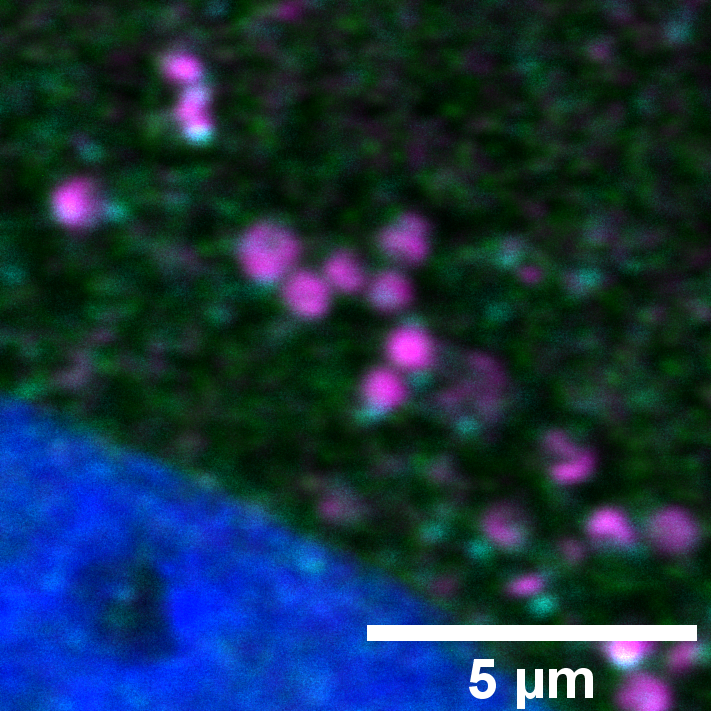

Supplement: Supplementary file 3 — Source data Fig. 1 [file 44318_2025_672_MOESM3_ESM.zip › Figure 1/1F/W145G_scale.tif]

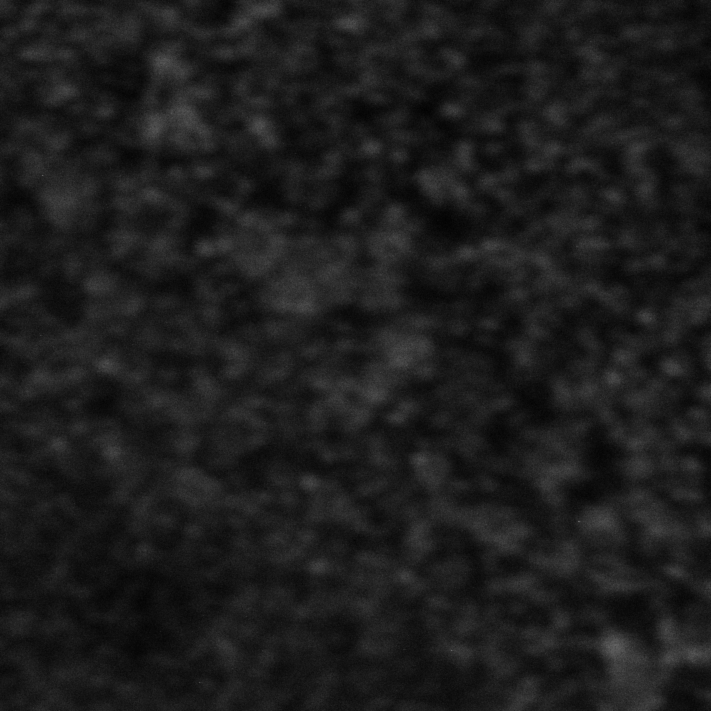

Supplement: Supplementary file 3 — Source data Fig. 1 [file 44318_2025_672_MOESM3_ESM.zip › Figure 1/1F/W145G_TECPR1.tif]

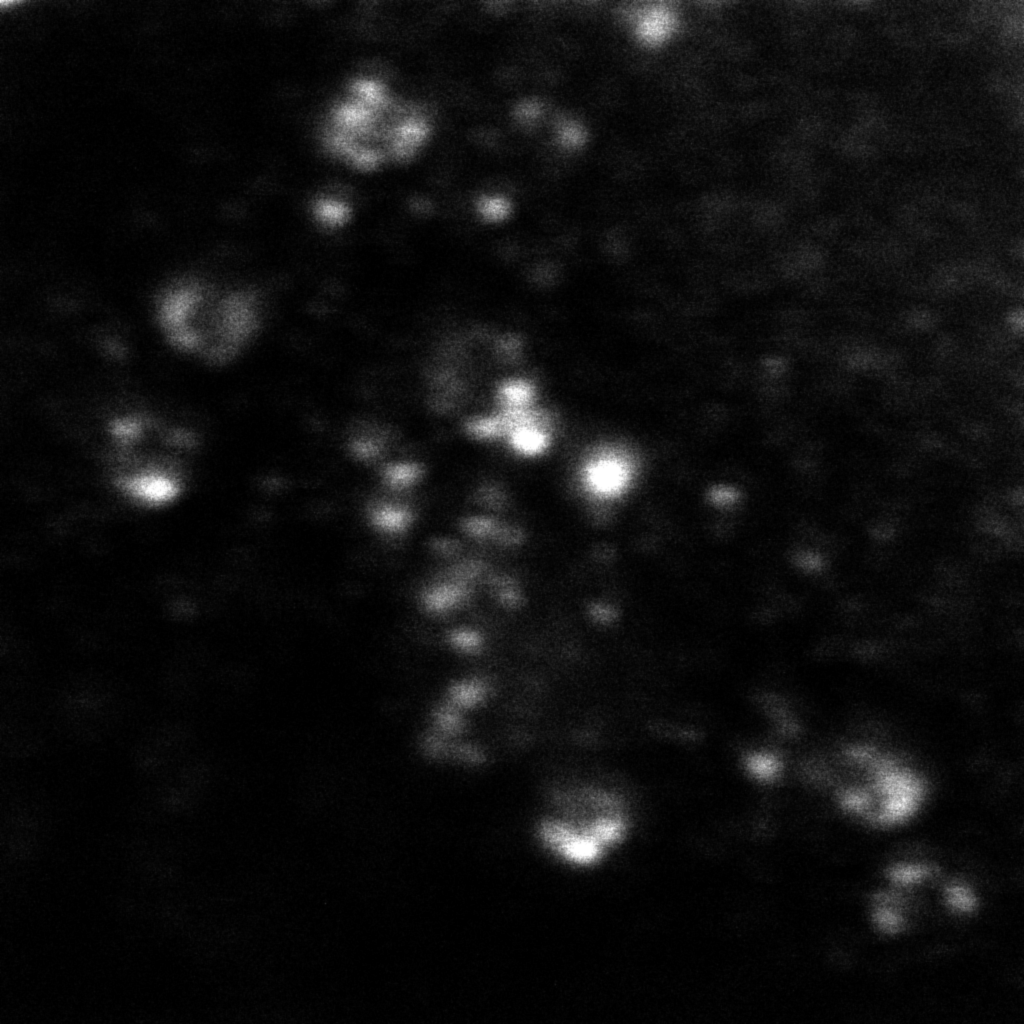

Supplement: Supplementary file 3 — Source data Fig. 1 [file 44318_2025_672_MOESM3_ESM.zip › Figure 1/1F/WT_ALIX.tif]

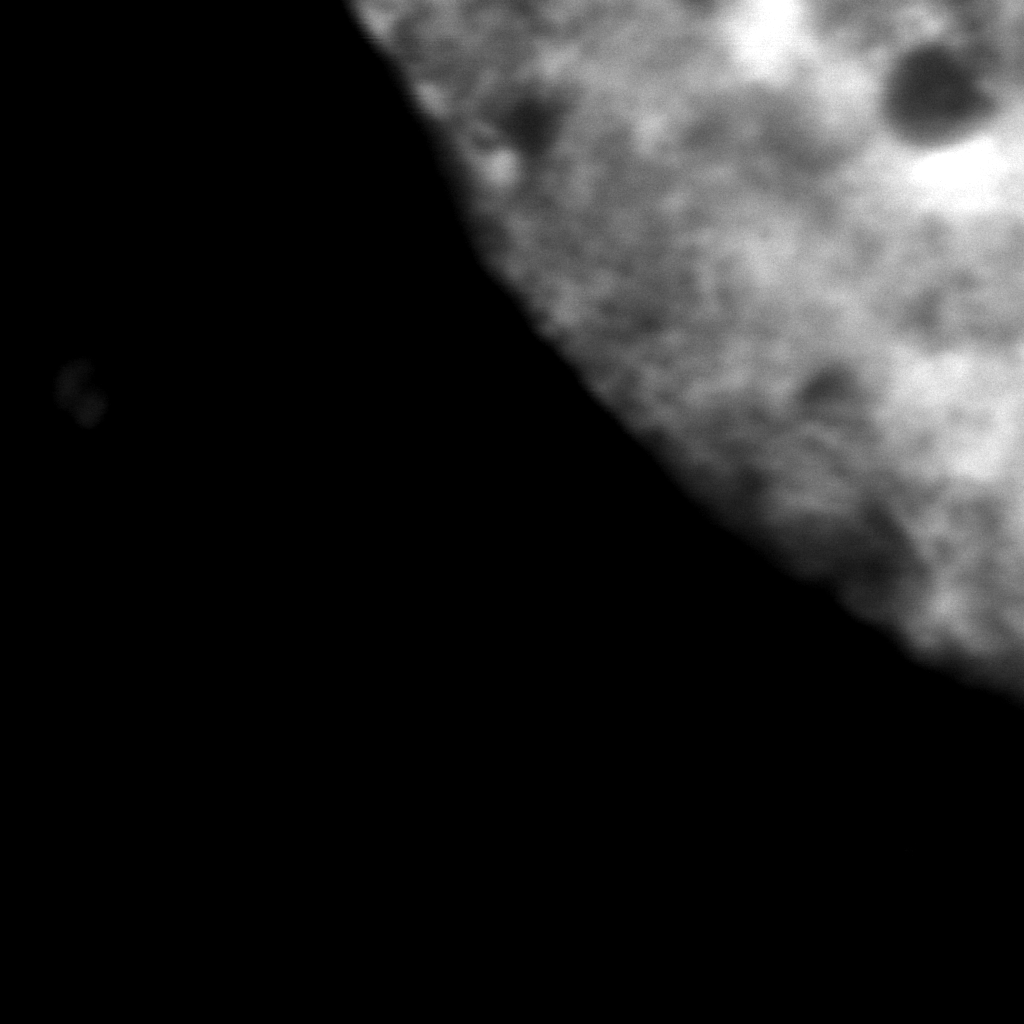

Supplement: Supplementary file 3 — Source data Fig. 1 [file 44318_2025_672_MOESM3_ESM.zip › Figure 1/1F/WT_DAPI.tif]

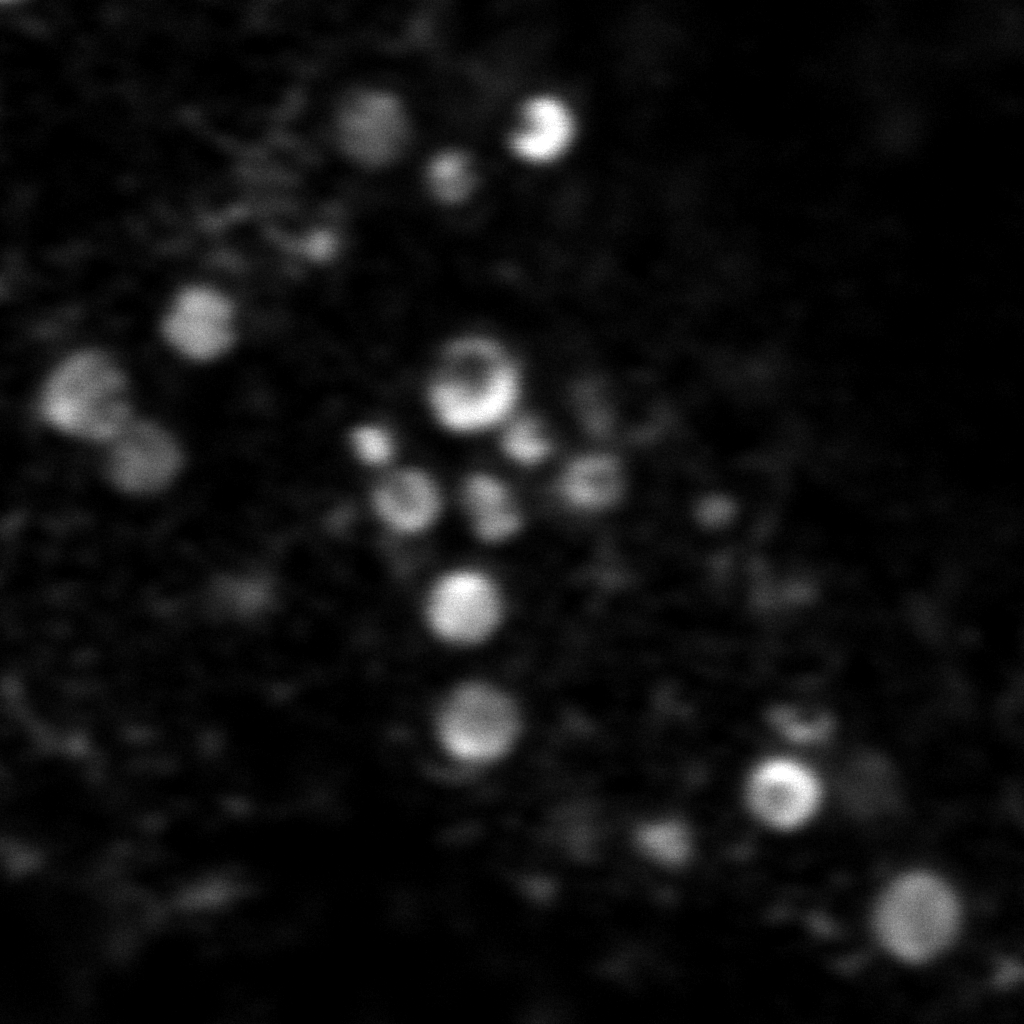

Supplement: Supplementary file 3 — Source data Fig. 1 [file 44318_2025_672_MOESM3_ESM.zip › Figure 1/1F/WT_LAMP.tif]

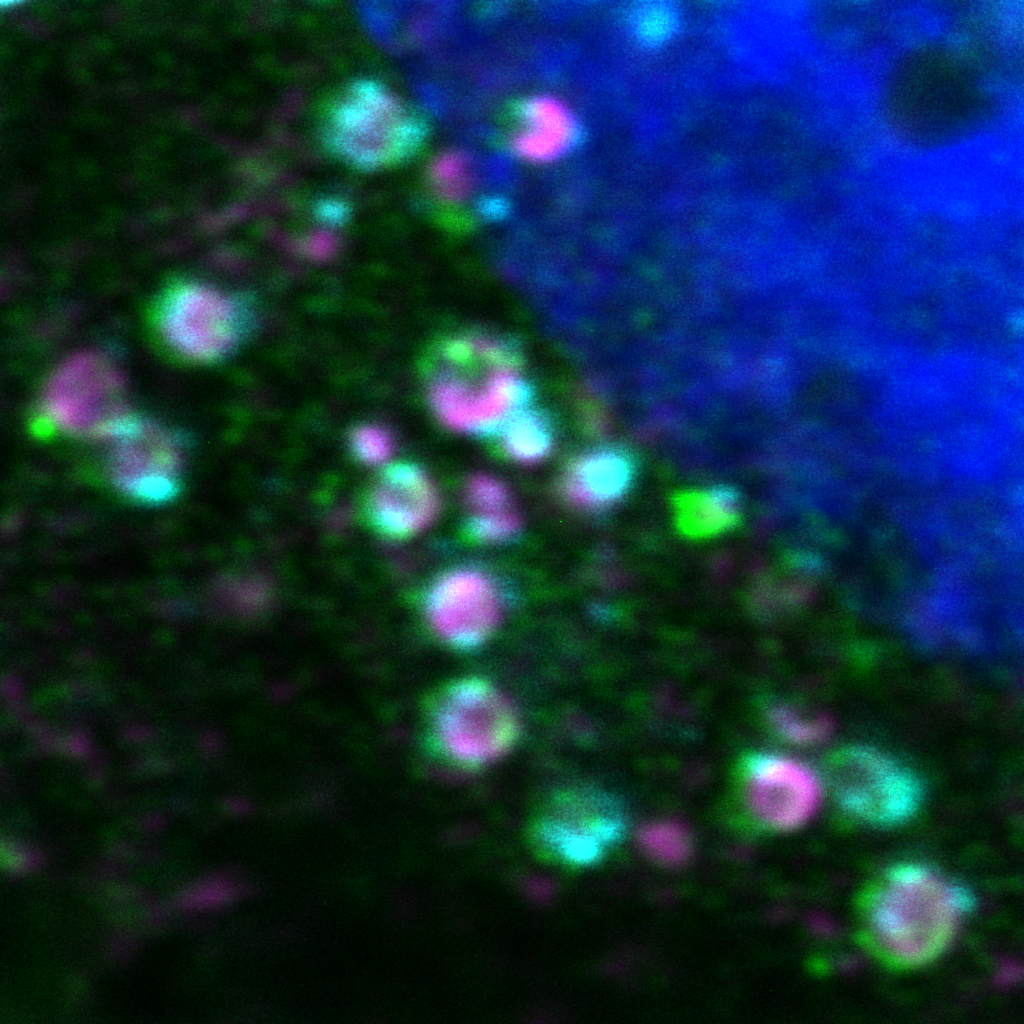

Supplement: Supplementary file 3 — Source data Fig. 1 [file 44318_2025_672_MOESM3_ESM.zip › Figure 1/1F/WT_merge.tif]

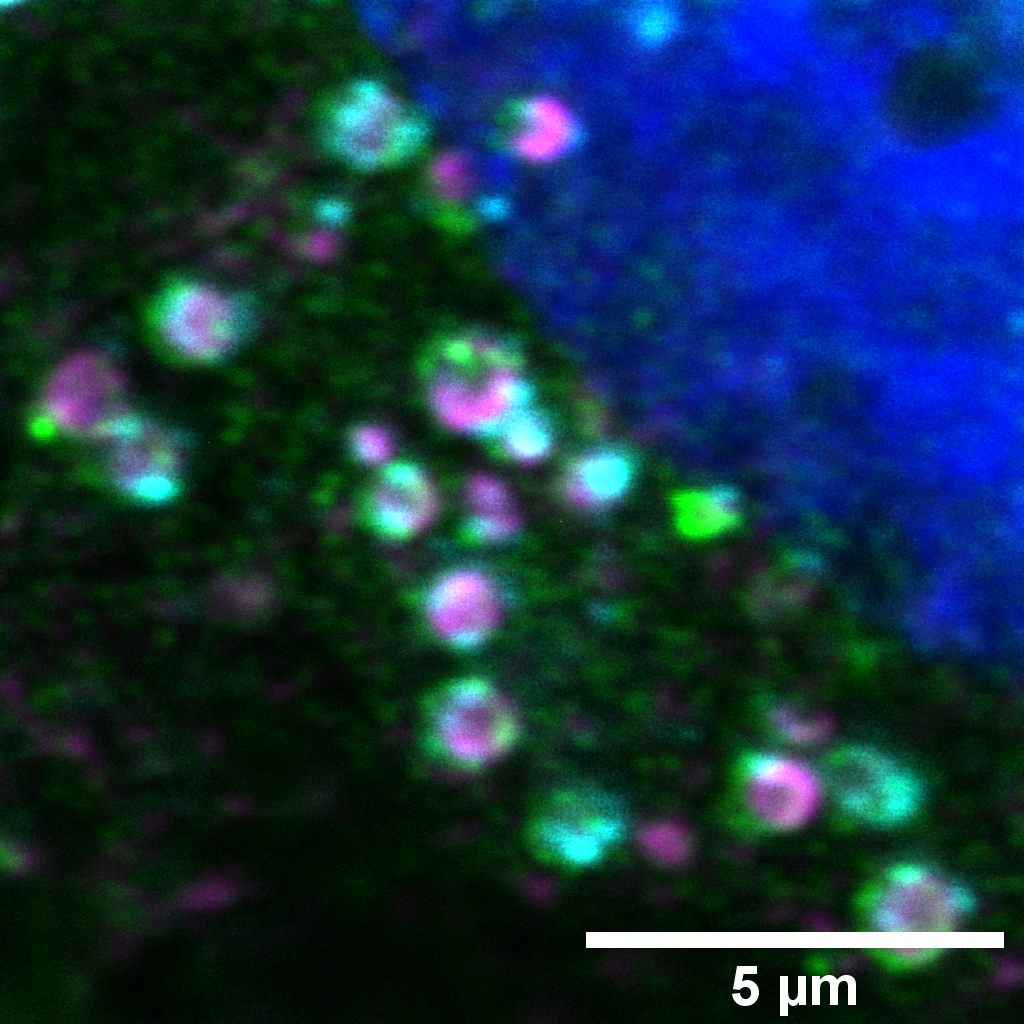

Supplement: Supplementary file 3 — Source data Fig. 1 [file 44318_2025_672_MOESM3_ESM.zip › Figure 1/1F/WT_scale.tif]

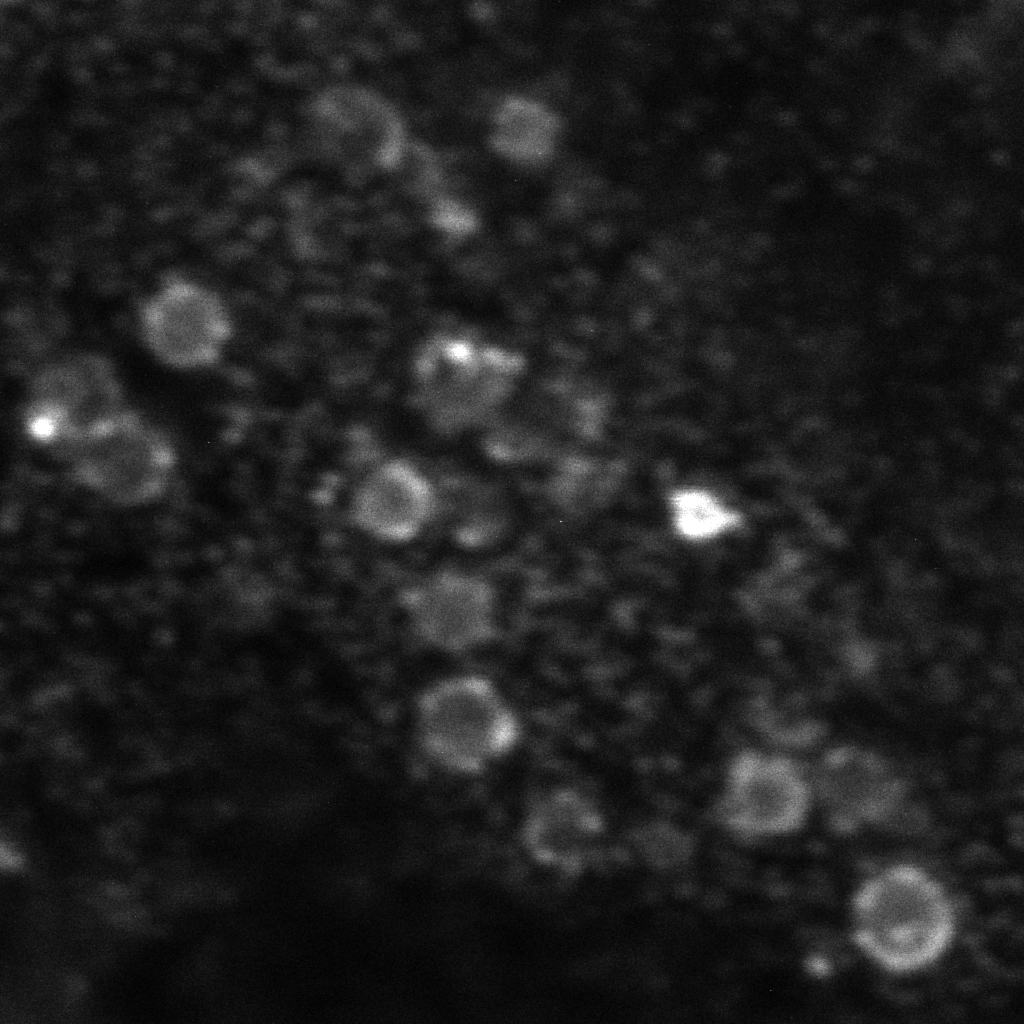

Supplement: Supplementary file 3 — Source data Fig. 1 [file 44318_2025_672_MOESM3_ESM.zip › Figure 1/1F/WT_TECPR1.tif]

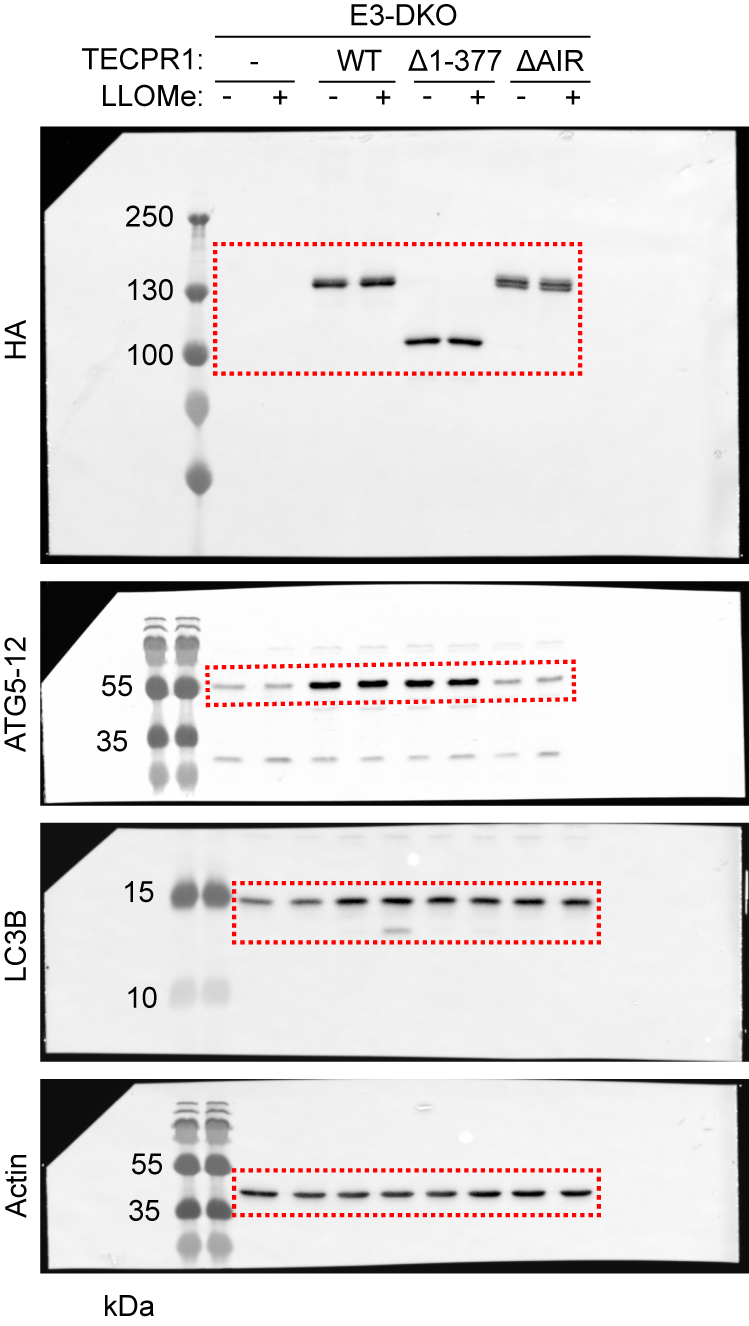

Supplement: Supplementary file 3 — Source data Fig. 1 [file 44318_2025_672_MOESM3_ESM.zip › Figure 1/1G/1G_uncropped blots.jpg]

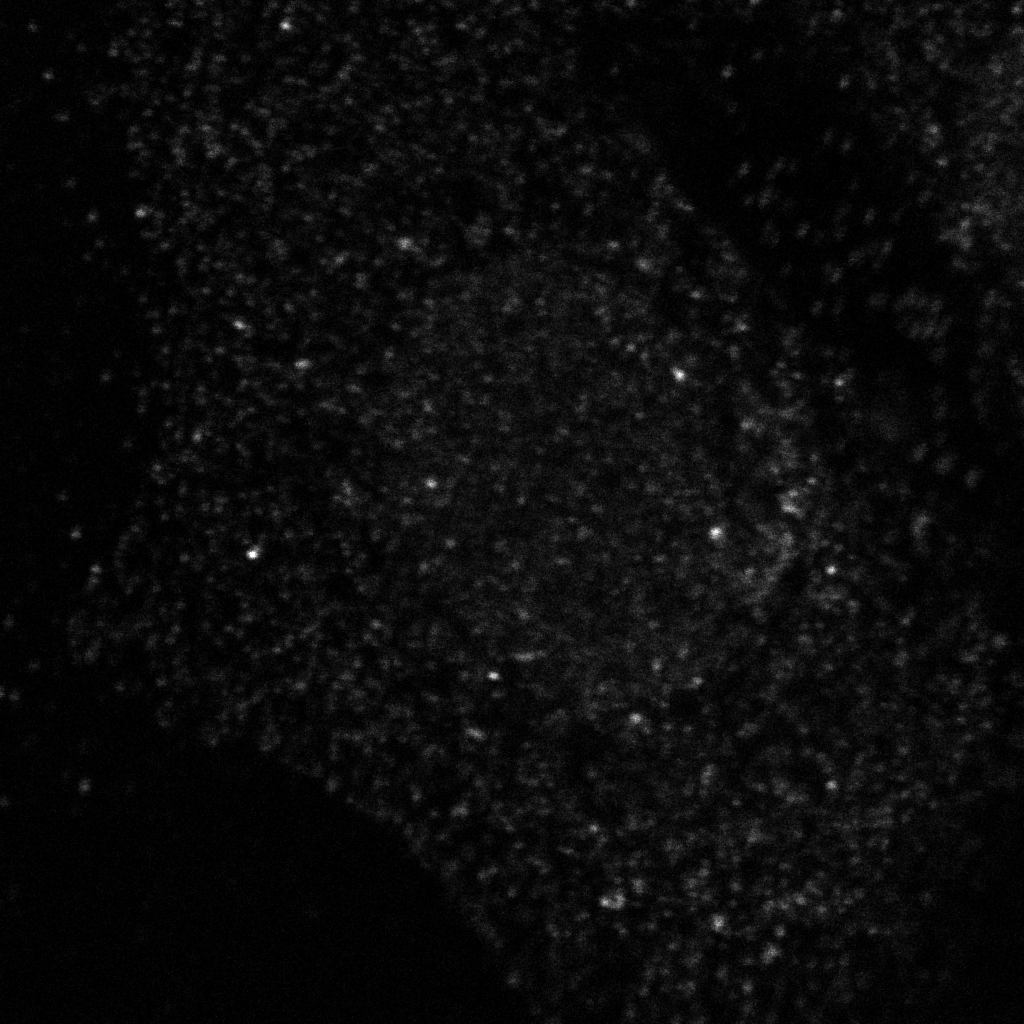

Supplement: Supplementary file 3 — Source data Fig. 1 [file 44318_2025_672_MOESM3_ESM.zip › Figure 1/1H/d1-377_ALIX.tif]

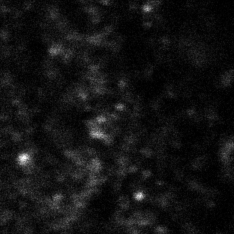

Supplement: Supplementary file 3 — Source data Fig. 1 [file 44318_2025_672_MOESM3_ESM.zip › Figure 1/1H/d1-377_ALIX_zoom.tif]

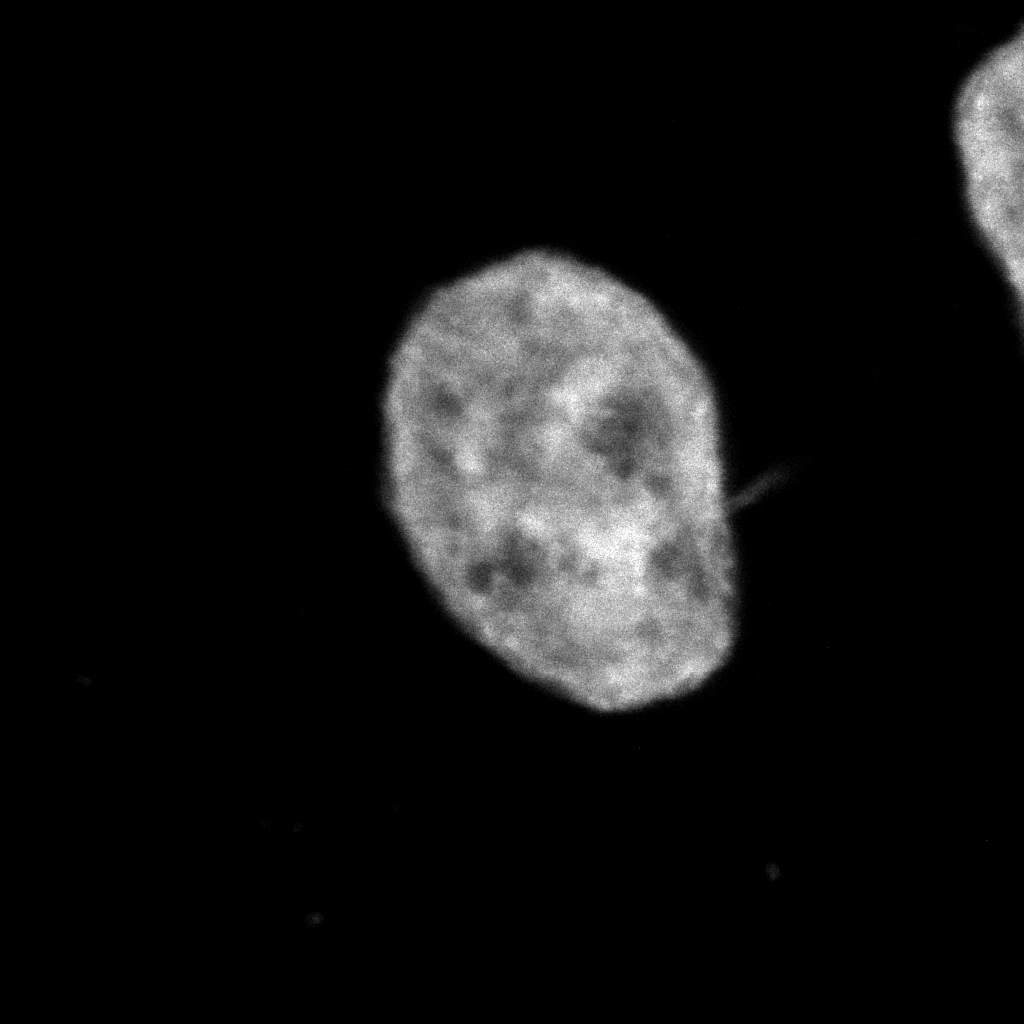

Supplement: Supplementary file 3 — Source data Fig. 1 [file 44318_2025_672_MOESM3_ESM.zip › Figure 1/1H/d1-377_DAPI.tif]

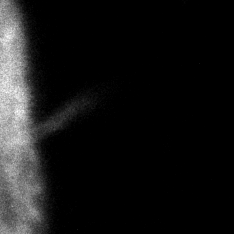

Supplement: Supplementary file 3 — Source data Fig. 1 [file 44318_2025_672_MOESM3_ESM.zip › Figure 1/1H/d1-377_DAPI_zoom.tif]

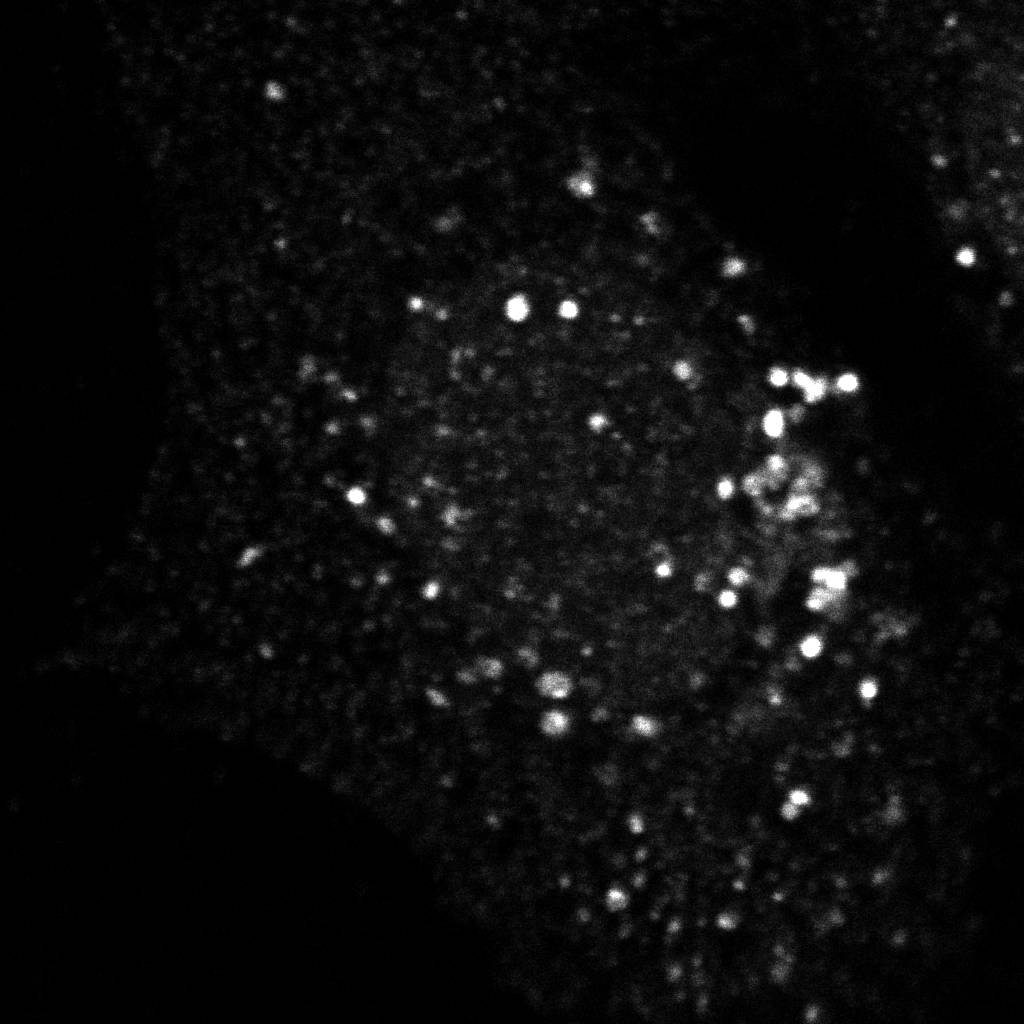

Supplement: Supplementary file 3 — Source data Fig. 1 [file 44318_2025_672_MOESM3_ESM.zip › Figure 1/1H/d1-377_Gal3.tif]

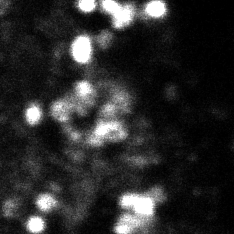

Supplement: Supplementary file 3 — Source data Fig. 1 [file 44318_2025_672_MOESM3_ESM.zip › Figure 1/1H/d1-377_Gal3_zoom.tif]

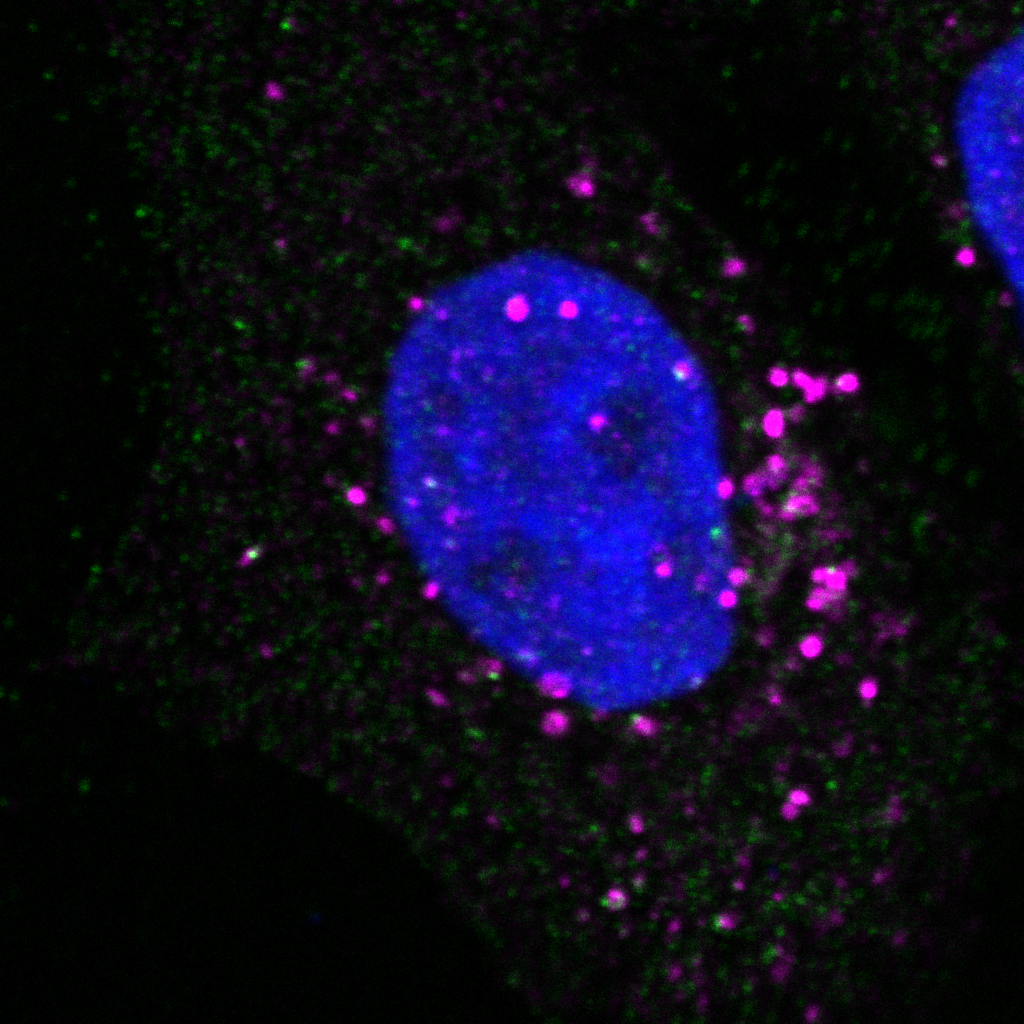

Supplement: Supplementary file 3 — Source data Fig. 1 [file 44318_2025_672_MOESM3_ESM.zip › Figure 1/1H/d1-377_merge.tif]

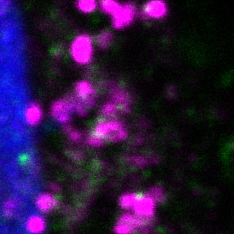

Supplement: Supplementary file 3 — Source data Fig. 1 [file 44318_2025_672_MOESM3_ESM.zip › Figure 1/1H/d1-377_merge_zoom.tif]

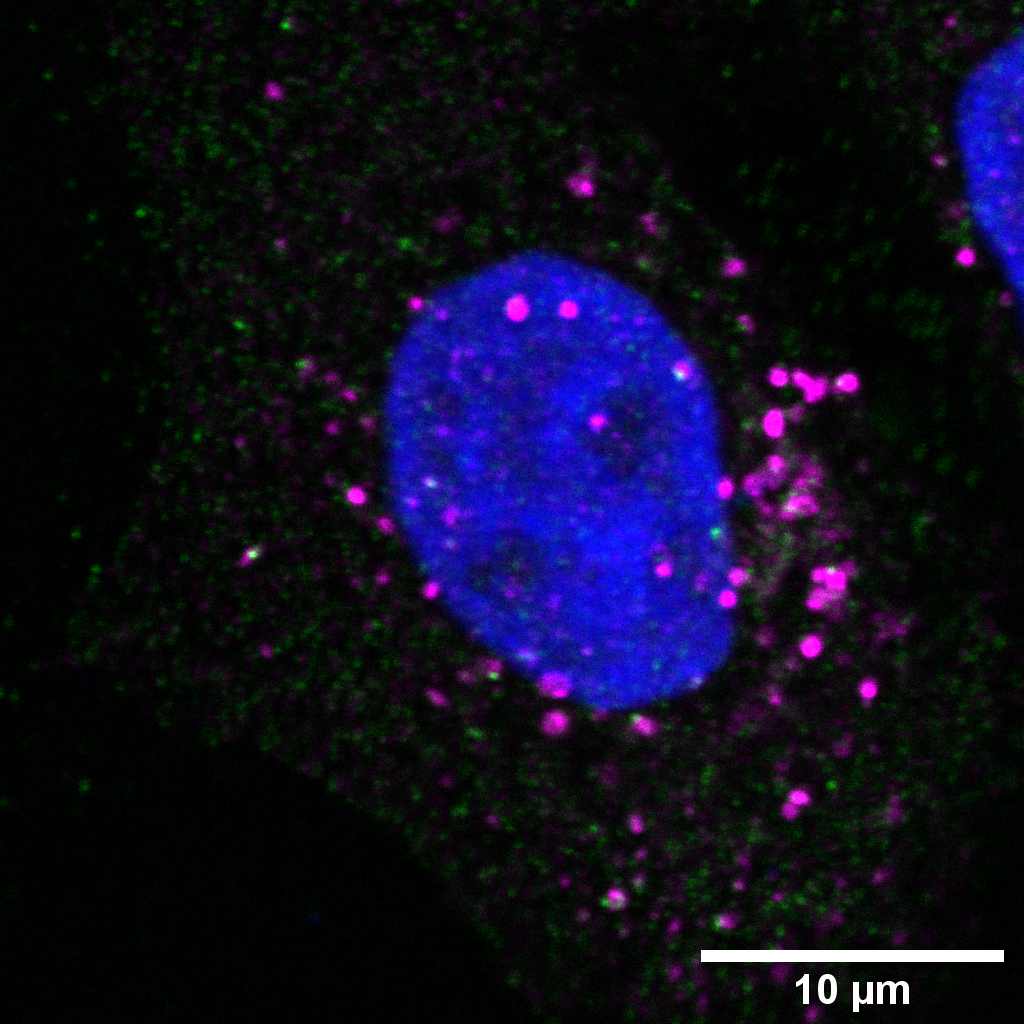

Supplement: Supplementary file 3 — Source data Fig. 1 [file 44318_2025_672_MOESM3_ESM.zip › Figure 1/1H/d1-377_scale.tif]

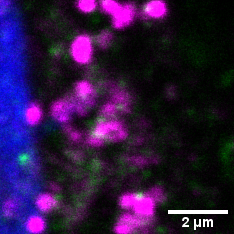

Supplement: Supplementary file 3 — Source data Fig. 1 [file 44318_2025_672_MOESM3_ESM.zip › Figure 1/1H/d1-377_scale_zoom.tif]

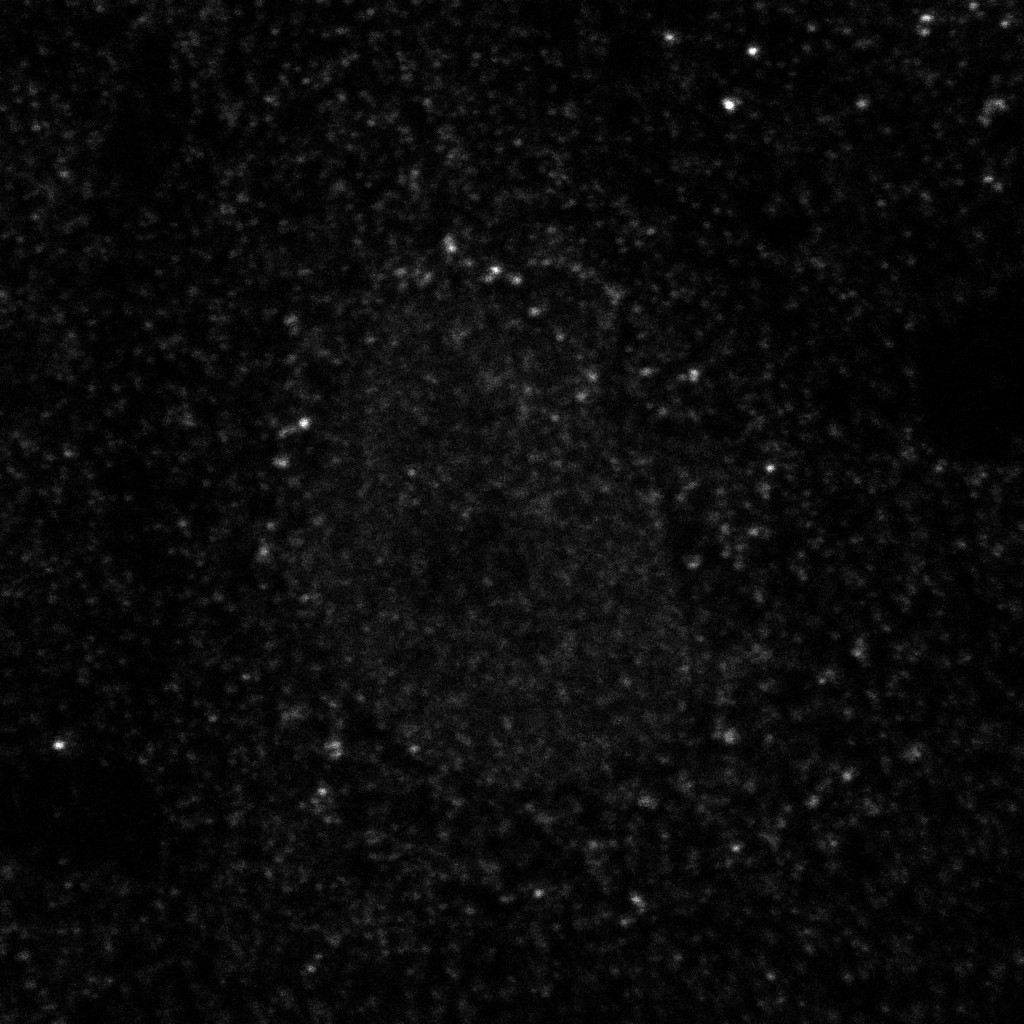

Supplement: Supplementary file 3 — Source data Fig. 1 [file 44318_2025_672_MOESM3_ESM.zip › Figure 1/1H/dAIR_ALIX.tif]

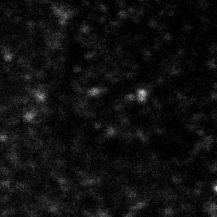

Supplement: Supplementary file 3 — Source data Fig. 1 [file 44318_2025_672_MOESM3_ESM.zip › Figure 1/1H/dAIR_ALIX_zoom.tif]

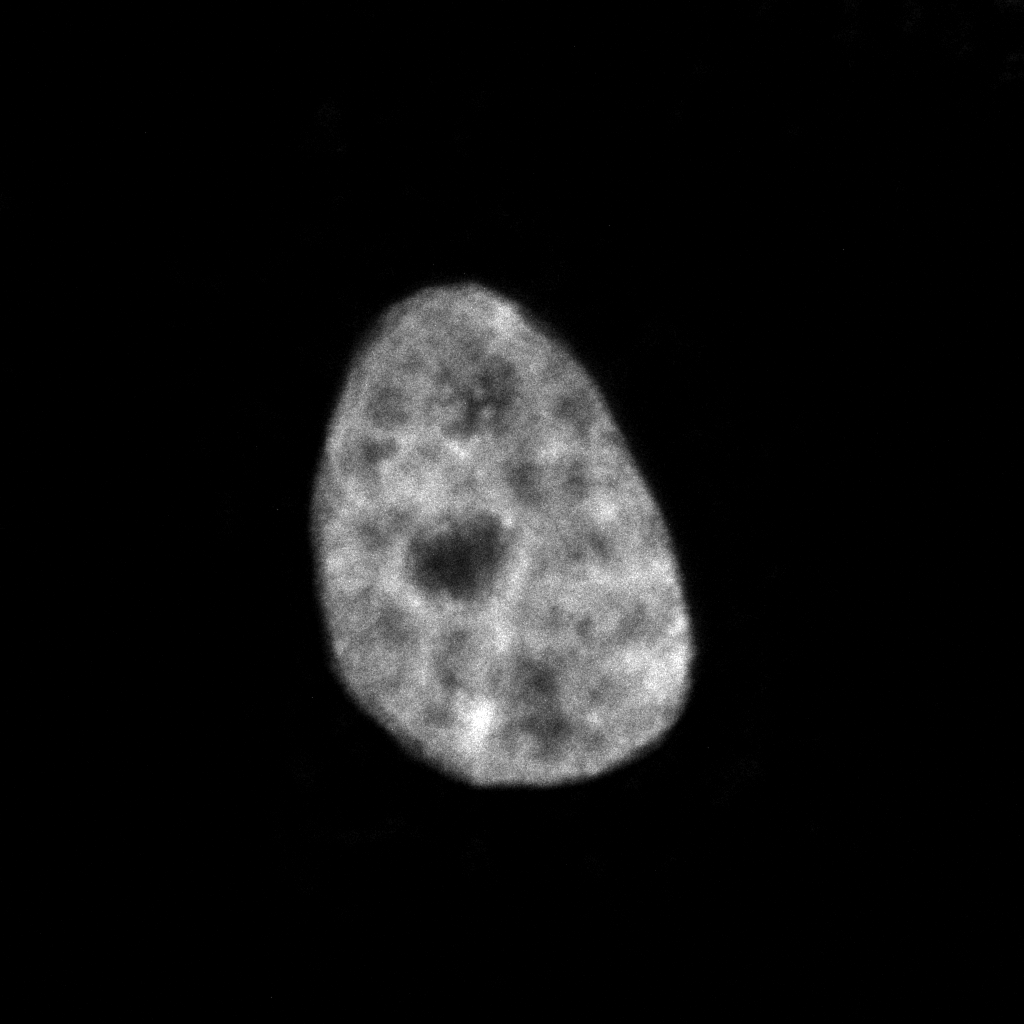

Supplement: Supplementary file 3 — Source data Fig. 1 [file 44318_2025_672_MOESM3_ESM.zip › Figure 1/1H/dAIR_DAPI.tif]

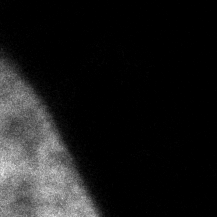

Supplement: Supplementary file 3 — Source data Fig. 1 [file 44318_2025_672_MOESM3_ESM.zip › Figure 1/1H/dAIR_DAPI_zoom.tif]

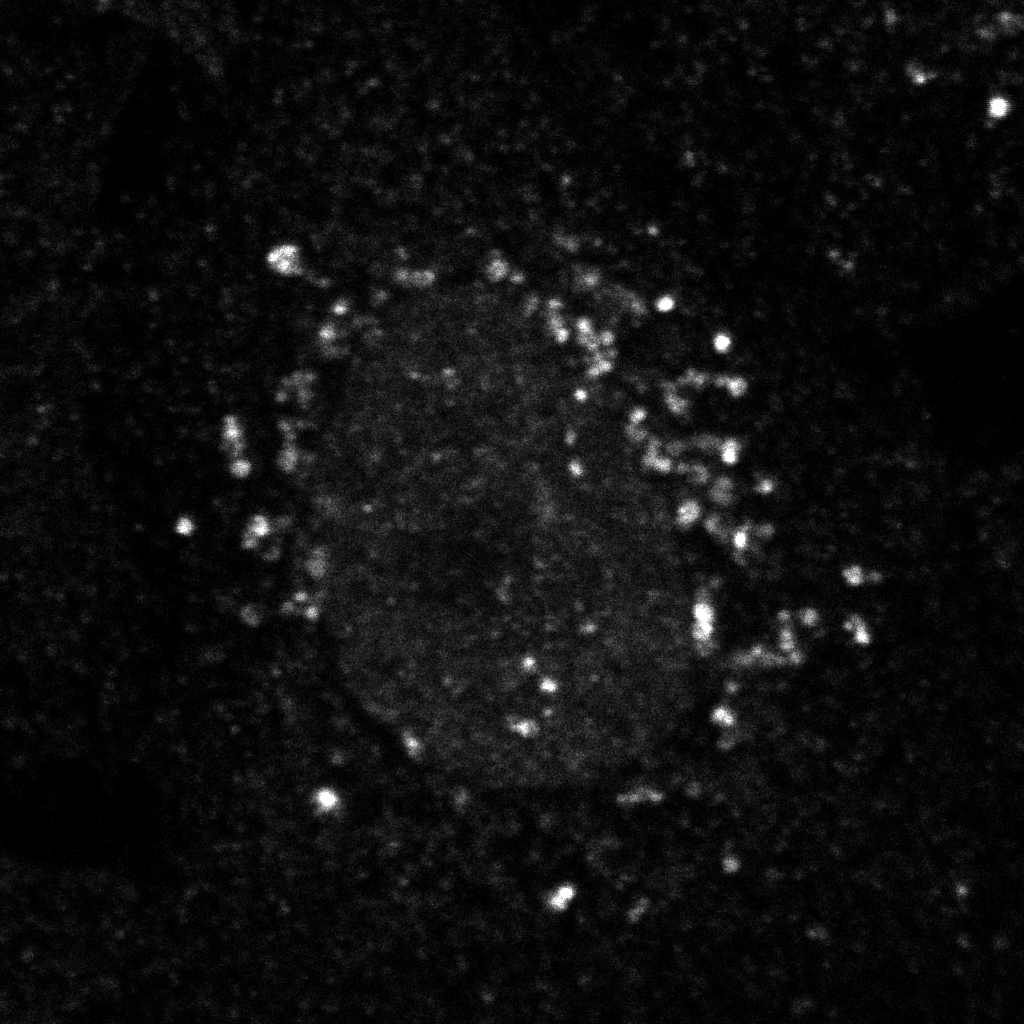

Supplement: Supplementary file 3 — Source data Fig. 1 [file 44318_2025_672_MOESM3_ESM.zip › Figure 1/1H/dAIR_Gal3.tif]

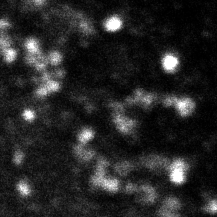

Supplement: Supplementary file 3 — Source data Fig. 1 [file 44318_2025_672_MOESM3_ESM.zip › Figure 1/1H/dAIR_Gal3_zoom.tif]

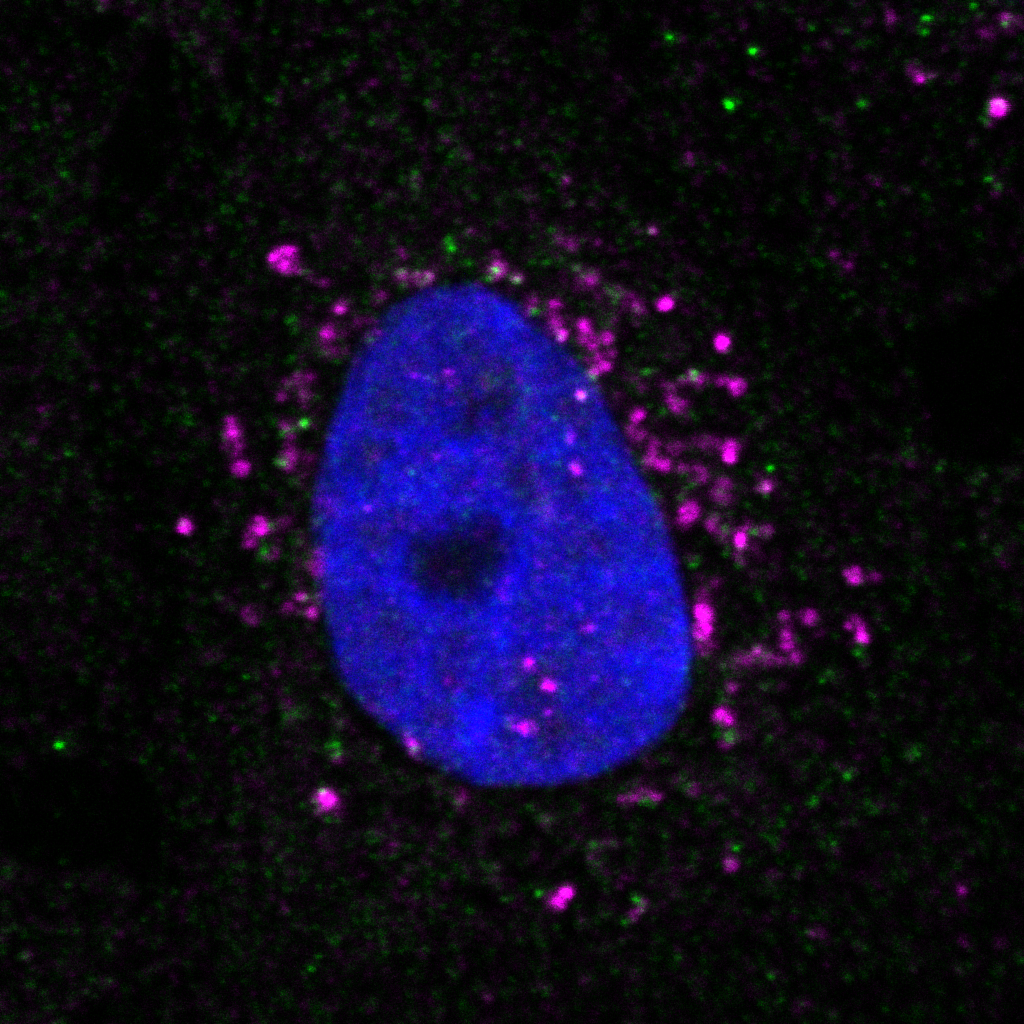

Supplement: Supplementary file 3 — Source data Fig. 1 [file 44318_2025_672_MOESM3_ESM.zip › Figure 1/1H/dAIR_merge.tif]

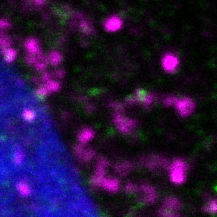

Supplement: Supplementary file 3 — Source data Fig. 1 [file 44318_2025_672_MOESM3_ESM.zip › Figure 1/1H/dAIR_merge_zoom.tif]

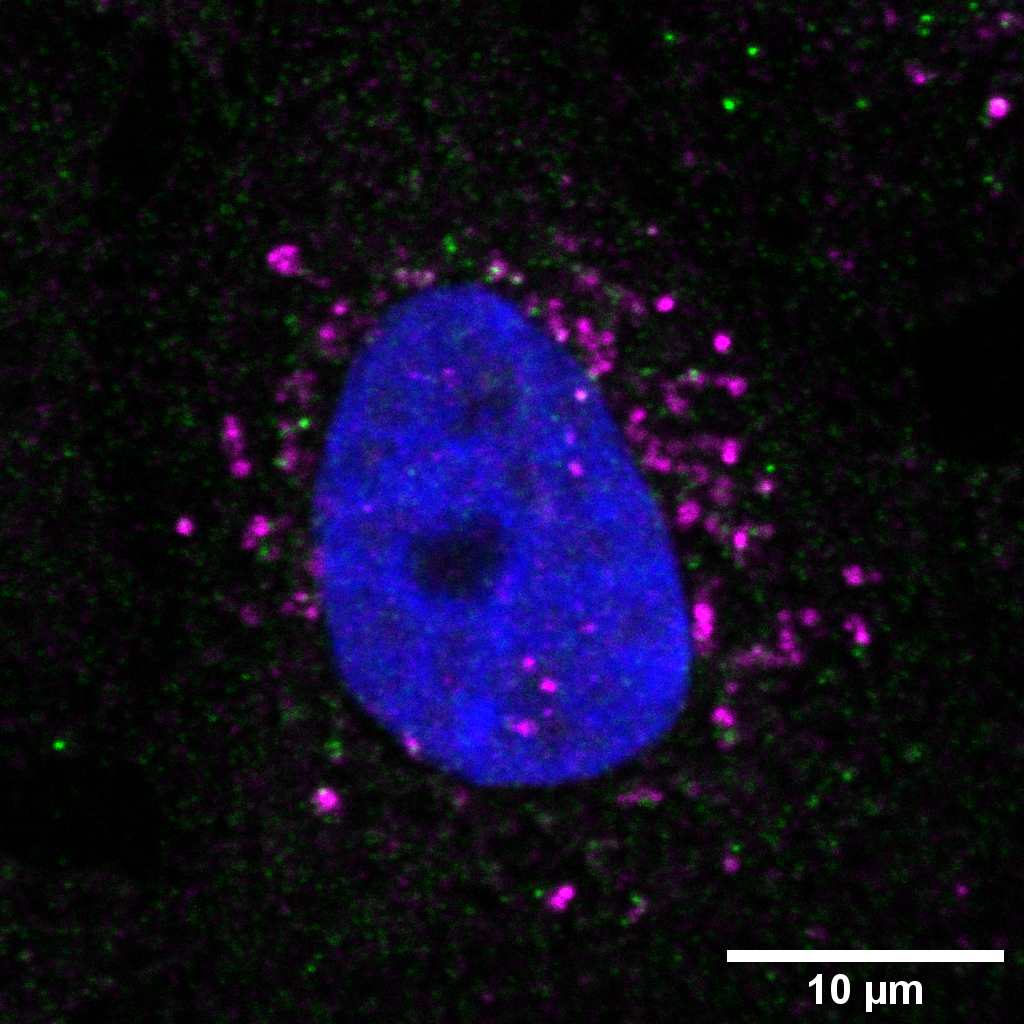

Supplement: Supplementary file 3 — Source data Fig. 1 [file 44318_2025_672_MOESM3_ESM.zip › Figure 1/1H/dAIR_scale.tif]
