# Supplementary figures and images for: The ATG8 E3-like ligases sense lysosomal damage and initiate ESCRT-mediated membrane repair (part 4 of 7)
Source: EMBO J. 2026 Jan 3;45(3):930–52. doi: 10.1038/s44318-025-00672-1 (PMC12865045; doi:10.1038/s44318-025-00672-1)

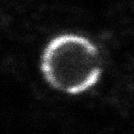

Supplement: Supplementary file 5 — Source data Fig. 3 [file 44318_2025_672_MOESM5_ESM.zip › Figure 3/3D/Individual Vesicles/WT/a_IST1.tif]

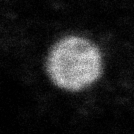

Supplement: Supplementary file 5 — Source data Fig. 3 [file 44318_2025_672_MOESM5_ESM.zip › Figure 3/3D/Individual Vesicles/WT/a_LAMP.tif]

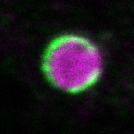

Supplement: Supplementary file 5 — Source data Fig. 3 [file 44318_2025_672_MOESM5_ESM.zip › Figure 3/3D/Individual Vesicles/WT/a_merge.tif]

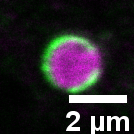

Supplement: Supplementary file 5 — Source data Fig. 3 [file 44318_2025_672_MOESM5_ESM.zip › Figure 3/3D/Individual Vesicles/WT/a_scale.tif]

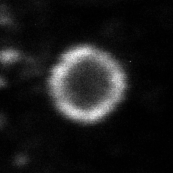

Supplement: Supplementary file 5 — Source data Fig. 3 [file 44318_2025_672_MOESM5_ESM.zip › Figure 3/3D/Individual Vesicles/WT/b_IST1.tif]

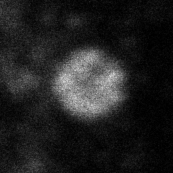

Supplement: Supplementary file 5 — Source data Fig. 3 [file 44318_2025_672_MOESM5_ESM.zip › Figure 3/3D/Individual Vesicles/WT/b_LAMP.tif]

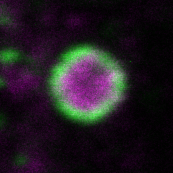

Supplement: Supplementary file 5 — Source data Fig. 3 [file 44318_2025_672_MOESM5_ESM.zip › Figure 3/3D/Individual Vesicles/WT/b_merge.tif]

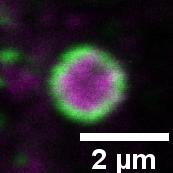

Supplement: Supplementary file 5 — Source data Fig. 3 [file 44318_2025_672_MOESM5_ESM.zip › Figure 3/3D/Individual Vesicles/WT/b_scale.tif]

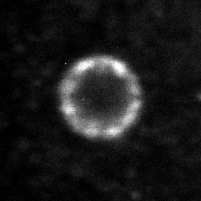

Supplement: Supplementary file 5 — Source data Fig. 3 [file 44318_2025_672_MOESM5_ESM.zip › Figure 3/3D/Individual Vesicles/WT/c_IST1.tif]

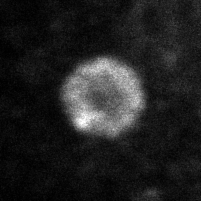

Supplement: Supplementary file 5 — Source data Fig. 3 [file 44318_2025_672_MOESM5_ESM.zip › Figure 3/3D/Individual Vesicles/WT/c_LAMP.tif]

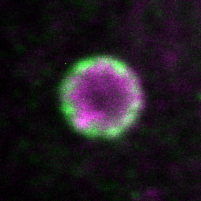

Supplement: Supplementary file 5 — Source data Fig. 3 [file 44318_2025_672_MOESM5_ESM.zip › Figure 3/3D/Individual Vesicles/WT/c_merge.tif]

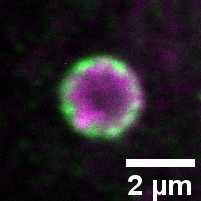

Supplement: Supplementary file 5 — Source data Fig. 3 [file 44318_2025_672_MOESM5_ESM.zip › Figure 3/3D/Individual Vesicles/WT/c_scale.tif]

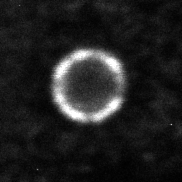

Supplement: Supplementary file 5 — Source data Fig. 3 [file 44318_2025_672_MOESM5_ESM.zip › Figure 3/3D/Individual Vesicles/WT/d_IST1.tif]

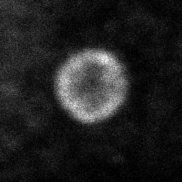

Supplement: Supplementary file 5 — Source data Fig. 3 [file 44318_2025_672_MOESM5_ESM.zip › Figure 3/3D/Individual Vesicles/WT/d_LAMP.tif]

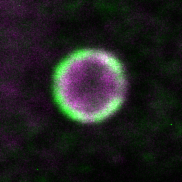

Supplement: Supplementary file 5 — Source data Fig. 3 [file 44318_2025_672_MOESM5_ESM.zip › Figure 3/3D/Individual Vesicles/WT/d_merge.tif]

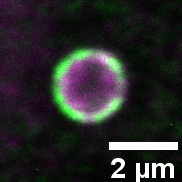

Supplement: Supplementary file 5 — Source data Fig. 3 [file 44318_2025_672_MOESM5_ESM.zip › Figure 3/3D/Individual Vesicles/WT/d_scale.tif]

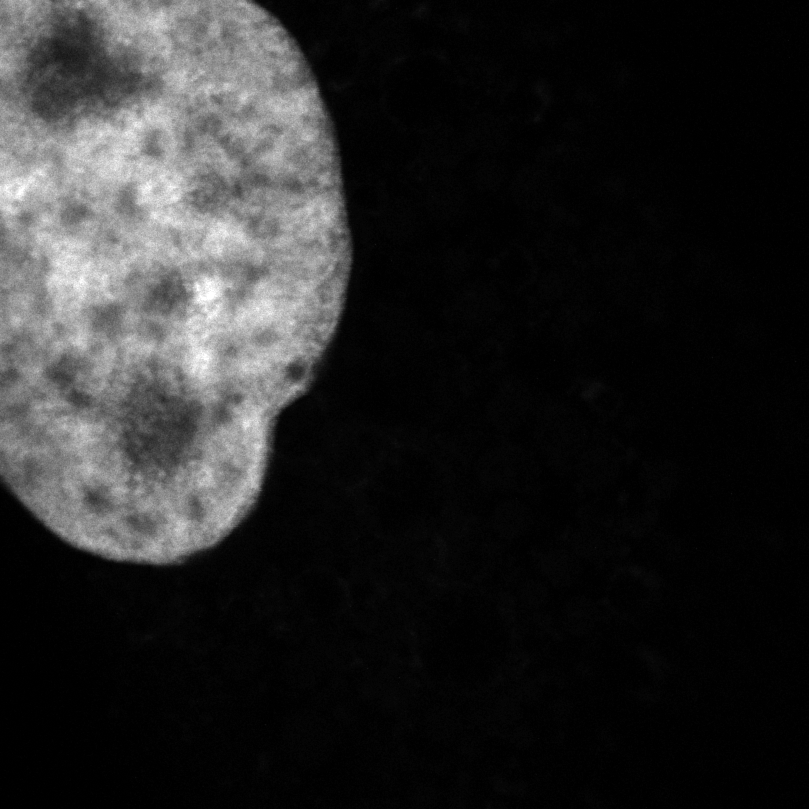

Supplement: Supplementary file 5 — Source data Fig. 3 [file 44318_2025_672_MOESM5_ESM.zip › Figure 3/3D/Individual Vesicles/WT/DAPI.tif]

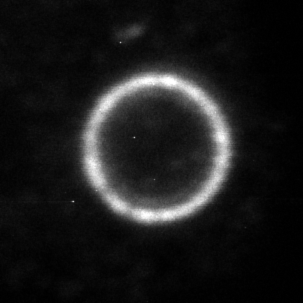

Supplement: Supplementary file 5 — Source data Fig. 3 [file 44318_2025_672_MOESM5_ESM.zip › Figure 3/3D/Individual Vesicles/WT/e_IST1.tif]

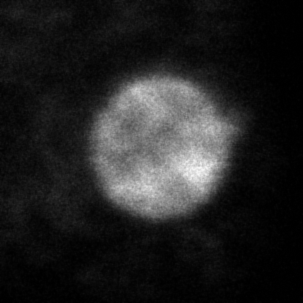

Supplement: Supplementary file 5 — Source data Fig. 3 [file 44318_2025_672_MOESM5_ESM.zip › Figure 3/3D/Individual Vesicles/WT/e_LAMP.tif]

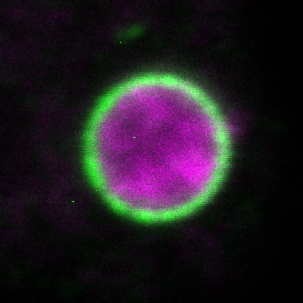

Supplement: Supplementary file 5 — Source data Fig. 3 [file 44318_2025_672_MOESM5_ESM.zip › Figure 3/3D/Individual Vesicles/WT/e_merge.tif]

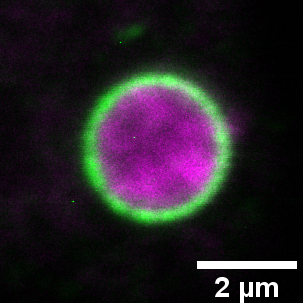

Supplement: Supplementary file 5 — Source data Fig. 3 [file 44318_2025_672_MOESM5_ESM.zip › Figure 3/3D/Individual Vesicles/WT/e_scale.tif]

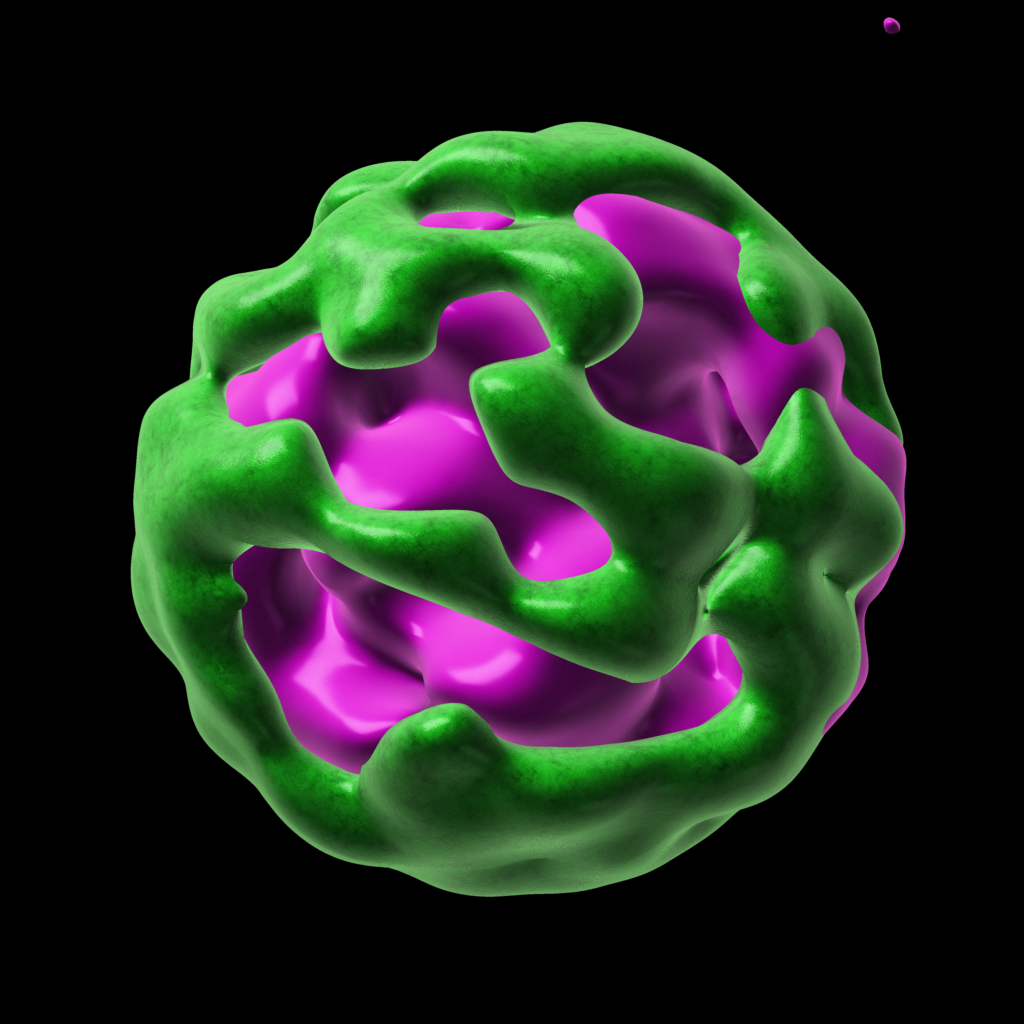

Supplement: Supplementary file 5 — Source data Fig. 3 [file 44318_2025_672_MOESM5_ESM.zip › Figure 3/3D/Individual Vesicles/WT/WT_3D.tif]

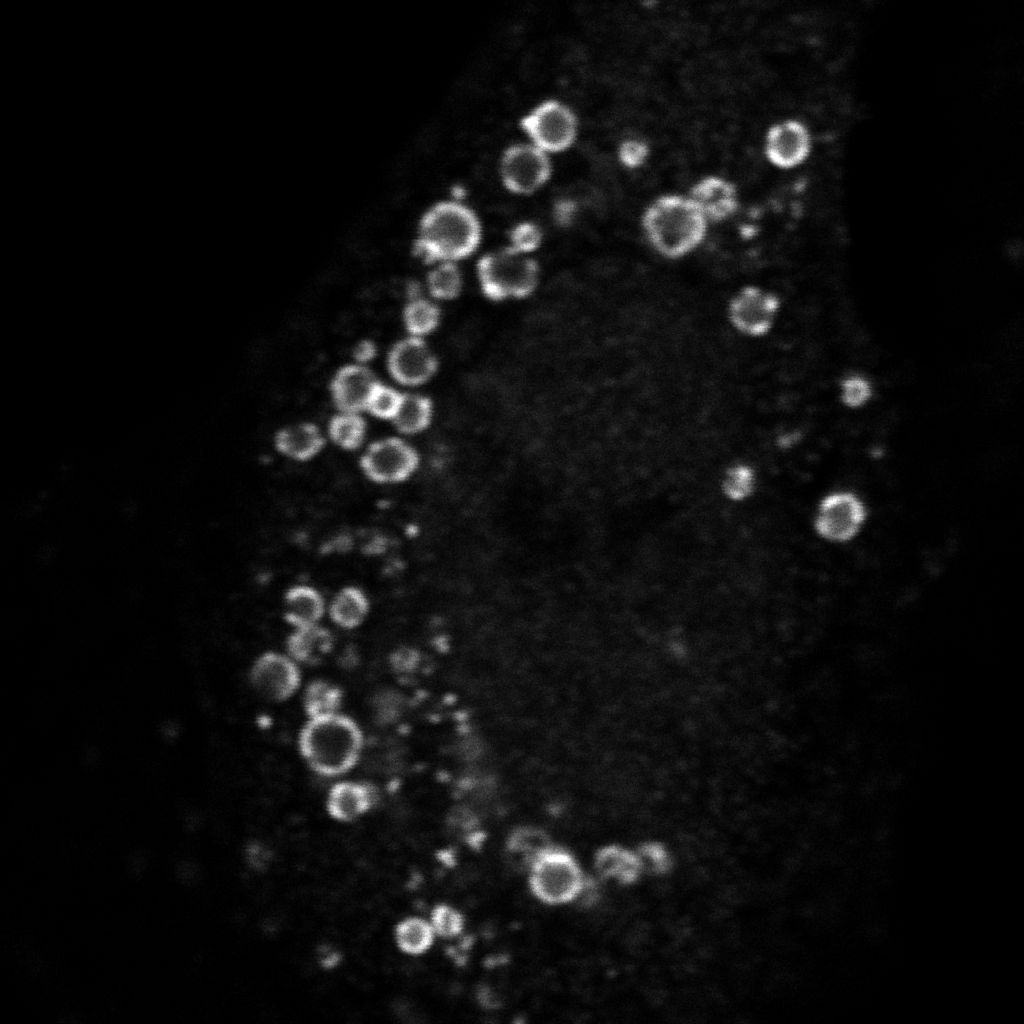

Supplement: Supplementary file 5 — Source data Fig. 3 [file 44318_2025_672_MOESM5_ESM.zip › Figure 3/3D/WT_IST1.tif]

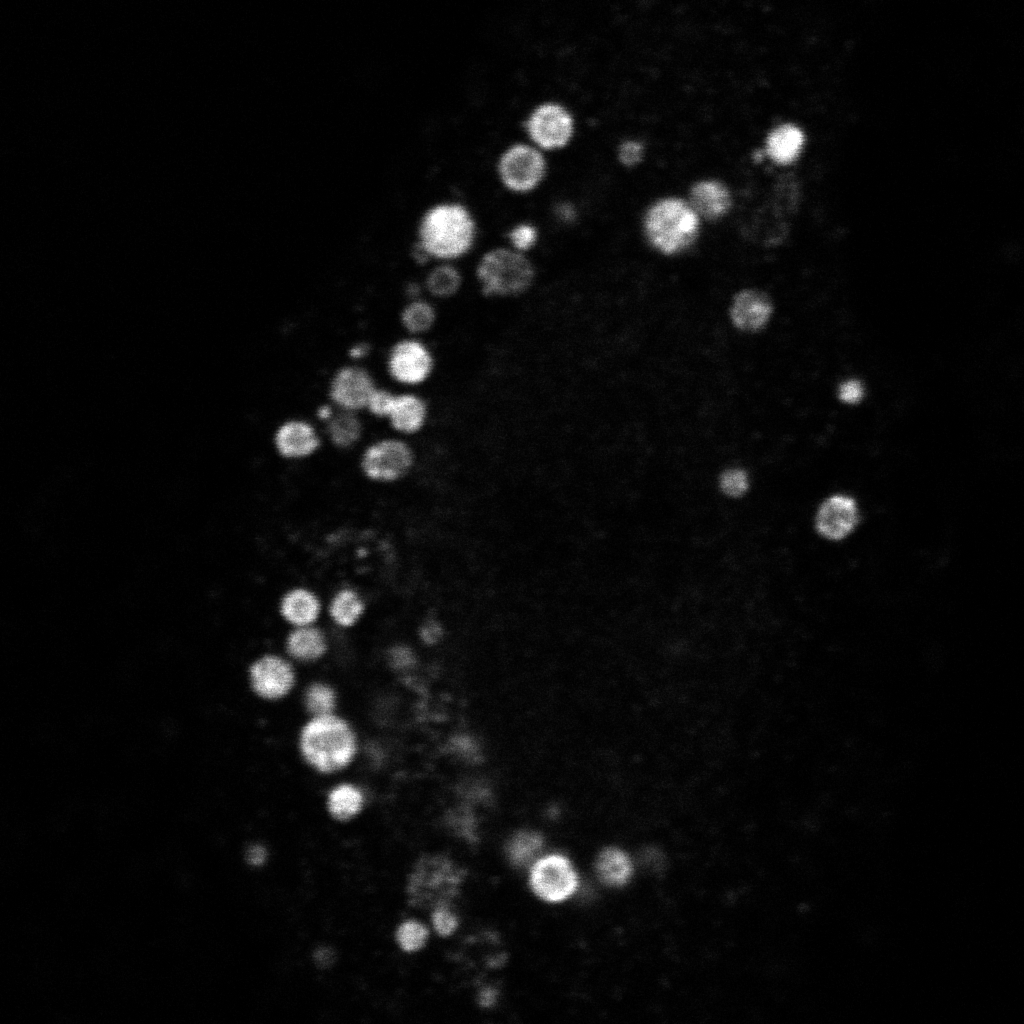

Supplement: Supplementary file 5 — Source data Fig. 3 [file 44318_2025_672_MOESM5_ESM.zip › Figure 3/3D/WT_LAMP.tif]

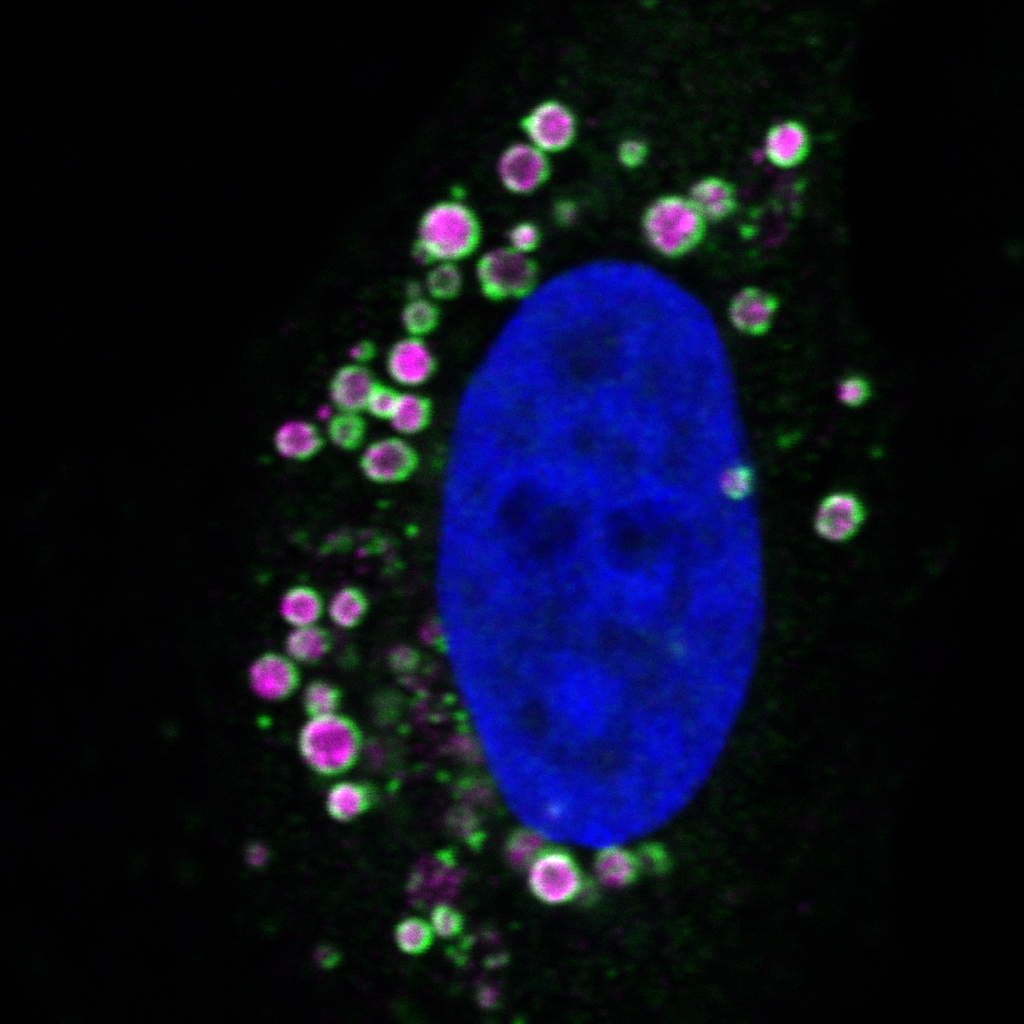

Supplement: Supplementary file 5 — Source data Fig. 3 [file 44318_2025_672_MOESM5_ESM.zip › Figure 3/3D/WT_merge.tif]

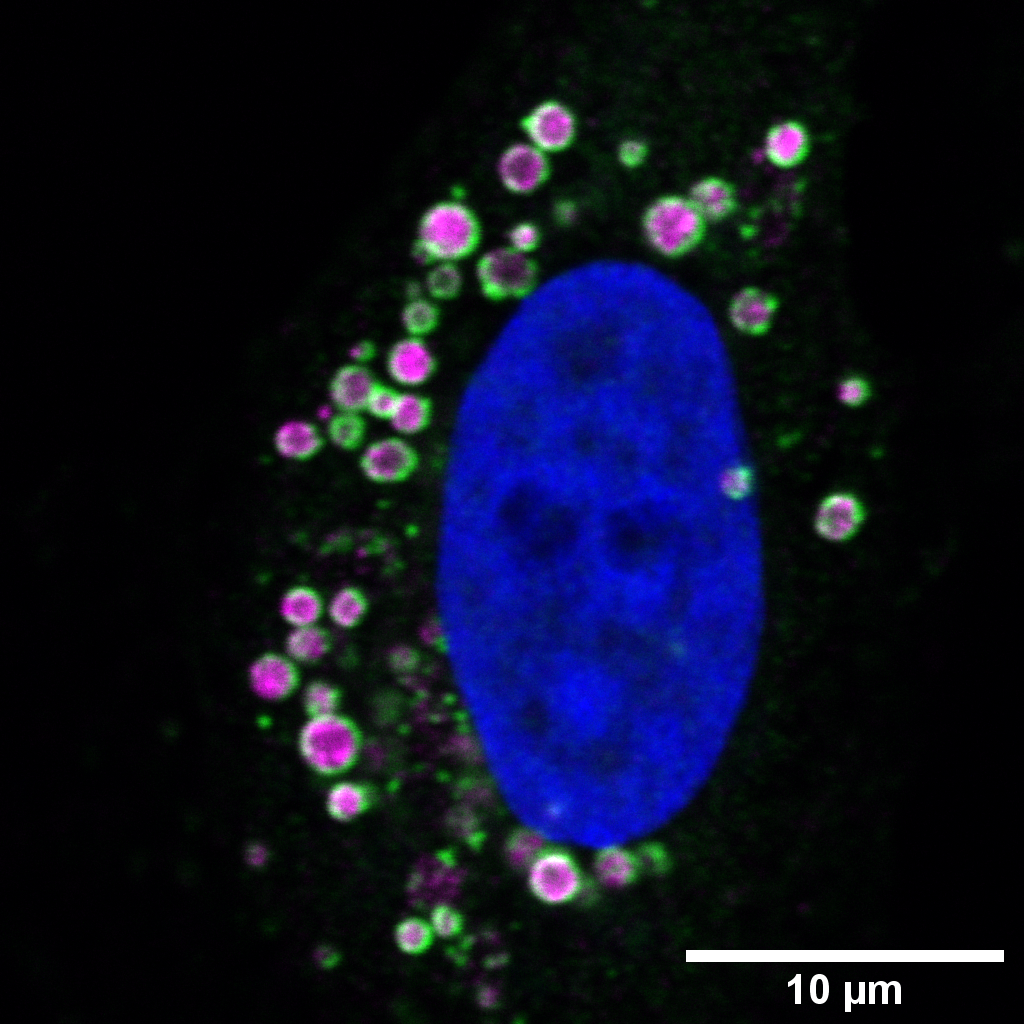

Supplement: Supplementary file 5 — Source data Fig. 3 [file 44318_2025_672_MOESM5_ESM.zip › Figure 3/3D/WT_scale.tif]

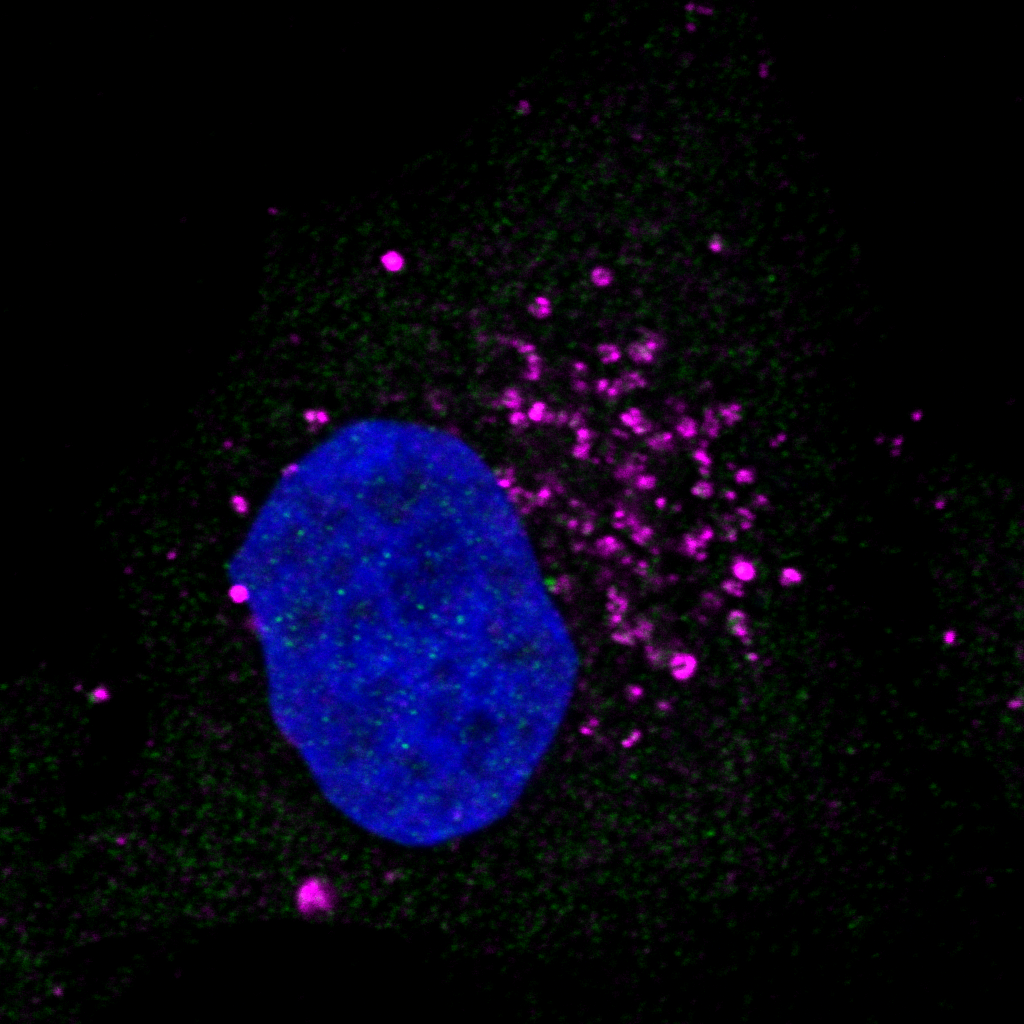

Supplement: Supplementary file 6 — Source data Fig. 4 [file 44318_2025_672_MOESM6_ESM.zip › Figure 4/4A/WT_LLOMe.tif]

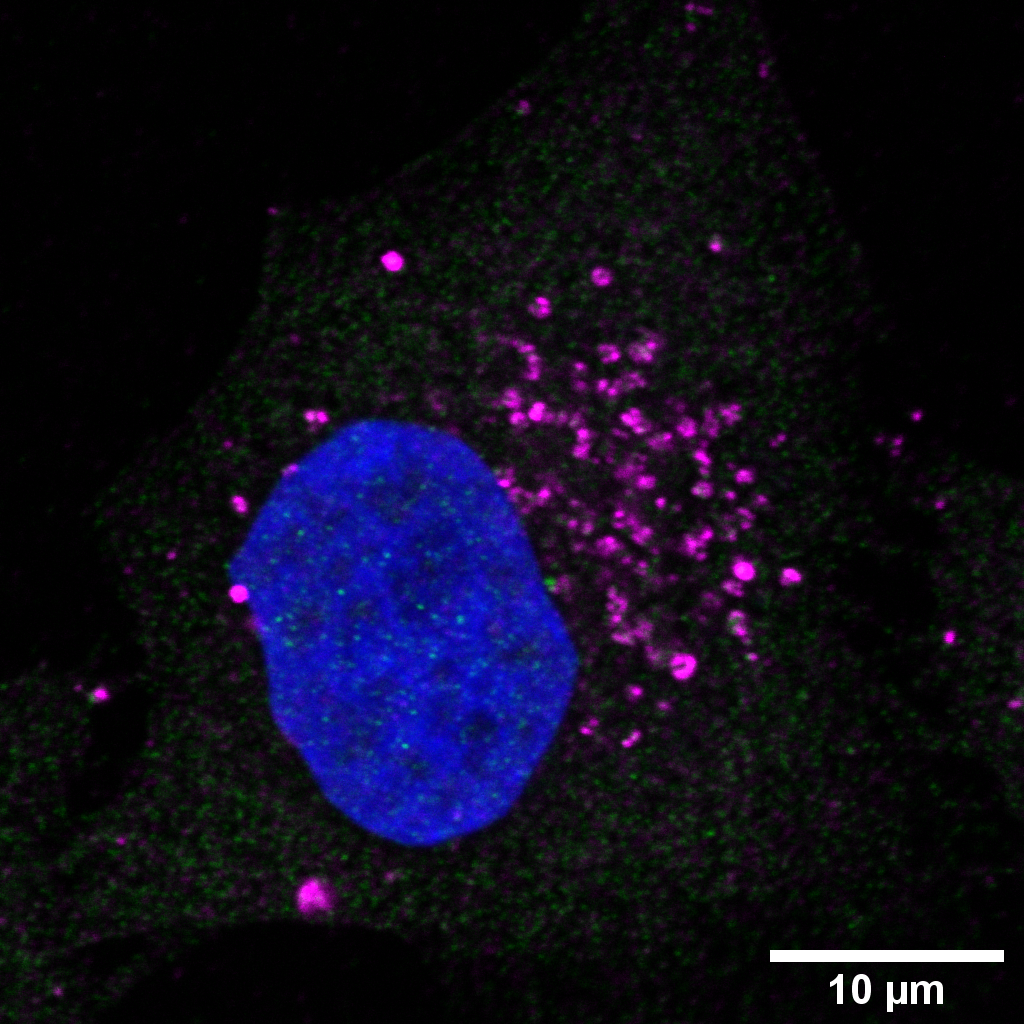

Supplement: Supplementary file 6 — Source data Fig. 4 [file 44318_2025_672_MOESM6_ESM.zip › Figure 4/4A/WT_LLOMe_scale.tif]

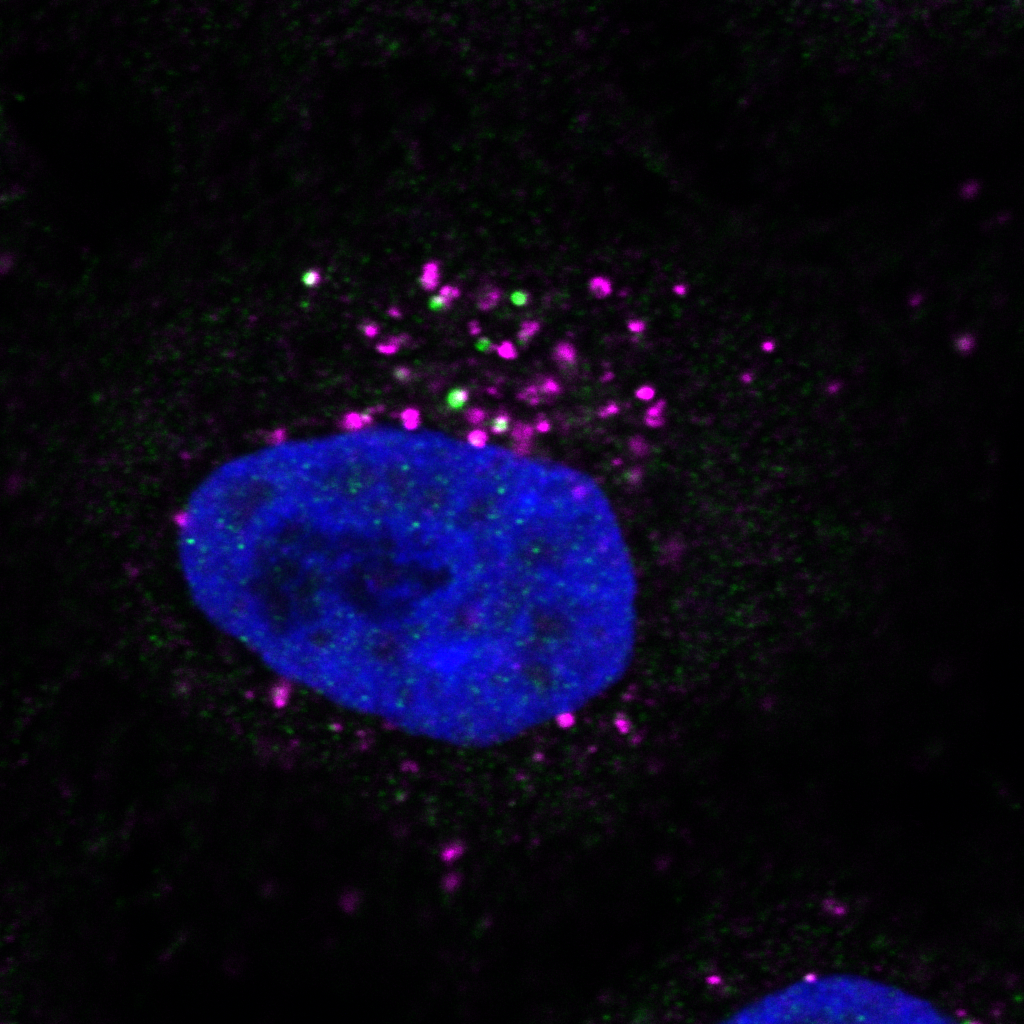

Supplement: Supplementary file 6 — Source data Fig. 4 [file 44318_2025_672_MOESM6_ESM.zip › Figure 4/4A/WT_LLOMe_Wash.tif]

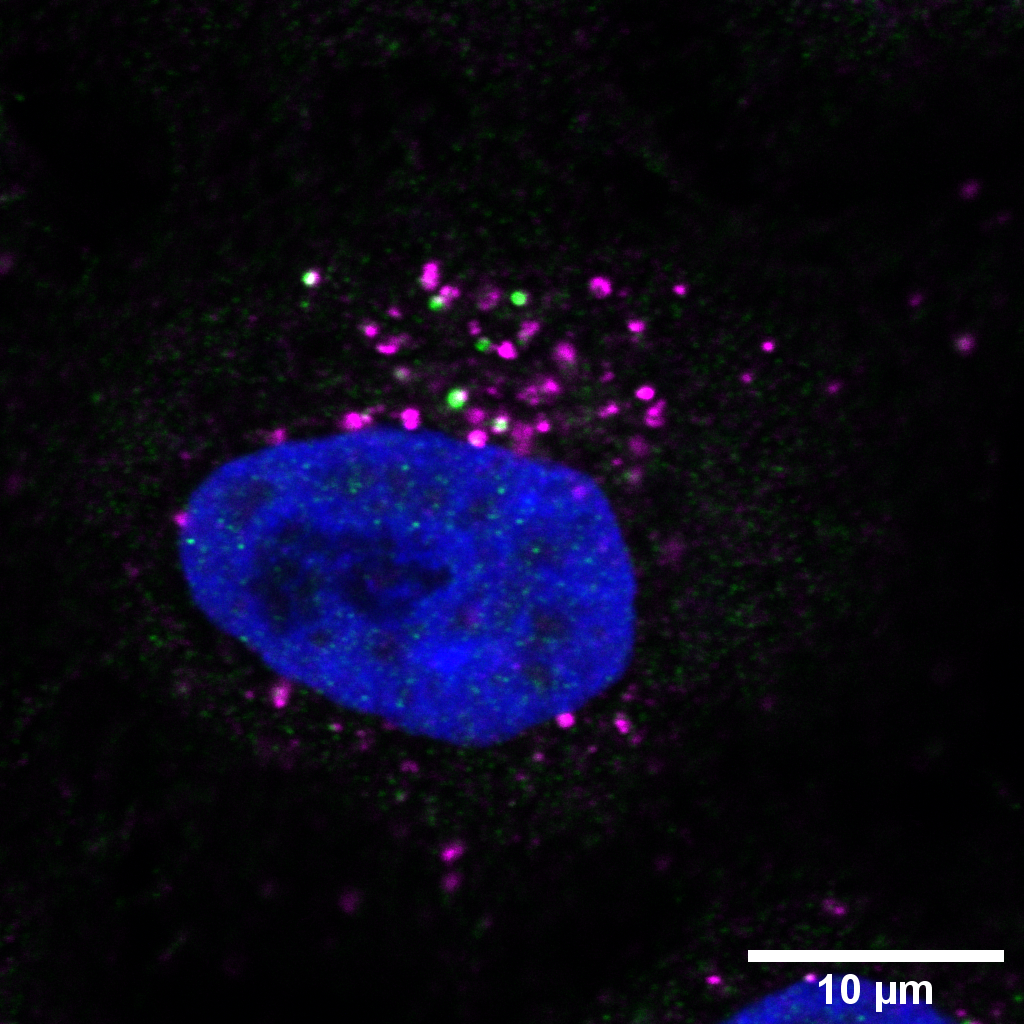

Supplement: Supplementary file 6 — Source data Fig. 4 [file 44318_2025_672_MOESM6_ESM.zip › Figure 4/4A/WT_LLOMe_Wash_scale.tif]

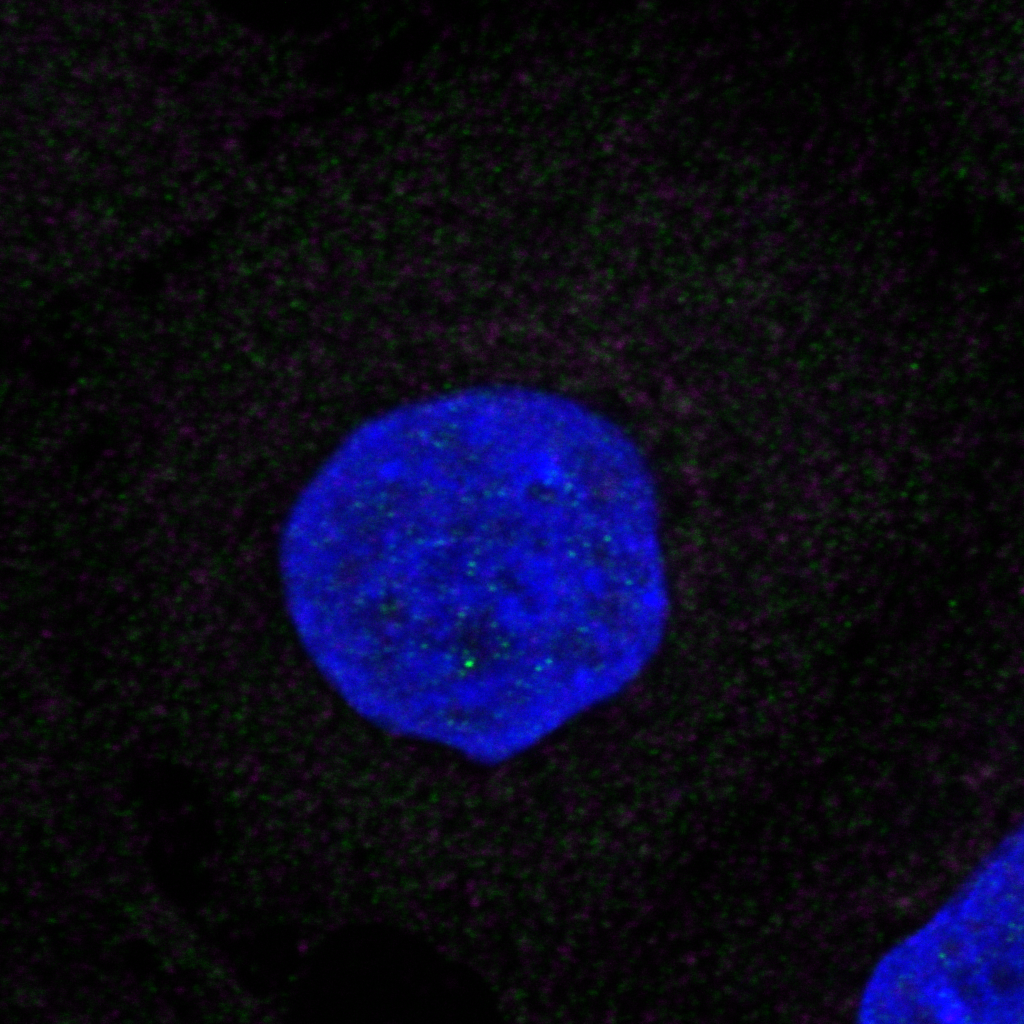

Supplement: Supplementary file 6 — Source data Fig. 4 [file 44318_2025_672_MOESM6_ESM.zip › Figure 4/4A/WT_VEH.tif]

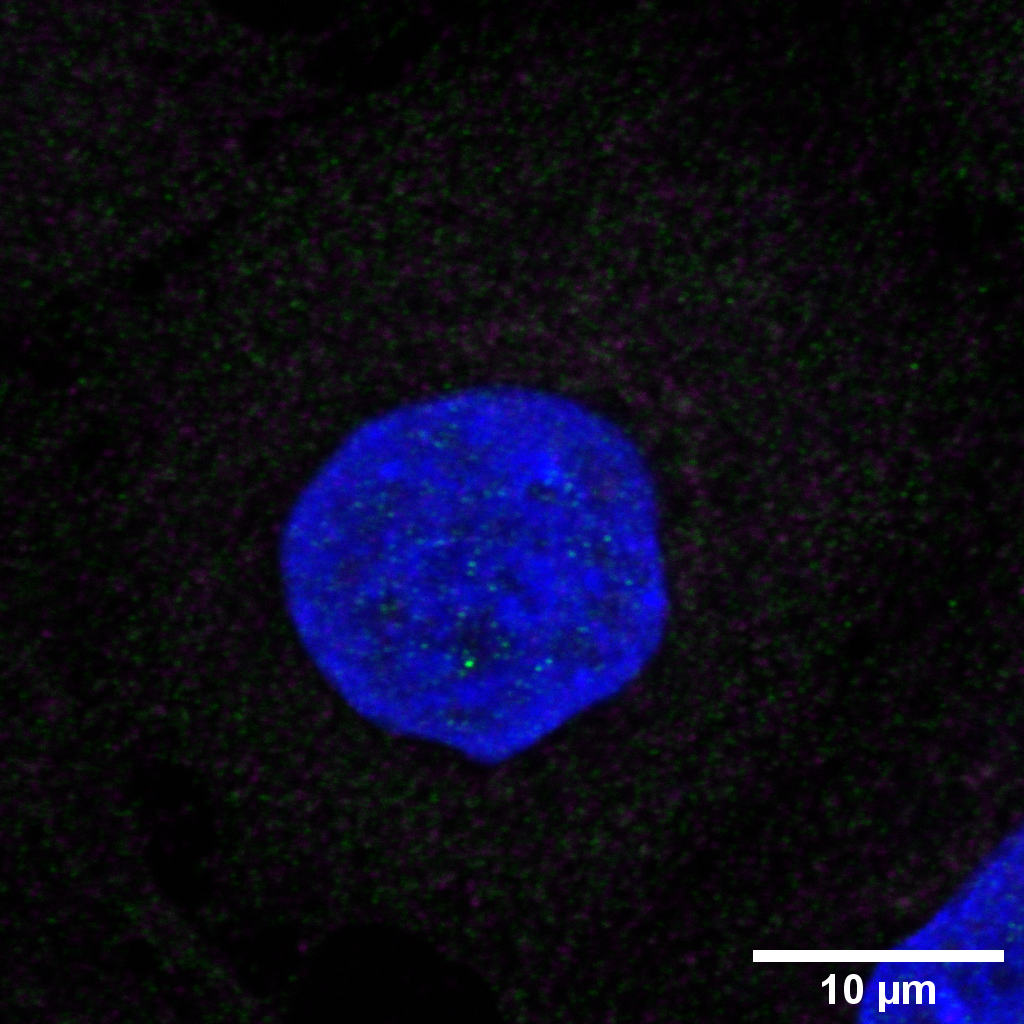

Supplement: Supplementary file 6 — Source data Fig. 4 [file 44318_2025_672_MOESM6_ESM.zip › Figure 4/4A/WT_VEH_scale.tif]

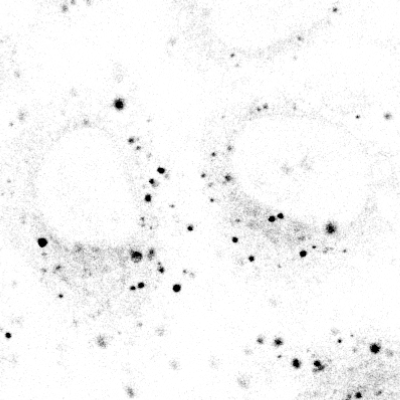

Supplement: Supplementary file 6 — Source data Fig. 4 [file 44318_2025_672_MOESM6_ESM.zip › Figure 4/4C/WT_45min.tif]

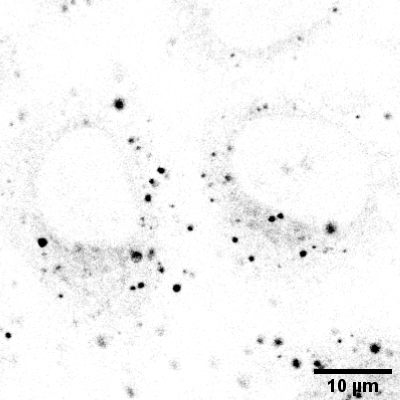

Supplement: Supplementary file 6 — Source data Fig. 4 [file 44318_2025_672_MOESM6_ESM.zip › Figure 4/4C/WT_45min_scale.tif]

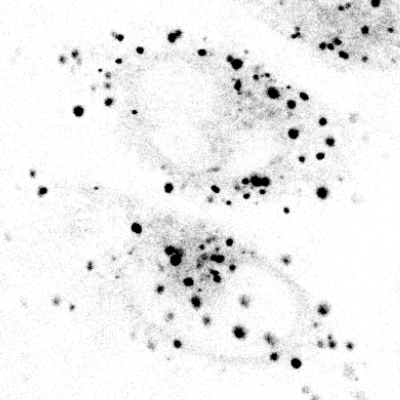

Supplement: Supplementary file 6 — Source data Fig. 4 [file 44318_2025_672_MOESM6_ESM.zip › Figure 4/4C/WT_90min.tif]

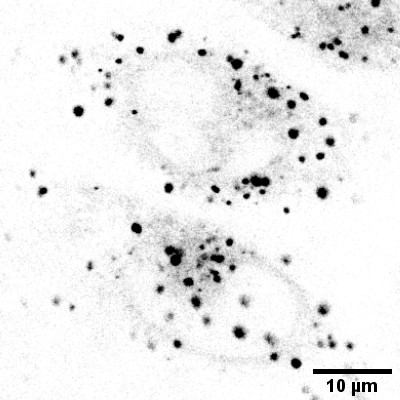

Supplement: Supplementary file 6 — Source data Fig. 4 [file 44318_2025_672_MOESM6_ESM.zip › Figure 4/4C/WT_90min_scale.tif]

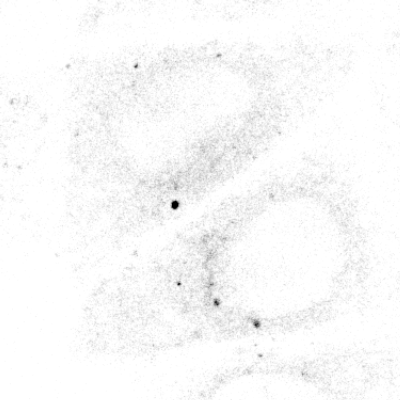

Supplement: Supplementary file 6 — Source data Fig. 4 [file 44318_2025_672_MOESM6_ESM.zip › Figure 4/4C/WT_LLOMe.tif]

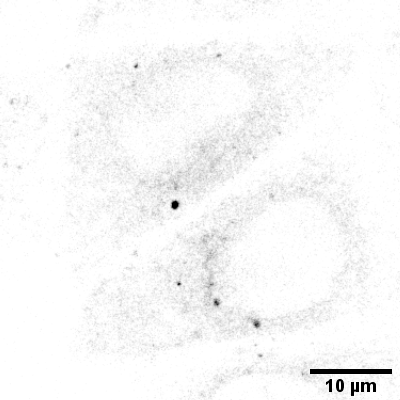

Supplement: Supplementary file 6 — Source data Fig. 4 [file 44318_2025_672_MOESM6_ESM.zip › Figure 4/4C/WT_LLOMe_scale.tif]

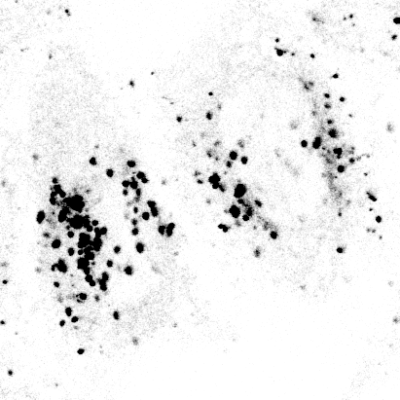

Supplement: Supplementary file 6 — Source data Fig. 4 [file 44318_2025_672_MOESM6_ESM.zip › Figure 4/4C/WT_UNT.tif]

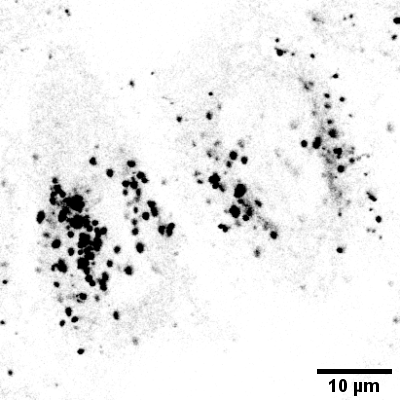

Supplement: Supplementary file 6 — Source data Fig. 4 [file 44318_2025_672_MOESM6_ESM.zip › Figure 4/4C/WT_UNT_scale.tif]

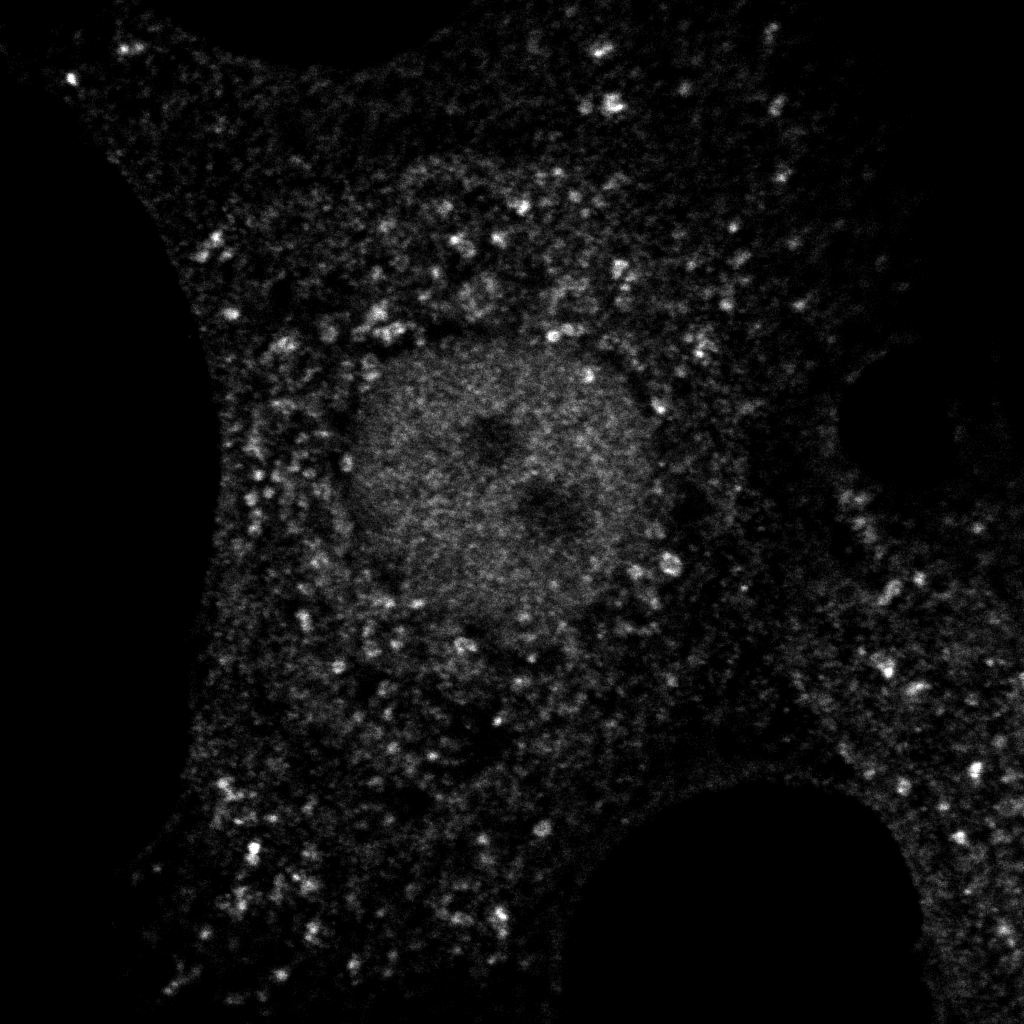

Supplement: Supplementary file 7 — Source data Fig. 5 [file 44318_2025_672_MOESM7_ESM.zip › Figure 5/5A/16KO_ALG2.tif]

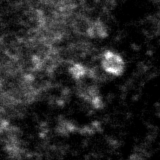

Supplement: Supplementary file 7 — Source data Fig. 5 [file 44318_2025_672_MOESM7_ESM.zip › Figure 5/5A/16KO_ALG2_zoom.tif]

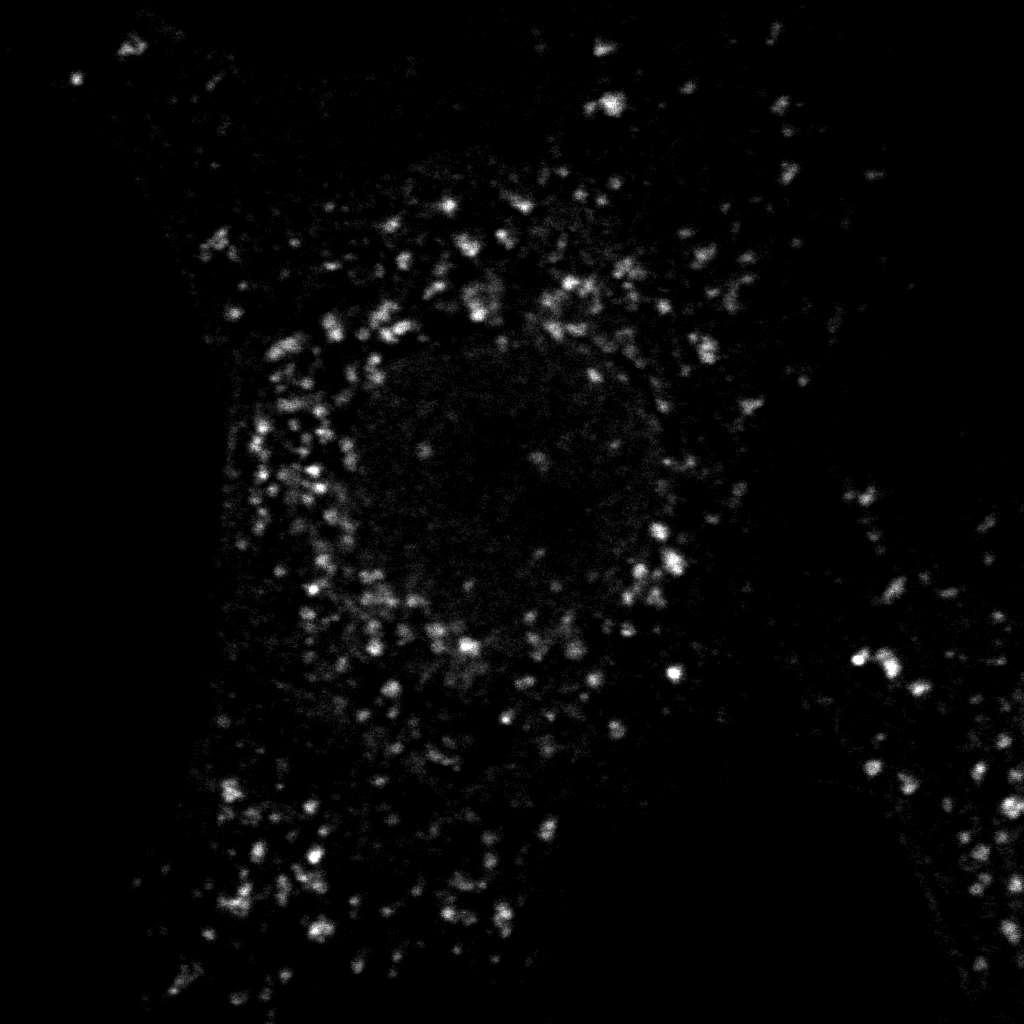

Supplement: Supplementary file 7 — Source data Fig. 5 [file 44318_2025_672_MOESM7_ESM.zip › Figure 5/5A/16KO_LAMP.tif]

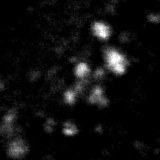

Supplement: Supplementary file 7 — Source data Fig. 5 [file 44318_2025_672_MOESM7_ESM.zip › Figure 5/5A/16KO_LAMP_zoom.tif]

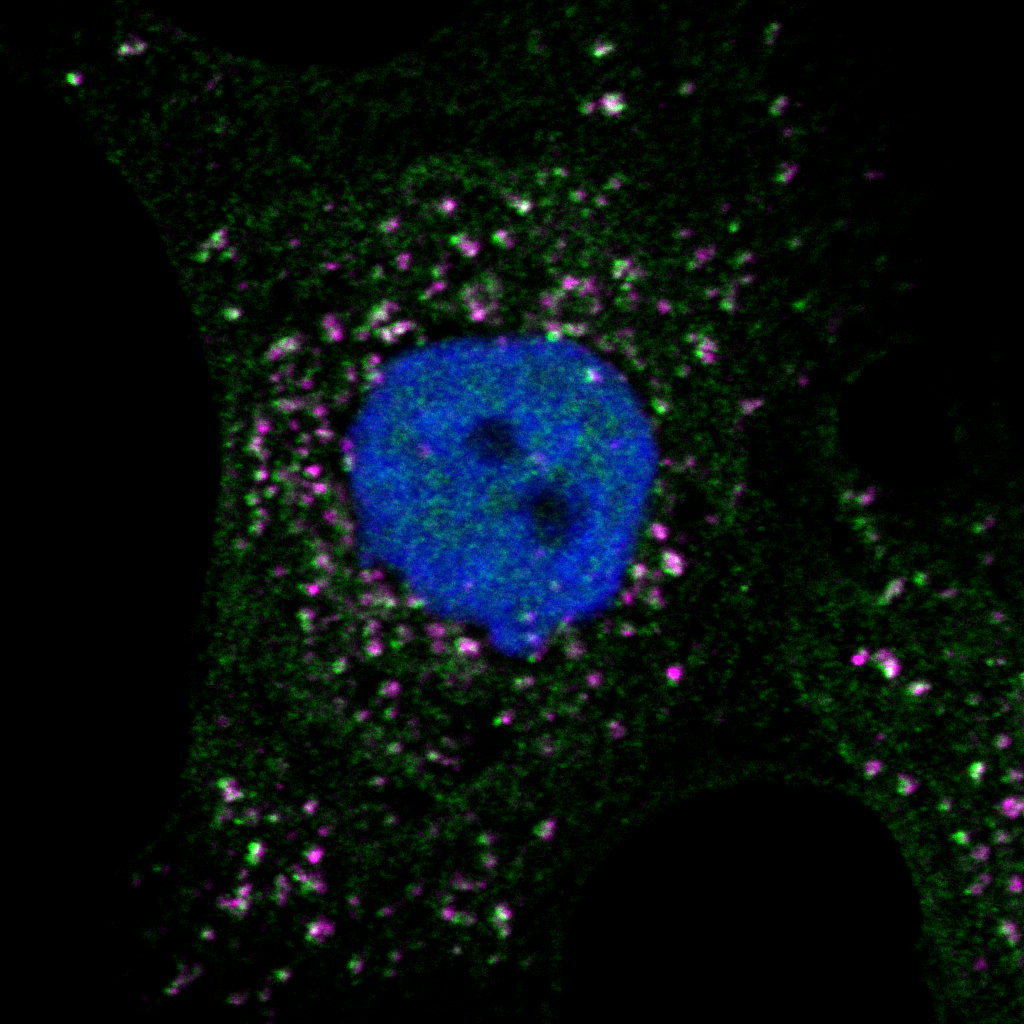

Supplement: Supplementary file 7 — Source data Fig. 5 [file 44318_2025_672_MOESM7_ESM.zip › Figure 5/5A/16KO_merge.tif]

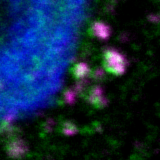

Supplement: Supplementary file 7 — Source data Fig. 5 [file 44318_2025_672_MOESM7_ESM.zip › Figure 5/5A/16KO_merge_zoom.tif]

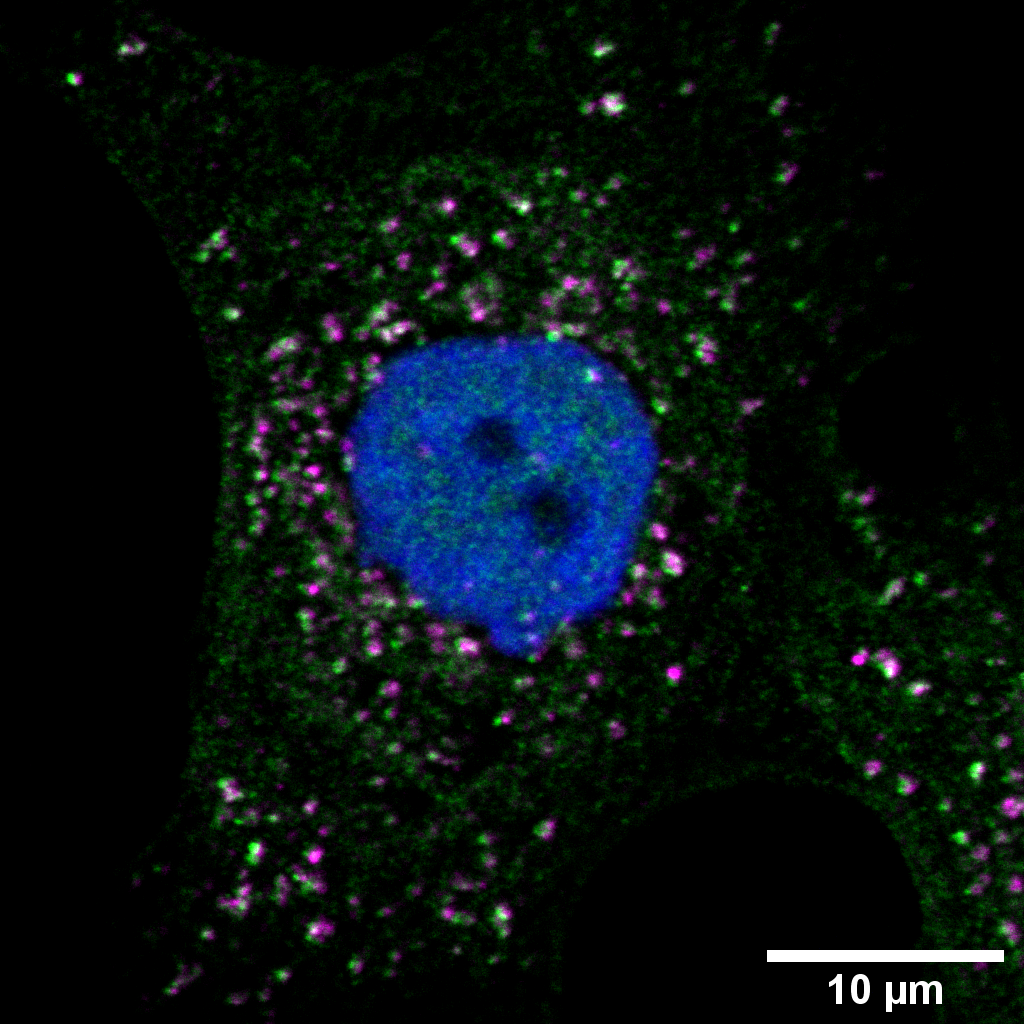

Supplement: Supplementary file 7 — Source data Fig. 5 [file 44318_2025_672_MOESM7_ESM.zip › Figure 5/5A/16KO_scale.tif]

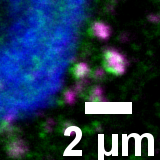

Supplement: Supplementary file 7 — Source data Fig. 5 [file 44318_2025_672_MOESM7_ESM.zip › Figure 5/5A/16KO_scale_zoom.tif]

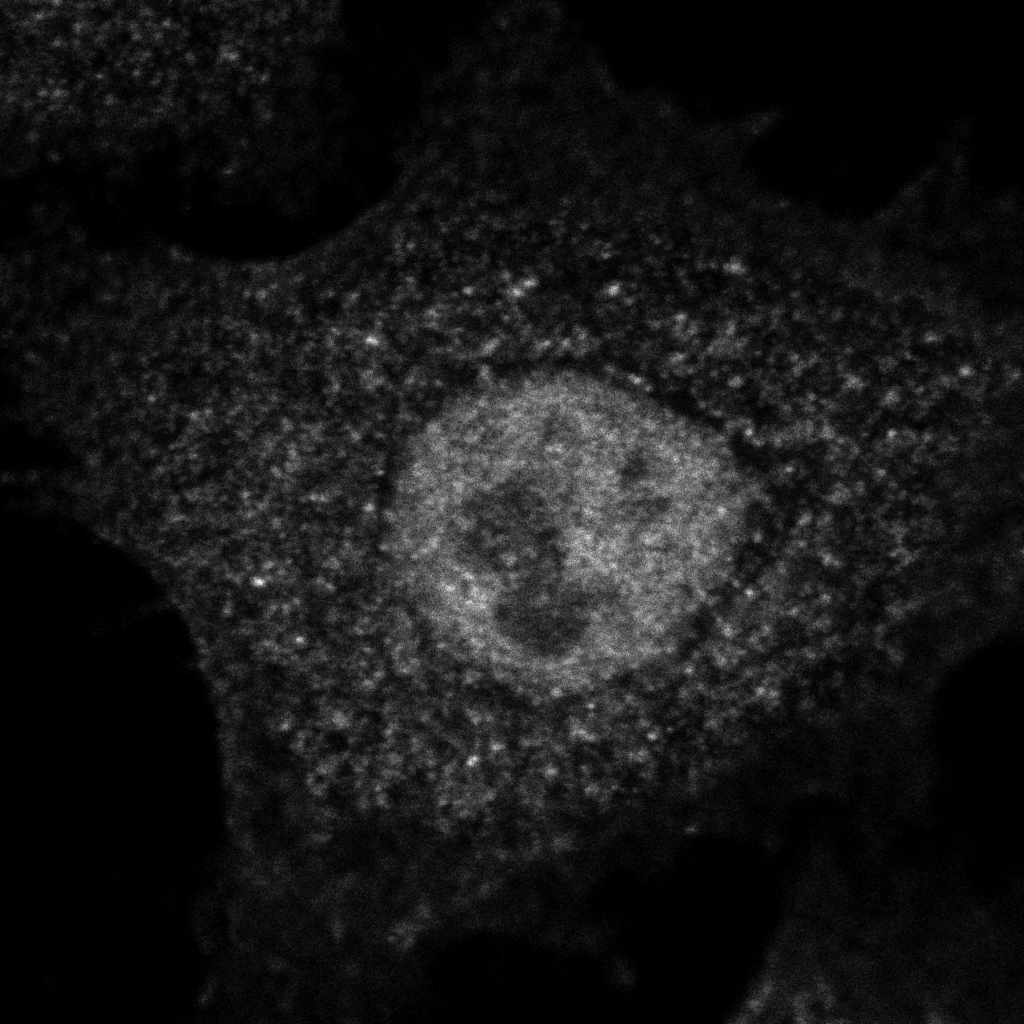

Supplement: Supplementary file 7 — Source data Fig. 5 [file 44318_2025_672_MOESM7_ESM.zip › Figure 5/5A/5KO_ALG2.tif]

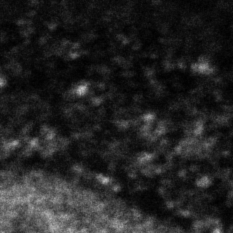

Supplement: Supplementary file 7 — Source data Fig. 5 [file 44318_2025_672_MOESM7_ESM.zip › Figure 5/5A/5KO_ALG2_zoom.tif]

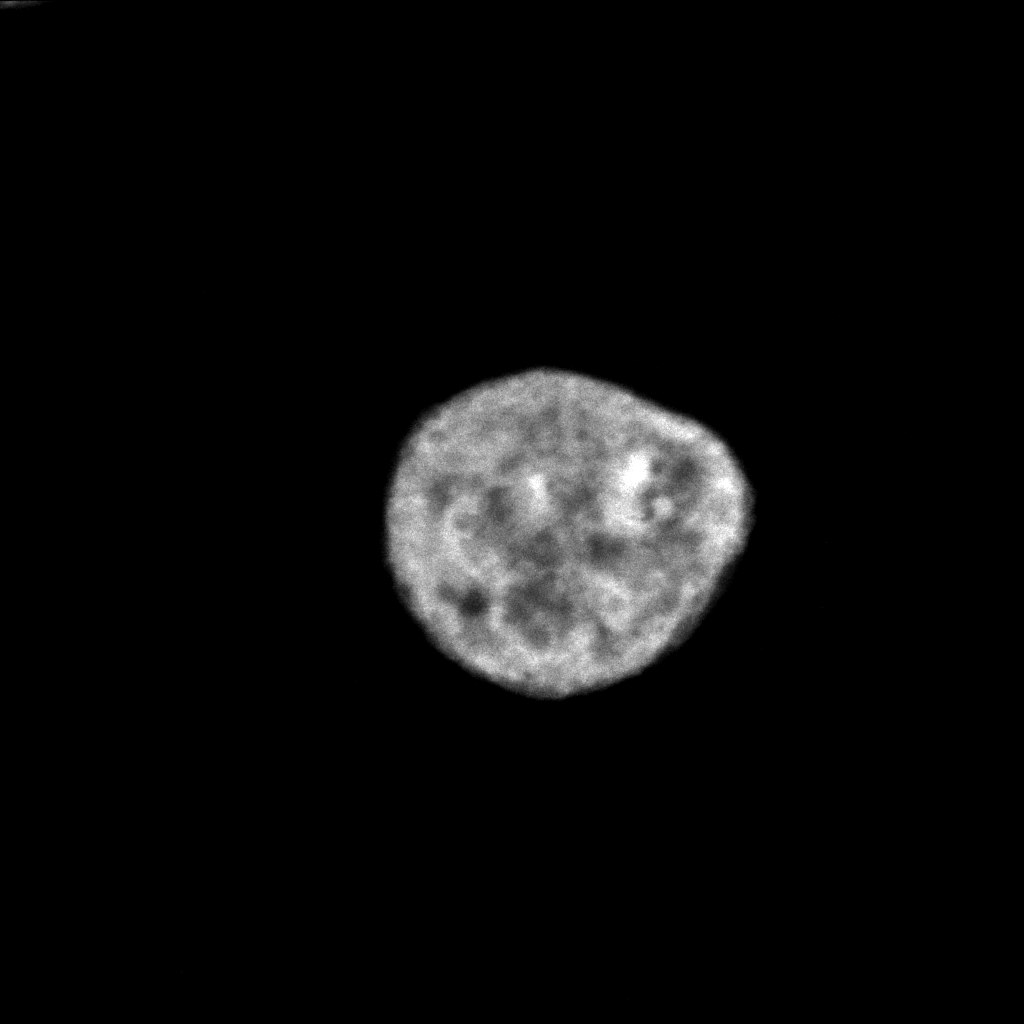

Supplement: Supplementary file 7 — Source data Fig. 5 [file 44318_2025_672_MOESM7_ESM.zip › Figure 5/5A/5KO_DAPI.tif]

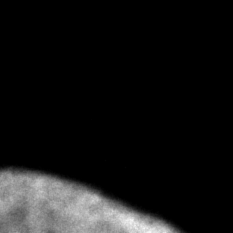

Supplement: Supplementary file 7 — Source data Fig. 5 [file 44318_2025_672_MOESM7_ESM.zip › Figure 5/5A/5KO_DAPI_zoom.tif]

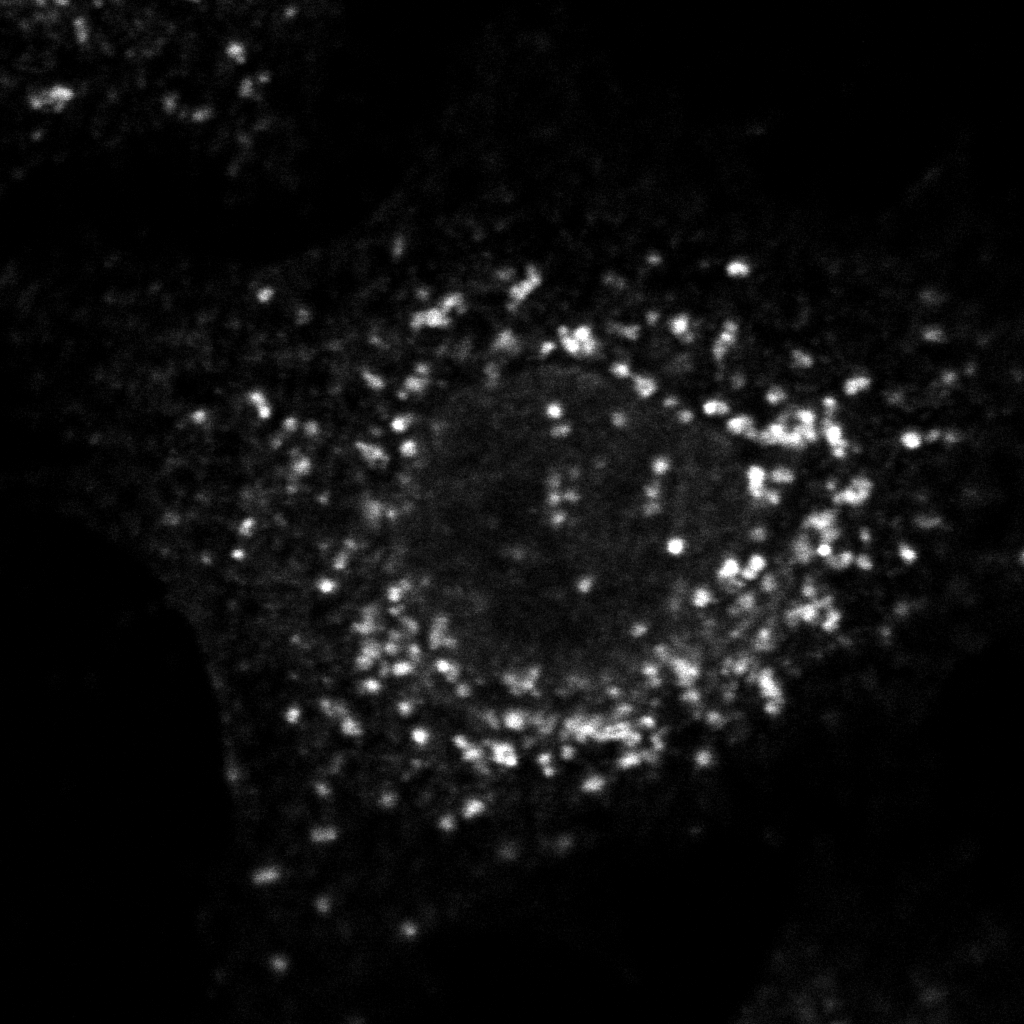

Supplement: Supplementary file 7 — Source data Fig. 5 [file 44318_2025_672_MOESM7_ESM.zip › Figure 5/5A/5KO_LAMP.tif]

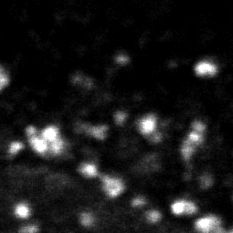

Supplement: Supplementary file 7 — Source data Fig. 5 [file 44318_2025_672_MOESM7_ESM.zip › Figure 5/5A/5KO_LAMP_zoom.tif]

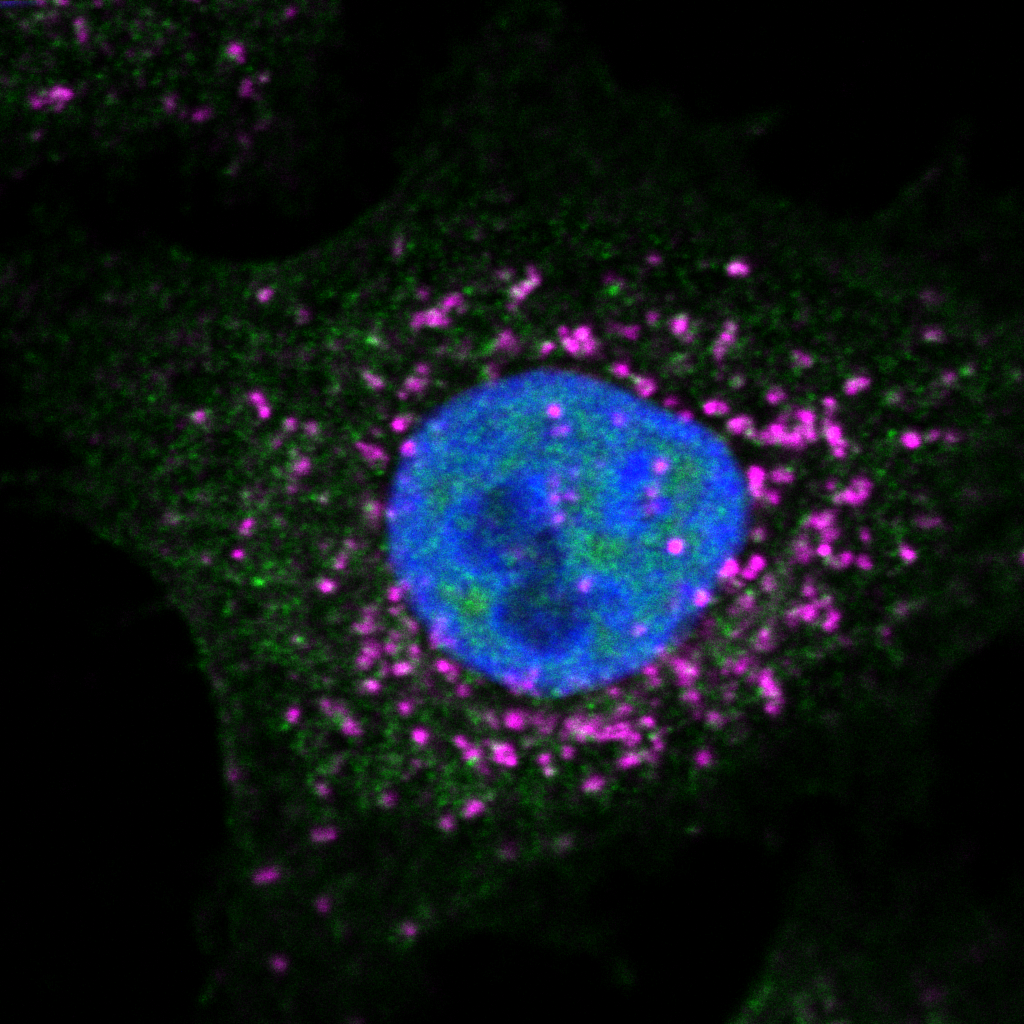

Supplement: Supplementary file 7 — Source data Fig. 5 [file 44318_2025_672_MOESM7_ESM.zip › Figure 5/5A/5KO_merge.tif]

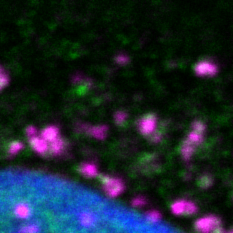

Supplement: Supplementary file 7 — Source data Fig. 5 [file 44318_2025_672_MOESM7_ESM.zip › Figure 5/5A/5KO_merge_zoom.tif]

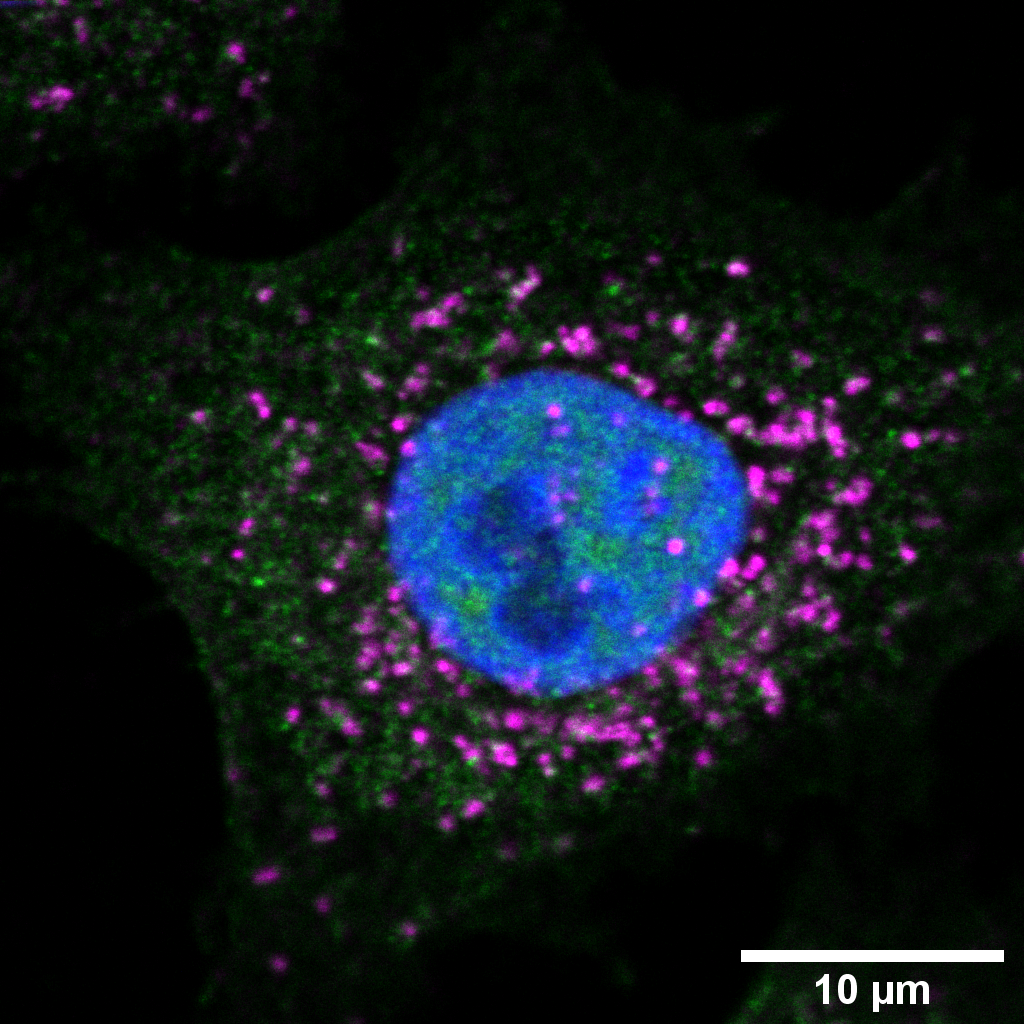

Supplement: Supplementary file 7 — Source data Fig. 5 [file 44318_2025_672_MOESM7_ESM.zip › Figure 5/5A/5KO_scale.tif]

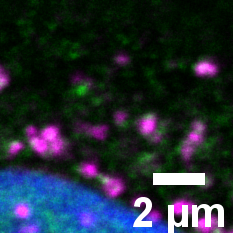

Supplement: Supplementary file 7 — Source data Fig. 5 [file 44318_2025_672_MOESM7_ESM.zip › Figure 5/5A/5KO_scale_zoom.tif]

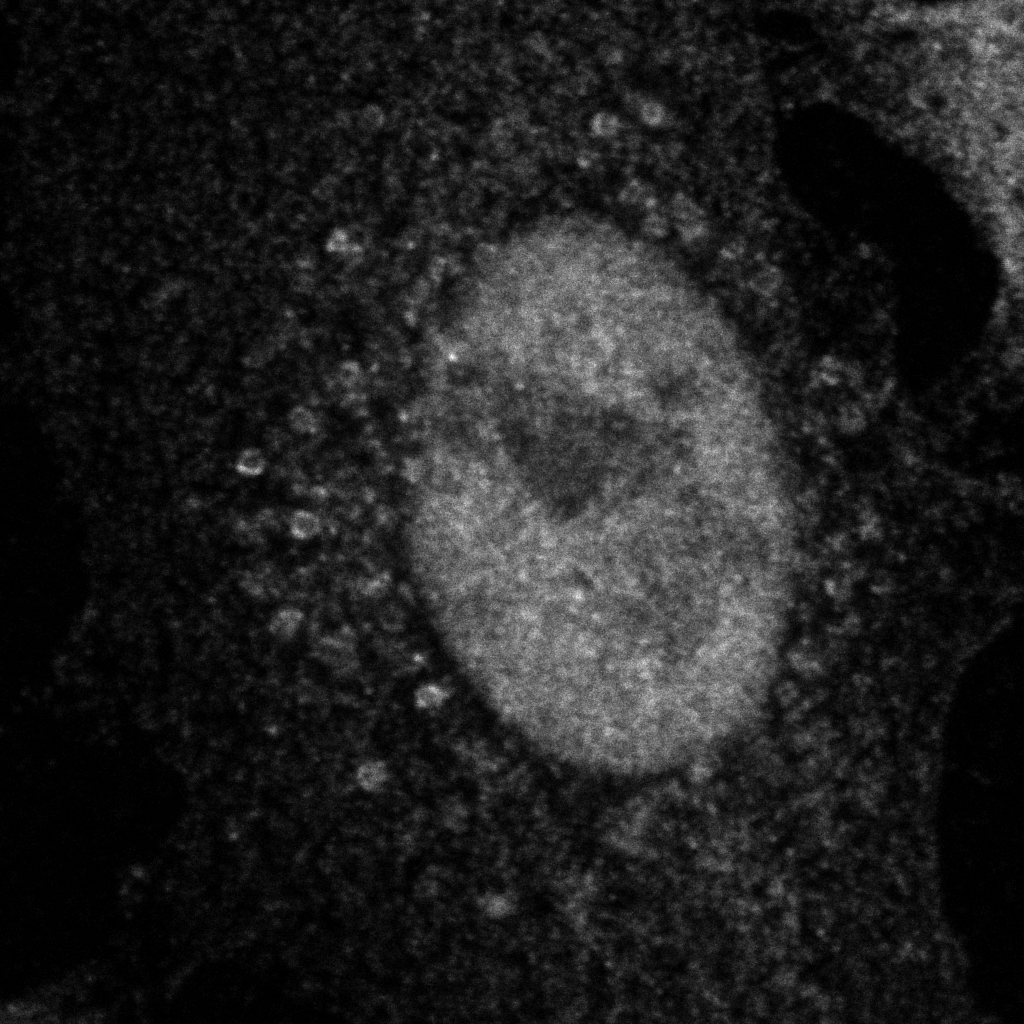

Supplement: Supplementary file 7 — Source data Fig. 5 [file 44318_2025_672_MOESM7_ESM.zip › Figure 5/5A/8KO_ALG2.tif]

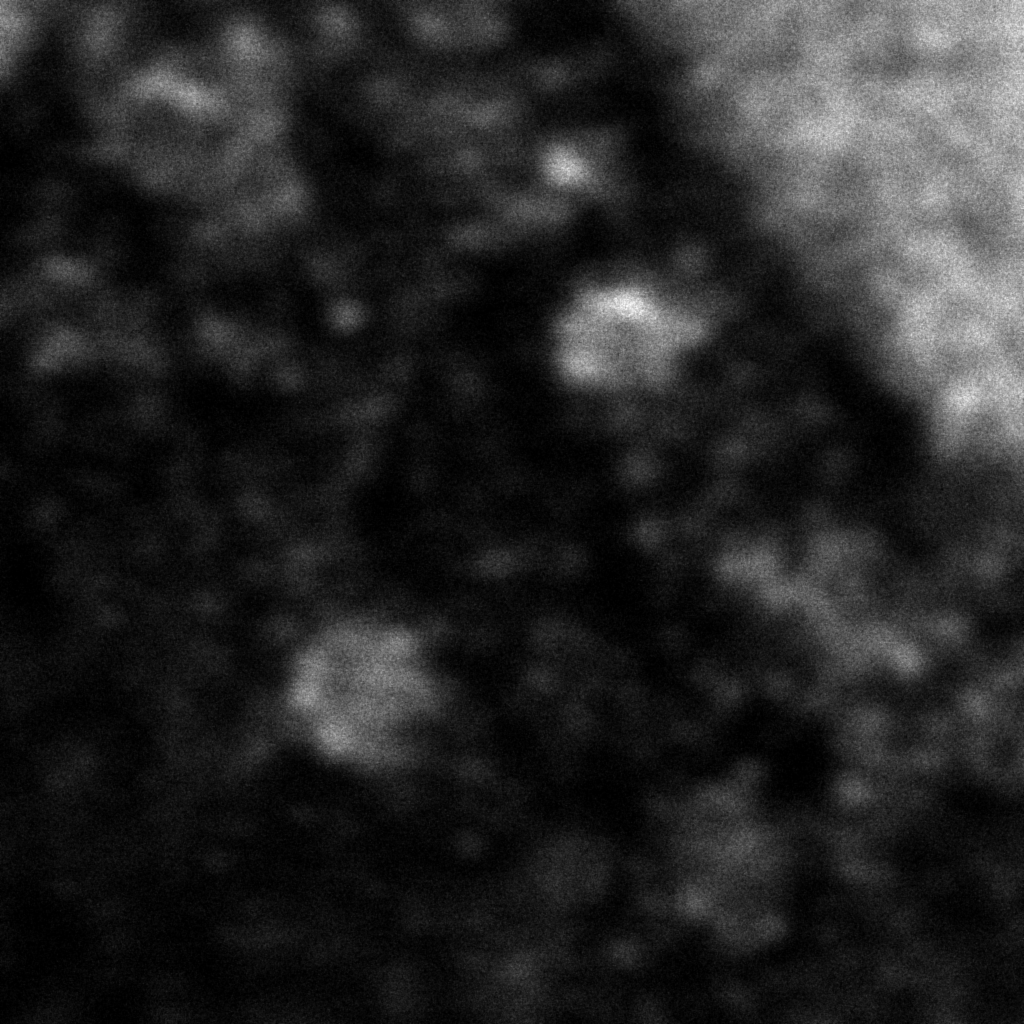

Supplement: Supplementary file 7 — Source data Fig. 5 [file 44318_2025_672_MOESM7_ESM.zip › Figure 5/5A/8KO_ALG2_zoom.tif]

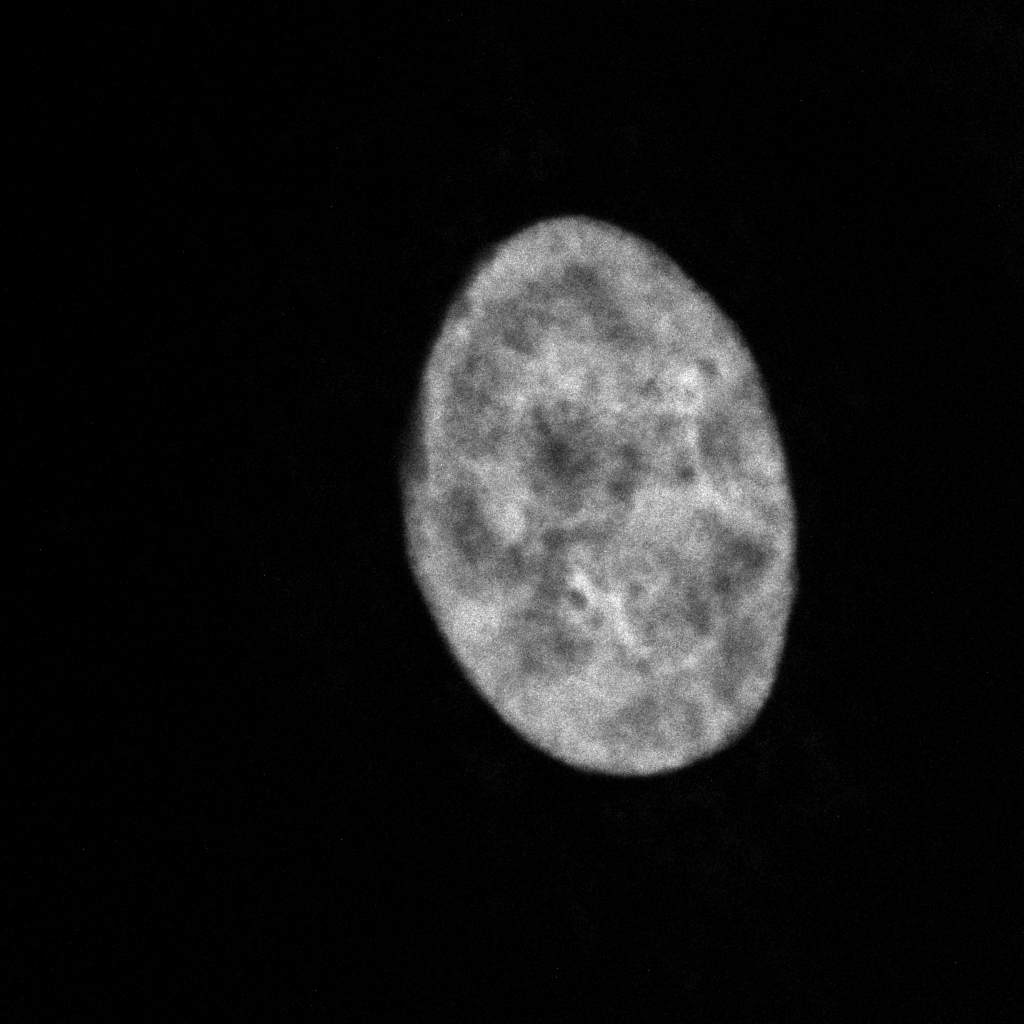

Supplement: Supplementary file 7 — Source data Fig. 5 [file 44318_2025_672_MOESM7_ESM.zip › Figure 5/5A/8KO_dapi.tif]

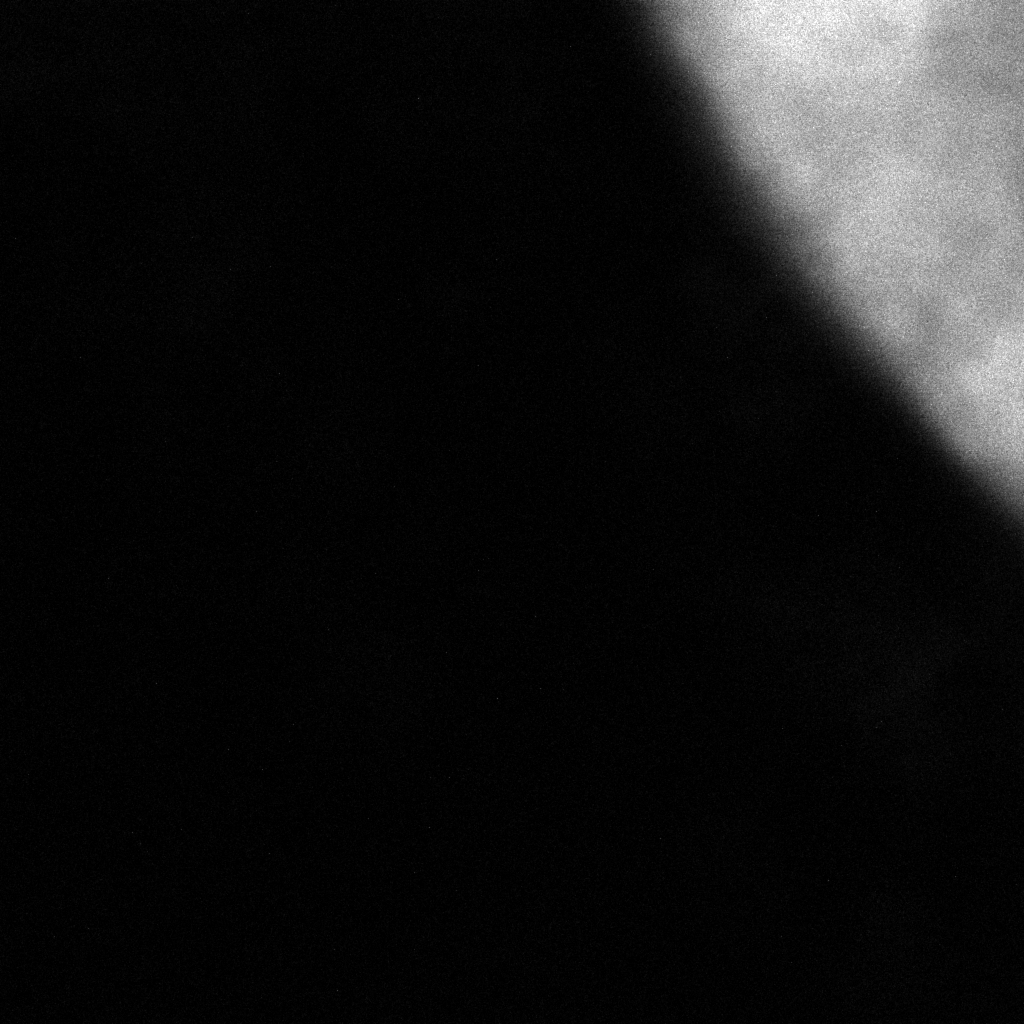

Supplement: Supplementary file 7 — Source data Fig. 5 [file 44318_2025_672_MOESM7_ESM.zip › Figure 5/5A/8KO_dapi_zoom.tif]

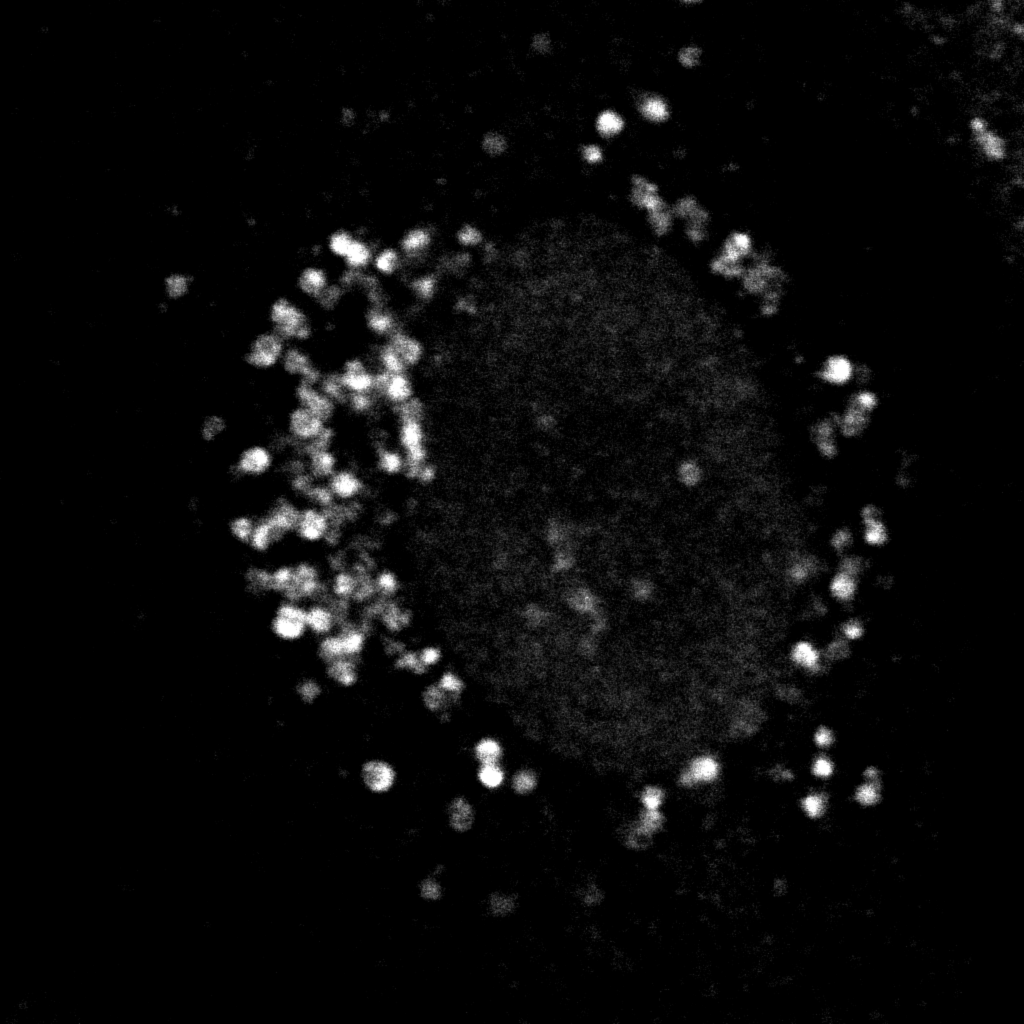

Supplement: Supplementary file 7 — Source data Fig. 5 [file 44318_2025_672_MOESM7_ESM.zip › Figure 5/5A/8KO_LAMP.tif]

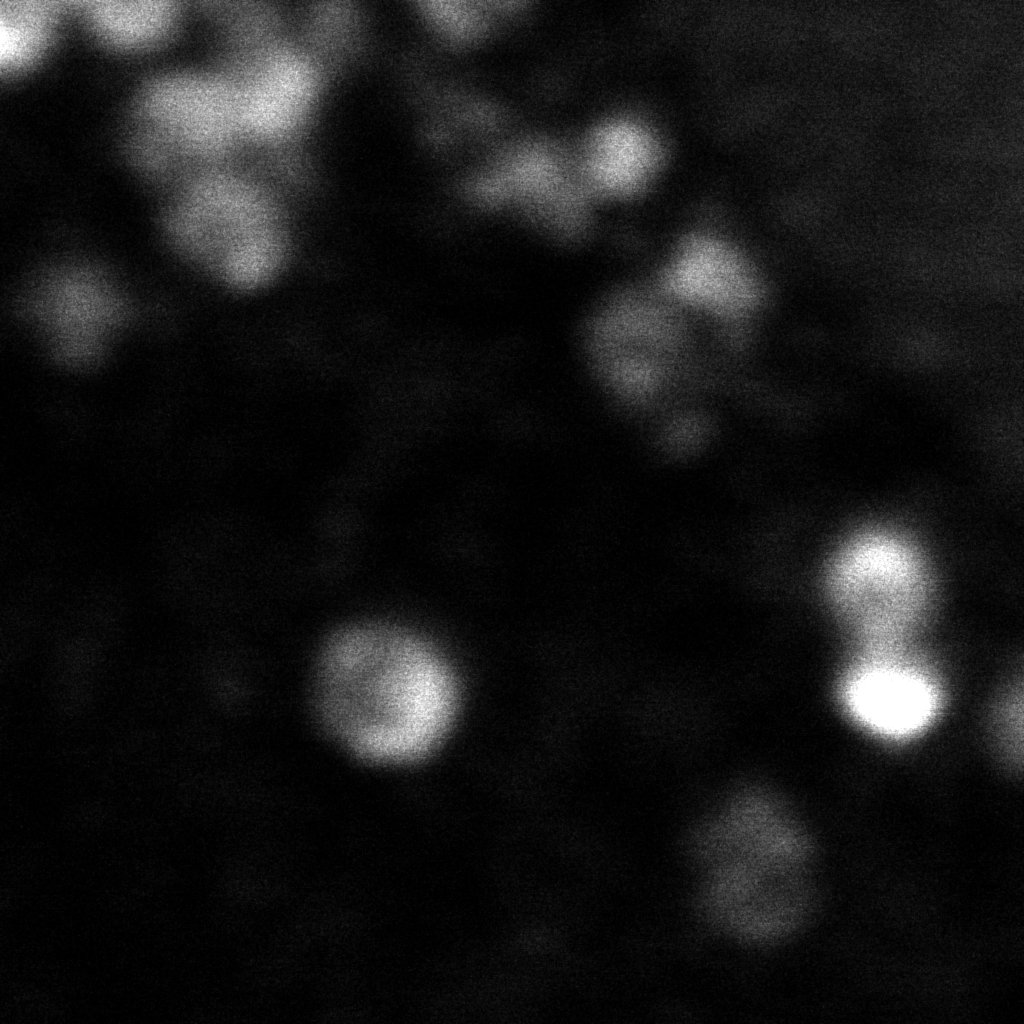

Supplement: Supplementary file 7 — Source data Fig. 5 [file 44318_2025_672_MOESM7_ESM.zip › Figure 5/5A/8KO_LAMP_zoom.tif]

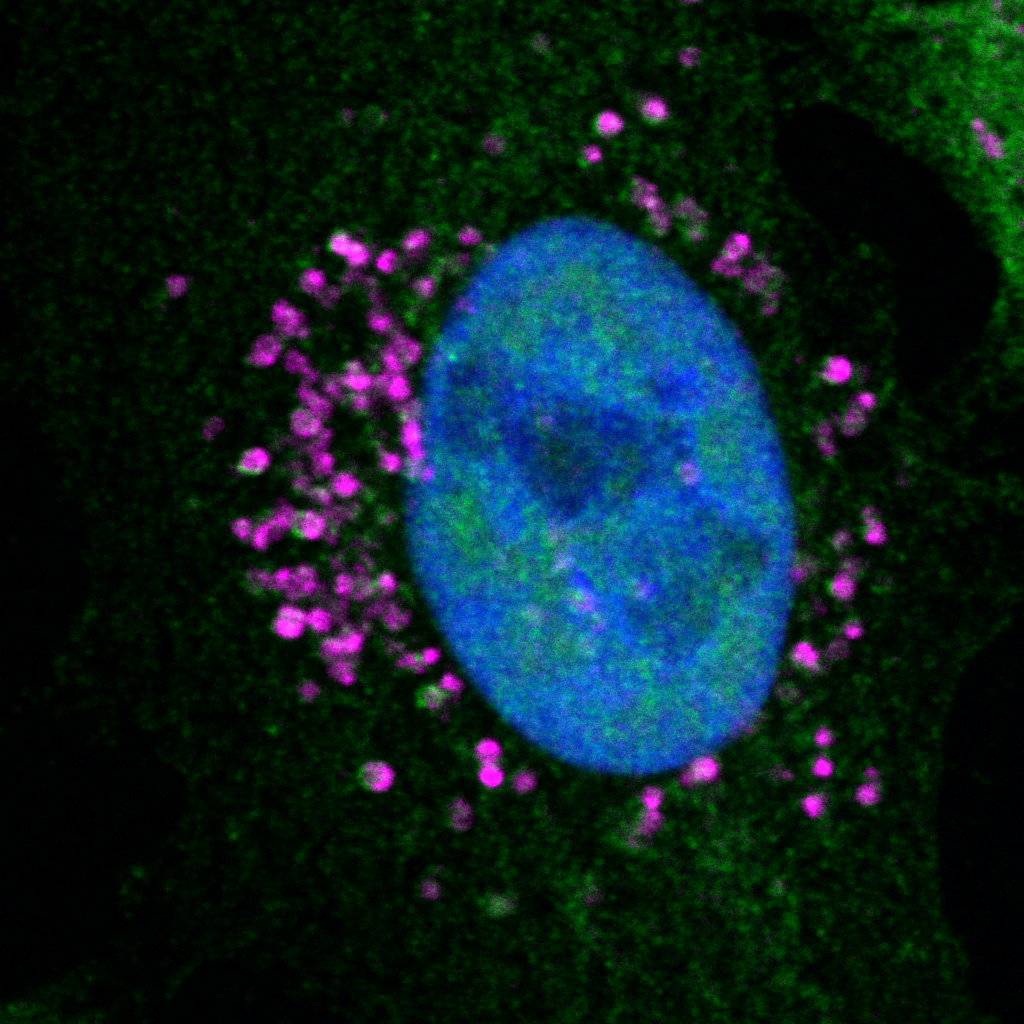

Supplement: Supplementary file 7 — Source data Fig. 5 [file 44318_2025_672_MOESM7_ESM.zip › Figure 5/5A/8KO_merge.tif]

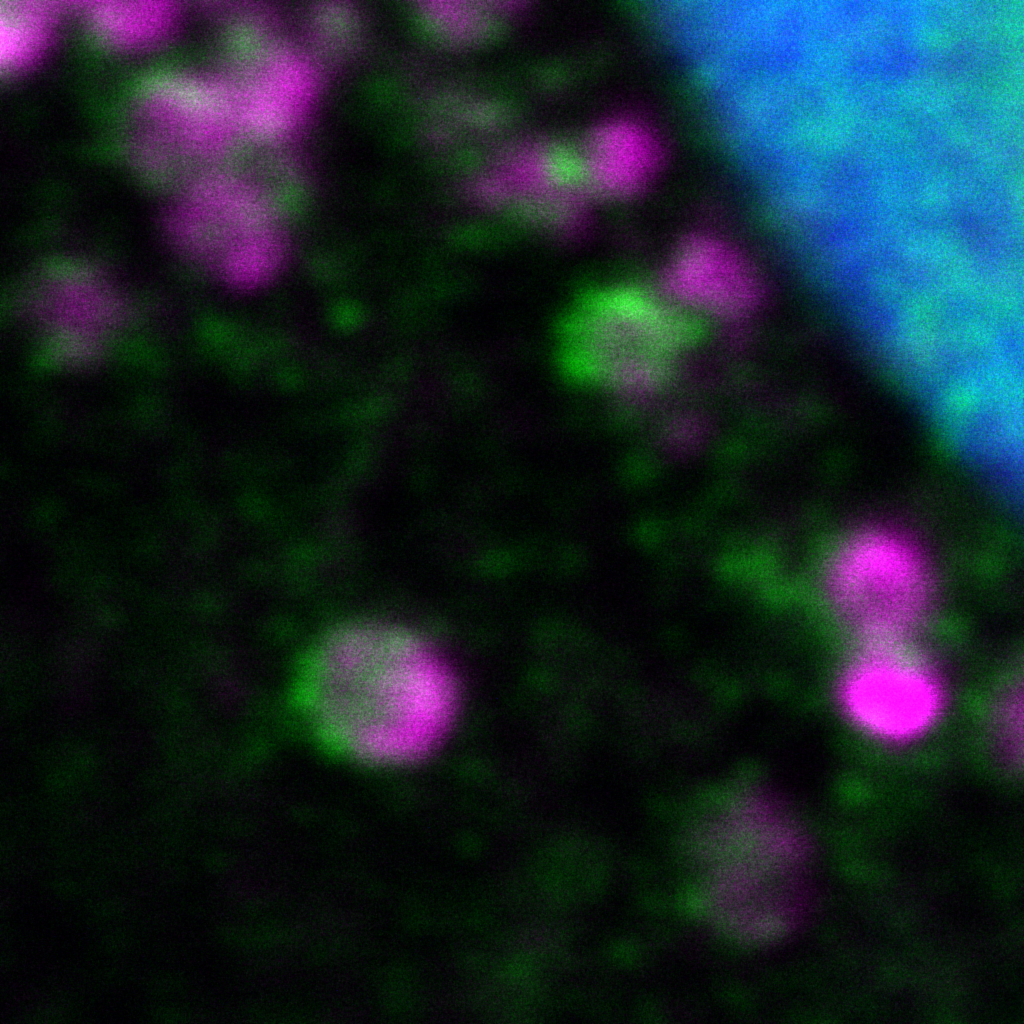

Supplement: Supplementary file 7 — Source data Fig. 5 [file 44318_2025_672_MOESM7_ESM.zip › Figure 5/5A/8KO_merge_zoom.tif]

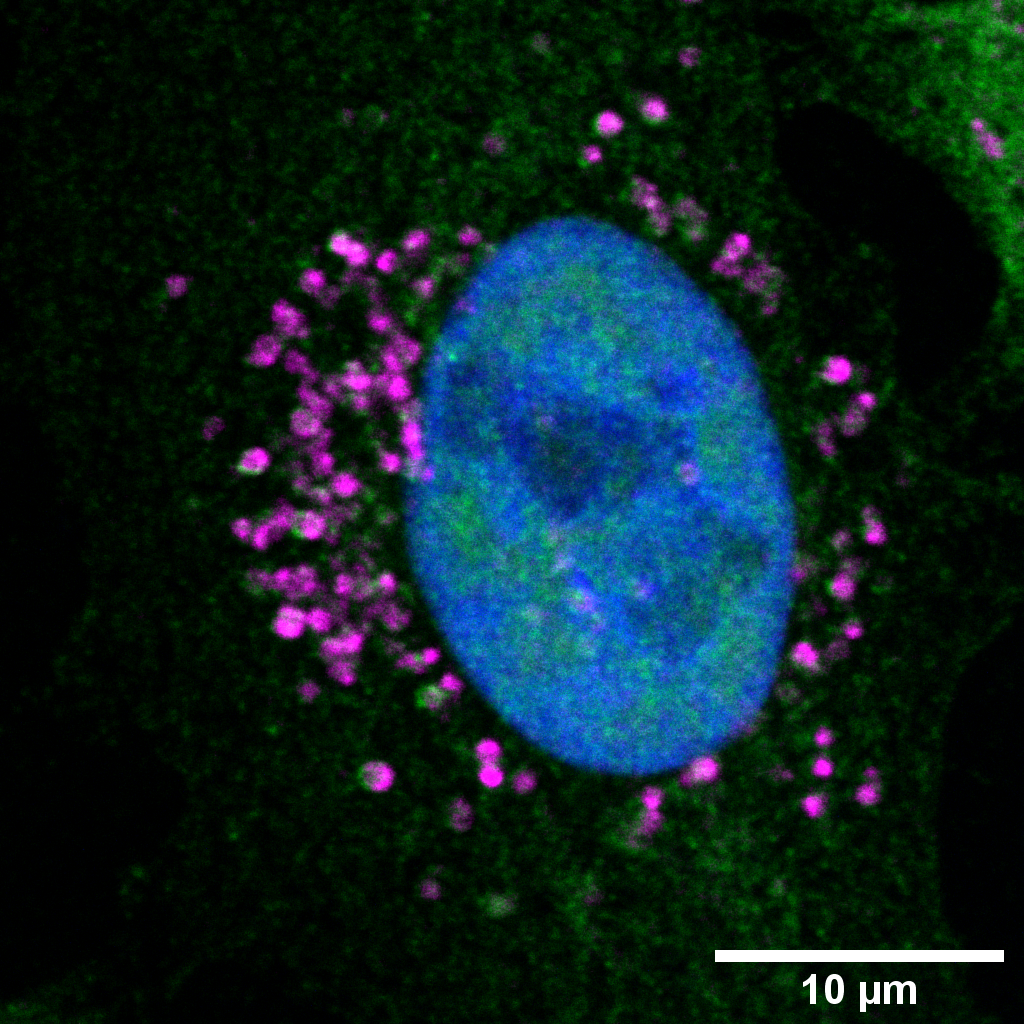

Supplement: Supplementary file 7 — Source data Fig. 5 [file 44318_2025_672_MOESM7_ESM.zip › Figure 5/5A/8KO_scale.tif]

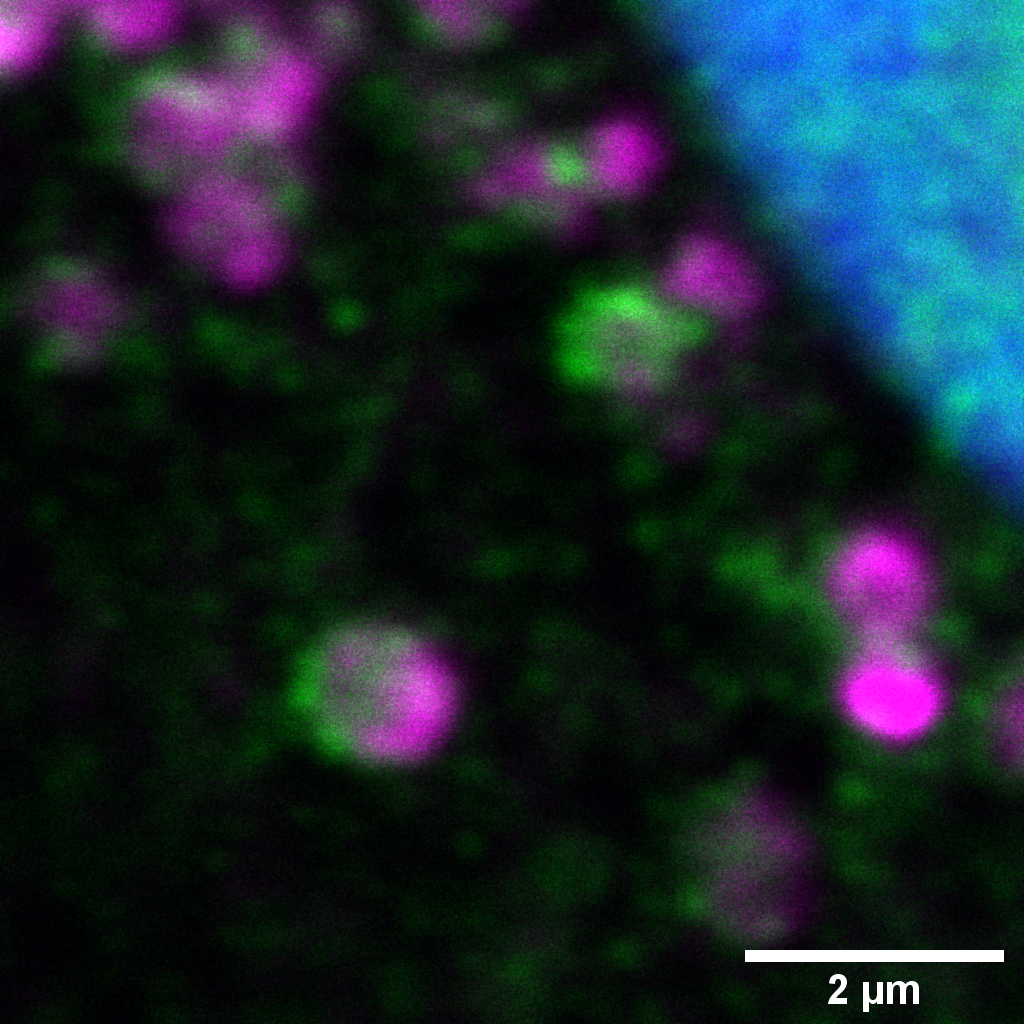

Supplement: Supplementary file 7 — Source data Fig. 5 [file 44318_2025_672_MOESM7_ESM.zip › Figure 5/5A/8KO_scale_zoom.tif]

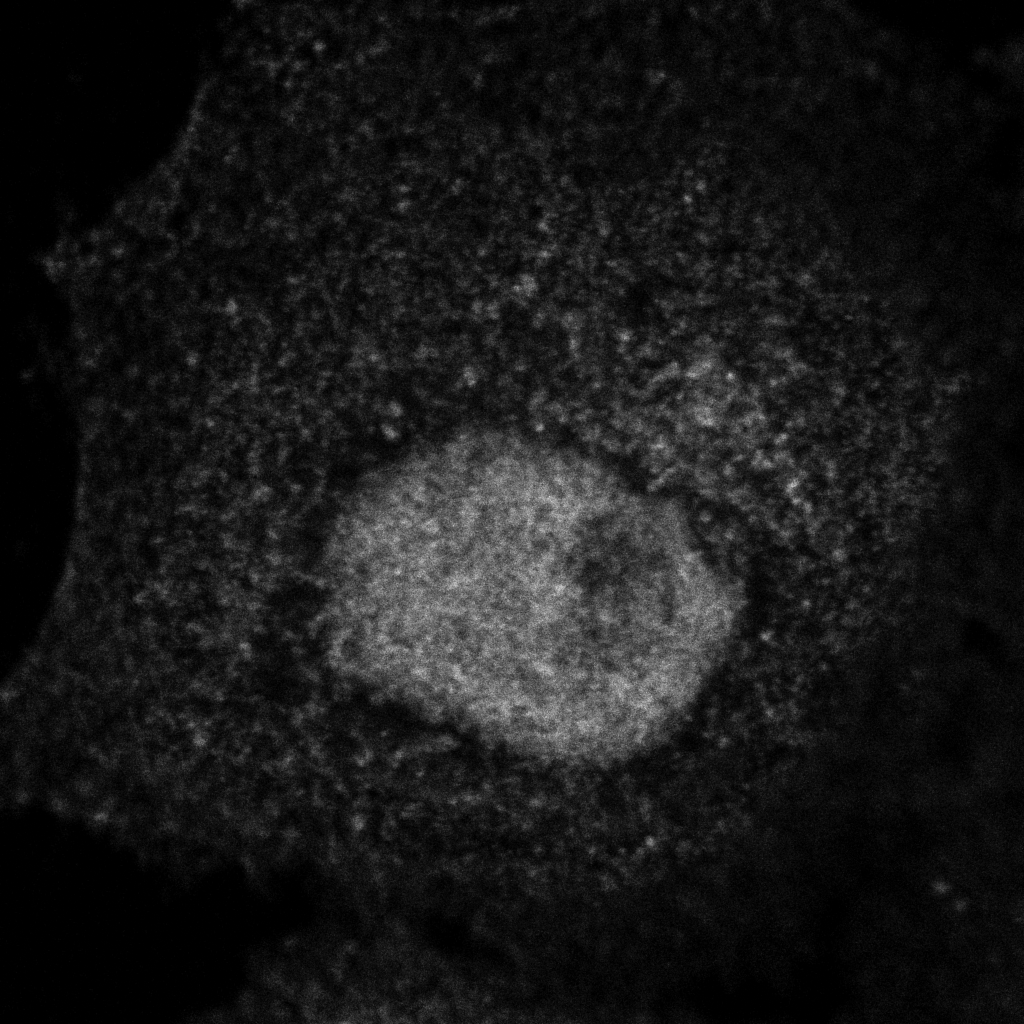

Supplement: Supplementary file 7 — Source data Fig. 5 [file 44318_2025_672_MOESM7_ESM.zip › Figure 5/5A/DKO_ALG2.tif]

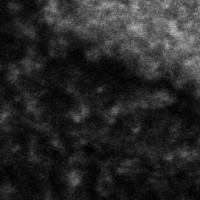

Supplement: Supplementary file 7 — Source data Fig. 5 [file 44318_2025_672_MOESM7_ESM.zip › Figure 5/5A/DKO_ALG2_zoom.tif]

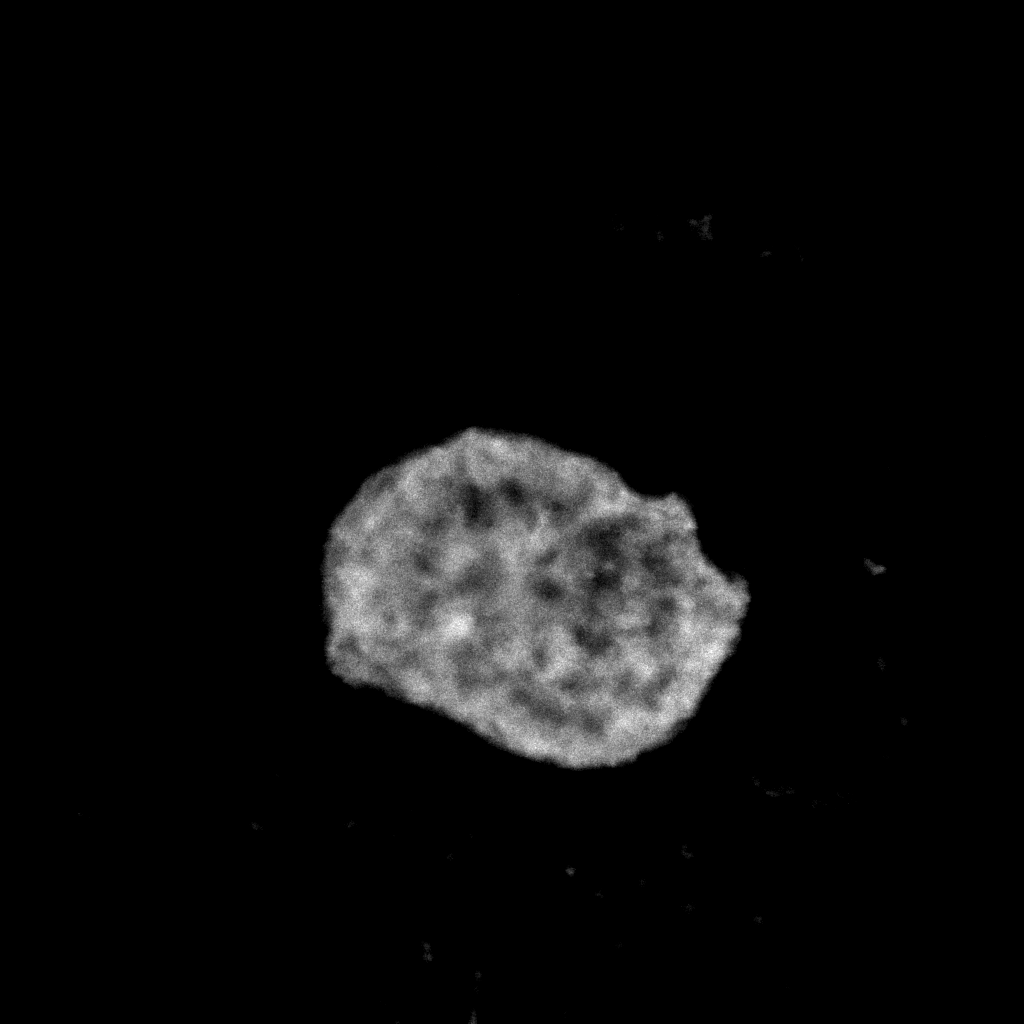

Supplement: Supplementary file 7 — Source data Fig. 5 [file 44318_2025_672_MOESM7_ESM.zip › Figure 5/5A/DKO_DAPI.tif]

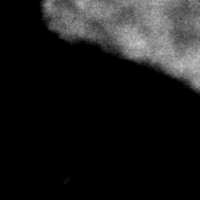

Supplement: Supplementary file 7 — Source data Fig. 5 [file 44318_2025_672_MOESM7_ESM.zip › Figure 5/5A/DKO_DAPI_zoom.tif]

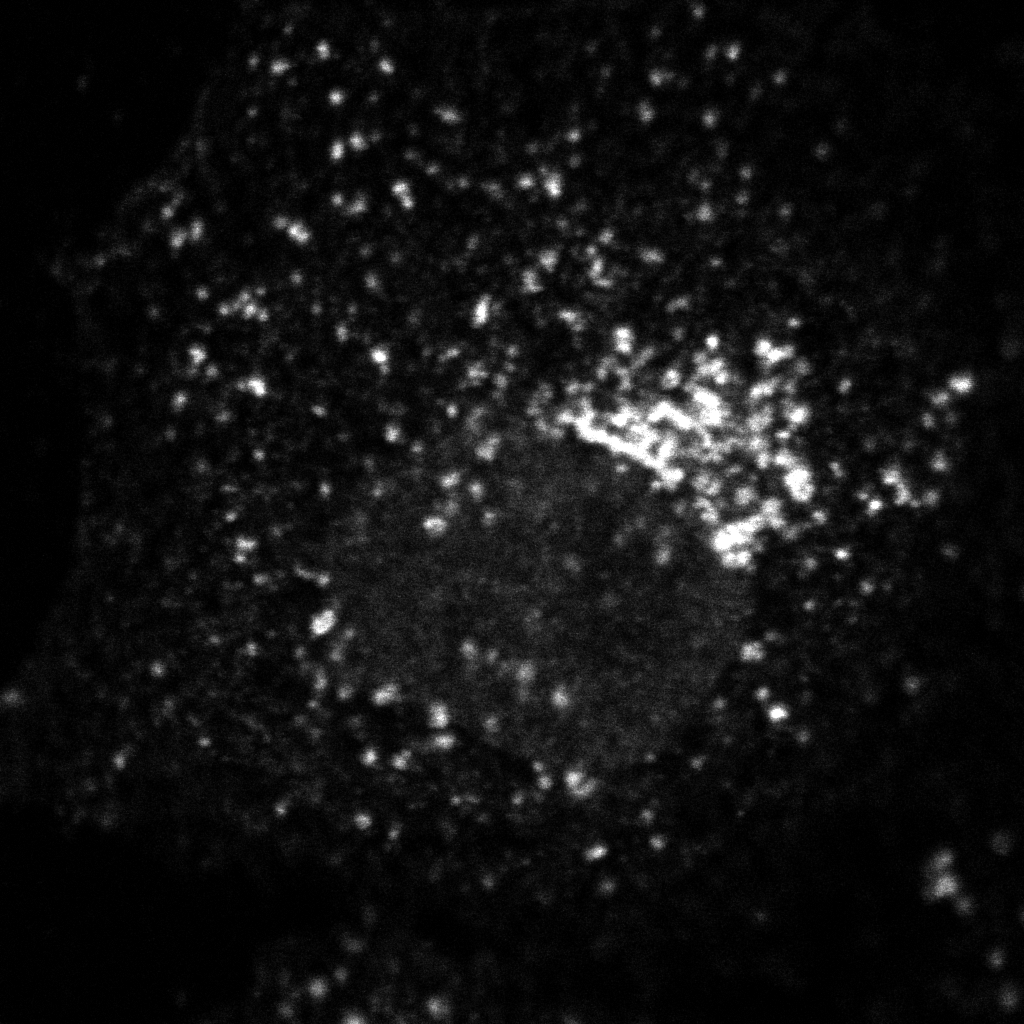

Supplement: Supplementary file 7 — Source data Fig. 5 [file 44318_2025_672_MOESM7_ESM.zip › Figure 5/5A/DKO_LAMP.tif]

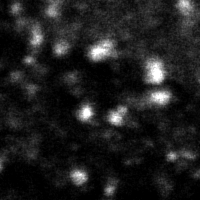

Supplement: Supplementary file 7 — Source data Fig. 5 [file 44318_2025_672_MOESM7_ESM.zip › Figure 5/5A/DKO_LAMP_zoom.tif]

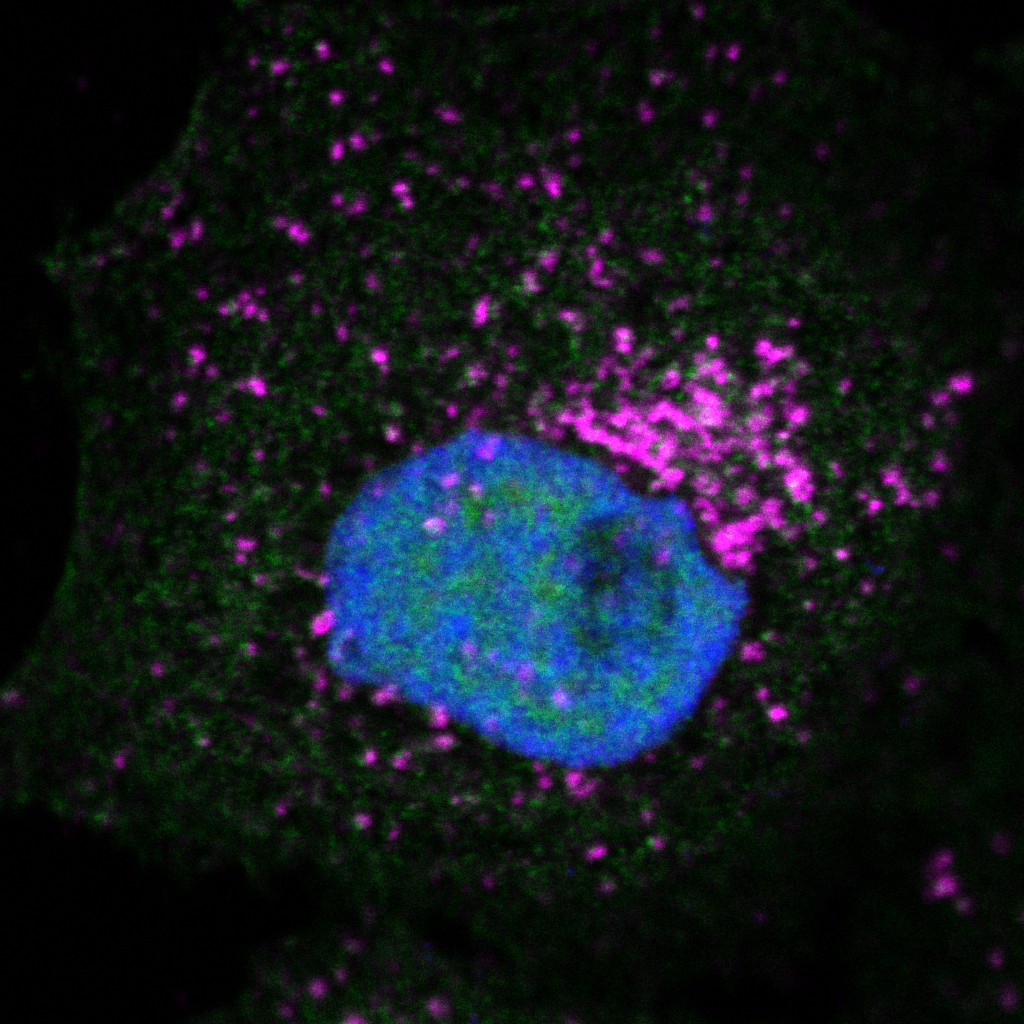

Supplement: Supplementary file 7 — Source data Fig. 5 [file 44318_2025_672_MOESM7_ESM.zip › Figure 5/5A/DKO_merge.tif]

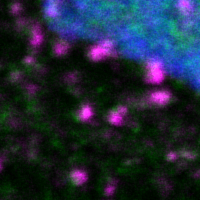

Supplement: Supplementary file 7 — Source data Fig. 5 [file 44318_2025_672_MOESM7_ESM.zip › Figure 5/5A/DKO_merge_zoom.tif]

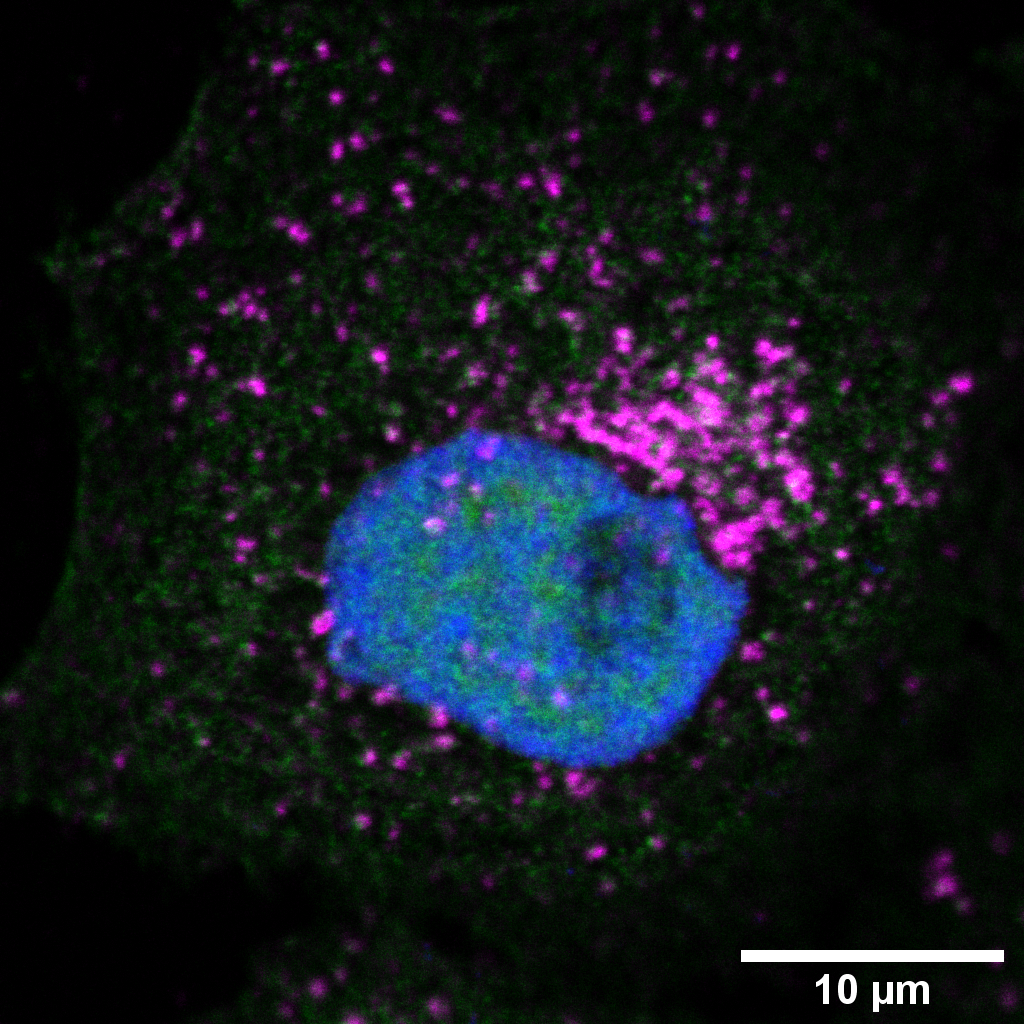

Supplement: Supplementary file 7 — Source data Fig. 5 [file 44318_2025_672_MOESM7_ESM.zip › Figure 5/5A/DKO_scale.tif]

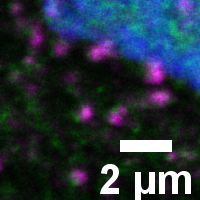

Supplement: Supplementary file 7 — Source data Fig. 5 [file 44318_2025_672_MOESM7_ESM.zip › Figure 5/5A/DKO_scale_zoom.tif]

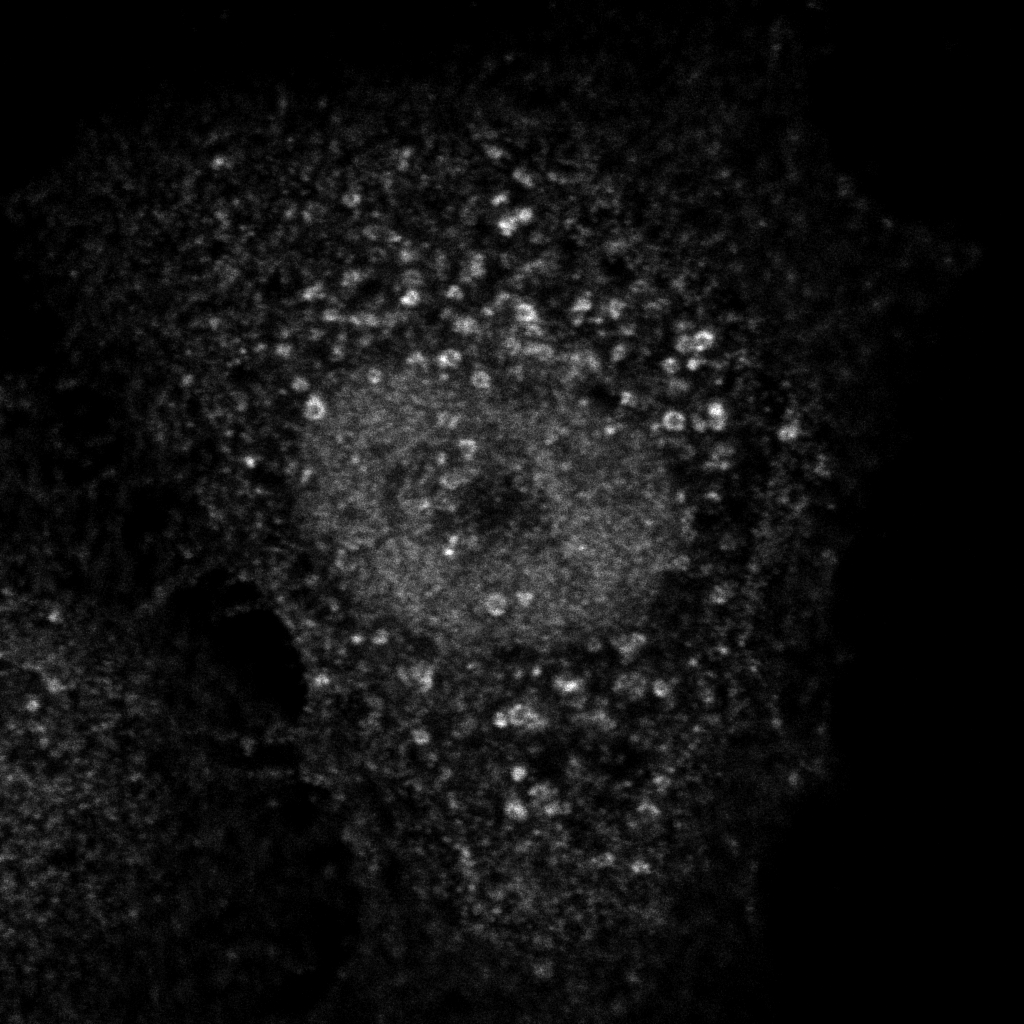

Supplement: Supplementary file 7 — Source data Fig. 5 [file 44318_2025_672_MOESM7_ESM.zip › Figure 5/5A/TECKO_ALG2.tif]

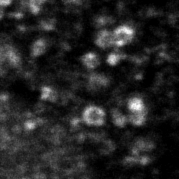

Supplement: Supplementary file 7 — Source data Fig. 5 [file 44318_2025_672_MOESM7_ESM.zip › Figure 5/5A/TECKO_ALG2_zoom.tif]

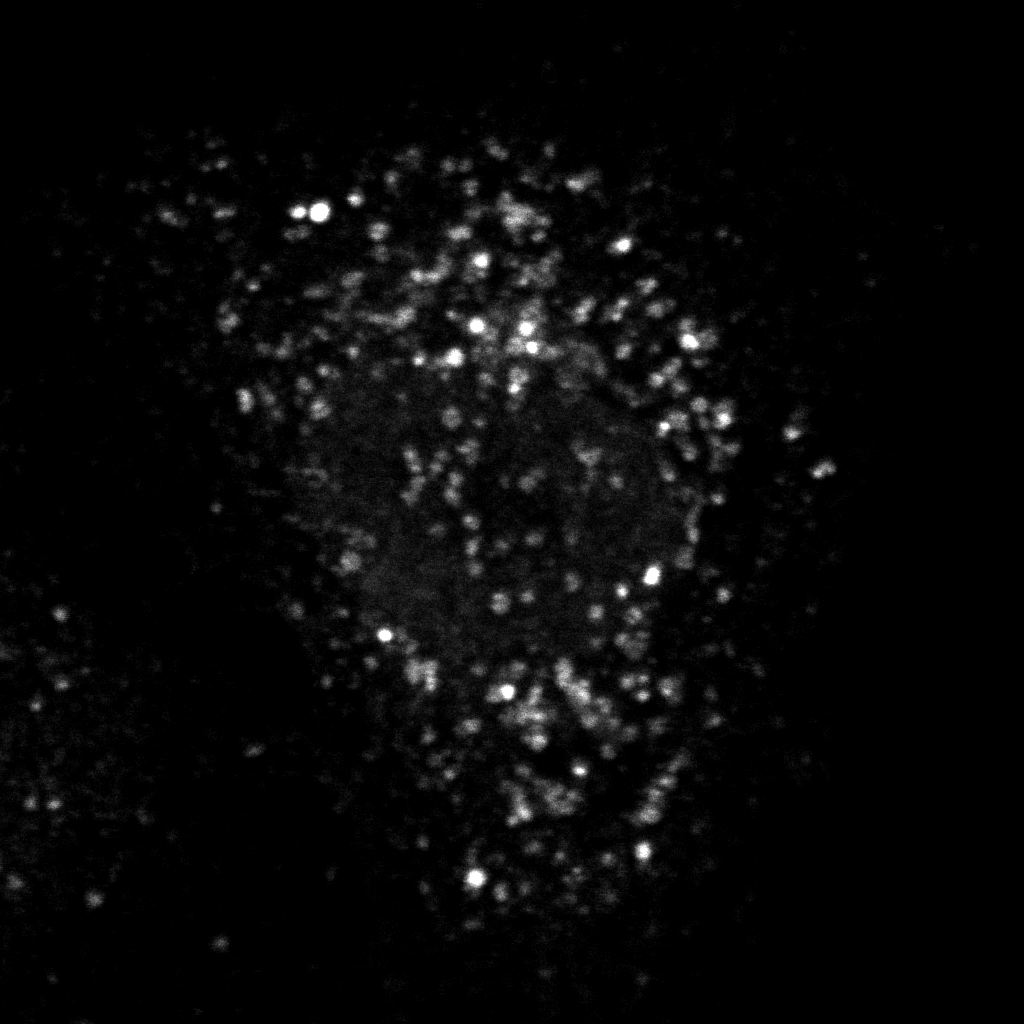

Supplement: Supplementary file 7 — Source data Fig. 5 [file 44318_2025_672_MOESM7_ESM.zip › Figure 5/5A/TECKO_LAMP.tif]

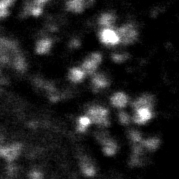

Supplement: Supplementary file 7 — Source data Fig. 5 [file 44318_2025_672_MOESM7_ESM.zip › Figure 5/5A/TECKO_LAMP_zoom.tif]

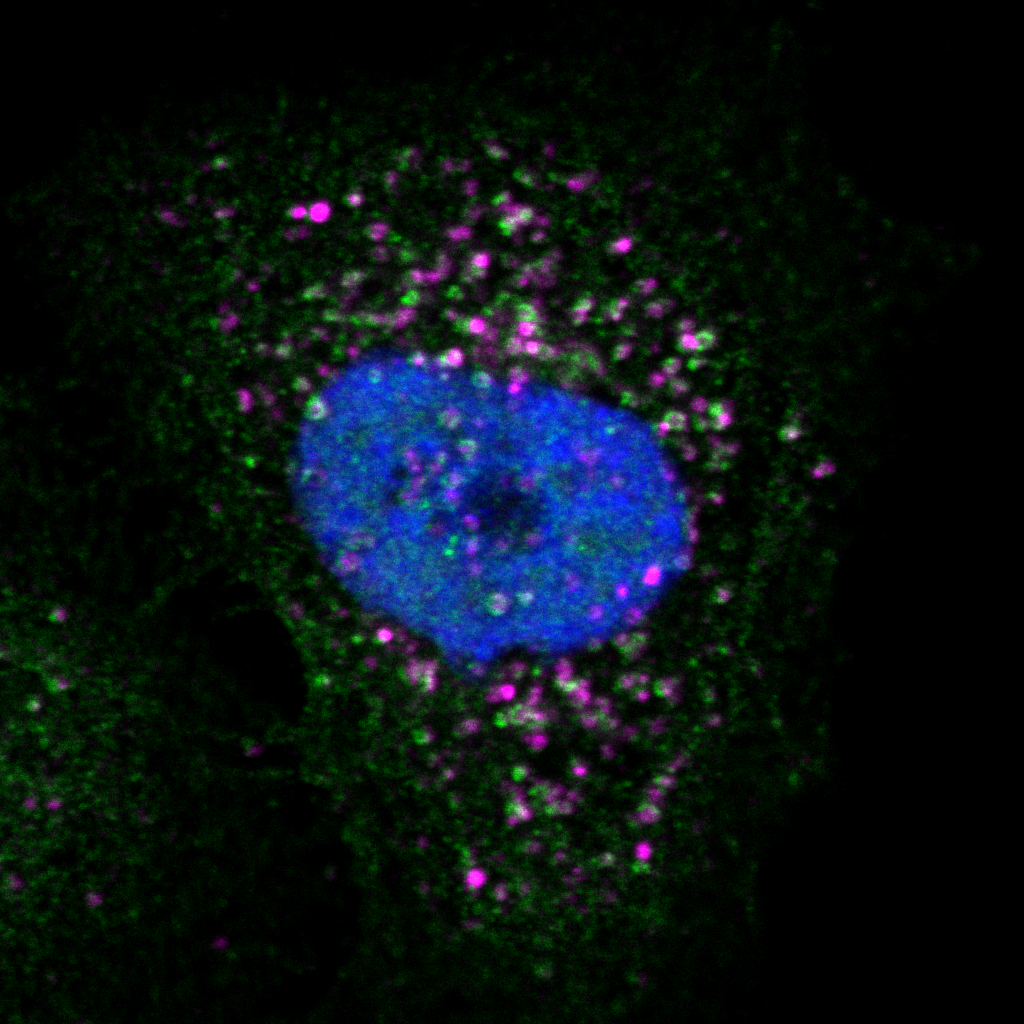

Supplement: Supplementary file 7 — Source data Fig. 5 [file 44318_2025_672_MOESM7_ESM.zip › Figure 5/5A/TECKO_merge.tif]

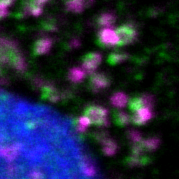

Supplement: Supplementary file 7 — Source data Fig. 5 [file 44318_2025_672_MOESM7_ESM.zip › Figure 5/5A/TECKO_merge_zoom.tif]

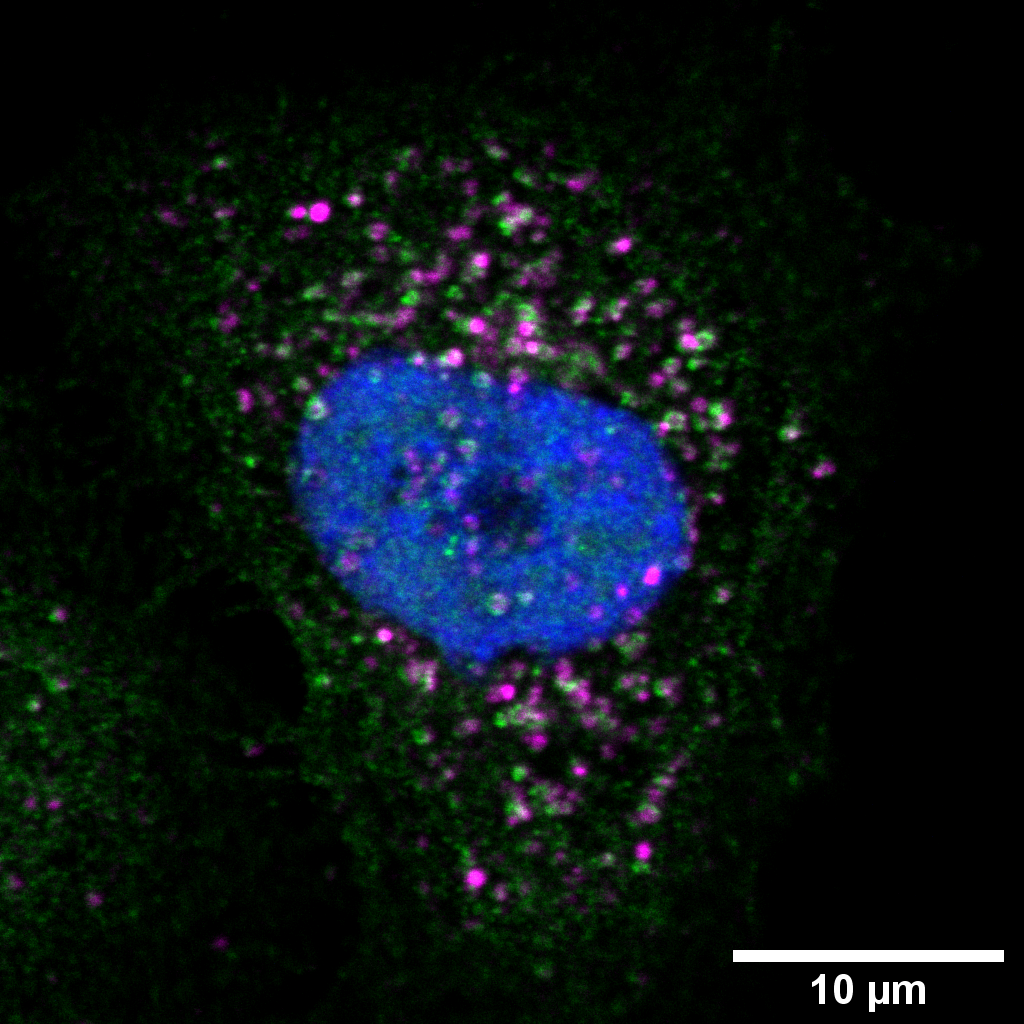

Supplement: Supplementary file 7 — Source data Fig. 5 [file 44318_2025_672_MOESM7_ESM.zip › Figure 5/5A/TECKO_scale.tif]

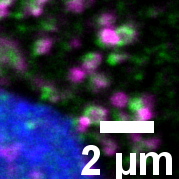

Supplement: Supplementary file 7 — Source data Fig. 5 [file 44318_2025_672_MOESM7_ESM.zip › Figure 5/5A/TECKO_scale_zoom.tif]

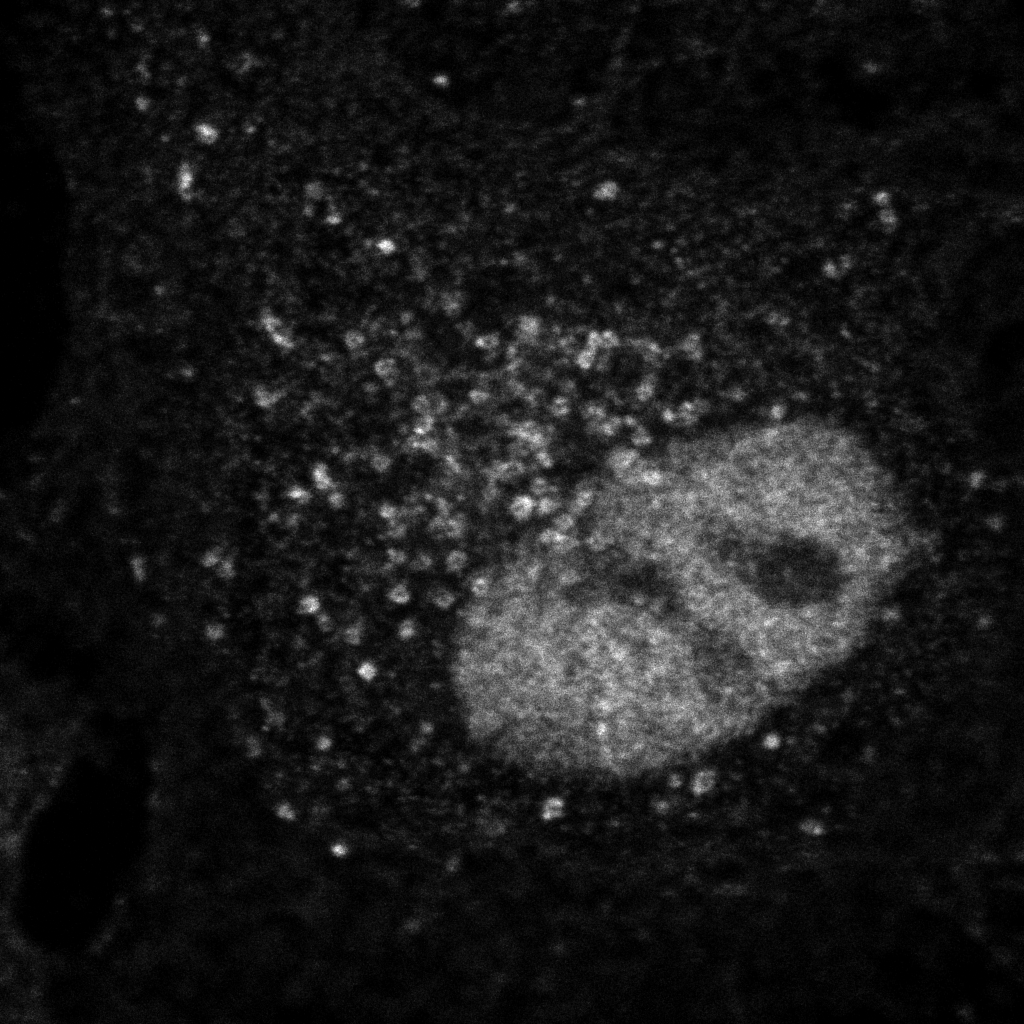

Supplement: Supplementary file 7 — Source data Fig. 5 [file 44318_2025_672_MOESM7_ESM.zip › Figure 5/5A/WT_ALG2.tif]

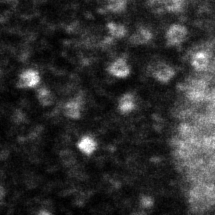

Supplement: Supplementary file 7 — Source data Fig. 5 [file 44318_2025_672_MOESM7_ESM.zip › Figure 5/5A/WT_ALG2_zoom.tif]

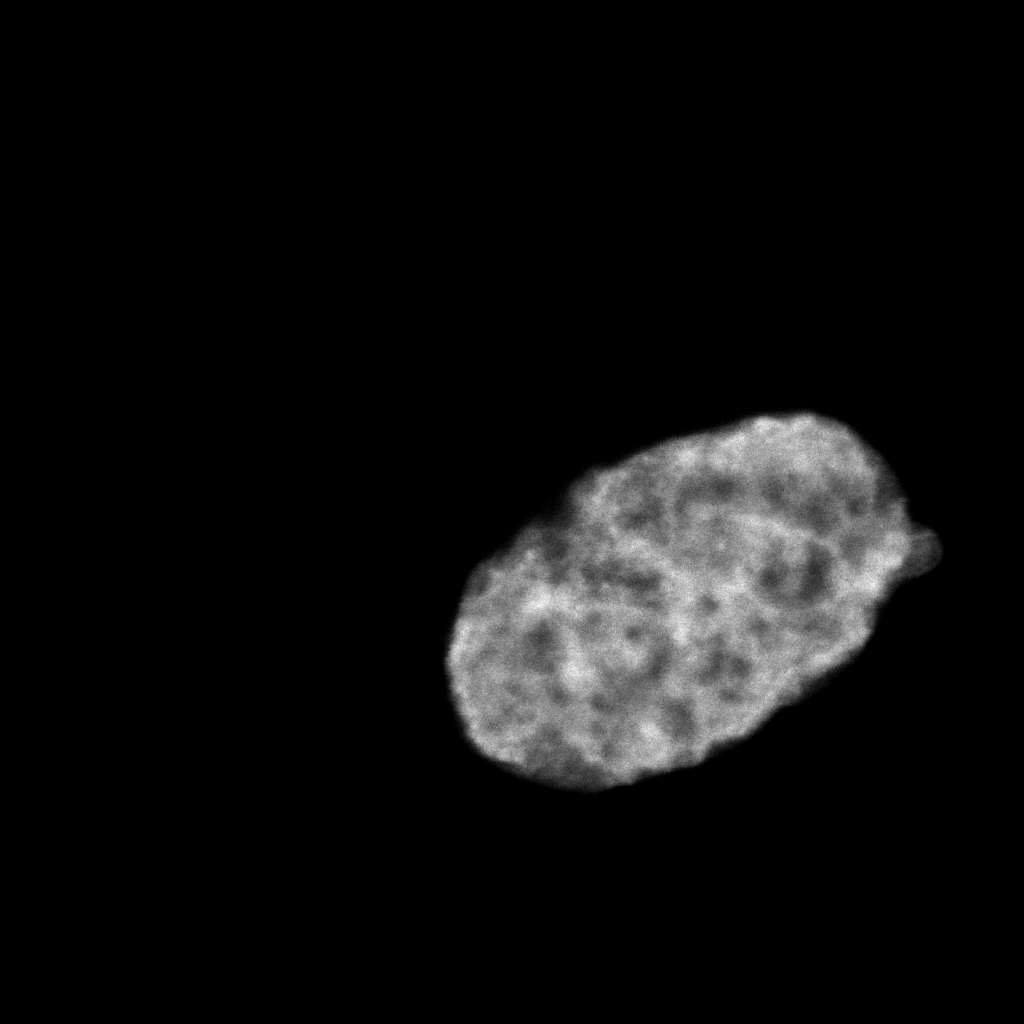

Supplement: Supplementary file 7 — Source data Fig. 5 [file 44318_2025_672_MOESM7_ESM.zip › Figure 5/5A/WT_DAPI.tif]

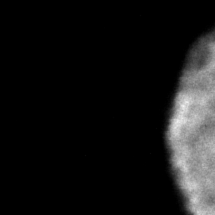

Supplement: Supplementary file 7 — Source data Fig. 5 [file 44318_2025_672_MOESM7_ESM.zip › Figure 5/5A/WT_DAPI_zoom.tif]

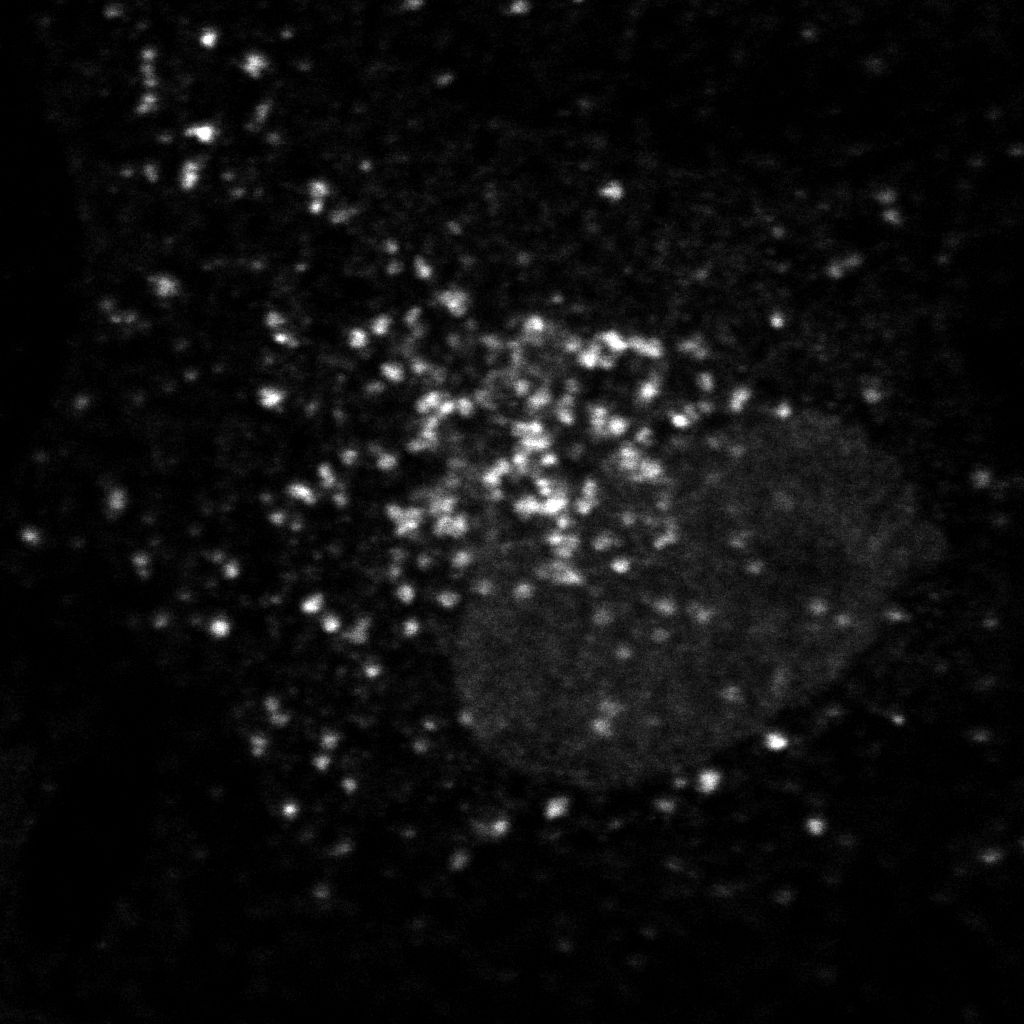

Supplement: Supplementary file 7 — Source data Fig. 5 [file 44318_2025_672_MOESM7_ESM.zip › Figure 5/5A/WT_LAMP.tif]

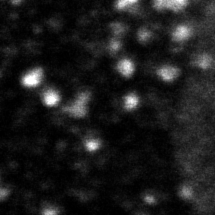

Supplement: Supplementary file 7 — Source data Fig. 5 [file 44318_2025_672_MOESM7_ESM.zip › Figure 5/5A/WT_LAMP_zoom.tif]

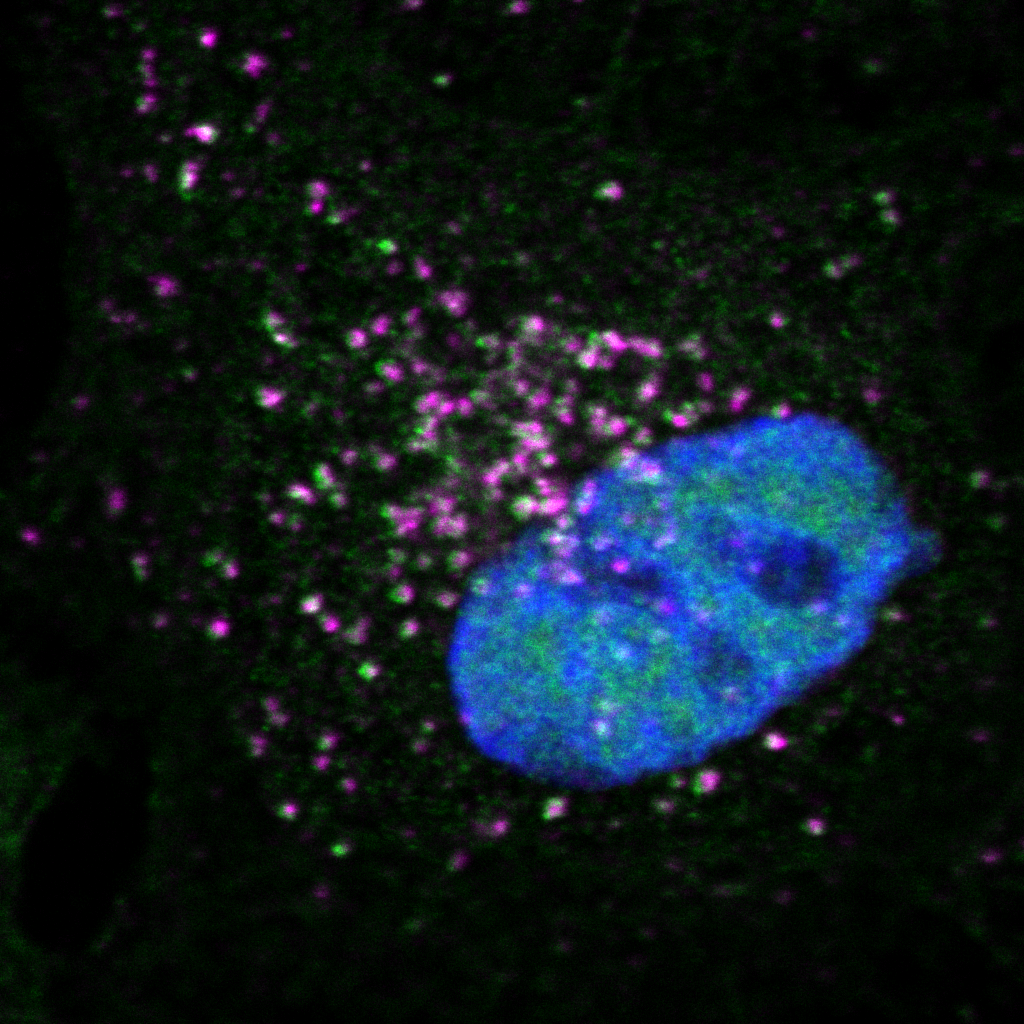

Supplement: Supplementary file 7 — Source data Fig. 5 [file 44318_2025_672_MOESM7_ESM.zip › Figure 5/5A/WT_merge.tif]

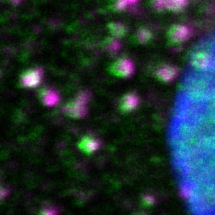

Supplement: Supplementary file 7 — Source data Fig. 5 [file 44318_2025_672_MOESM7_ESM.zip › Figure 5/5A/WT_merge_zoom.tif]

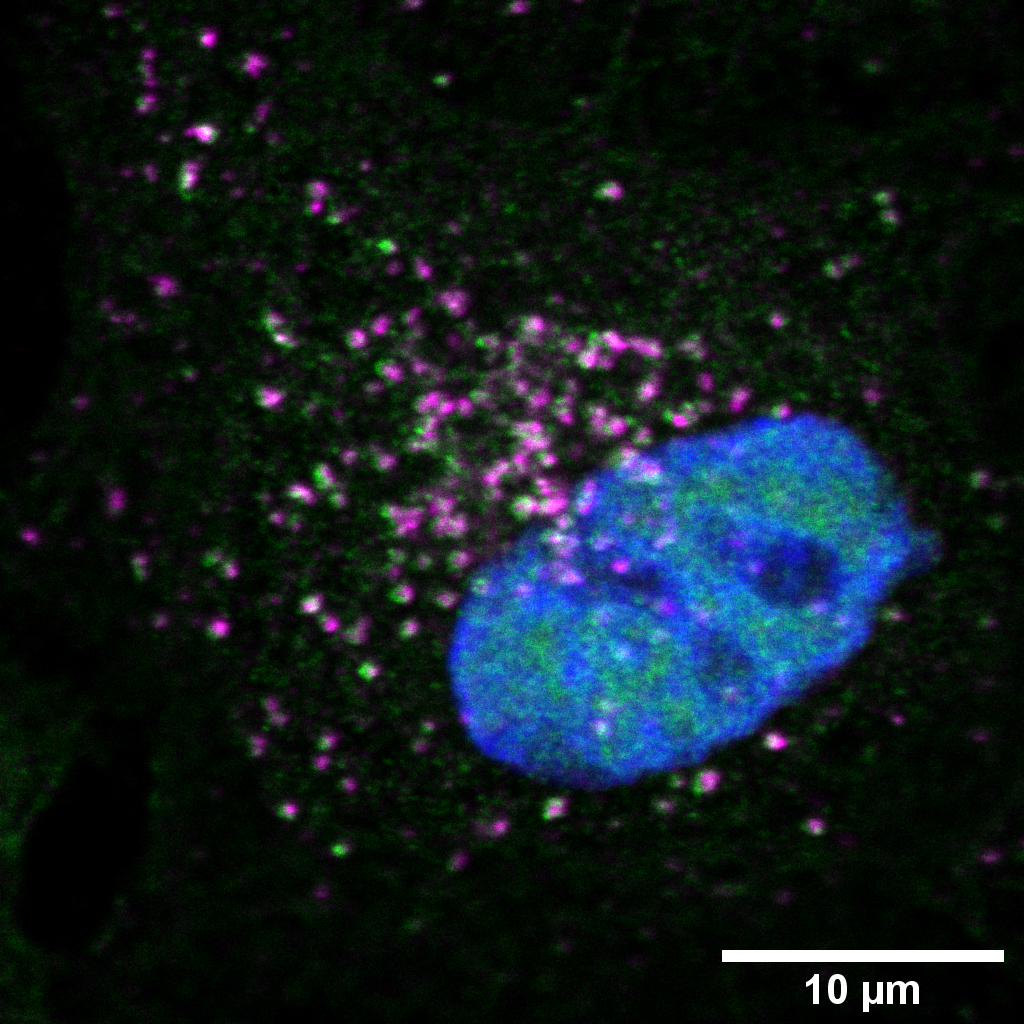

Supplement: Supplementary file 7 — Source data Fig. 5 [file 44318_2025_672_MOESM7_ESM.zip › Figure 5/5A/WT_scale.tif]

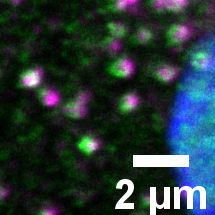

Supplement: Supplementary file 7 — Source data Fig. 5 [file 44318_2025_672_MOESM7_ESM.zip › Figure 5/5A/WT_scale_zoom.tif]

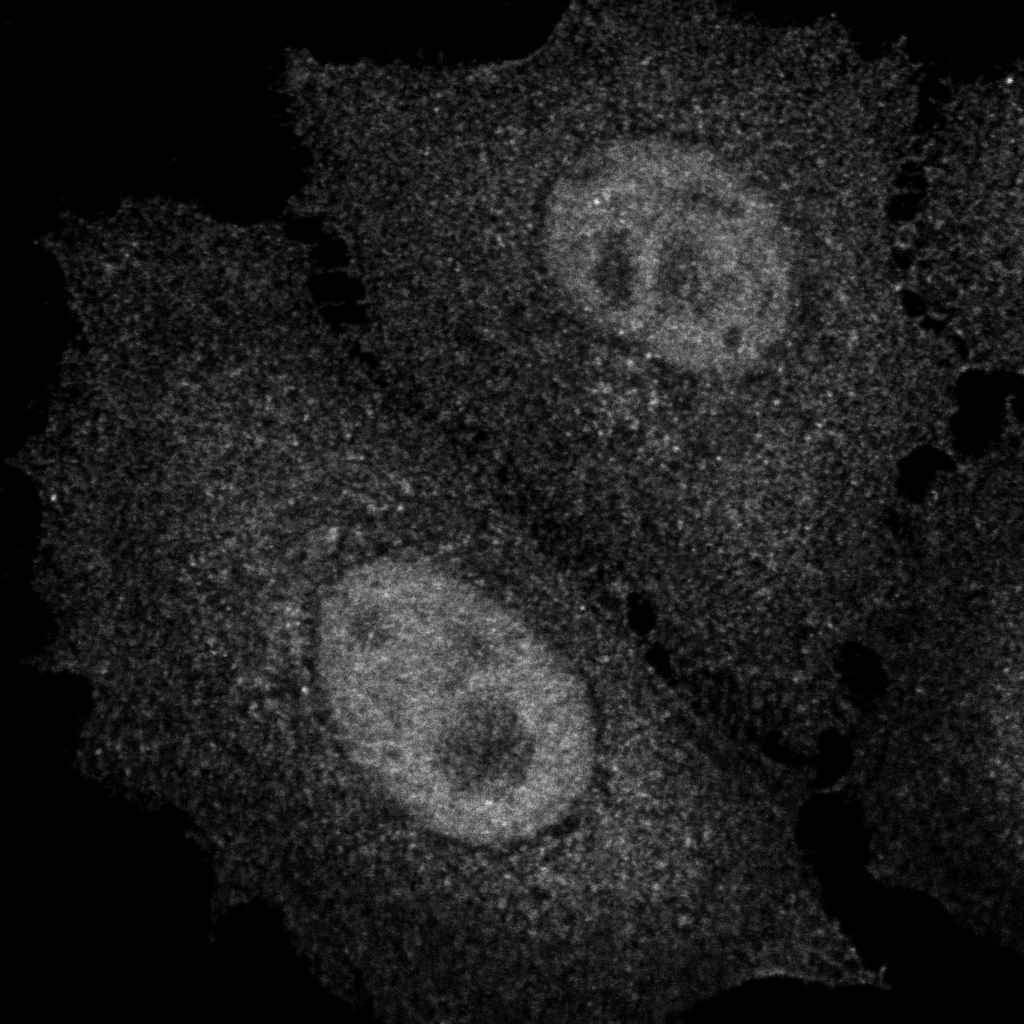

Supplement: Supplementary file 7 — Source data Fig. 5 [file 44318_2025_672_MOESM7_ESM.zip › Figure 5/5C/K130R_LLOMe_ALG2.tif]

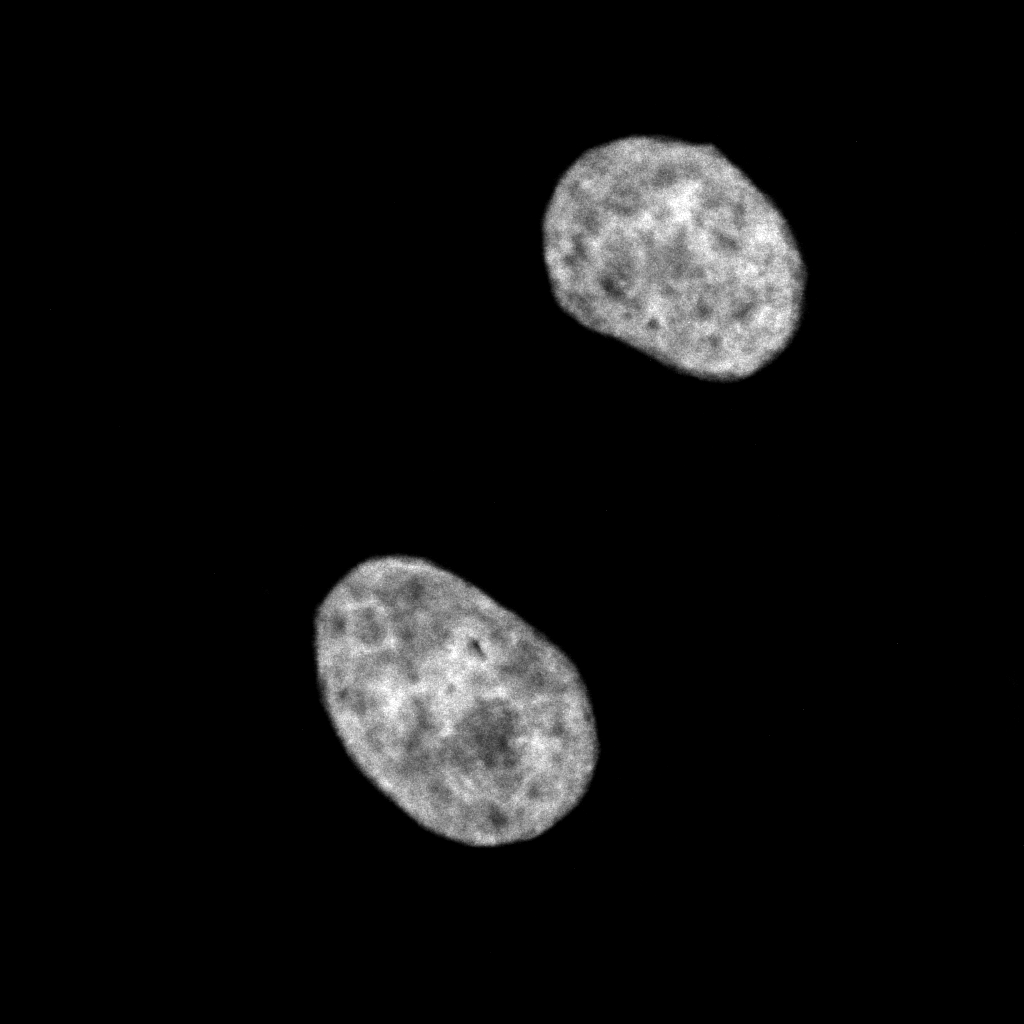

Supplement: Supplementary file 7 — Source data Fig. 5 [file 44318_2025_672_MOESM7_ESM.zip › Figure 5/5C/K130R_LLOMe_DAPI.tif]

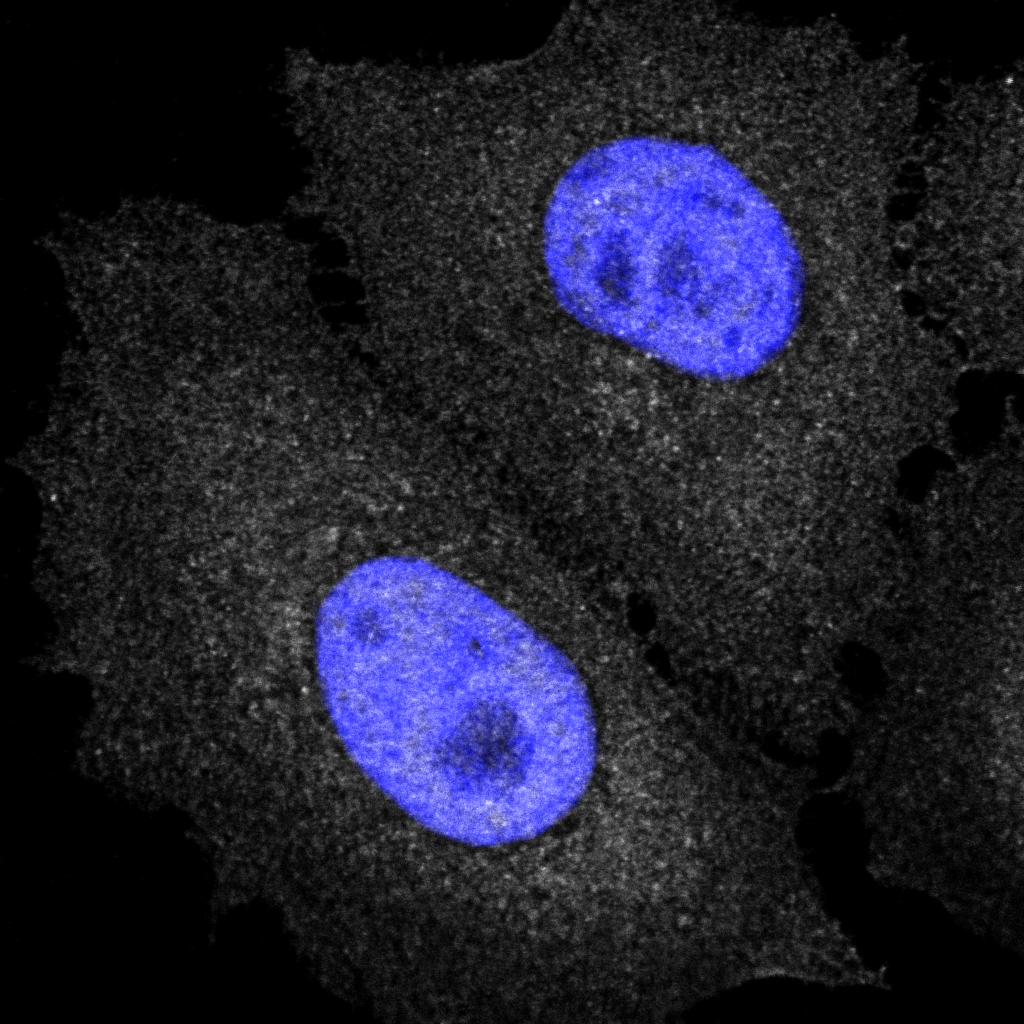

Supplement: Supplementary file 7 — Source data Fig. 5 [file 44318_2025_672_MOESM7_ESM.zip › Figure 5/5C/K130R_LLOMe_merge.tif]

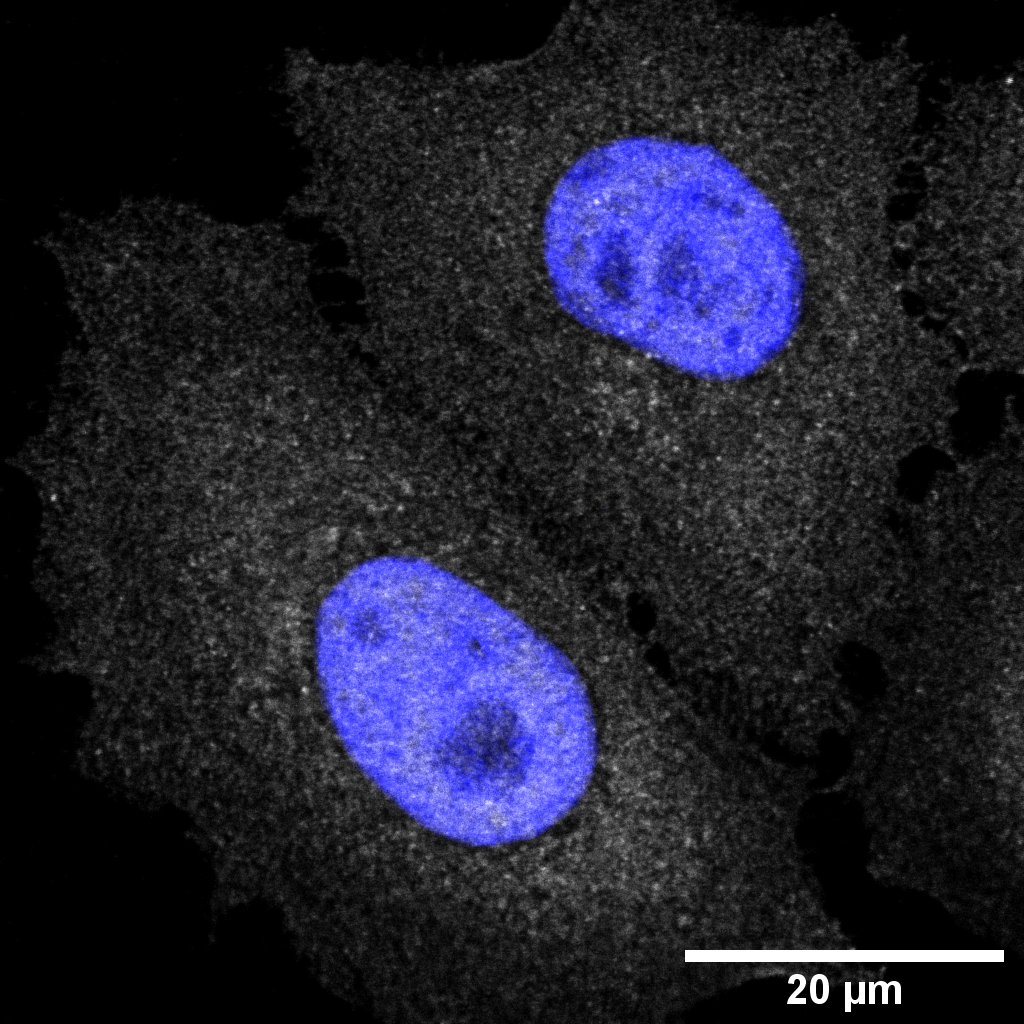

Supplement: Supplementary file 7 — Source data Fig. 5 [file 44318_2025_672_MOESM7_ESM.zip › Figure 5/5C/K130R_LLOMe_scale.tif]
