# Supplementary figures and images for: The ATG8 E3-like ligases sense lysosomal damage and initiate ESCRT-mediated membrane repair (part 3 of 7)
Source: EMBO J. 2026 Jan 3;45(3):930–52. doi: 10.1038/s44318-025-00672-1 (PMC12865045; doi:10.1038/s44318-025-00672-1)

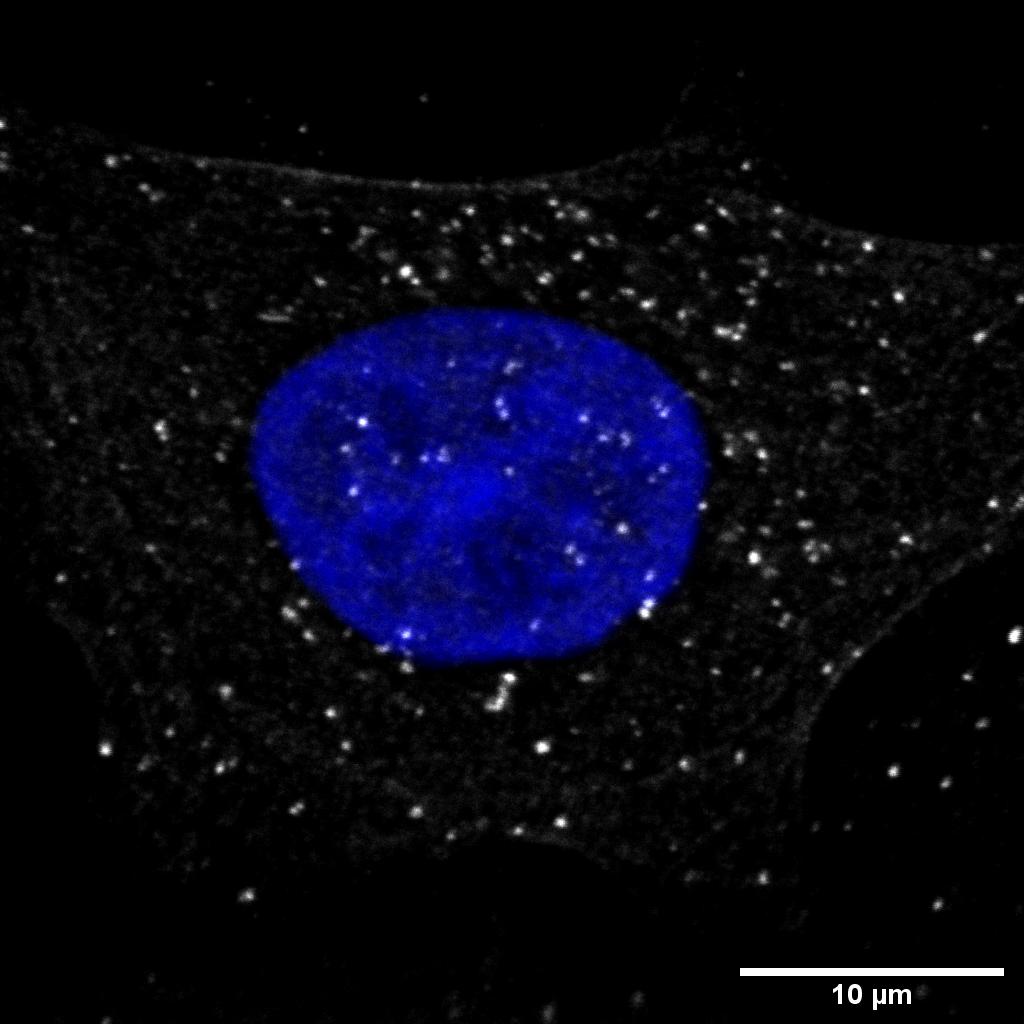

Supplement: Supplementary file 5 — Source data Fig. 3 [file 44318_2025_672_MOESM5_ESM.zip › Figure 3/3A/5KO_IST1_scale.tif]

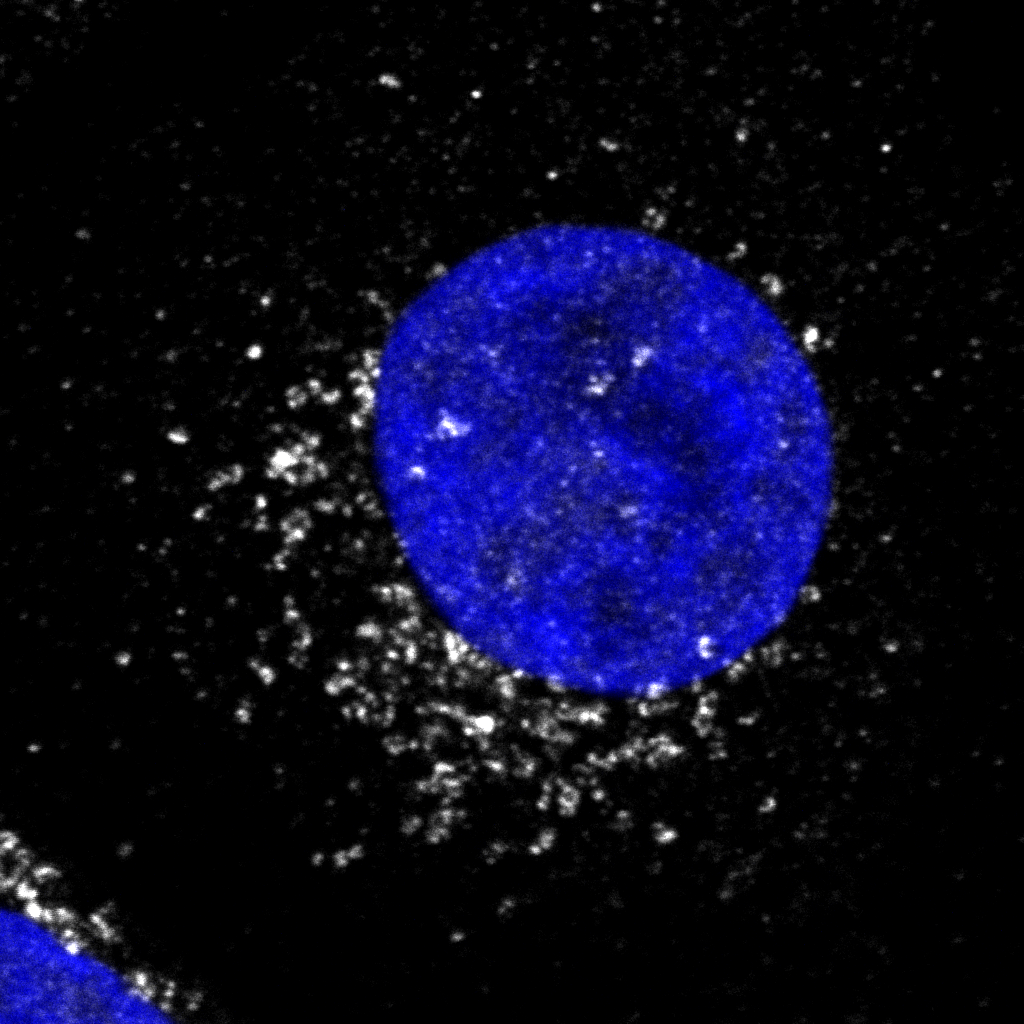

Supplement: Supplementary file 5 — Source data Fig. 3 [file 44318_2025_672_MOESM5_ESM.zip › Figure 3/3A/8KO_CHMP.tif]

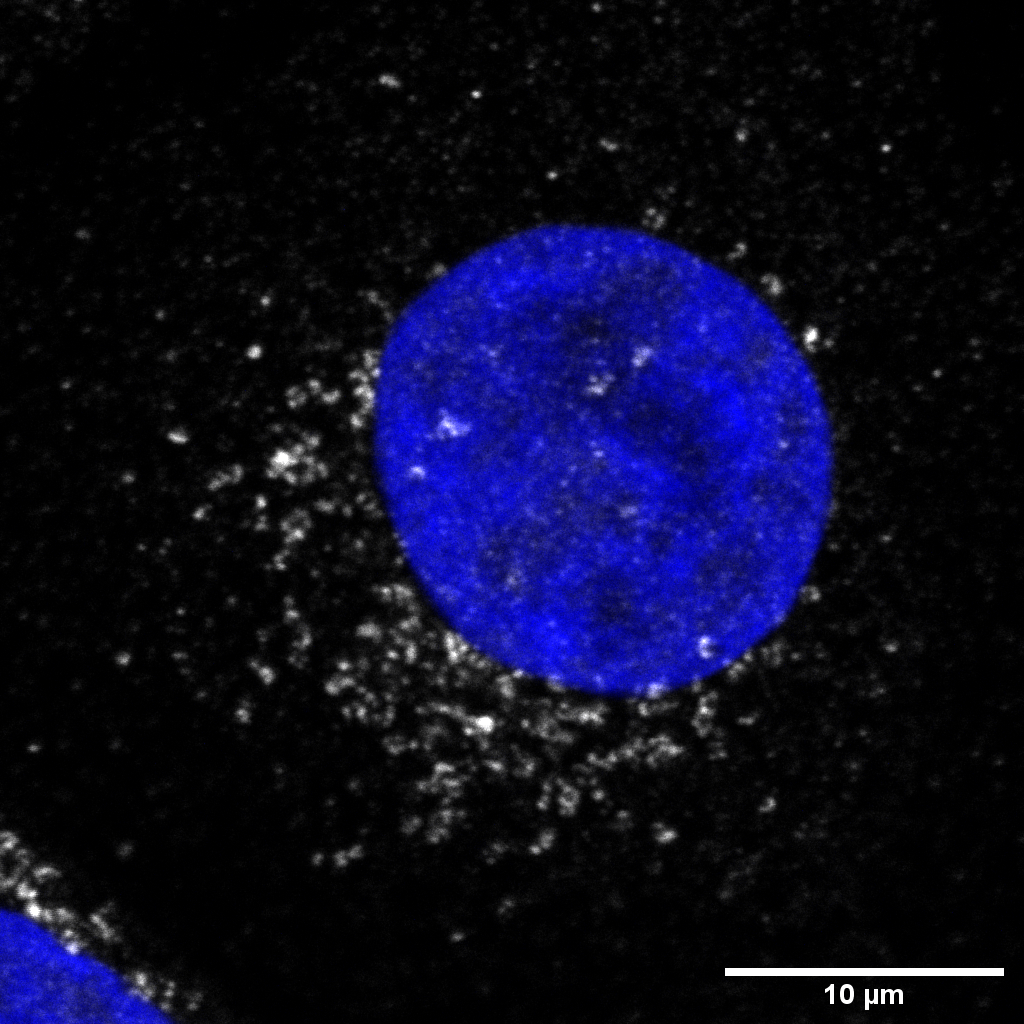

Supplement: Supplementary file 5 — Source data Fig. 3 [file 44318_2025_672_MOESM5_ESM.zip › Figure 3/3A/8KO_CHMP_scale.tif]

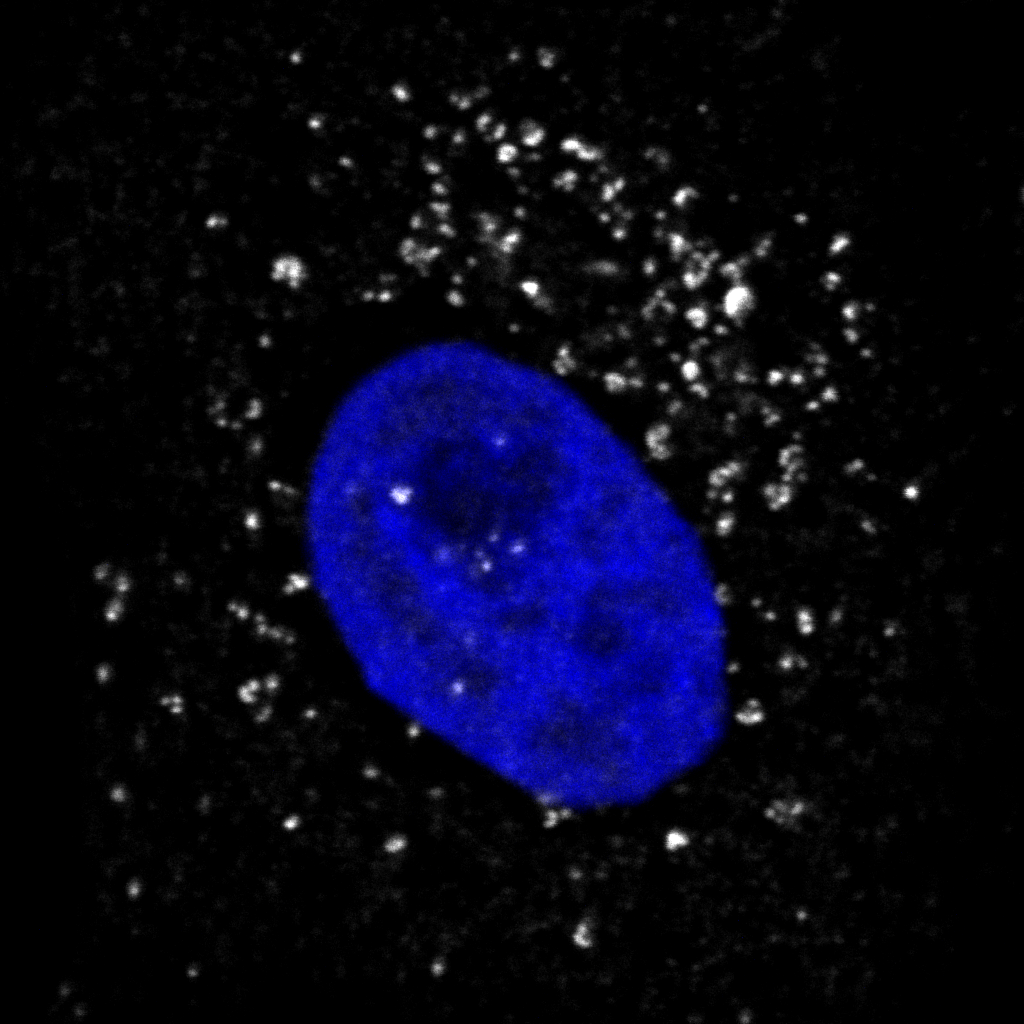

Supplement: Supplementary file 5 — Source data Fig. 3 [file 44318_2025_672_MOESM5_ESM.zip › Figure 3/3A/8KO_IST1.tif]

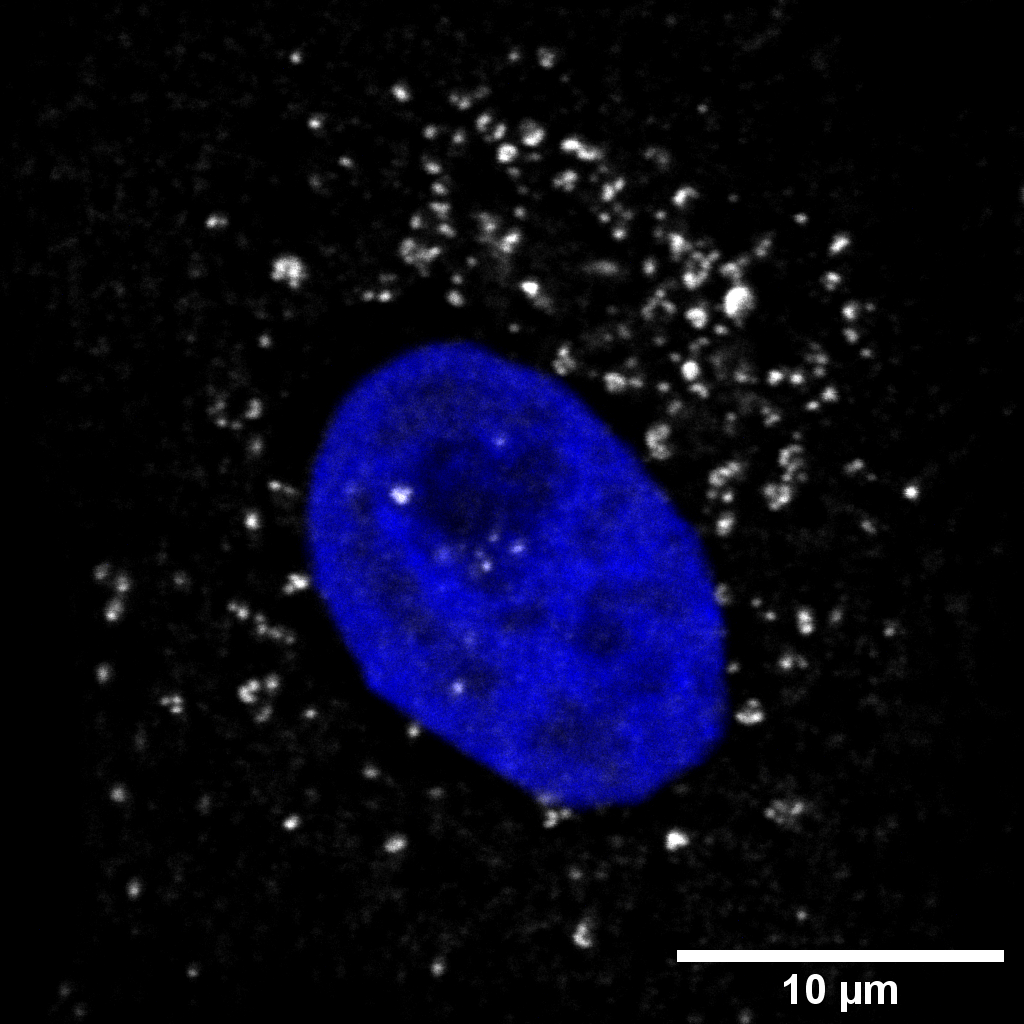

Supplement: Supplementary file 5 — Source data Fig. 3 [file 44318_2025_672_MOESM5_ESM.zip › Figure 3/3A/8KO_IST1_scale.tif]

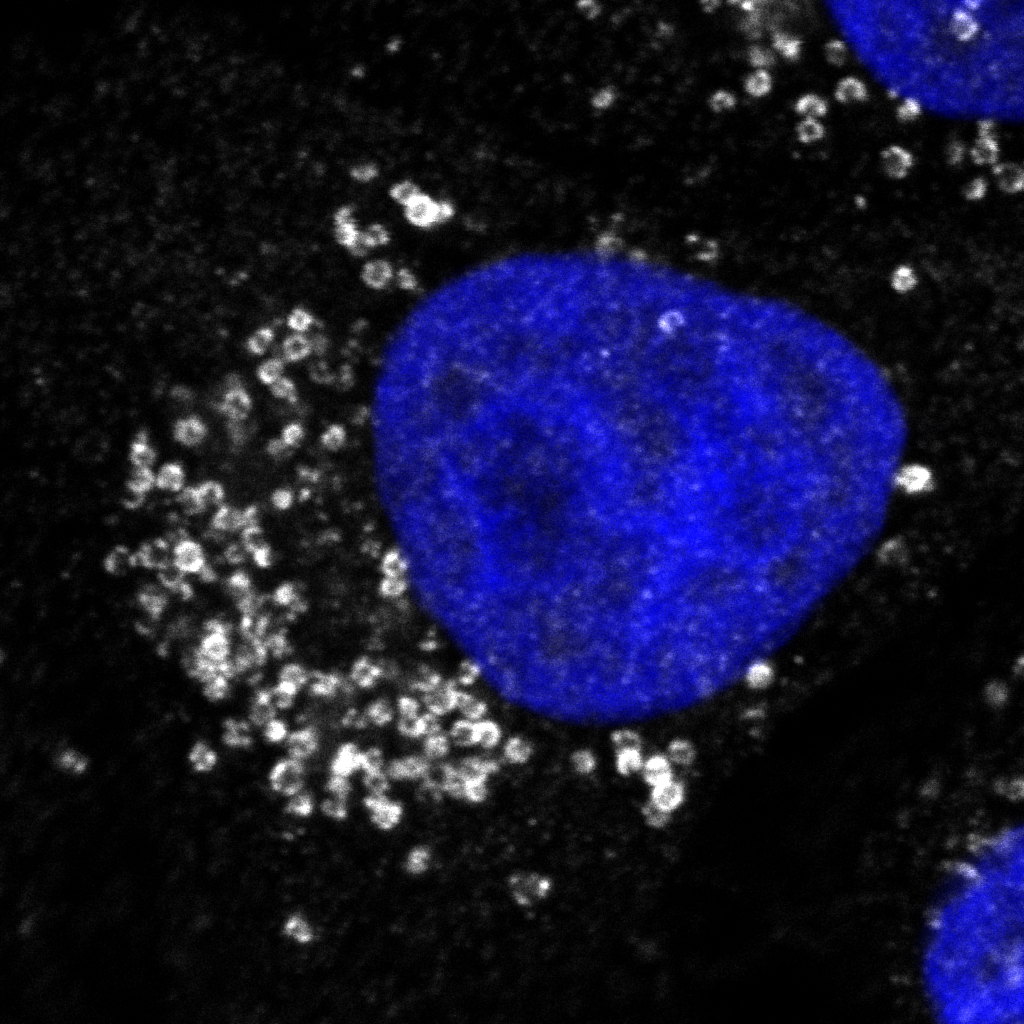

Supplement: Supplementary file 5 — Source data Fig. 3 [file 44318_2025_672_MOESM5_ESM.zip › Figure 3/3A/WT_CHMP.tif]

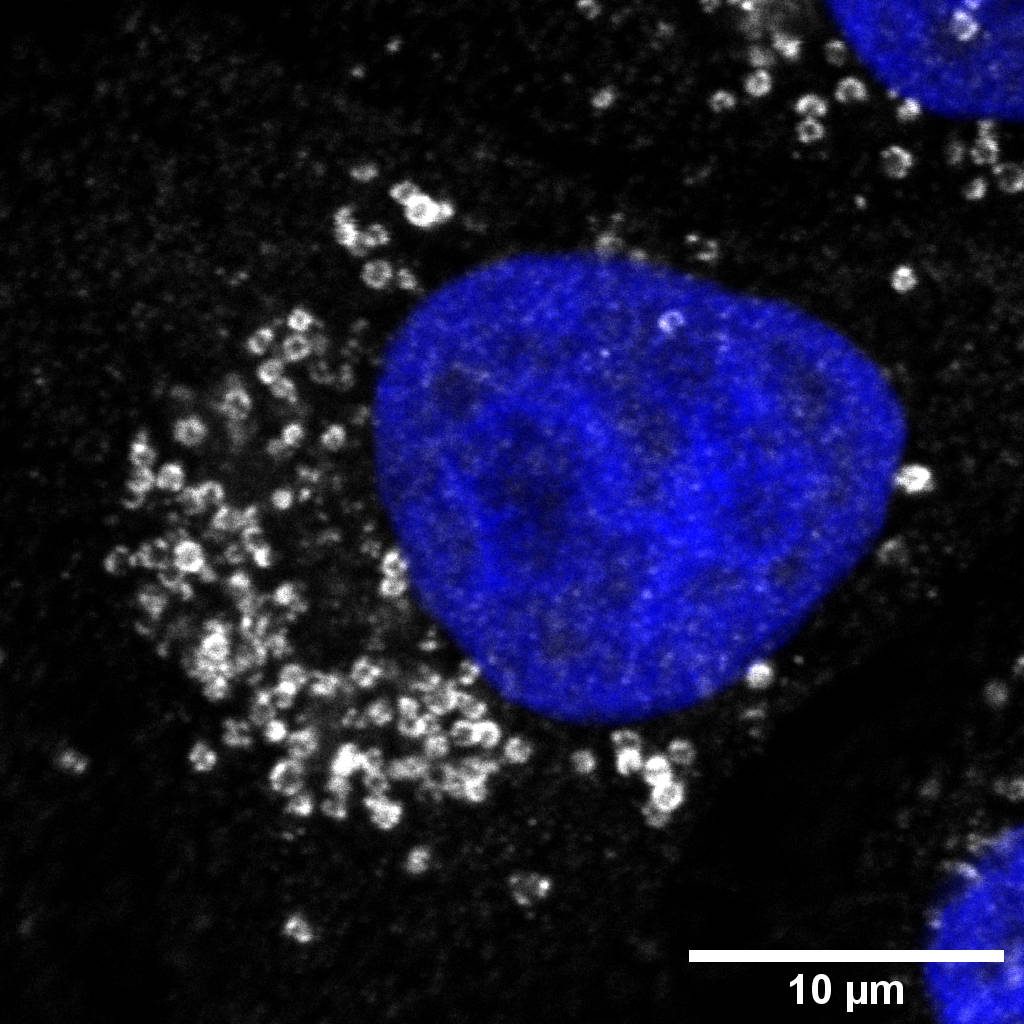

Supplement: Supplementary file 5 — Source data Fig. 3 [file 44318_2025_672_MOESM5_ESM.zip › Figure 3/3A/WT_CHMP_scale.tif]

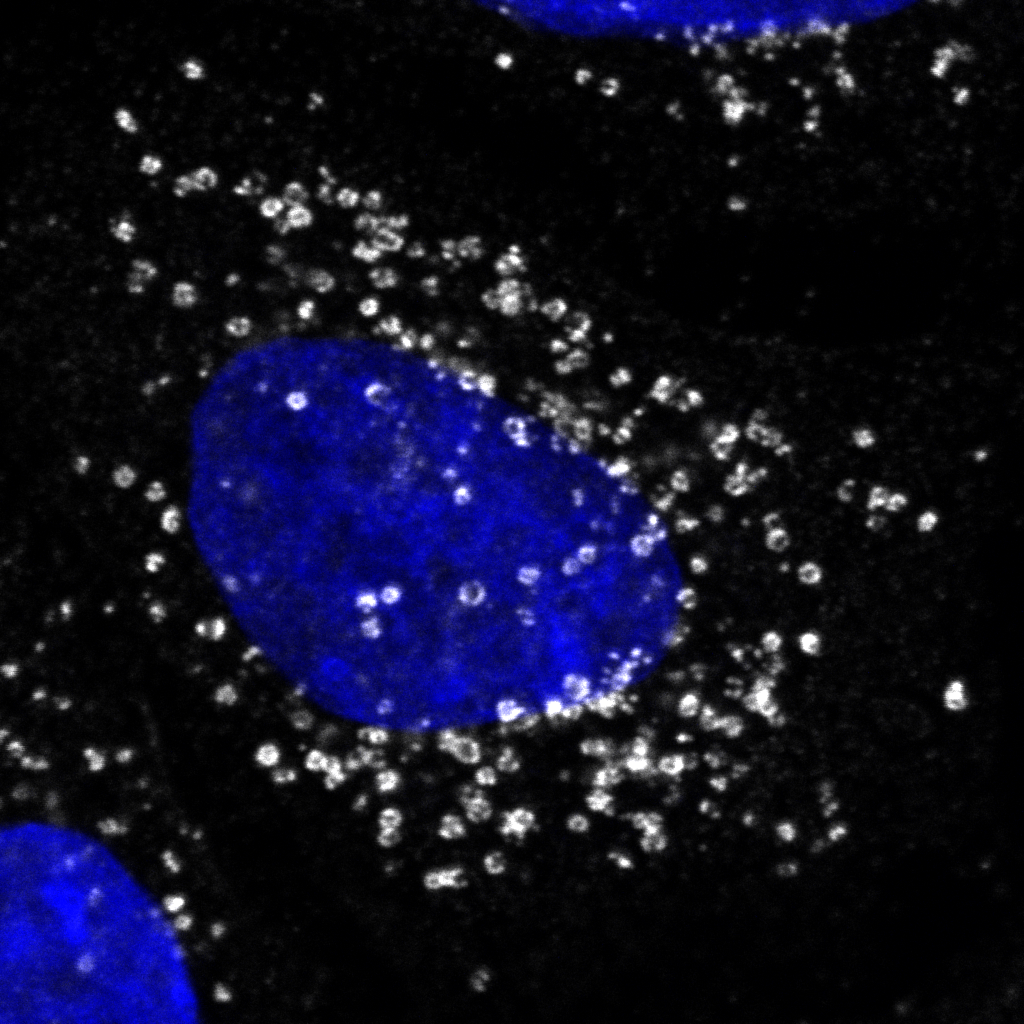

Supplement: Supplementary file 5 — Source data Fig. 3 [file 44318_2025_672_MOESM5_ESM.zip › Figure 3/3A/WT_IST1.tif]

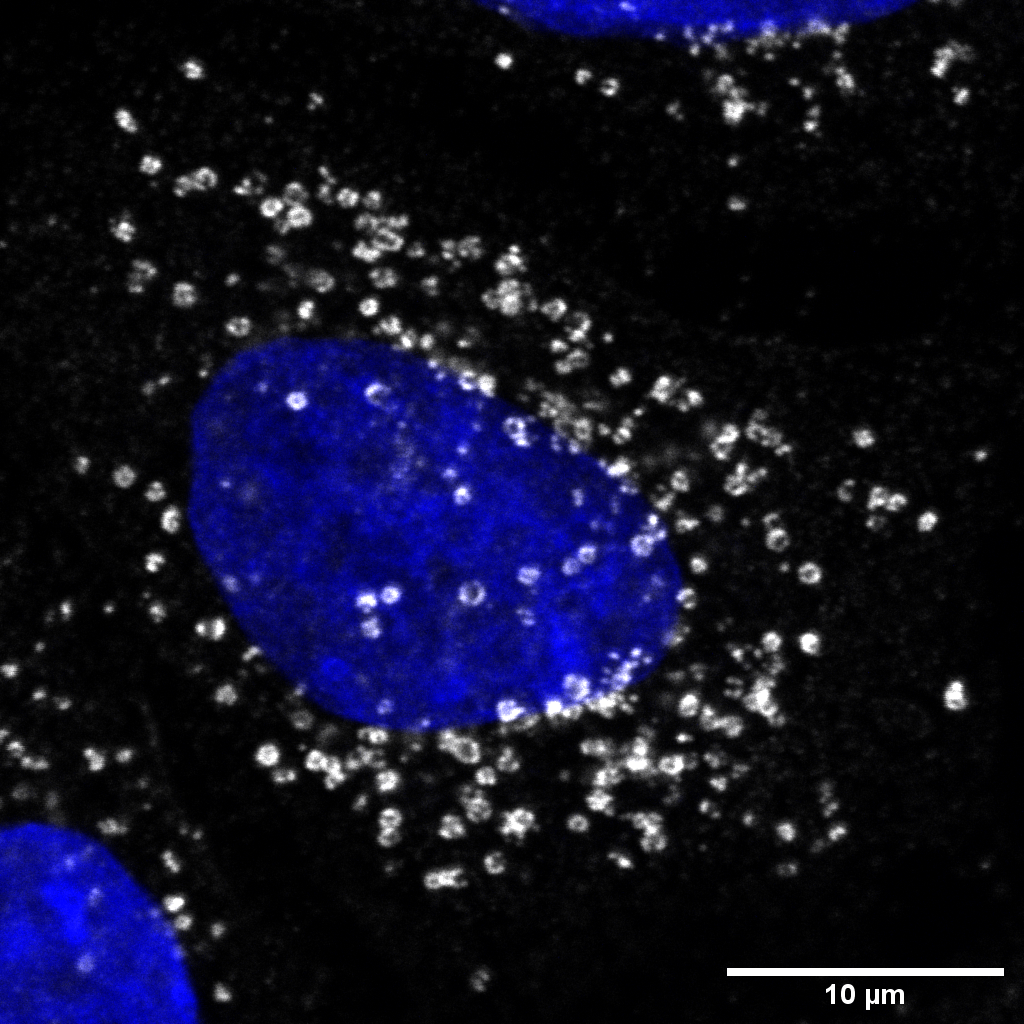

Supplement: Supplementary file 5 — Source data Fig. 3 [file 44318_2025_672_MOESM5_ESM.zip › Figure 3/3A/WT_IST1_scale.tif]

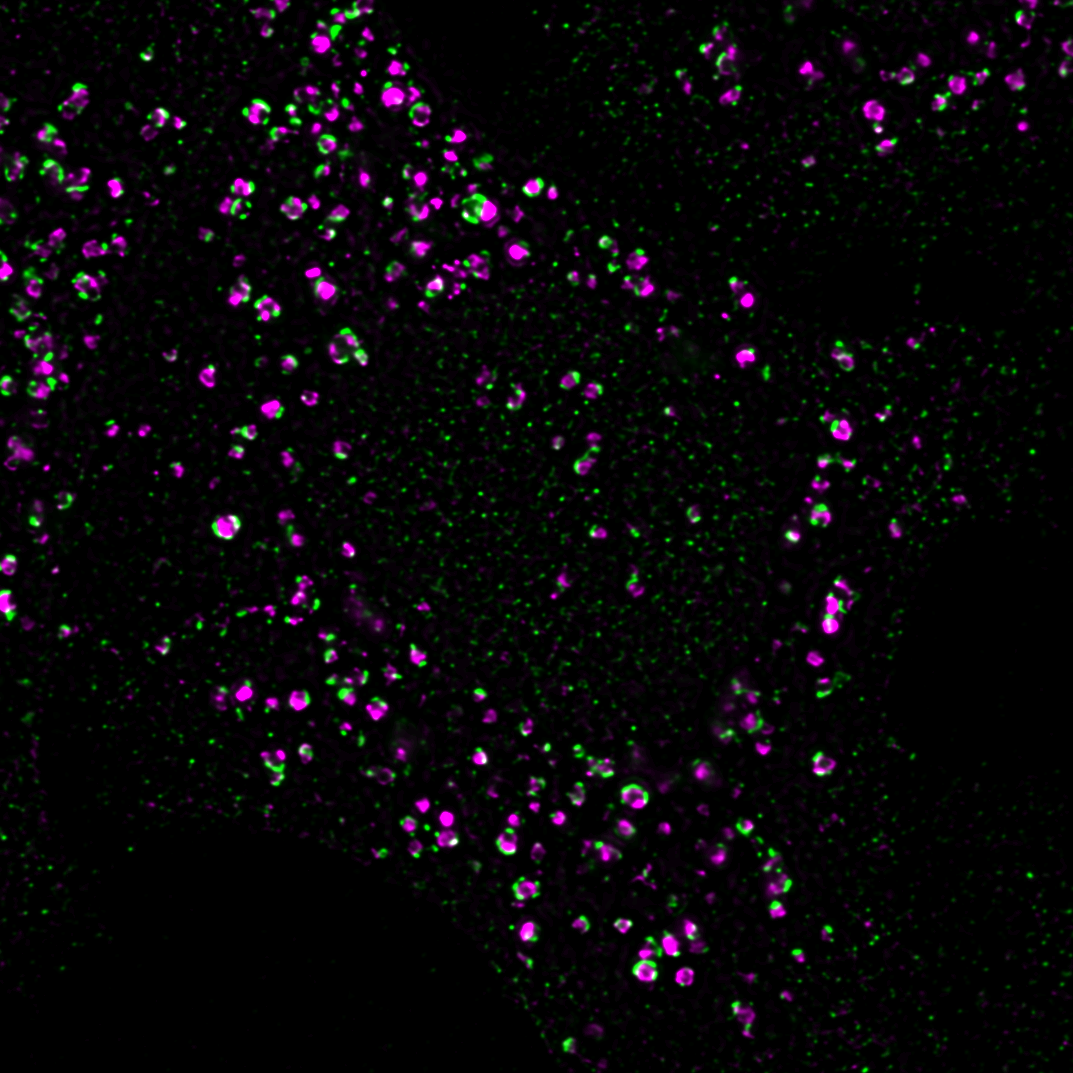

Supplement: Supplementary file 5 — Source data Fig. 3 [file 44318_2025_672_MOESM5_ESM.zip › Figure 3/3C/8KO.tif]

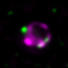

Supplement: Supplementary file 5 — Source data Fig. 3 [file 44318_2025_672_MOESM5_ESM.zip › Figure 3/3C/8KO_a.tif]

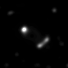

Supplement: Supplementary file 5 — Source data Fig. 3 [file 44318_2025_672_MOESM5_ESM.zip › Figure 3/3C/8KO_a_CHMP.tif]

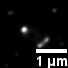

Supplement: Supplementary file 5 — Source data Fig. 3 [file 44318_2025_672_MOESM5_ESM.zip › Figure 3/3C/8KO_a_scale.tif]

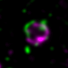

Supplement: Supplementary file 5 — Source data Fig. 3 [file 44318_2025_672_MOESM5_ESM.zip › Figure 3/3C/8KO_b.tif]

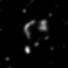

Supplement: Supplementary file 5 — Source data Fig. 3 [file 44318_2025_672_MOESM5_ESM.zip › Figure 3/3C/8KO_b_CHMP.tif]

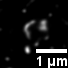

Supplement: Supplementary file 5 — Source data Fig. 3 [file 44318_2025_672_MOESM5_ESM.zip › Figure 3/3C/8KO_b_scale.tif]

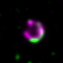

Supplement: Supplementary file 5 — Source data Fig. 3 [file 44318_2025_672_MOESM5_ESM.zip › Figure 3/3C/8KO_c.tif]

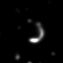

Supplement: Supplementary file 5 — Source data Fig. 3 [file 44318_2025_672_MOESM5_ESM.zip › Figure 3/3C/8KO_c_CHMP.tif]

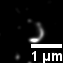

Supplement: Supplementary file 5 — Source data Fig. 3 [file 44318_2025_672_MOESM5_ESM.zip › Figure 3/3C/8KO_c_scale.tif]

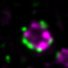

Supplement: Supplementary file 5 — Source data Fig. 3 [file 44318_2025_672_MOESM5_ESM.zip › Figure 3/3C/8KO_d.tif]

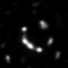

Supplement: Supplementary file 5 — Source data Fig. 3 [file 44318_2025_672_MOESM5_ESM.zip › Figure 3/3C/8KO_d_CHMP.tif]

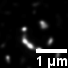

Supplement: Supplementary file 5 — Source data Fig. 3 [file 44318_2025_672_MOESM5_ESM.zip › Figure 3/3C/8KO_d_scale.tif]

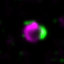

Supplement: Supplementary file 5 — Source data Fig. 3 [file 44318_2025_672_MOESM5_ESM.zip › Figure 3/3C/8KO_e.tif]

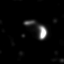

Supplement: Supplementary file 5 — Source data Fig. 3 [file 44318_2025_672_MOESM5_ESM.zip › Figure 3/3C/8KO_e_CHMP.tif]

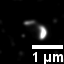

Supplement: Supplementary file 5 — Source data Fig. 3 [file 44318_2025_672_MOESM5_ESM.zip › Figure 3/3C/8KO_e_scale.tif]

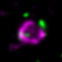

Supplement: Supplementary file 5 — Source data Fig. 3 [file 44318_2025_672_MOESM5_ESM.zip › Figure 3/3C/8KO_f.tif]

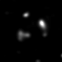

Supplement: Supplementary file 5 — Source data Fig. 3 [file 44318_2025_672_MOESM5_ESM.zip › Figure 3/3C/8KO_f_CHMP.tif]

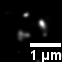

Supplement: Supplementary file 5 — Source data Fig. 3 [file 44318_2025_672_MOESM5_ESM.zip › Figure 3/3C/8KO_f_scale.tif]

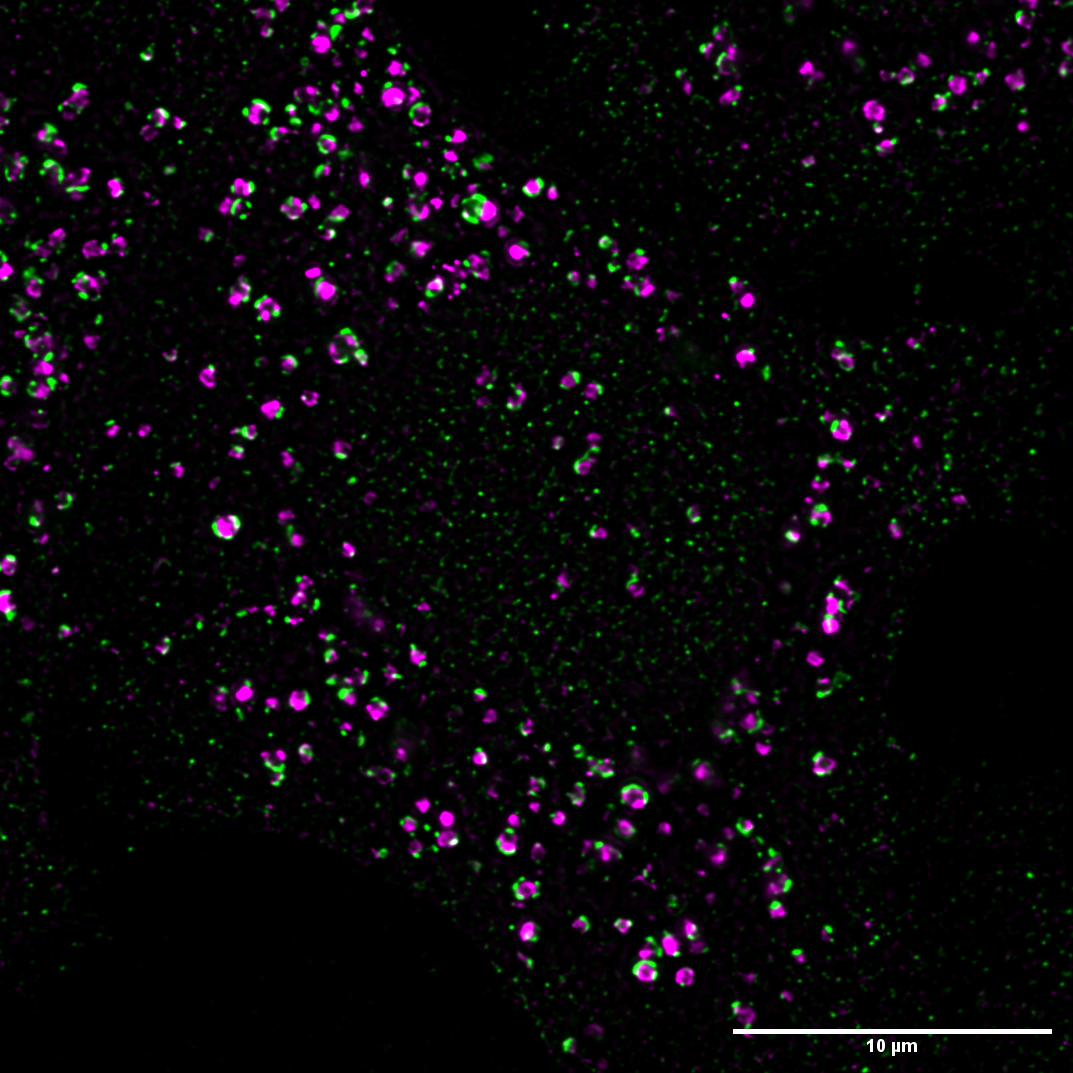

Supplement: Supplementary file 5 — Source data Fig. 3 [file 44318_2025_672_MOESM5_ESM.zip › Figure 3/3C/8KO_scale.tif]

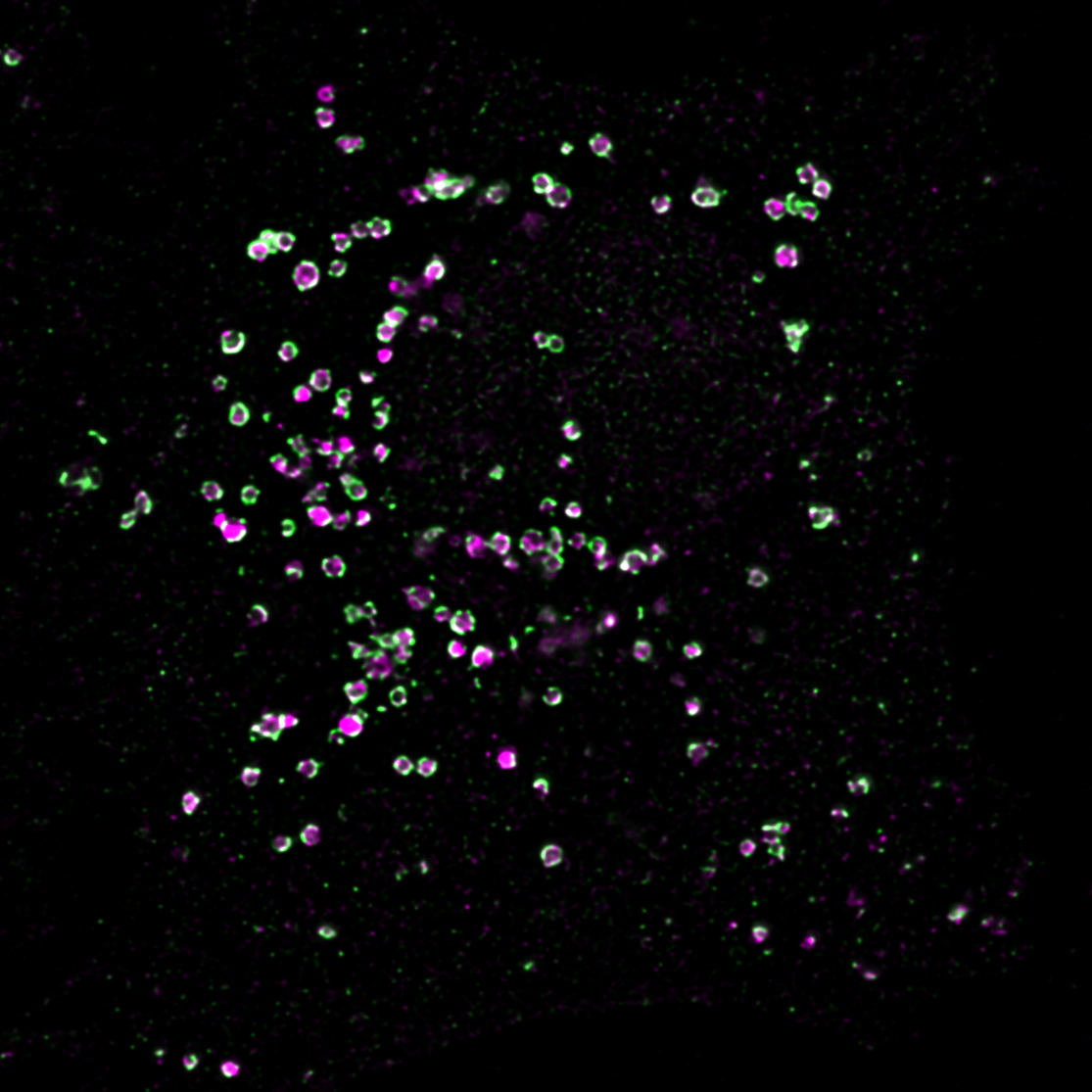

Supplement: Supplementary file 5 — Source data Fig. 3 [file 44318_2025_672_MOESM5_ESM.zip › Figure 3/3C/WT.tif]

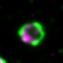

Supplement: Supplementary file 5 — Source data Fig. 3 [file 44318_2025_672_MOESM5_ESM.zip › Figure 3/3C/WT_a.tif]

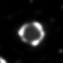

Supplement: Supplementary file 5 — Source data Fig. 3 [file 44318_2025_672_MOESM5_ESM.zip › Figure 3/3C/WT_a_CHMP.tif]

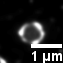

Supplement: Supplementary file 5 — Source data Fig. 3 [file 44318_2025_672_MOESM5_ESM.zip › Figure 3/3C/WT_a_scale.tif]

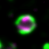

Supplement: Supplementary file 5 — Source data Fig. 3 [file 44318_2025_672_MOESM5_ESM.zip › Figure 3/3C/WT_b.tif]

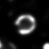

Supplement: Supplementary file 5 — Source data Fig. 3 [file 44318_2025_672_MOESM5_ESM.zip › Figure 3/3C/WT_b_CHMP.tif]

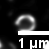

Supplement: Supplementary file 5 — Source data Fig. 3 [file 44318_2025_672_MOESM5_ESM.zip › Figure 3/3C/WT_b_scale.tif]

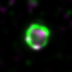

Supplement: Supplementary file 5 — Source data Fig. 3 [file 44318_2025_672_MOESM5_ESM.zip › Figure 3/3C/WT_c.tif]

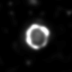

Supplement: Supplementary file 5 — Source data Fig. 3 [file 44318_2025_672_MOESM5_ESM.zip › Figure 3/3C/WT_c_CHMP.tif]

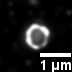

Supplement: Supplementary file 5 — Source data Fig. 3 [file 44318_2025_672_MOESM5_ESM.zip › Figure 3/3C/WT_c_scale.tif]

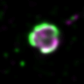

Supplement: Supplementary file 5 — Source data Fig. 3 [file 44318_2025_672_MOESM5_ESM.zip › Figure 3/3C/WT_d.tif]

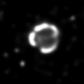

Supplement: Supplementary file 5 — Source data Fig. 3 [file 44318_2025_672_MOESM5_ESM.zip › Figure 3/3C/WT_d_CHMP.tif]

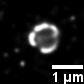

Supplement: Supplementary file 5 — Source data Fig. 3 [file 44318_2025_672_MOESM5_ESM.zip › Figure 3/3C/WT_d_scale.tif]

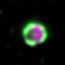

Supplement: Supplementary file 5 — Source data Fig. 3 [file 44318_2025_672_MOESM5_ESM.zip › Figure 3/3C/WT_e.tif]

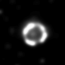

Supplement: Supplementary file 5 — Source data Fig. 3 [file 44318_2025_672_MOESM5_ESM.zip › Figure 3/3C/WT_e_CHMP.tif]

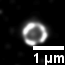

Supplement: Supplementary file 5 — Source data Fig. 3 [file 44318_2025_672_MOESM5_ESM.zip › Figure 3/3C/WT_e_CHMP_scale.tif]

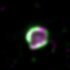

Supplement: Supplementary file 5 — Source data Fig. 3 [file 44318_2025_672_MOESM5_ESM.zip › Figure 3/3C/WT_f.tif]

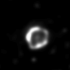

Supplement: Supplementary file 5 — Source data Fig. 3 [file 44318_2025_672_MOESM5_ESM.zip › Figure 3/3C/WT_f_CHMP.tif]

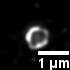

Supplement: Supplementary file 5 — Source data Fig. 3 [file 44318_2025_672_MOESM5_ESM.zip › Figure 3/3C/WT_f_scale.tif]

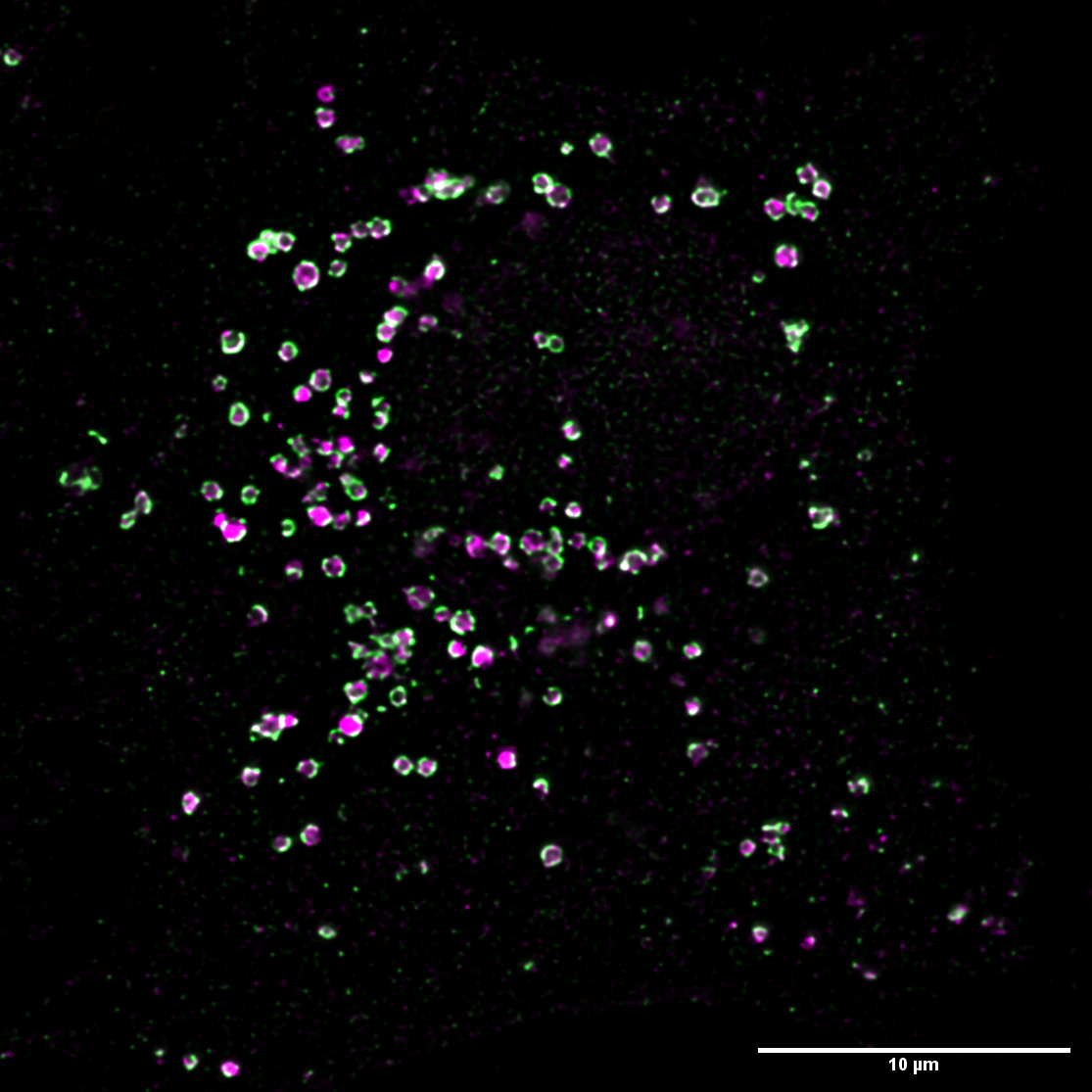

Supplement: Supplementary file 5 — Source data Fig. 3 [file 44318_2025_672_MOESM5_ESM.zip › Figure 3/3C/WT_scale.tif]

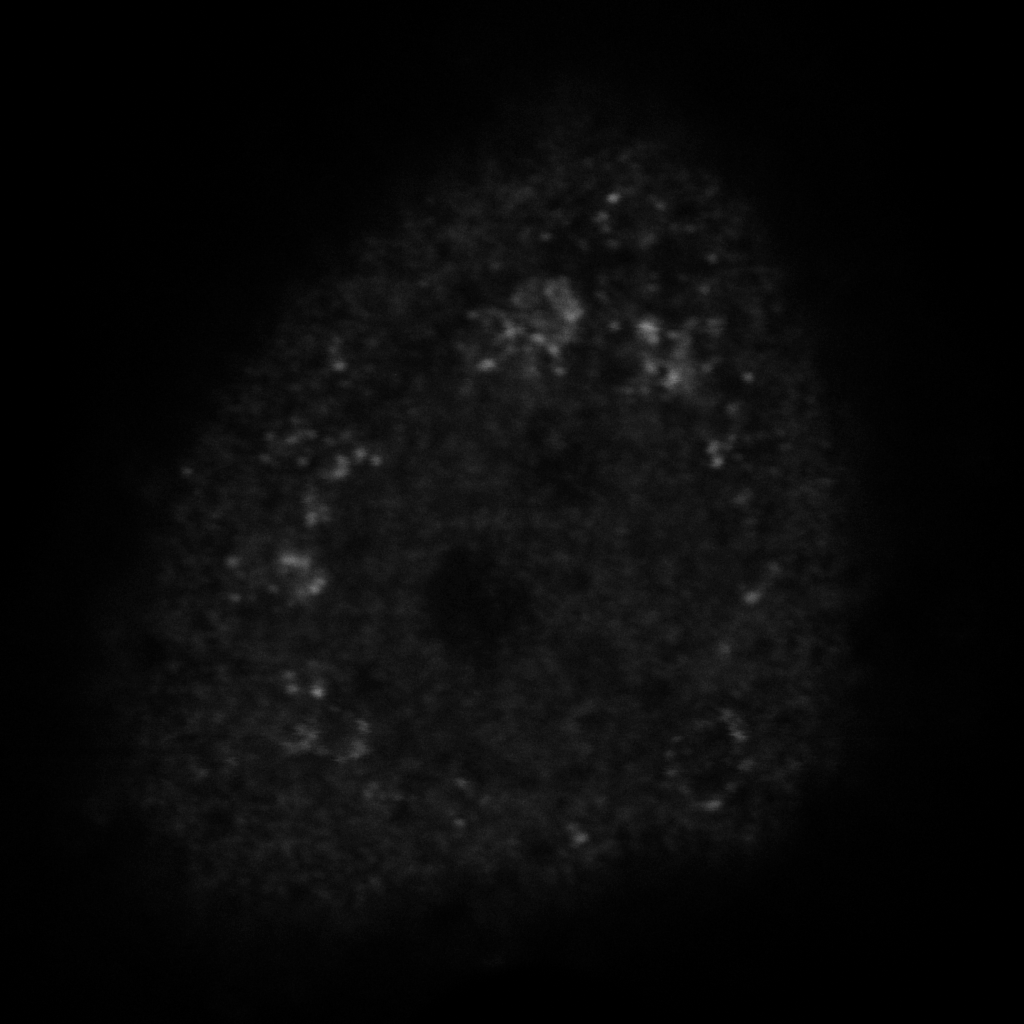

Supplement: Supplementary file 5 — Source data Fig. 3 [file 44318_2025_672_MOESM5_ESM.zip › Figure 3/3D/5KO_IST1.tif]

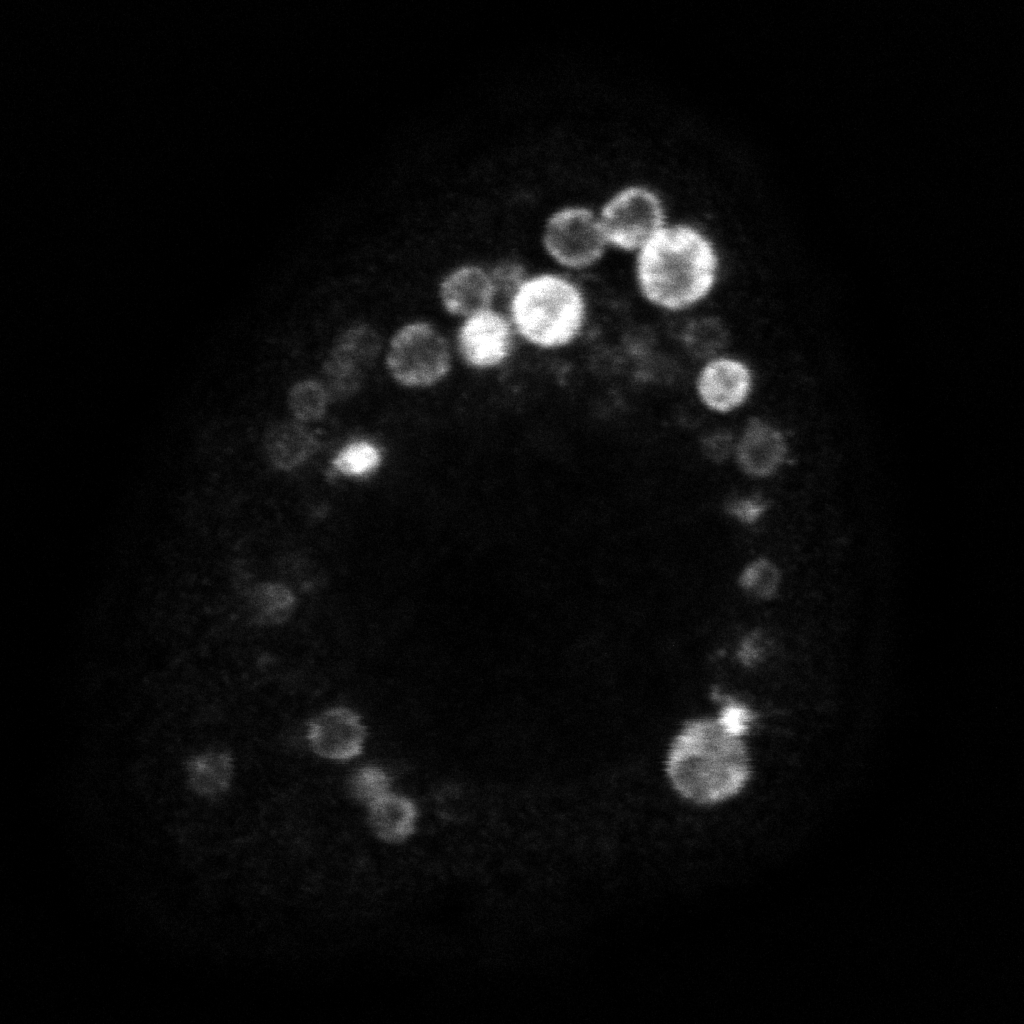

Supplement: Supplementary file 5 — Source data Fig. 3 [file 44318_2025_672_MOESM5_ESM.zip › Figure 3/3D/5KO_LAMP.tif]

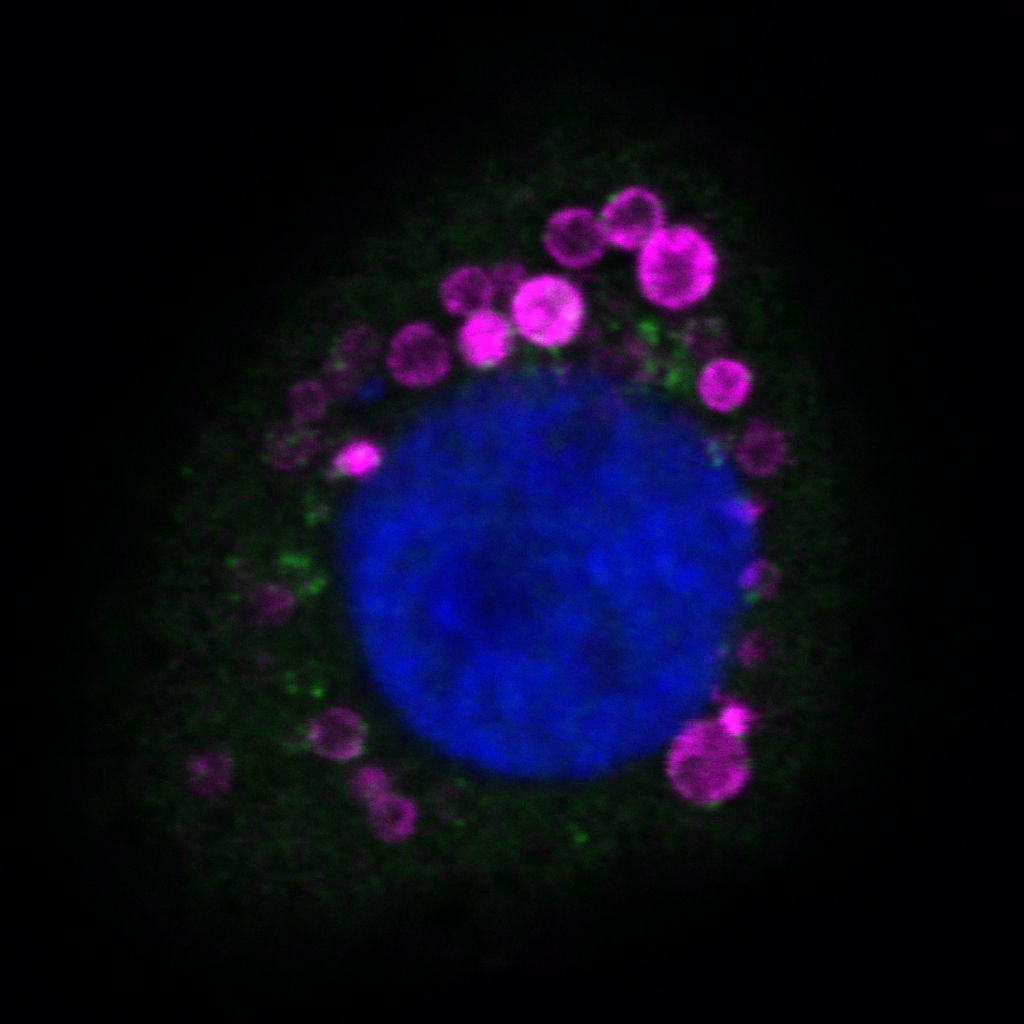

Supplement: Supplementary file 5 — Source data Fig. 3 [file 44318_2025_672_MOESM5_ESM.zip › Figure 3/3D/5KO_merge.tif]

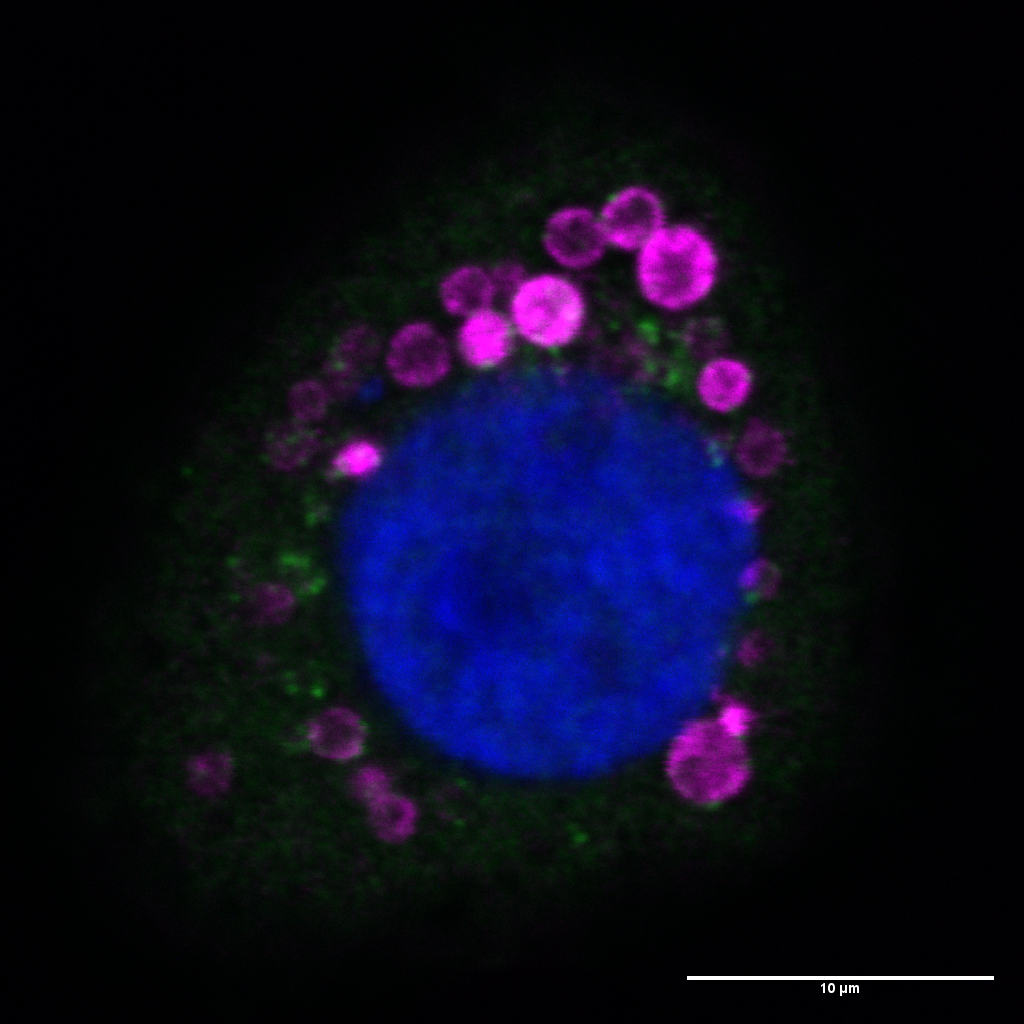

Supplement: Supplementary file 5 — Source data Fig. 3 [file 44318_2025_672_MOESM5_ESM.zip › Figure 3/3D/5KO_scale.tif]

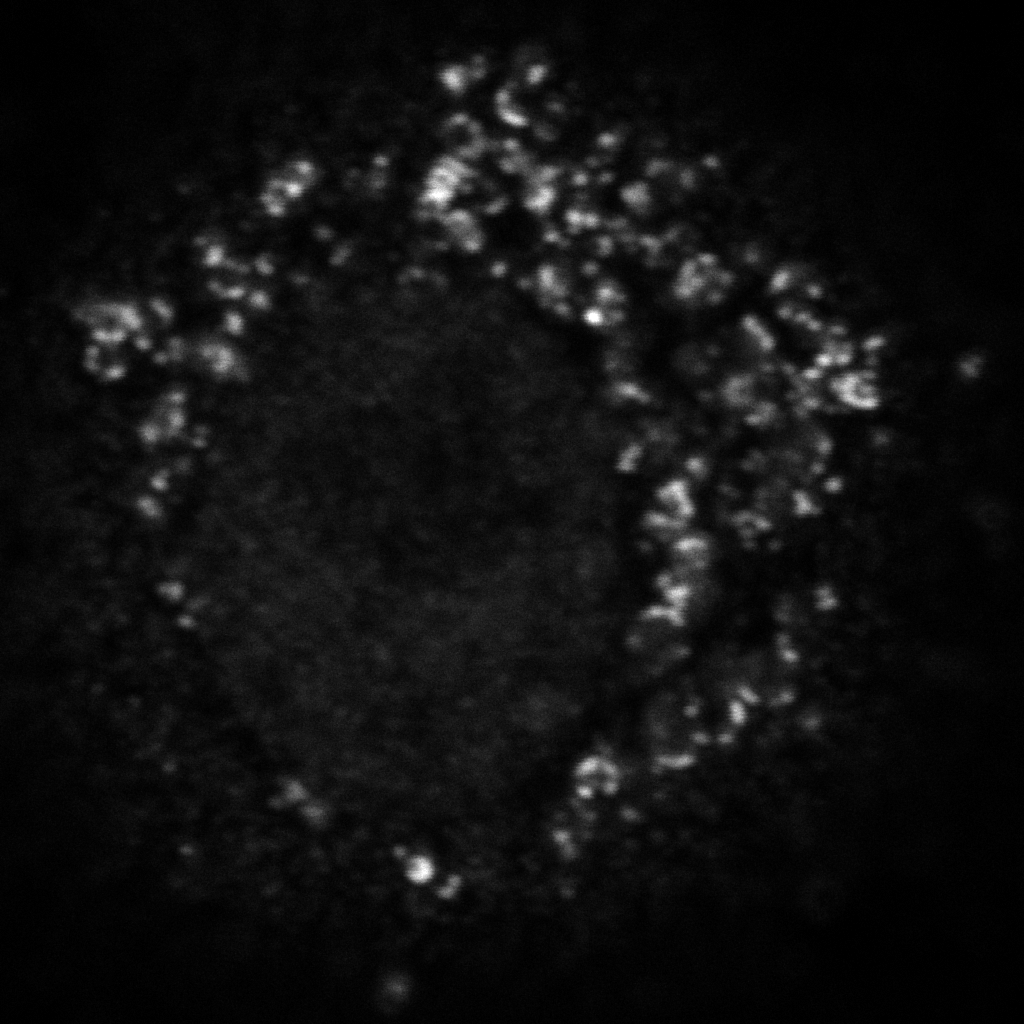

Supplement: Supplementary file 5 — Source data Fig. 3 [file 44318_2025_672_MOESM5_ESM.zip › Figure 3/3D/8KO_IST1.tif]

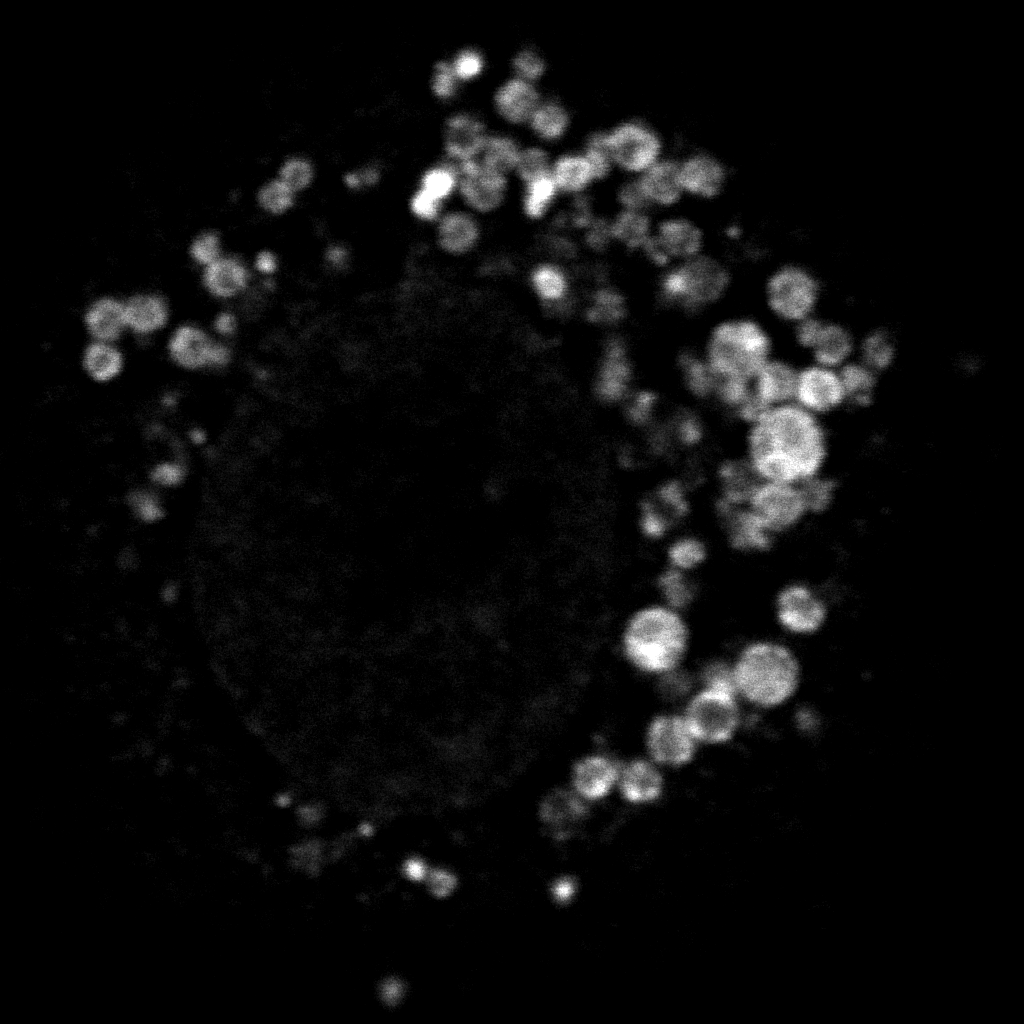

Supplement: Supplementary file 5 — Source data Fig. 3 [file 44318_2025_672_MOESM5_ESM.zip › Figure 3/3D/8KO_LAMP.tif]

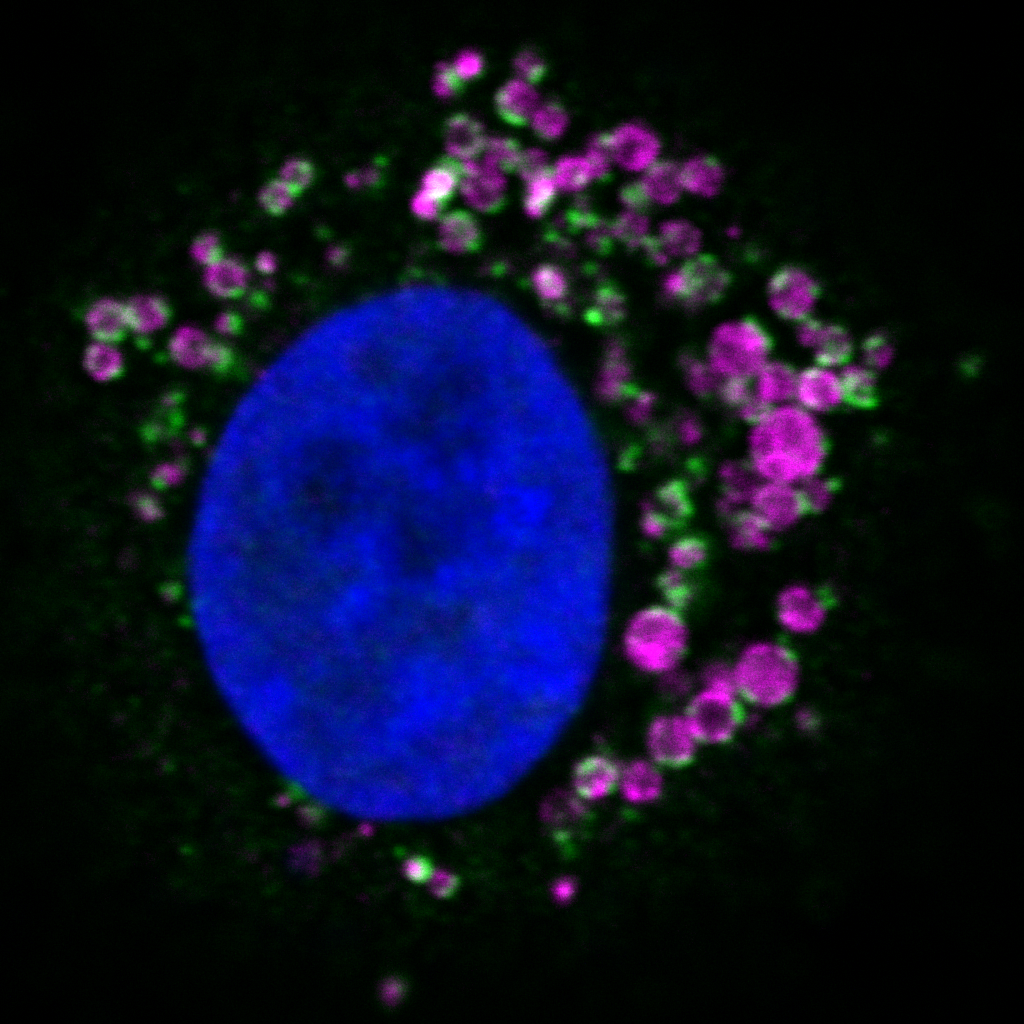

Supplement: Supplementary file 5 — Source data Fig. 3 [file 44318_2025_672_MOESM5_ESM.zip › Figure 3/3D/8KO_merge.tif]

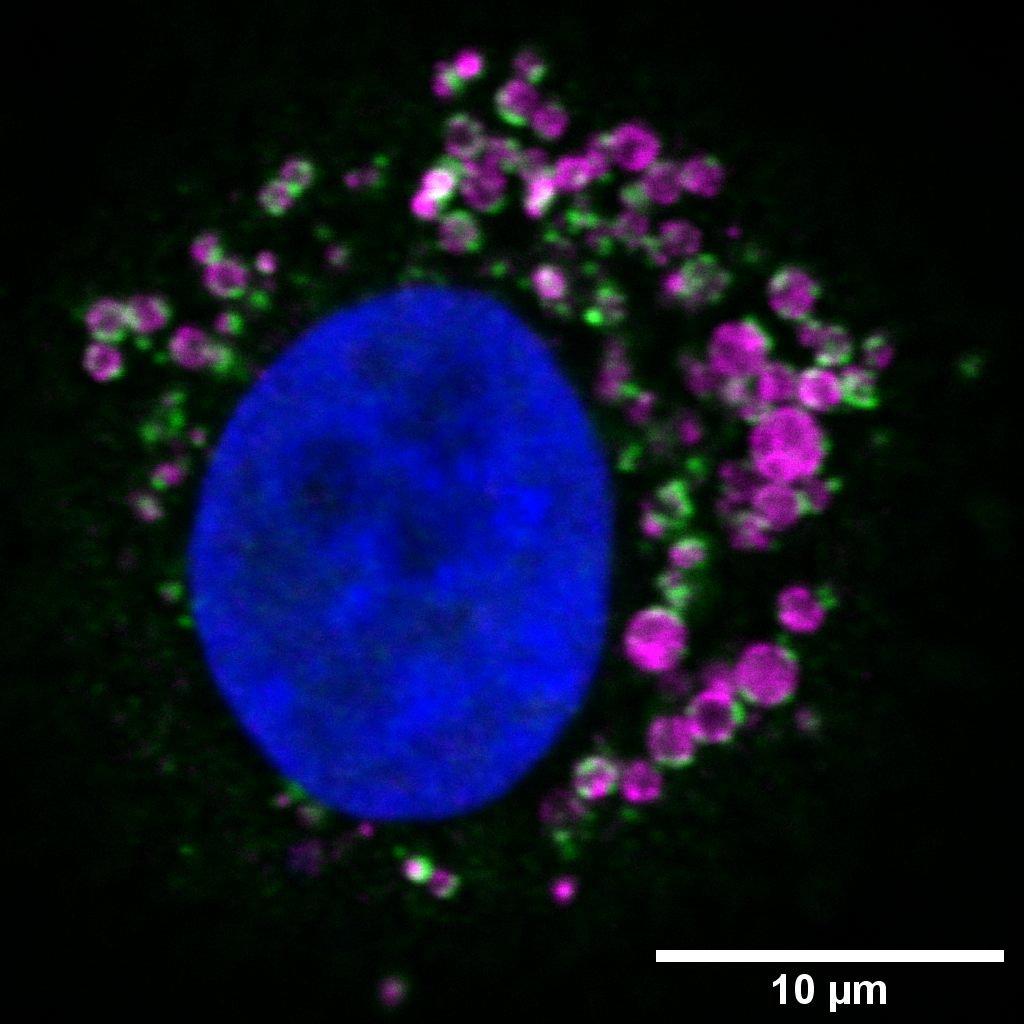

Supplement: Supplementary file 5 — Source data Fig. 3 [file 44318_2025_672_MOESM5_ESM.zip › Figure 3/3D/8KO_scale.tif]

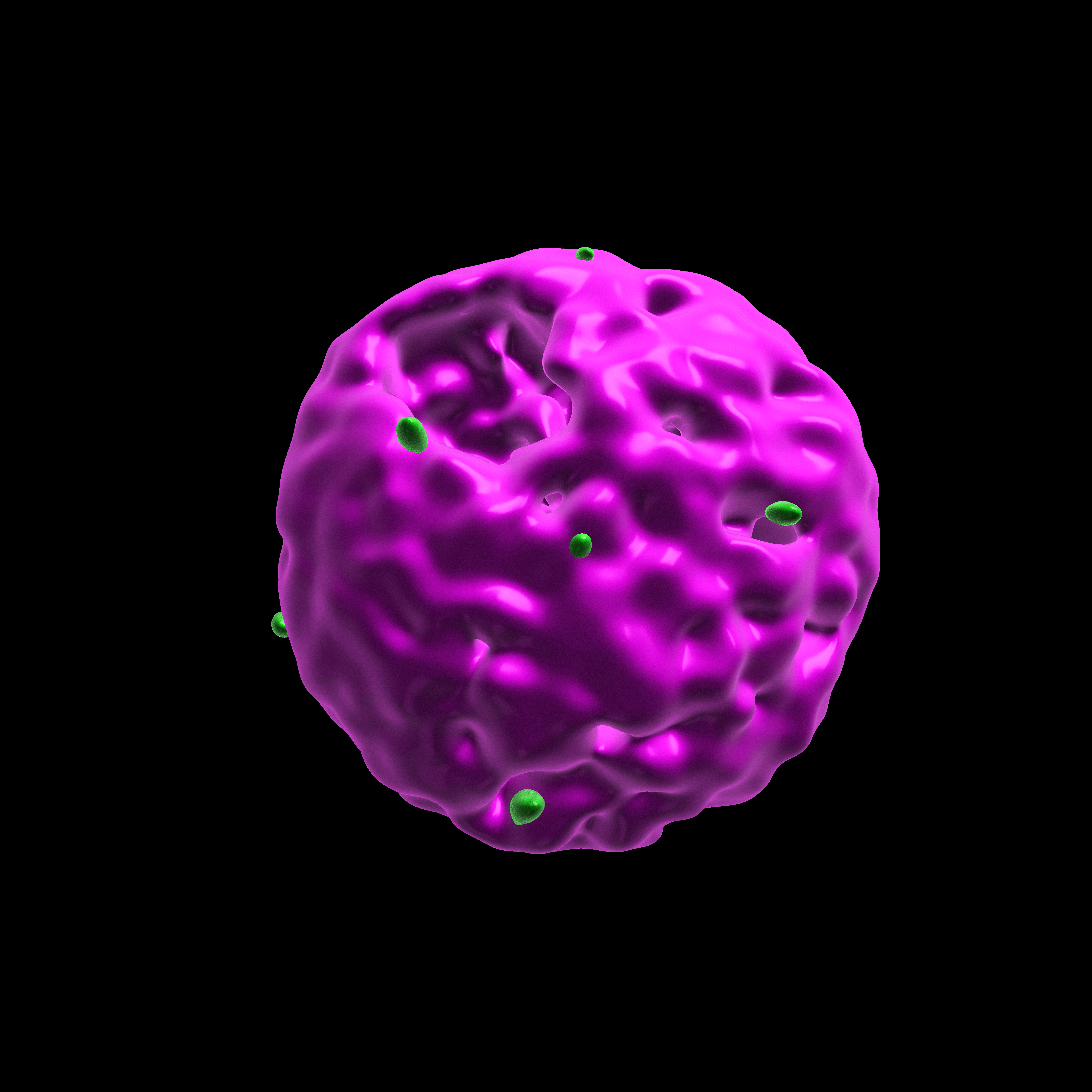

Supplement: Supplementary file 5 — Source data Fig. 3 [file 44318_2025_672_MOESM5_ESM.zip › Figure 3/3D/Individual Vesicles/5KO/5KO_3D.tif]

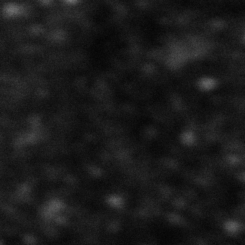

Supplement: Supplementary file 5 — Source data Fig. 3 [file 44318_2025_672_MOESM5_ESM.zip › Figure 3/3D/Individual Vesicles/5KO/a_IST1.tif]

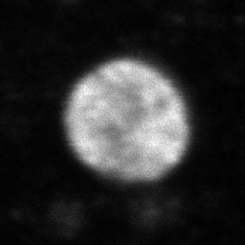

Supplement: Supplementary file 5 — Source data Fig. 3 [file 44318_2025_672_MOESM5_ESM.zip › Figure 3/3D/Individual Vesicles/5KO/a_LAMP.tif]

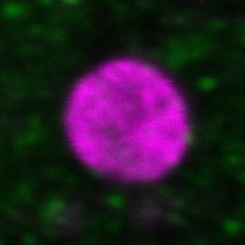

Supplement: Supplementary file 5 — Source data Fig. 3 [file 44318_2025_672_MOESM5_ESM.zip › Figure 3/3D/Individual Vesicles/5KO/a_merge.tif]

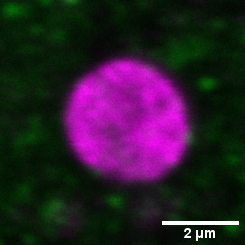

Supplement: Supplementary file 5 — Source data Fig. 3 [file 44318_2025_672_MOESM5_ESM.zip › Figure 3/3D/Individual Vesicles/5KO/a_scale.tif]

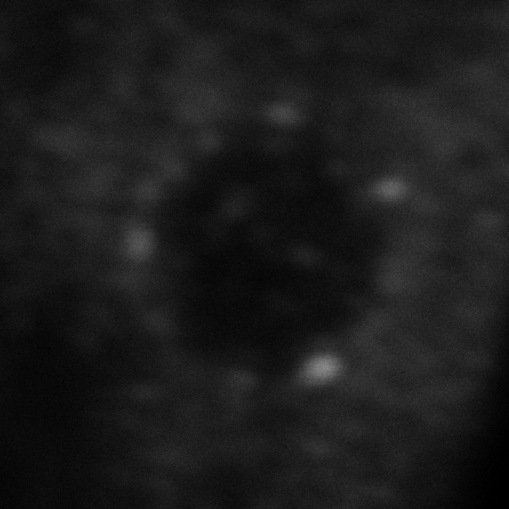

Supplement: Supplementary file 5 — Source data Fig. 3 [file 44318_2025_672_MOESM5_ESM.zip › Figure 3/3D/Individual Vesicles/5KO/b_IST1.tif]

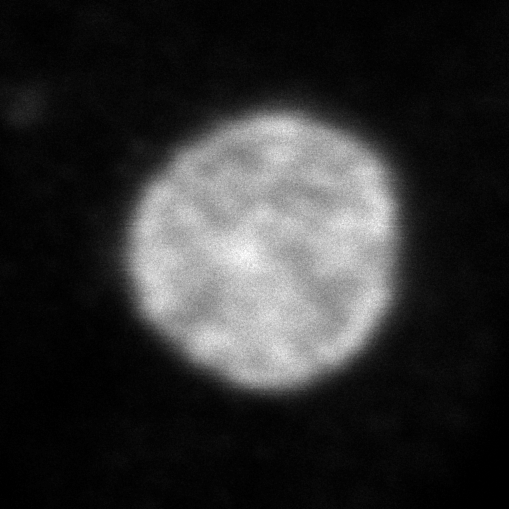

Supplement: Supplementary file 5 — Source data Fig. 3 [file 44318_2025_672_MOESM5_ESM.zip › Figure 3/3D/Individual Vesicles/5KO/b_LAMP.tif]

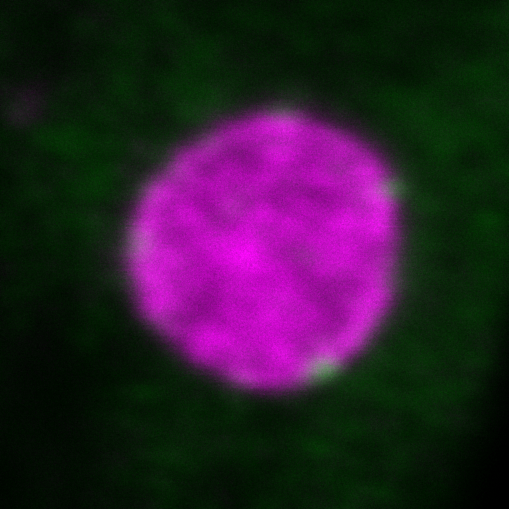

Supplement: Supplementary file 5 — Source data Fig. 3 [file 44318_2025_672_MOESM5_ESM.zip › Figure 3/3D/Individual Vesicles/5KO/b_merge.tif]

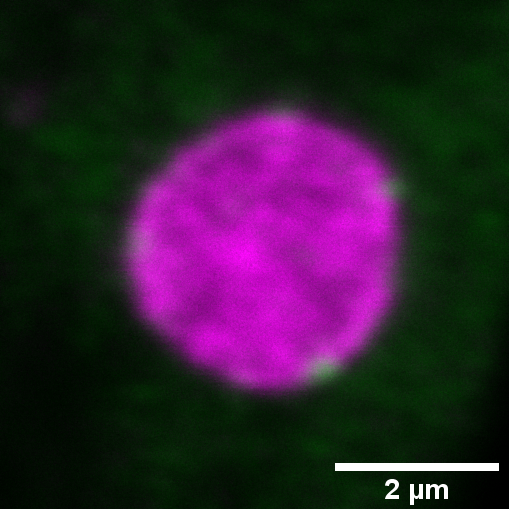

Supplement: Supplementary file 5 — Source data Fig. 3 [file 44318_2025_672_MOESM5_ESM.zip › Figure 3/3D/Individual Vesicles/5KO/b_scale.tif]

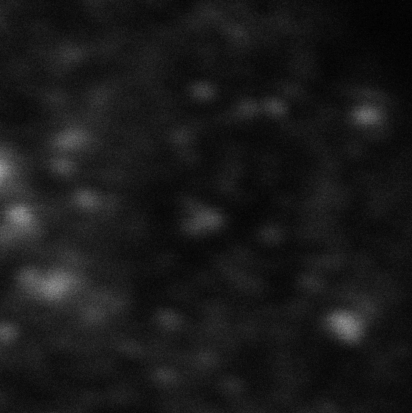

Supplement: Supplementary file 5 — Source data Fig. 3 [file 44318_2025_672_MOESM5_ESM.zip › Figure 3/3D/Individual Vesicles/5KO/c_IST1.tif]

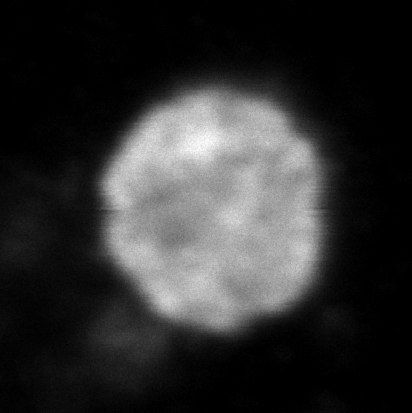

Supplement: Supplementary file 5 — Source data Fig. 3 [file 44318_2025_672_MOESM5_ESM.zip › Figure 3/3D/Individual Vesicles/5KO/c_LAMP.tif]

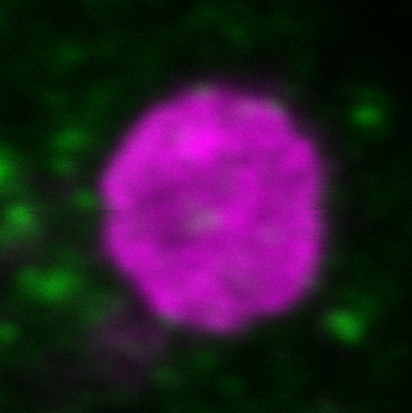

Supplement: Supplementary file 5 — Source data Fig. 3 [file 44318_2025_672_MOESM5_ESM.zip › Figure 3/3D/Individual Vesicles/5KO/c_merge.tif]

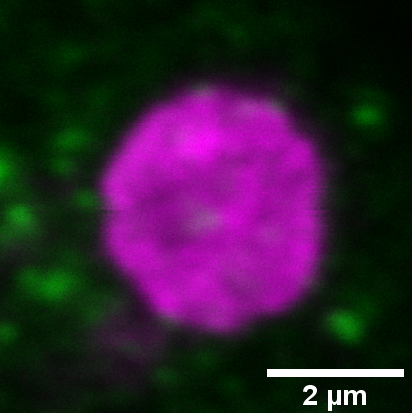

Supplement: Supplementary file 5 — Source data Fig. 3 [file 44318_2025_672_MOESM5_ESM.zip › Figure 3/3D/Individual Vesicles/5KO/c_scale.tif]

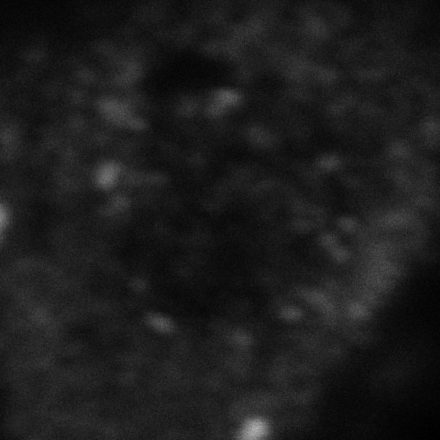

Supplement: Supplementary file 5 — Source data Fig. 3 [file 44318_2025_672_MOESM5_ESM.zip › Figure 3/3D/Individual Vesicles/5KO/d_IST1.tif]

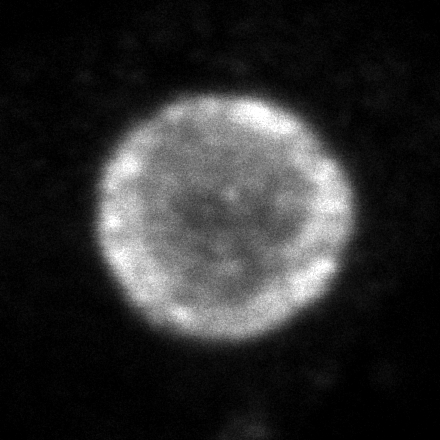

Supplement: Supplementary file 5 — Source data Fig. 3 [file 44318_2025_672_MOESM5_ESM.zip › Figure 3/3D/Individual Vesicles/5KO/d_LAMP.tif]

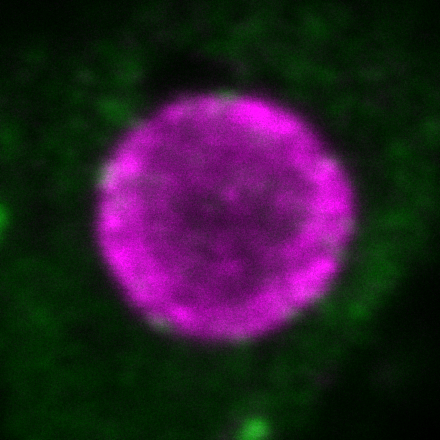

Supplement: Supplementary file 5 — Source data Fig. 3 [file 44318_2025_672_MOESM5_ESM.zip › Figure 3/3D/Individual Vesicles/5KO/d_merge.tif]

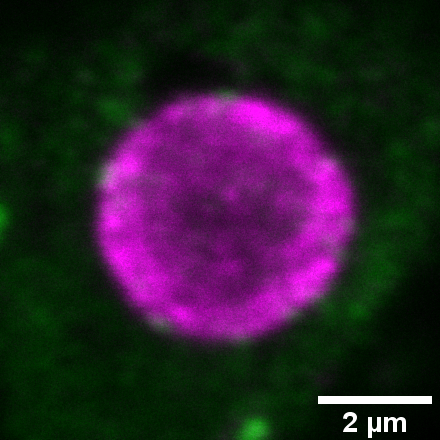

Supplement: Supplementary file 5 — Source data Fig. 3 [file 44318_2025_672_MOESM5_ESM.zip › Figure 3/3D/Individual Vesicles/5KO/d_scale.tif]

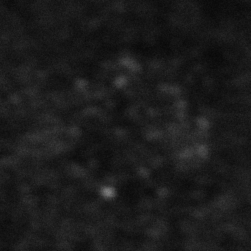

Supplement: Supplementary file 5 — Source data Fig. 3 [file 44318_2025_672_MOESM5_ESM.zip › Figure 3/3D/Individual Vesicles/5KO/e_IST1.tif]

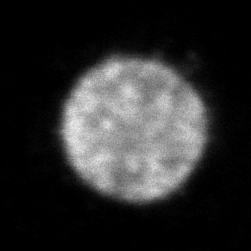

Supplement: Supplementary file 5 — Source data Fig. 3 [file 44318_2025_672_MOESM5_ESM.zip › Figure 3/3D/Individual Vesicles/5KO/e_LAMP.tif]

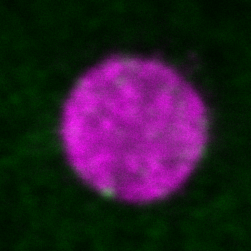

Supplement: Supplementary file 5 — Source data Fig. 3 [file 44318_2025_672_MOESM5_ESM.zip › Figure 3/3D/Individual Vesicles/5KO/e_merge.tif]

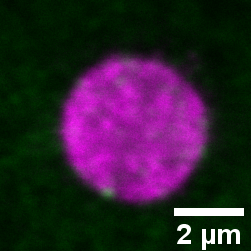

Supplement: Supplementary file 5 — Source data Fig. 3 [file 44318_2025_672_MOESM5_ESM.zip › Figure 3/3D/Individual Vesicles/5KO/e_scale.tif]

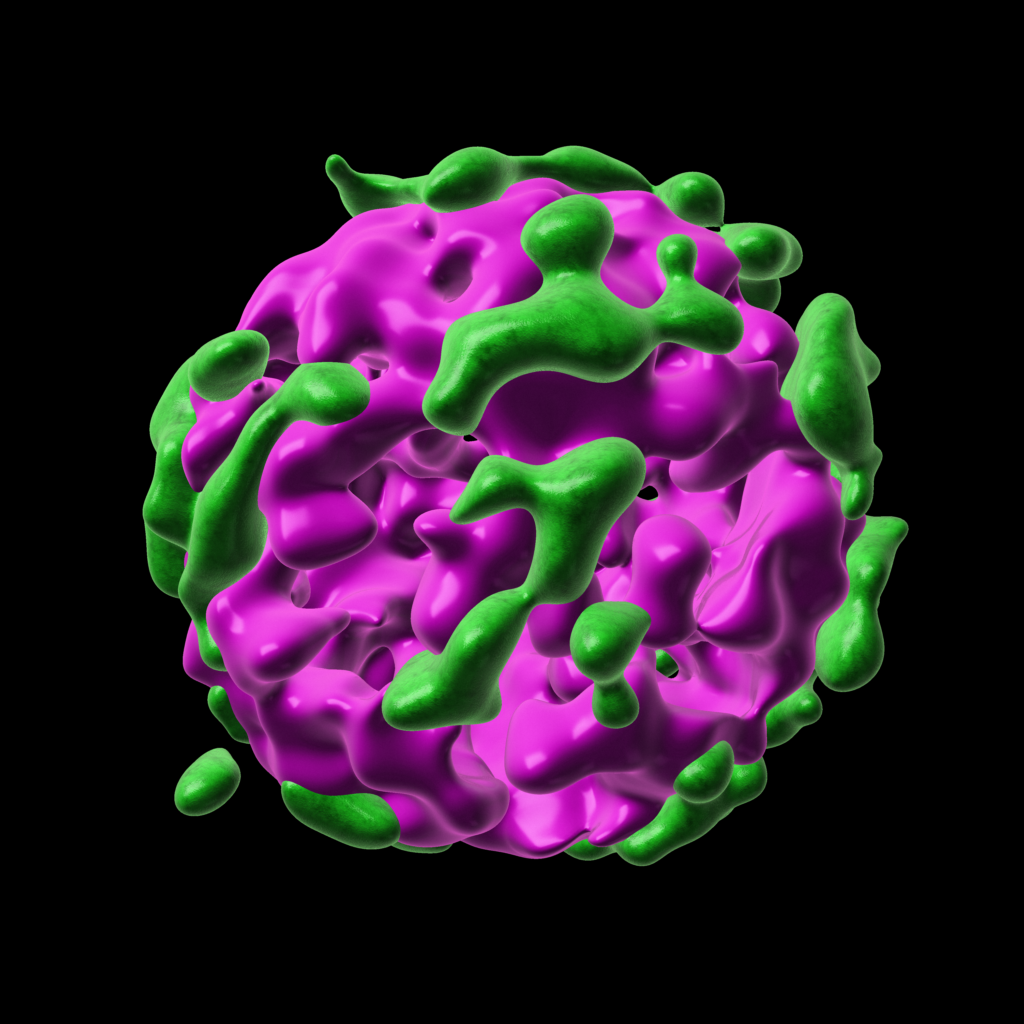

Supplement: Supplementary file 5 — Source data Fig. 3 [file 44318_2025_672_MOESM5_ESM.zip › Figure 3/3D/Individual Vesicles/8KO/8KO_3D.tif]

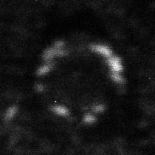

Supplement: Supplementary file 5 — Source data Fig. 3 [file 44318_2025_672_MOESM5_ESM.zip › Figure 3/3D/Individual Vesicles/8KO/a_IST1.tif]

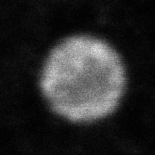

Supplement: Supplementary file 5 — Source data Fig. 3 [file 44318_2025_672_MOESM5_ESM.zip › Figure 3/3D/Individual Vesicles/8KO/a_LAMP.tif]

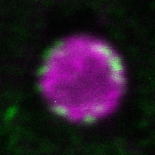

Supplement: Supplementary file 5 — Source data Fig. 3 [file 44318_2025_672_MOESM5_ESM.zip › Figure 3/3D/Individual Vesicles/8KO/a_merge.tif]

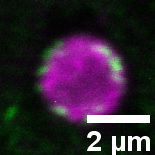

Supplement: Supplementary file 5 — Source data Fig. 3 [file 44318_2025_672_MOESM5_ESM.zip › Figure 3/3D/Individual Vesicles/8KO/a_scale.tif]

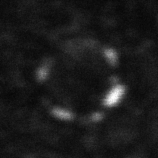

Supplement: Supplementary file 5 — Source data Fig. 3 [file 44318_2025_672_MOESM5_ESM.zip › Figure 3/3D/Individual Vesicles/8KO/b_IST1.tif]

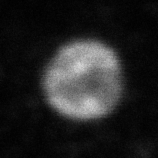

Supplement: Supplementary file 5 — Source data Fig. 3 [file 44318_2025_672_MOESM5_ESM.zip › Figure 3/3D/Individual Vesicles/8KO/b_LAMP.tif]

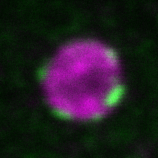

Supplement: Supplementary file 5 — Source data Fig. 3 [file 44318_2025_672_MOESM5_ESM.zip › Figure 3/3D/Individual Vesicles/8KO/b_merge.tif]

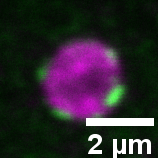

Supplement: Supplementary file 5 — Source data Fig. 3 [file 44318_2025_672_MOESM5_ESM.zip › Figure 3/3D/Individual Vesicles/8KO/b_scale.tif]

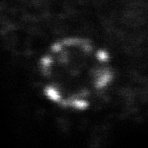

Supplement: Supplementary file 5 — Source data Fig. 3 [file 44318_2025_672_MOESM5_ESM.zip › Figure 3/3D/Individual Vesicles/8KO/c_IST1.tif]

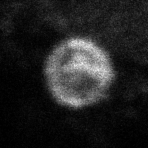

Supplement: Supplementary file 5 — Source data Fig. 3 [file 44318_2025_672_MOESM5_ESM.zip › Figure 3/3D/Individual Vesicles/8KO/c_LAMP.tif]

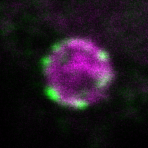

Supplement: Supplementary file 5 — Source data Fig. 3 [file 44318_2025_672_MOESM5_ESM.zip › Figure 3/3D/Individual Vesicles/8KO/c_merge.tif]

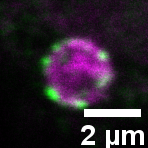

Supplement: Supplementary file 5 — Source data Fig. 3 [file 44318_2025_672_MOESM5_ESM.zip › Figure 3/3D/Individual Vesicles/8KO/c_scale.tif]

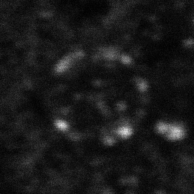

Supplement: Supplementary file 5 — Source data Fig. 3 [file 44318_2025_672_MOESM5_ESM.zip › Figure 3/3D/Individual Vesicles/8KO/d_IST1.tif]

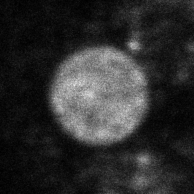

Supplement: Supplementary file 5 — Source data Fig. 3 [file 44318_2025_672_MOESM5_ESM.zip › Figure 3/3D/Individual Vesicles/8KO/d_LAMP.tif]

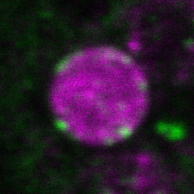

Supplement: Supplementary file 5 — Source data Fig. 3 [file 44318_2025_672_MOESM5_ESM.zip › Figure 3/3D/Individual Vesicles/8KO/d_merge.tif]

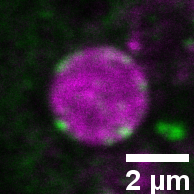

Supplement: Supplementary file 5 — Source data Fig. 3 [file 44318_2025_672_MOESM5_ESM.zip › Figure 3/3D/Individual Vesicles/8KO/d_scale.tif]

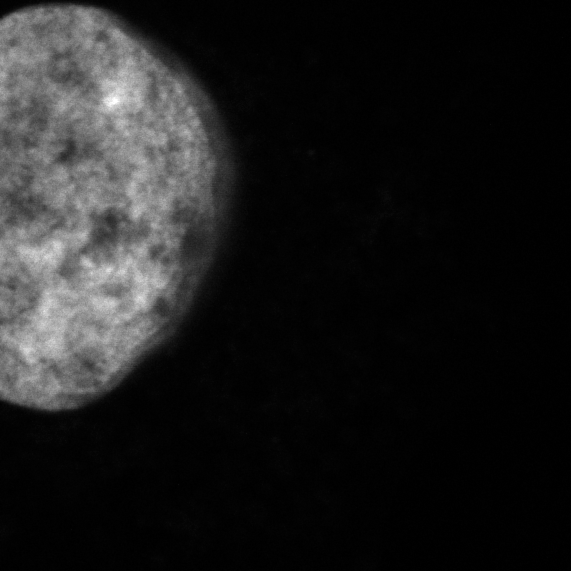

Supplement: Supplementary file 5 — Source data Fig. 3 [file 44318_2025_672_MOESM5_ESM.zip › Figure 3/3D/Individual Vesicles/8KO/DAPI.tif]

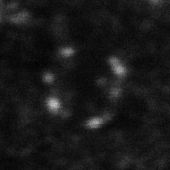

Supplement: Supplementary file 5 — Source data Fig. 3 [file 44318_2025_672_MOESM5_ESM.zip › Figure 3/3D/Individual Vesicles/8KO/e_IST1.tif]

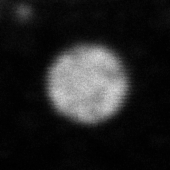

Supplement: Supplementary file 5 — Source data Fig. 3 [file 44318_2025_672_MOESM5_ESM.zip › Figure 3/3D/Individual Vesicles/8KO/e_LAMP.tif]

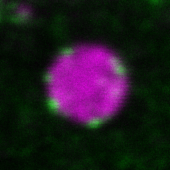

Supplement: Supplementary file 5 — Source data Fig. 3 [file 44318_2025_672_MOESM5_ESM.zip › Figure 3/3D/Individual Vesicles/8KO/e_merge.tif]

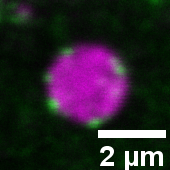

Supplement: Supplementary file 5 — Source data Fig. 3 [file 44318_2025_672_MOESM5_ESM.zip › Figure 3/3D/Individual Vesicles/8KO/e_scale.tif]
