# Supplementary figures and images for: The ATG8 E3-like ligases sense lysosomal damage and initiate ESCRT-mediated membrane repair (part 5 of 7)
Source: EMBO J. 2026 Jan 3;45(3):930–52. doi: 10.1038/s44318-025-00672-1 (PMC12865045; doi:10.1038/s44318-025-00672-1)

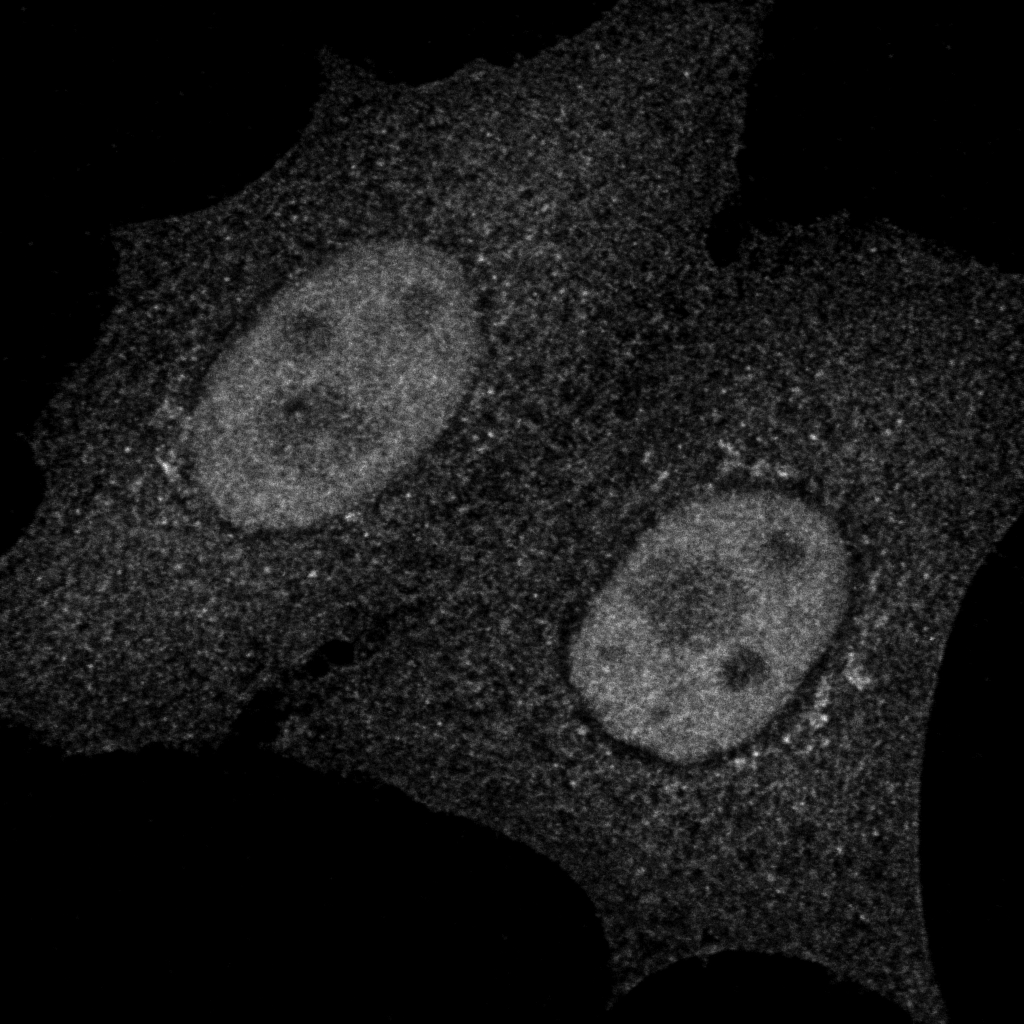

Supplement: Supplementary file 7 — Source data Fig. 5 [file 44318_2025_672_MOESM7_ESM.zip › Figure 5/5C/K130R_VEH_ALG2.tif]

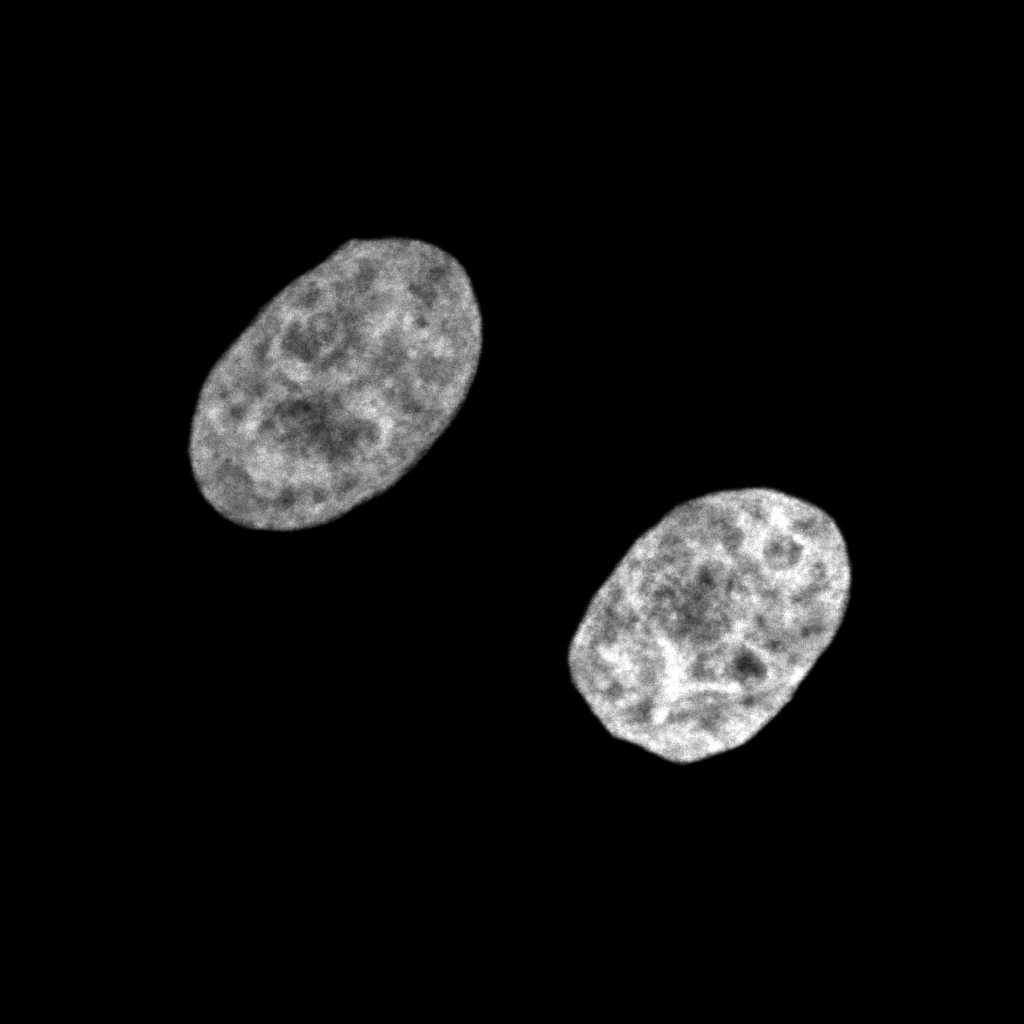

Supplement: Supplementary file 7 — Source data Fig. 5 [file 44318_2025_672_MOESM7_ESM.zip › Figure 5/5C/K130R_VEH_DAPI.tif]

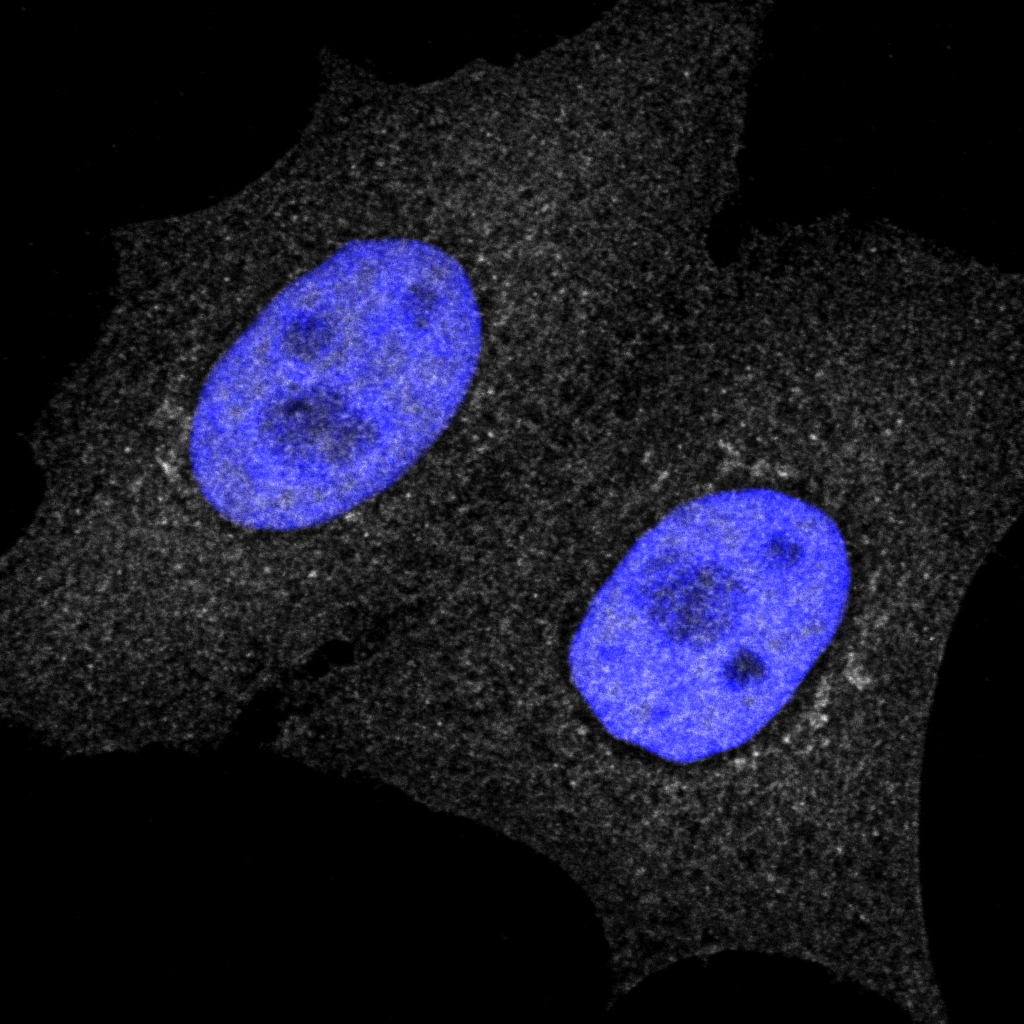

Supplement: Supplementary file 7 — Source data Fig. 5 [file 44318_2025_672_MOESM7_ESM.zip › Figure 5/5C/K130R_VEH_merge.tif]

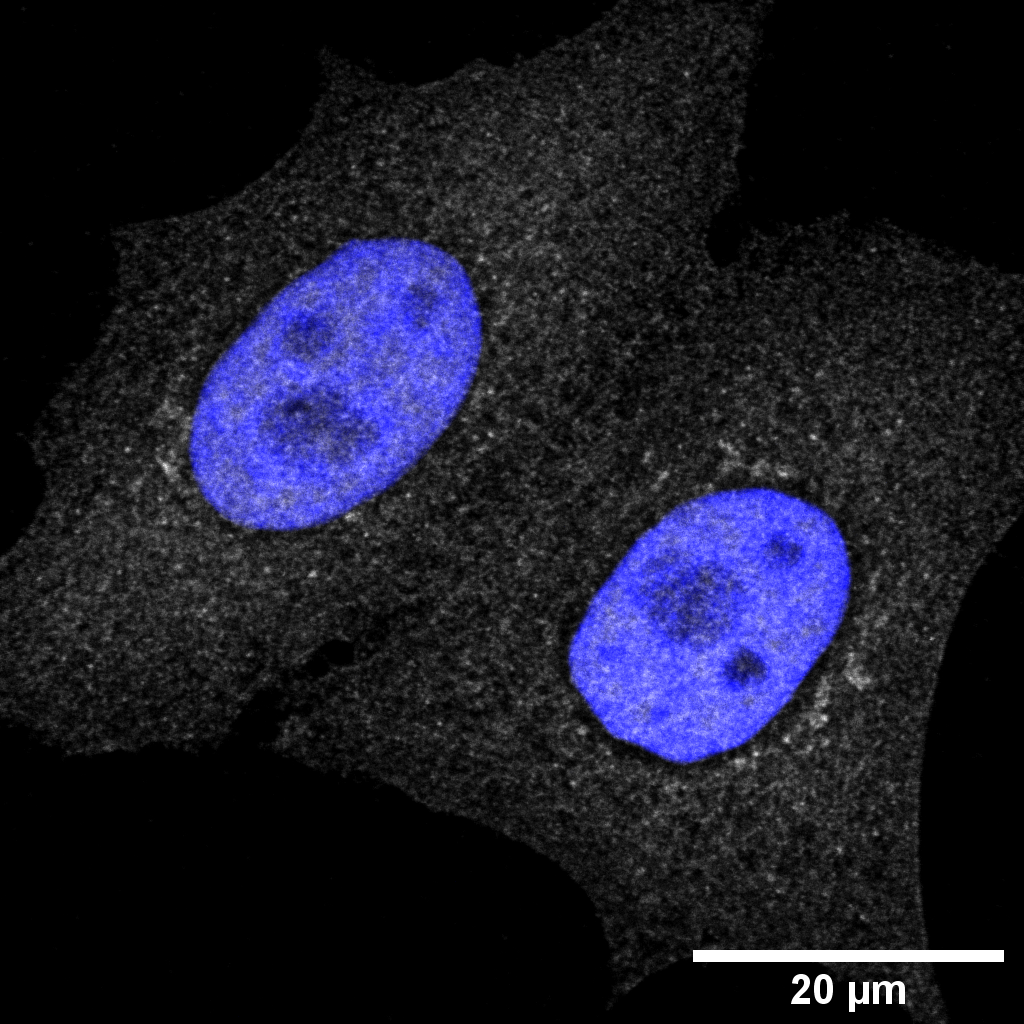

Supplement: Supplementary file 7 — Source data Fig. 5 [file 44318_2025_672_MOESM7_ESM.zip › Figure 5/5C/K130R_VEH_scale.tif]

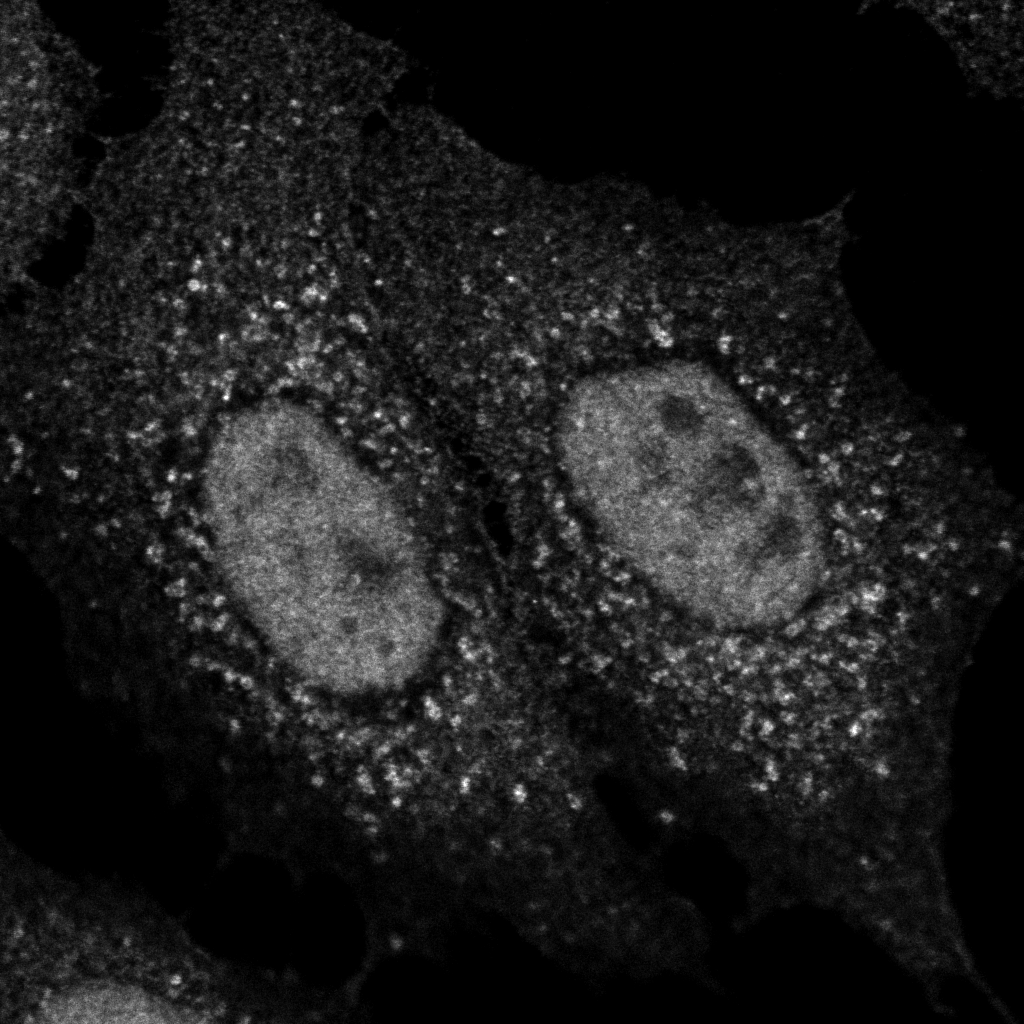

Supplement: Supplementary file 7 — Source data Fig. 5 [file 44318_2025_672_MOESM7_ESM.zip › Figure 5/5C/WT_LLOMe_ALG2.tif]

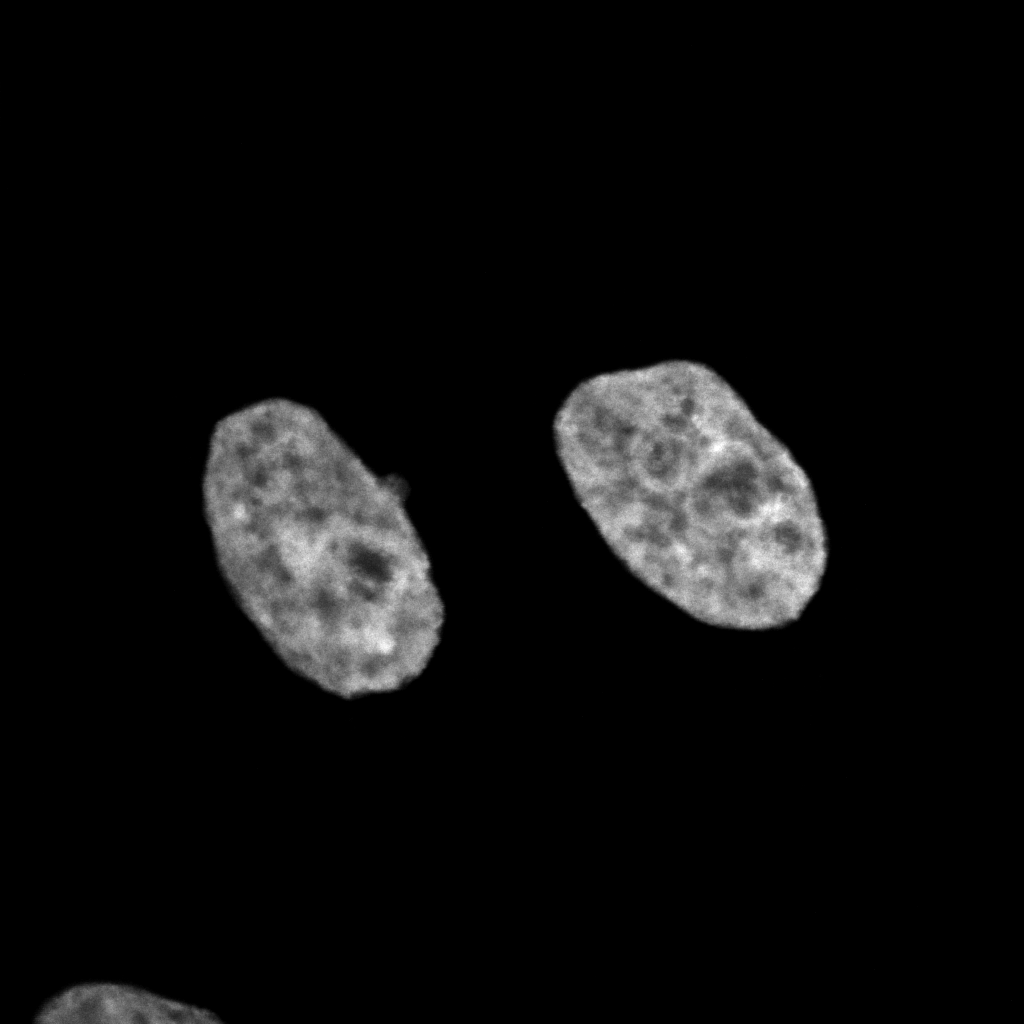

Supplement: Supplementary file 7 — Source data Fig. 5 [file 44318_2025_672_MOESM7_ESM.zip › Figure 5/5C/WT_LLOMe_DAPI.tif]

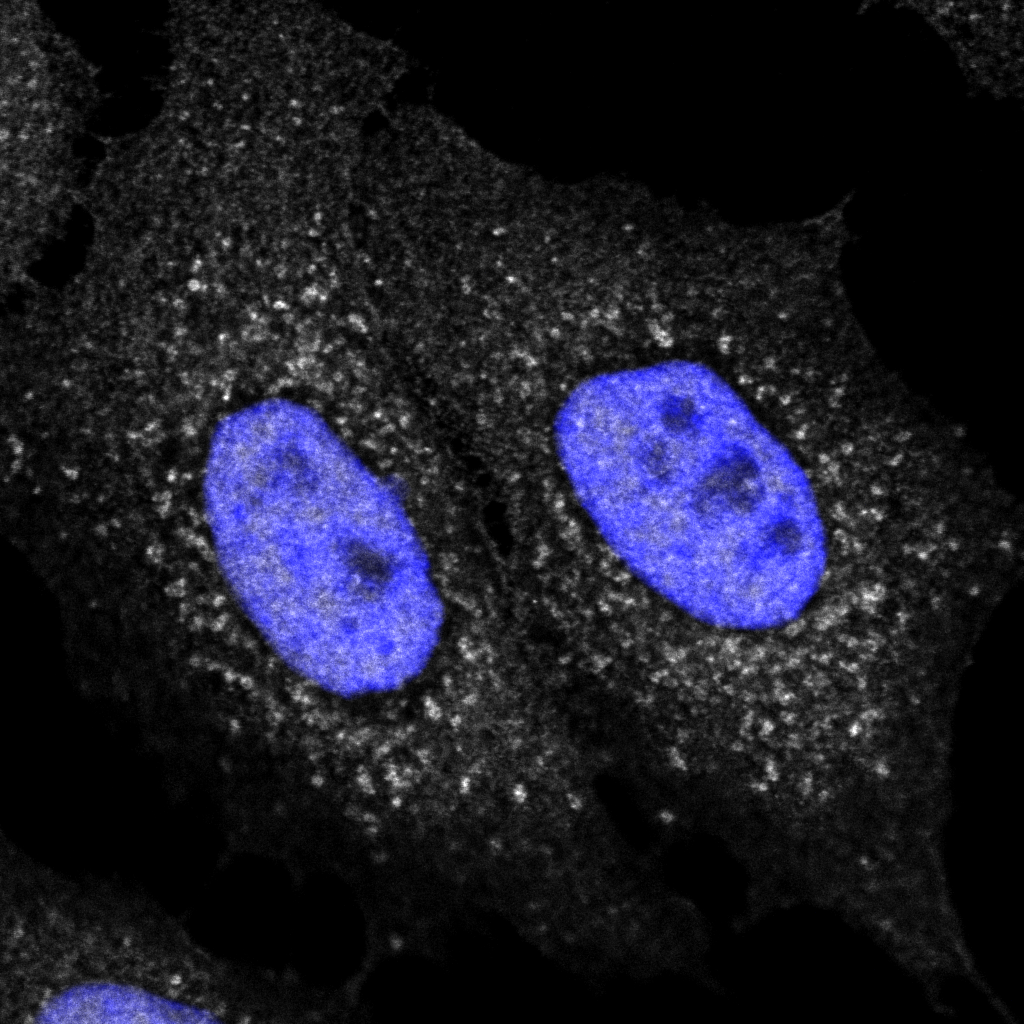

Supplement: Supplementary file 7 — Source data Fig. 5 [file 44318_2025_672_MOESM7_ESM.zip › Figure 5/5C/WT_LLOMe_merge.tif]

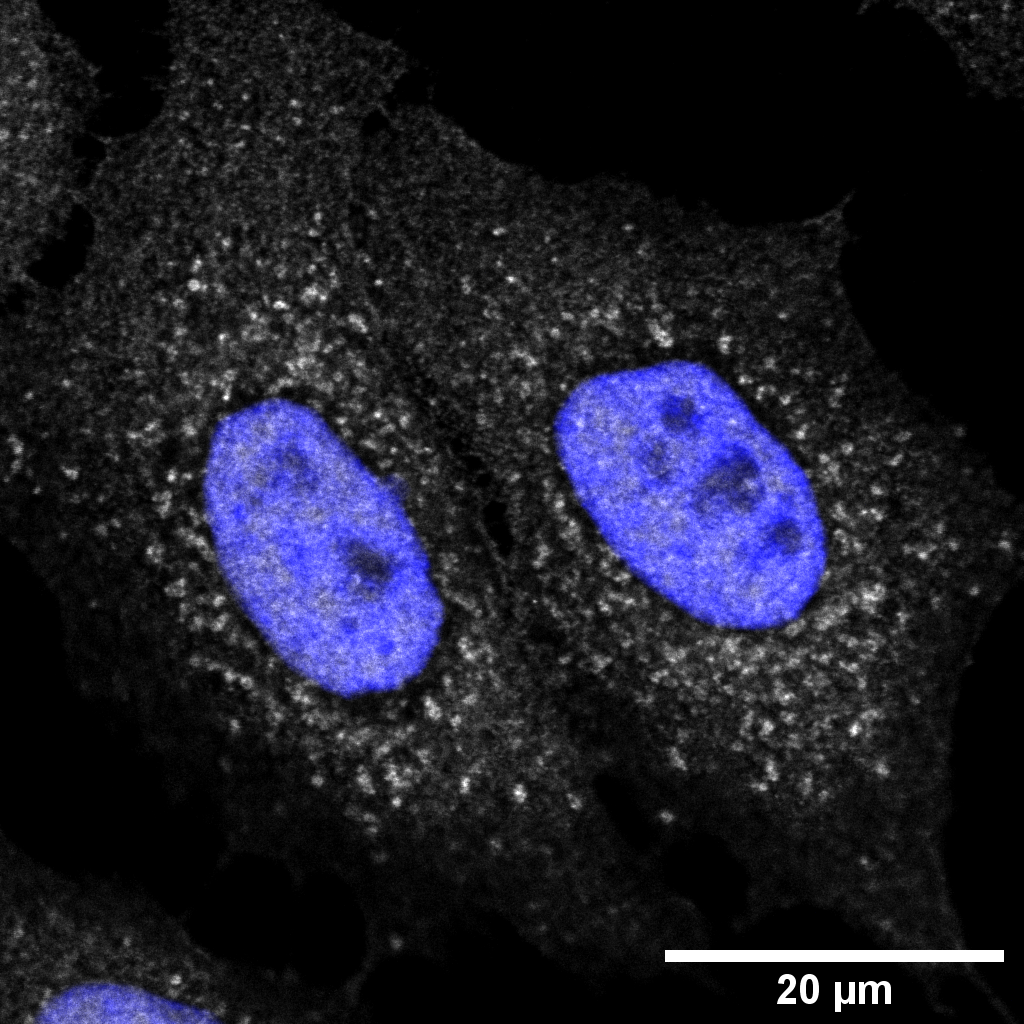

Supplement: Supplementary file 7 — Source data Fig. 5 [file 44318_2025_672_MOESM7_ESM.zip › Figure 5/5C/WT_LLOMe_scale.tif]

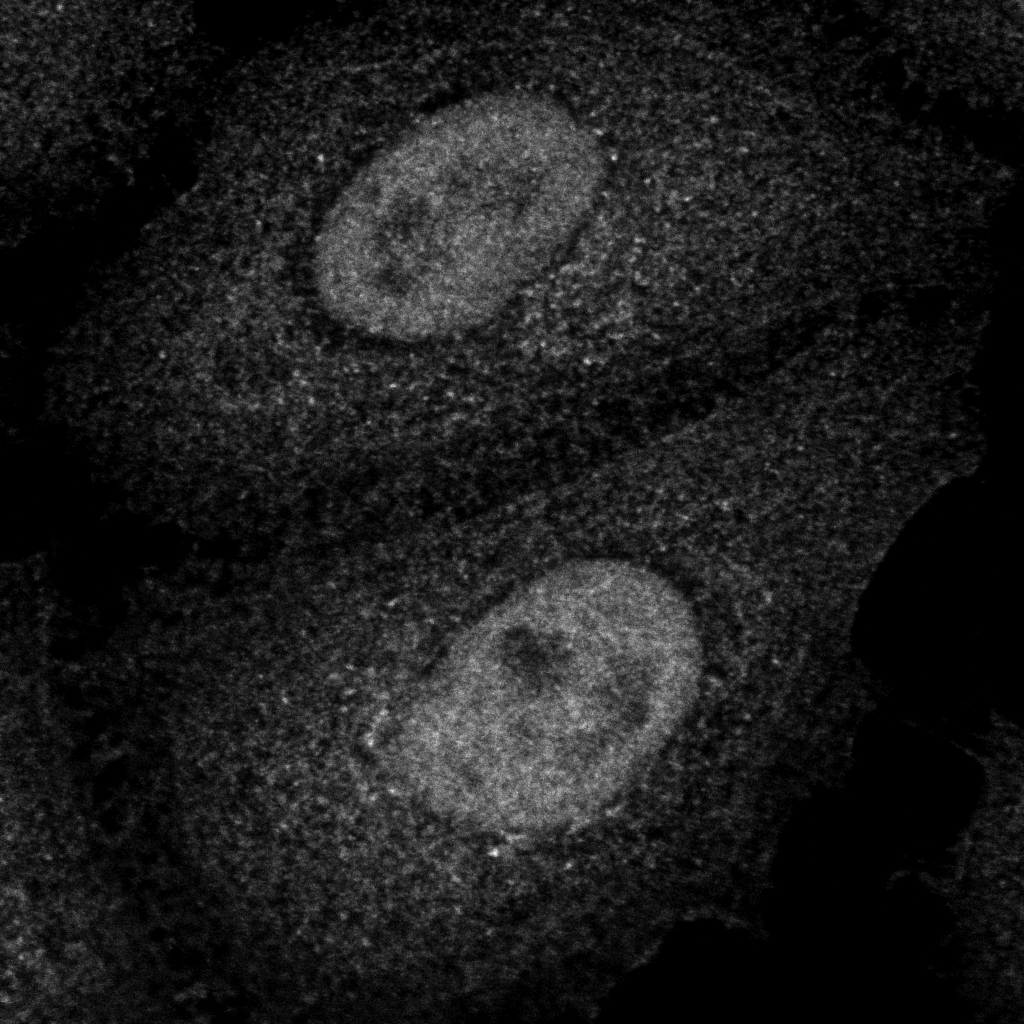

Supplement: Supplementary file 7 — Source data Fig. 5 [file 44318_2025_672_MOESM7_ESM.zip › Figure 5/5C/WT_VEH_ALG2.tif]

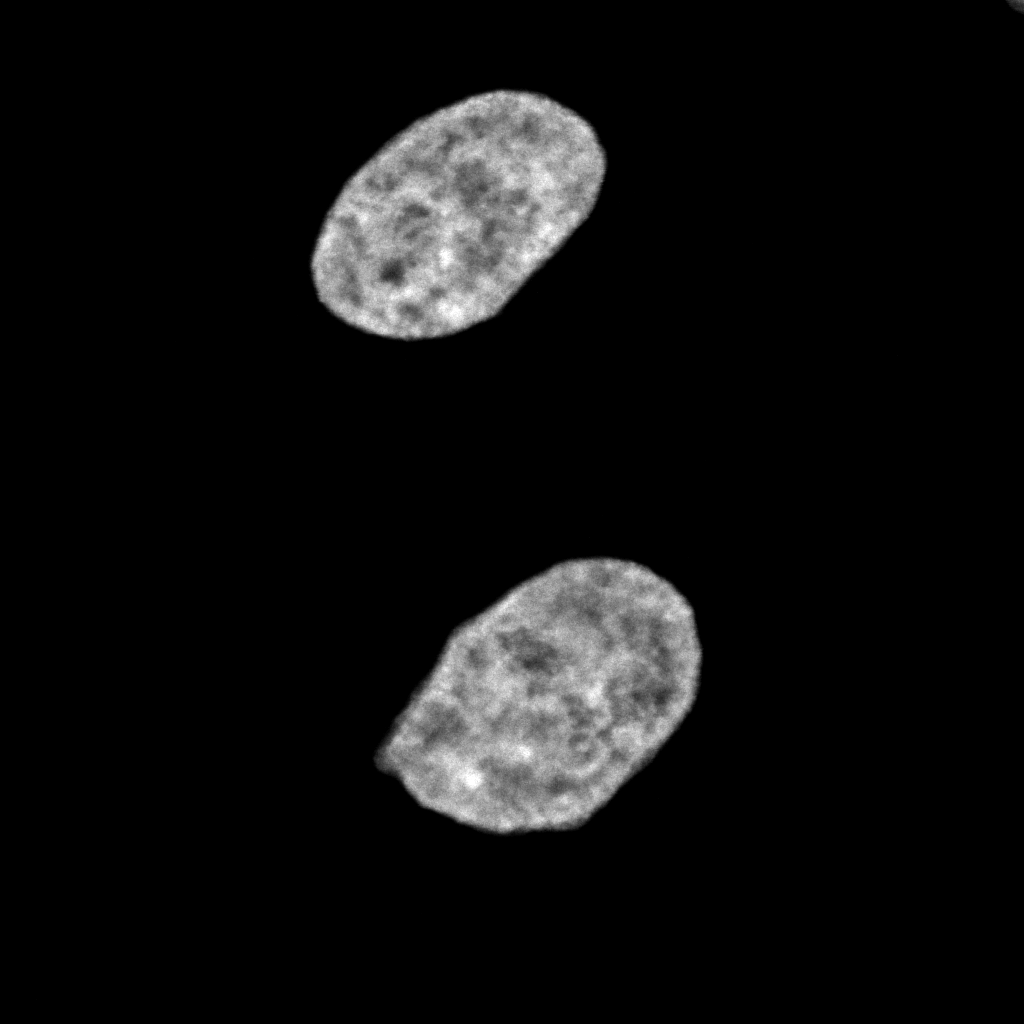

Supplement: Supplementary file 7 — Source data Fig. 5 [file 44318_2025_672_MOESM7_ESM.zip › Figure 5/5C/WT_VEH_DAPI.tif]

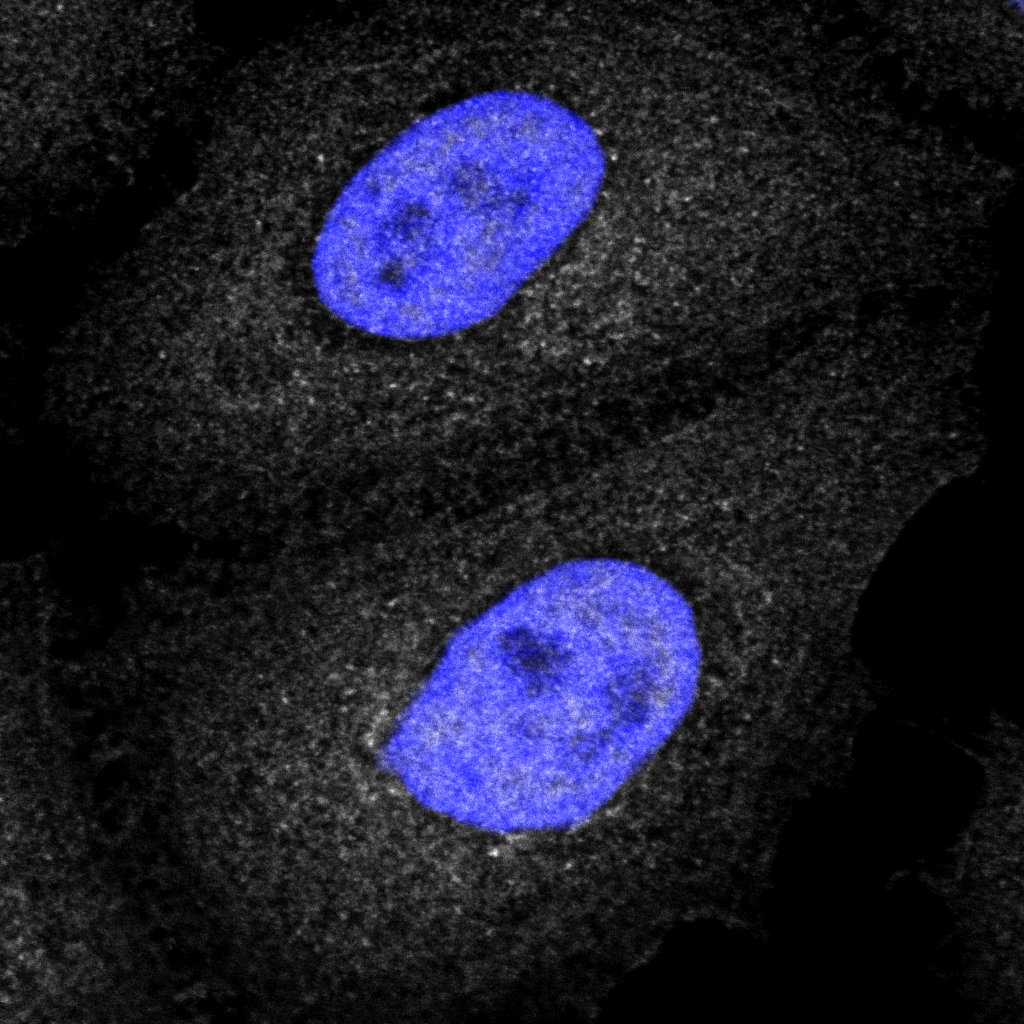

Supplement: Supplementary file 7 — Source data Fig. 5 [file 44318_2025_672_MOESM7_ESM.zip › Figure 5/5C/WT_VEH_merge.tif]

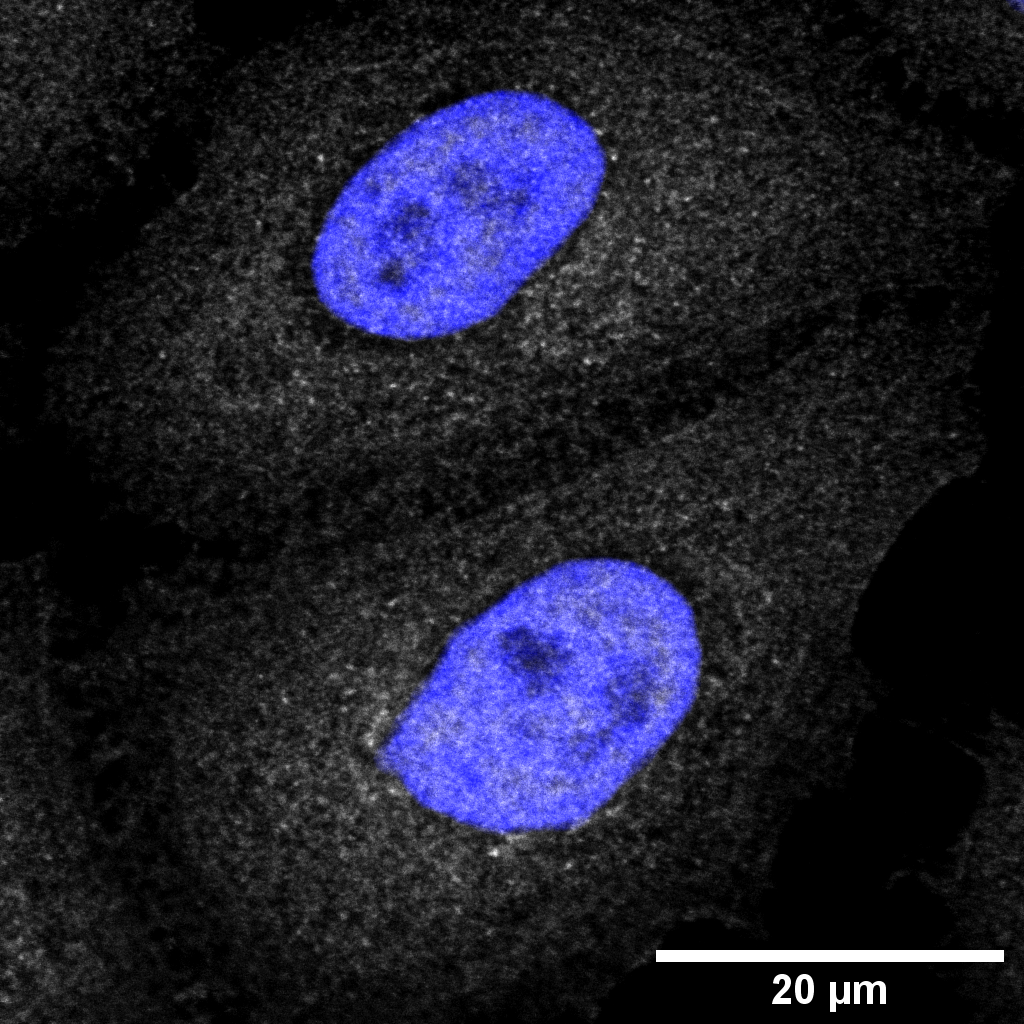

Supplement: Supplementary file 7 — Source data Fig. 5 [file 44318_2025_672_MOESM7_ESM.zip › Figure 5/5C/WT_VEH_scale.tif]

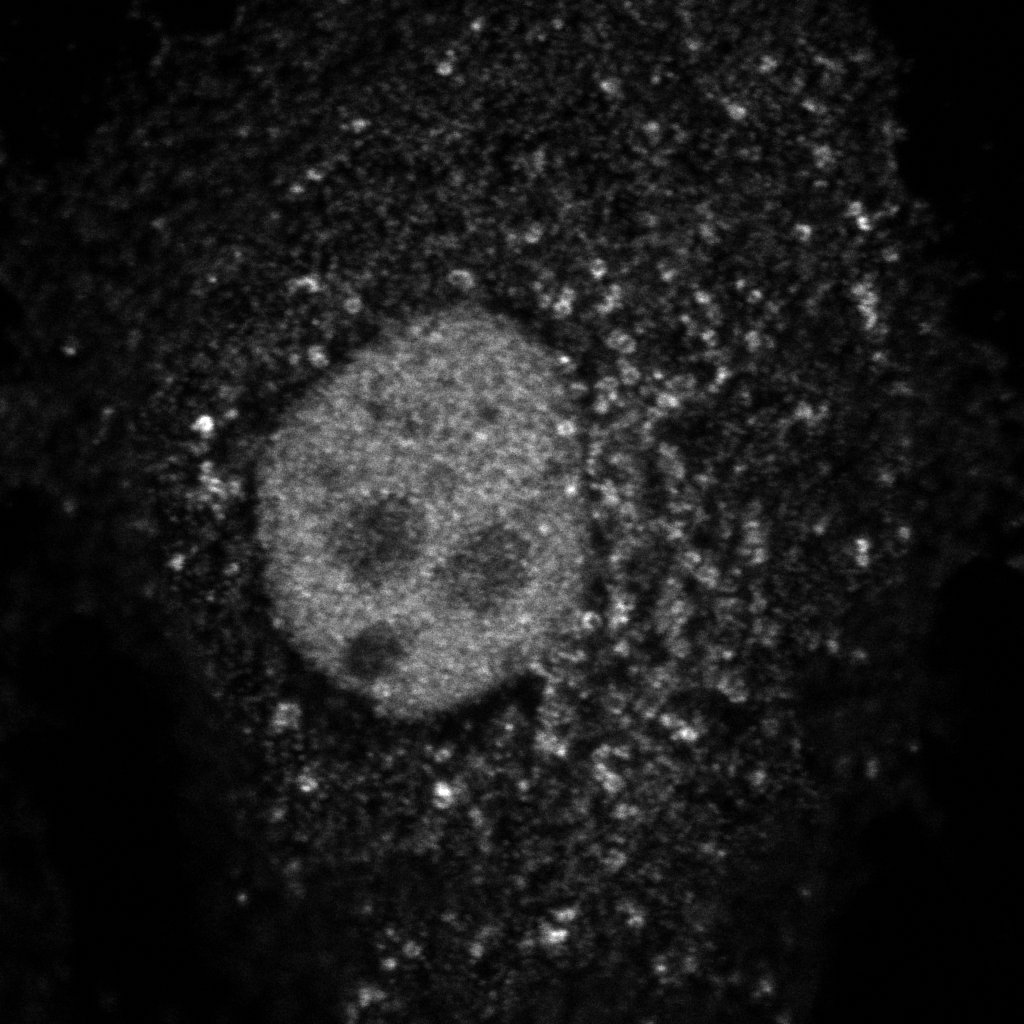

Supplement: Supplementary file 7 — Source data Fig. 5 [file 44318_2025_672_MOESM7_ESM.zip › Figure 5/5D/WT_LLOMe_ALG2.tif]

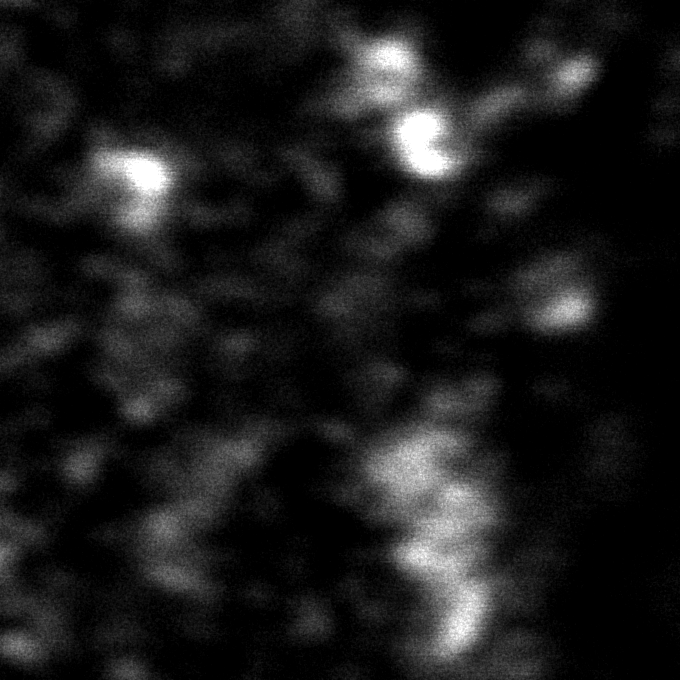

Supplement: Supplementary file 7 — Source data Fig. 5 [file 44318_2025_672_MOESM7_ESM.zip › Figure 5/5D/WT_LLOMe_ALG2_zoom.tif]

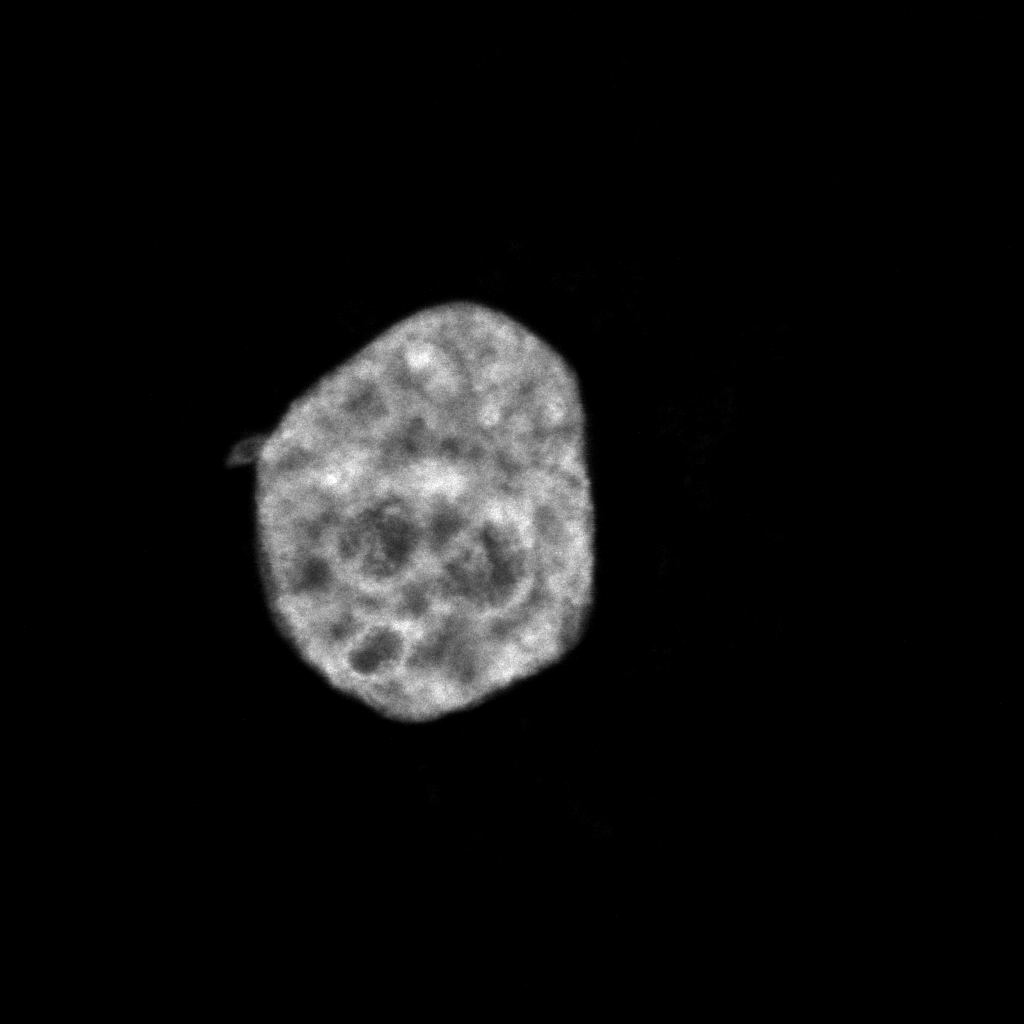

Supplement: Supplementary file 7 — Source data Fig. 5 [file 44318_2025_672_MOESM7_ESM.zip › Figure 5/5D/WT_LLOMe_DAPI.tif]

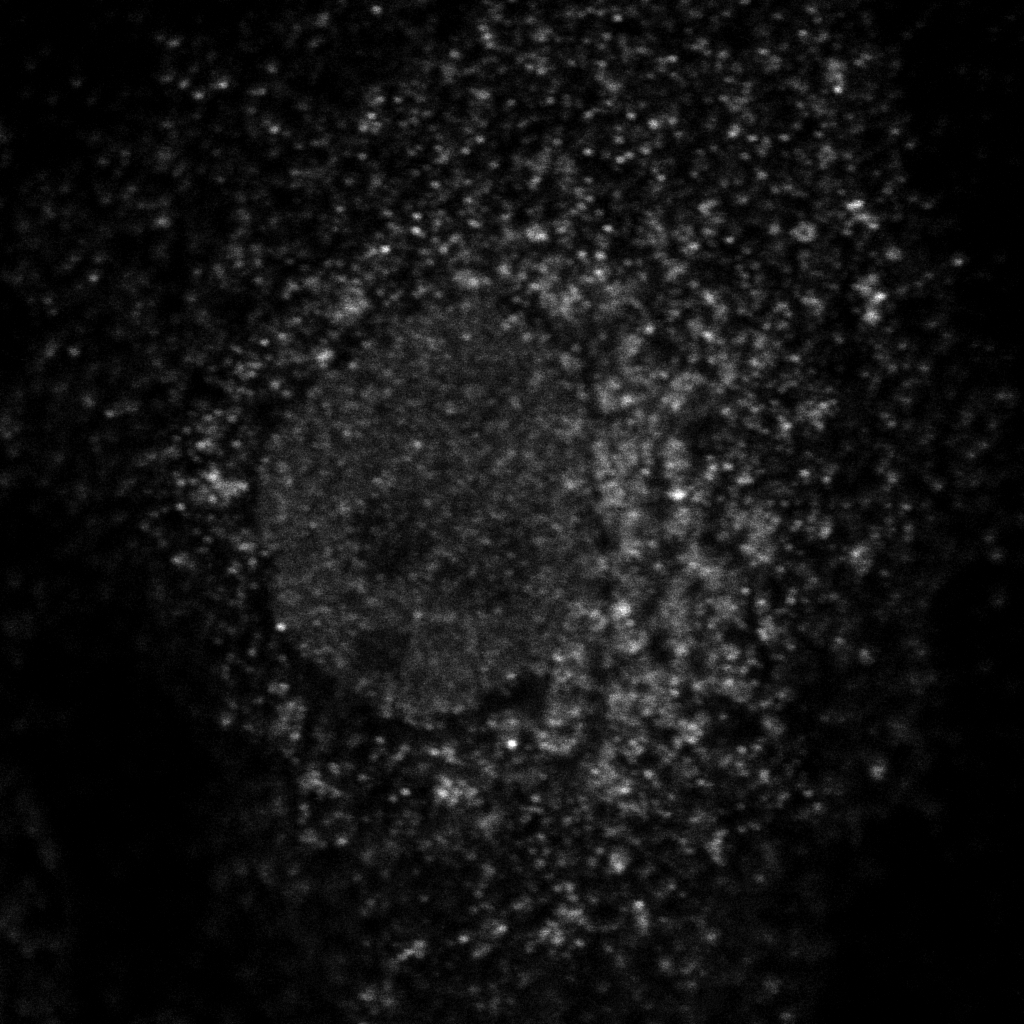

Supplement: Supplementary file 7 — Source data Fig. 5 [file 44318_2025_672_MOESM7_ESM.zip › Figure 5/5D/WT_LLOMe_HA.tif]

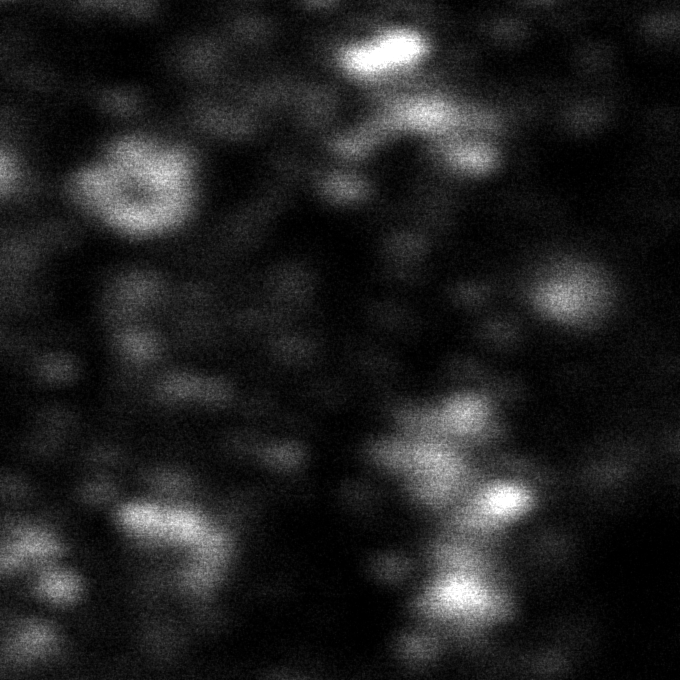

Supplement: Supplementary file 7 — Source data Fig. 5 [file 44318_2025_672_MOESM7_ESM.zip › Figure 5/5D/WT_LLOMe_HA_zoom.tif]

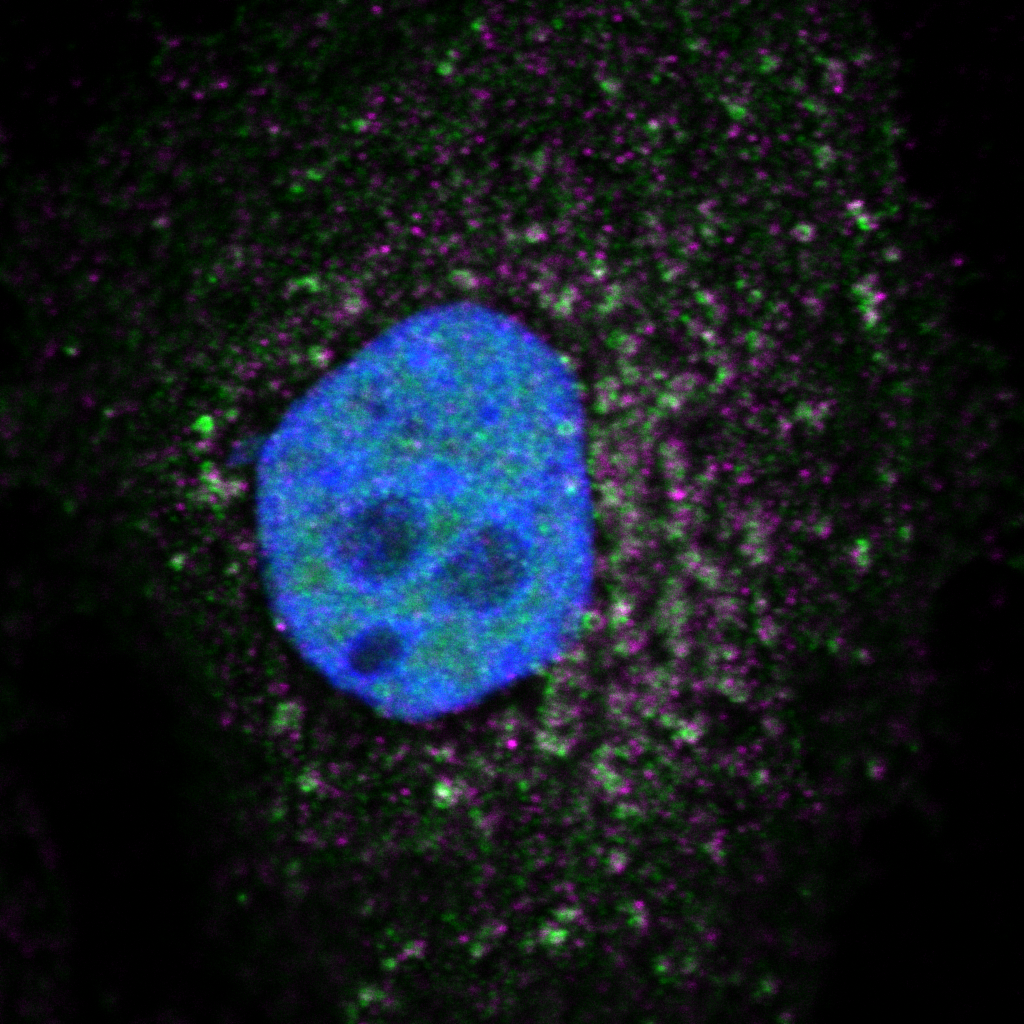

Supplement: Supplementary file 7 — Source data Fig. 5 [file 44318_2025_672_MOESM7_ESM.zip › Figure 5/5D/WT_LLOMe_merge.tif]

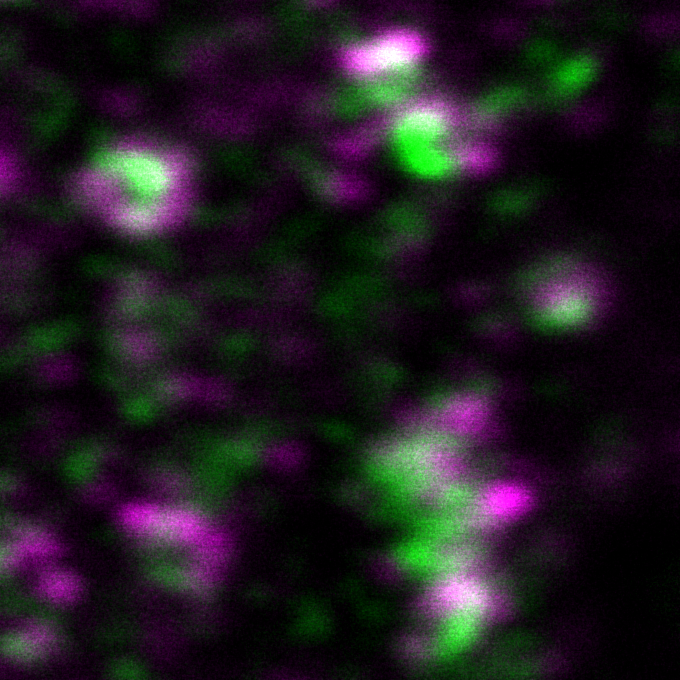

Supplement: Supplementary file 7 — Source data Fig. 5 [file 44318_2025_672_MOESM7_ESM.zip › Figure 5/5D/WT_LLOMe_merge_zoom.tif]

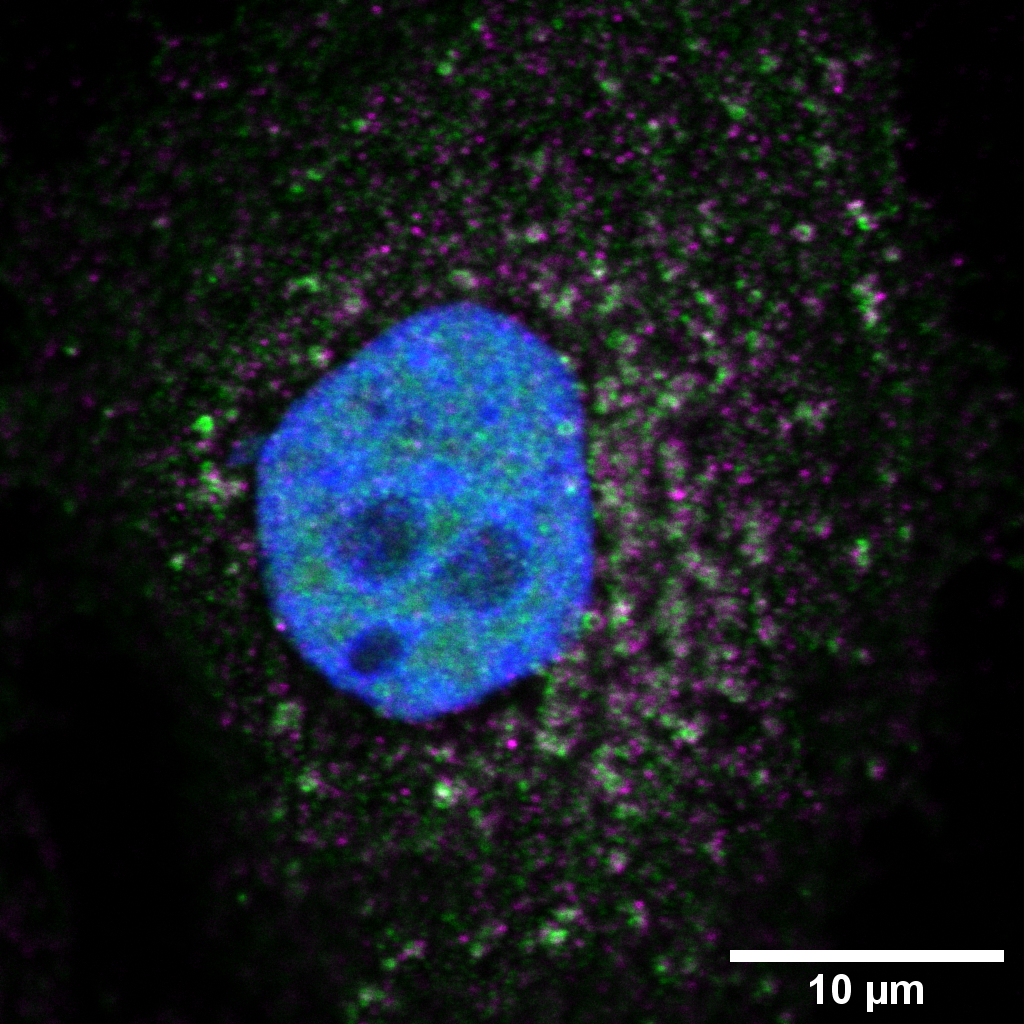

Supplement: Supplementary file 7 — Source data Fig. 5 [file 44318_2025_672_MOESM7_ESM.zip › Figure 5/5D/WT_LLOMe_scale.tif]

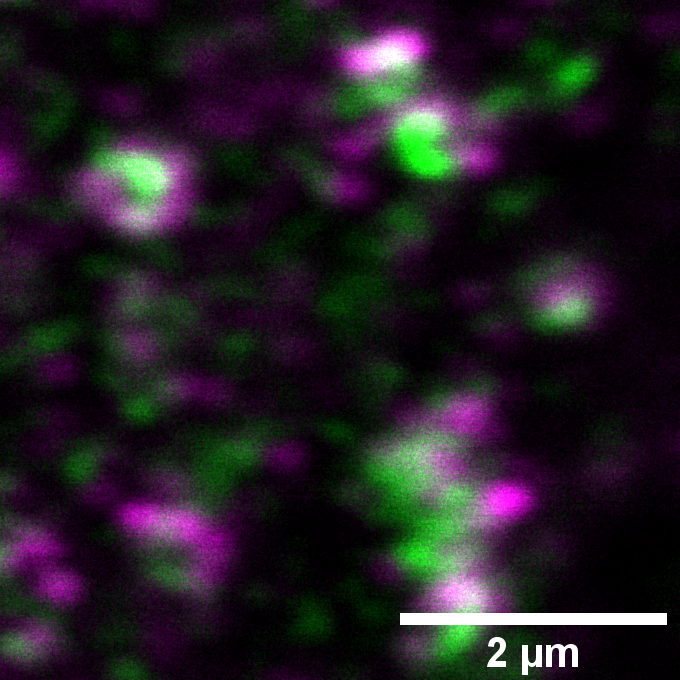

Supplement: Supplementary file 7 — Source data Fig. 5 [file 44318_2025_672_MOESM7_ESM.zip › Figure 5/5D/WT_LLOMe_scale_zoom.tif]

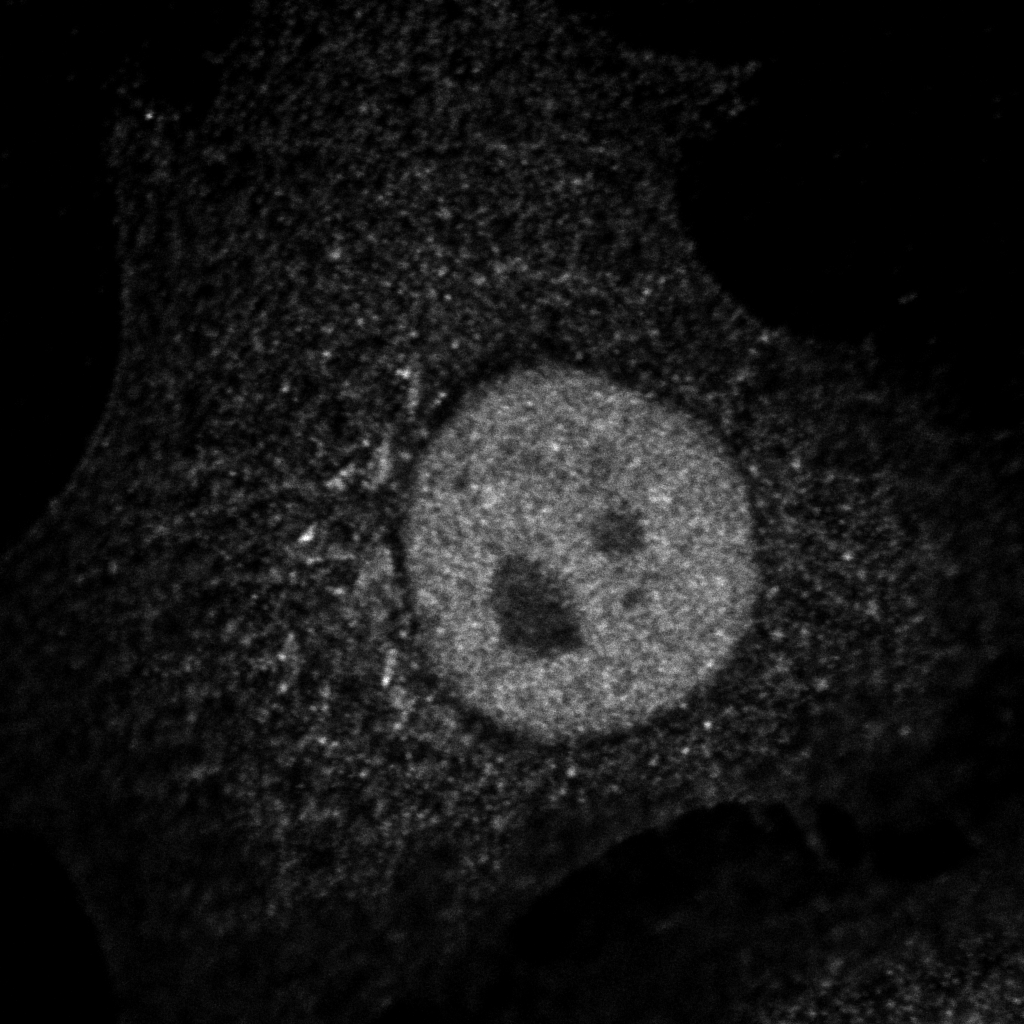

Supplement: Supplementary file 7 — Source data Fig. 5 [file 44318_2025_672_MOESM7_ESM.zip › Figure 5/5D/WT_VEH_ALG2.tif]

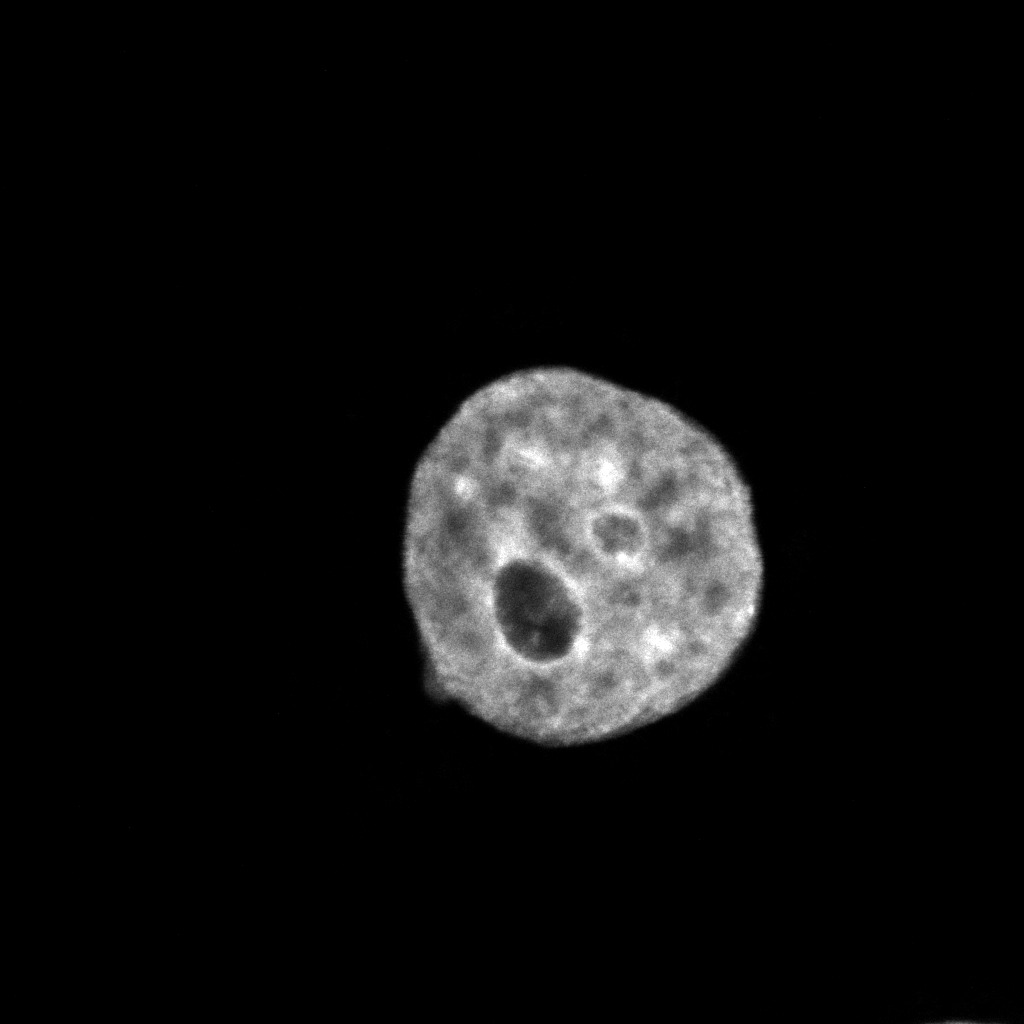

Supplement: Supplementary file 7 — Source data Fig. 5 [file 44318_2025_672_MOESM7_ESM.zip › Figure 5/5D/WT_VEH_DAPI.tif]

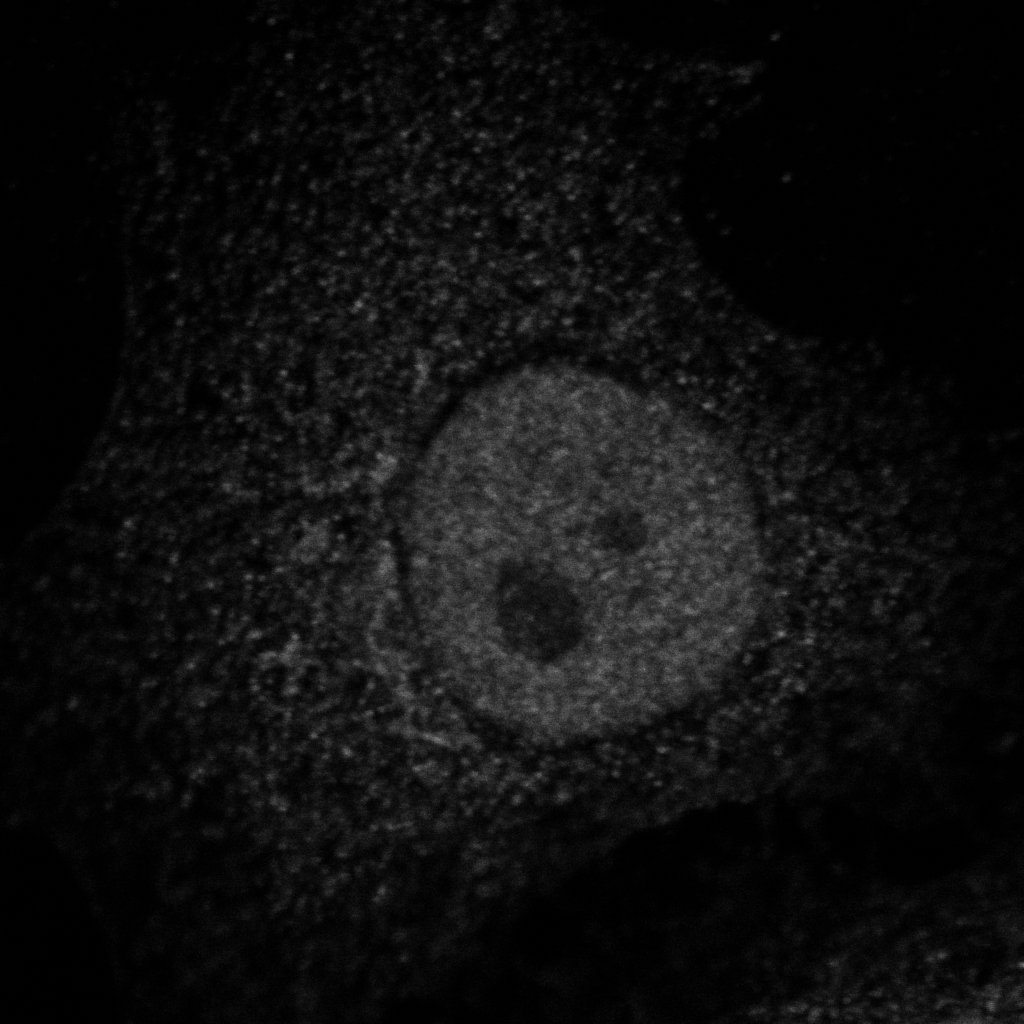

Supplement: Supplementary file 7 — Source data Fig. 5 [file 44318_2025_672_MOESM7_ESM.zip › Figure 5/5D/WT_VEH_HA.tif]

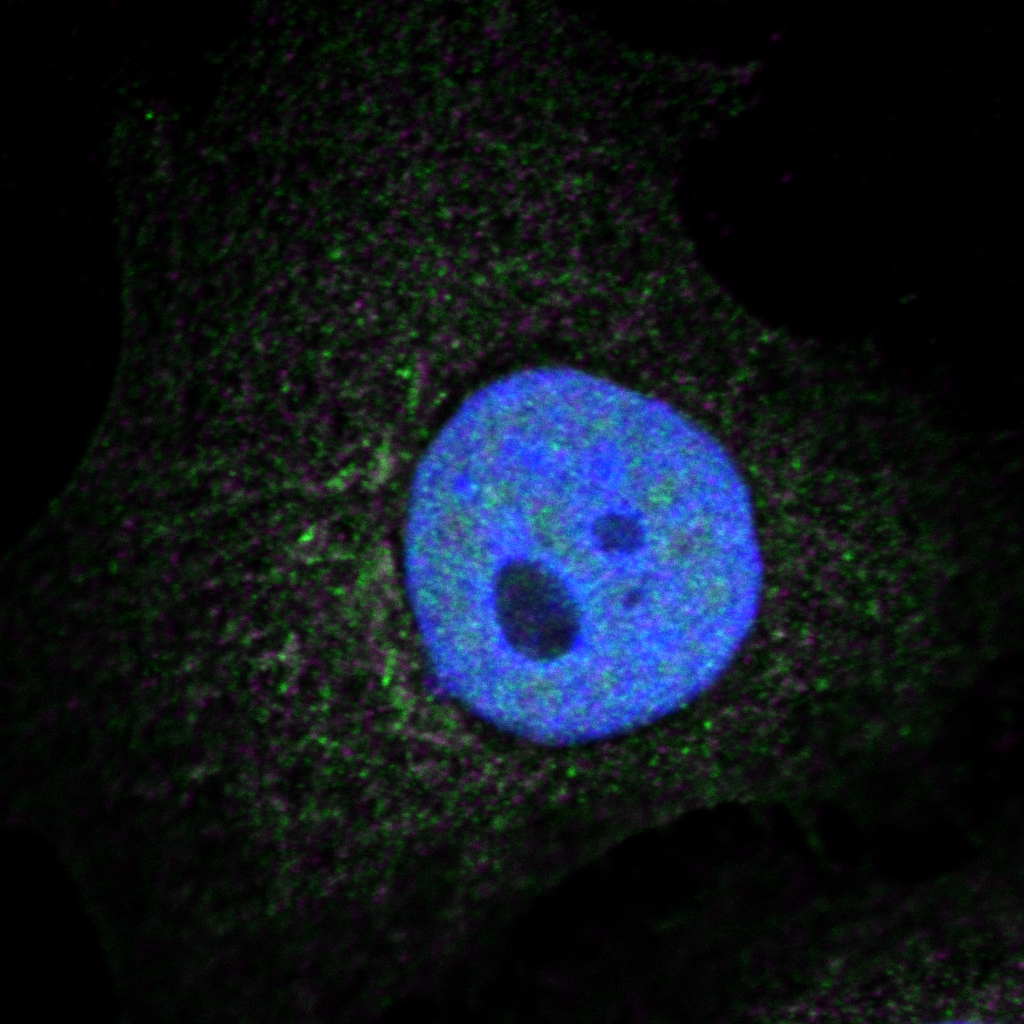

Supplement: Supplementary file 7 — Source data Fig. 5 [file 44318_2025_672_MOESM7_ESM.zip › Figure 5/5D/WT_VEH_merge.tif]

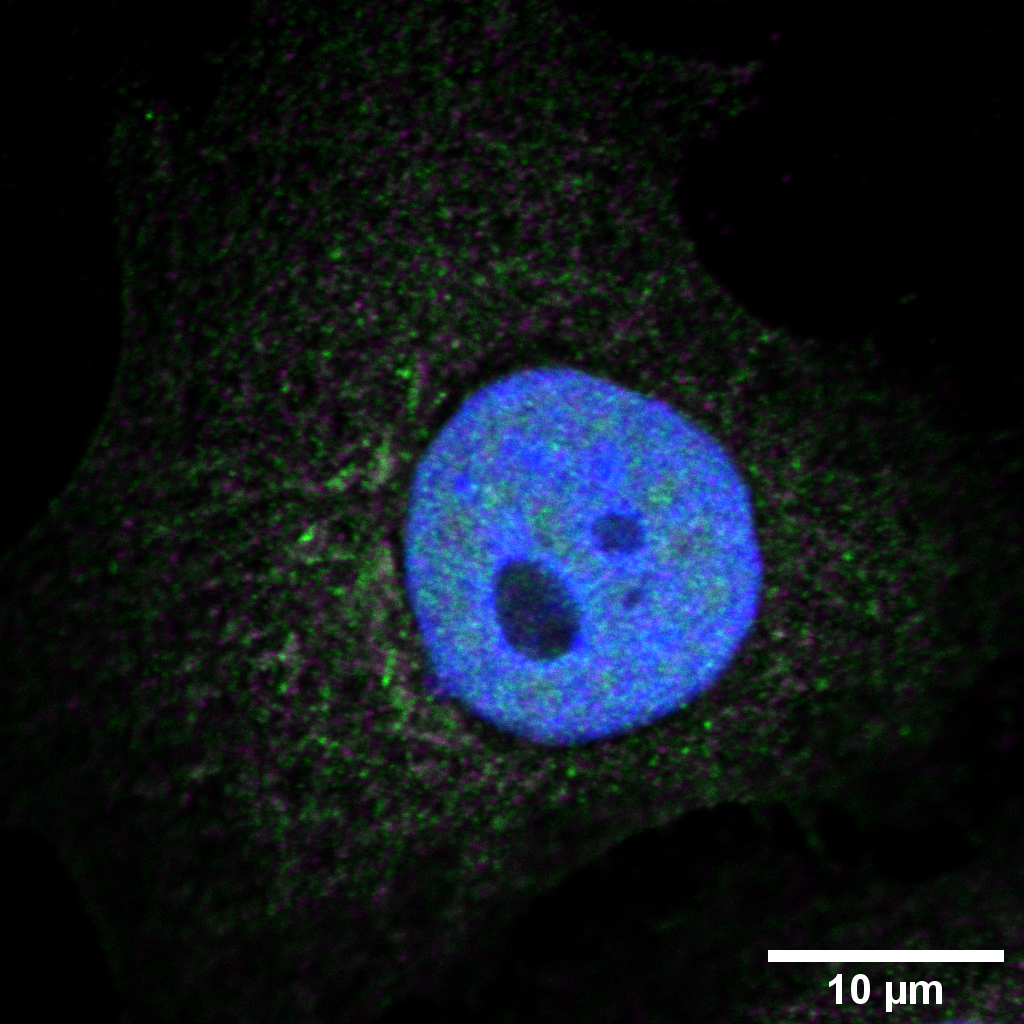

Supplement: Supplementary file 7 — Source data Fig. 5 [file 44318_2025_672_MOESM7_ESM.zip › Figure 5/5D/WT_VEH_scale.tif]

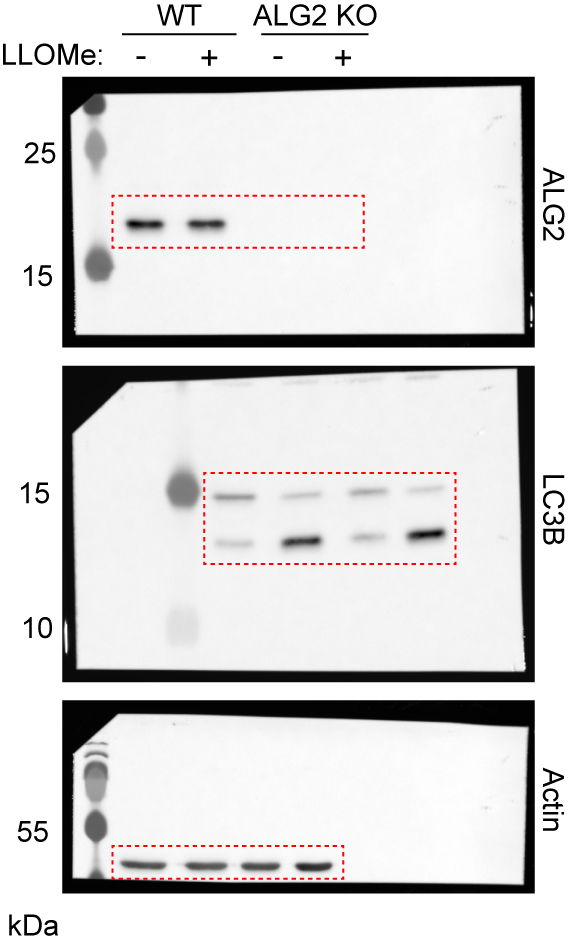

Supplement: Supplementary file 8 — Source data Fig. 6 [file 44318_2025_672_MOESM8_ESM.zip › Figure 6/6A/HeLa ALG2KO_uncropped blots.jpg]

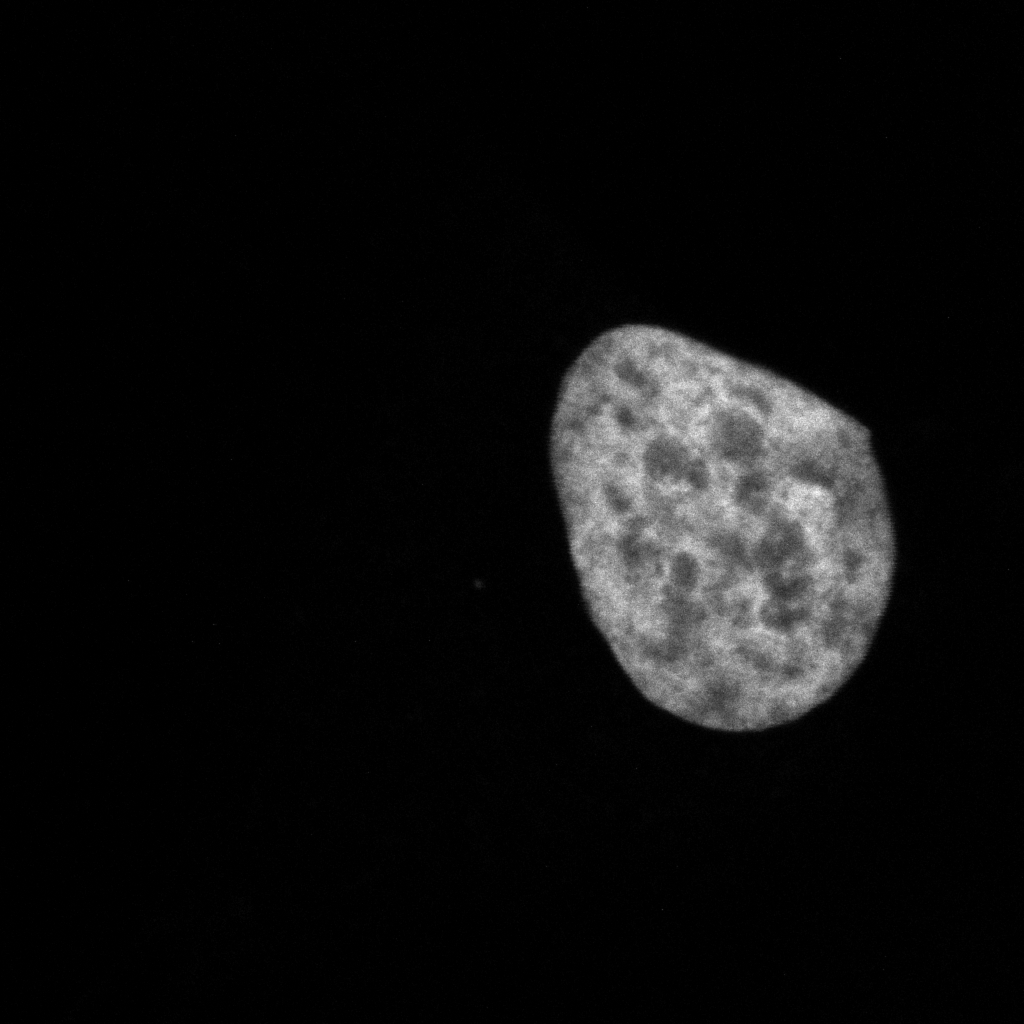

Supplement: Supplementary file 8 — Source data Fig. 6 [file 44318_2025_672_MOESM8_ESM.zip › Figure 6/6B/ALG2KO_LLOMe_DAPI.tif]

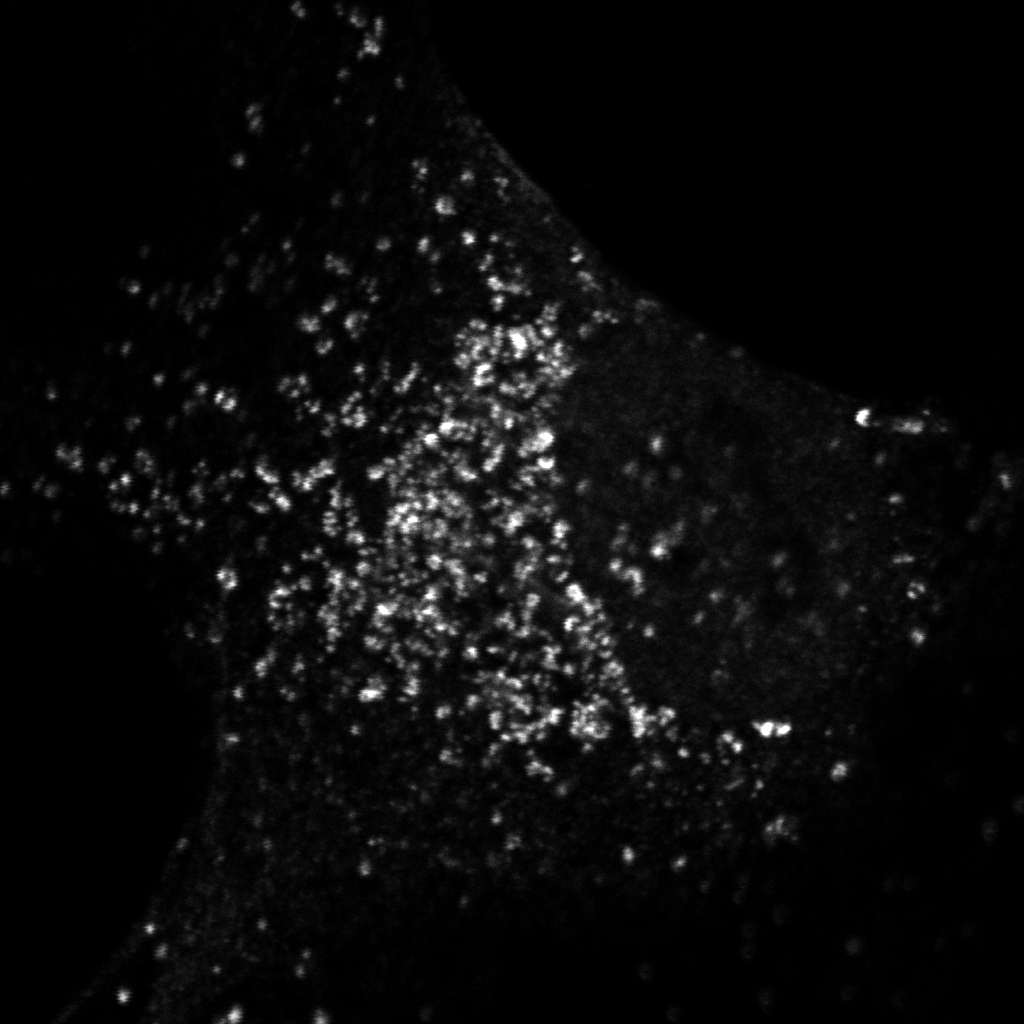

Supplement: Supplementary file 8 — Source data Fig. 6 [file 44318_2025_672_MOESM8_ESM.zip › Figure 6/6B/ALG2KO_LLOMe_IST1.tif]

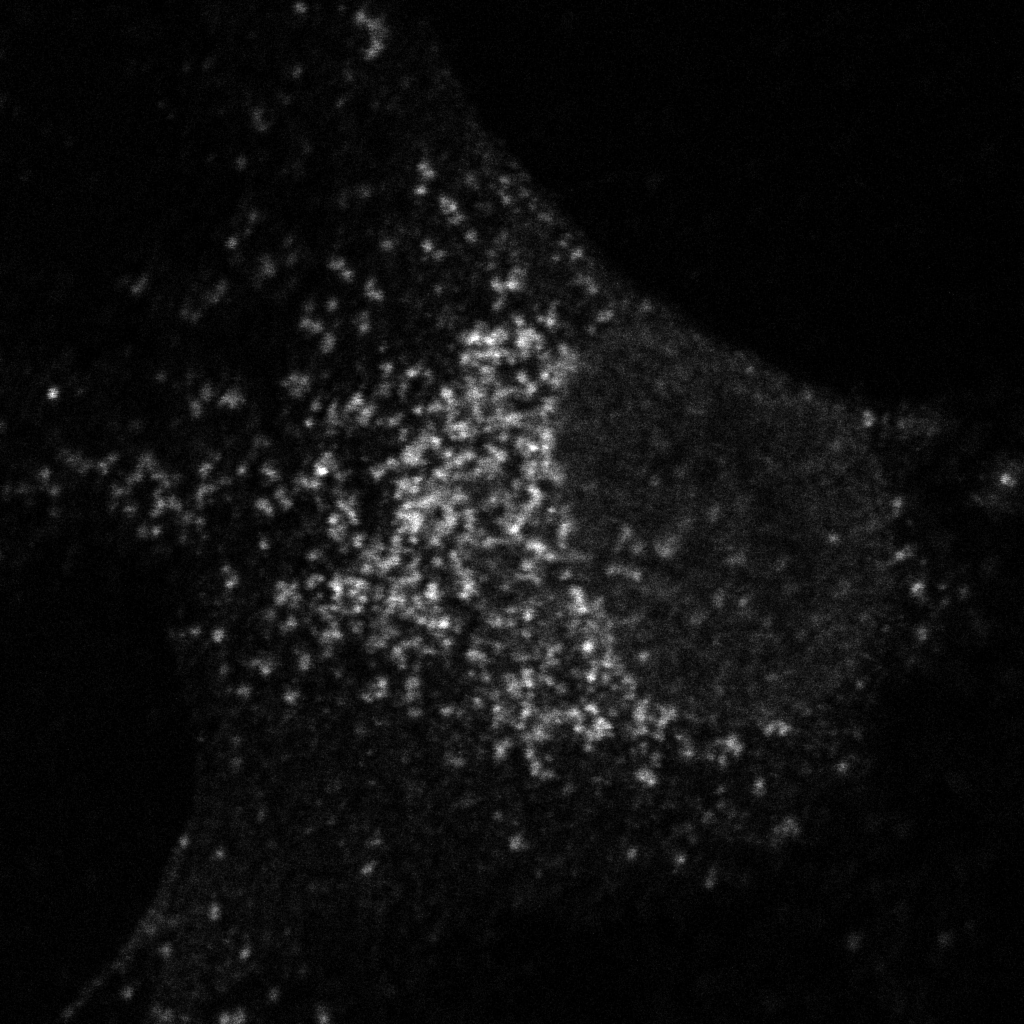

Supplement: Supplementary file 8 — Source data Fig. 6 [file 44318_2025_672_MOESM8_ESM.zip › Figure 6/6B/ALG2KO_LLOMe_LAMP.tif]

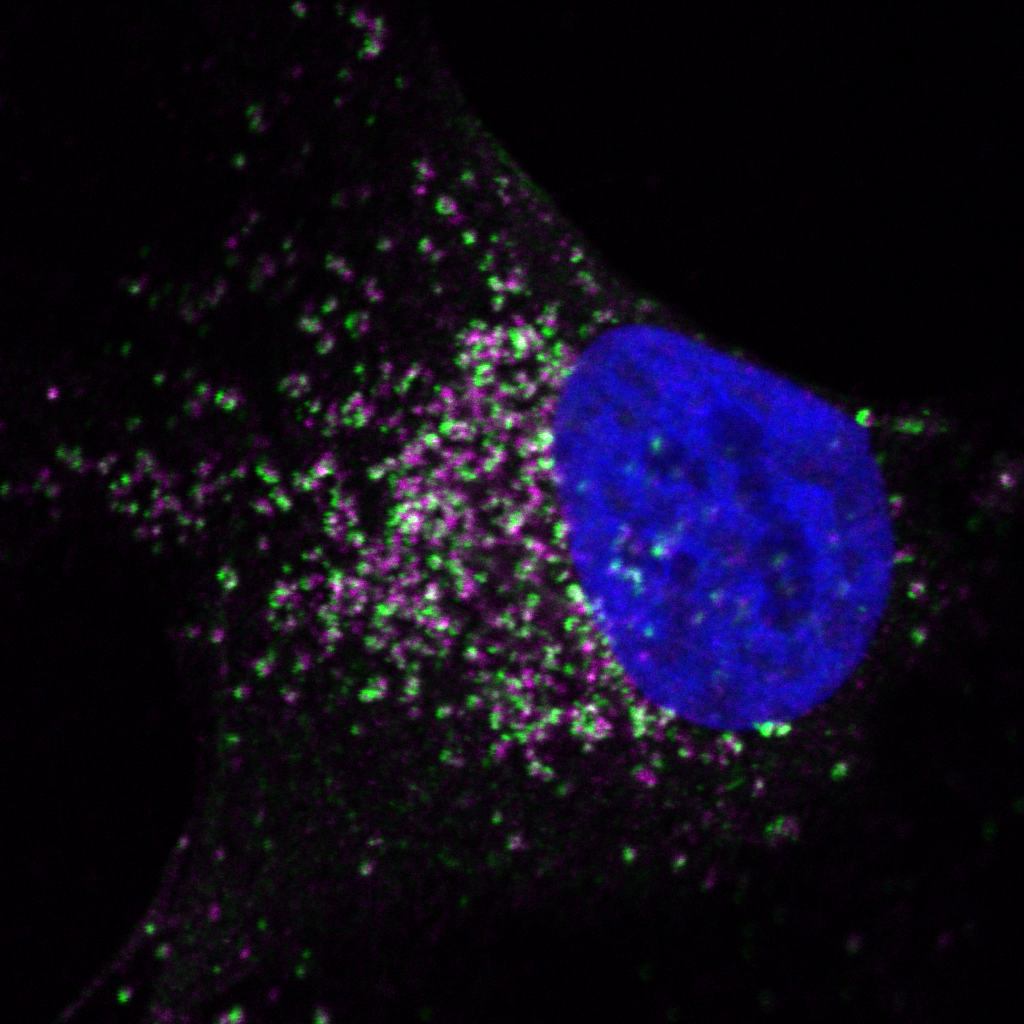

Supplement: Supplementary file 8 — Source data Fig. 6 [file 44318_2025_672_MOESM8_ESM.zip › Figure 6/6B/ALG2KO_LLOMe_Merge.tif]

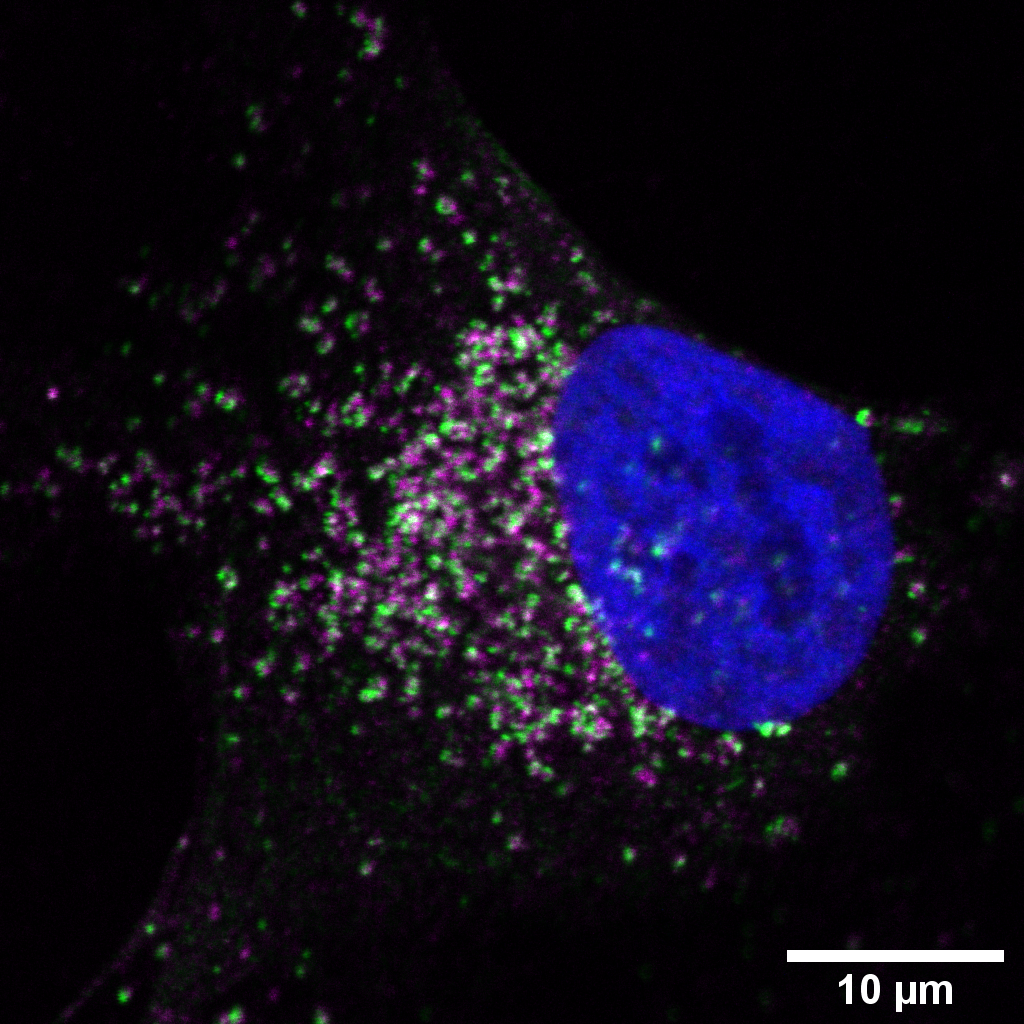

Supplement: Supplementary file 8 — Source data Fig. 6 [file 44318_2025_672_MOESM8_ESM.zip › Figure 6/6B/ALG2KO_LLOMe_scale.tif]

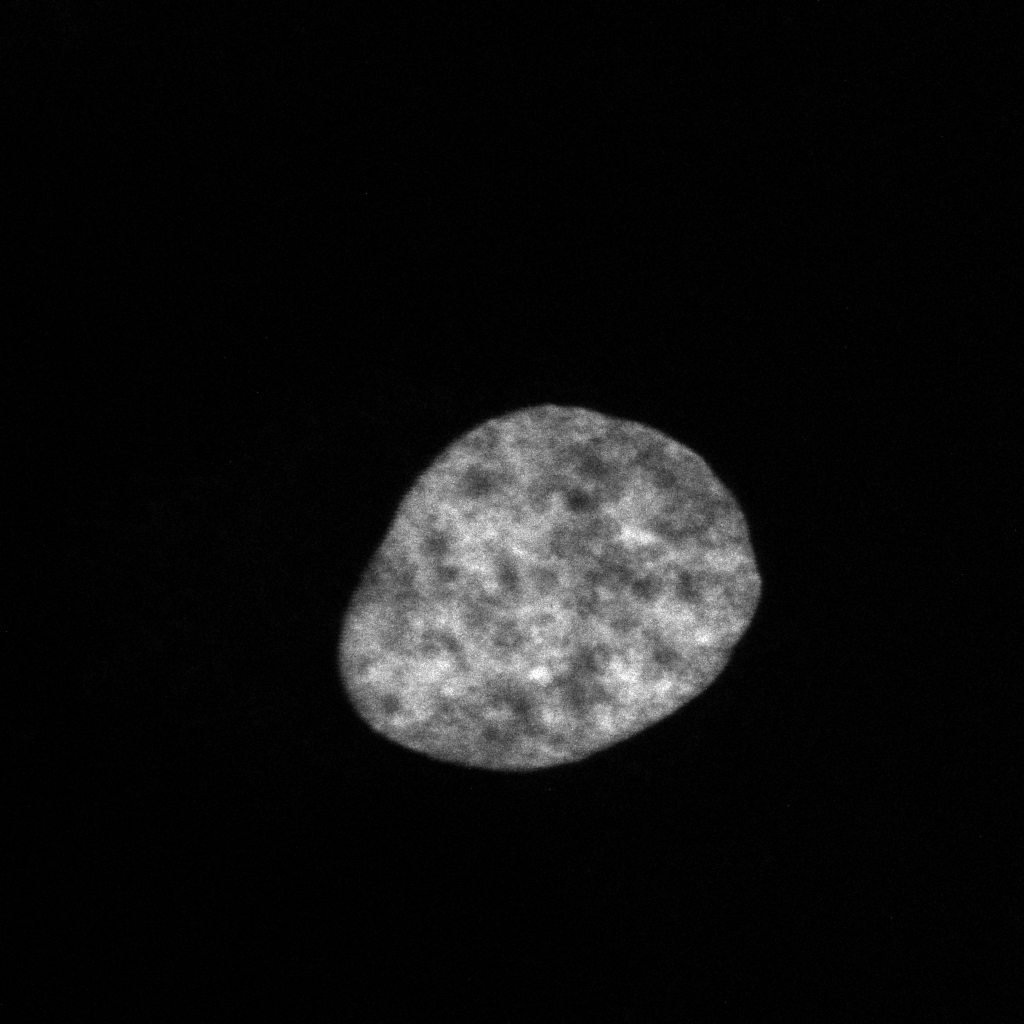

Supplement: Supplementary file 8 — Source data Fig. 6 [file 44318_2025_672_MOESM8_ESM.zip › Figure 6/6B/ALG2KO_VEH_DAPI.tif]

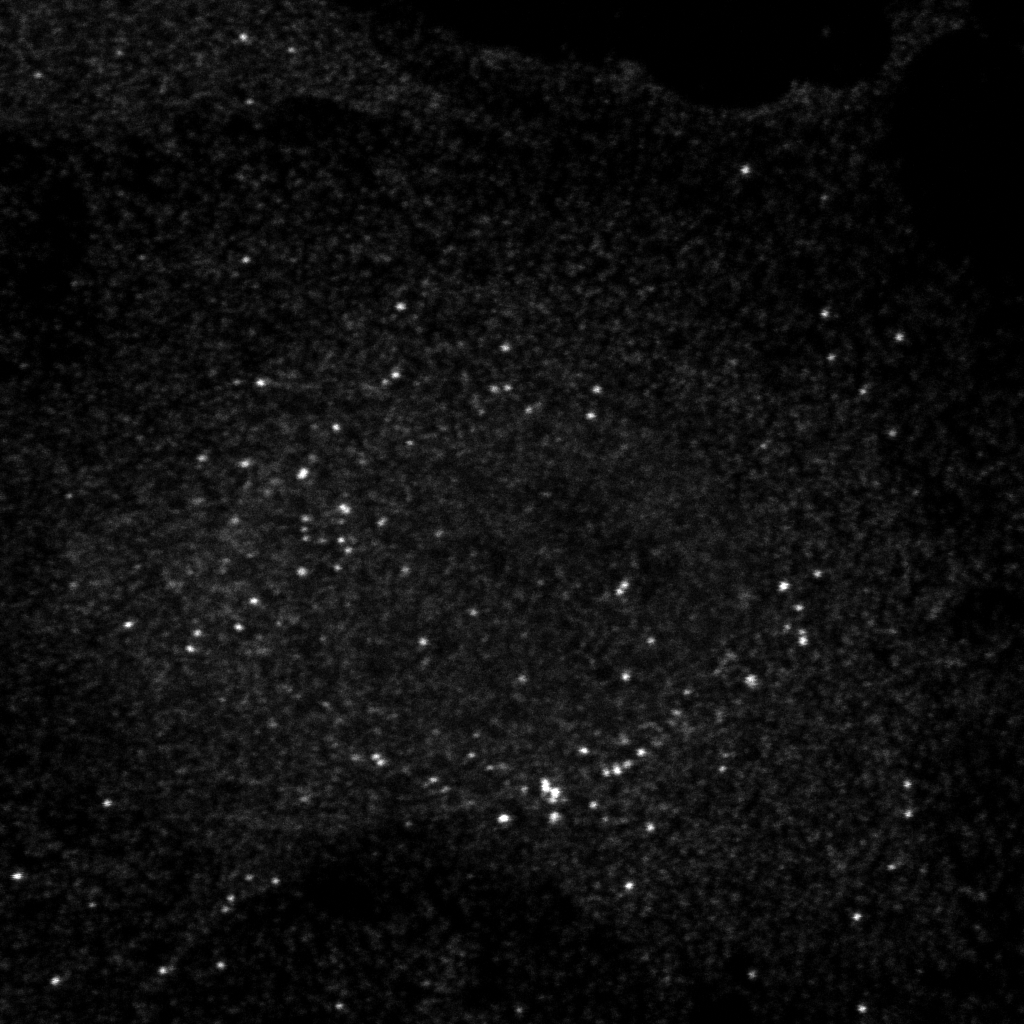

Supplement: Supplementary file 8 — Source data Fig. 6 [file 44318_2025_672_MOESM8_ESM.zip › Figure 6/6B/ALG2KO_VEH_IST1.tif]

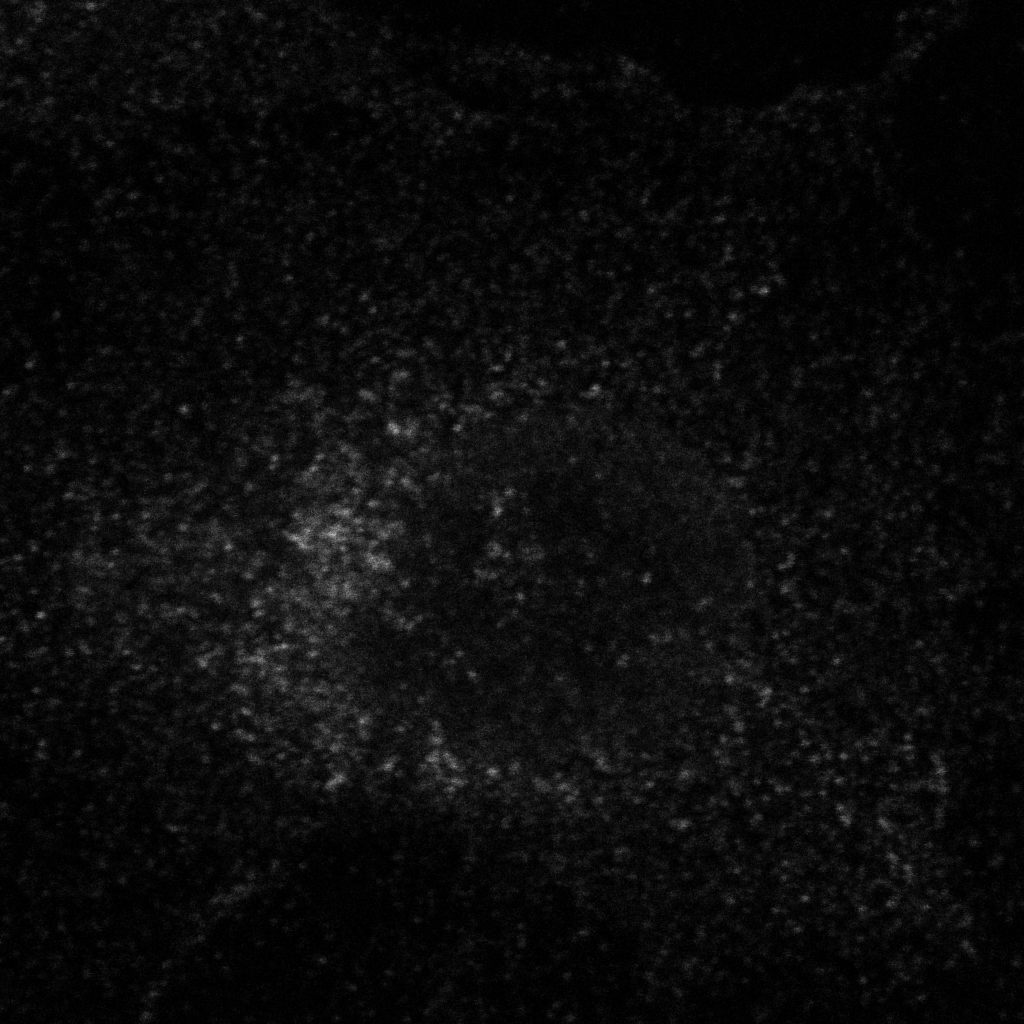

Supplement: Supplementary file 8 — Source data Fig. 6 [file 44318_2025_672_MOESM8_ESM.zip › Figure 6/6B/ALG2KO_VEH_LAMP.tif]

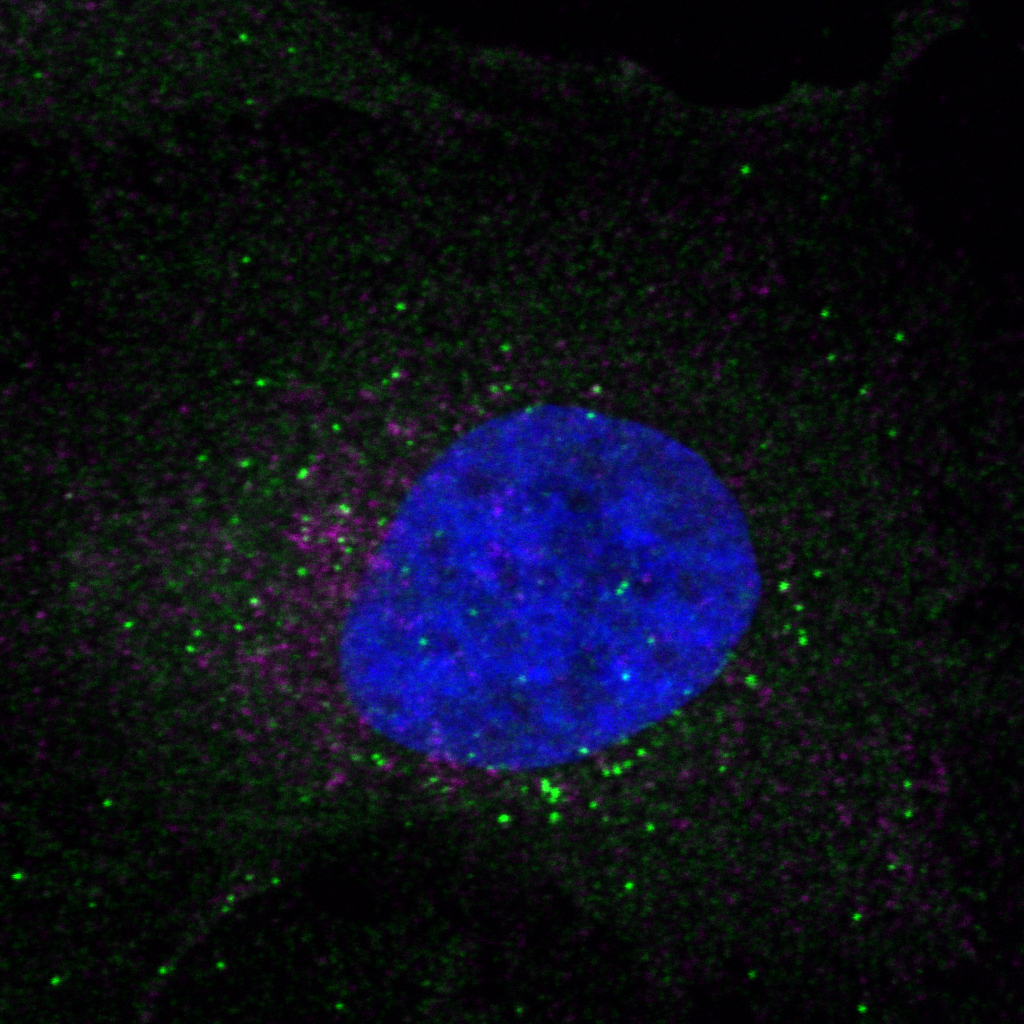

Supplement: Supplementary file 8 — Source data Fig. 6 [file 44318_2025_672_MOESM8_ESM.zip › Figure 6/6B/ALG2KO_VEH_Merge.tif]

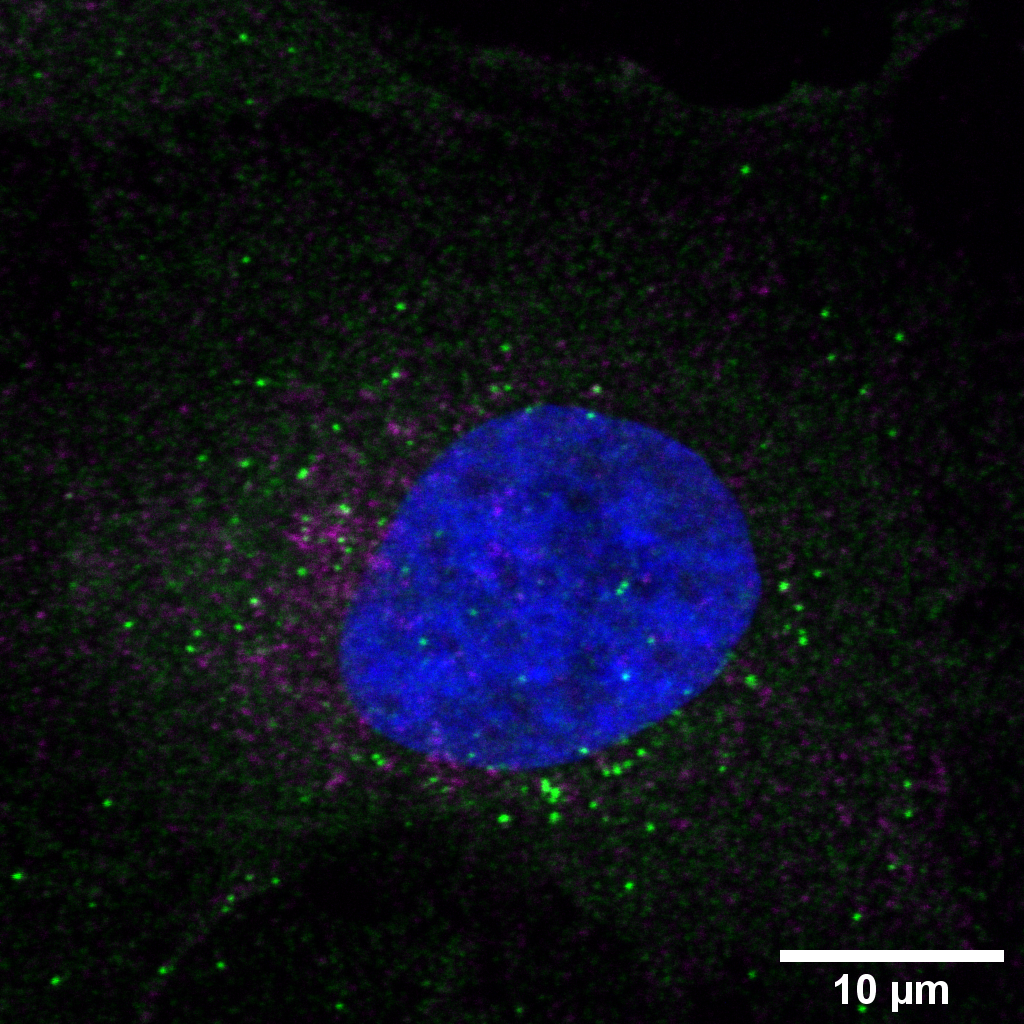

Supplement: Supplementary file 8 — Source data Fig. 6 [file 44318_2025_672_MOESM8_ESM.zip › Figure 6/6B/ALG2KO_VEH_scale.tif]

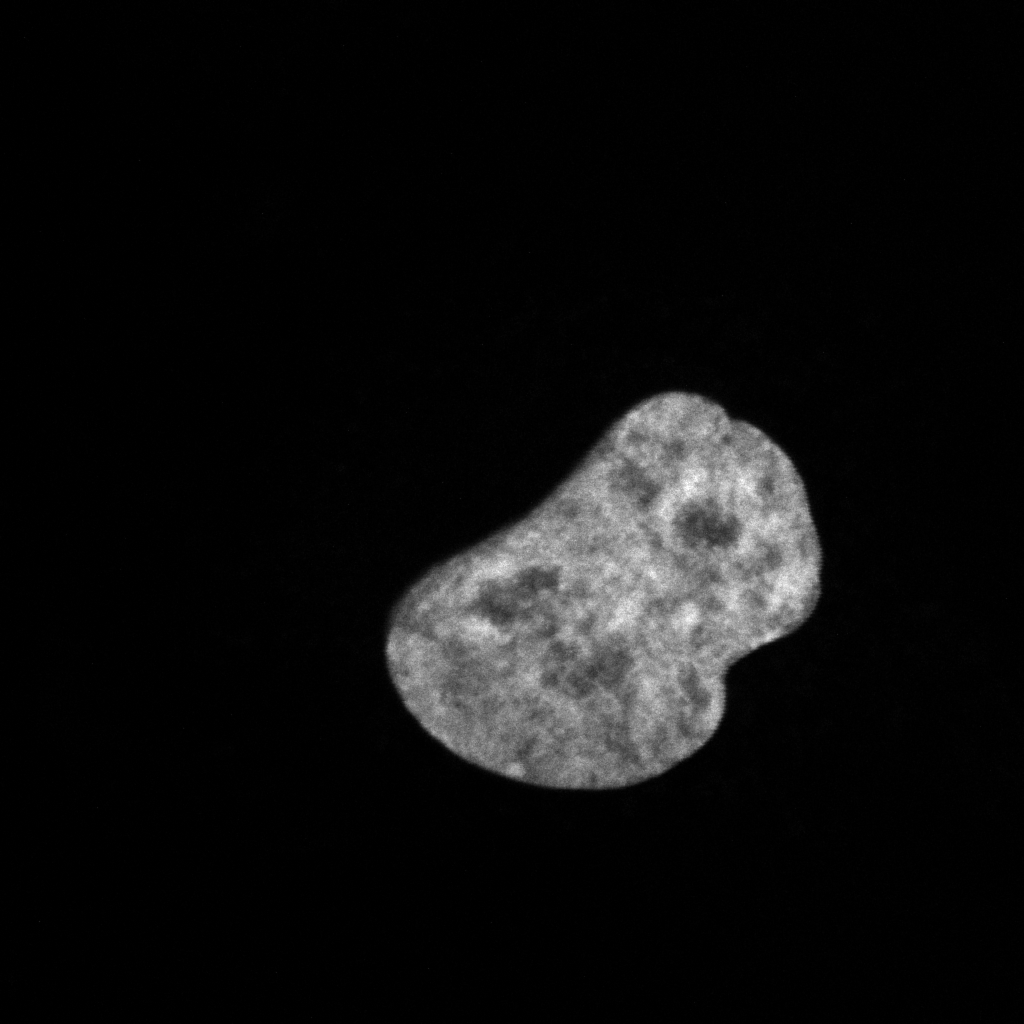

Supplement: Supplementary file 8 — Source data Fig. 6 [file 44318_2025_672_MOESM8_ESM.zip › Figure 6/6B/WT_LLOMe_DAPI.tif]

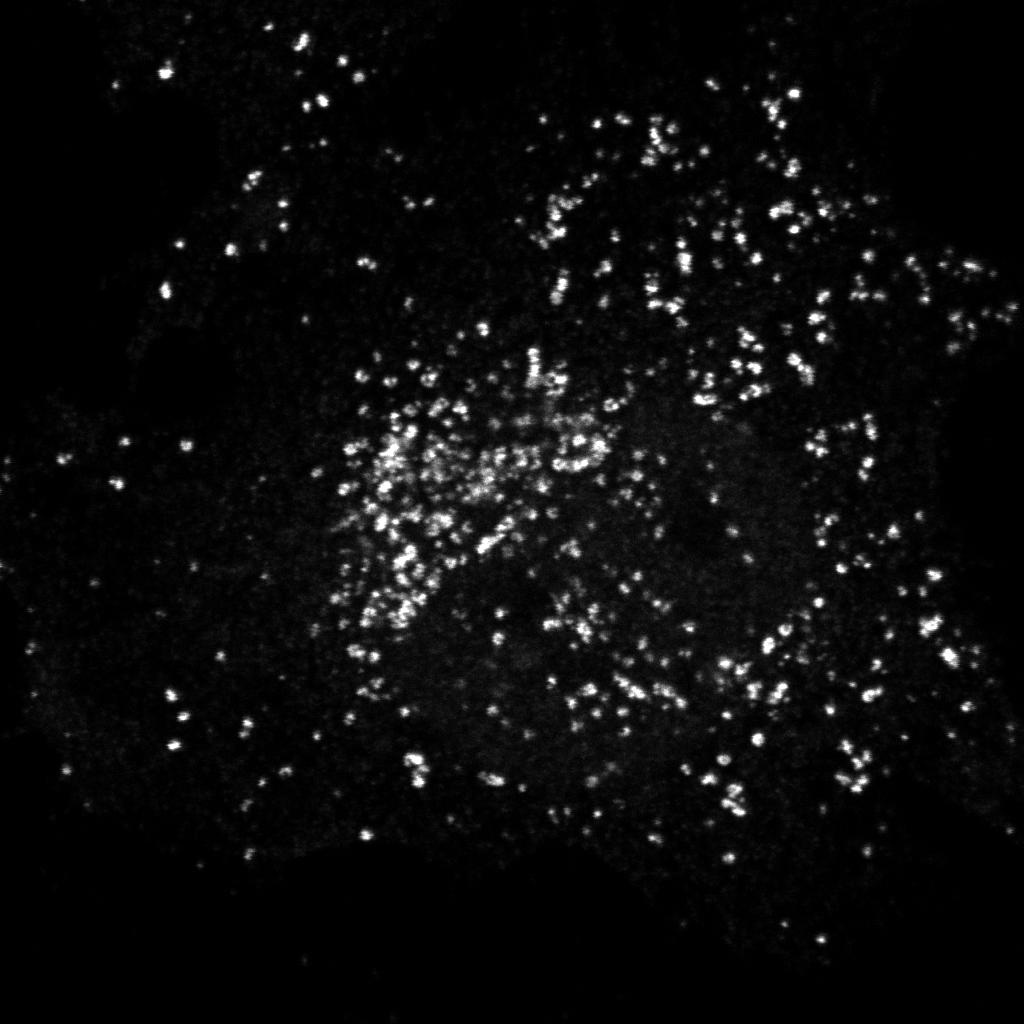

Supplement: Supplementary file 8 — Source data Fig. 6 [file 44318_2025_672_MOESM8_ESM.zip › Figure 6/6B/WT_LLOMe_IST1.tif]

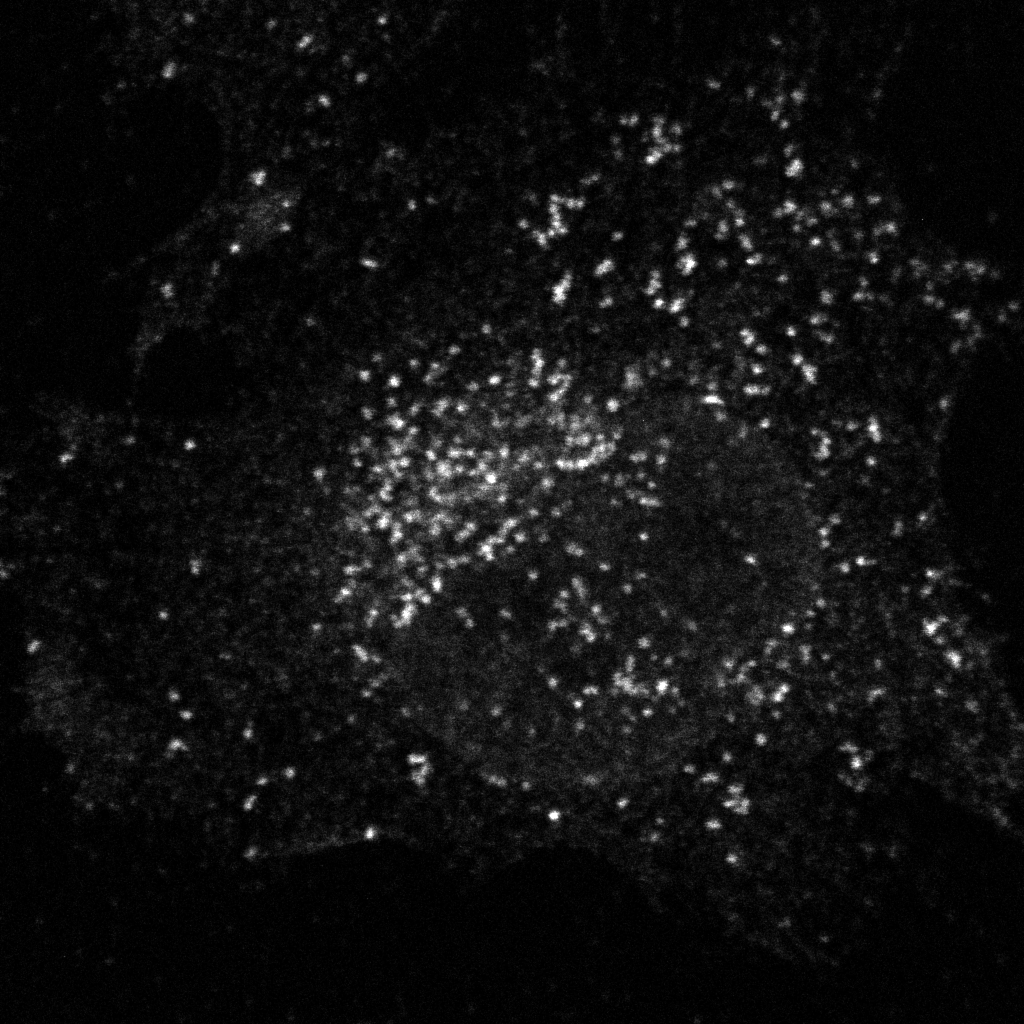

Supplement: Supplementary file 8 — Source data Fig. 6 [file 44318_2025_672_MOESM8_ESM.zip › Figure 6/6B/WT_LLOMe_LAMP.tif]

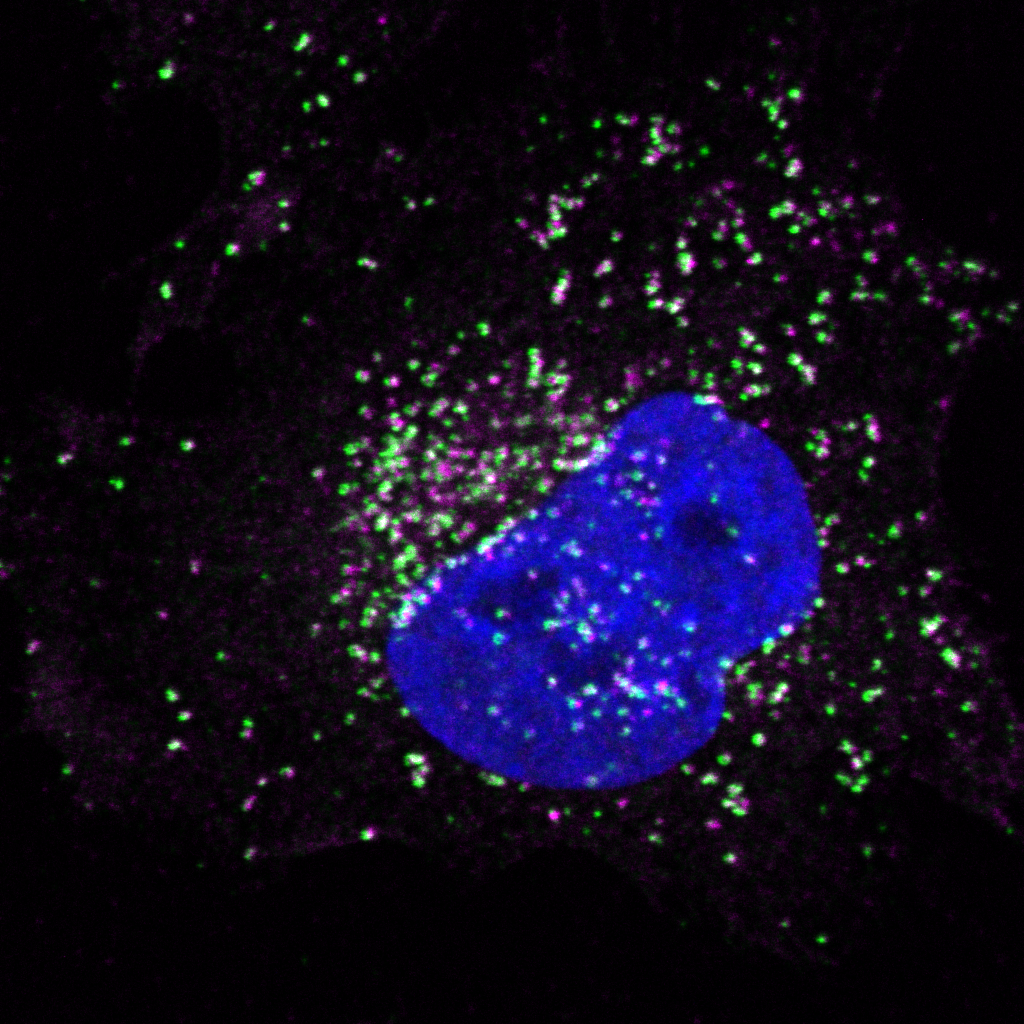

Supplement: Supplementary file 8 — Source data Fig. 6 [file 44318_2025_672_MOESM8_ESM.zip › Figure 6/6B/WT_LLOMe_Merge.tif]

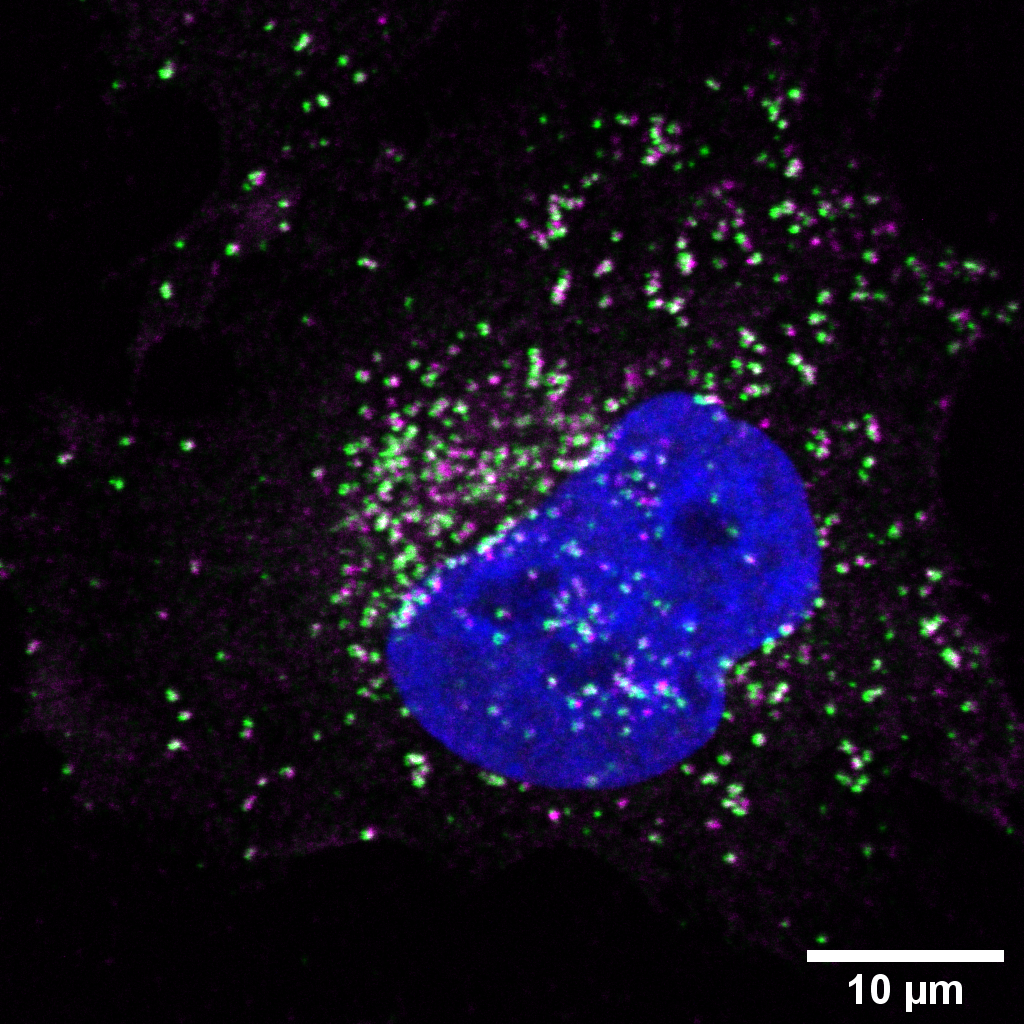

Supplement: Supplementary file 8 — Source data Fig. 6 [file 44318_2025_672_MOESM8_ESM.zip › Figure 6/6B/WT_LLOMe_scale.tif]

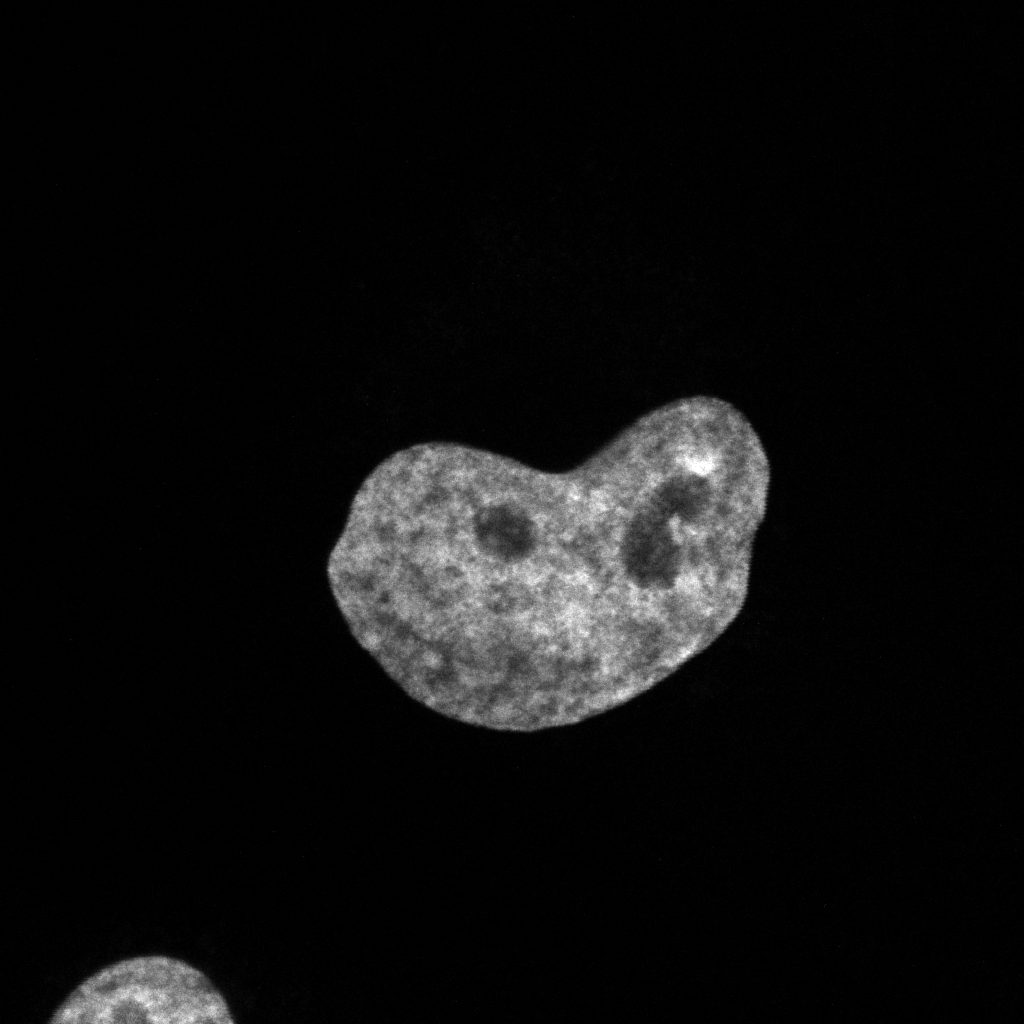

Supplement: Supplementary file 8 — Source data Fig. 6 [file 44318_2025_672_MOESM8_ESM.zip › Figure 6/6B/WT_VEH_DAPI.tif]

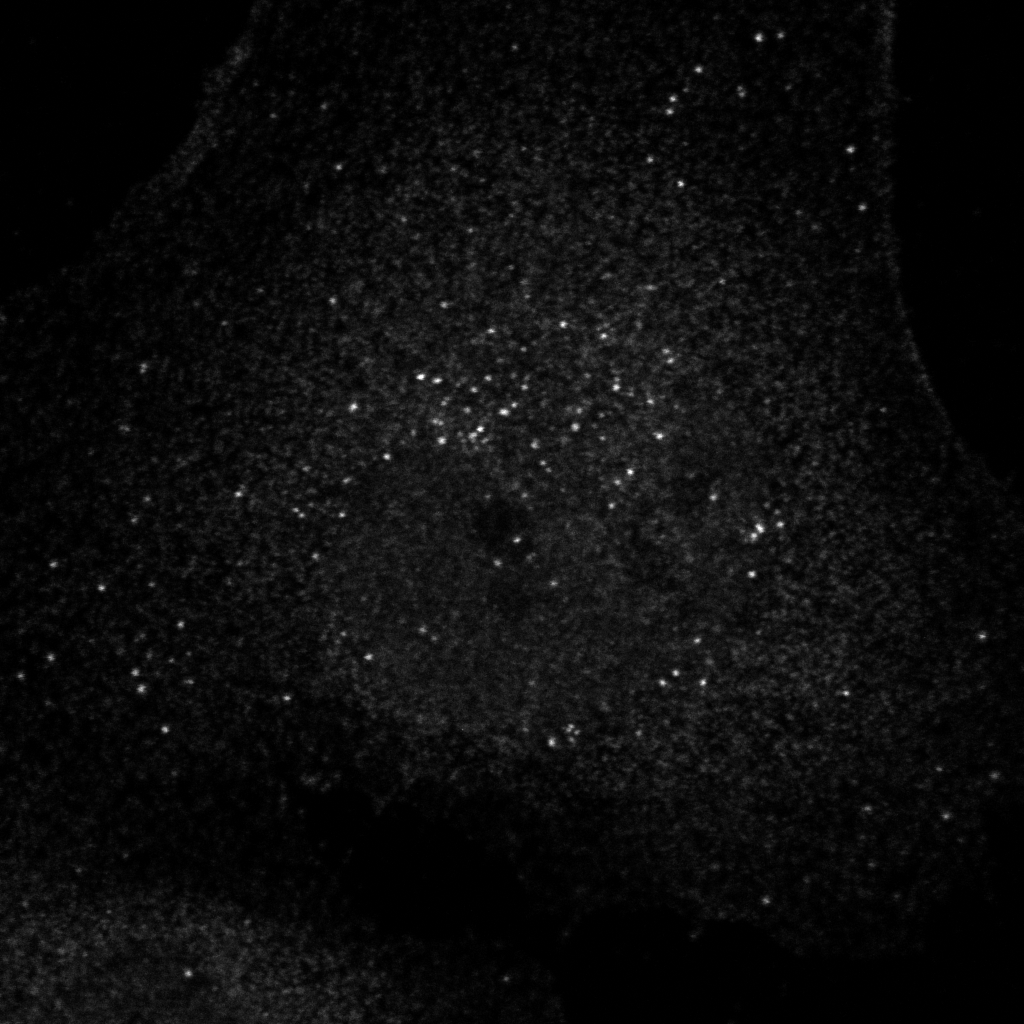

Supplement: Supplementary file 8 — Source data Fig. 6 [file 44318_2025_672_MOESM8_ESM.zip › Figure 6/6B/WT_VEH_IST1.tif]

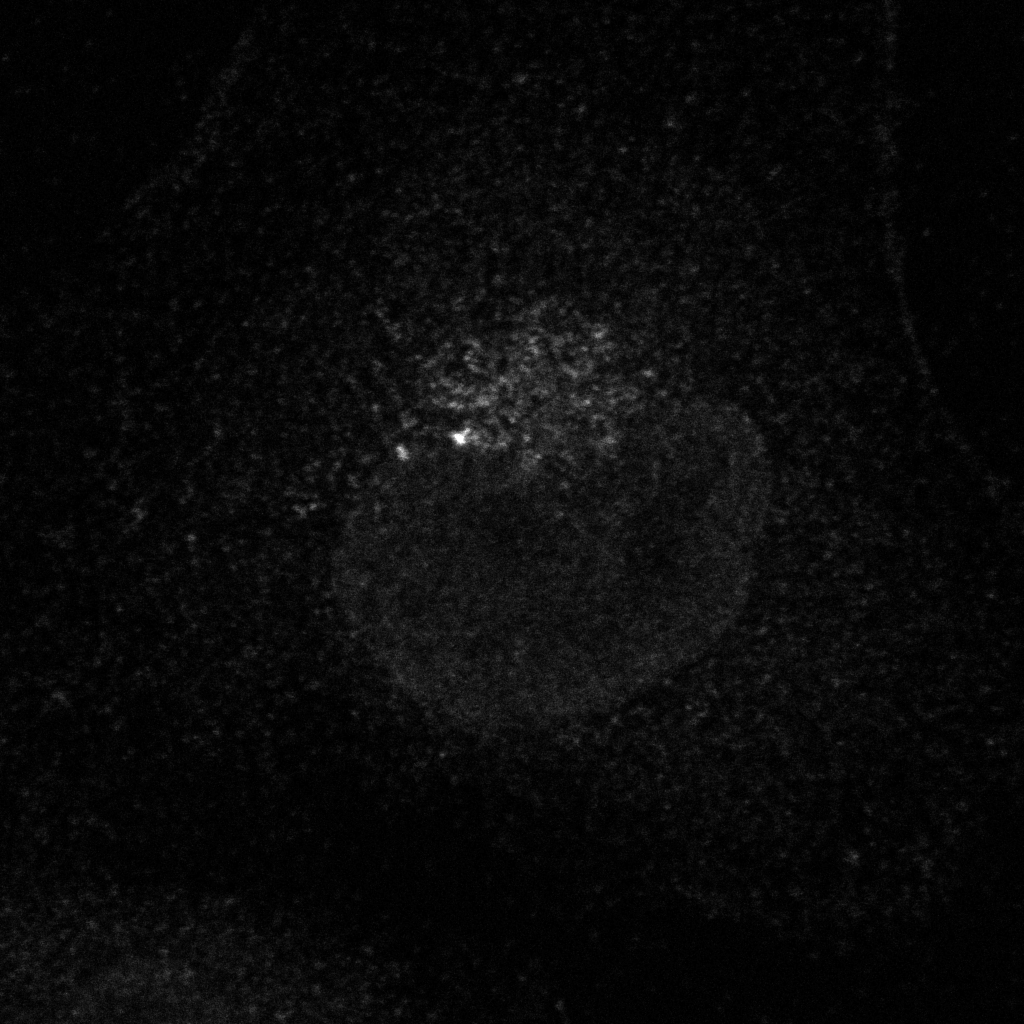

Supplement: Supplementary file 8 — Source data Fig. 6 [file 44318_2025_672_MOESM8_ESM.zip › Figure 6/6B/WT_VEH_LAMP.tif]

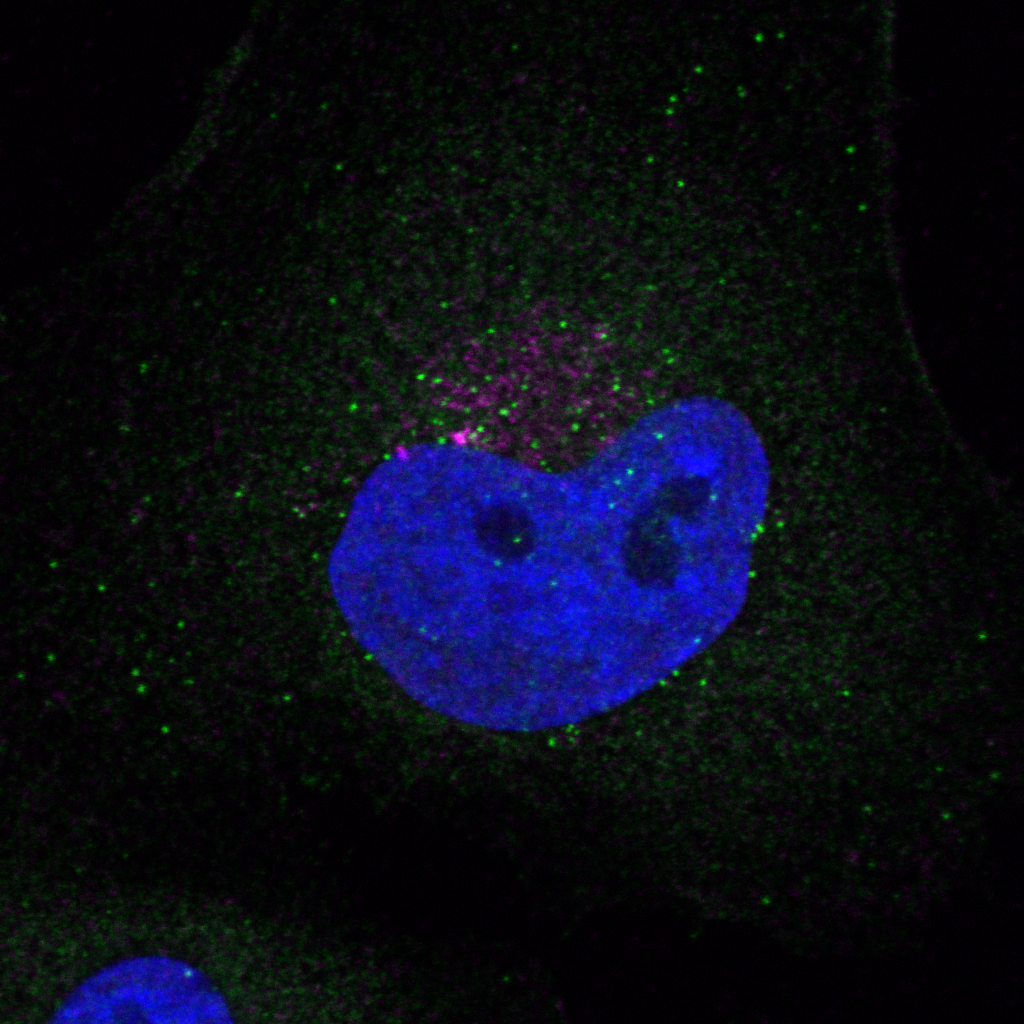

Supplement: Supplementary file 8 — Source data Fig. 6 [file 44318_2025_672_MOESM8_ESM.zip › Figure 6/6B/WT_VEH_Merge.tif]

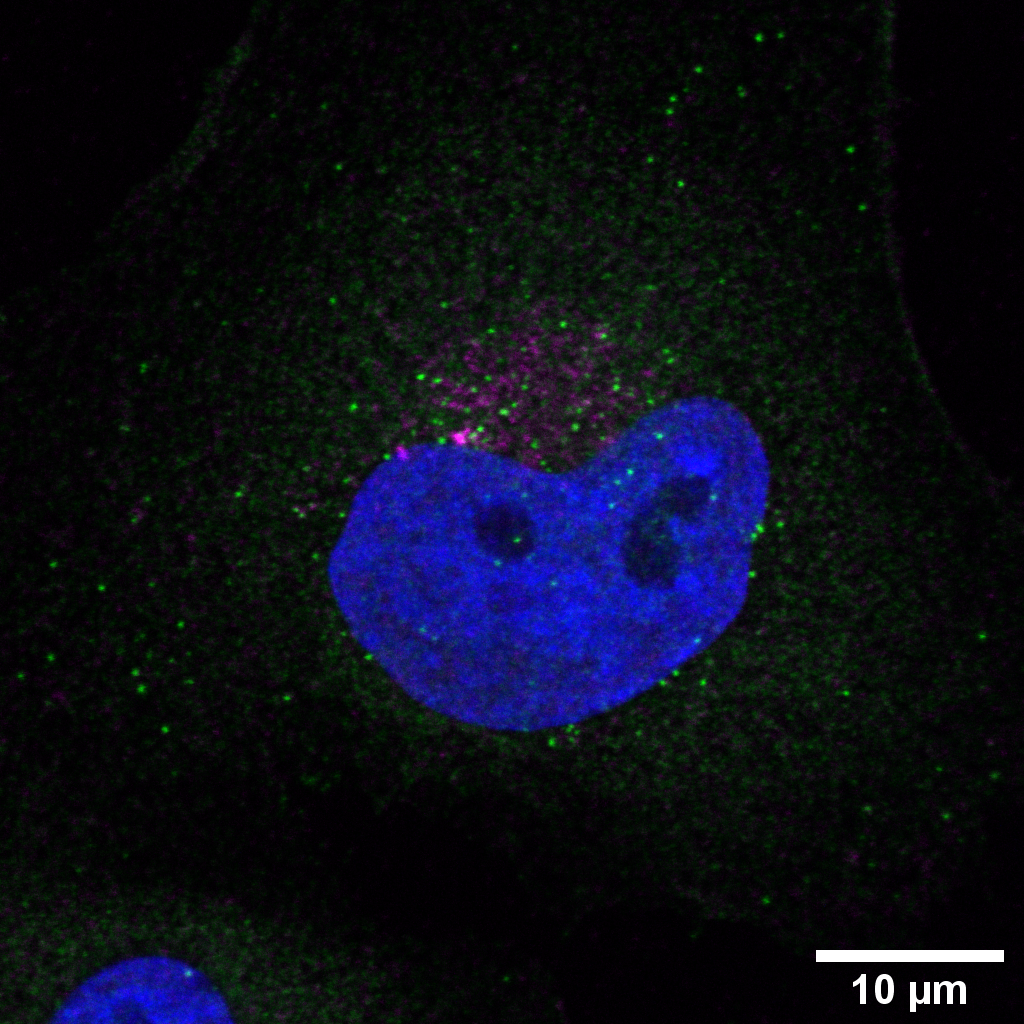

Supplement: Supplementary file 8 — Source data Fig. 6 [file 44318_2025_672_MOESM8_ESM.zip › Figure 6/6B/WT_VEH_scale.tif]

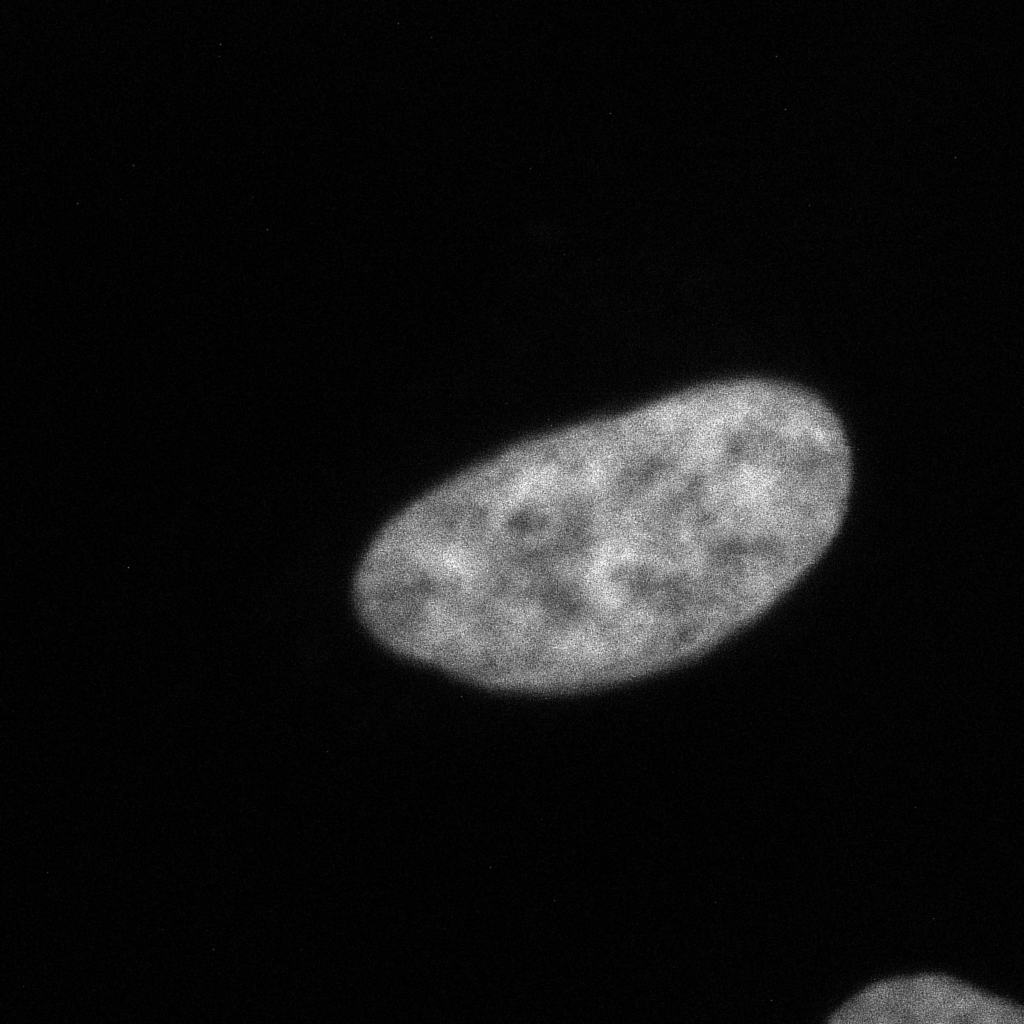

Supplement: Supplementary file 8 — Source data Fig. 6 [file 44318_2025_672_MOESM8_ESM.zip › Figure 6/6E/ALG2KO_LLOMe Wash_DAPI.tif]

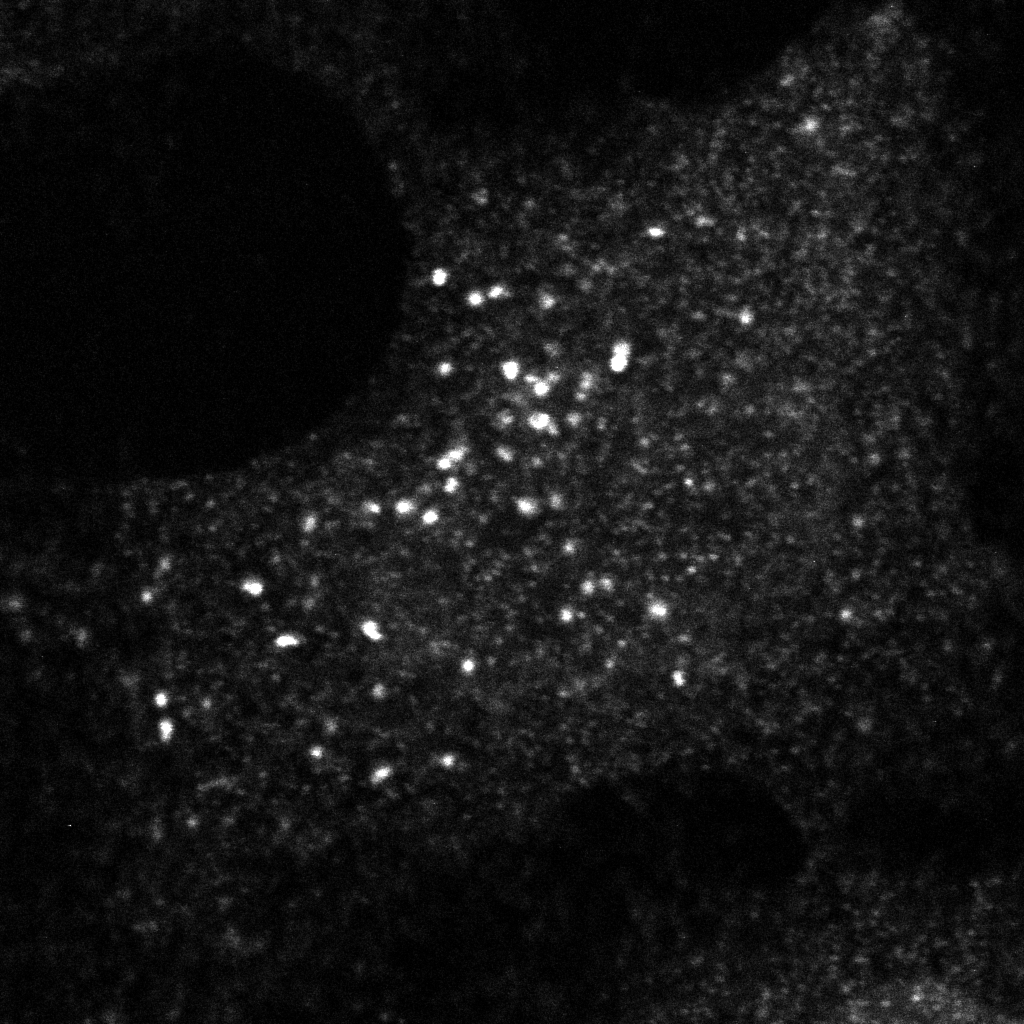

Supplement: Supplementary file 8 — Source data Fig. 6 [file 44318_2025_672_MOESM8_ESM.zip › Figure 6/6E/ALG2KO_LLOMe Wash_Gal3.tif]

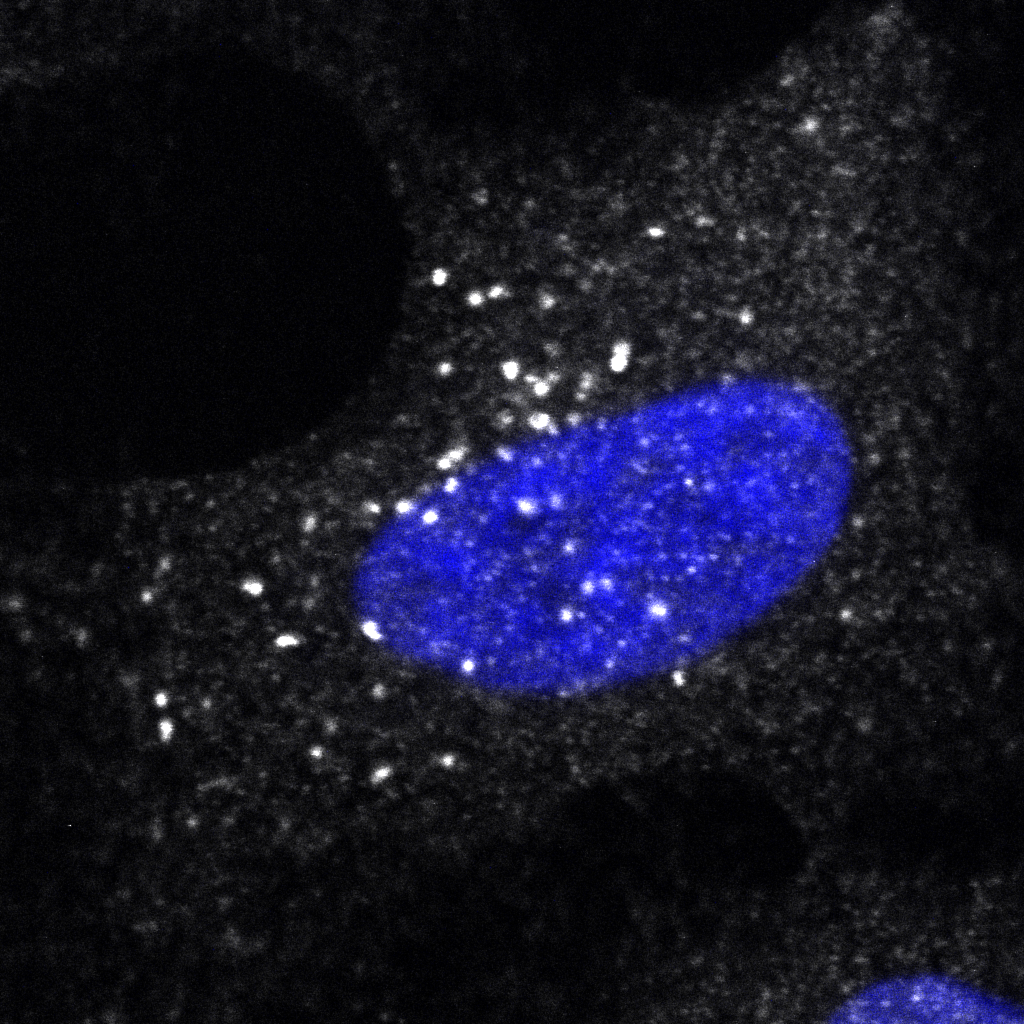

Supplement: Supplementary file 8 — Source data Fig. 6 [file 44318_2025_672_MOESM8_ESM.zip › Figure 6/6E/ALG2KO_LLOMe Wash_Merge.tif]

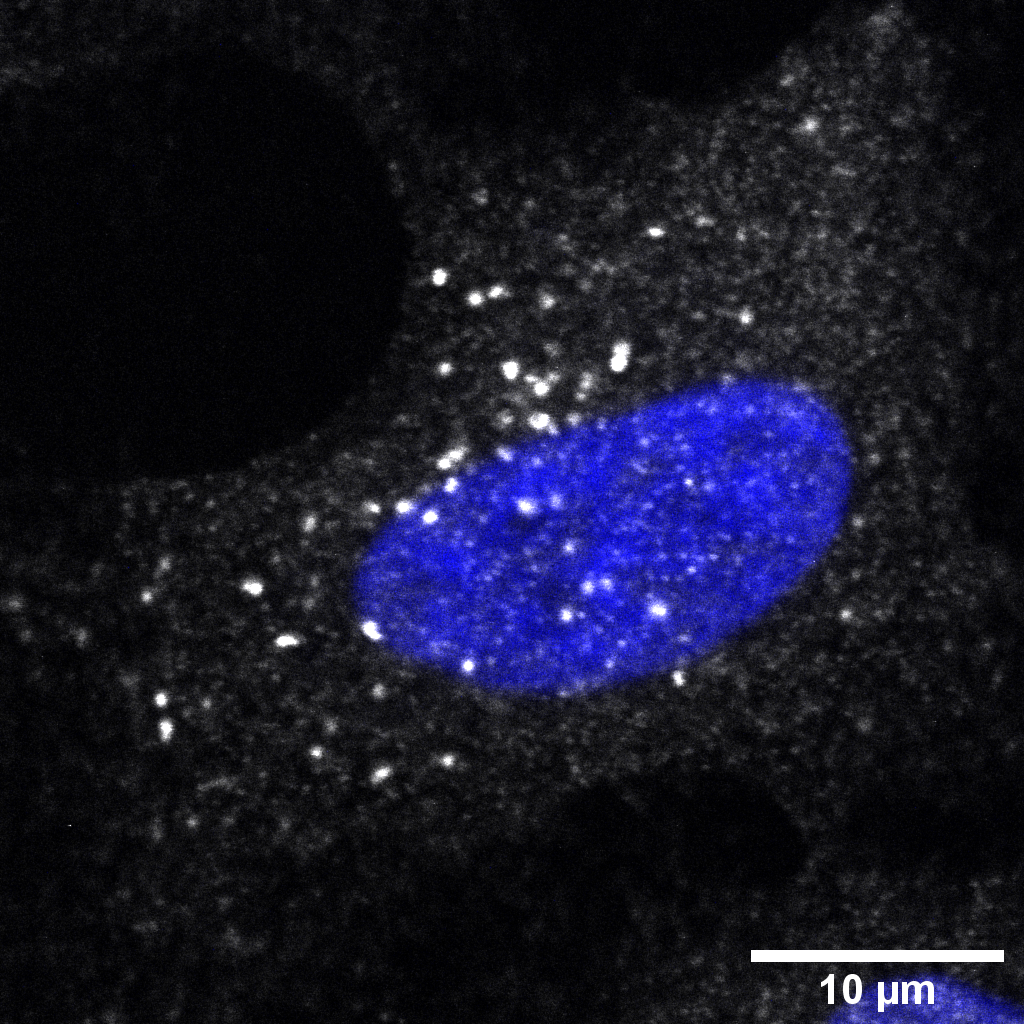

Supplement: Supplementary file 8 — Source data Fig. 6 [file 44318_2025_672_MOESM8_ESM.zip › Figure 6/6E/ALG2KO_LLOMe Wash_Scale.tif]

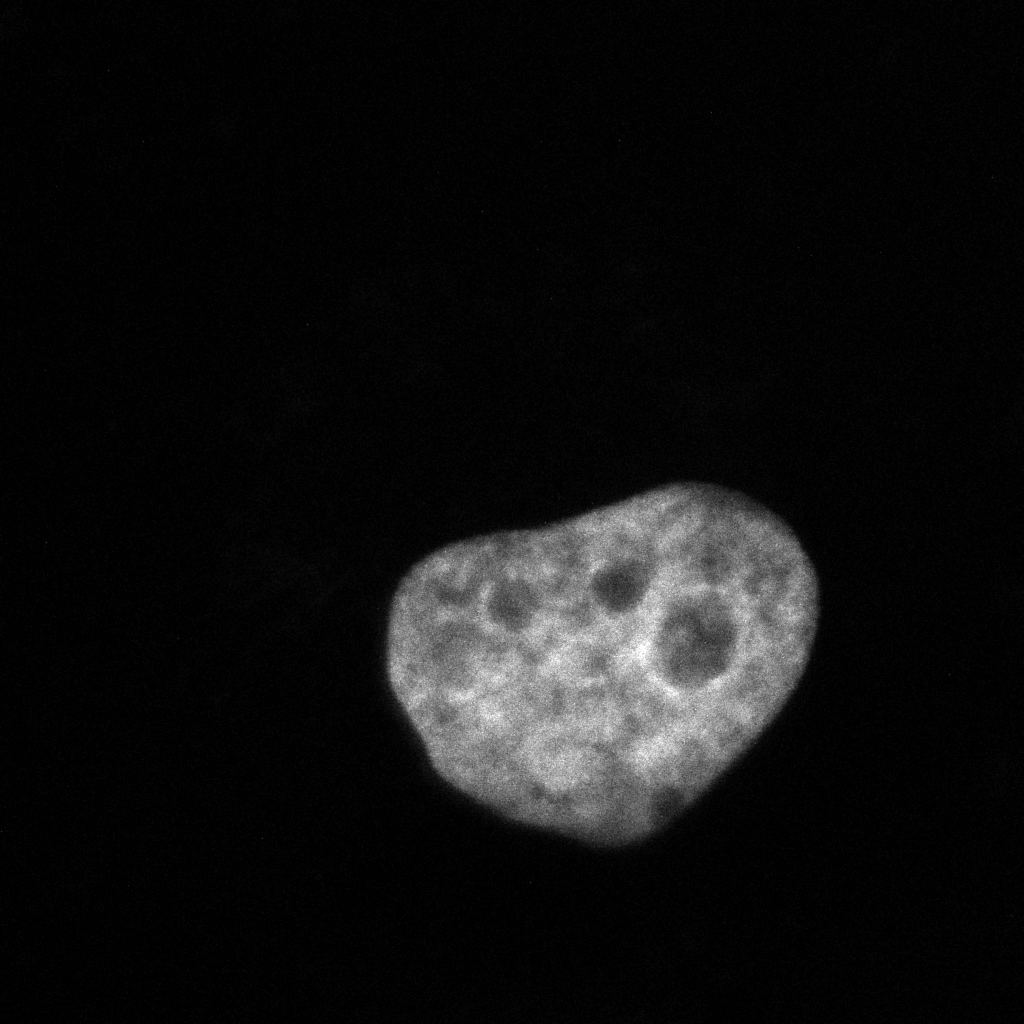

Supplement: Supplementary file 8 — Source data Fig. 6 [file 44318_2025_672_MOESM8_ESM.zip › Figure 6/6E/ALG2KO_LLOMe_DAPI.tif]

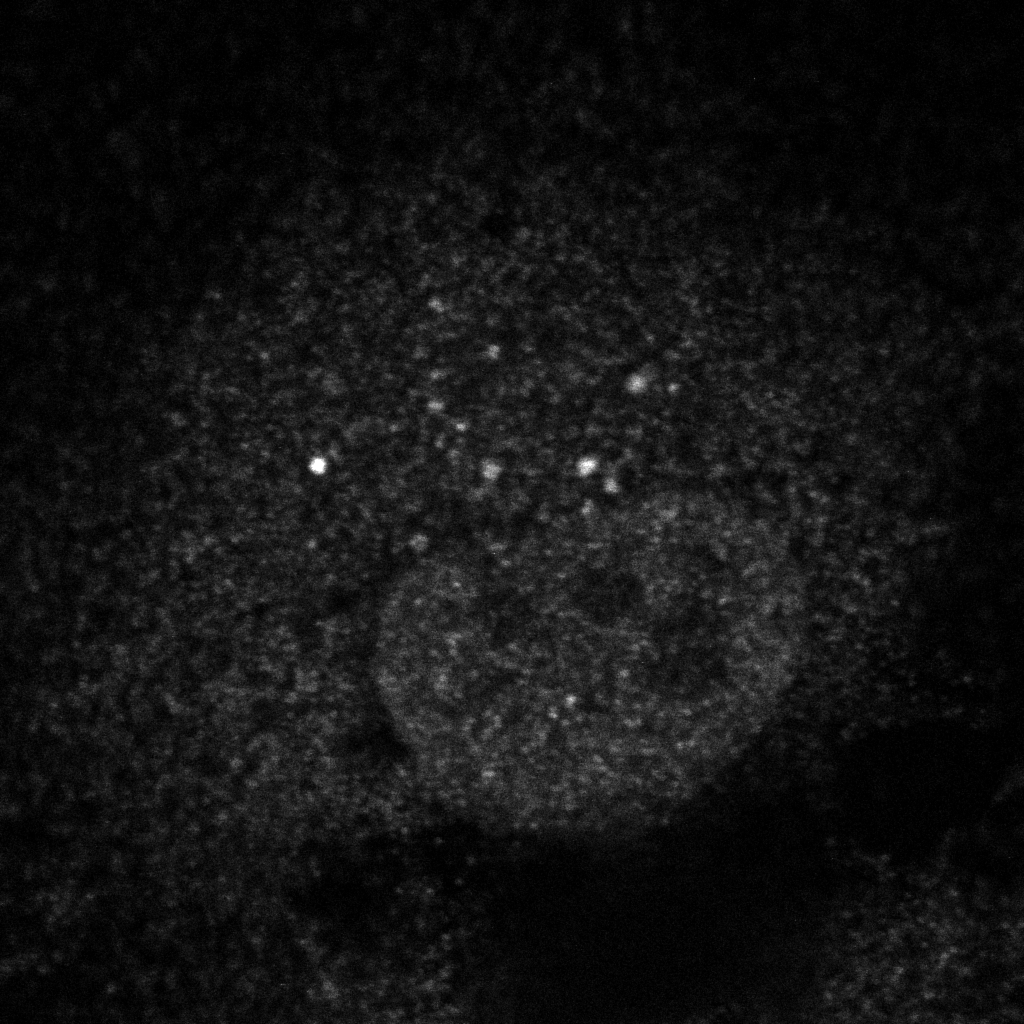

Supplement: Supplementary file 8 — Source data Fig. 6 [file 44318_2025_672_MOESM8_ESM.zip › Figure 6/6E/ALG2KO_LLOMe_Gal3.tif]

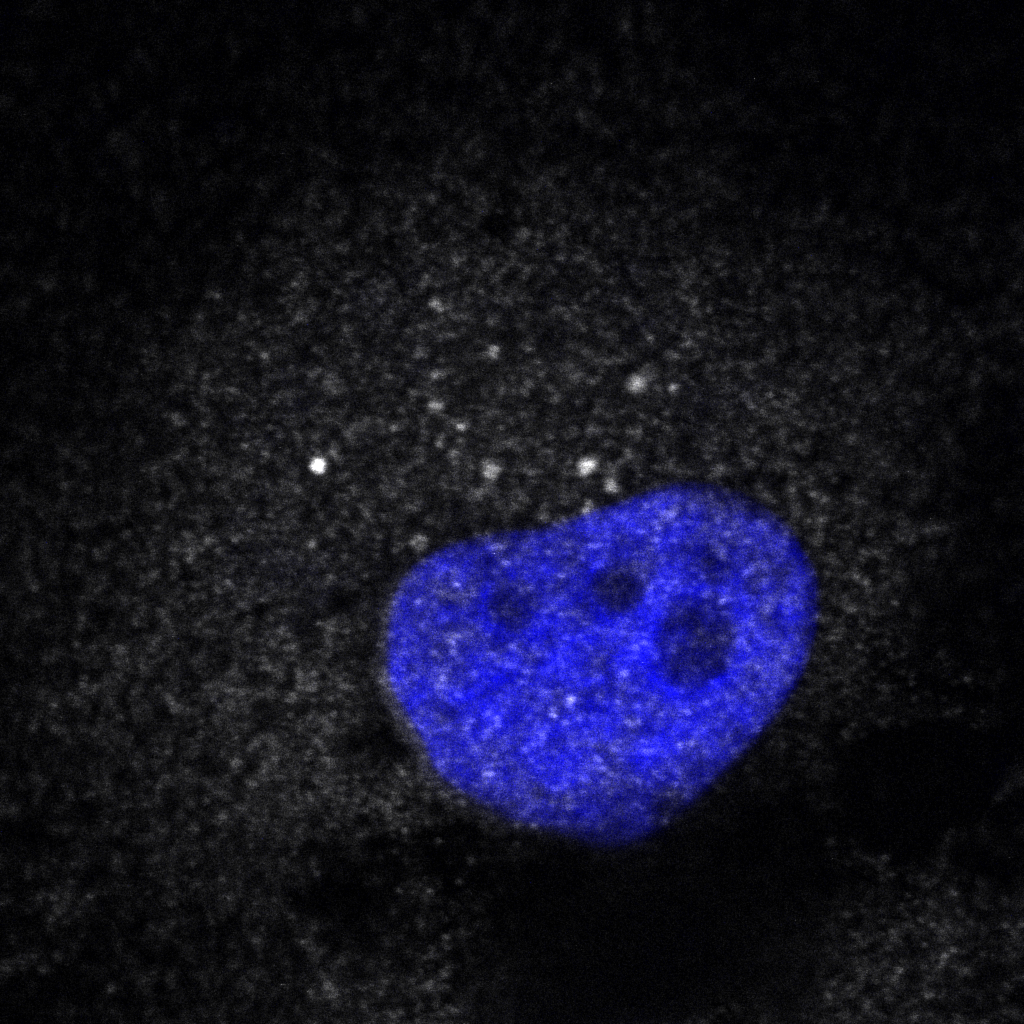

Supplement: Supplementary file 8 — Source data Fig. 6 [file 44318_2025_672_MOESM8_ESM.zip › Figure 6/6E/ALG2KO_LLOMe_Merge.tif]

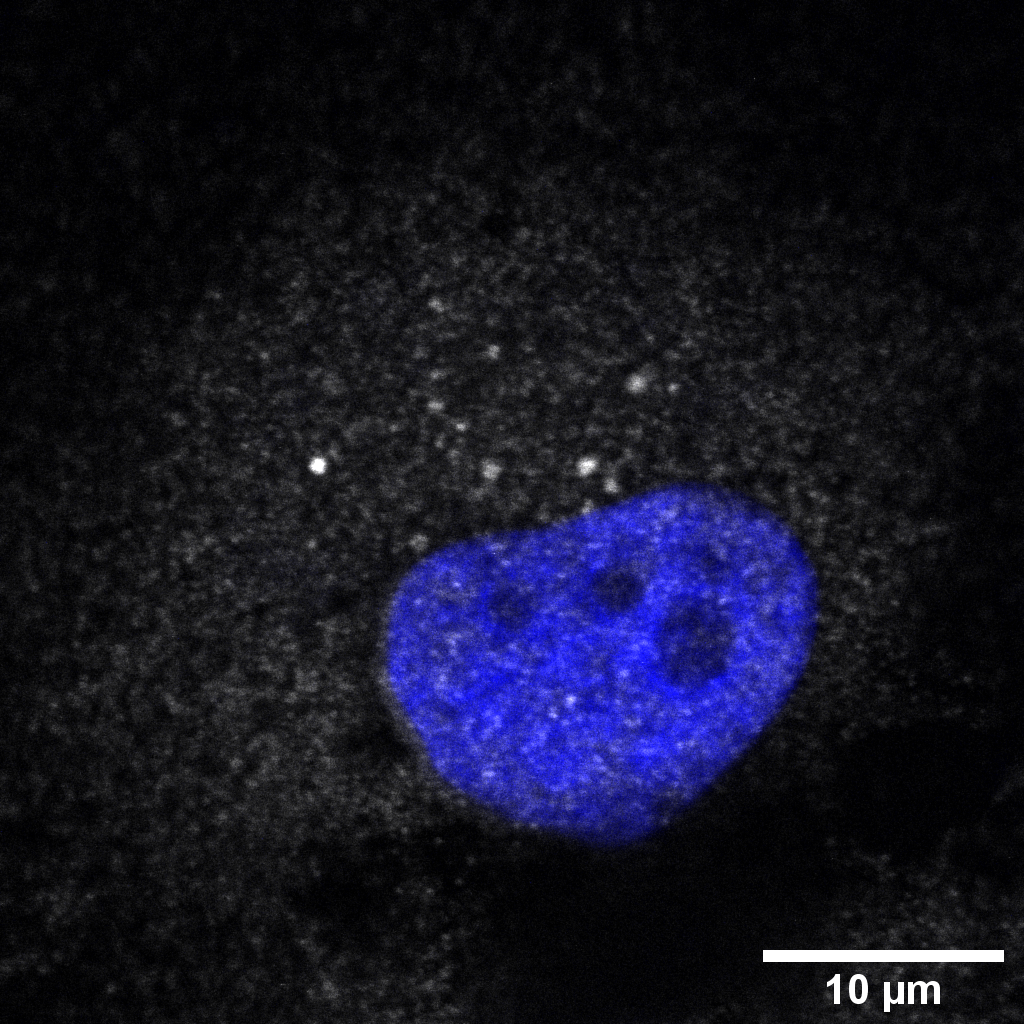

Supplement: Supplementary file 8 — Source data Fig. 6 [file 44318_2025_672_MOESM8_ESM.zip › Figure 6/6E/ALG2KO_LLOMe_Scale.tif]

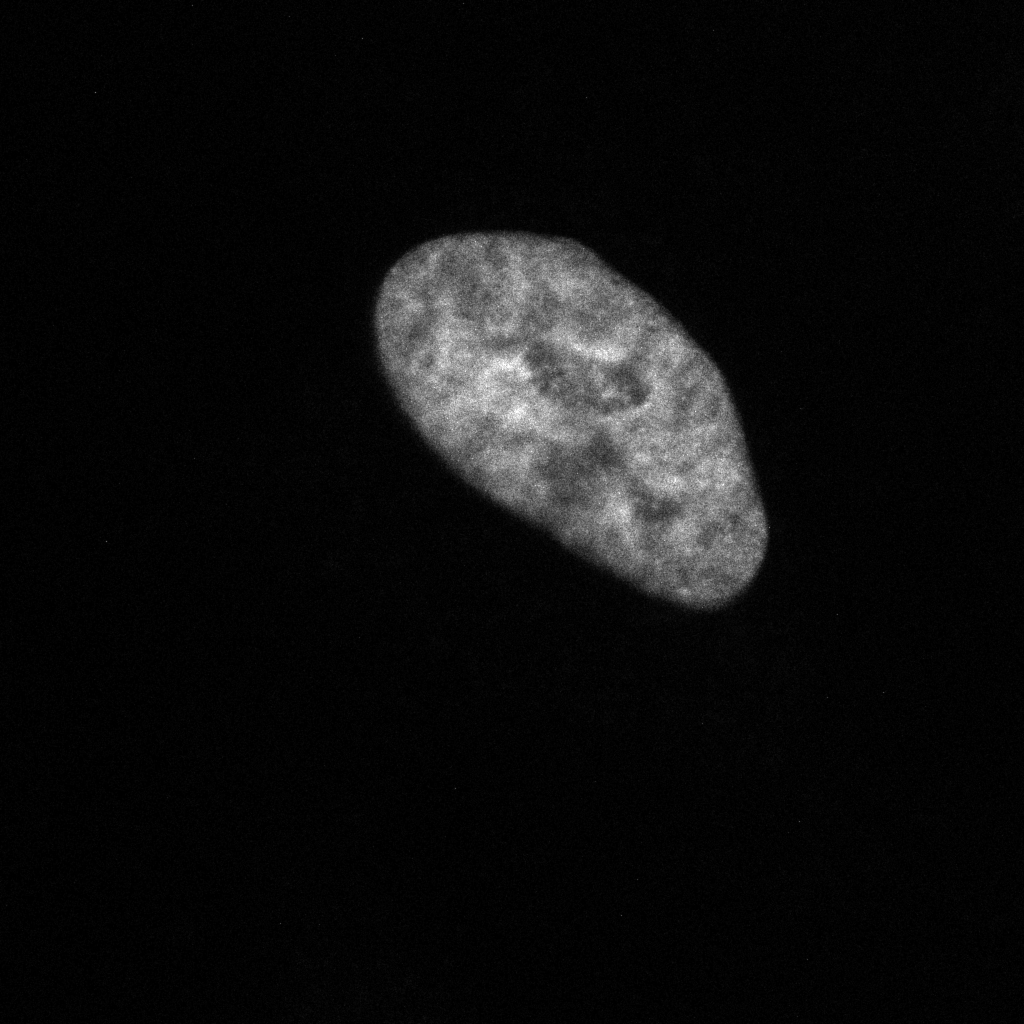

Supplement: Supplementary file 8 — Source data Fig. 6 [file 44318_2025_672_MOESM8_ESM.zip › Figure 6/6E/ALG2KO_UNT_DAPI.tif]

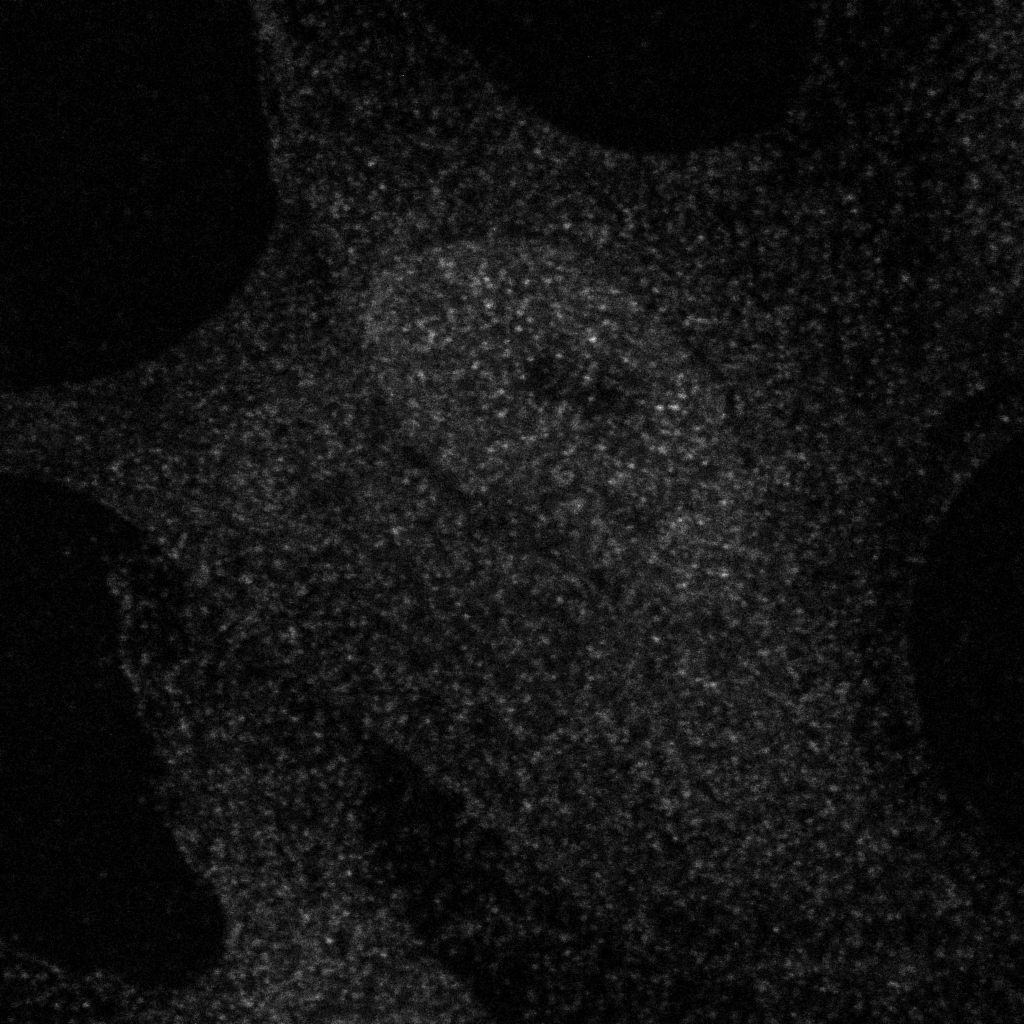

Supplement: Supplementary file 8 — Source data Fig. 6 [file 44318_2025_672_MOESM8_ESM.zip › Figure 6/6E/ALG2KO_UNT_Gal3.tif]

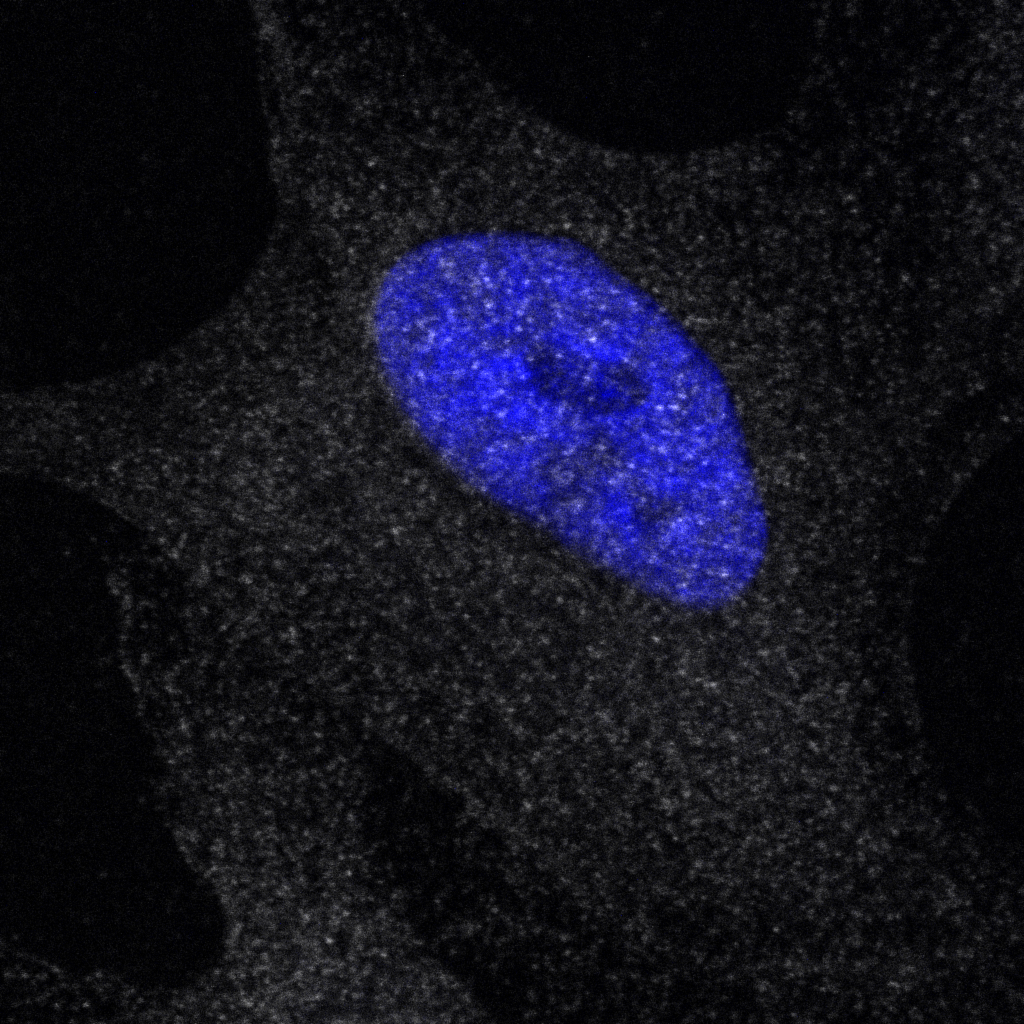

Supplement: Supplementary file 8 — Source data Fig. 6 [file 44318_2025_672_MOESM8_ESM.zip › Figure 6/6E/ALG2KO_UNT_Merge.tif]

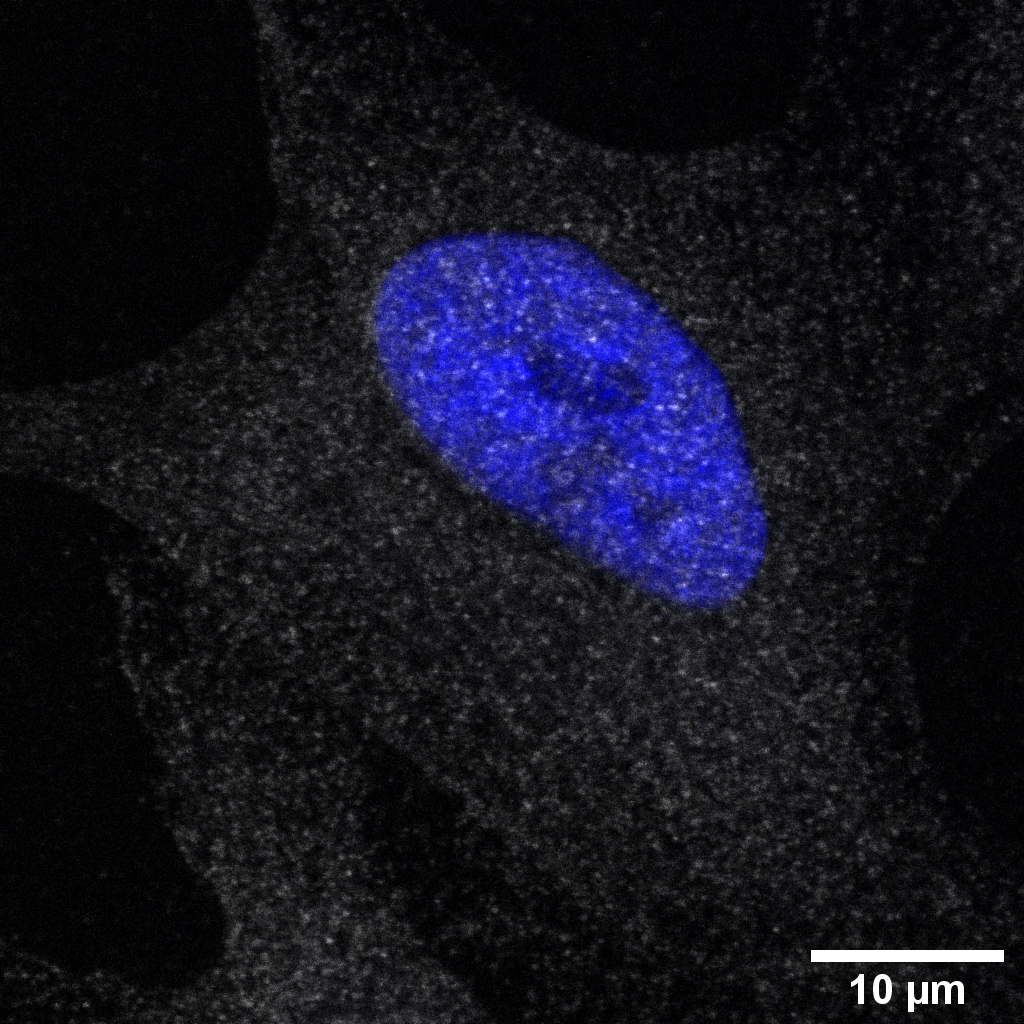

Supplement: Supplementary file 8 — Source data Fig. 6 [file 44318_2025_672_MOESM8_ESM.zip › Figure 6/6E/ALG2KO_UNT_Scale.tif]

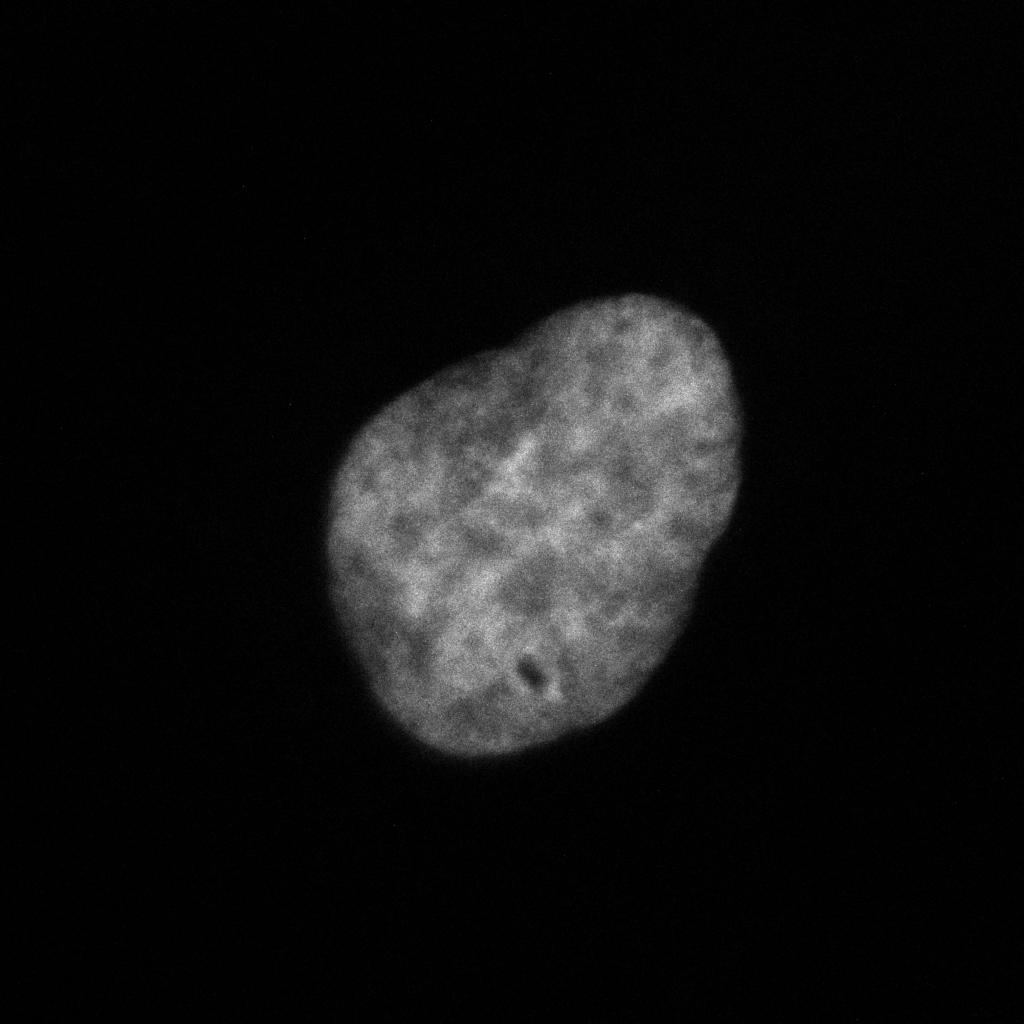

Supplement: Supplementary file 8 — Source data Fig. 6 [file 44318_2025_672_MOESM8_ESM.zip › Figure 6/6E/WT_LLOMe Wash_DAPI.tif]

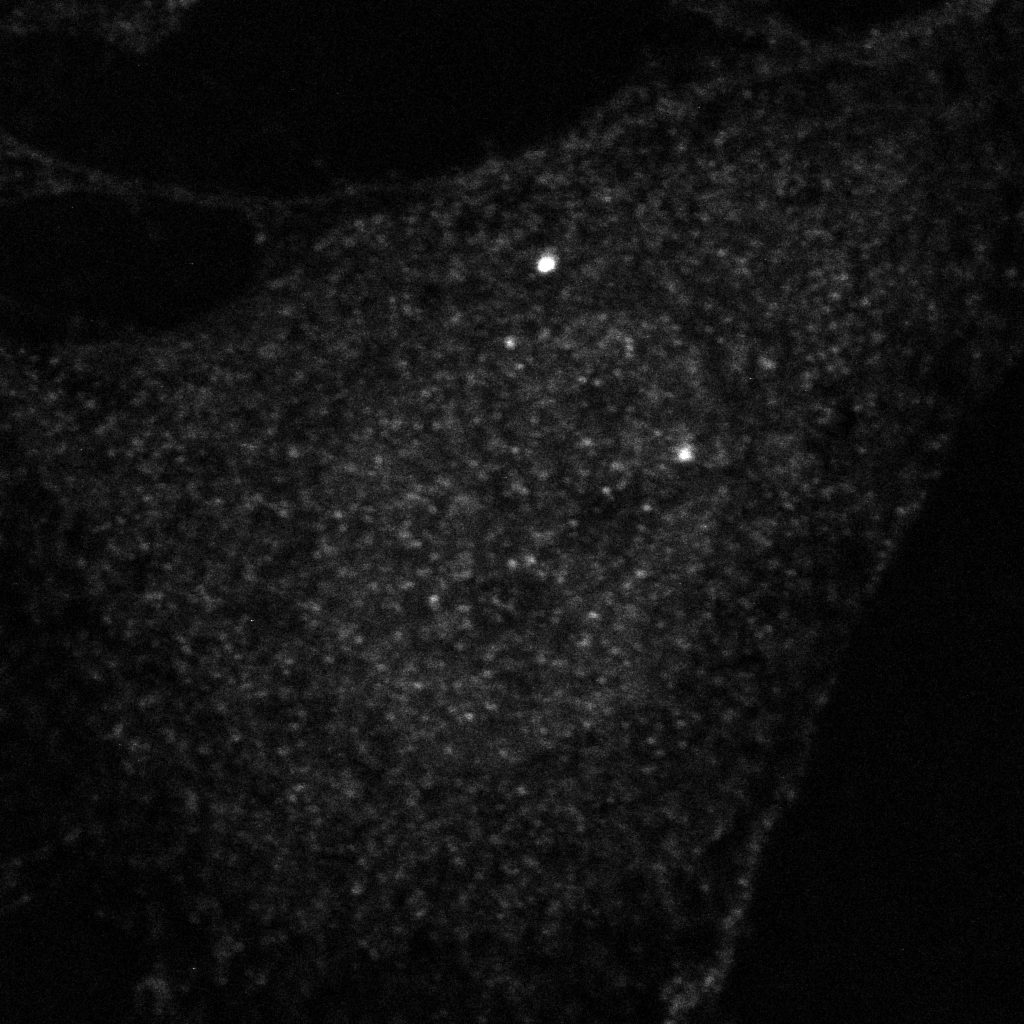

Supplement: Supplementary file 8 — Source data Fig. 6 [file 44318_2025_672_MOESM8_ESM.zip › Figure 6/6E/WT_LLOMe Wash_Gal3.tif]

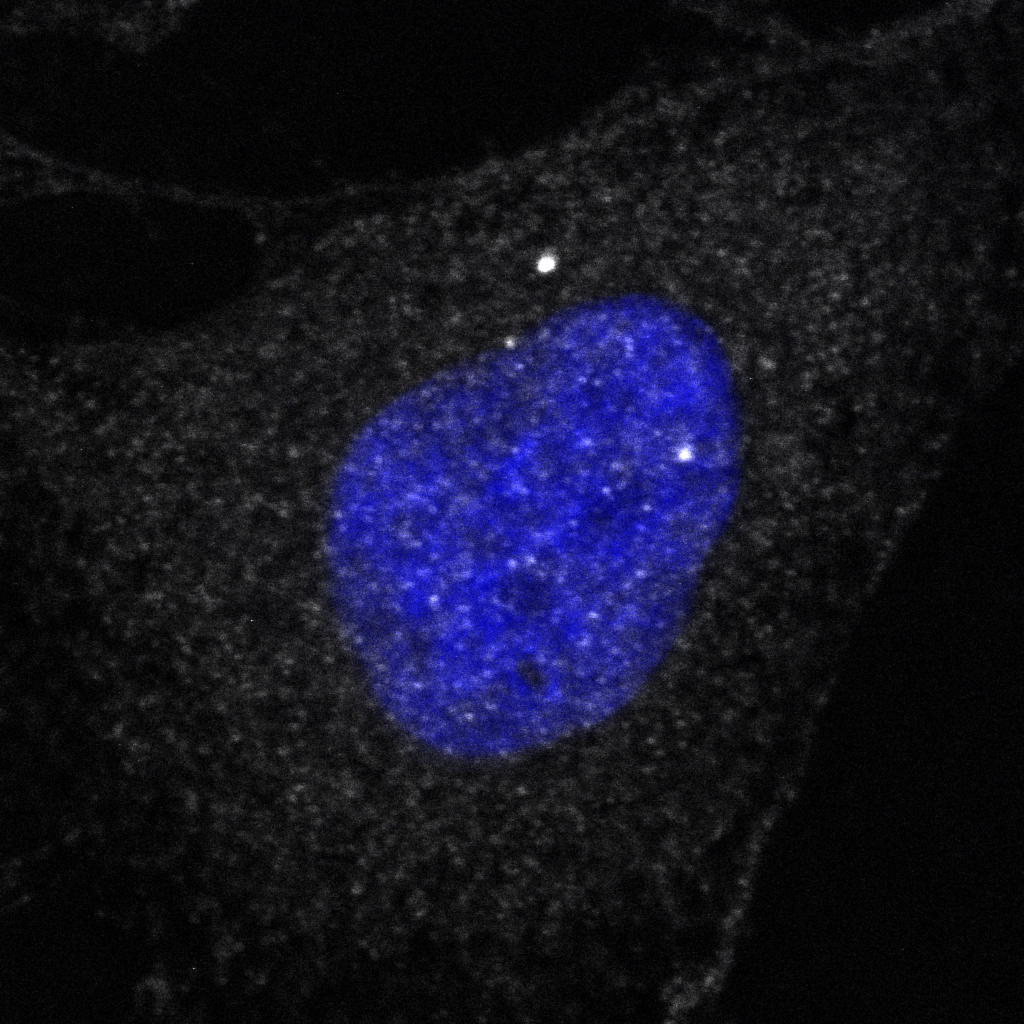

Supplement: Supplementary file 8 — Source data Fig. 6 [file 44318_2025_672_MOESM8_ESM.zip › Figure 6/6E/WT_LLOMe Wash_Merge.tif]

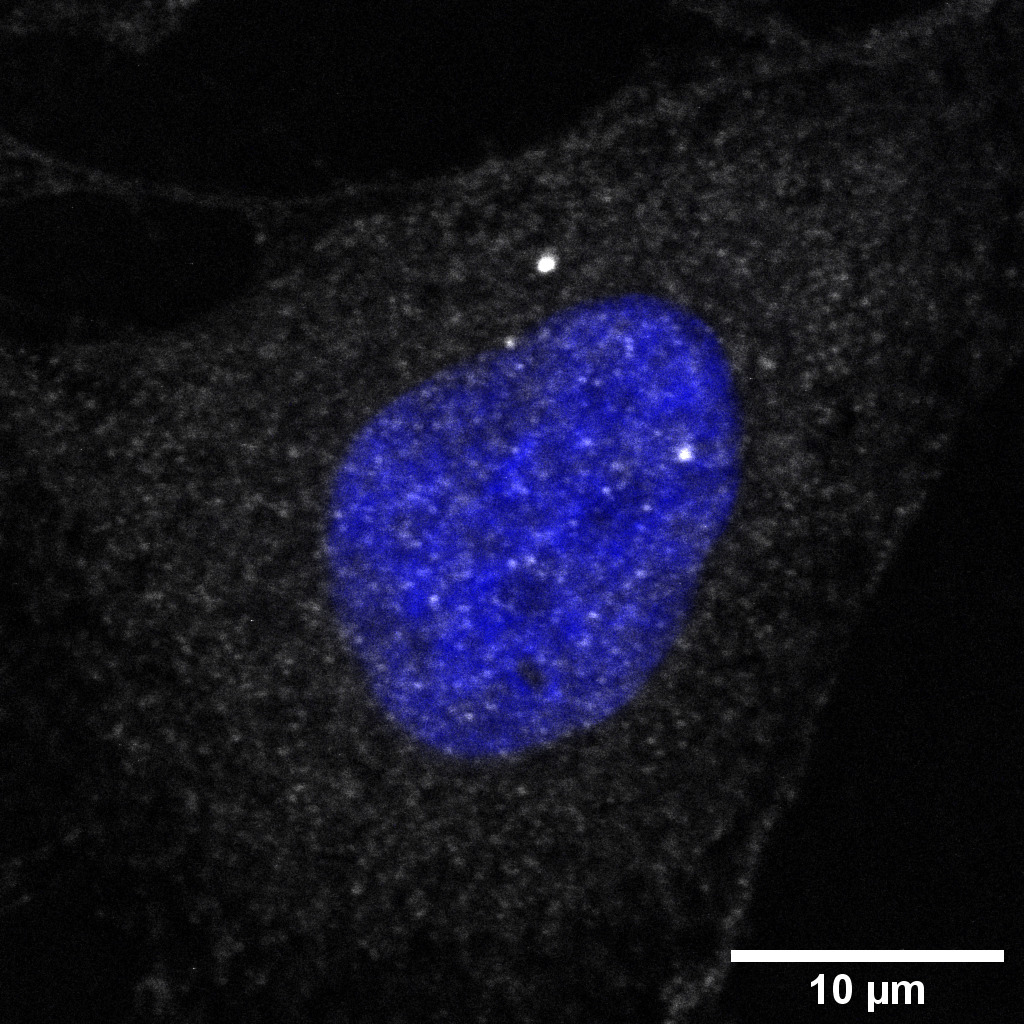

Supplement: Supplementary file 8 — Source data Fig. 6 [file 44318_2025_672_MOESM8_ESM.zip › Figure 6/6E/WT_LLOMe Wash_Scale.tif]

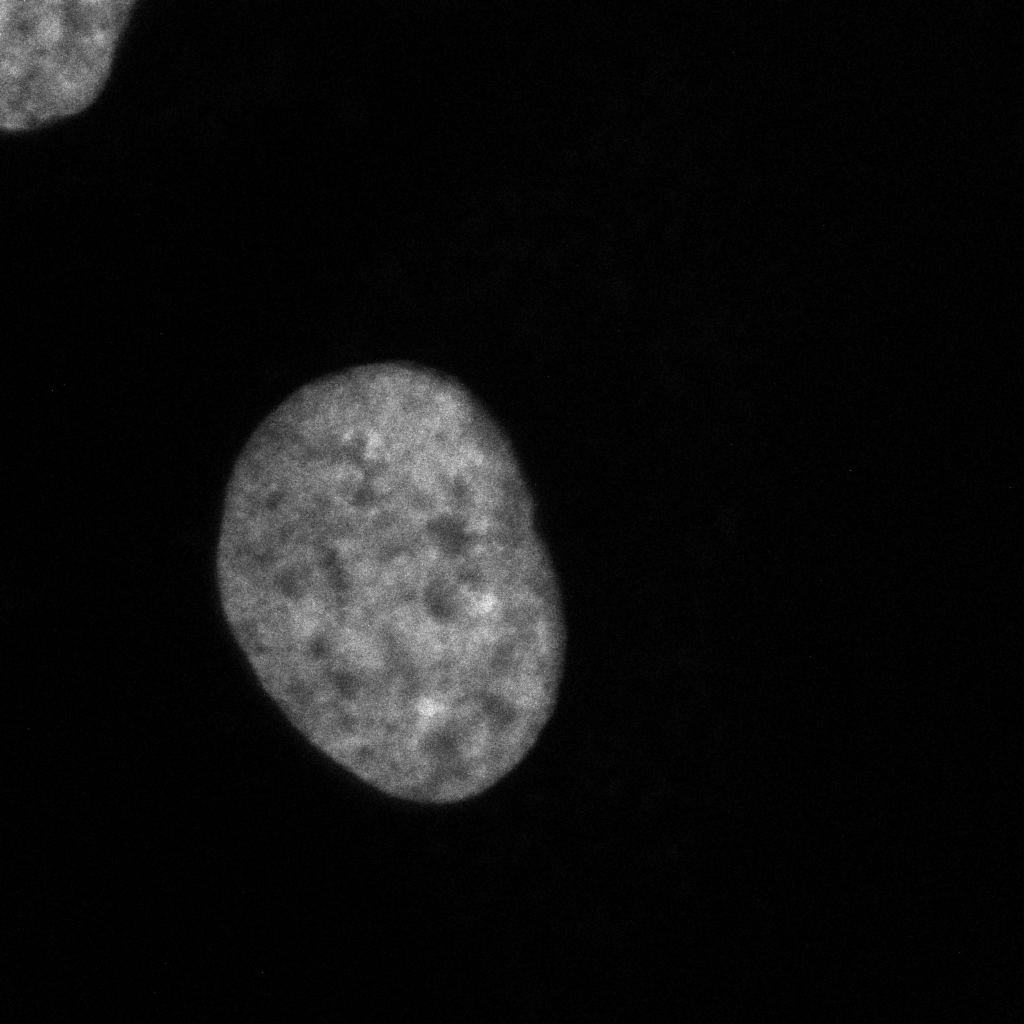

Supplement: Supplementary file 8 — Source data Fig. 6 [file 44318_2025_672_MOESM8_ESM.zip › Figure 6/6E/WT_LLOMe_DAPI.tif]

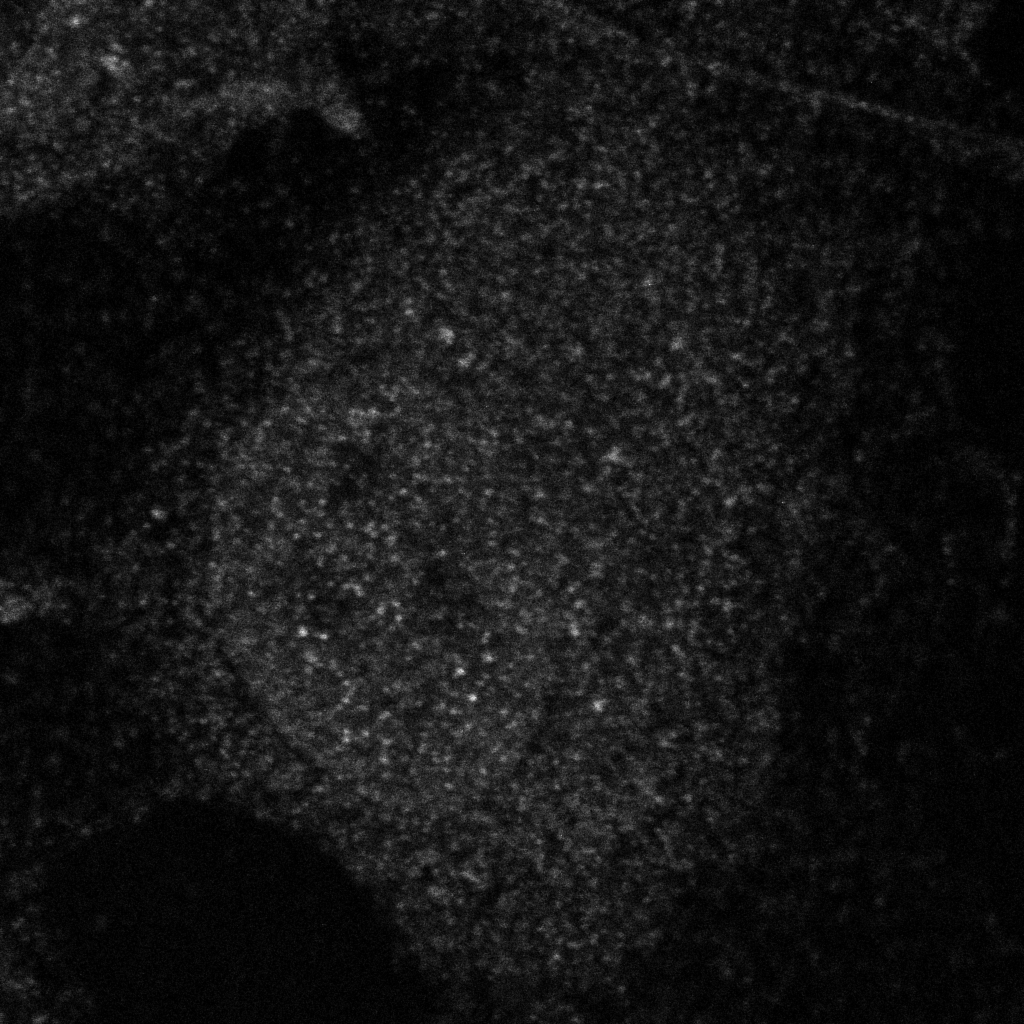

Supplement: Supplementary file 8 — Source data Fig. 6 [file 44318_2025_672_MOESM8_ESM.zip › Figure 6/6E/WT_LLOMe_Gal3.tif]

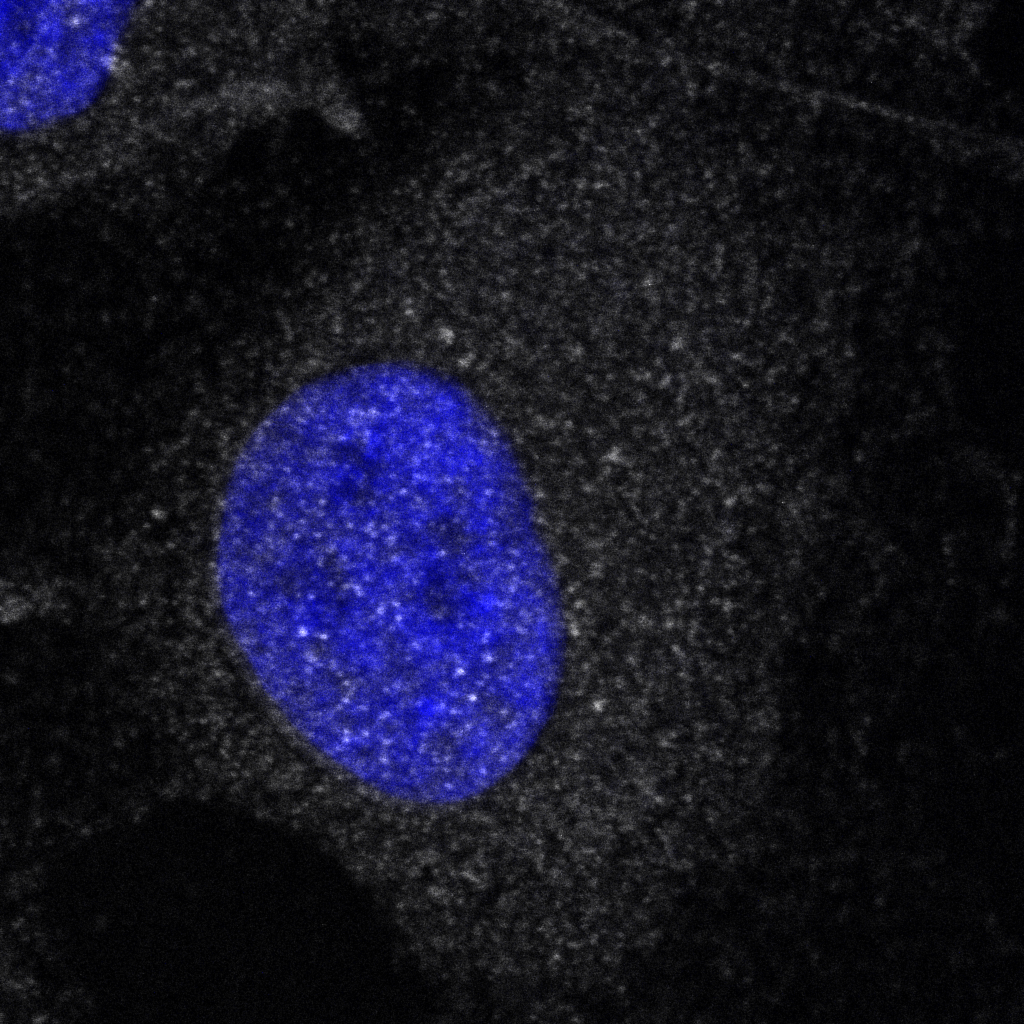

Supplement: Supplementary file 8 — Source data Fig. 6 [file 44318_2025_672_MOESM8_ESM.zip › Figure 6/6E/WT_LLOMe_Merge.tif]

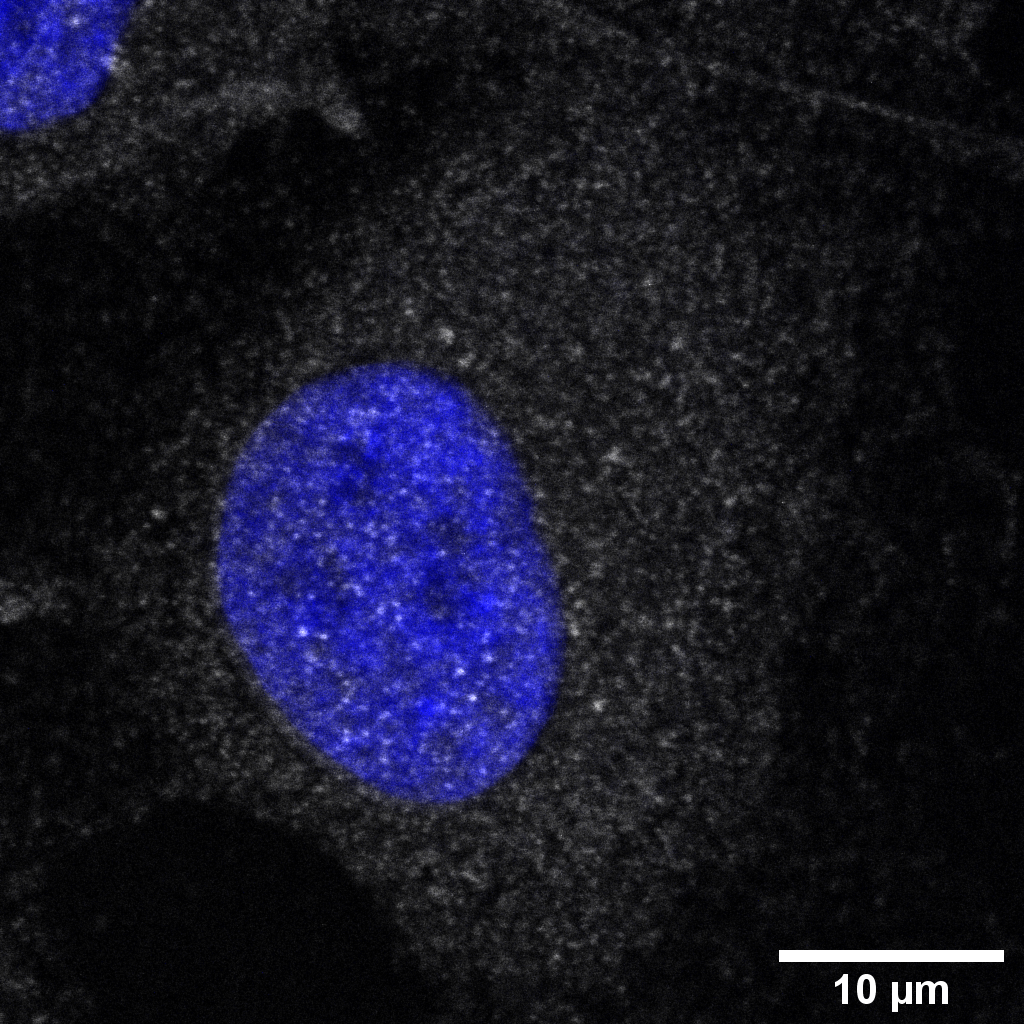

Supplement: Supplementary file 8 — Source data Fig. 6 [file 44318_2025_672_MOESM8_ESM.zip › Figure 6/6E/WT_LLOMe_Scale.tif]

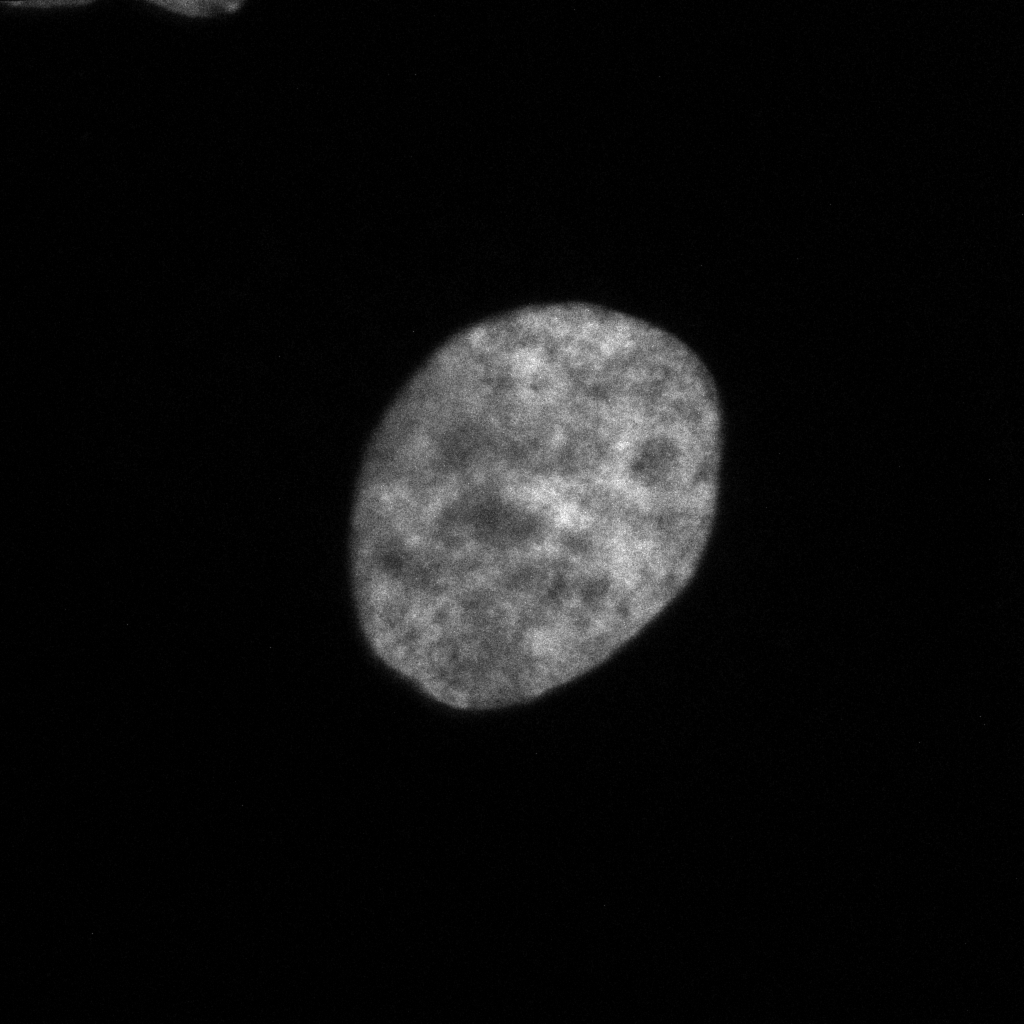

Supplement: Supplementary file 8 — Source data Fig. 6 [file 44318_2025_672_MOESM8_ESM.zip › Figure 6/6E/WT_UNT_DAPI.tif]

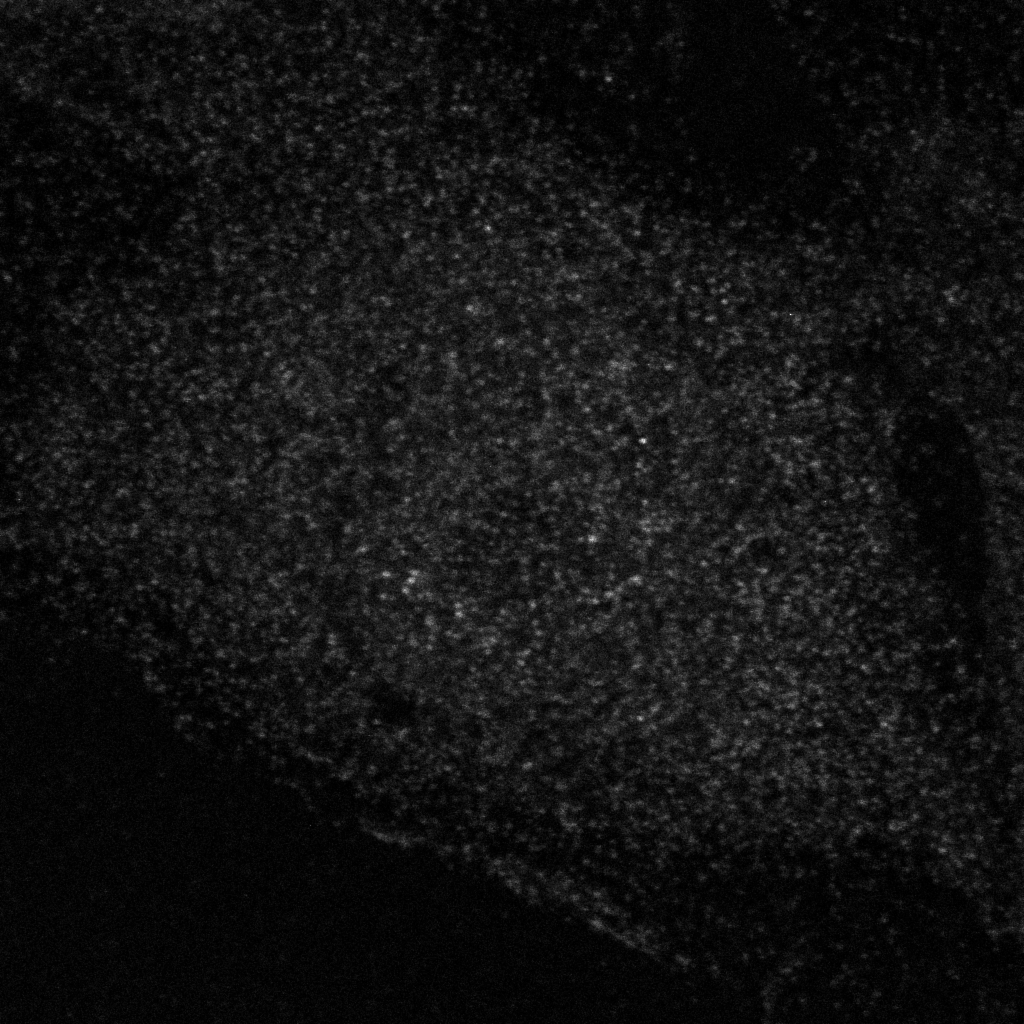

Supplement: Supplementary file 8 — Source data Fig. 6 [file 44318_2025_672_MOESM8_ESM.zip › Figure 6/6E/WT_UNT_Gal3.tif]

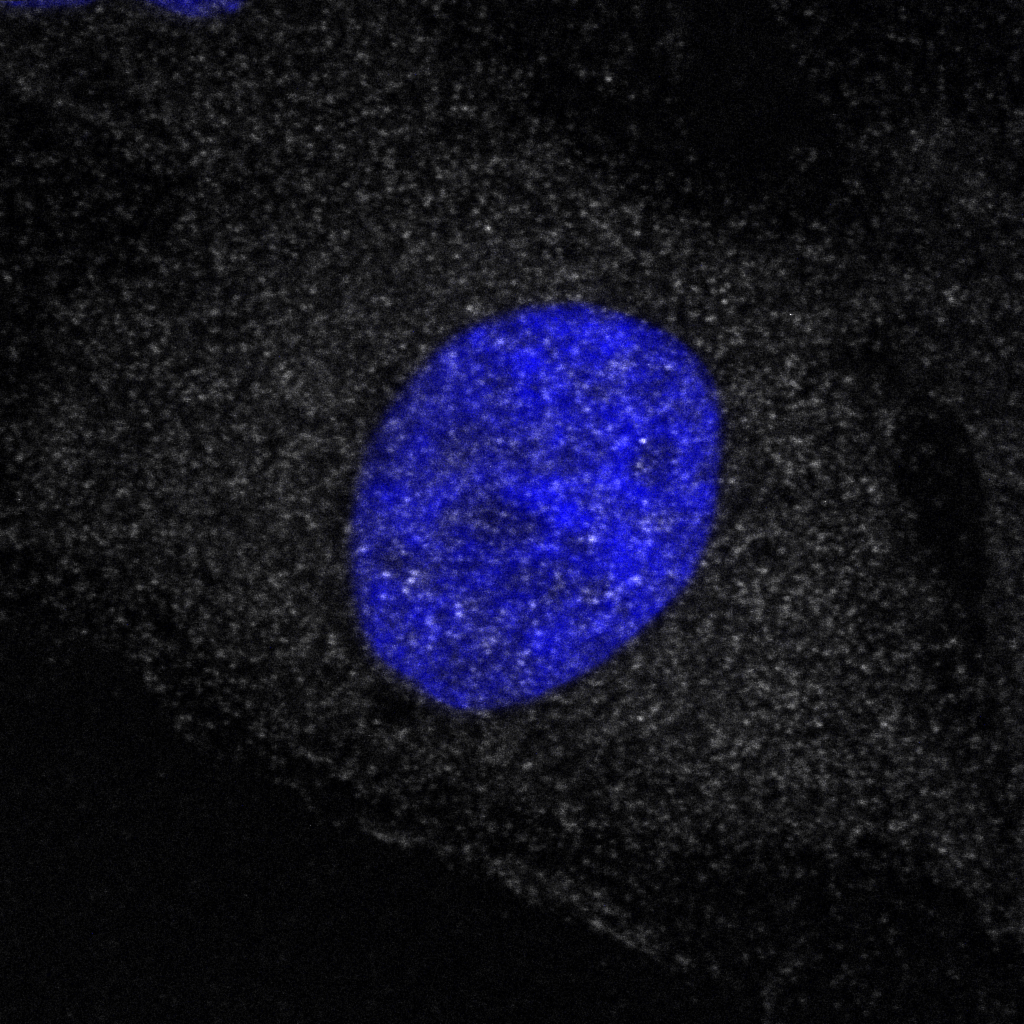

Supplement: Supplementary file 8 — Source data Fig. 6 [file 44318_2025_672_MOESM8_ESM.zip › Figure 6/6E/WT_UNT_Merge.tif]

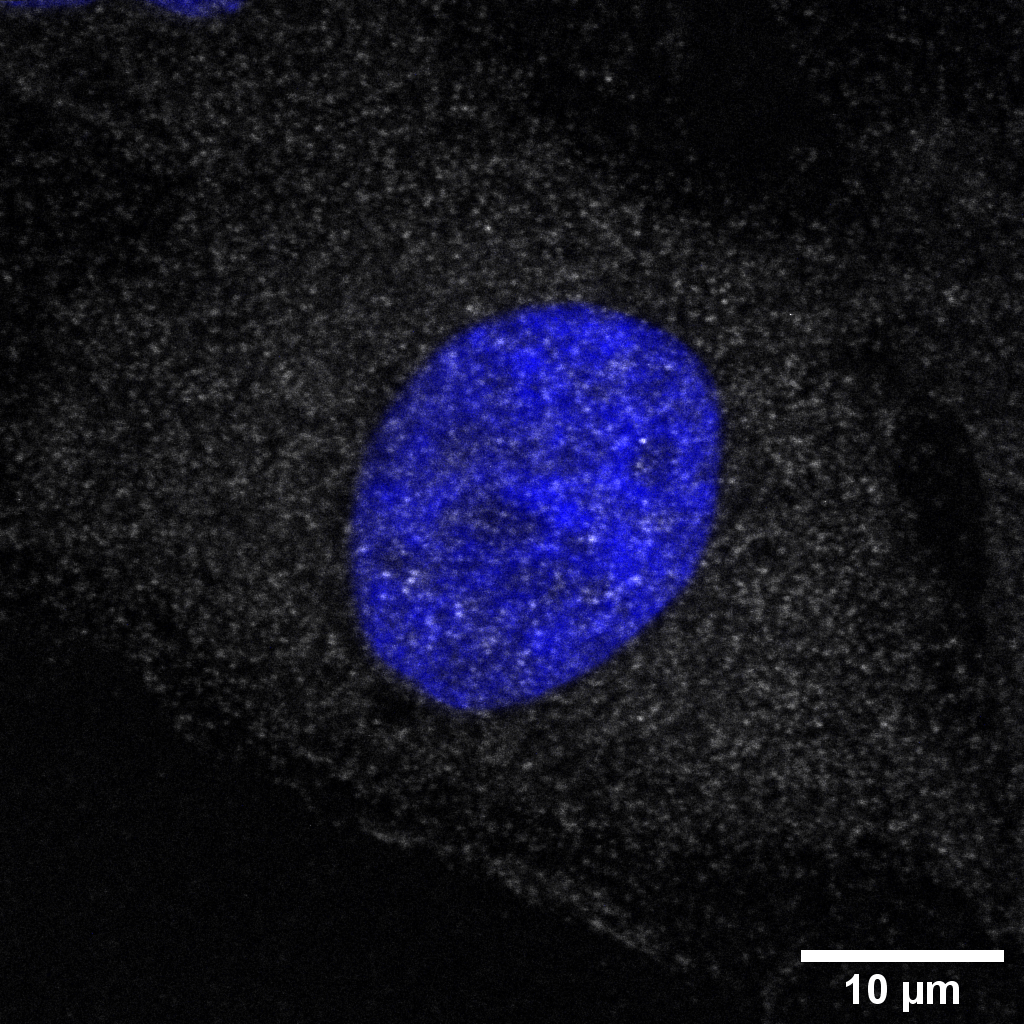

Supplement: Supplementary file 8 — Source data Fig. 6 [file 44318_2025_672_MOESM8_ESM.zip › Figure 6/6E/WT_UNT_Scale.tif]

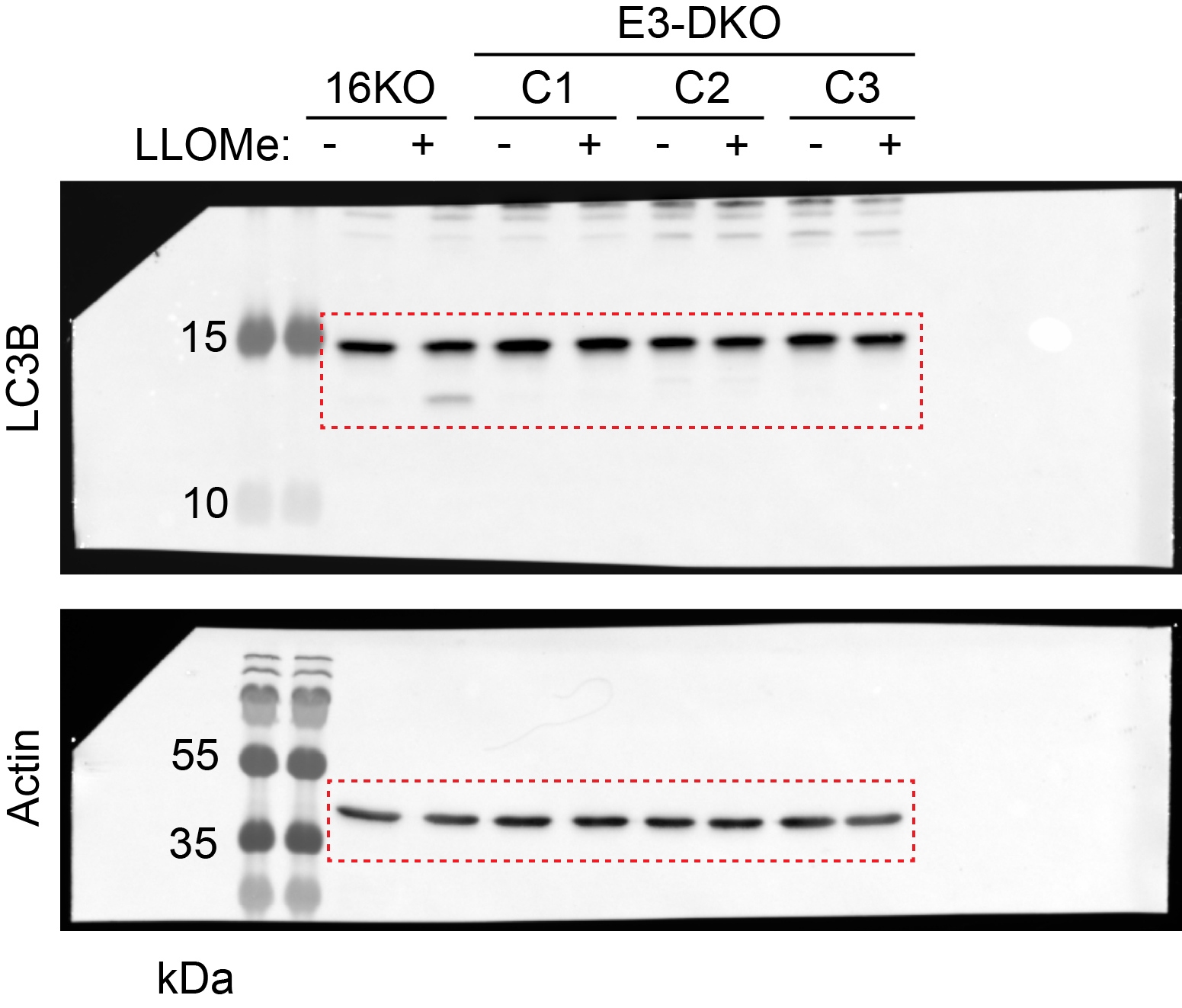

Supplement: Supplementary file 9 — Figure EV1-5 Source Data [file 44318_2025_672_MOESM9_ESM.zip › EV Source Data/EV1/EV1A/EV1A_uncropped blots.jpg]

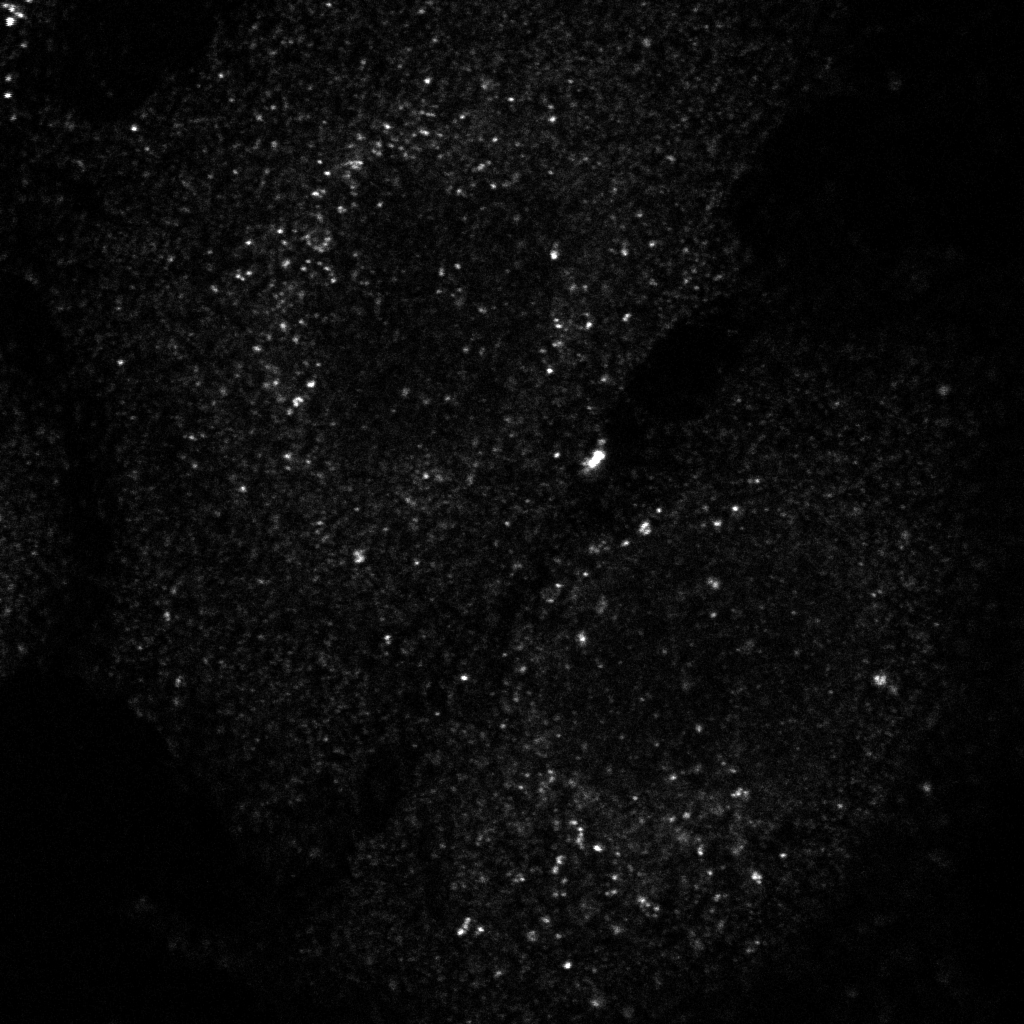

Supplement: Supplementary file 9 — Figure EV1-5 Source Data [file 44318_2025_672_MOESM9_ESM.zip › EV Source Data/EV1/EV1B/DKO1_ALIX.tif]

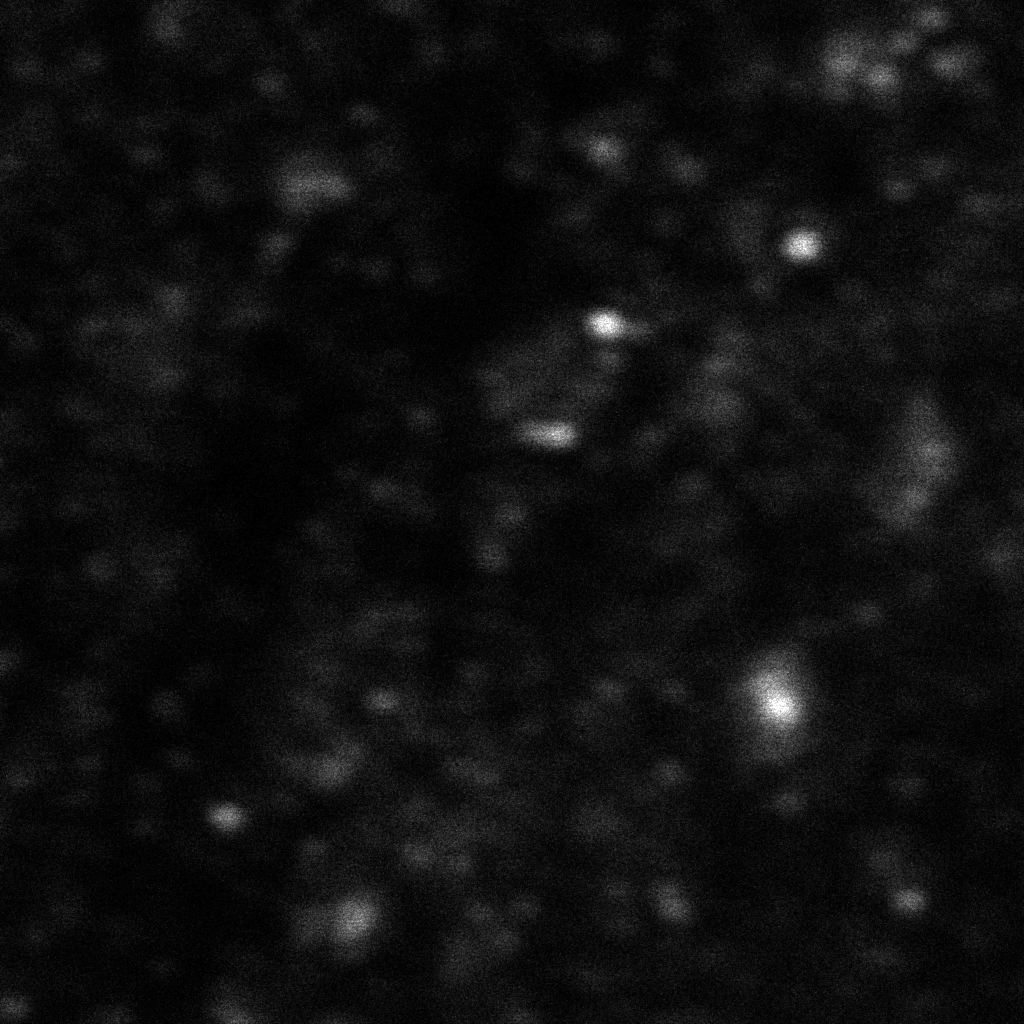

Supplement: Supplementary file 9 — Figure EV1-5 Source Data [file 44318_2025_672_MOESM9_ESM.zip › EV Source Data/EV1/EV1B/DKO1_ALIX_zoom.tif]

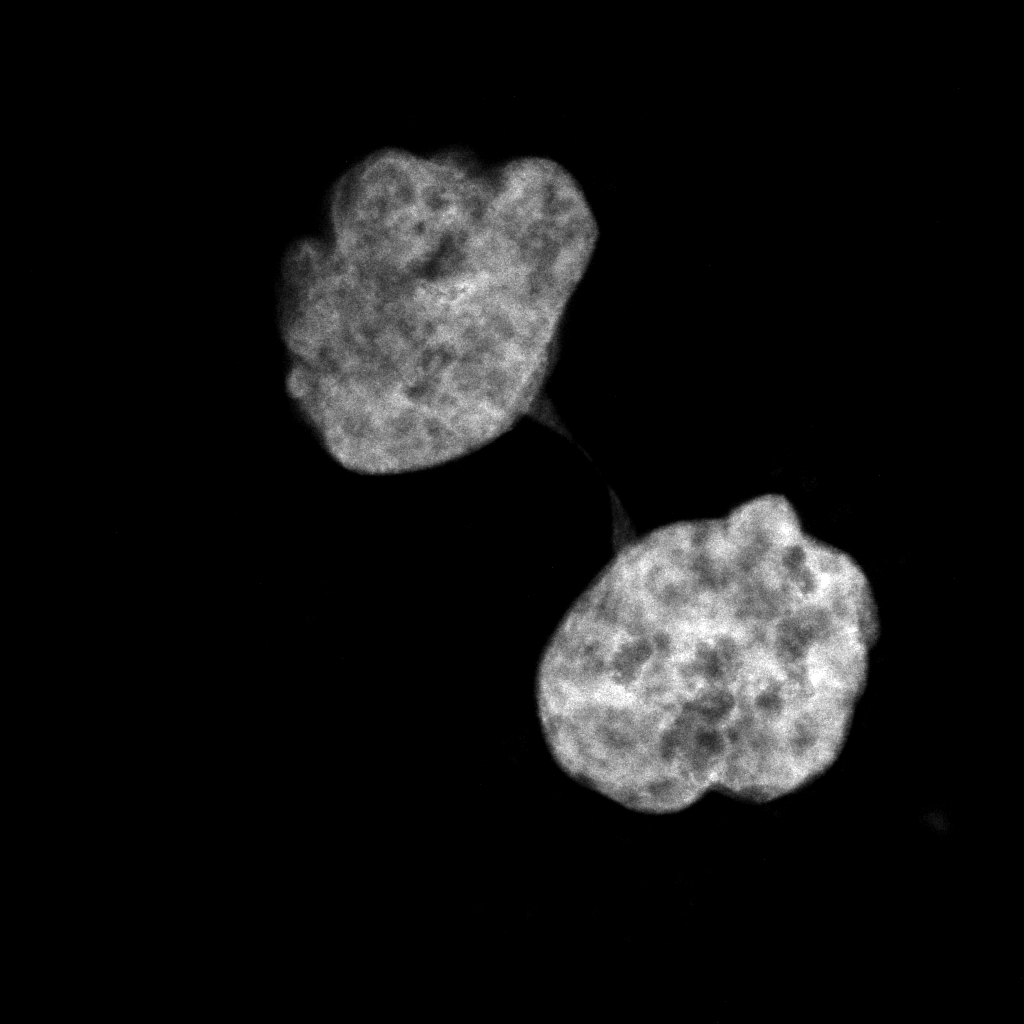

Supplement: Supplementary file 9 — Figure EV1-5 Source Data [file 44318_2025_672_MOESM9_ESM.zip › EV Source Data/EV1/EV1B/DKO1_DAPI.tif]

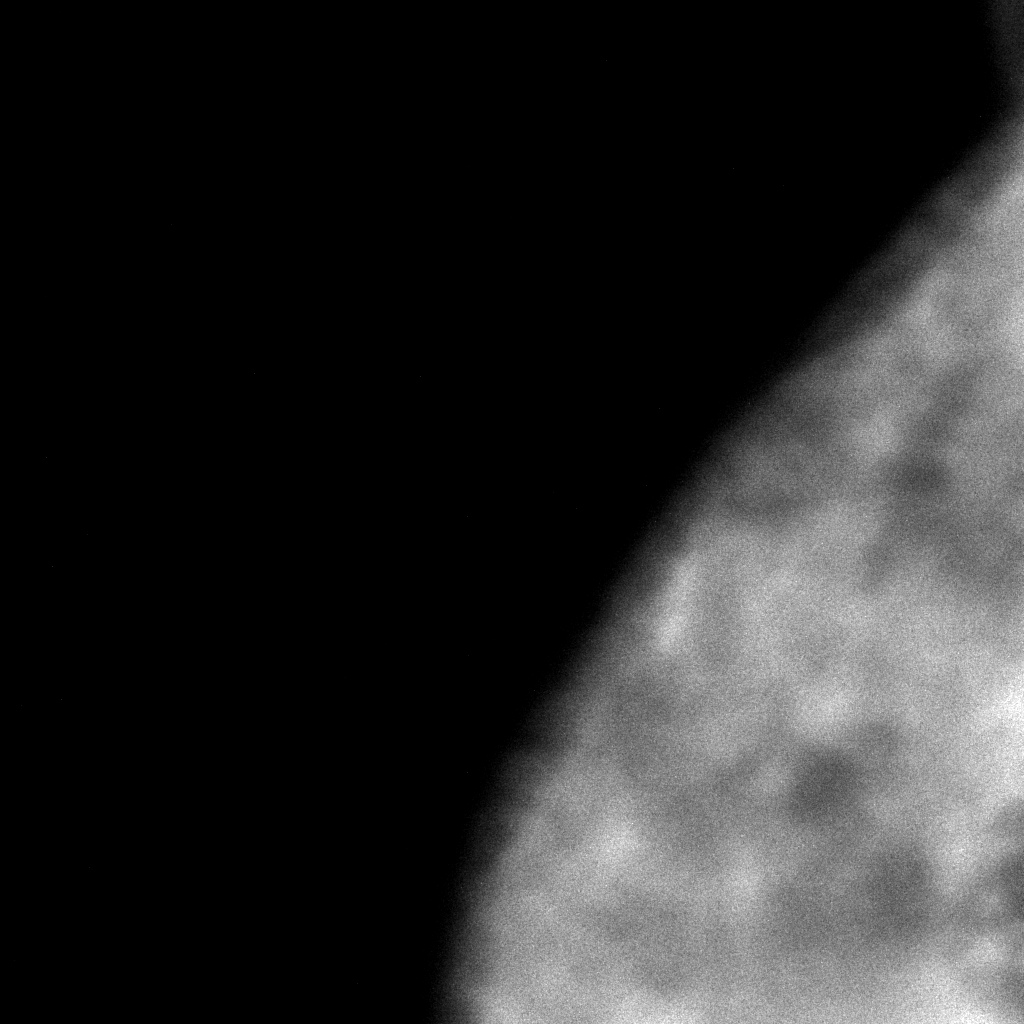

Supplement: Supplementary file 9 — Figure EV1-5 Source Data [file 44318_2025_672_MOESM9_ESM.zip › EV Source Data/EV1/EV1B/DKO1_DAPI_zoom.tif]

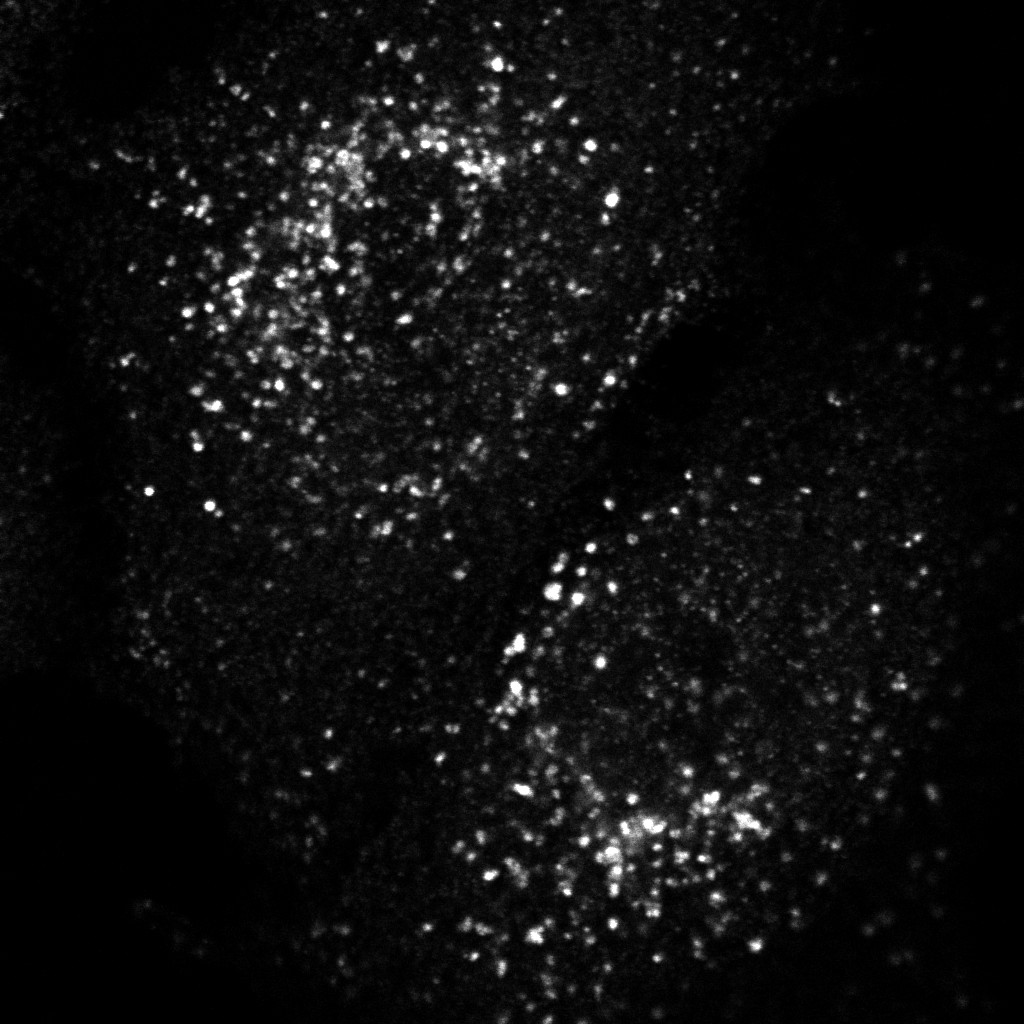

Supplement: Supplementary file 9 — Figure EV1-5 Source Data [file 44318_2025_672_MOESM9_ESM.zip › EV Source Data/EV1/EV1B/DKO1_Gal3.tif]

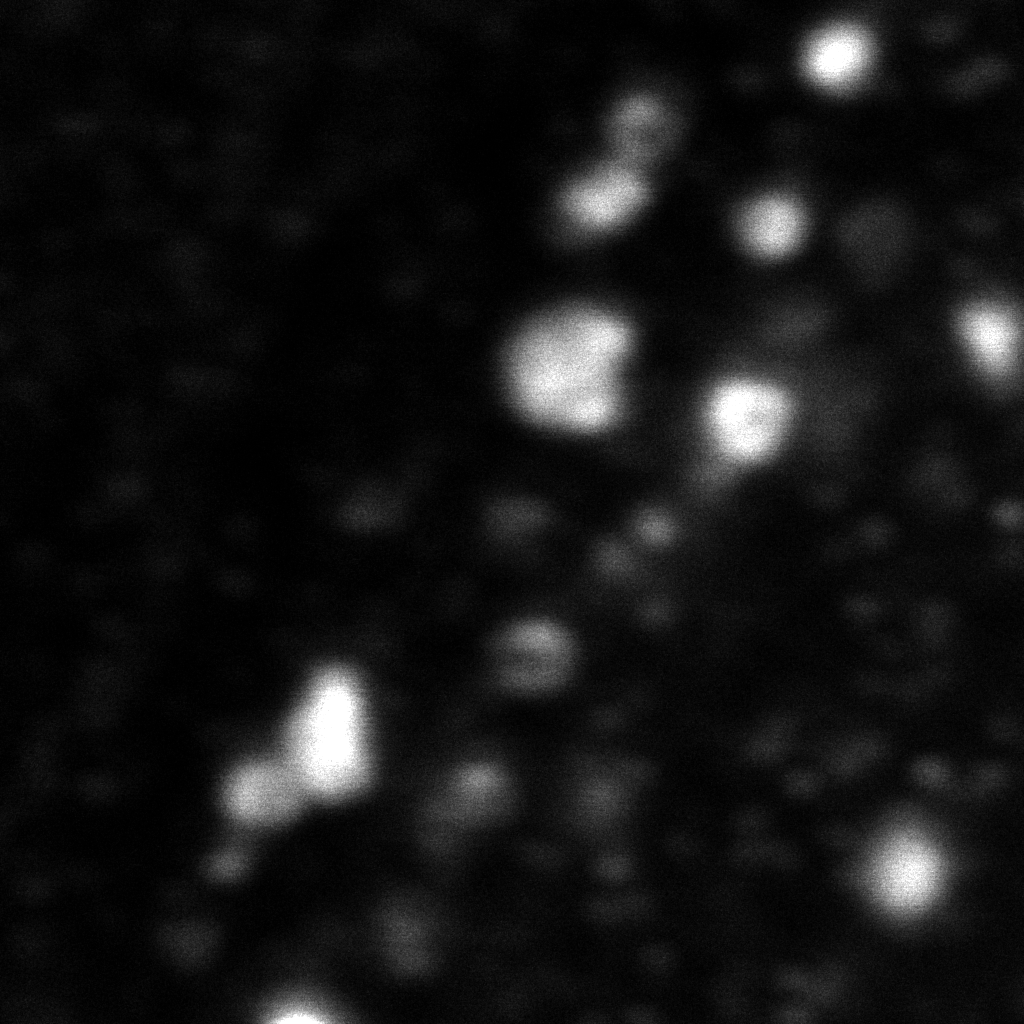

Supplement: Supplementary file 9 — Figure EV1-5 Source Data [file 44318_2025_672_MOESM9_ESM.zip › EV Source Data/EV1/EV1B/DKO1_Gal3_zoom.tif]

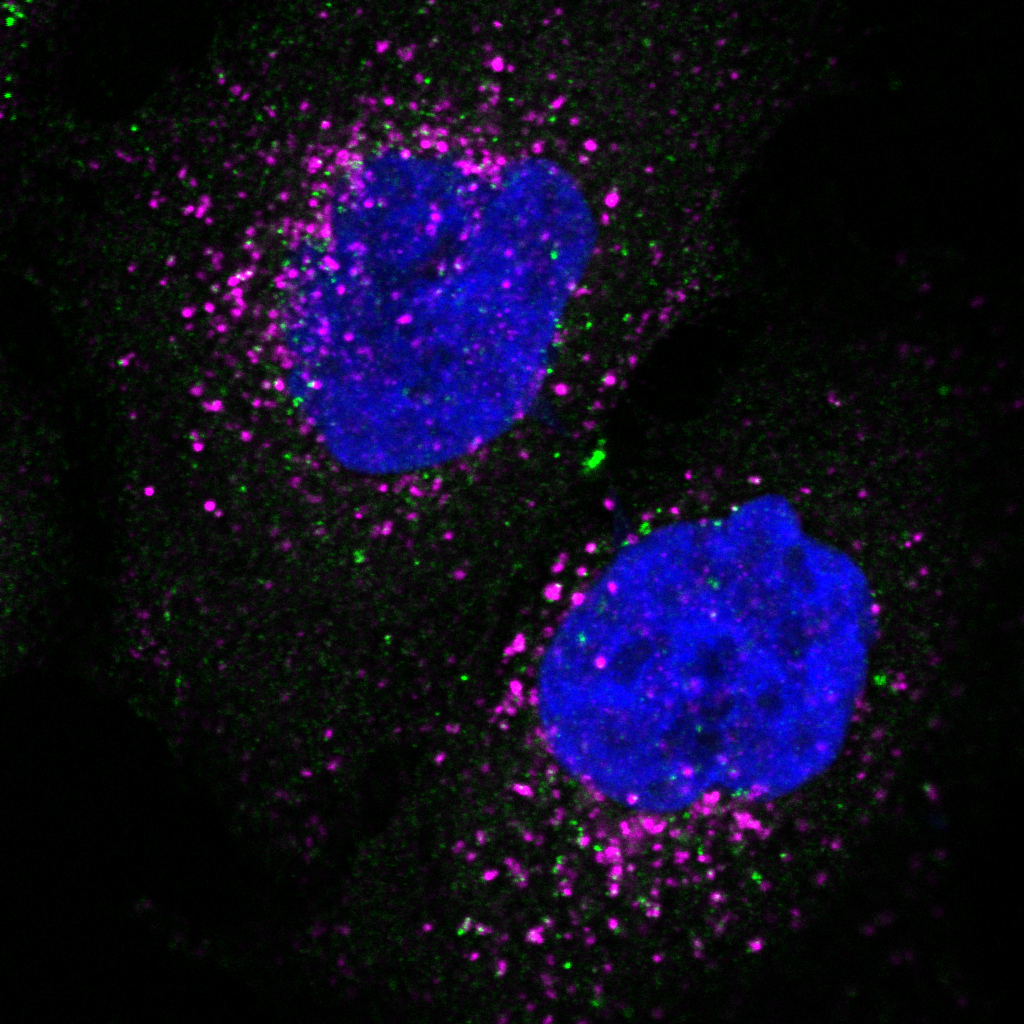

Supplement: Supplementary file 9 — Figure EV1-5 Source Data [file 44318_2025_672_MOESM9_ESM.zip › EV Source Data/EV1/EV1B/DKO1_merge.tif]

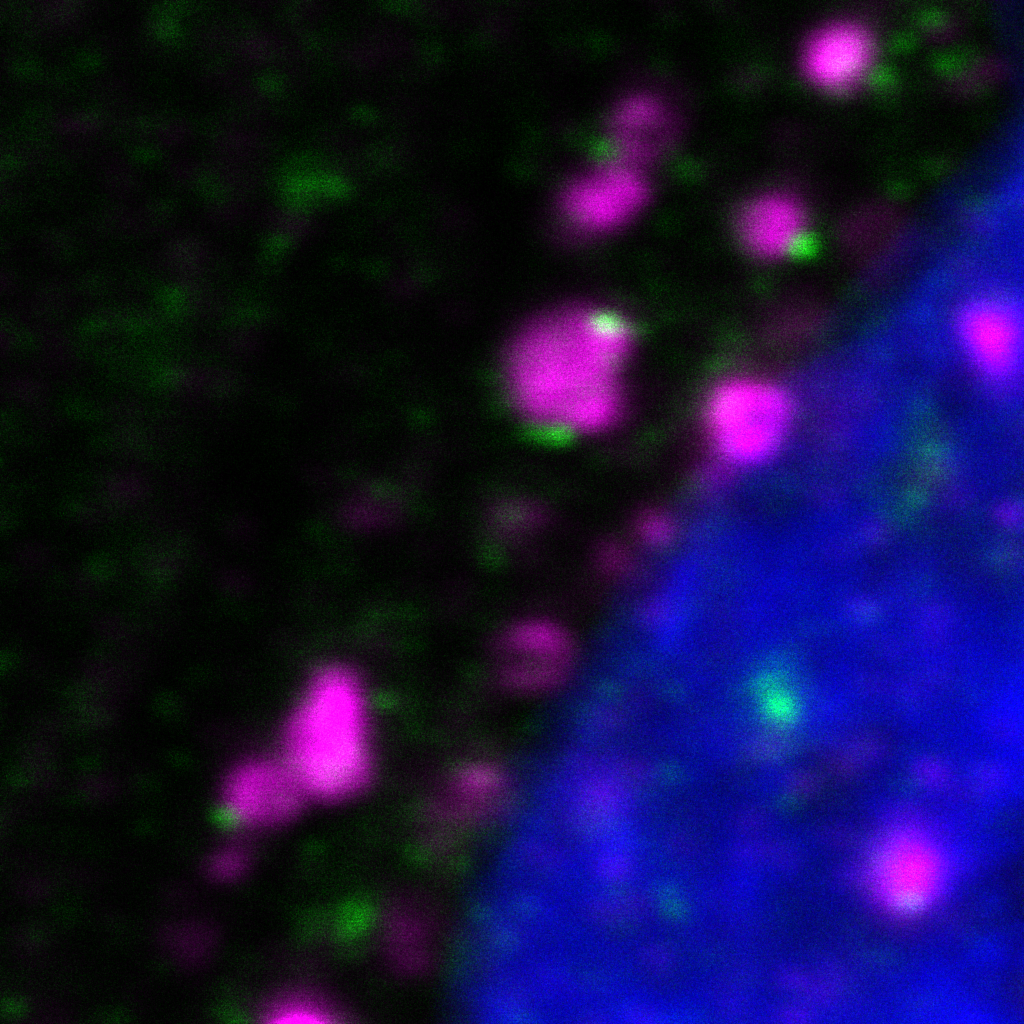

Supplement: Supplementary file 9 — Figure EV1-5 Source Data [file 44318_2025_672_MOESM9_ESM.zip › EV Source Data/EV1/EV1B/DKO1_merge_zoom.tif]

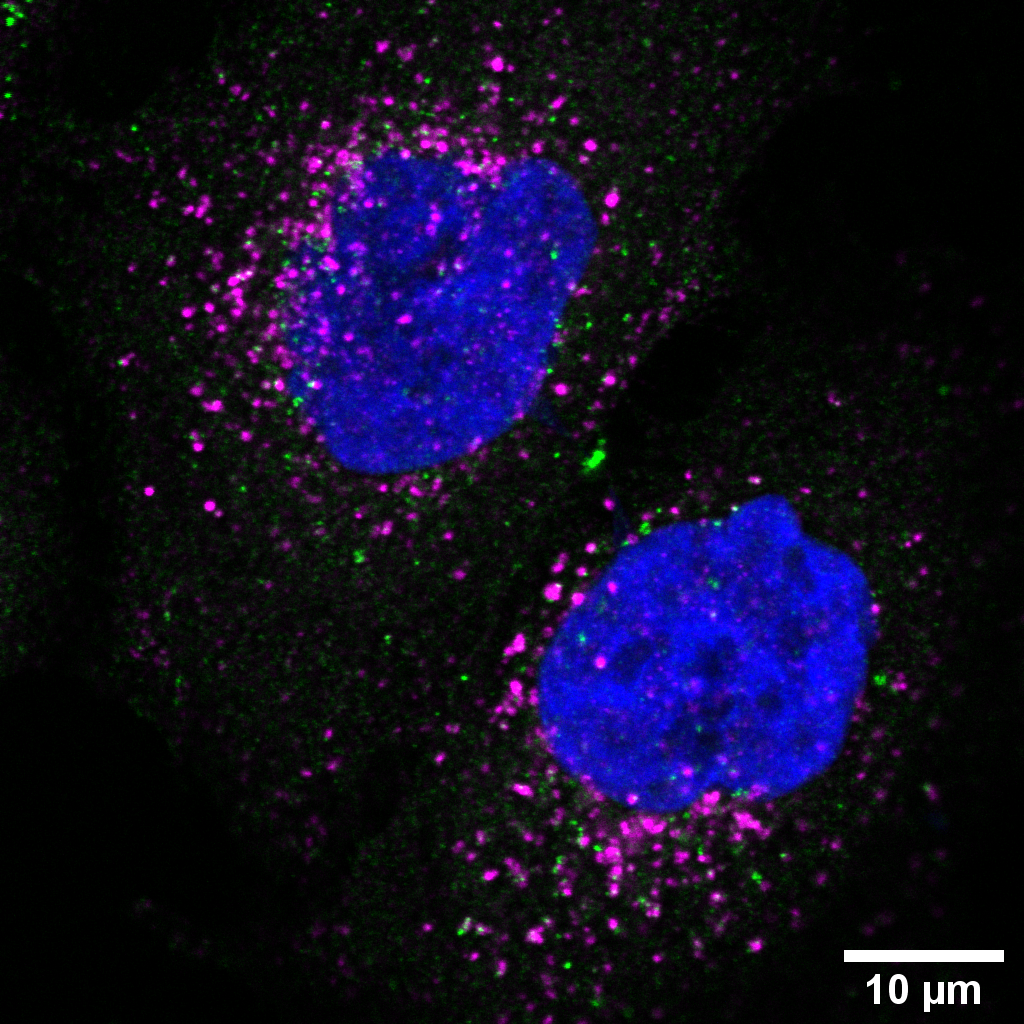

Supplement: Supplementary file 9 — Figure EV1-5 Source Data [file 44318_2025_672_MOESM9_ESM.zip › EV Source Data/EV1/EV1B/DKO1_scale.tif]

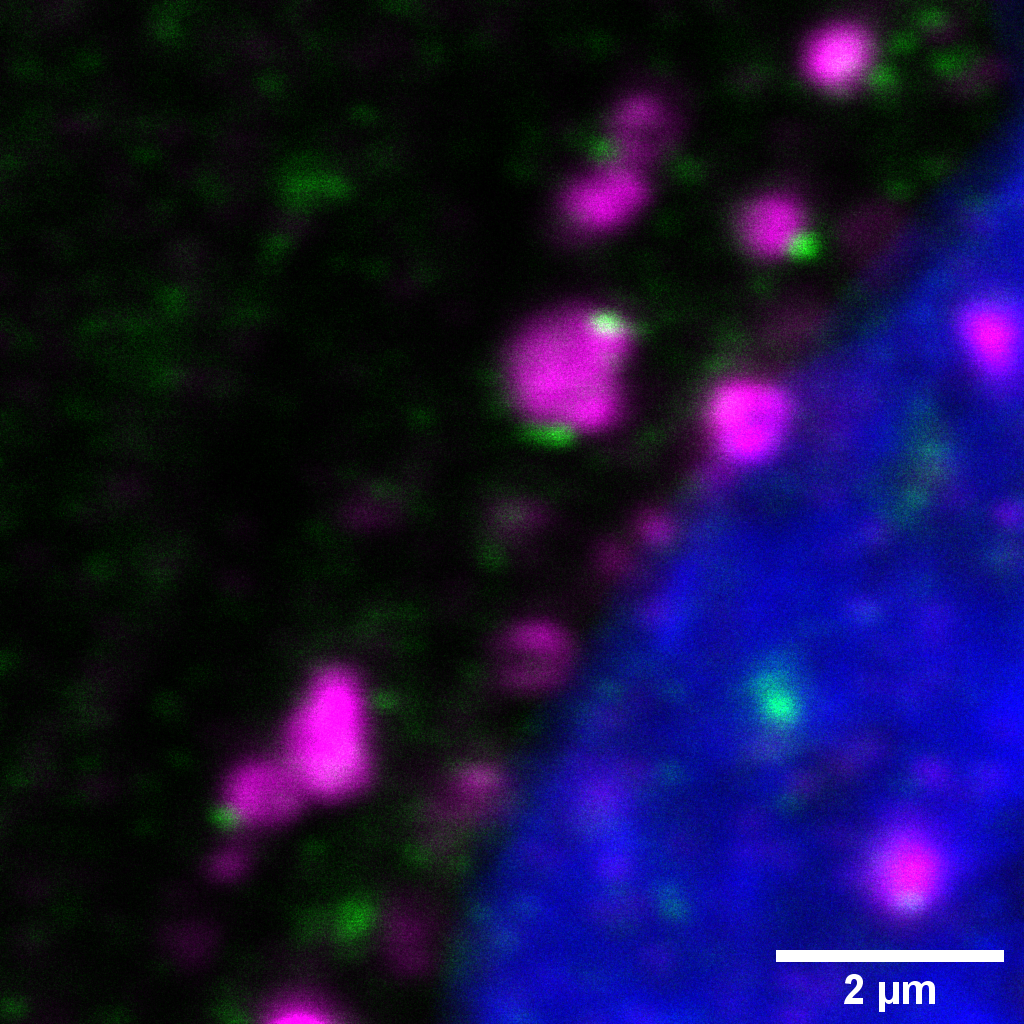

Supplement: Supplementary file 9 — Figure EV1-5 Source Data [file 44318_2025_672_MOESM9_ESM.zip › EV Source Data/EV1/EV1B/DKO1_scale_zoom.tif]

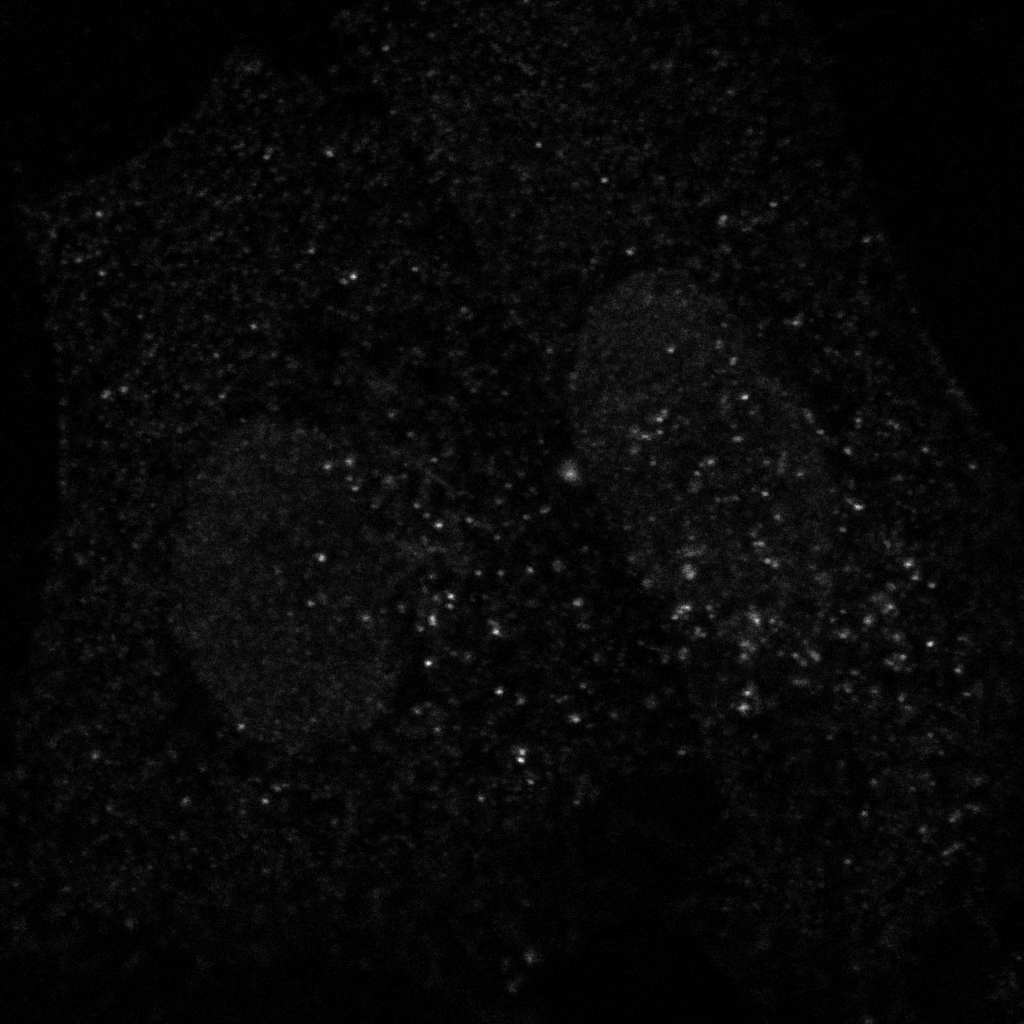

Supplement: Supplementary file 9 — Figure EV1-5 Source Data [file 44318_2025_672_MOESM9_ESM.zip › EV Source Data/EV1/EV1B/DKO2_ALIX.tif]

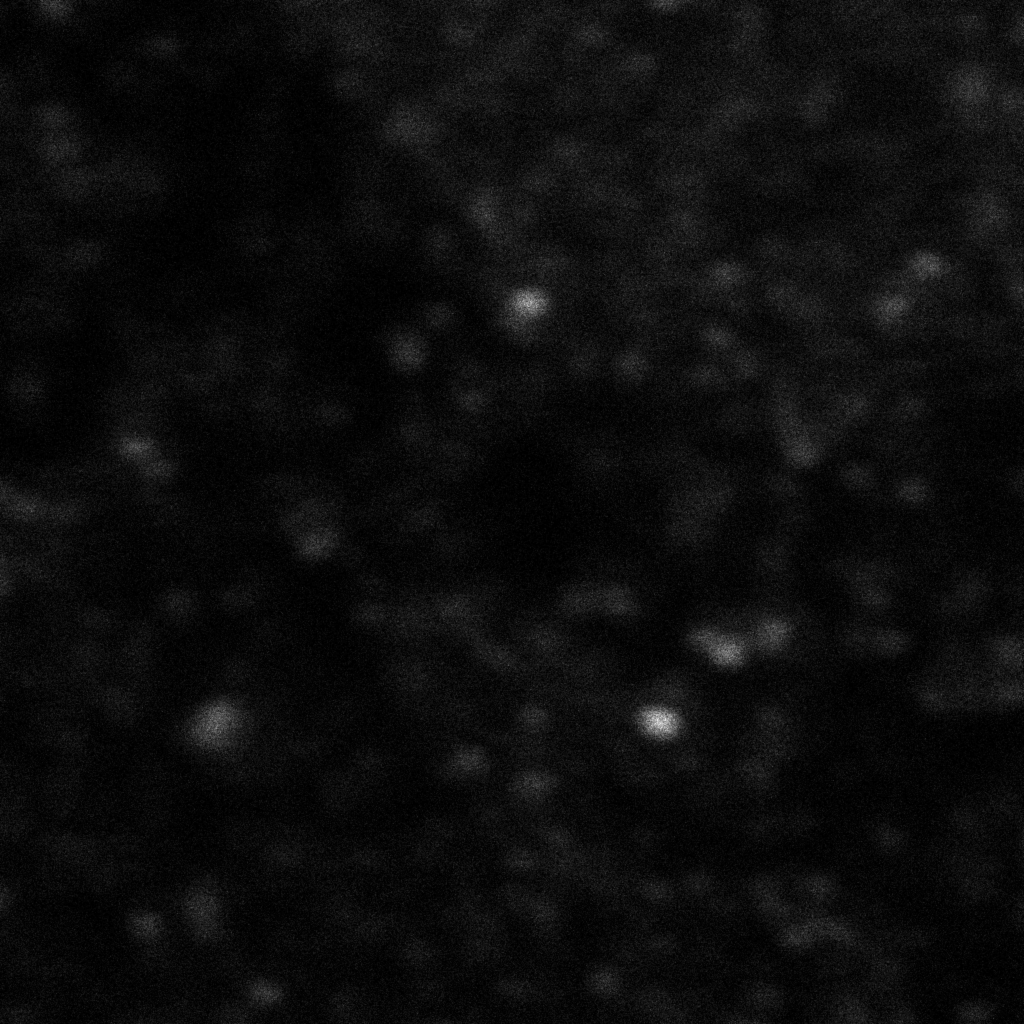

Supplement: Supplementary file 9 — Figure EV1-5 Source Data [file 44318_2025_672_MOESM9_ESM.zip › EV Source Data/EV1/EV1B/DKO2_ALIX_zoom.tif]

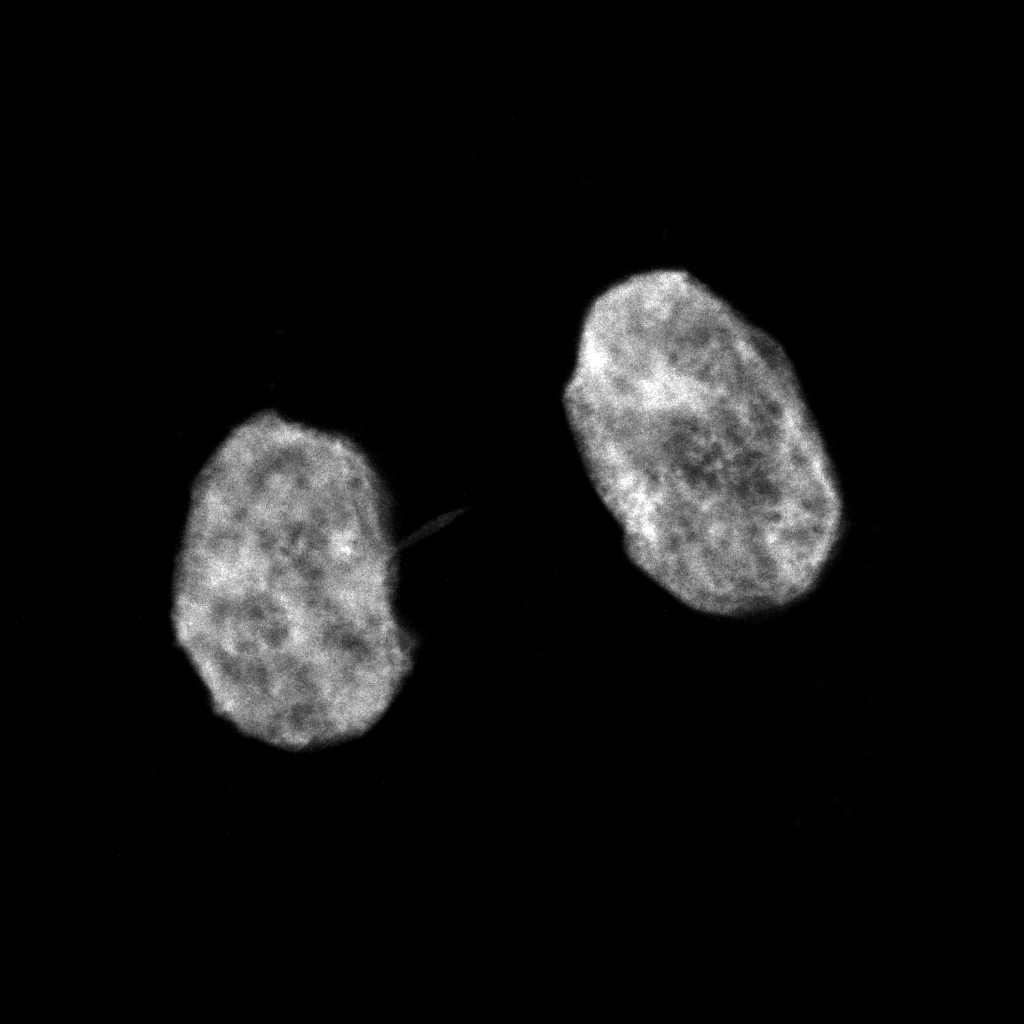

Supplement: Supplementary file 9 — Figure EV1-5 Source Data [file 44318_2025_672_MOESM9_ESM.zip › EV Source Data/EV1/EV1B/DKO2_DAPI.tif]

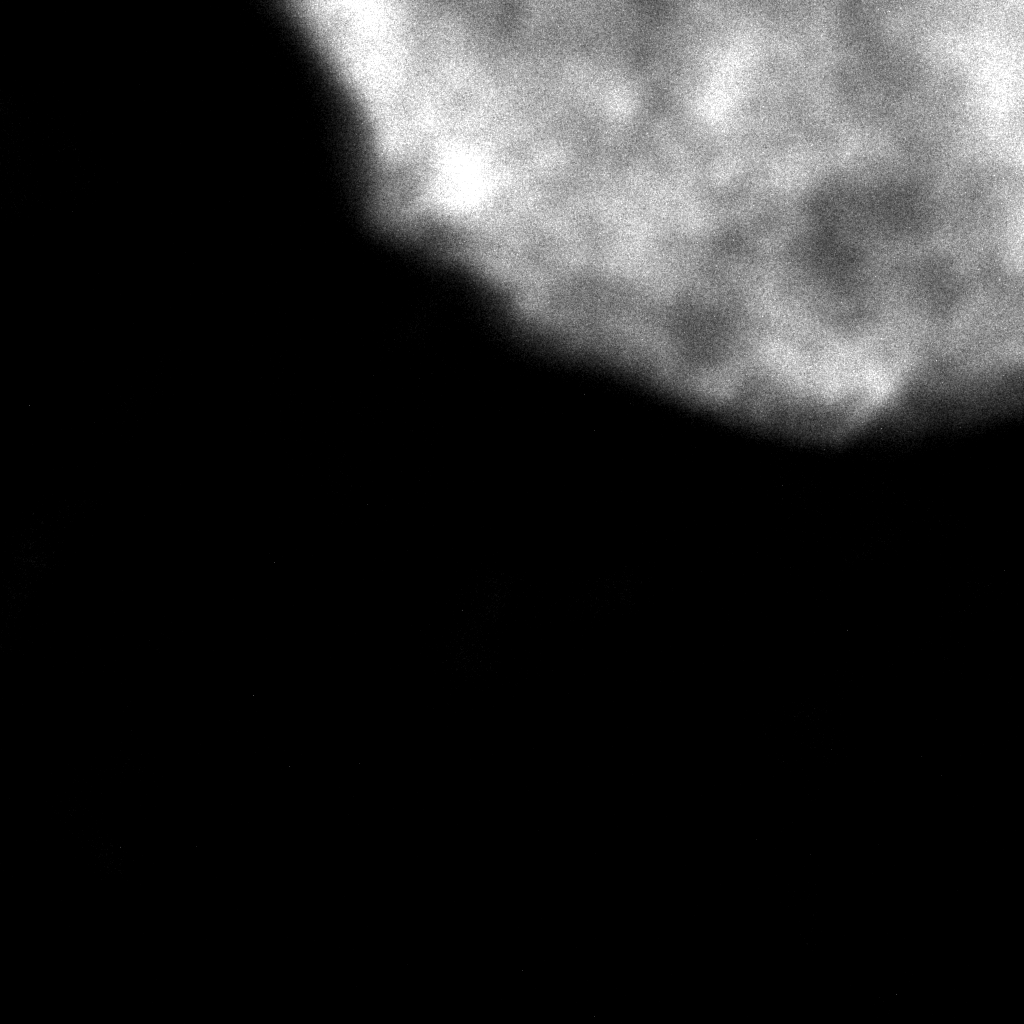

Supplement: Supplementary file 9 — Figure EV1-5 Source Data [file 44318_2025_672_MOESM9_ESM.zip › EV Source Data/EV1/EV1B/DKO2_DAPI_zoom.tif]

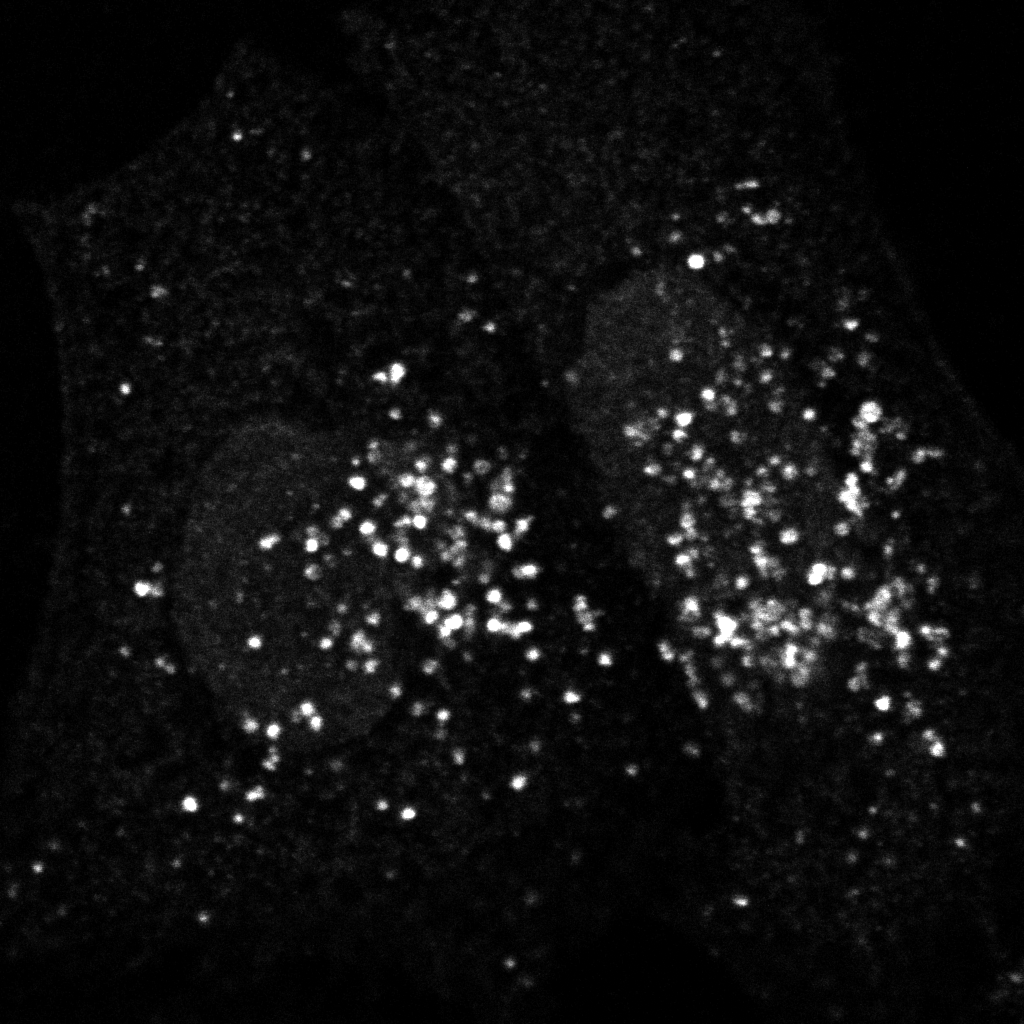

Supplement: Supplementary file 9 — Figure EV1-5 Source Data [file 44318_2025_672_MOESM9_ESM.zip › EV Source Data/EV1/EV1B/DKO2_Gal3.tif]

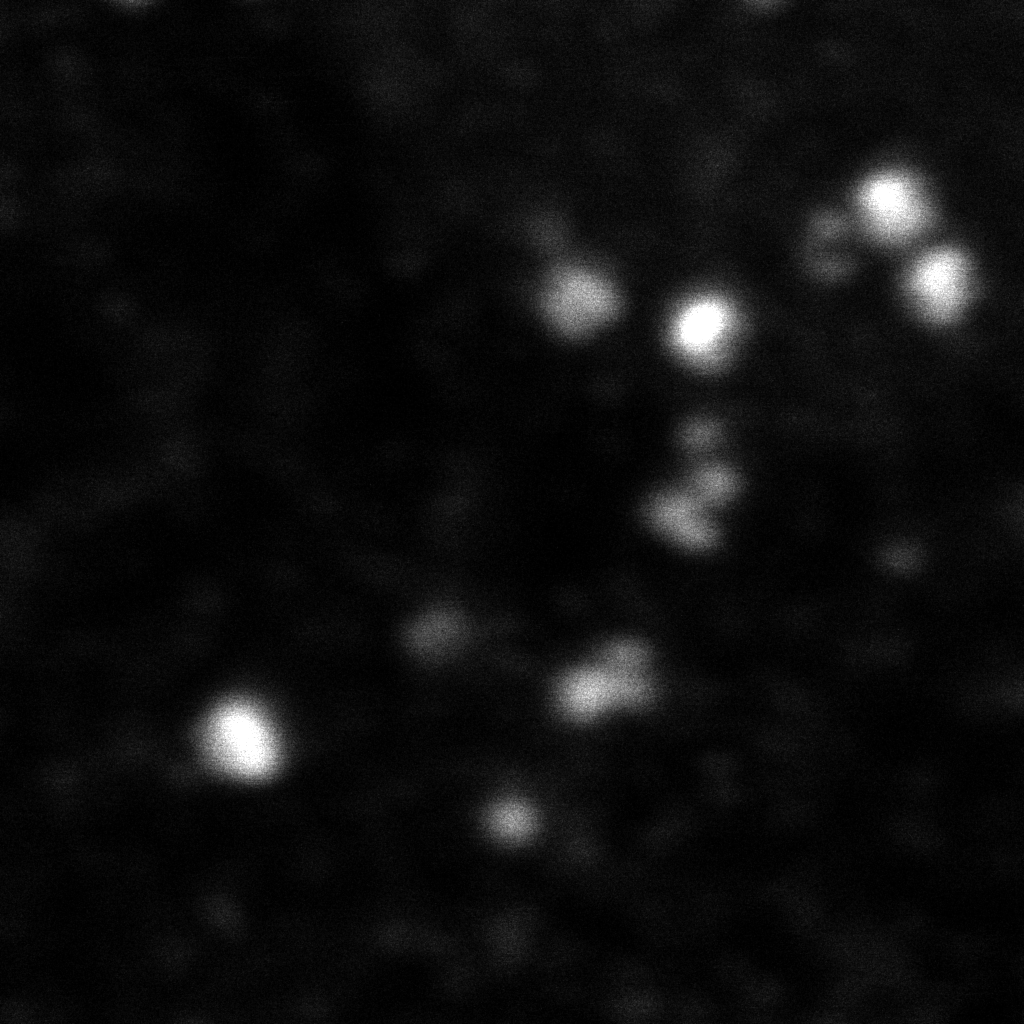

Supplement: Supplementary file 9 — Figure EV1-5 Source Data [file 44318_2025_672_MOESM9_ESM.zip › EV Source Data/EV1/EV1B/DKO2_Gal3_zoom.tif]

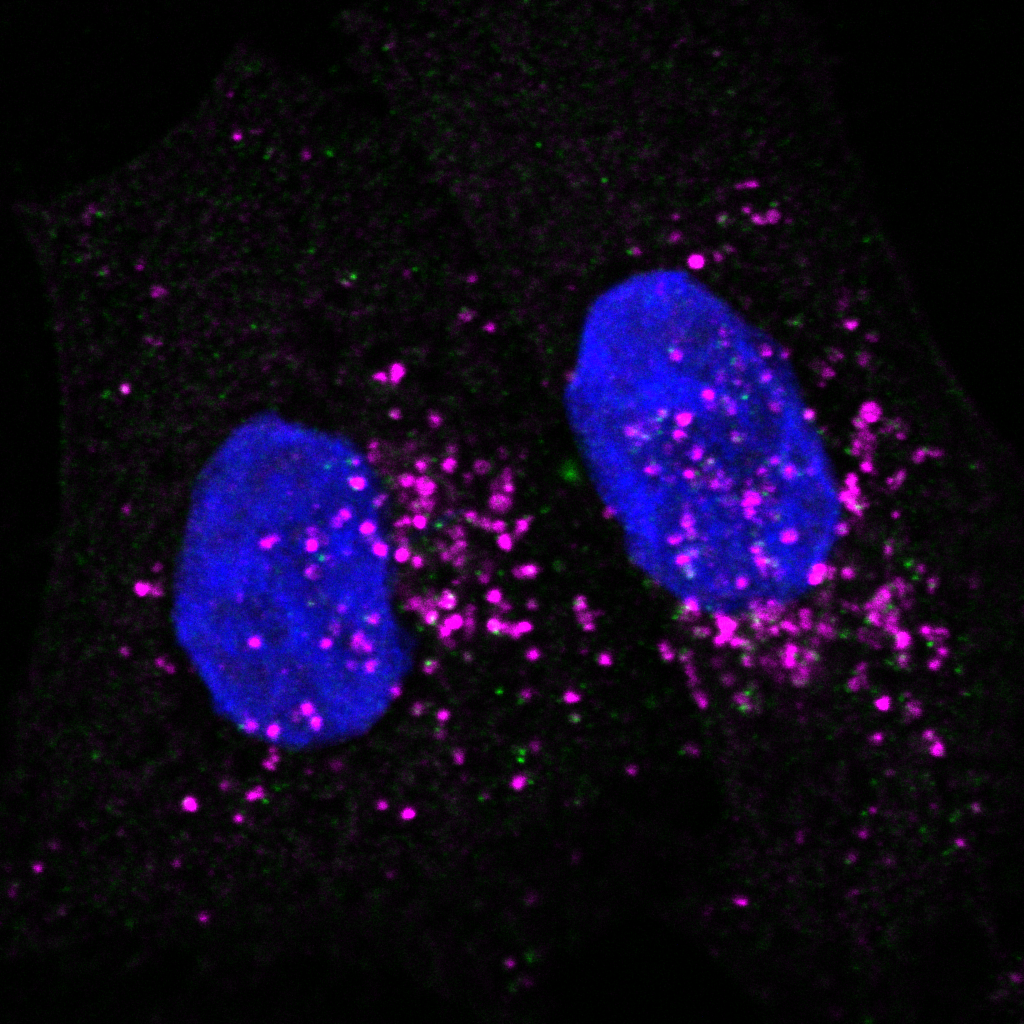

Supplement: Supplementary file 9 — Figure EV1-5 Source Data [file 44318_2025_672_MOESM9_ESM.zip › EV Source Data/EV1/EV1B/DKO2_merge.tif]

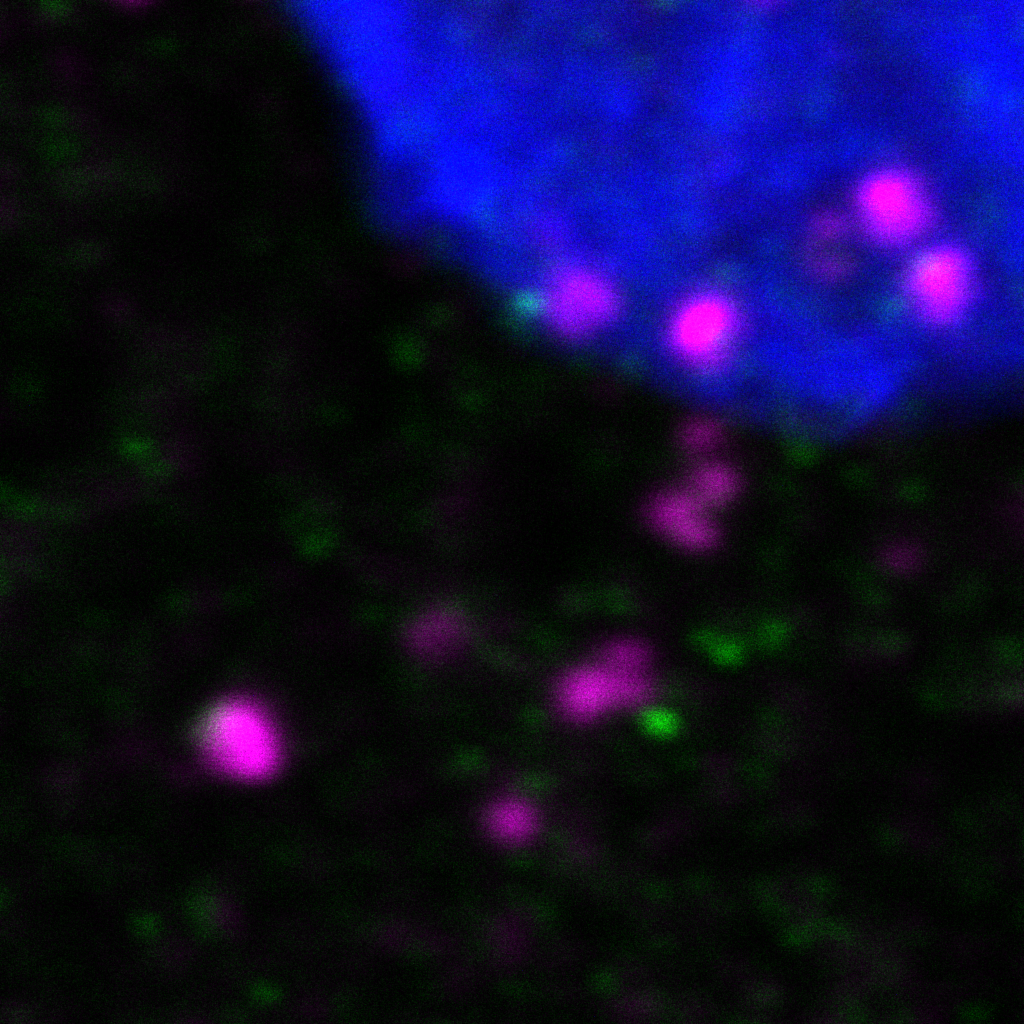

Supplement: Supplementary file 9 — Figure EV1-5 Source Data [file 44318_2025_672_MOESM9_ESM.zip › EV Source Data/EV1/EV1B/DKO2_merge_zoom.tif]

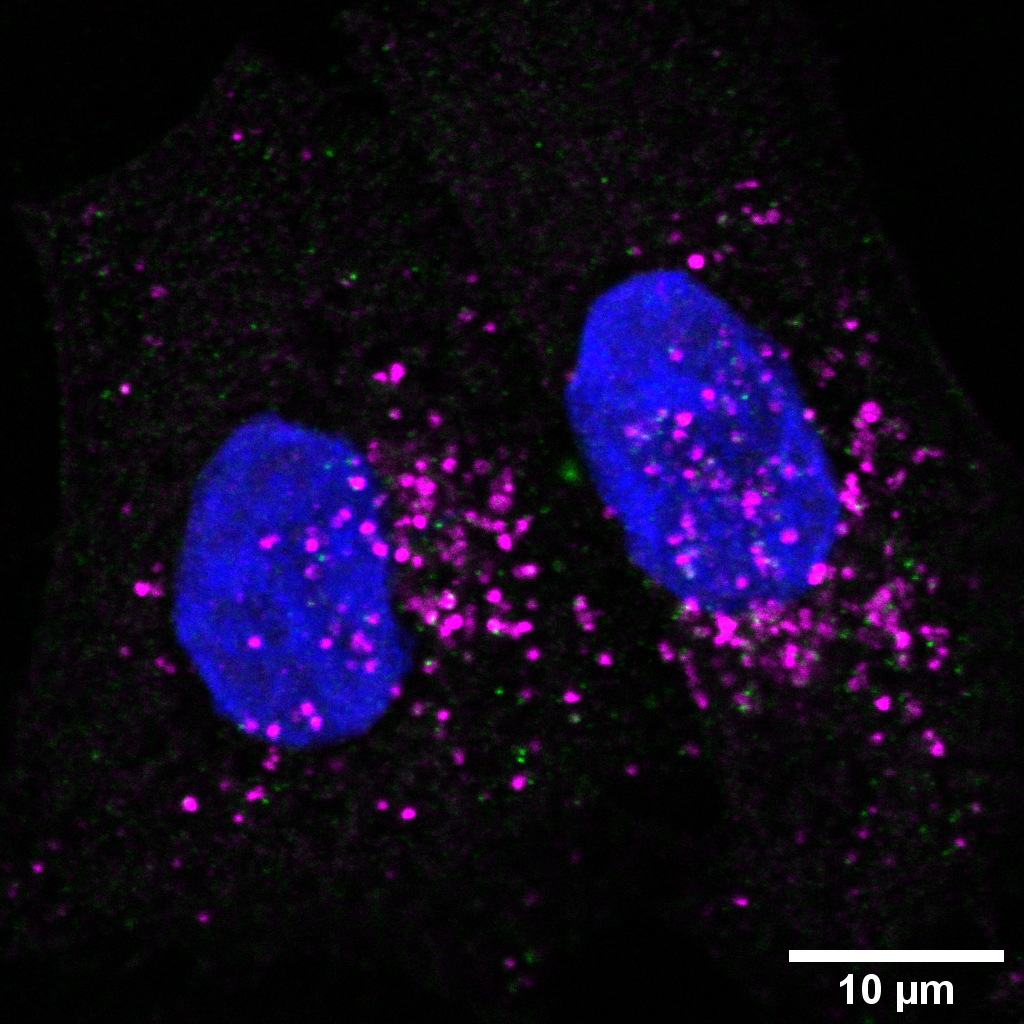

Supplement: Supplementary file 9 — Figure EV1-5 Source Data [file 44318_2025_672_MOESM9_ESM.zip › EV Source Data/EV1/EV1B/DKO2_scale.tif]

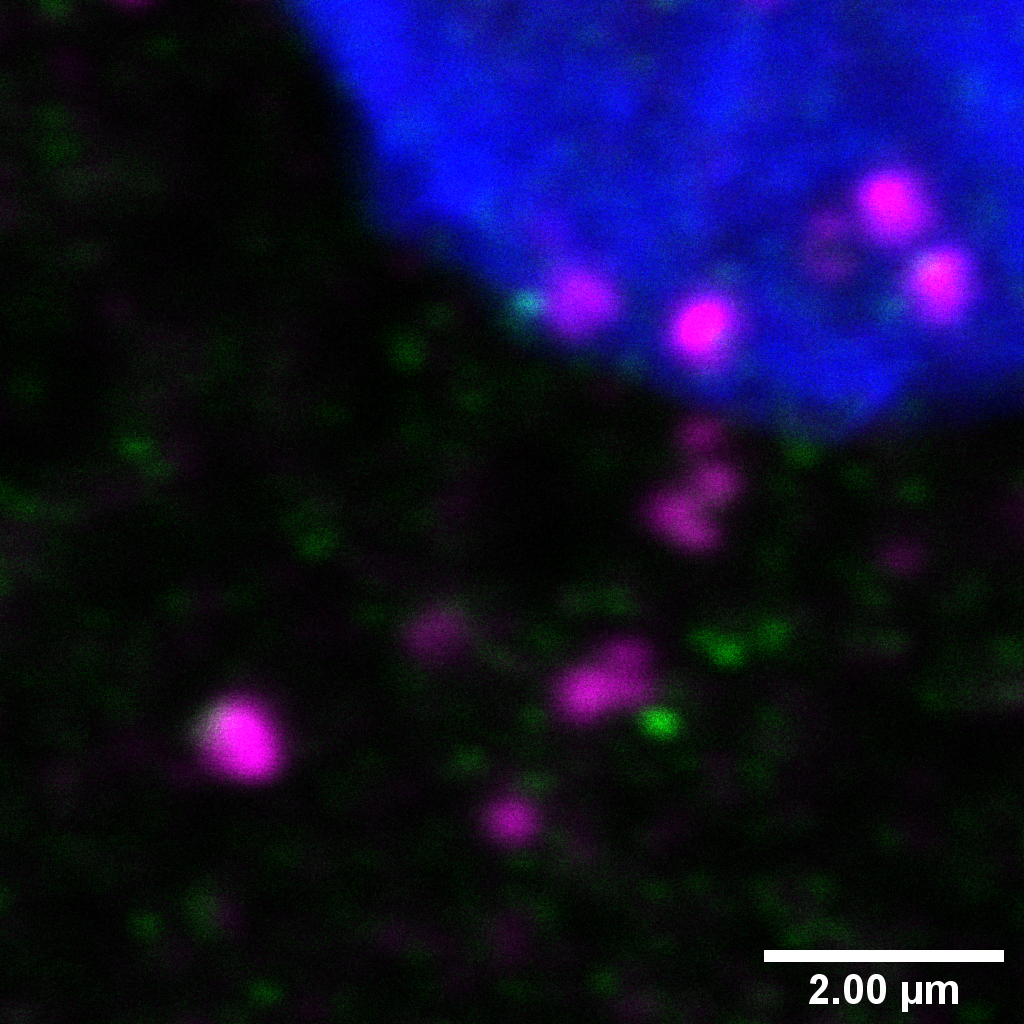

Supplement: Supplementary file 9 — Figure EV1-5 Source Data [file 44318_2025_672_MOESM9_ESM.zip › EV Source Data/EV1/EV1B/DKO2_scale_zoom.tif]

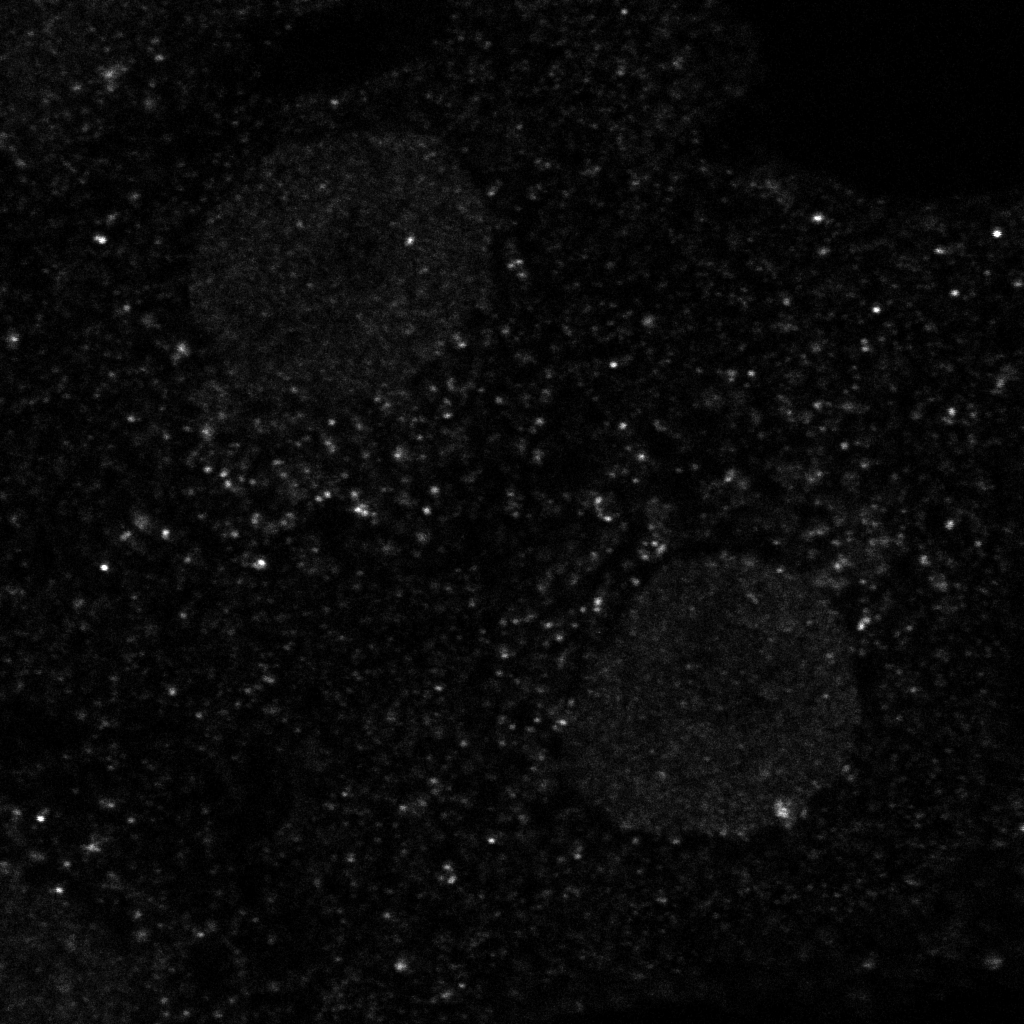

Supplement: Supplementary file 9 — Figure EV1-5 Source Data [file 44318_2025_672_MOESM9_ESM.zip › EV Source Data/EV1/EV1B/DKO3_ALIX.tif]

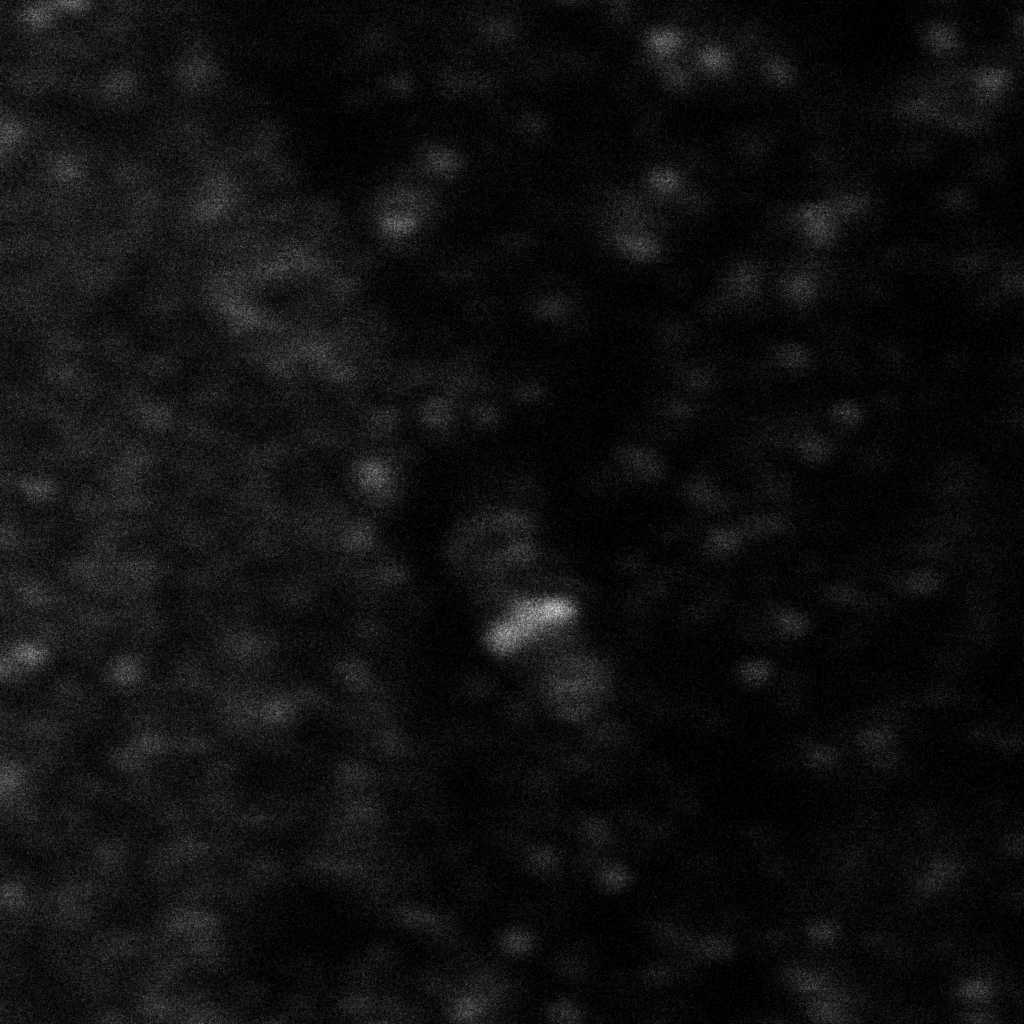

Supplement: Supplementary file 9 — Figure EV1-5 Source Data [file 44318_2025_672_MOESM9_ESM.zip › EV Source Data/EV1/EV1B/DKO3_ALIX_zoom.tif]

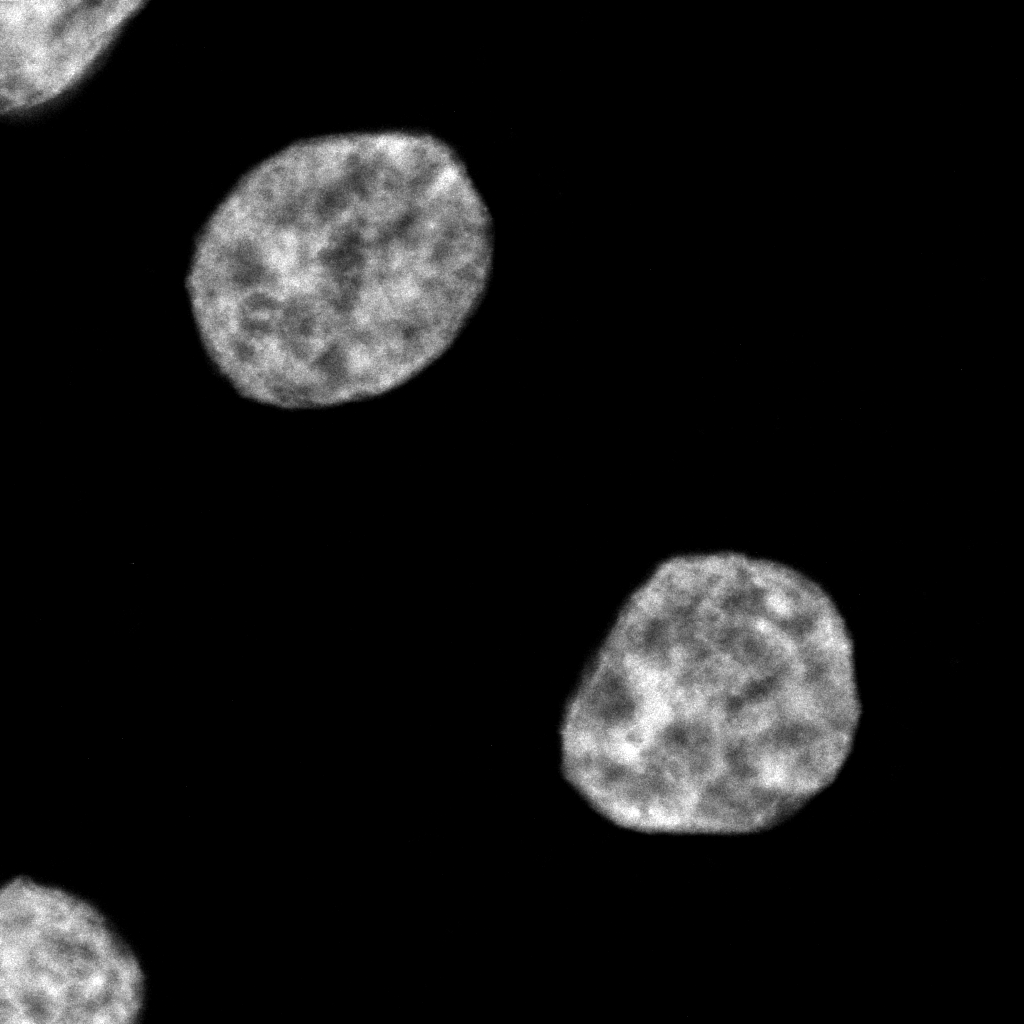

Supplement: Supplementary file 9 — Figure EV1-5 Source Data [file 44318_2025_672_MOESM9_ESM.zip › EV Source Data/EV1/EV1B/DKO3_DAPI.tif]

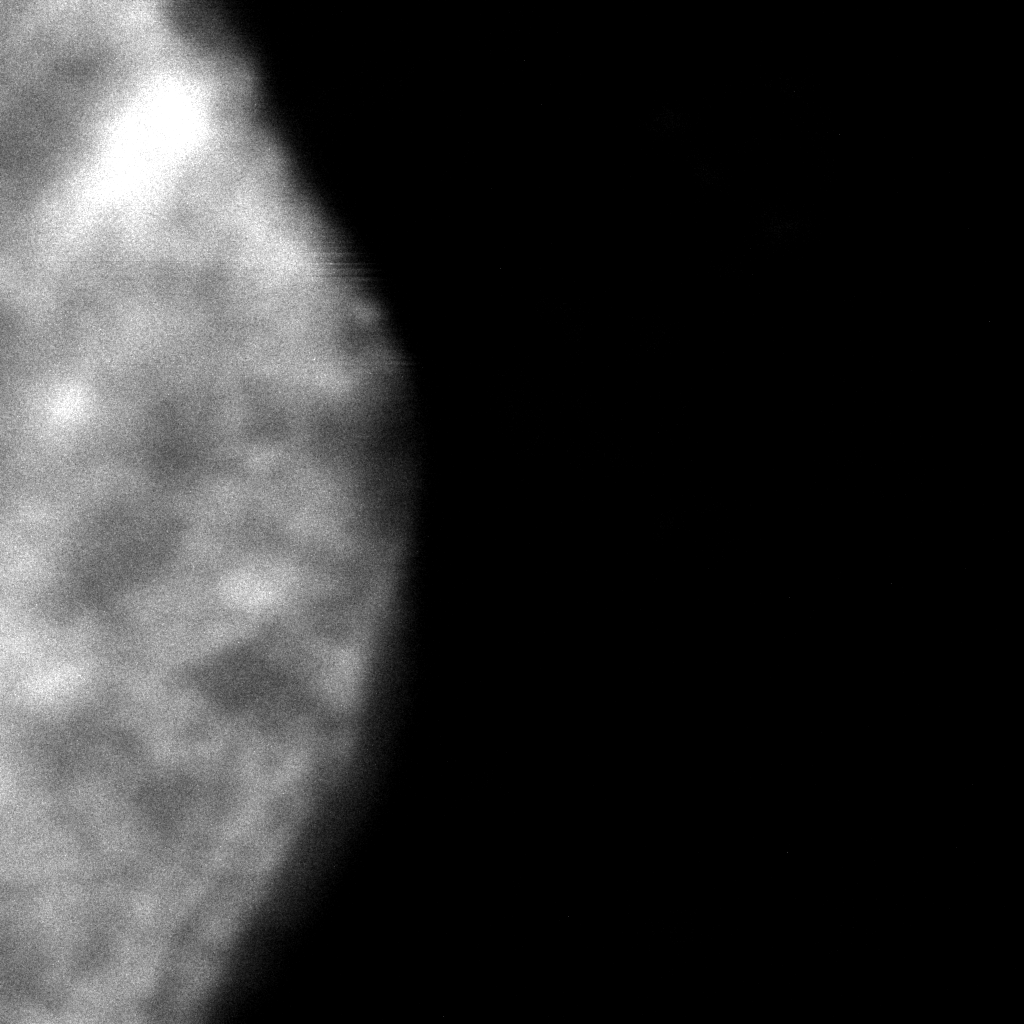

Supplement: Supplementary file 9 — Figure EV1-5 Source Data [file 44318_2025_672_MOESM9_ESM.zip › EV Source Data/EV1/EV1B/DKO3_DAPI_zoom.tif]

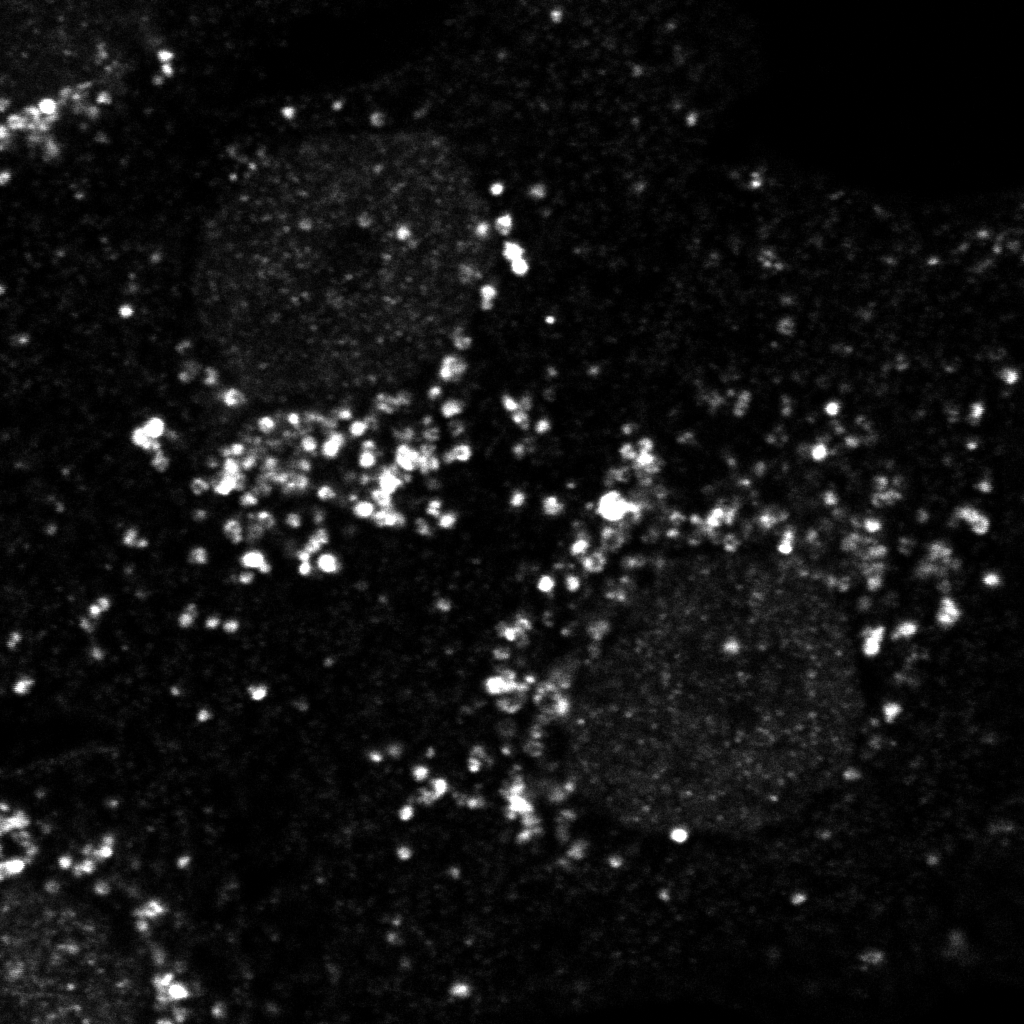

Supplement: Supplementary file 9 — Figure EV1-5 Source Data [file 44318_2025_672_MOESM9_ESM.zip › EV Source Data/EV1/EV1B/DKO3_Gal3.tif]

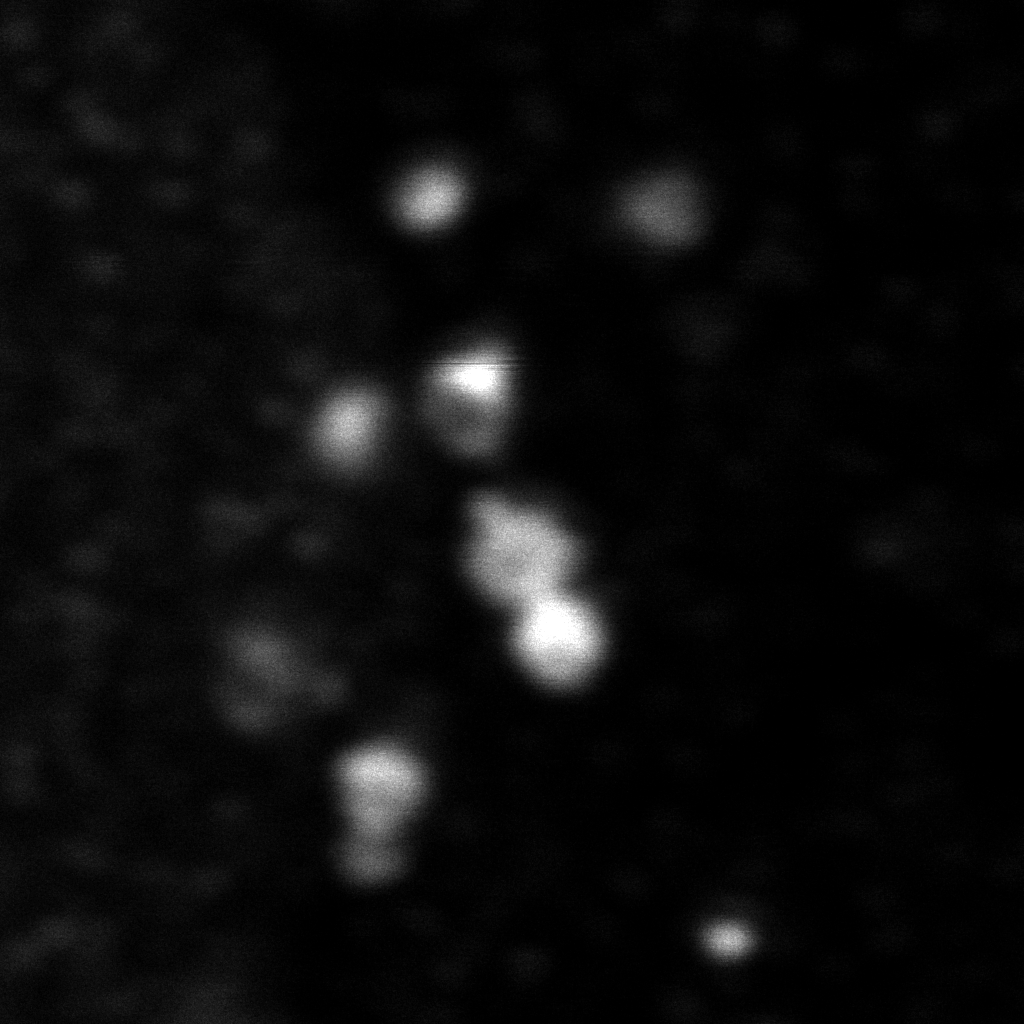

Supplement: Supplementary file 9 — Figure EV1-5 Source Data [file 44318_2025_672_MOESM9_ESM.zip › EV Source Data/EV1/EV1B/DKO3_Gal3_zoom.tif]

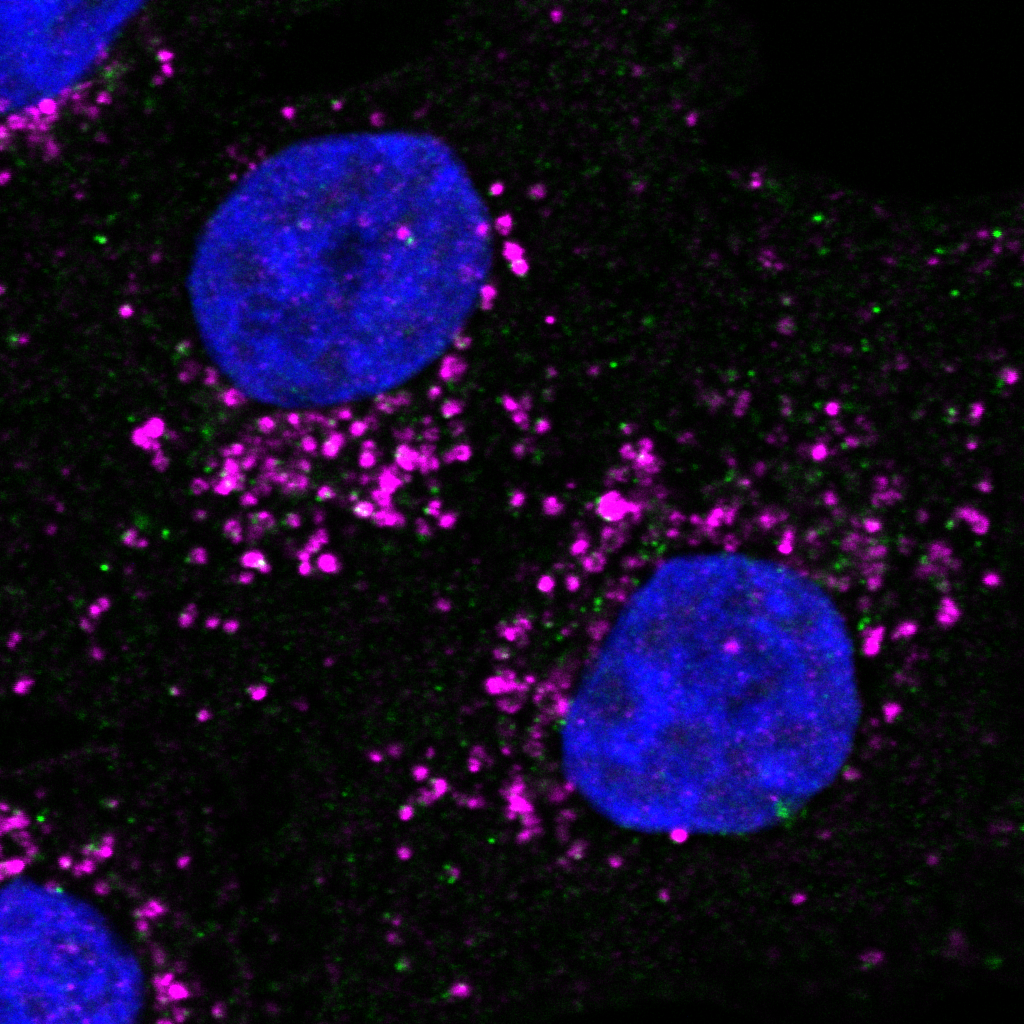

Supplement: Supplementary file 9 — Figure EV1-5 Source Data [file 44318_2025_672_MOESM9_ESM.zip › EV Source Data/EV1/EV1B/DKO3_merge.tif]

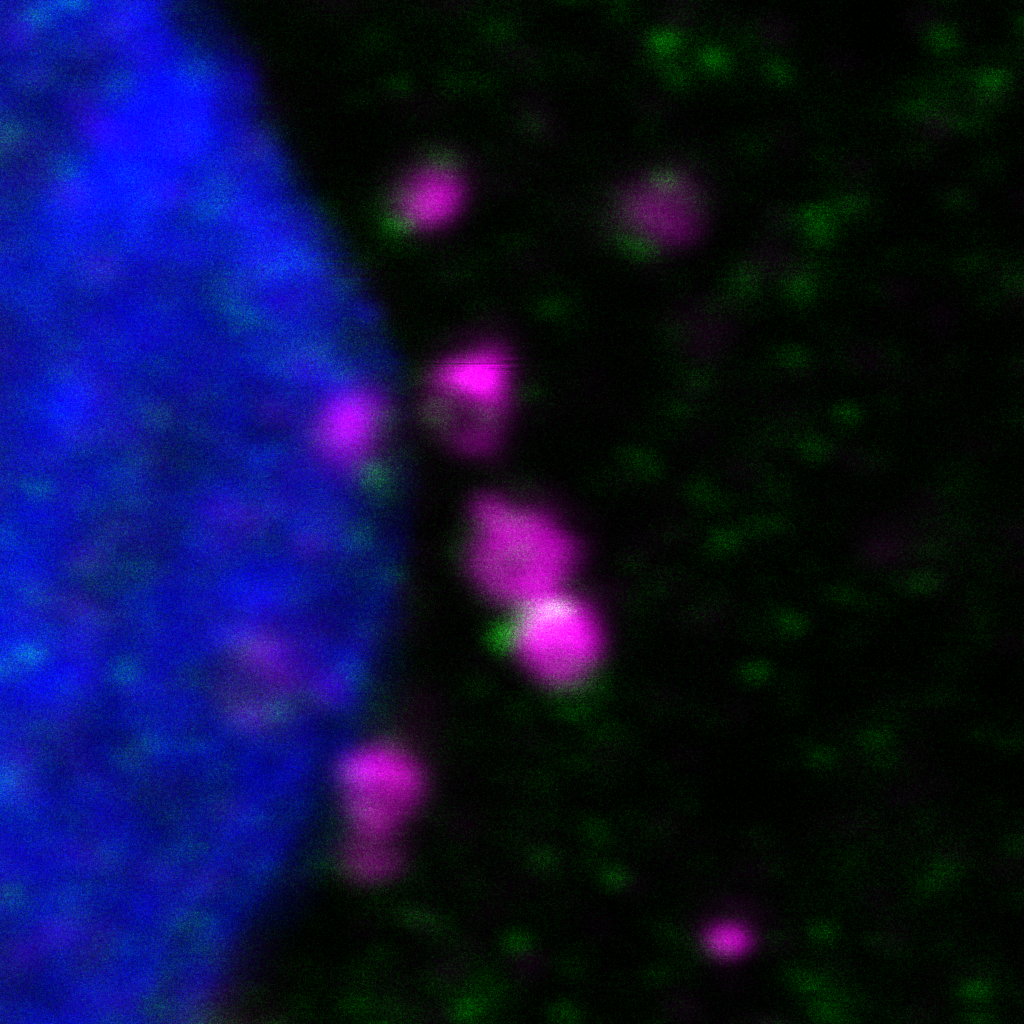

Supplement: Supplementary file 9 — Figure EV1-5 Source Data [file 44318_2025_672_MOESM9_ESM.zip › EV Source Data/EV1/EV1B/DKO3_merge_zoom.tif]
