# Supplementary figures and images for: The ATG8 E3-like ligases sense lysosomal damage and initiate ESCRT-mediated membrane repair (part 7 of 7)
Source: EMBO J. 2026 Jan 3;45(3):930–52. doi: 10.1038/s44318-025-00672-1 (PMC12865045; doi:10.1038/s44318-025-00672-1)

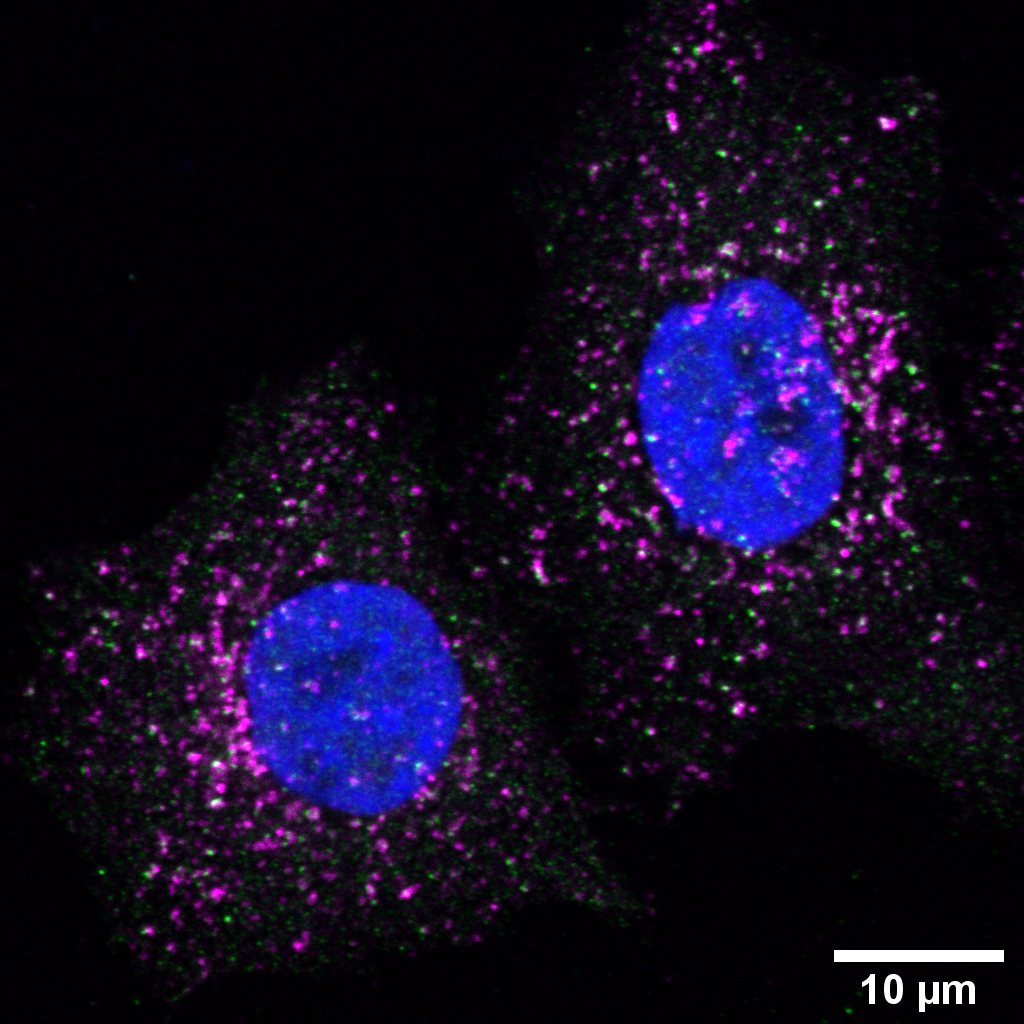

Supplement: Supplementary file 9 — Figure EV1-5 Source Data [file 44318_2025_672_MOESM9_ESM.zip › EV Source Data/EV4/EV4A/5KO_LLOMe_scale.tif]

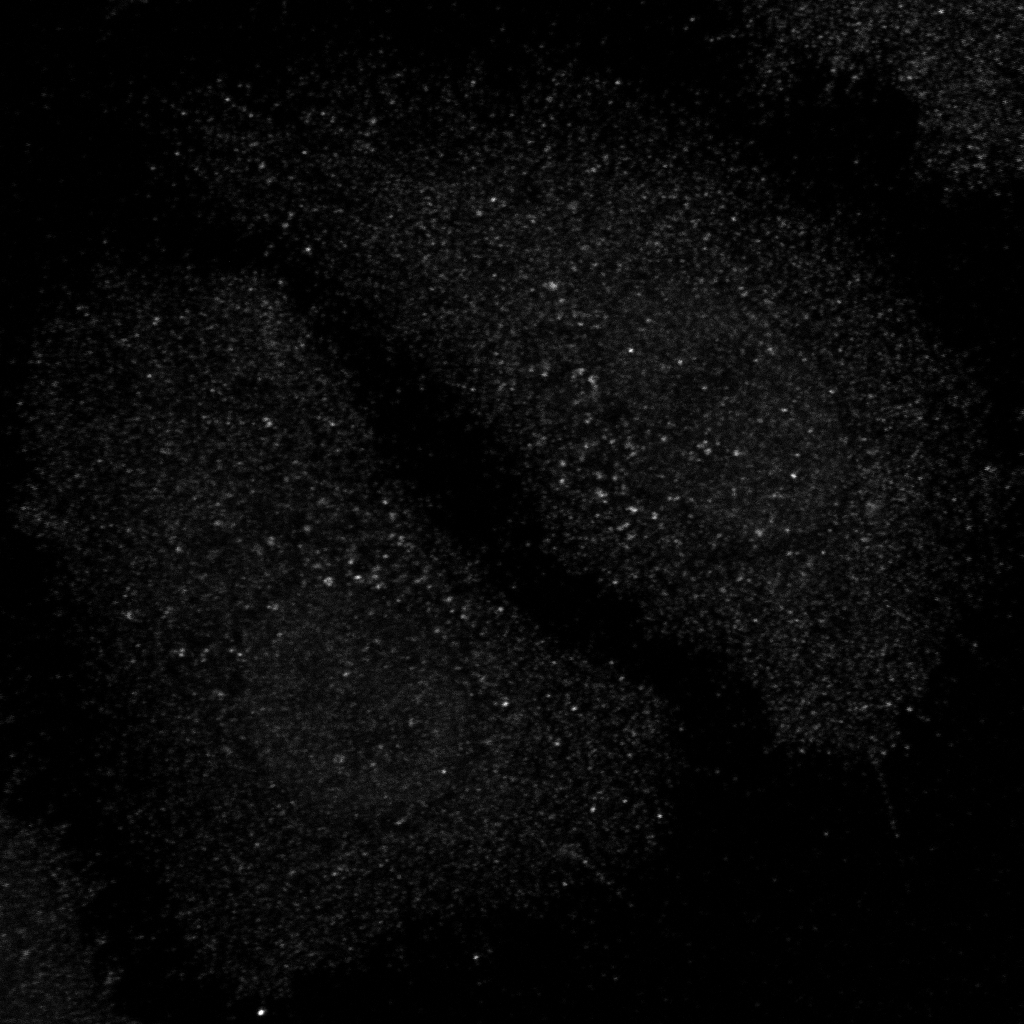

Supplement: Supplementary file 9 — Figure EV1-5 Source Data [file 44318_2025_672_MOESM9_ESM.zip › EV Source Data/EV4/EV4A/8KO_LLOMe_BAPTA_CHMP2A.tif]

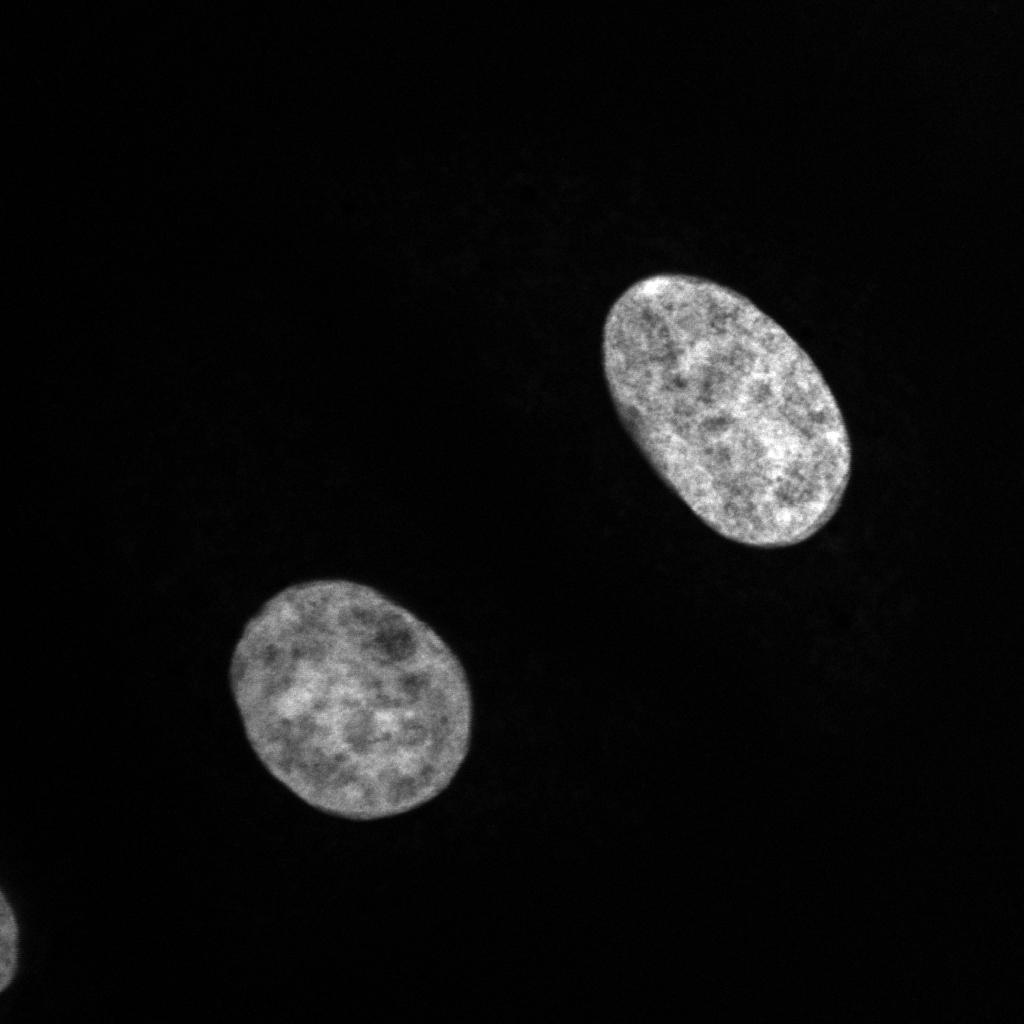

Supplement: Supplementary file 9 — Figure EV1-5 Source Data [file 44318_2025_672_MOESM9_ESM.zip › EV Source Data/EV4/EV4A/8KO_LLOMe_BAPTA_DAPI.tif]

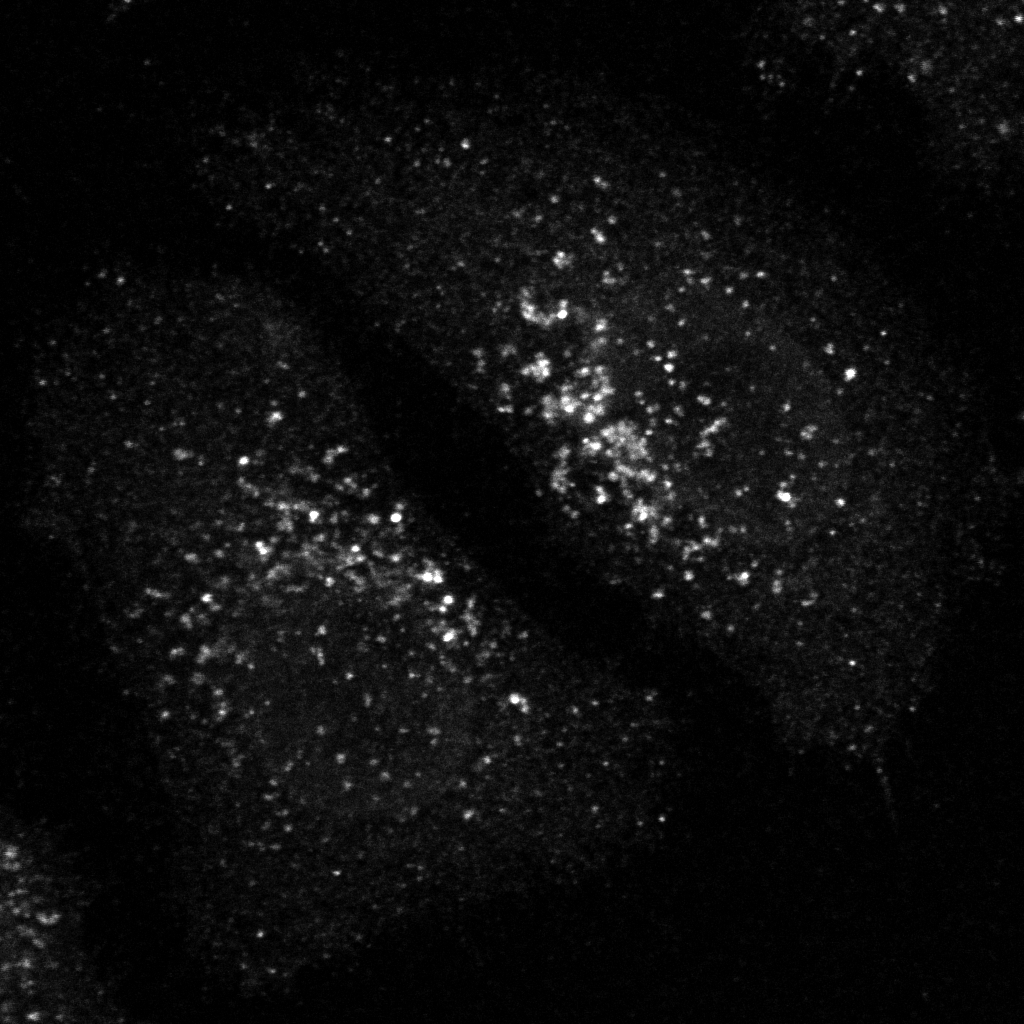

Supplement: Supplementary file 9 — Figure EV1-5 Source Data [file 44318_2025_672_MOESM9_ESM.zip › EV Source Data/EV4/EV4A/8KO_LLOMe_BAPTA_GAL3.tif]

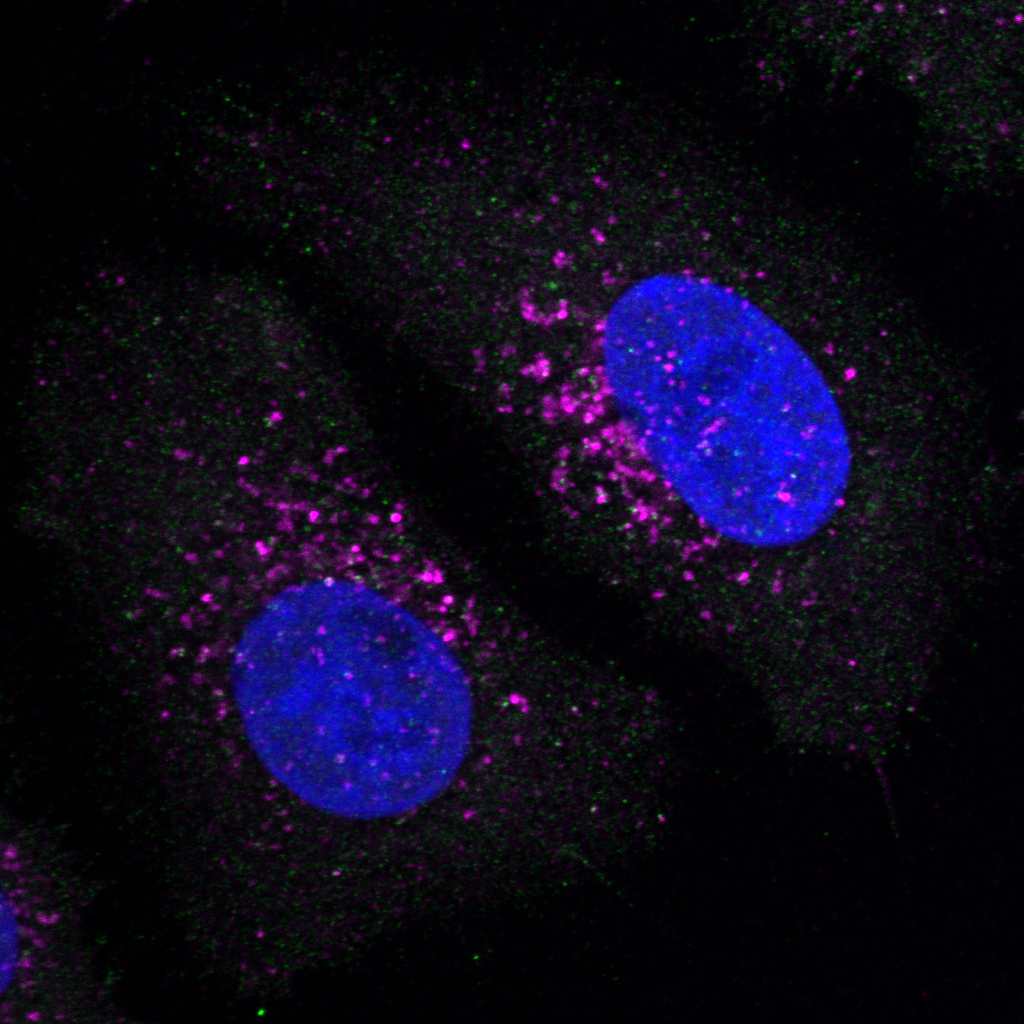

Supplement: Supplementary file 9 — Figure EV1-5 Source Data [file 44318_2025_672_MOESM9_ESM.zip › EV Source Data/EV4/EV4A/8KO_LLOMe_BAPTA_merge.tif]

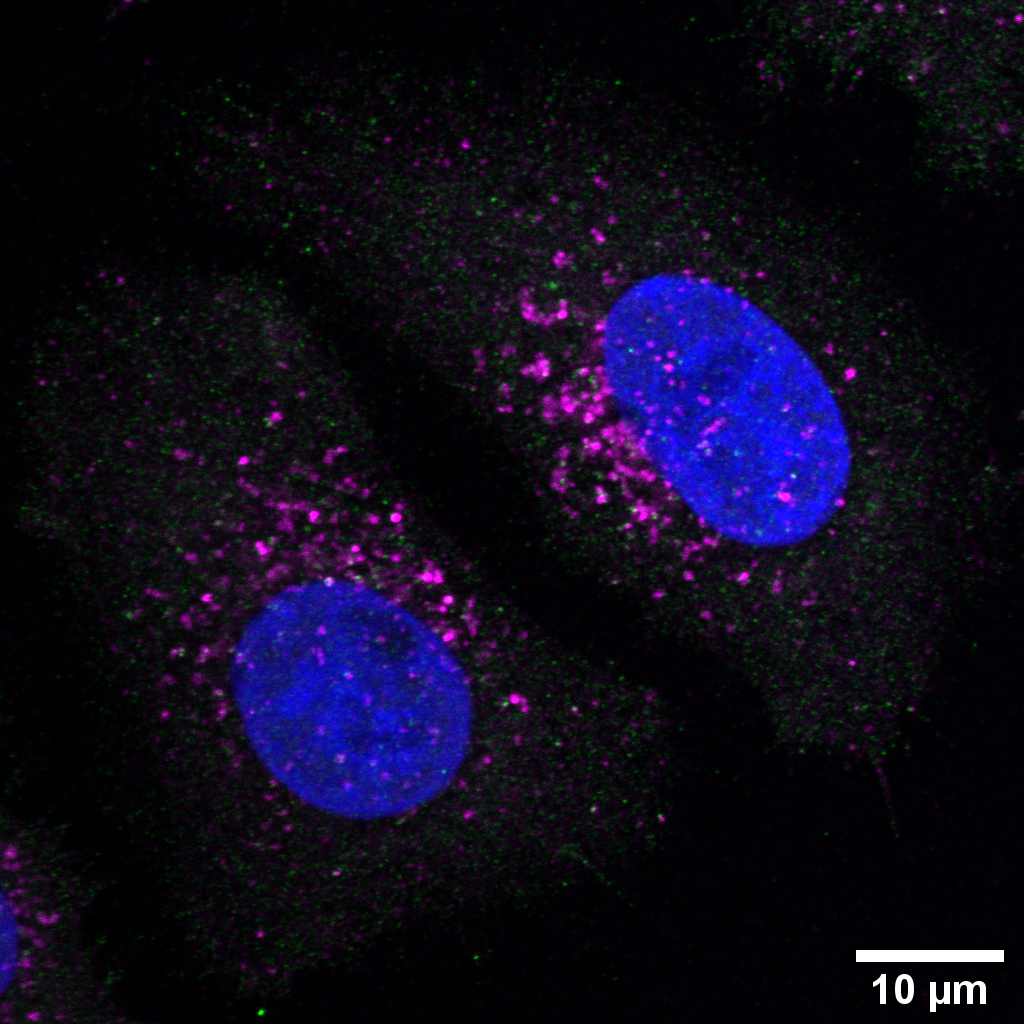

Supplement: Supplementary file 9 — Figure EV1-5 Source Data [file 44318_2025_672_MOESM9_ESM.zip › EV Source Data/EV4/EV4A/8KO_LLOMe_BAPTA_scale.tif]

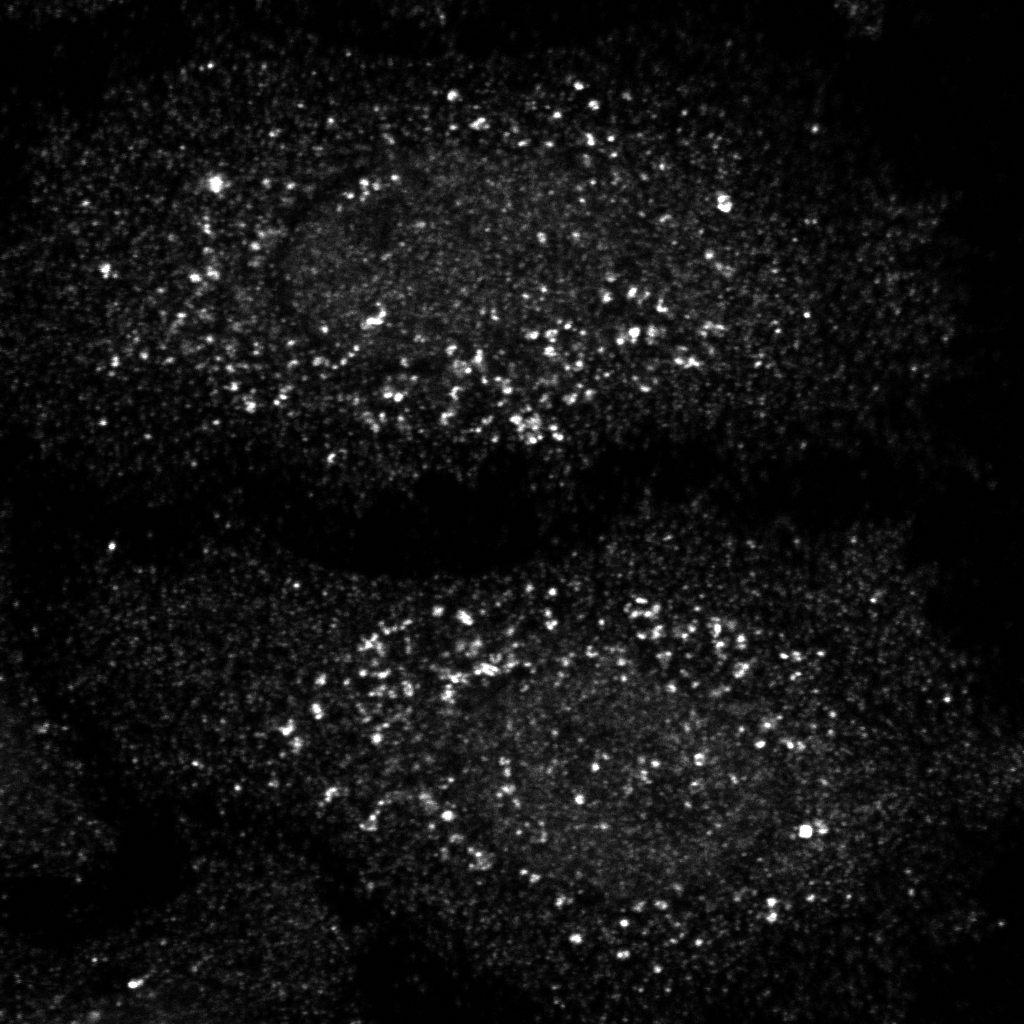

Supplement: Supplementary file 9 — Figure EV1-5 Source Data [file 44318_2025_672_MOESM9_ESM.zip › EV Source Data/EV4/EV4A/8KO_LLOMe_CHMP2A.tif]

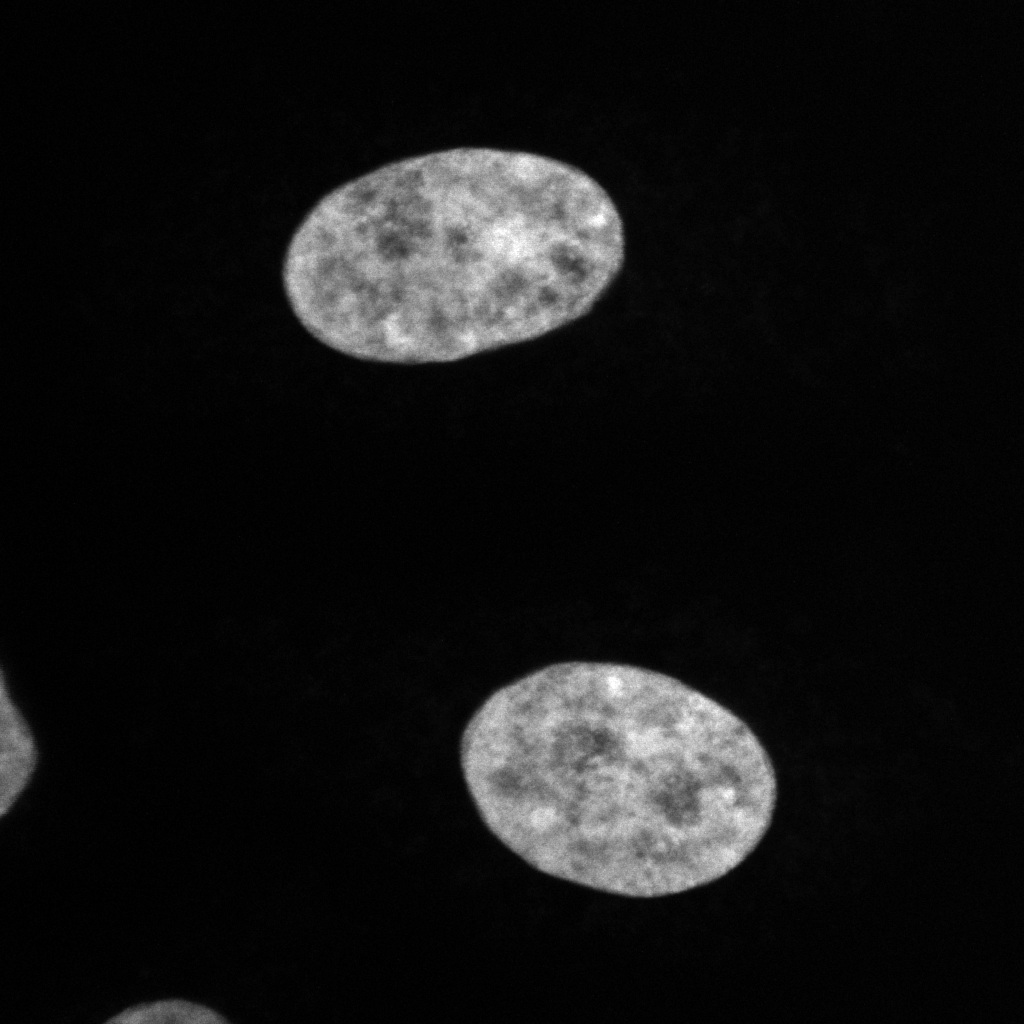

Supplement: Supplementary file 9 — Figure EV1-5 Source Data [file 44318_2025_672_MOESM9_ESM.zip › EV Source Data/EV4/EV4A/8KO_LLOMe_DAPI.tif]

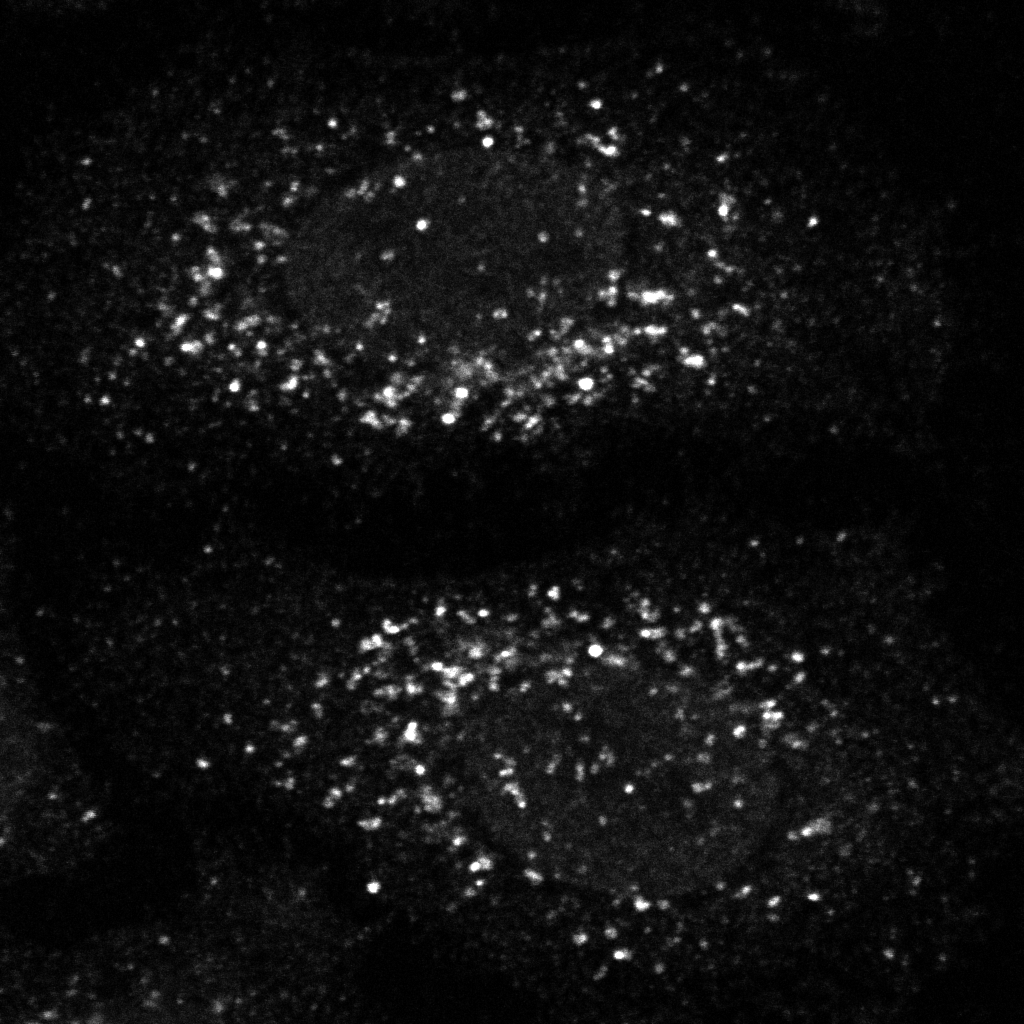

Supplement: Supplementary file 9 — Figure EV1-5 Source Data [file 44318_2025_672_MOESM9_ESM.zip › EV Source Data/EV4/EV4A/8KO_LLOMe_GAL3.tif]

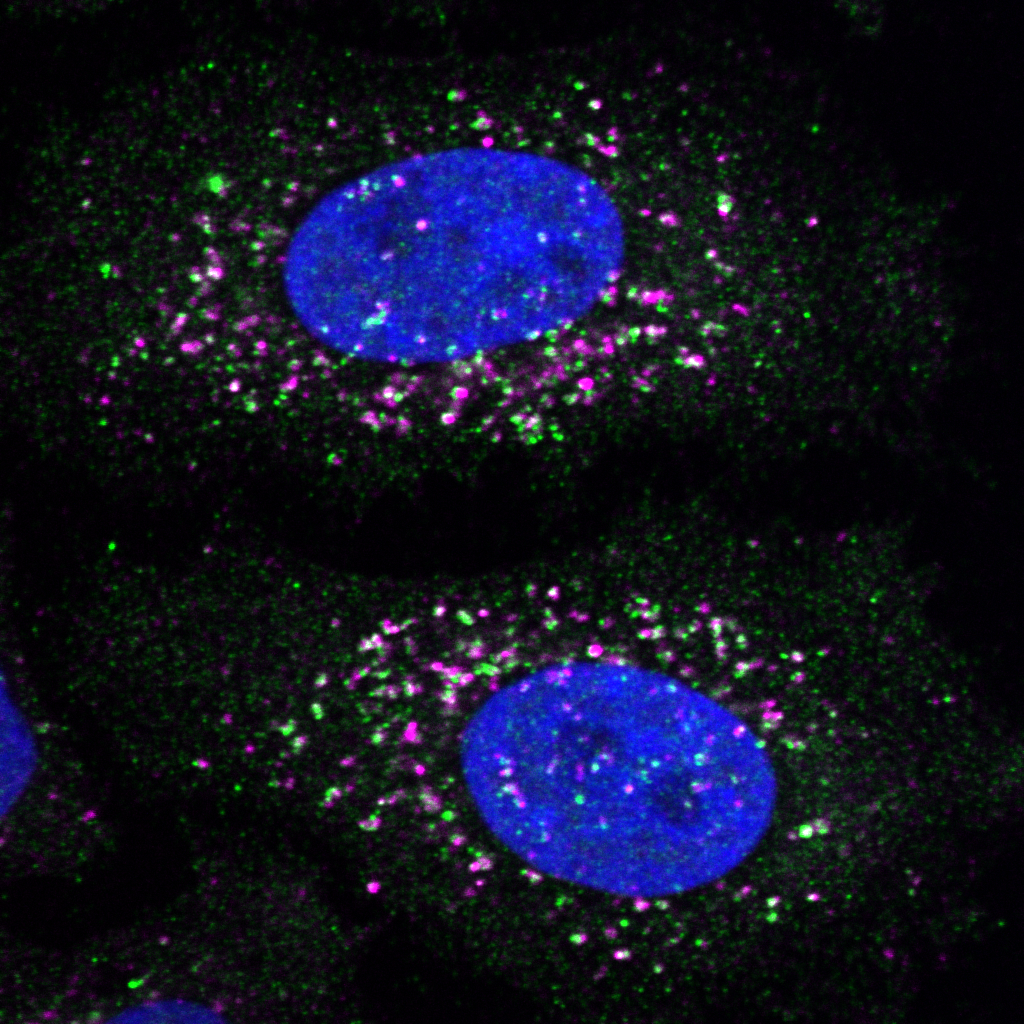

Supplement: Supplementary file 9 — Figure EV1-5 Source Data [file 44318_2025_672_MOESM9_ESM.zip › EV Source Data/EV4/EV4A/8KO_LLOMe_merge.tif]

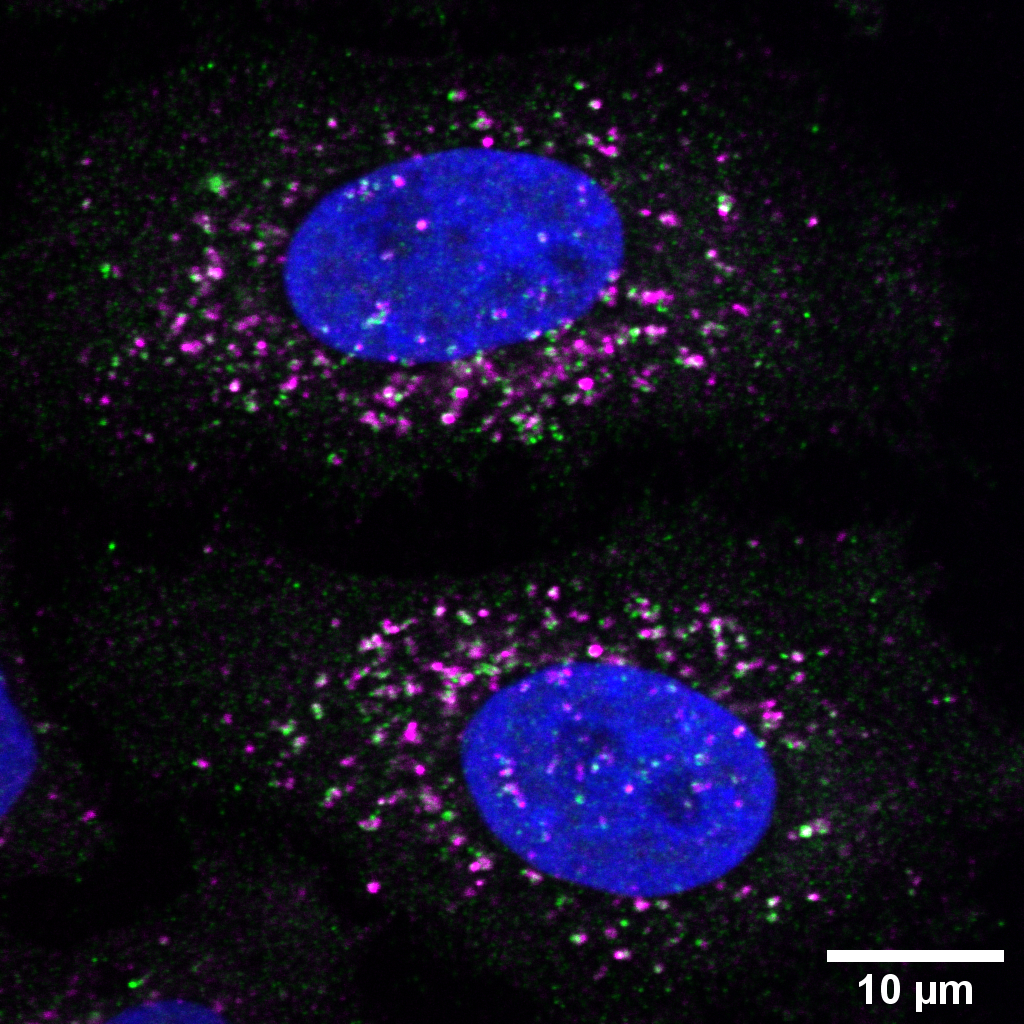

Supplement: Supplementary file 9 — Figure EV1-5 Source Data [file 44318_2025_672_MOESM9_ESM.zip › EV Source Data/EV4/EV4A/8KO_LLOMe_scale.tif]

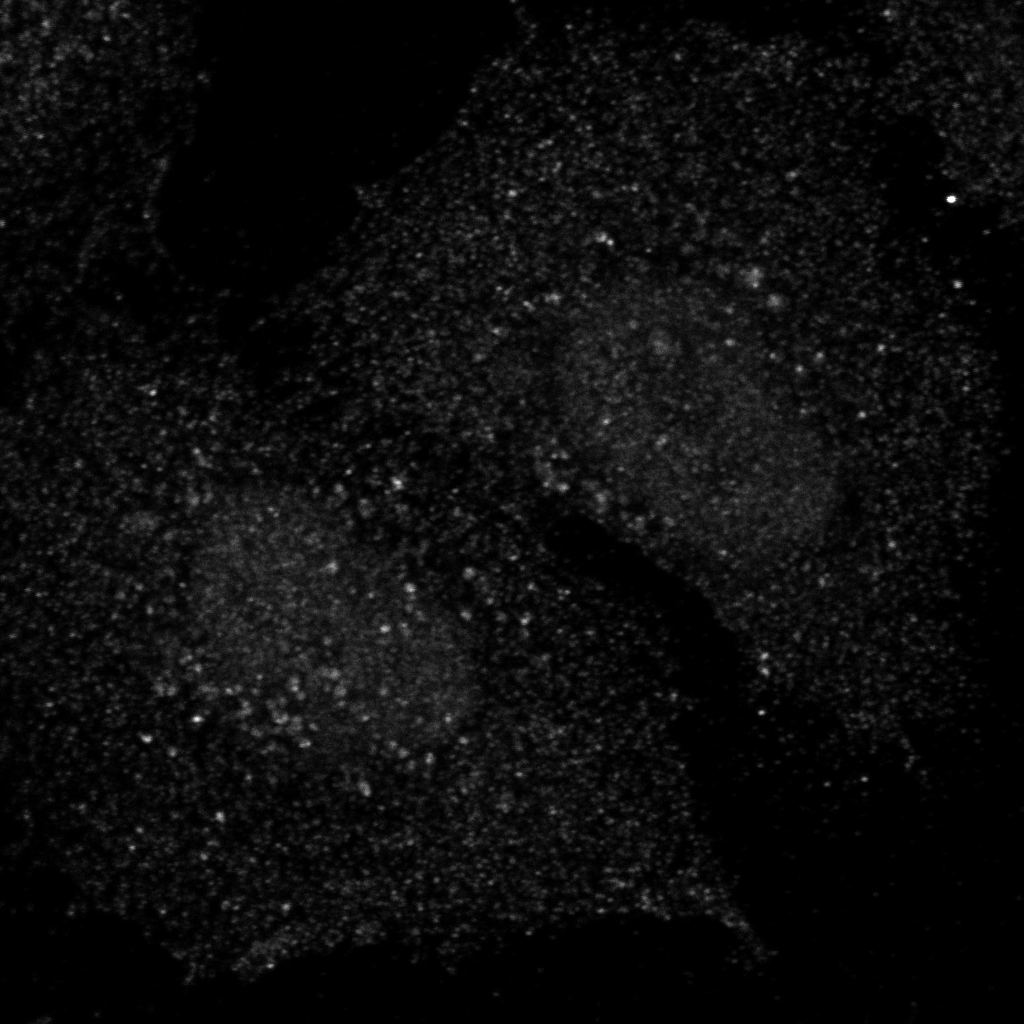

Supplement: Supplementary file 9 — Figure EV1-5 Source Data [file 44318_2025_672_MOESM9_ESM.zip › EV Source Data/EV4/EV4A/DKO_LLOMe_BAPTA_CHMP2A.tif]

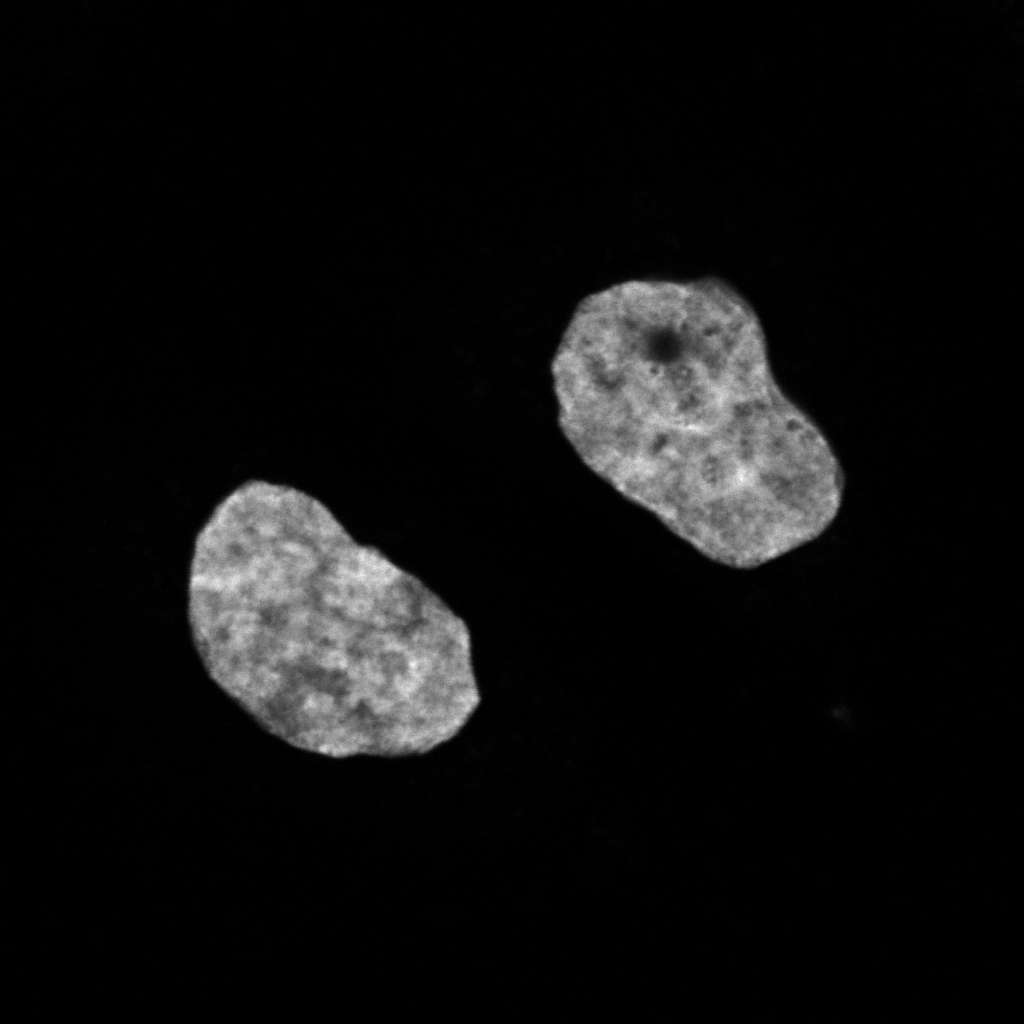

Supplement: Supplementary file 9 — Figure EV1-5 Source Data [file 44318_2025_672_MOESM9_ESM.zip › EV Source Data/EV4/EV4A/DKO_LLOMe_BAPTA_DAPI.tif]

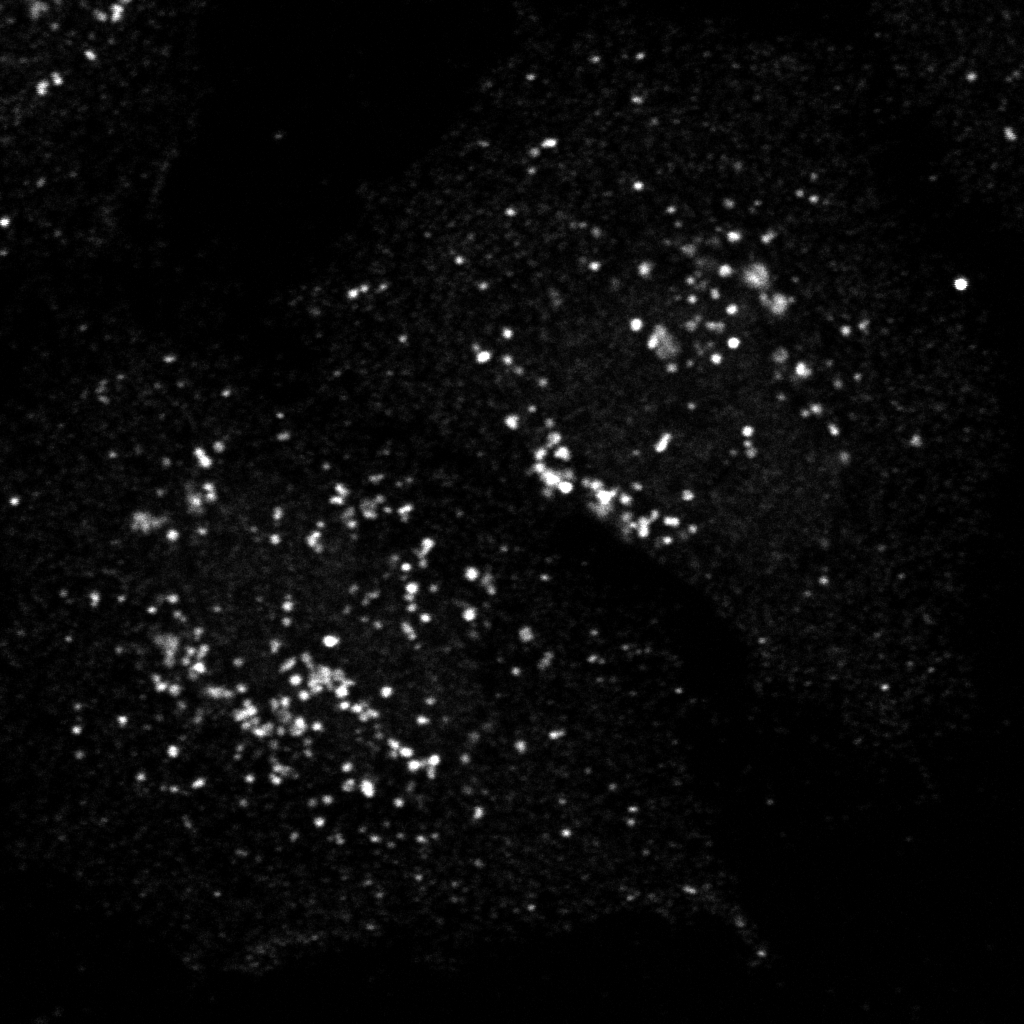

Supplement: Supplementary file 9 — Figure EV1-5 Source Data [file 44318_2025_672_MOESM9_ESM.zip › EV Source Data/EV4/EV4A/DKO_LLOMe_BAPTA_GAL3.tif]

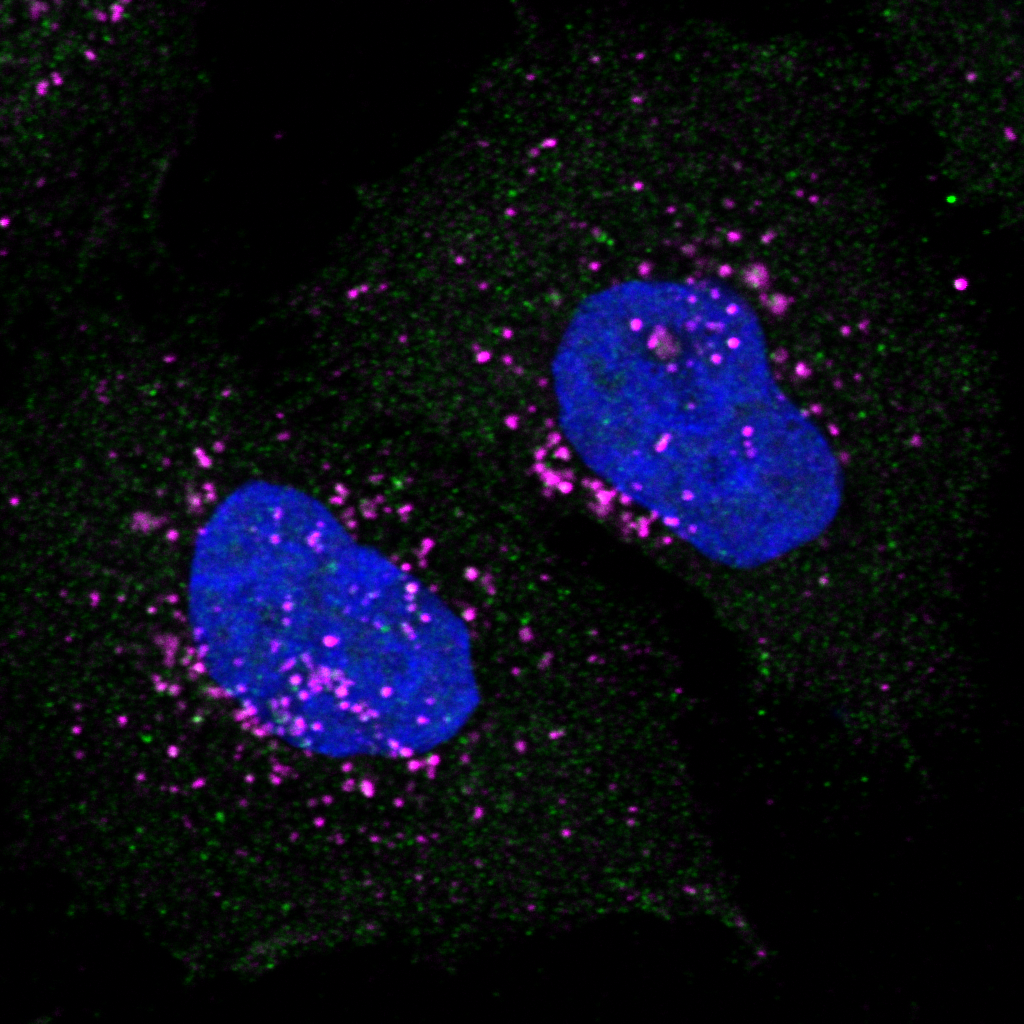

Supplement: Supplementary file 9 — Figure EV1-5 Source Data [file 44318_2025_672_MOESM9_ESM.zip › EV Source Data/EV4/EV4A/DKO_LLOMe_BAPTA_merge.tif]

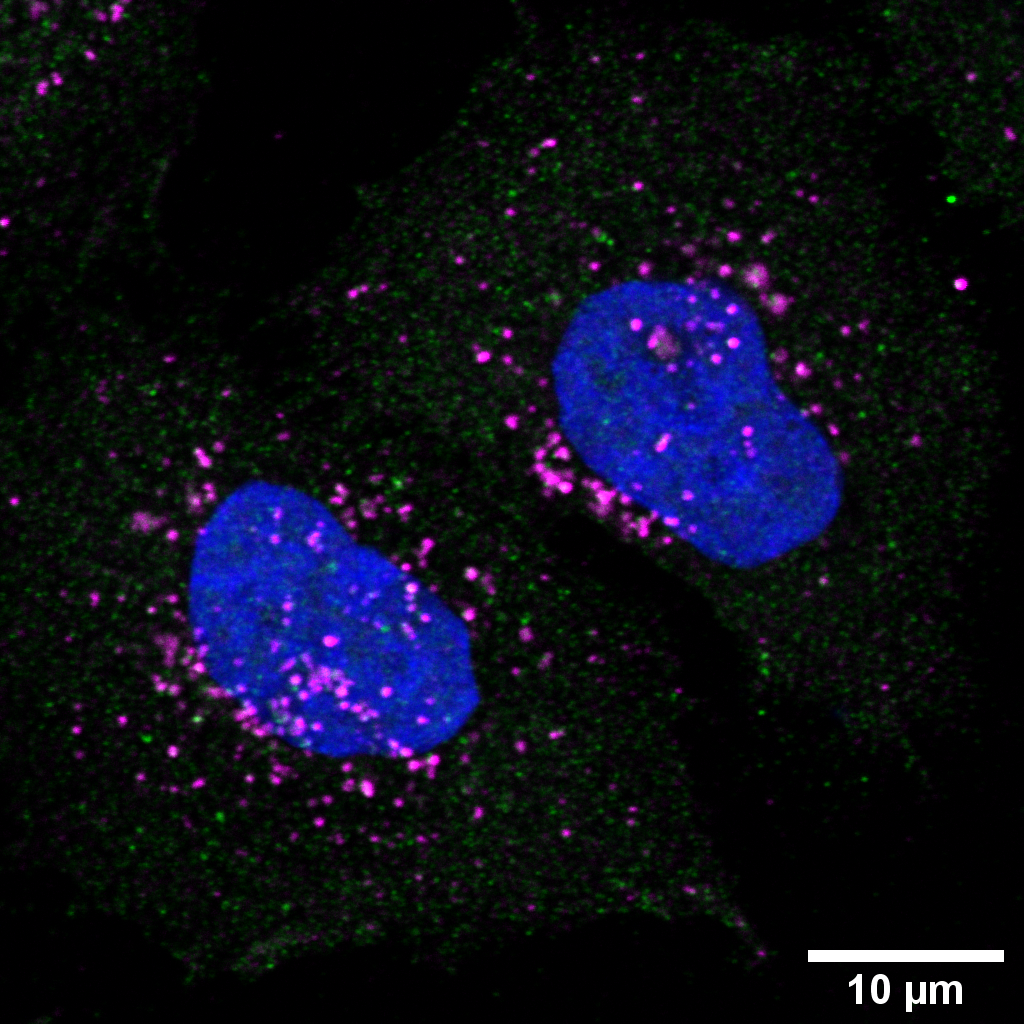

Supplement: Supplementary file 9 — Figure EV1-5 Source Data [file 44318_2025_672_MOESM9_ESM.zip › EV Source Data/EV4/EV4A/DKO_LLOMe_BAPTA_scale.tif]

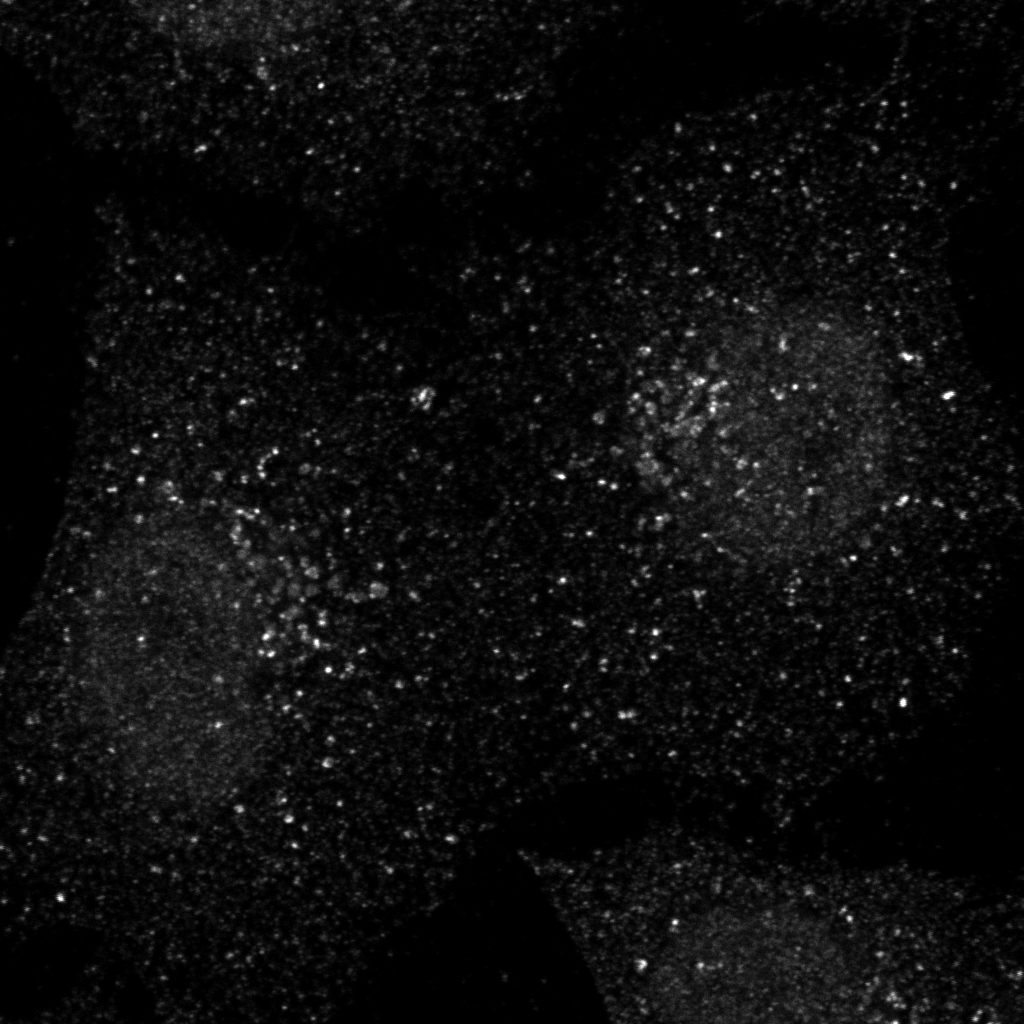

Supplement: Supplementary file 9 — Figure EV1-5 Source Data [file 44318_2025_672_MOESM9_ESM.zip › EV Source Data/EV4/EV4A/DKO_LLOMe_CHMP2A.tif]

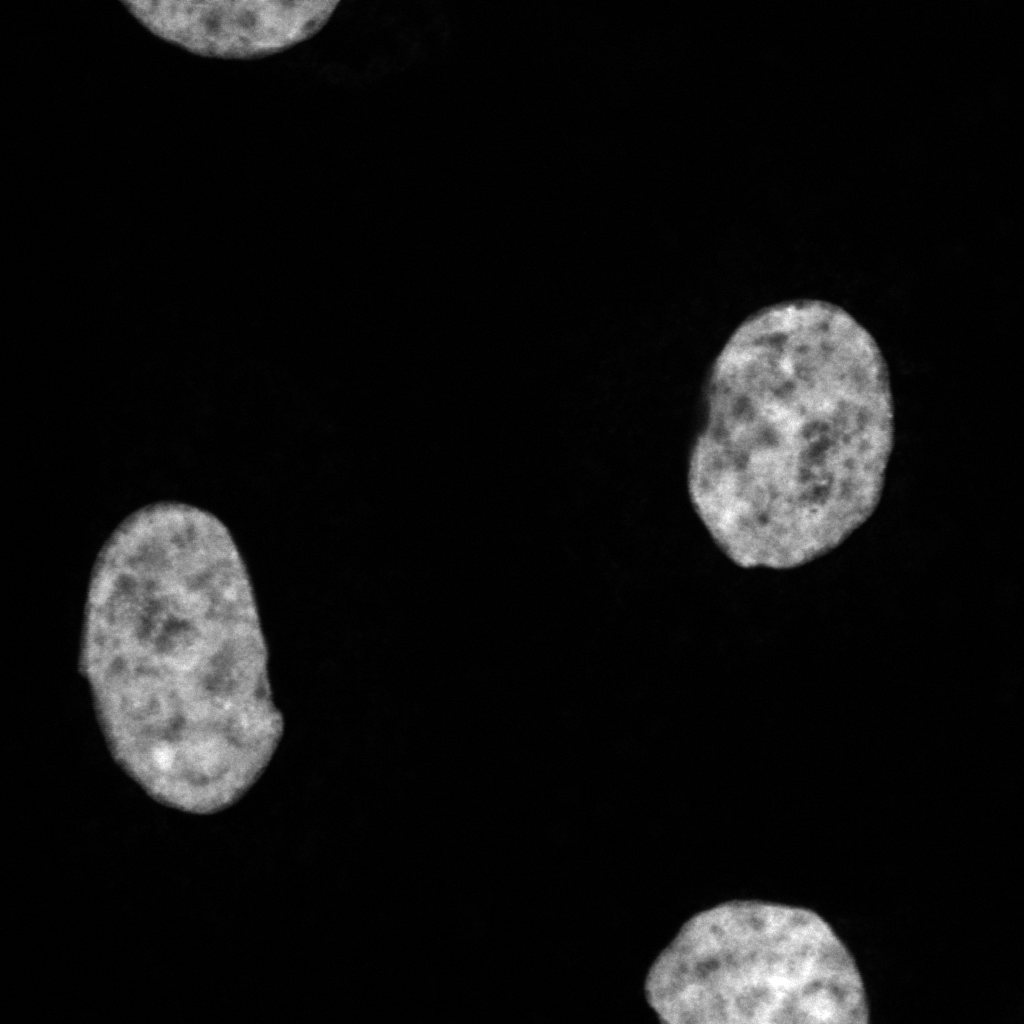

Supplement: Supplementary file 9 — Figure EV1-5 Source Data [file 44318_2025_672_MOESM9_ESM.zip › EV Source Data/EV4/EV4A/DKO_LLOMe_DAPI.tif]

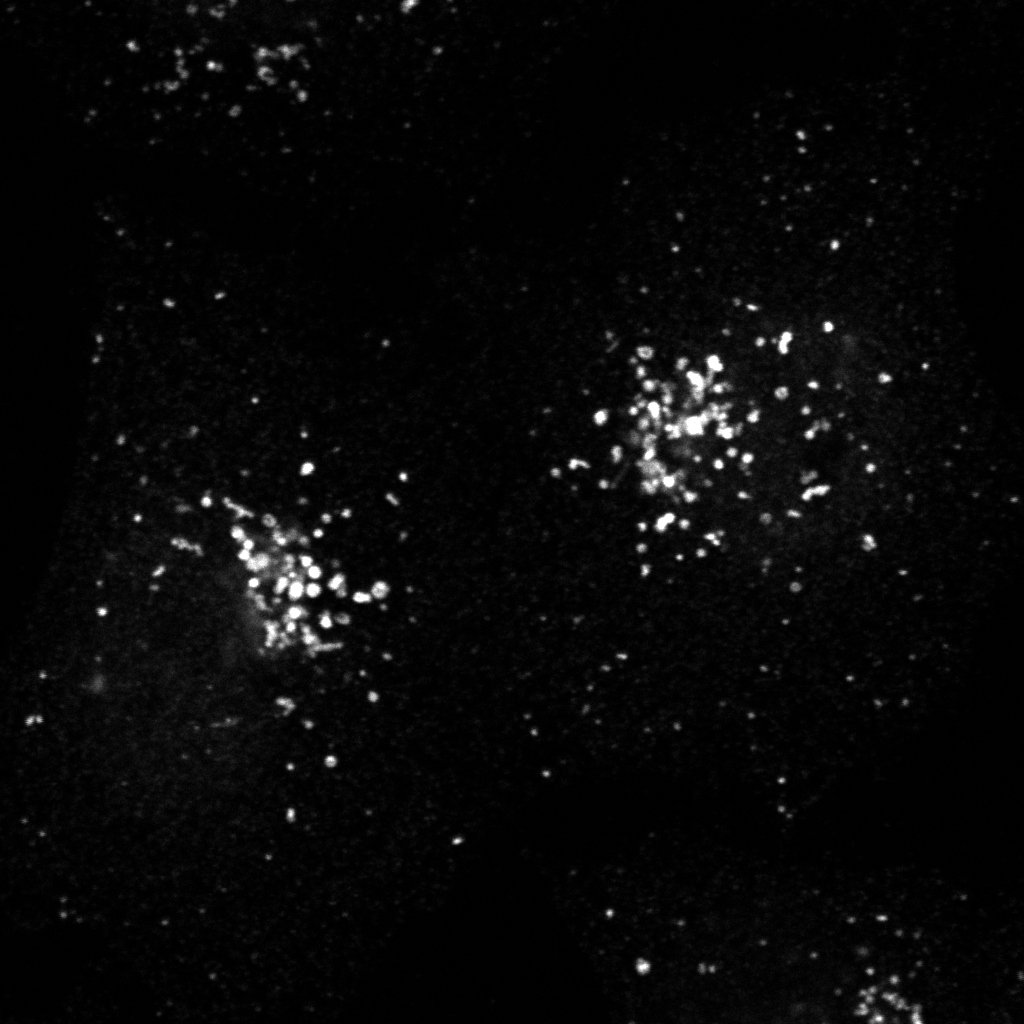

Supplement: Supplementary file 9 — Figure EV1-5 Source Data [file 44318_2025_672_MOESM9_ESM.zip › EV Source Data/EV4/EV4A/DKO_LLOMe_GAL3.tif]

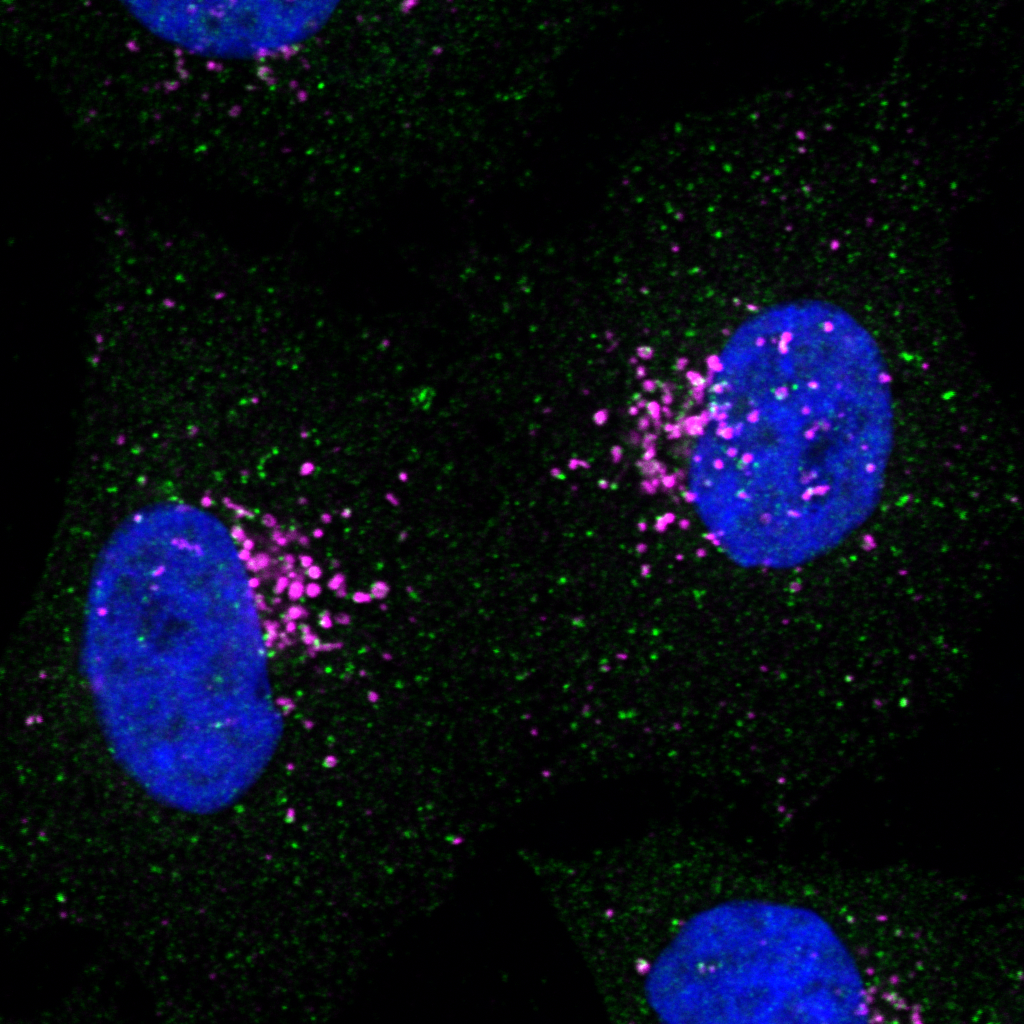

Supplement: Supplementary file 9 — Figure EV1-5 Source Data [file 44318_2025_672_MOESM9_ESM.zip › EV Source Data/EV4/EV4A/DKO_LLOMe_merge.tif]

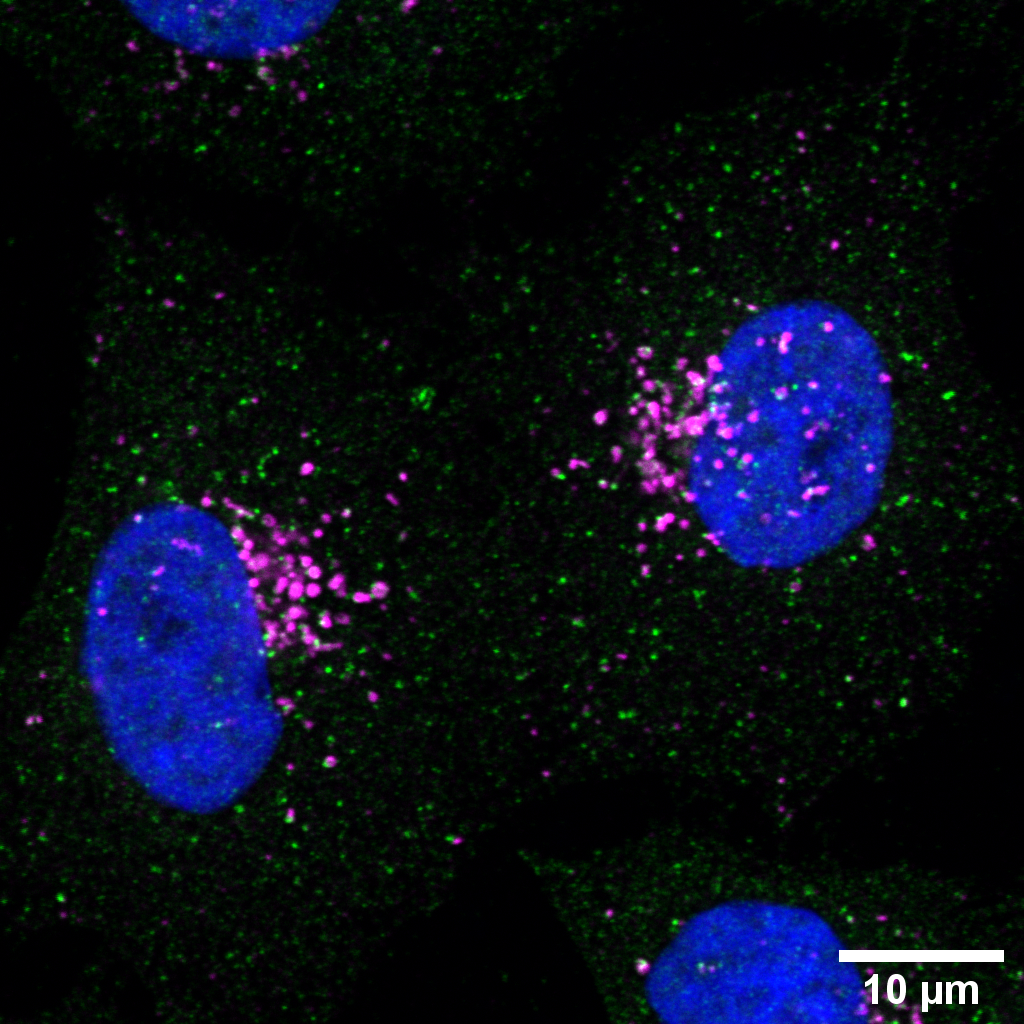

Supplement: Supplementary file 9 — Figure EV1-5 Source Data [file 44318_2025_672_MOESM9_ESM.zip › EV Source Data/EV4/EV4A/DKO_LLOMe_scale.tif]

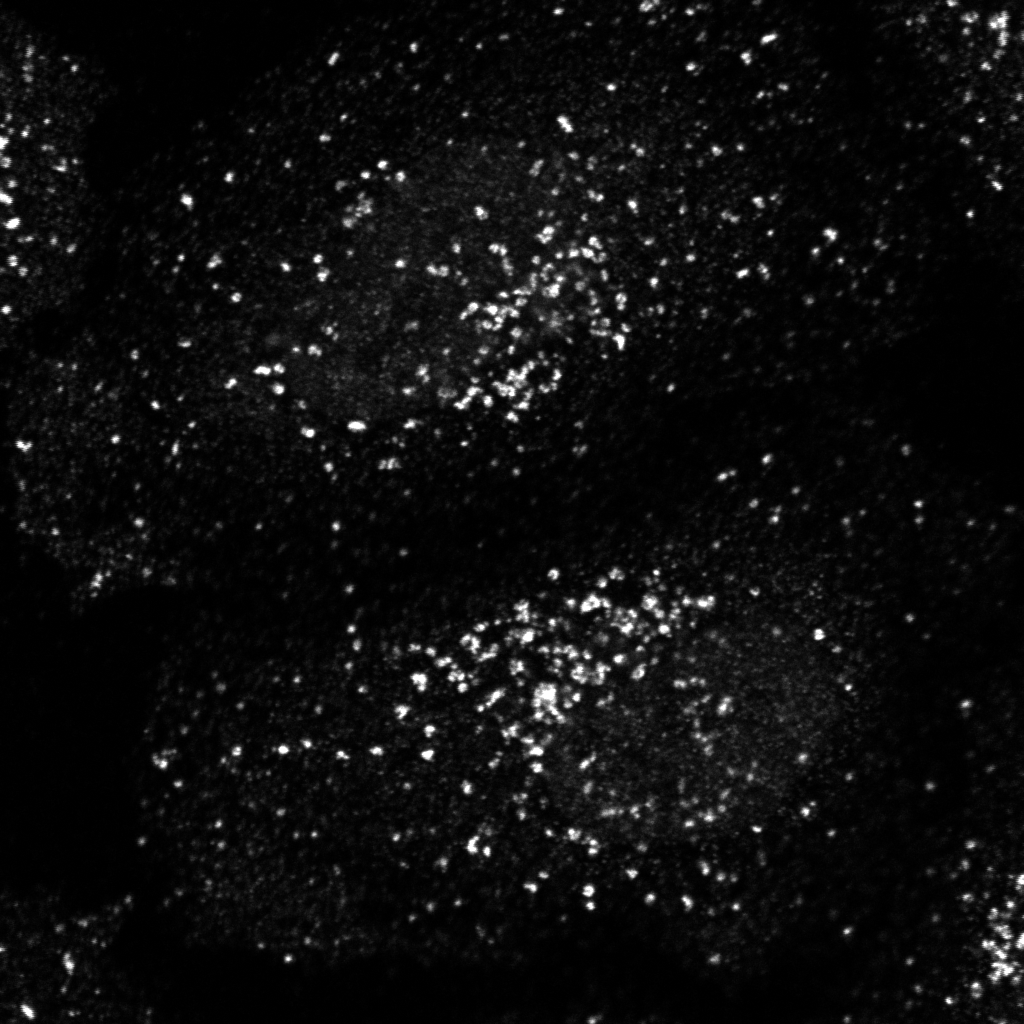

Supplement: Supplementary file 9 — Figure EV1-5 Source Data [file 44318_2025_672_MOESM9_ESM.zip › EV Source Data/EV4/EV4A/WT_LLOMe_BAPTA_CHMP2A.tif]

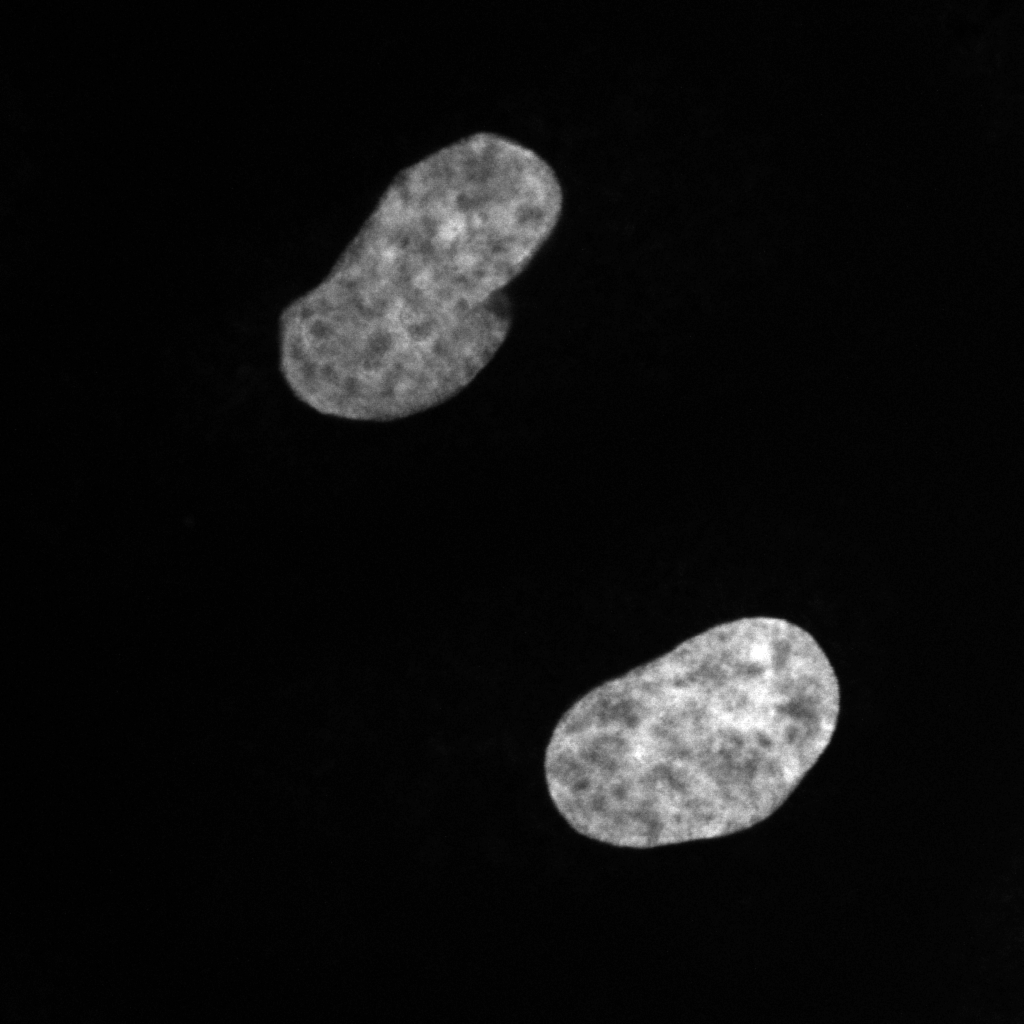

Supplement: Supplementary file 9 — Figure EV1-5 Source Data [file 44318_2025_672_MOESM9_ESM.zip › EV Source Data/EV4/EV4A/WT_LLOMe_BAPTA_DAPI.tif]

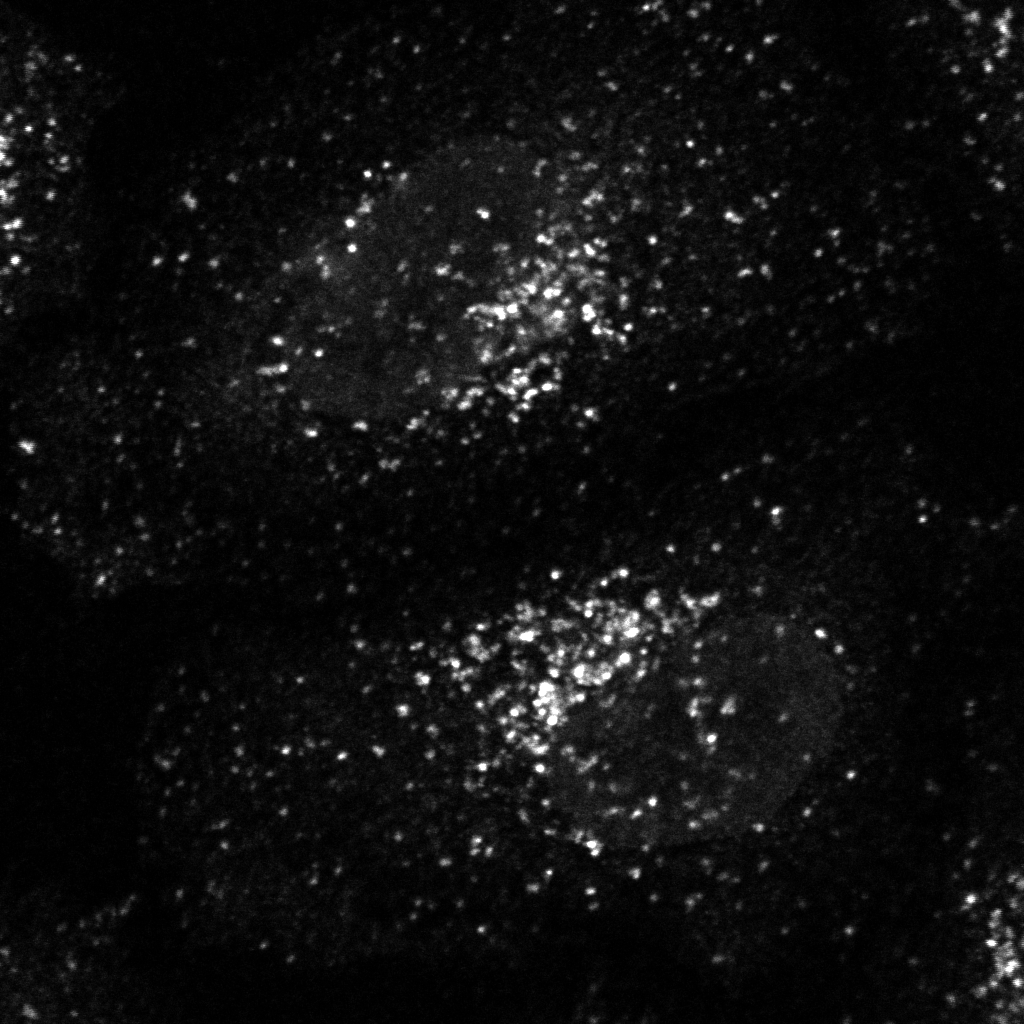

Supplement: Supplementary file 9 — Figure EV1-5 Source Data [file 44318_2025_672_MOESM9_ESM.zip › EV Source Data/EV4/EV4A/WT_LLOMe_BAPTA_GAL3.tif]

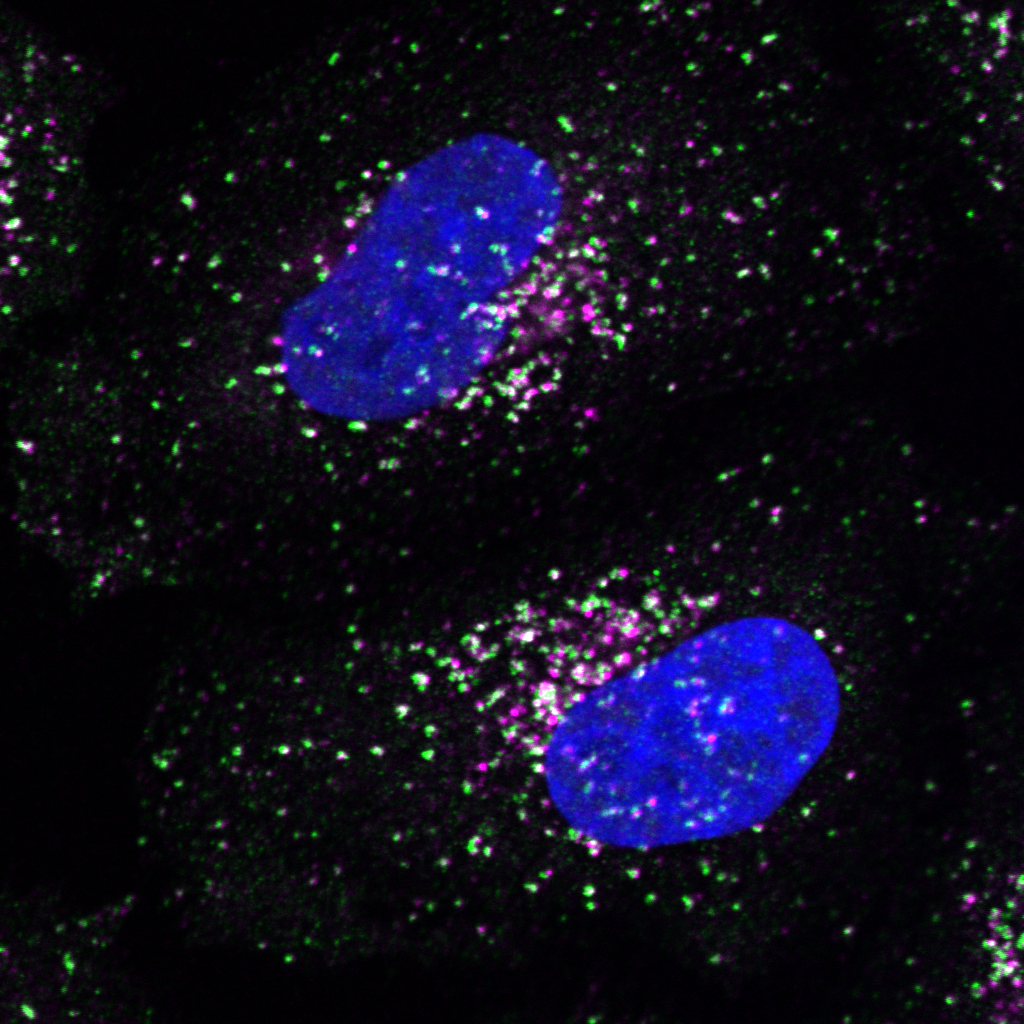

Supplement: Supplementary file 9 — Figure EV1-5 Source Data [file 44318_2025_672_MOESM9_ESM.zip › EV Source Data/EV4/EV4A/WT_LLOMe_BAPTA_merge.tif]

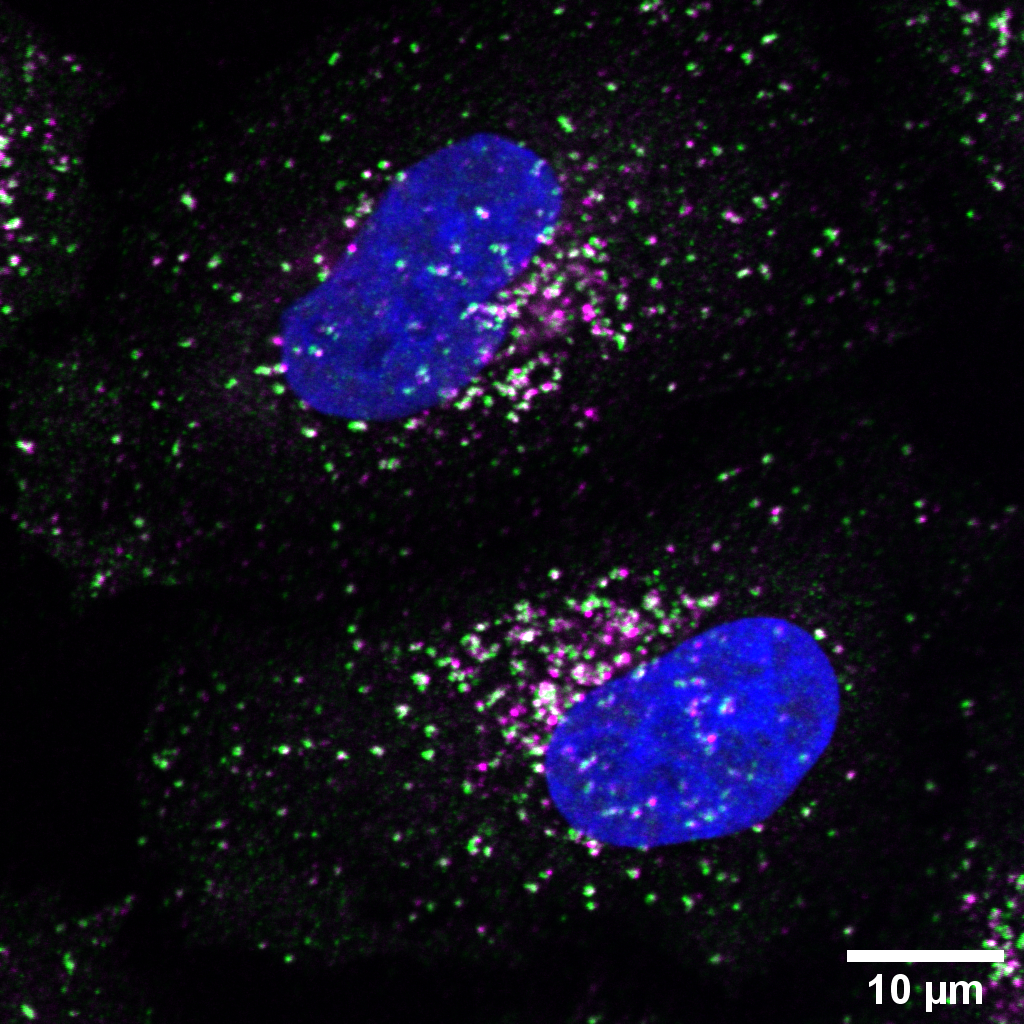

Supplement: Supplementary file 9 — Figure EV1-5 Source Data [file 44318_2025_672_MOESM9_ESM.zip › EV Source Data/EV4/EV4A/WT_LLOMe_BAPTA_scale.tif]

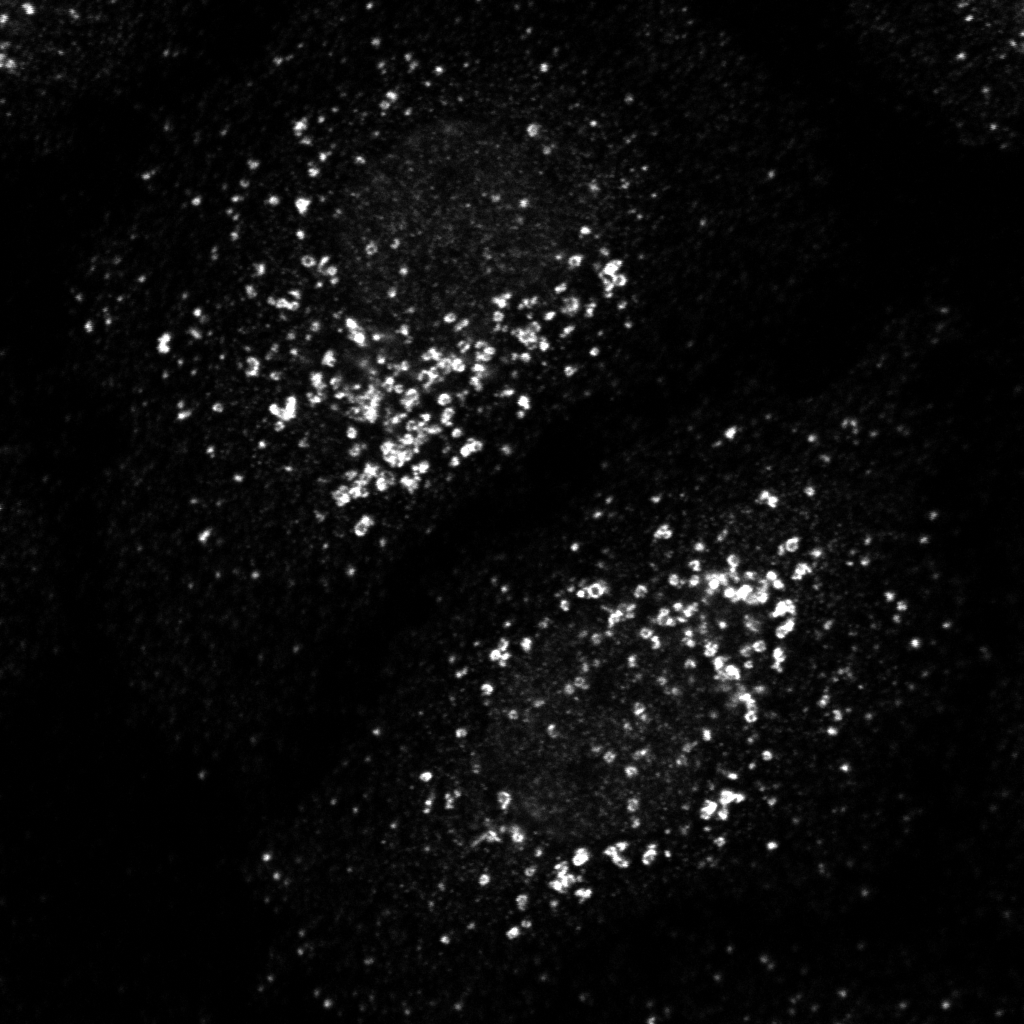

Supplement: Supplementary file 9 — Figure EV1-5 Source Data [file 44318_2025_672_MOESM9_ESM.zip › EV Source Data/EV4/EV4A/WT_LLOMe_CHMP2A.tif]

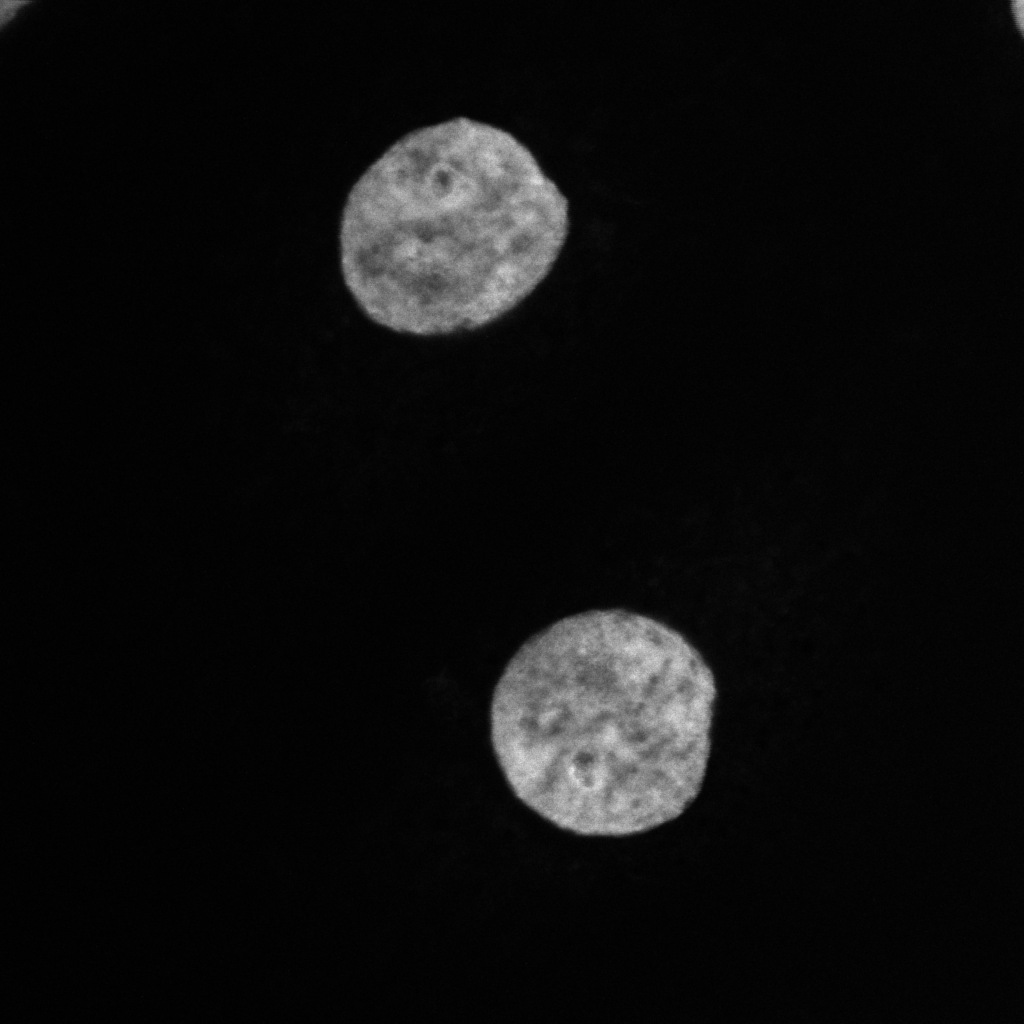

Supplement: Supplementary file 9 — Figure EV1-5 Source Data [file 44318_2025_672_MOESM9_ESM.zip › EV Source Data/EV4/EV4A/WT_LLOMe_DAPI.tif]

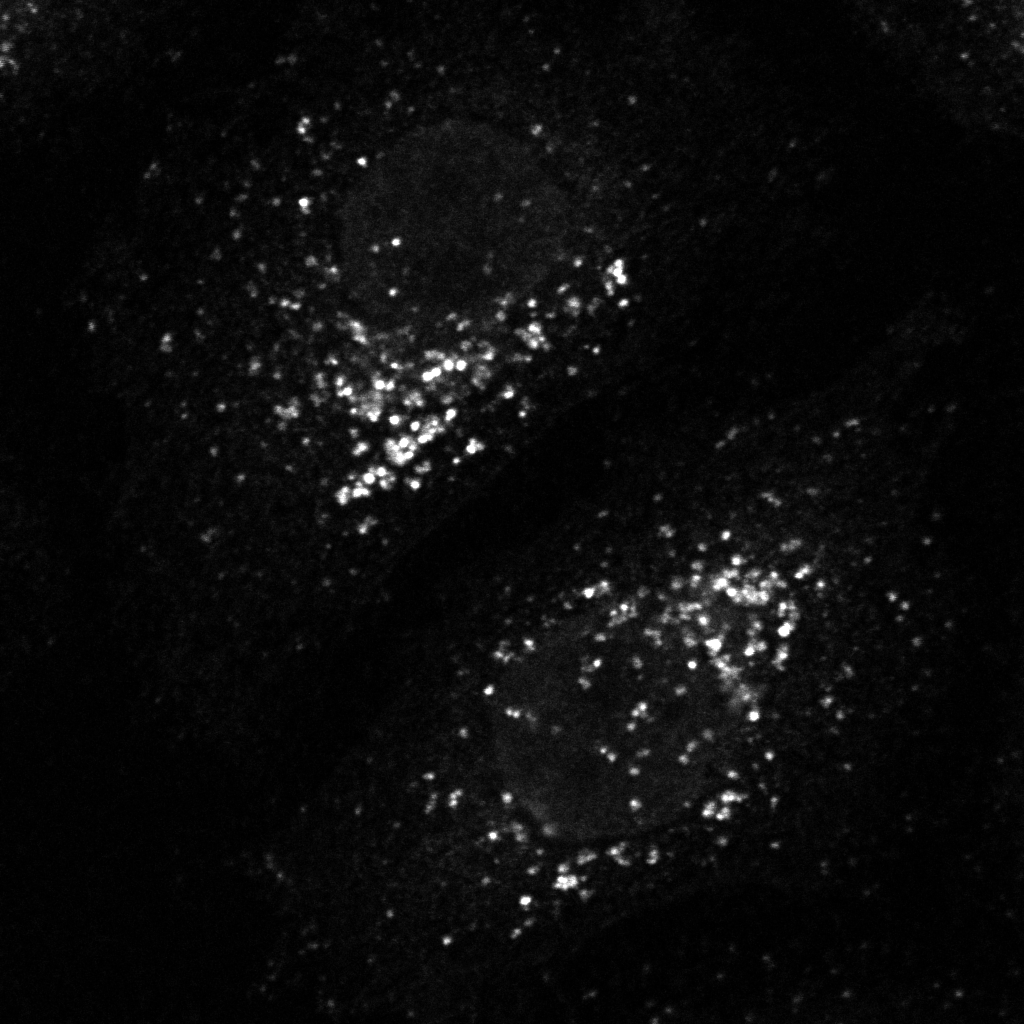

Supplement: Supplementary file 9 — Figure EV1-5 Source Data [file 44318_2025_672_MOESM9_ESM.zip › EV Source Data/EV4/EV4A/WT_LLOMe_GAL3.tif]

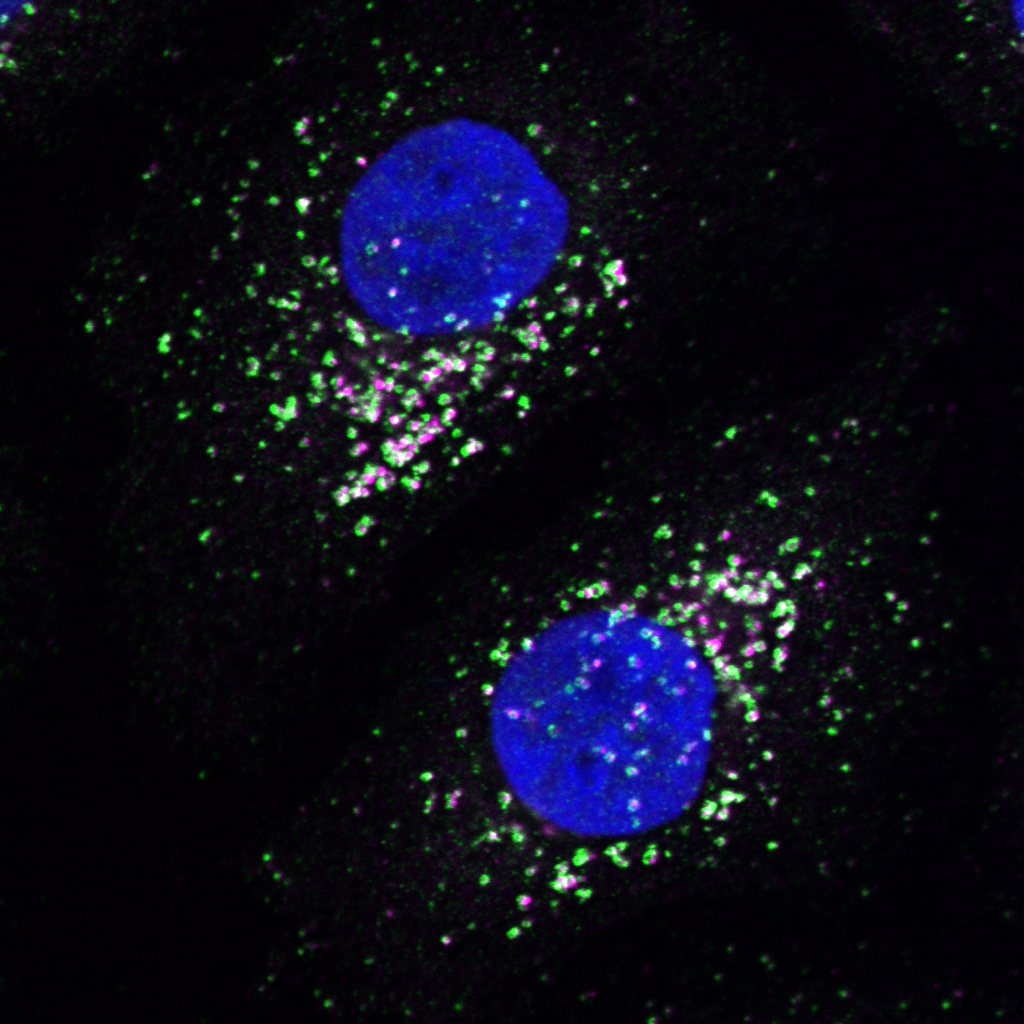

Supplement: Supplementary file 9 — Figure EV1-5 Source Data [file 44318_2025_672_MOESM9_ESM.zip › EV Source Data/EV4/EV4A/WT_LLOMe_merge.tif]

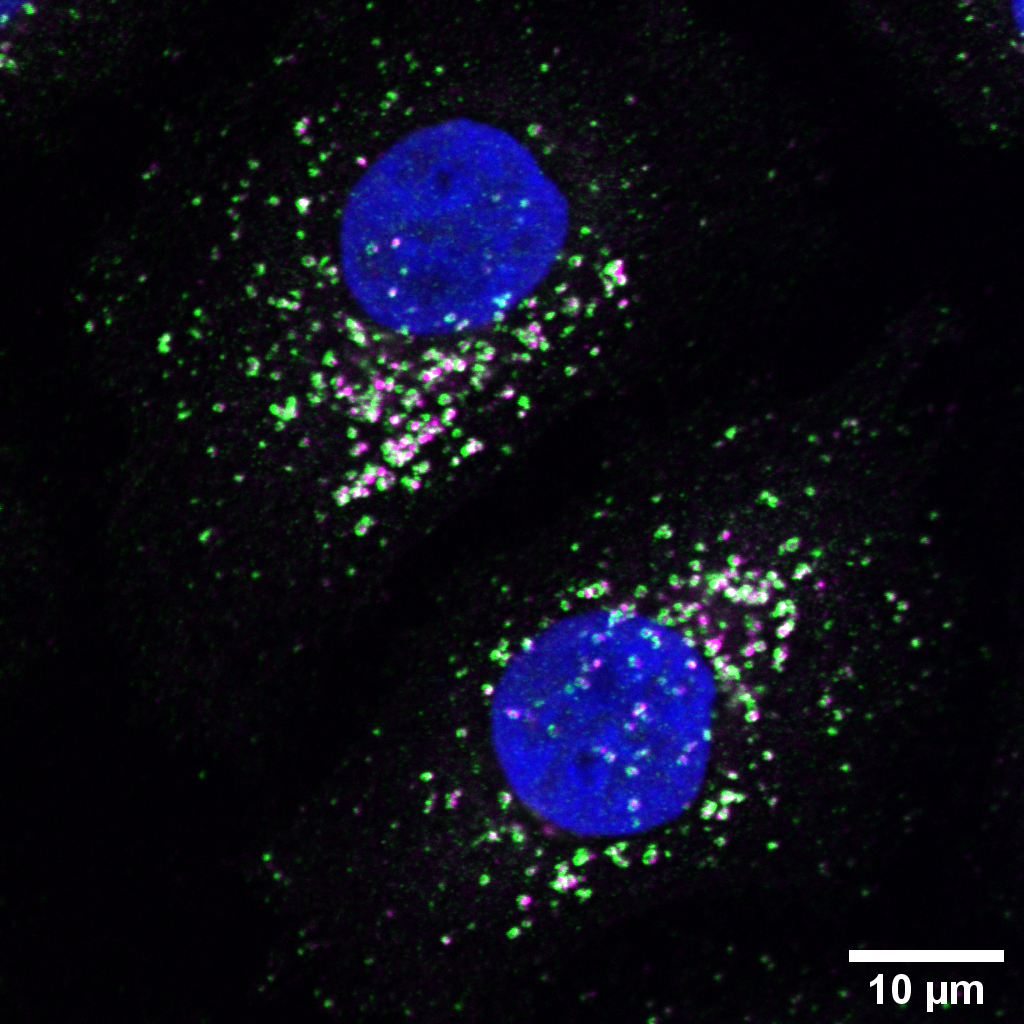

Supplement: Supplementary file 9 — Figure EV1-5 Source Data [file 44318_2025_672_MOESM9_ESM.zip › EV Source Data/EV4/EV4A/WT_LLOMe_scale.tif]

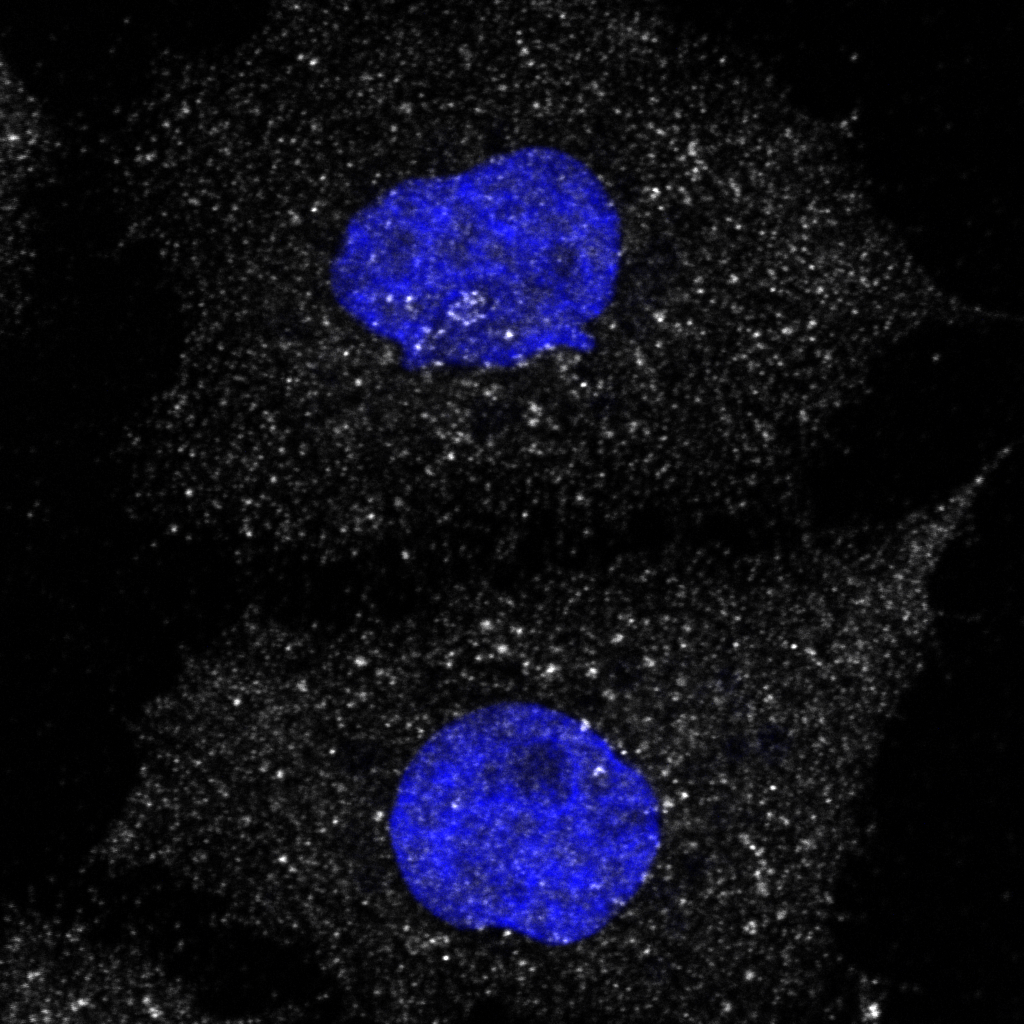

Supplement: Supplementary file 9 — Figure EV1-5 Source Data [file 44318_2025_672_MOESM9_ESM.zip › EV Source Data/EV4/EV4C/16KO_BAPTA LLOMe.tif]

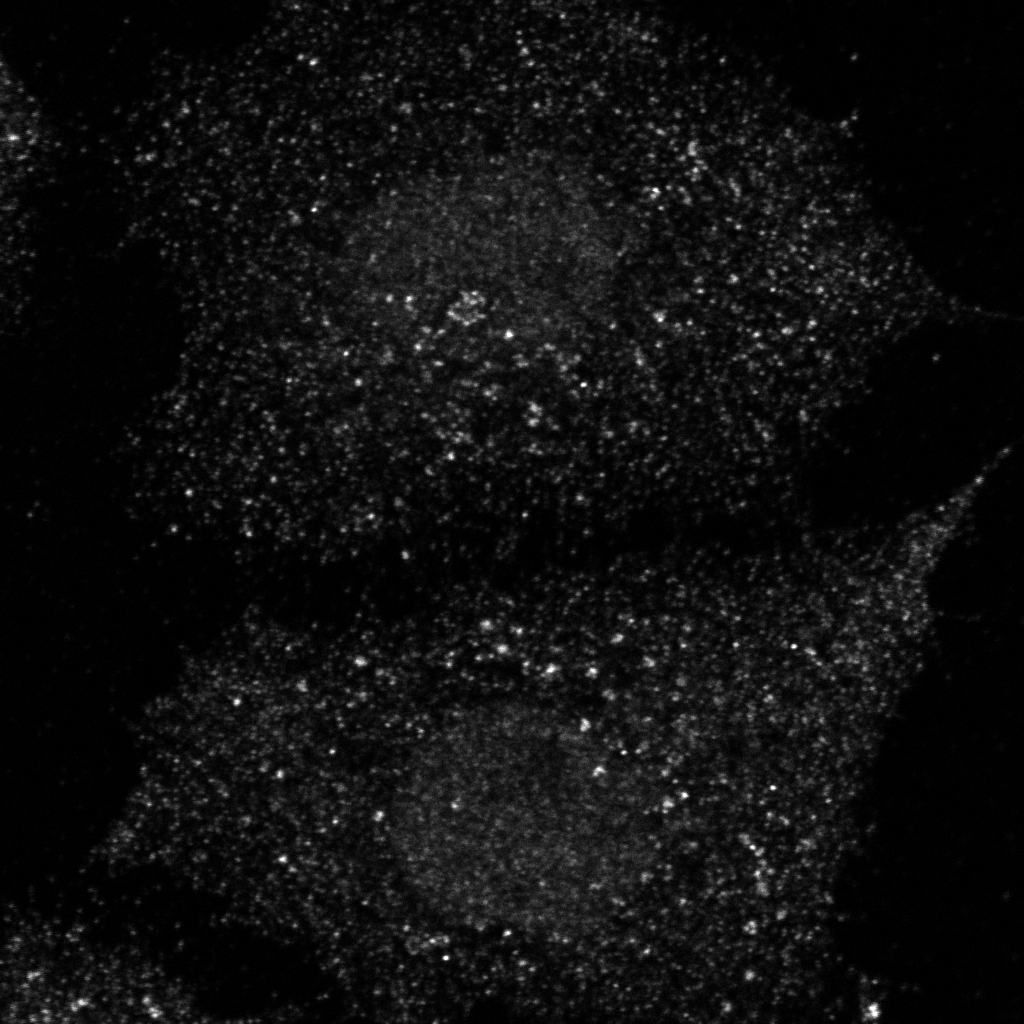

Supplement: Supplementary file 9 — Figure EV1-5 Source Data [file 44318_2025_672_MOESM9_ESM.zip › EV Source Data/EV4/EV4C/16KO_BAPTA LLOMe_CHMP2A.tif]

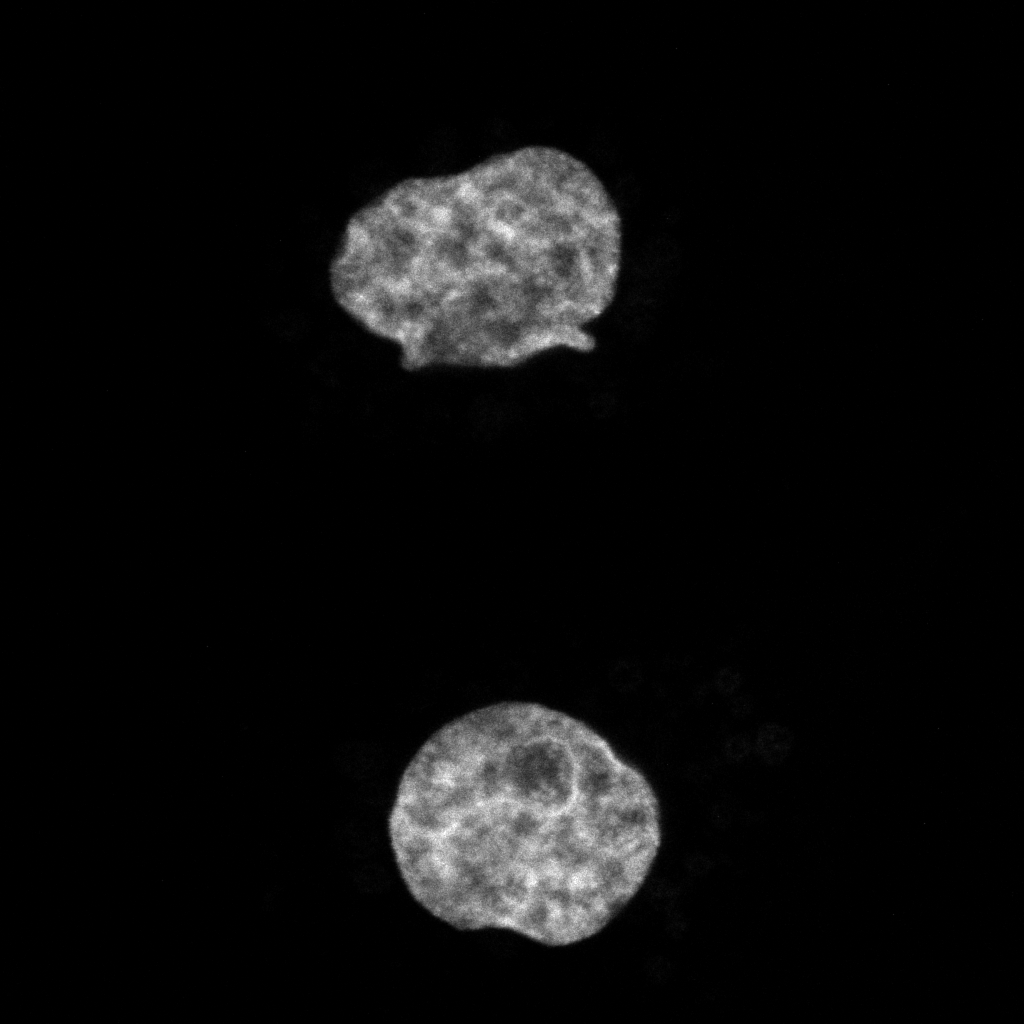

Supplement: Supplementary file 9 — Figure EV1-5 Source Data [file 44318_2025_672_MOESM9_ESM.zip › EV Source Data/EV4/EV4C/16KO_BAPTA LLOMe_DAPI.tif]

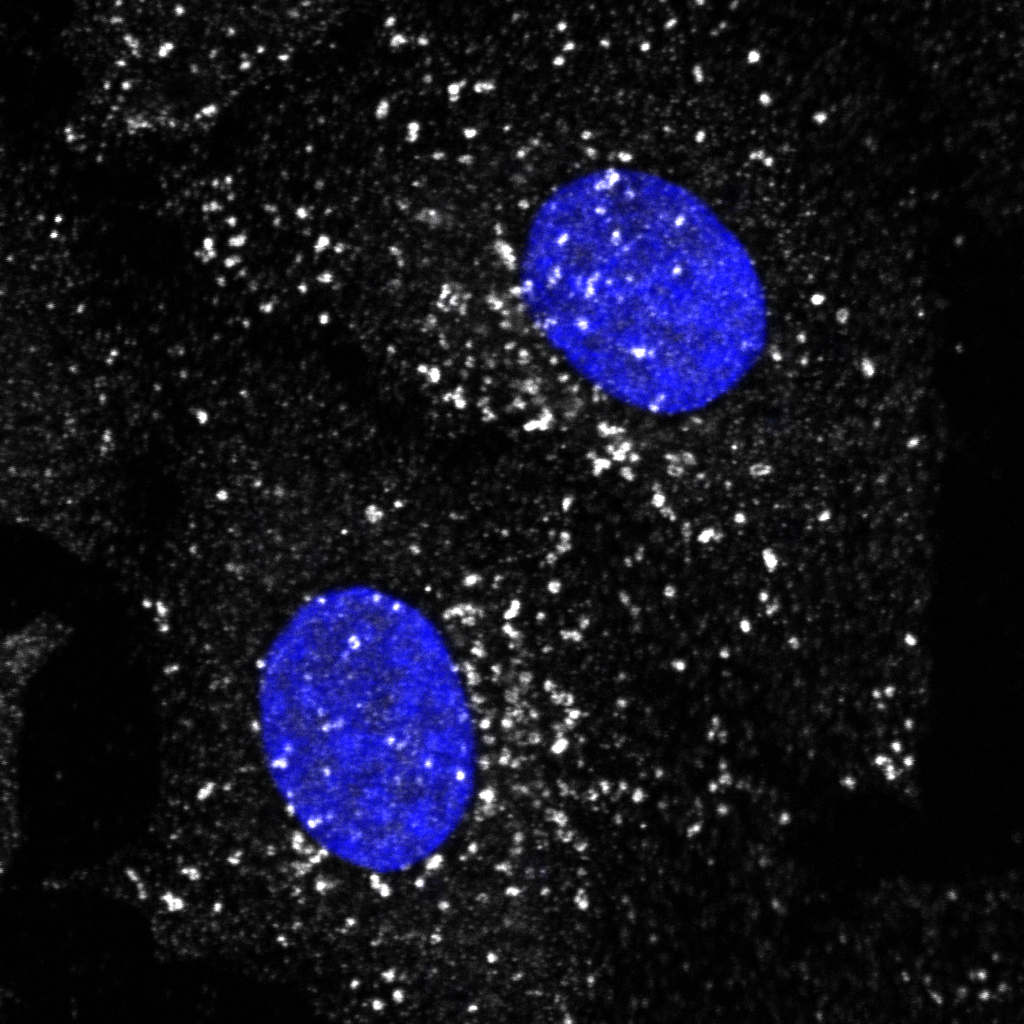

Supplement: Supplementary file 9 — Figure EV1-5 Source Data [file 44318_2025_672_MOESM9_ESM.zip › EV Source Data/EV4/EV4C/16KO_LLOMe.tif]

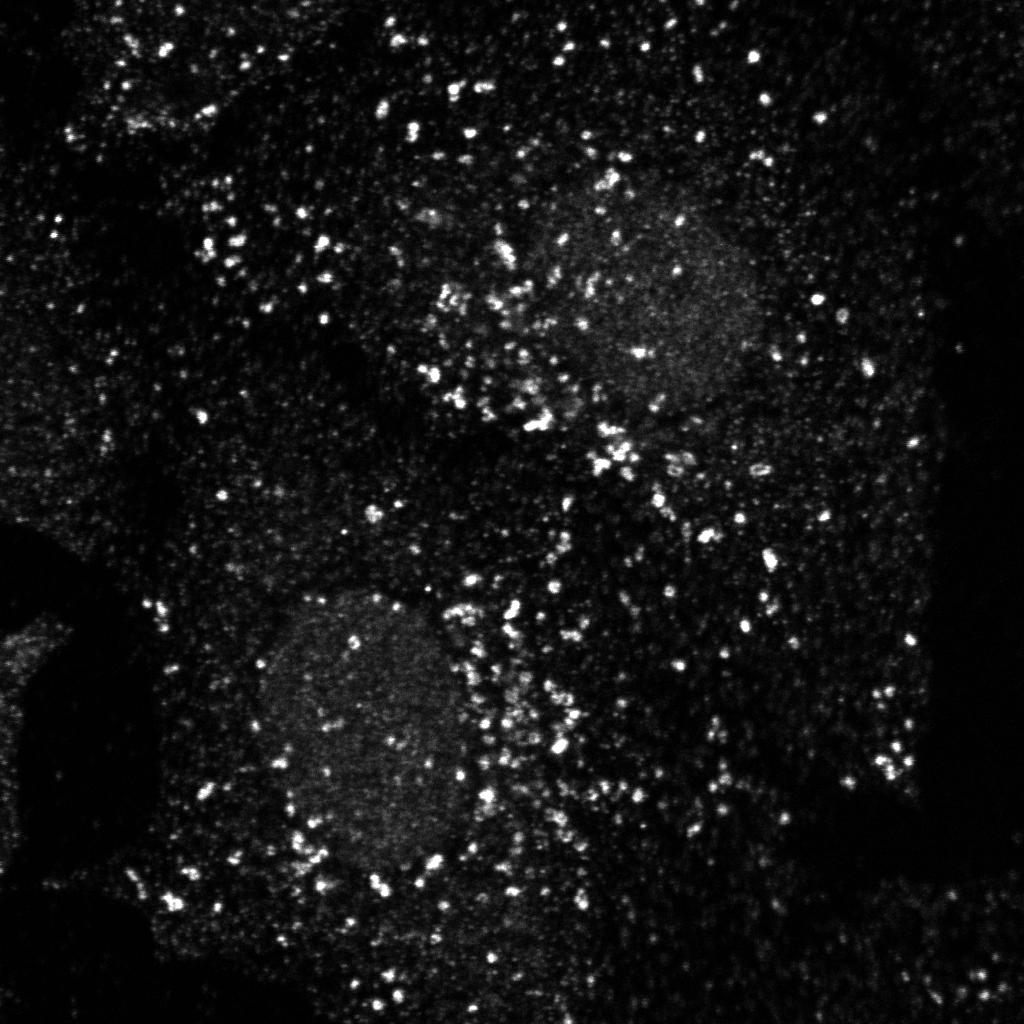

Supplement: Supplementary file 9 — Figure EV1-5 Source Data [file 44318_2025_672_MOESM9_ESM.zip › EV Source Data/EV4/EV4C/16KO_LLOMe_CHMP2A.tif]

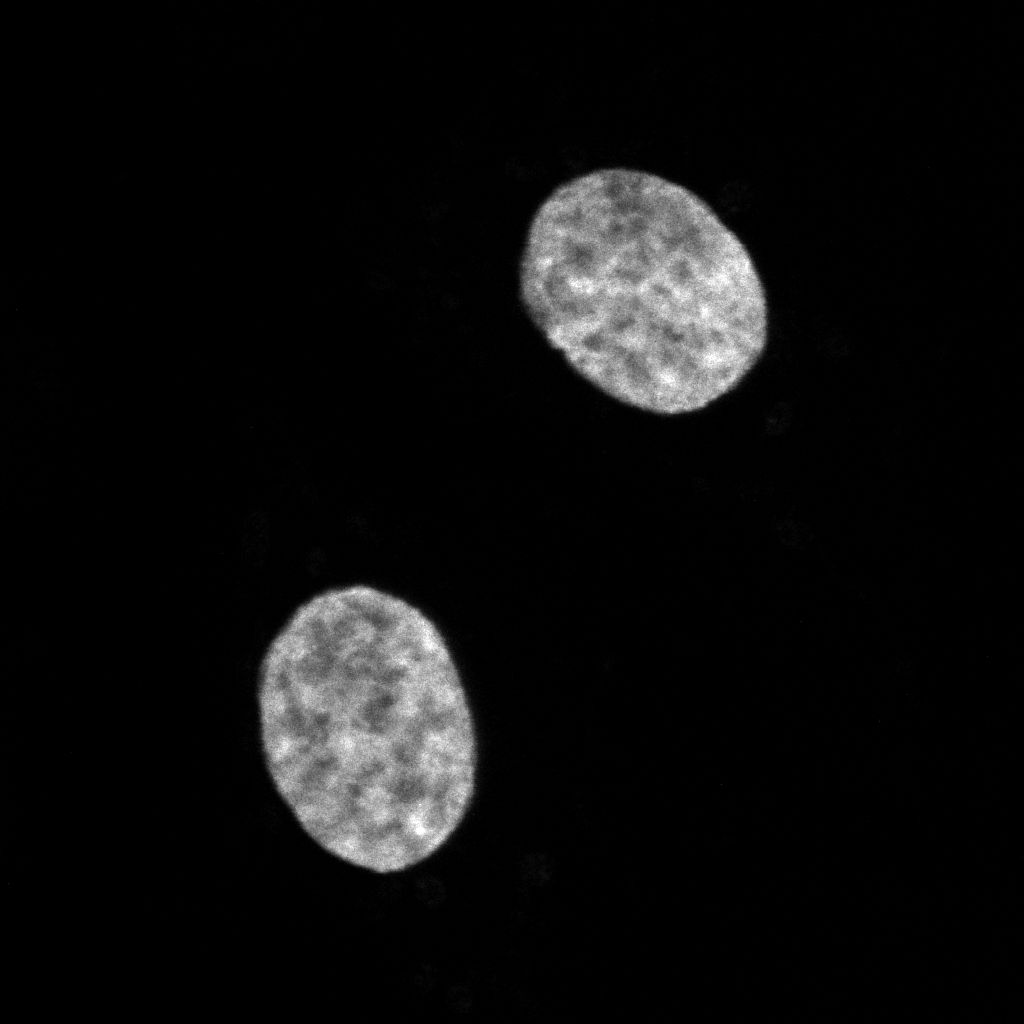

Supplement: Supplementary file 9 — Figure EV1-5 Source Data [file 44318_2025_672_MOESM9_ESM.zip › EV Source Data/EV4/EV4C/16KO_LLOMe_DAPI.tif]

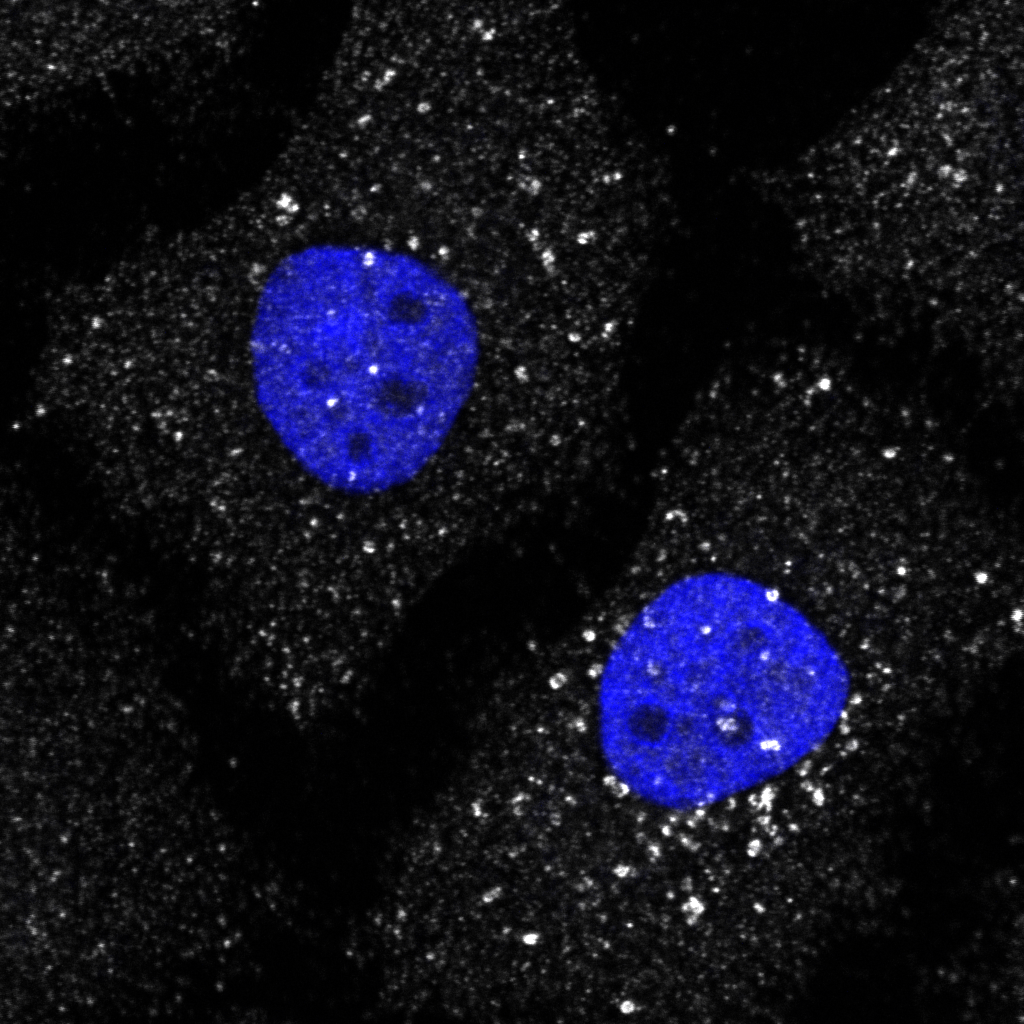

Supplement: Supplementary file 9 — Figure EV1-5 Source Data [file 44318_2025_672_MOESM9_ESM.zip › EV Source Data/EV4/EV4C/TECPR1KO_BAPTA LLOMe.tif]

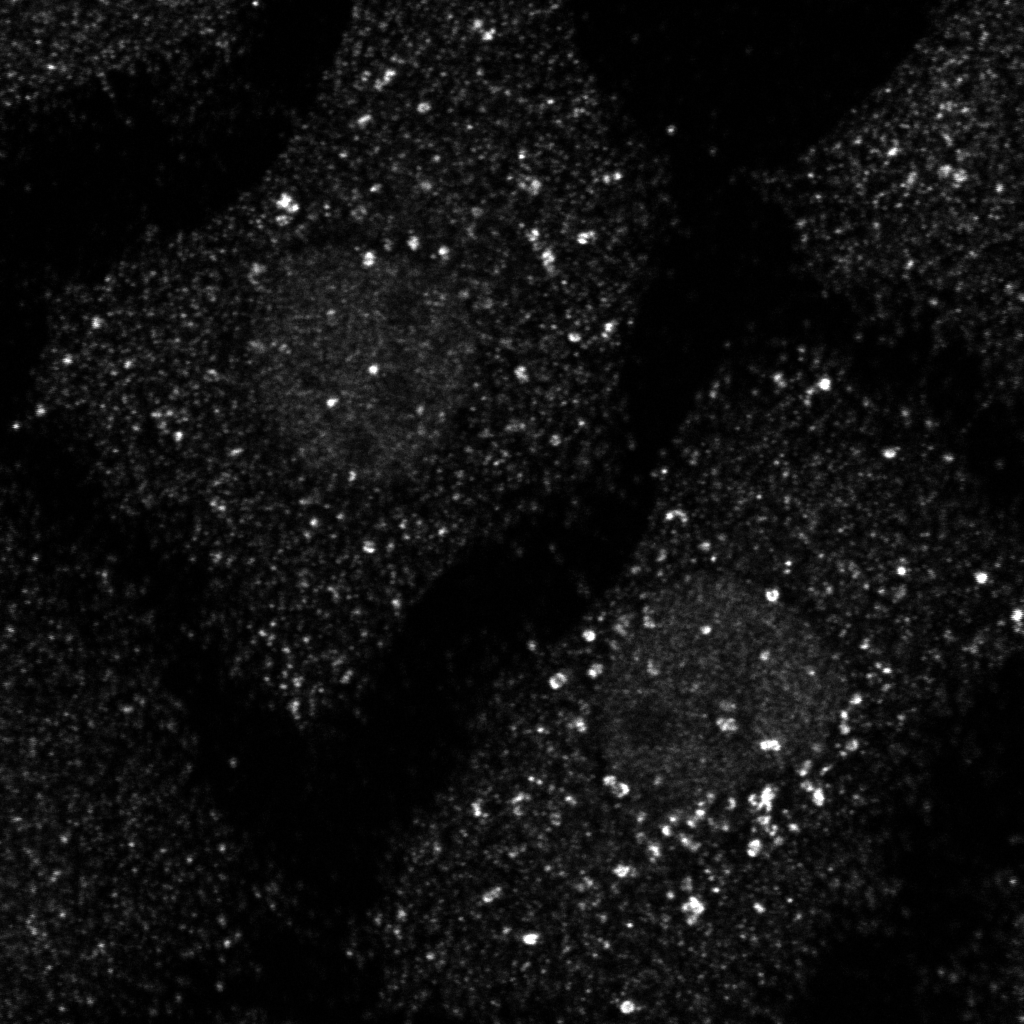

Supplement: Supplementary file 9 — Figure EV1-5 Source Data [file 44318_2025_672_MOESM9_ESM.zip › EV Source Data/EV4/EV4C/TECPR1KO_BAPTA LLOMe_CHMP2A.tif]

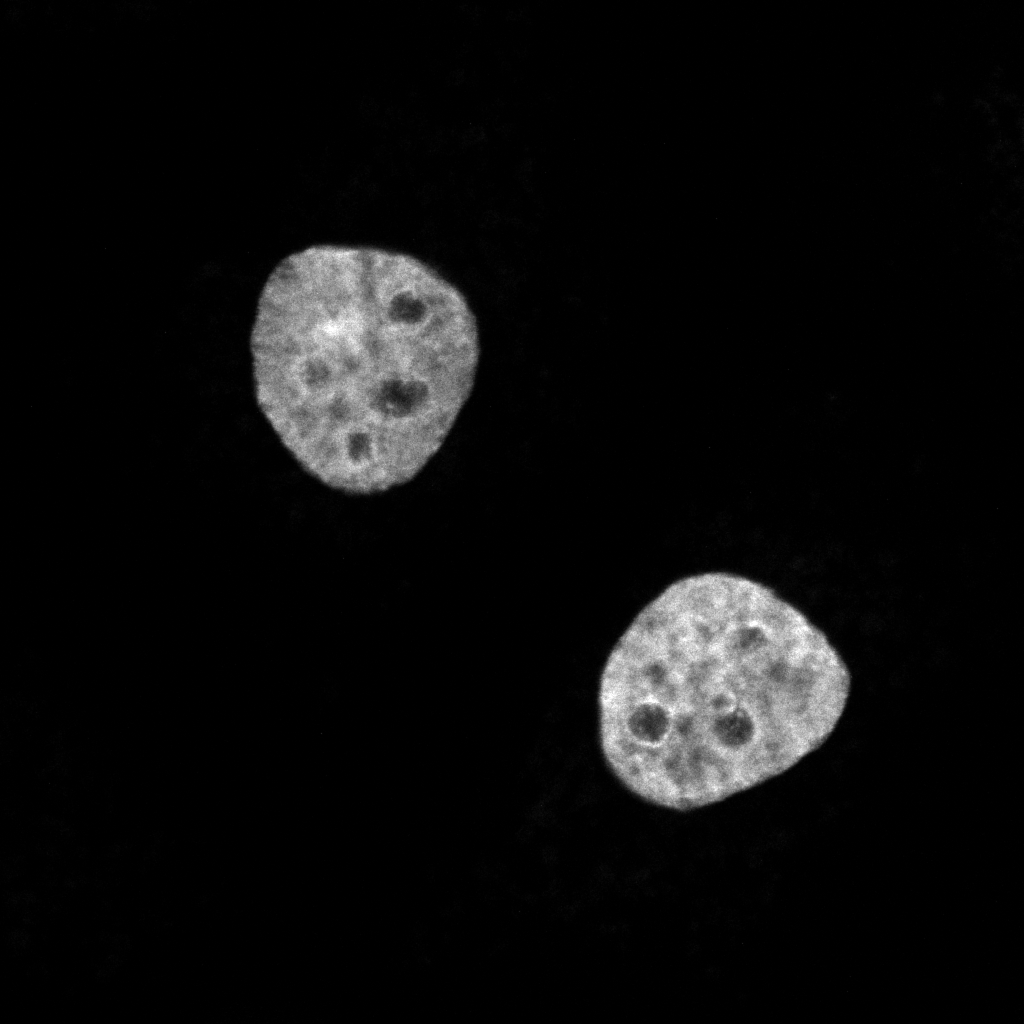

Supplement: Supplementary file 9 — Figure EV1-5 Source Data [file 44318_2025_672_MOESM9_ESM.zip › EV Source Data/EV4/EV4C/TECPR1KO_BAPTA LLOMe_DAPI.tif]

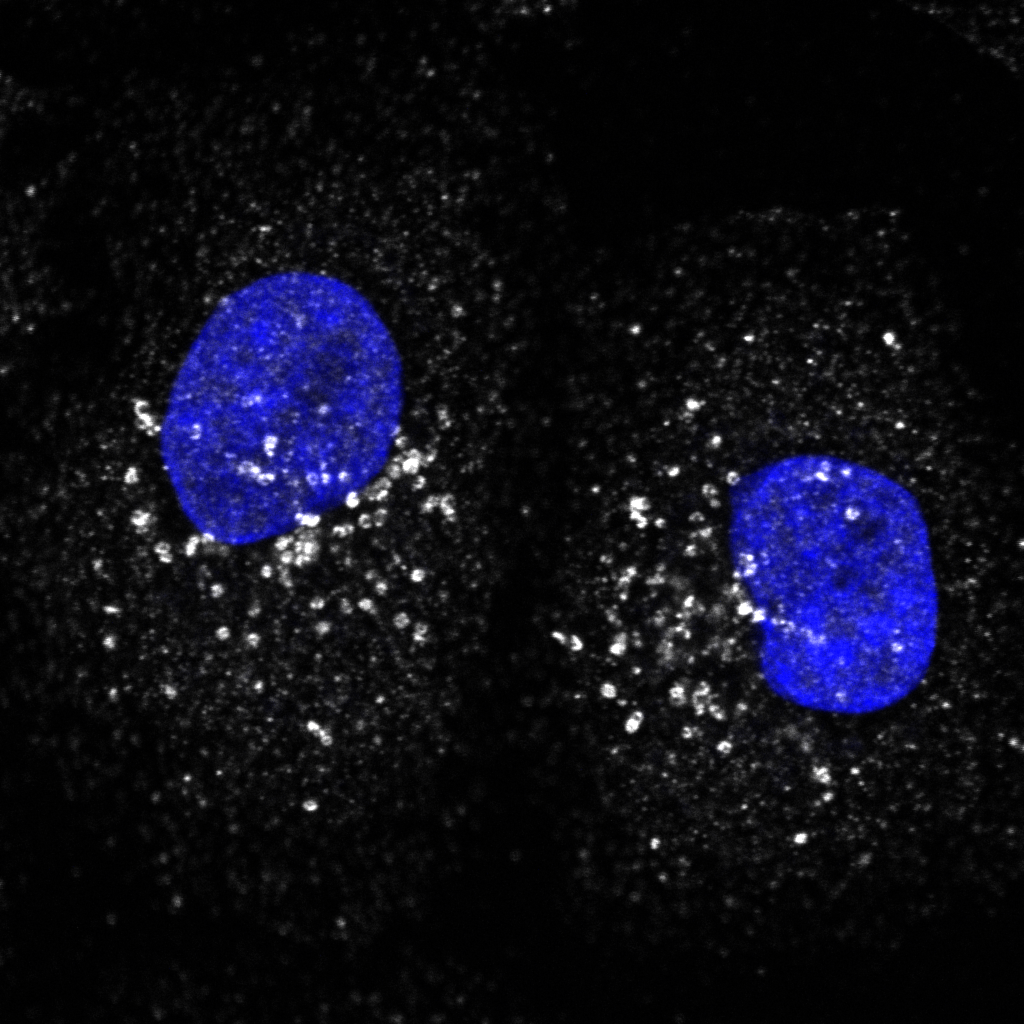

Supplement: Supplementary file 9 — Figure EV1-5 Source Data [file 44318_2025_672_MOESM9_ESM.zip › EV Source Data/EV4/EV4C/TECPR1KO_LLOMe.tif]

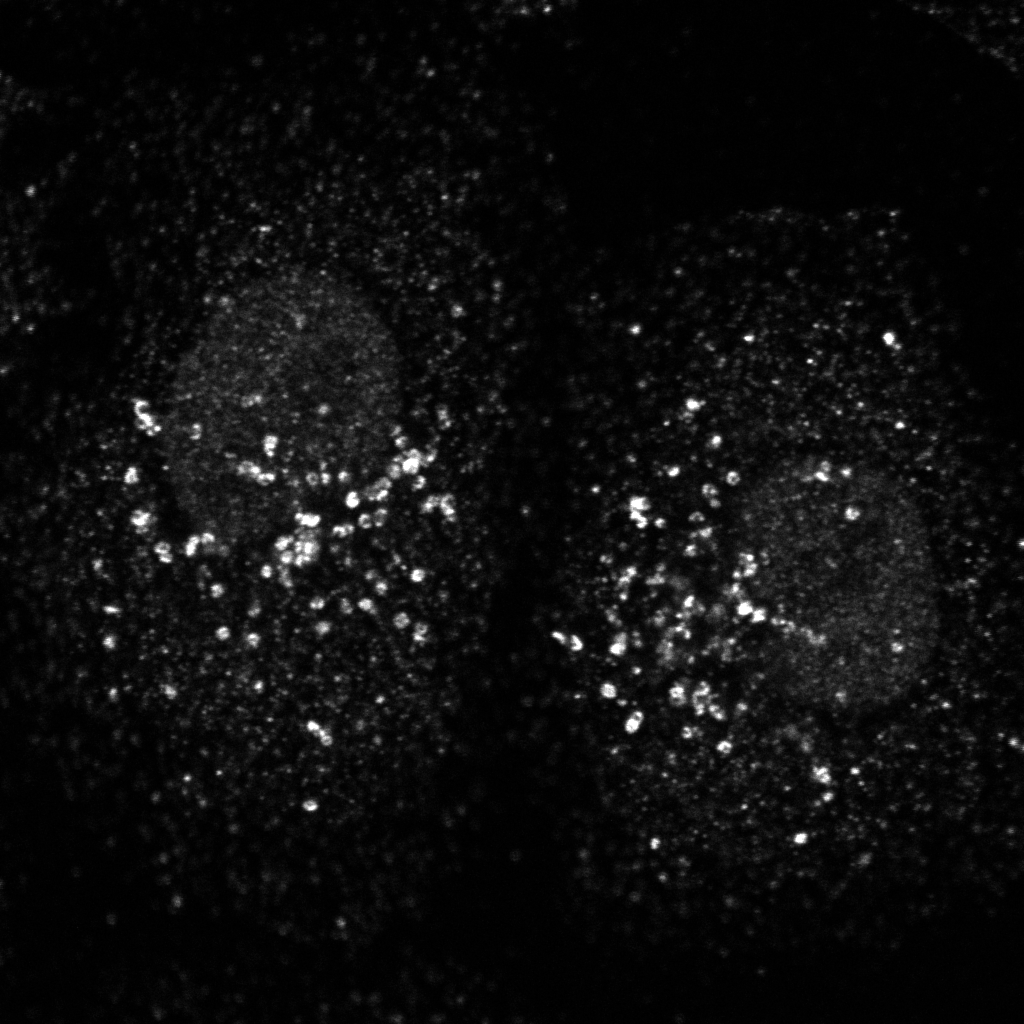

Supplement: Supplementary file 9 — Figure EV1-5 Source Data [file 44318_2025_672_MOESM9_ESM.zip › EV Source Data/EV4/EV4C/TECPR1KO_LLOMe_CHMP2A.tif]

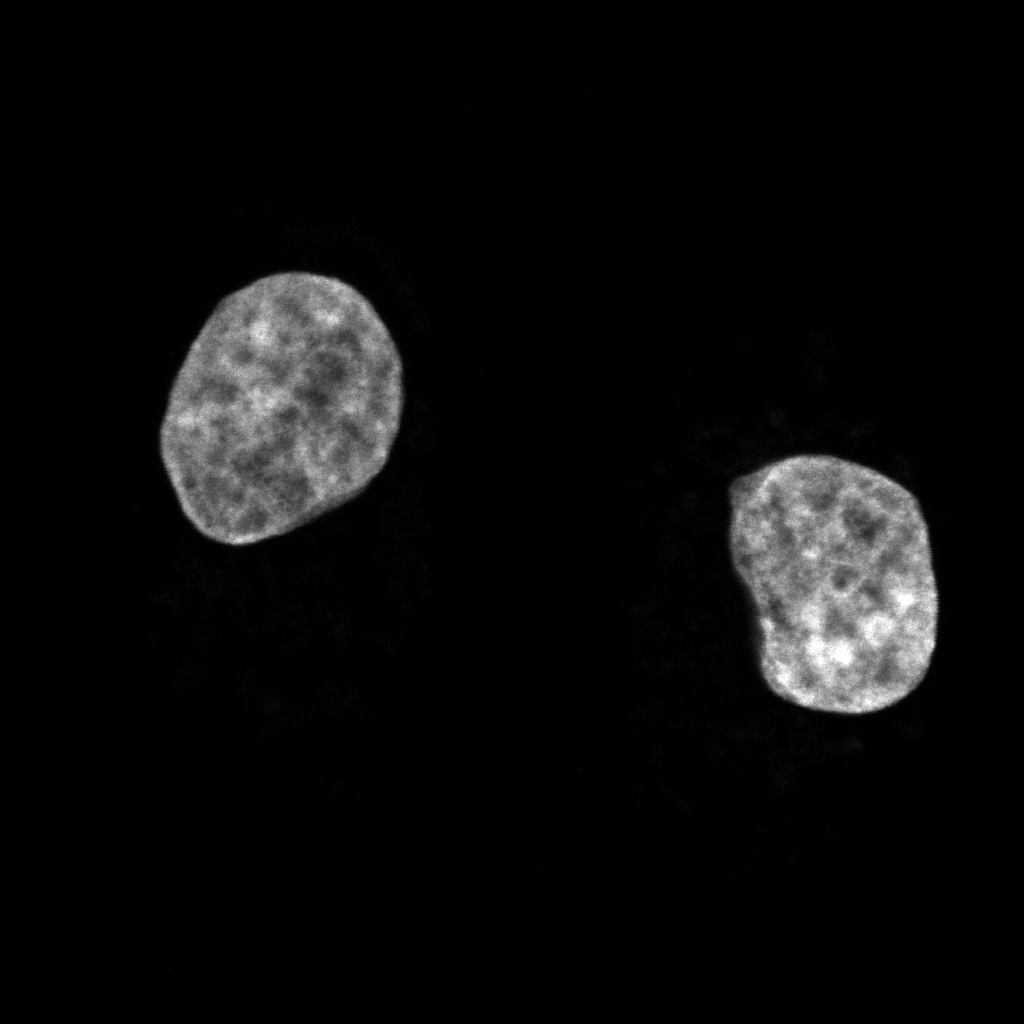

Supplement: Supplementary file 9 — Figure EV1-5 Source Data [file 44318_2025_672_MOESM9_ESM.zip › EV Source Data/EV4/EV4C/TECPR1KO_LLOMe_DAPI.tif]

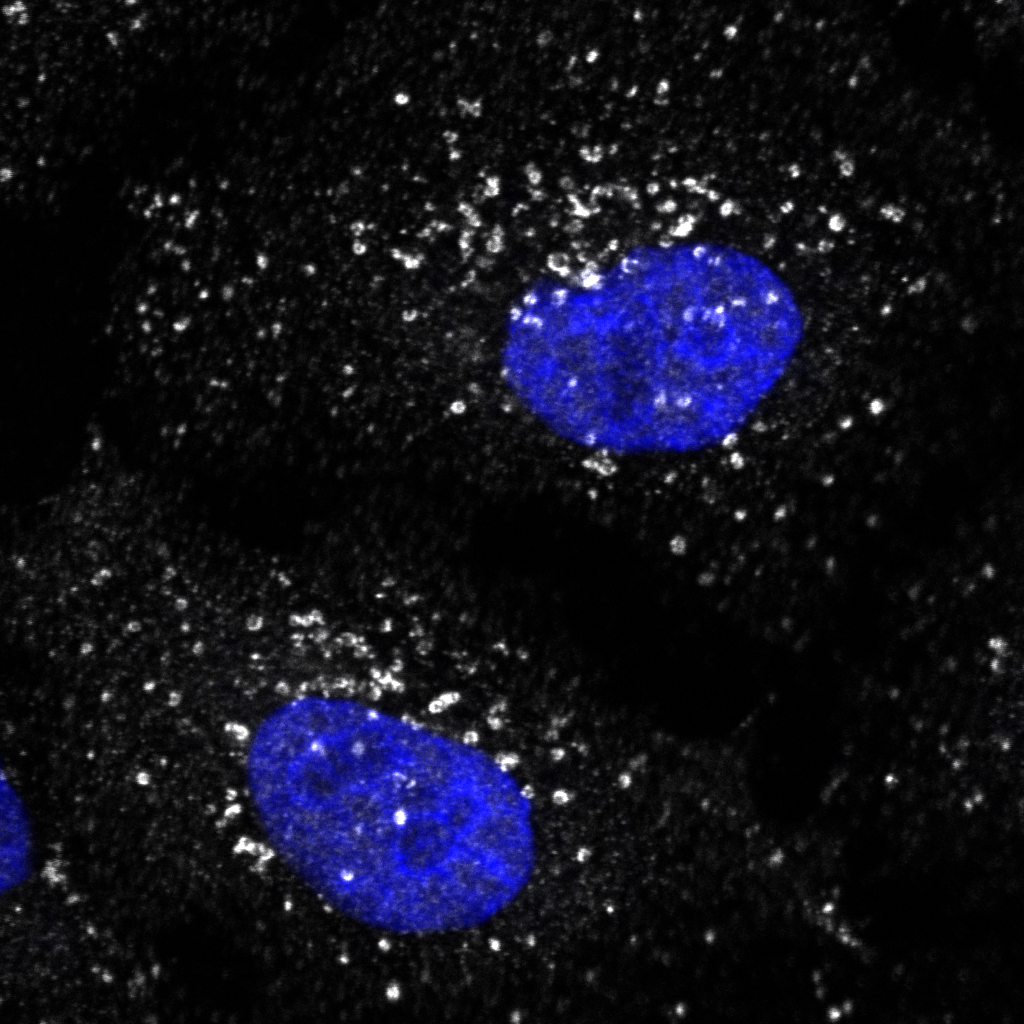

Supplement: Supplementary file 9 — Figure EV1-5 Source Data [file 44318_2025_672_MOESM9_ESM.zip › EV Source Data/EV4/EV4C/WT_BAPTA LLOMe.tif]

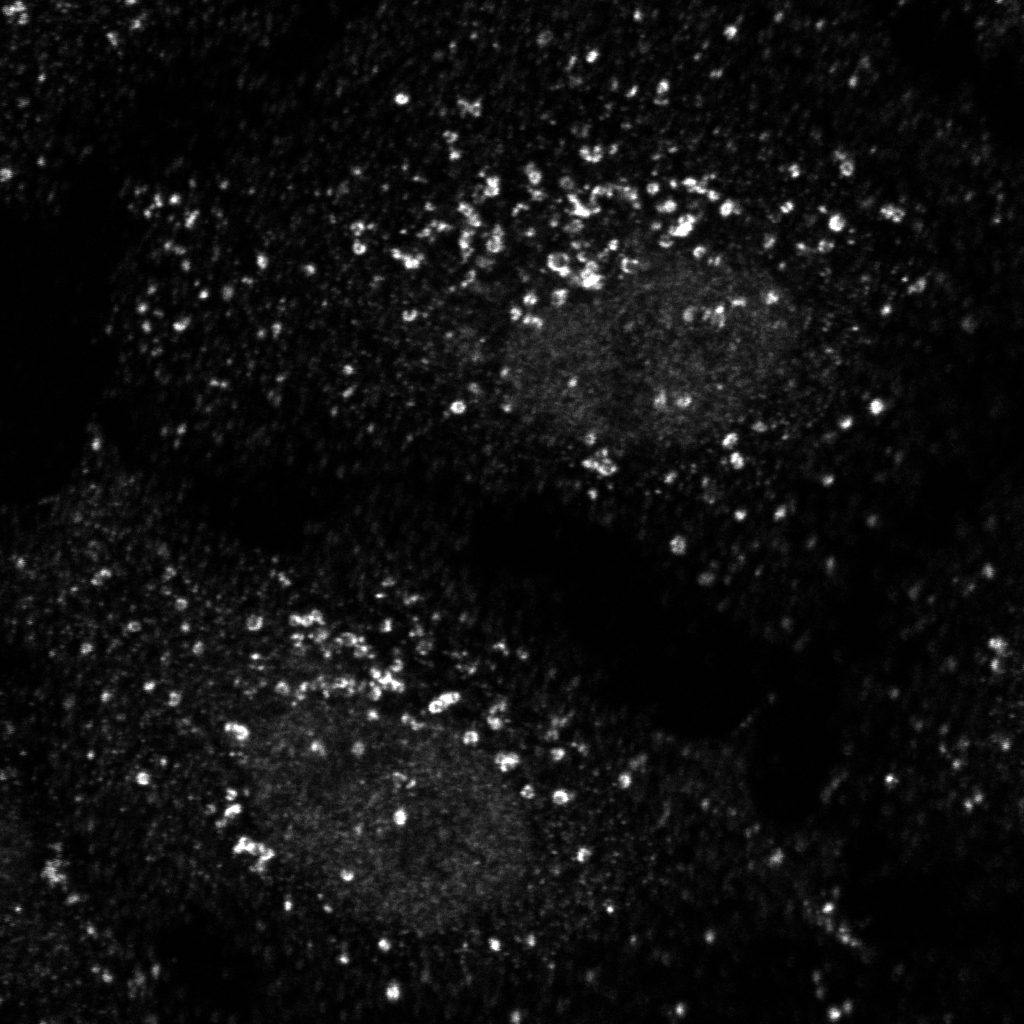

Supplement: Supplementary file 9 — Figure EV1-5 Source Data [file 44318_2025_672_MOESM9_ESM.zip › EV Source Data/EV4/EV4C/WT_BAPTA LLOMe_CHMP2A.tif]

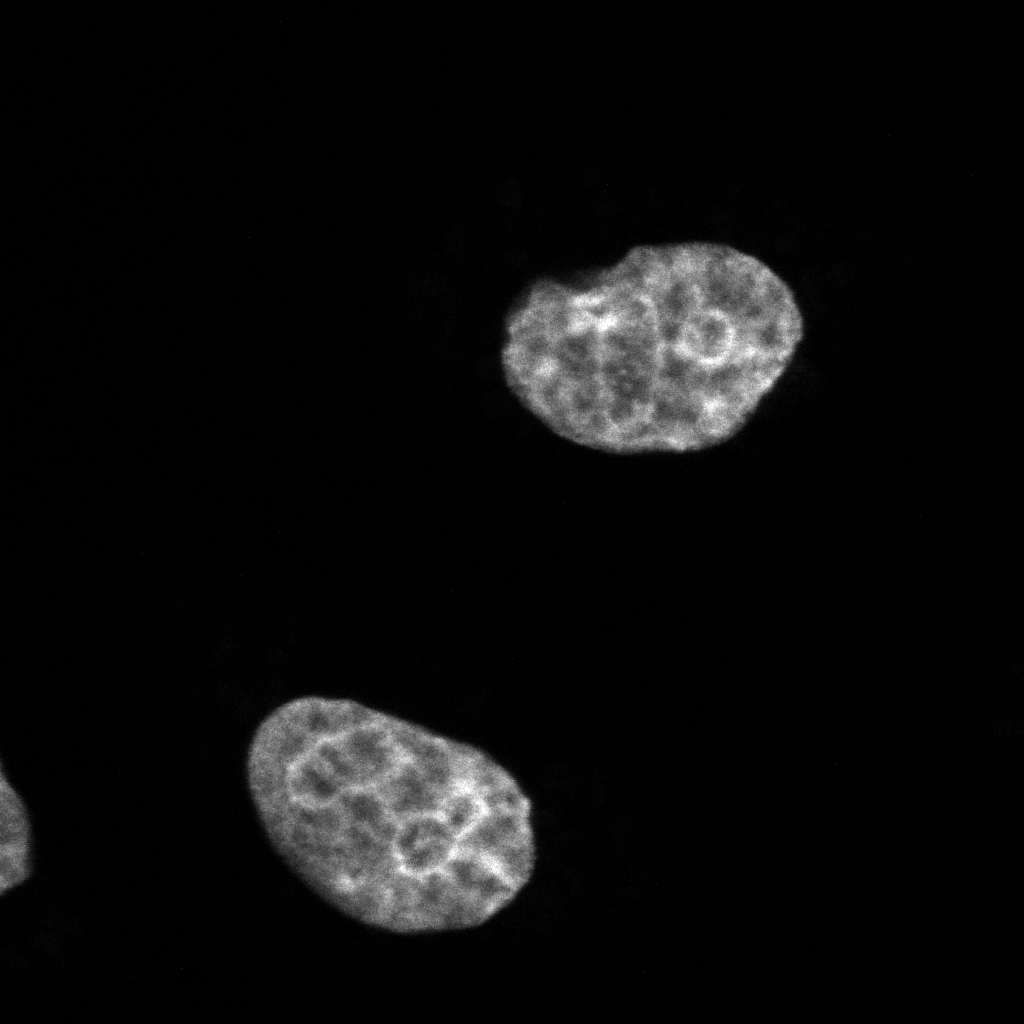

Supplement: Supplementary file 9 — Figure EV1-5 Source Data [file 44318_2025_672_MOESM9_ESM.zip › EV Source Data/EV4/EV4C/WT_BAPTA LLOMe_DAPI.tif]

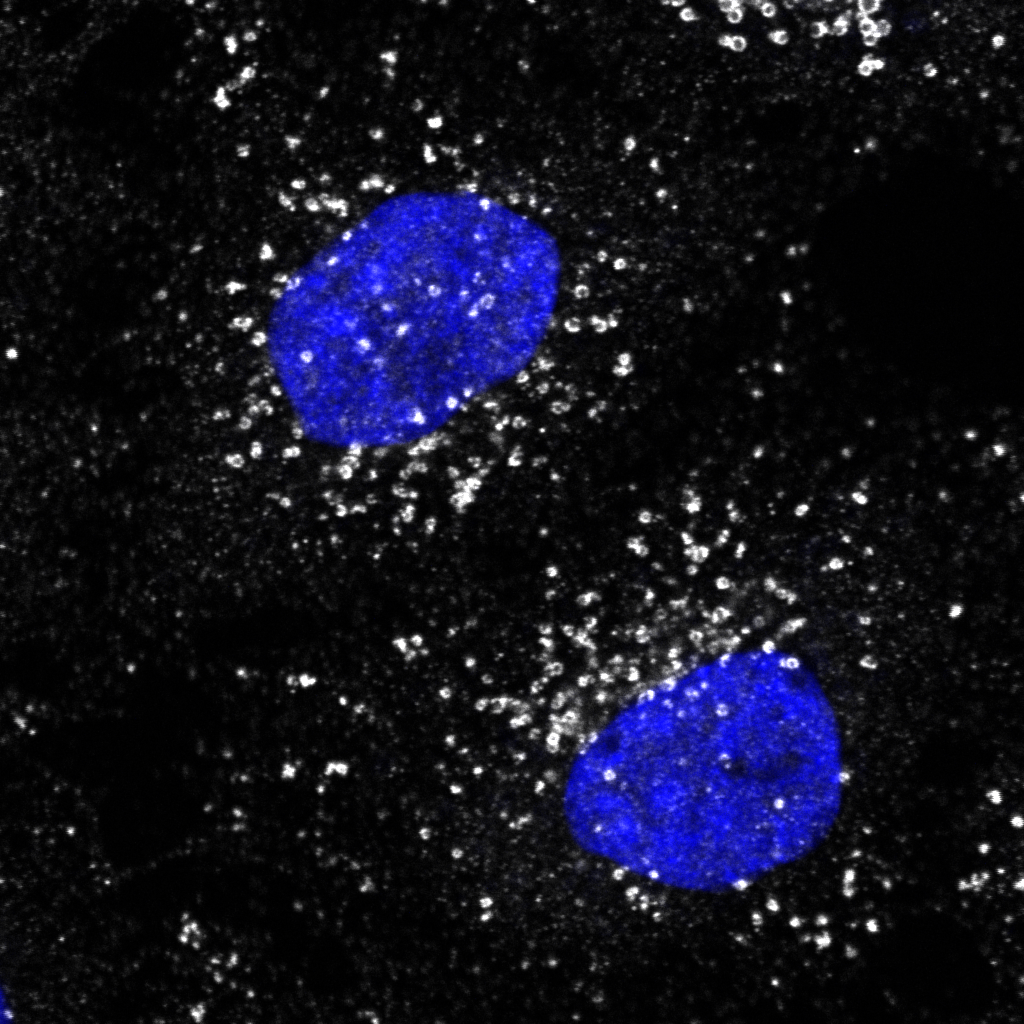

Supplement: Supplementary file 9 — Figure EV1-5 Source Data [file 44318_2025_672_MOESM9_ESM.zip › EV Source Data/EV4/EV4C/WT_LLOMe.tif]

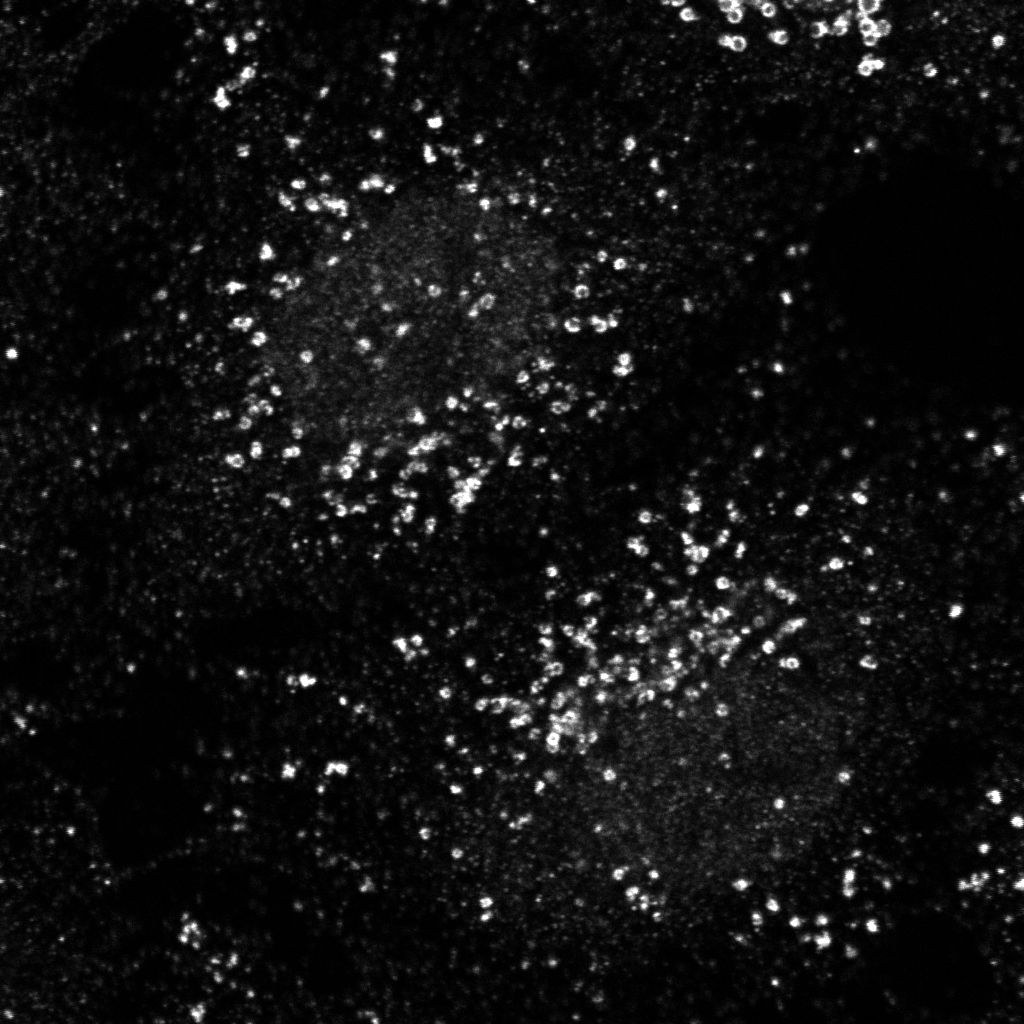

Supplement: Supplementary file 9 — Figure EV1-5 Source Data [file 44318_2025_672_MOESM9_ESM.zip › EV Source Data/EV4/EV4C/WT_LLOMe_CHMP2A.tif]

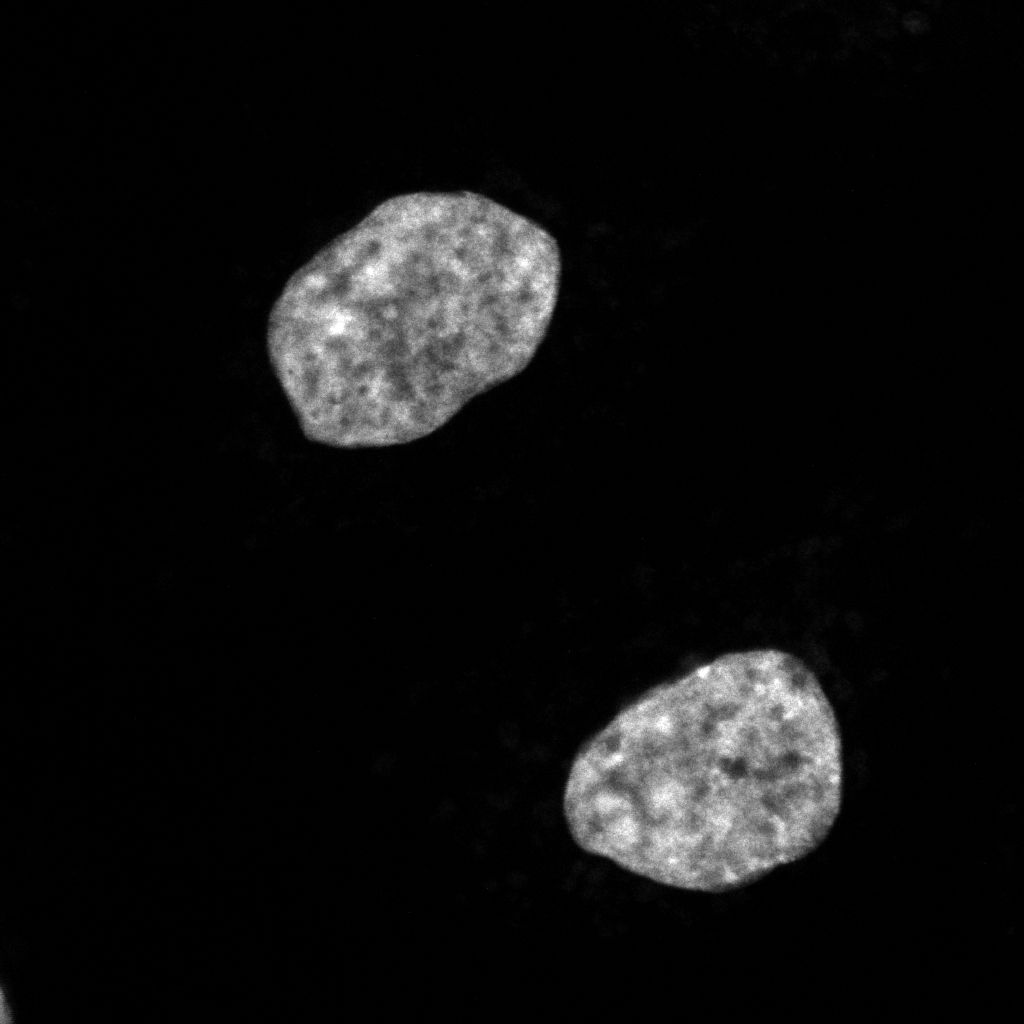

Supplement: Supplementary file 9 — Figure EV1-5 Source Data [file 44318_2025_672_MOESM9_ESM.zip › EV Source Data/EV4/EV4C/WT_LLOMe_DAPI.tif]

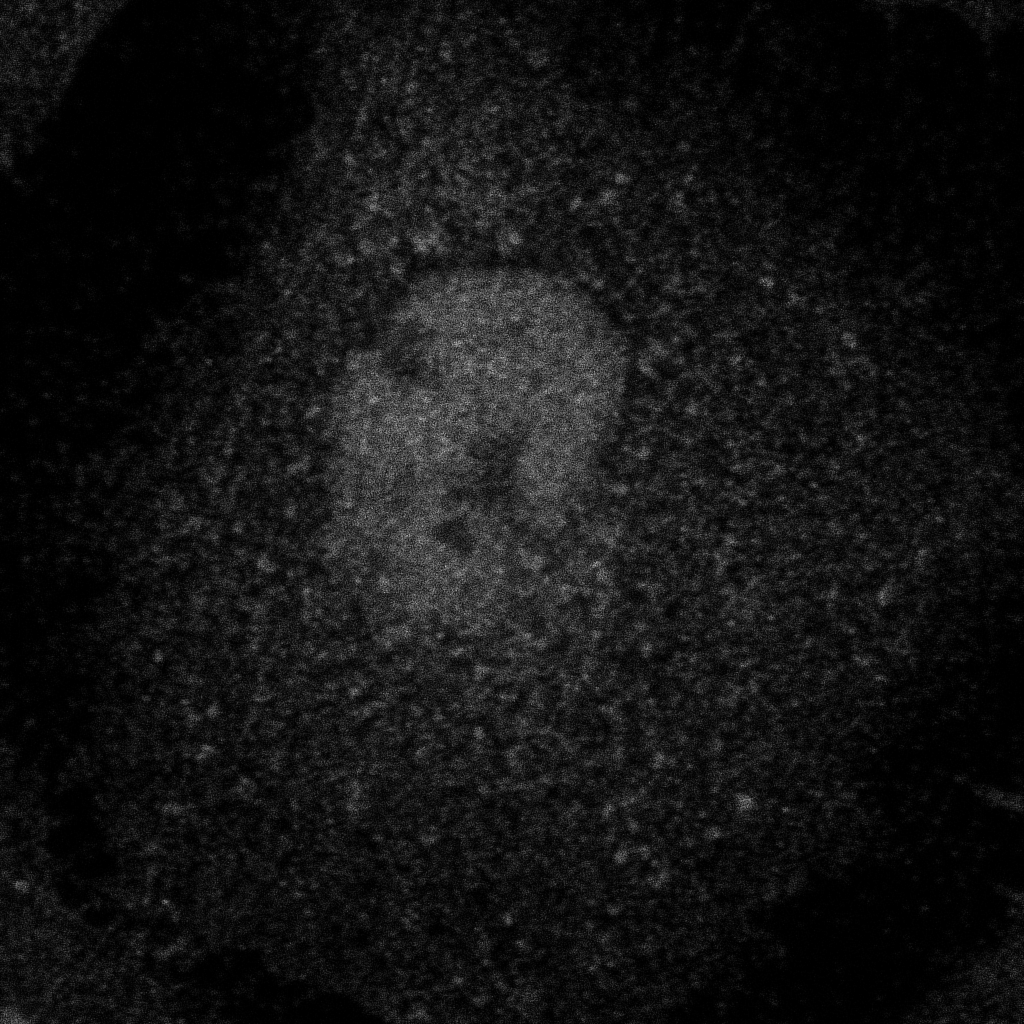

Supplement: Supplementary file 9 — Figure EV1-5 Source Data [file 44318_2025_672_MOESM9_ESM.zip › EV Source Data/EV5/EV5A/BAPTA_LLOMe_ALG2.tif]

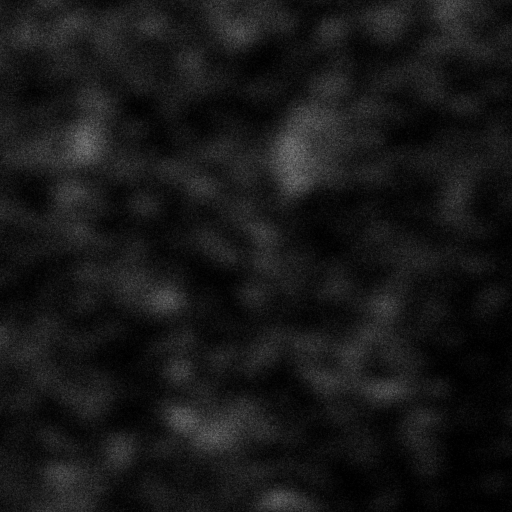

Supplement: Supplementary file 9 — Figure EV1-5 Source Data [file 44318_2025_672_MOESM9_ESM.zip › EV Source Data/EV5/EV5A/BAPTA_LLOMe_ALG2_zoom.tif]

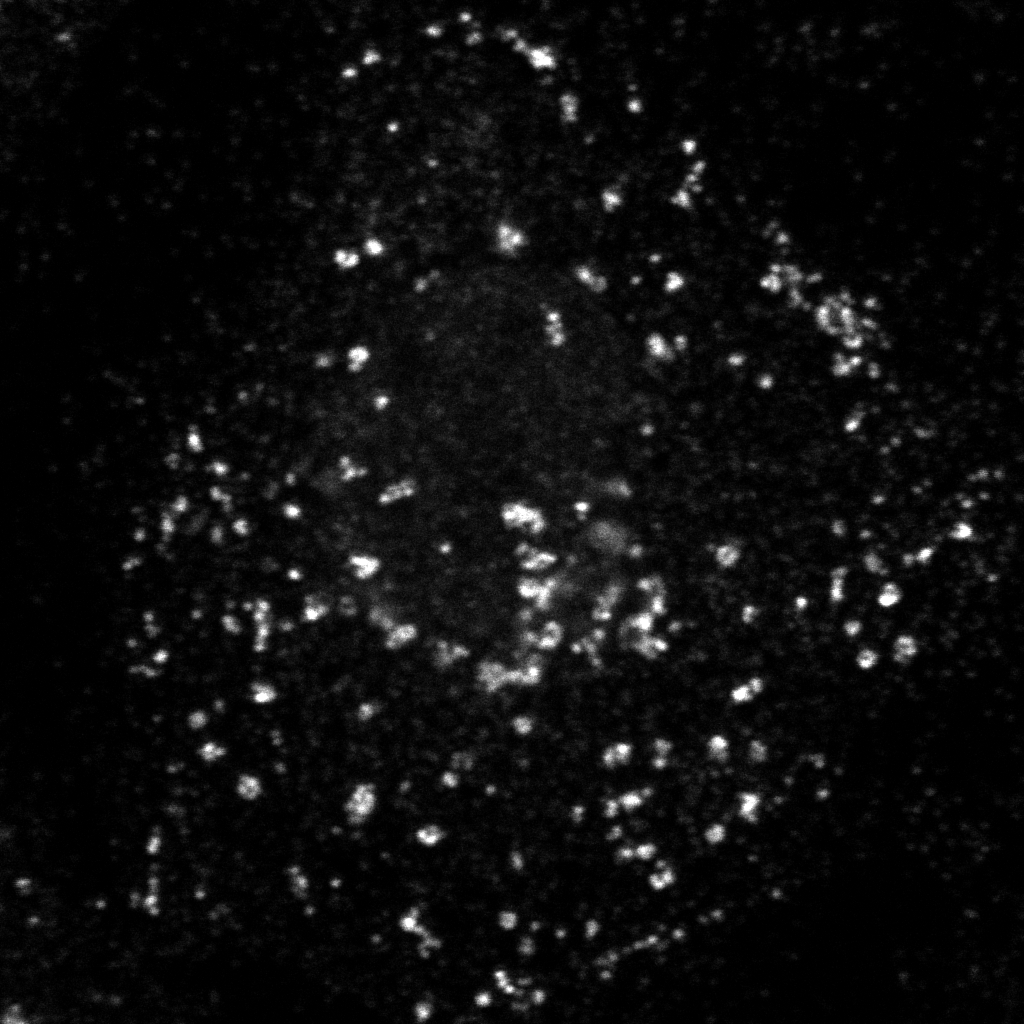

Supplement: Supplementary file 9 — Figure EV1-5 Source Data [file 44318_2025_672_MOESM9_ESM.zip › EV Source Data/EV5/EV5A/BAPTA_LLOMe_ALIX.tif]

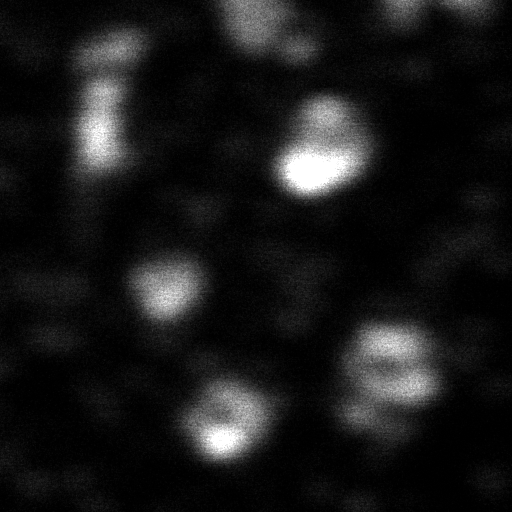

Supplement: Supplementary file 9 — Figure EV1-5 Source Data [file 44318_2025_672_MOESM9_ESM.zip › EV Source Data/EV5/EV5A/BAPTA_LLOMe_ALIX_zoom.tif]

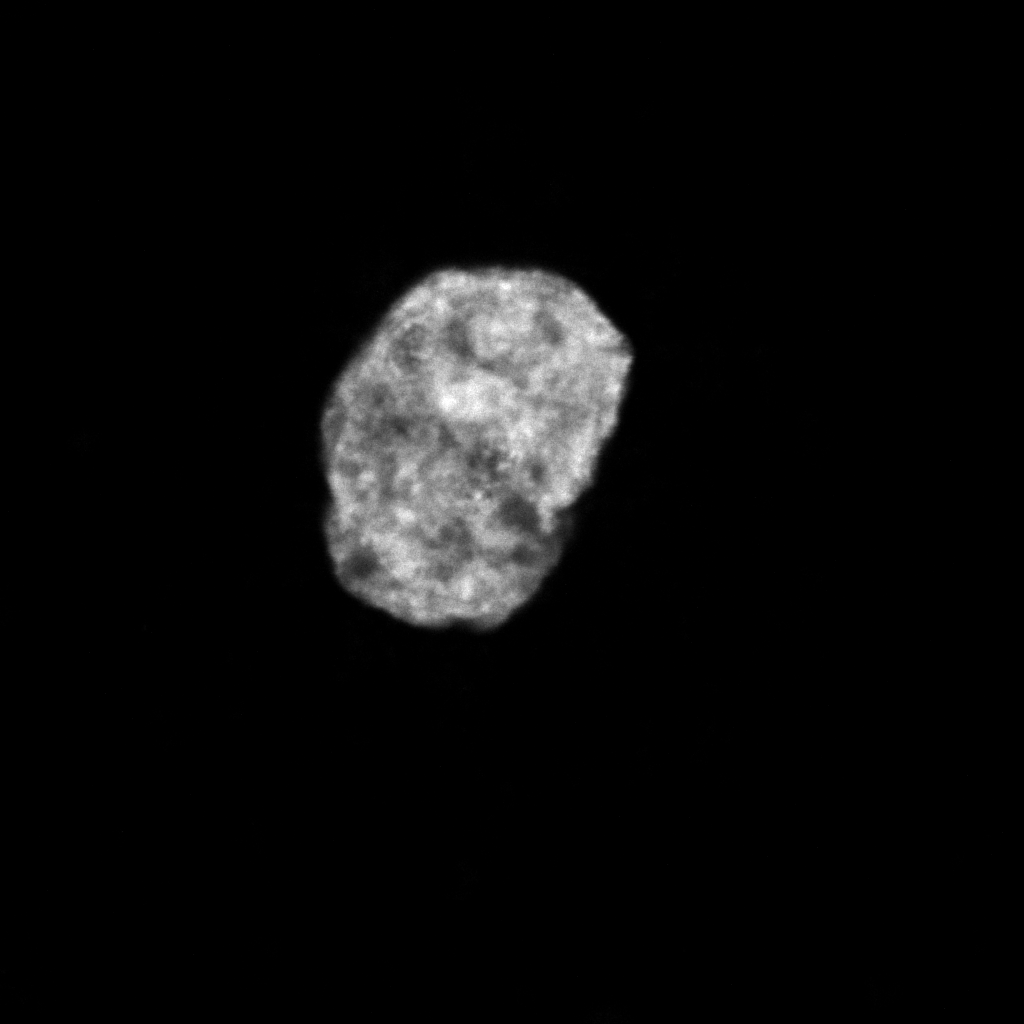

Supplement: Supplementary file 9 — Figure EV1-5 Source Data [file 44318_2025_672_MOESM9_ESM.zip › EV Source Data/EV5/EV5A/BAPTA_LLOMe_DAPI.tif]

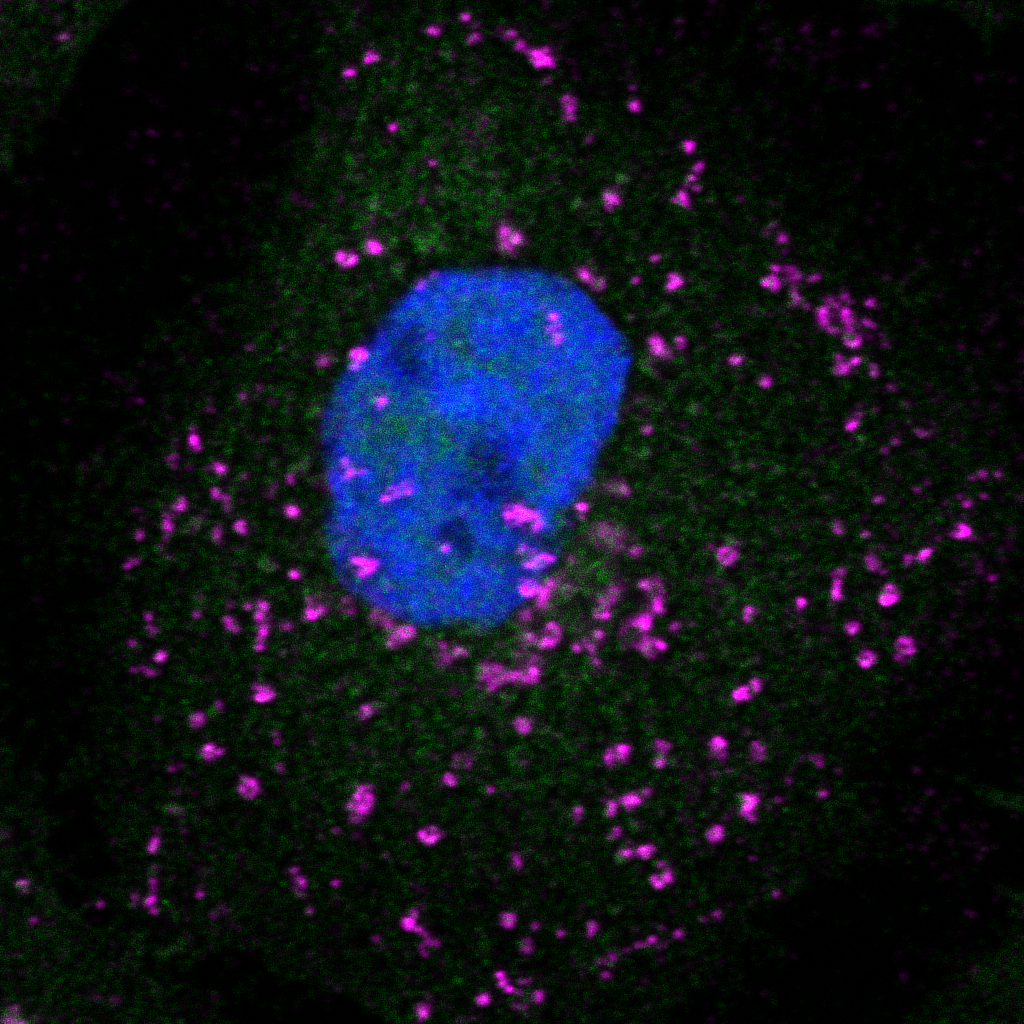

Supplement: Supplementary file 9 — Figure EV1-5 Source Data [file 44318_2025_672_MOESM9_ESM.zip › EV Source Data/EV5/EV5A/BAPTA_LLOMe_merge.tif]

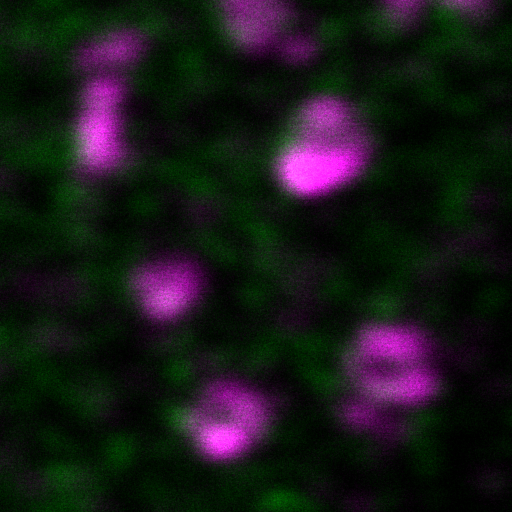

Supplement: Supplementary file 9 — Figure EV1-5 Source Data [file 44318_2025_672_MOESM9_ESM.zip › EV Source Data/EV5/EV5A/BAPTA_LLOMe_merge_zoom.tif]

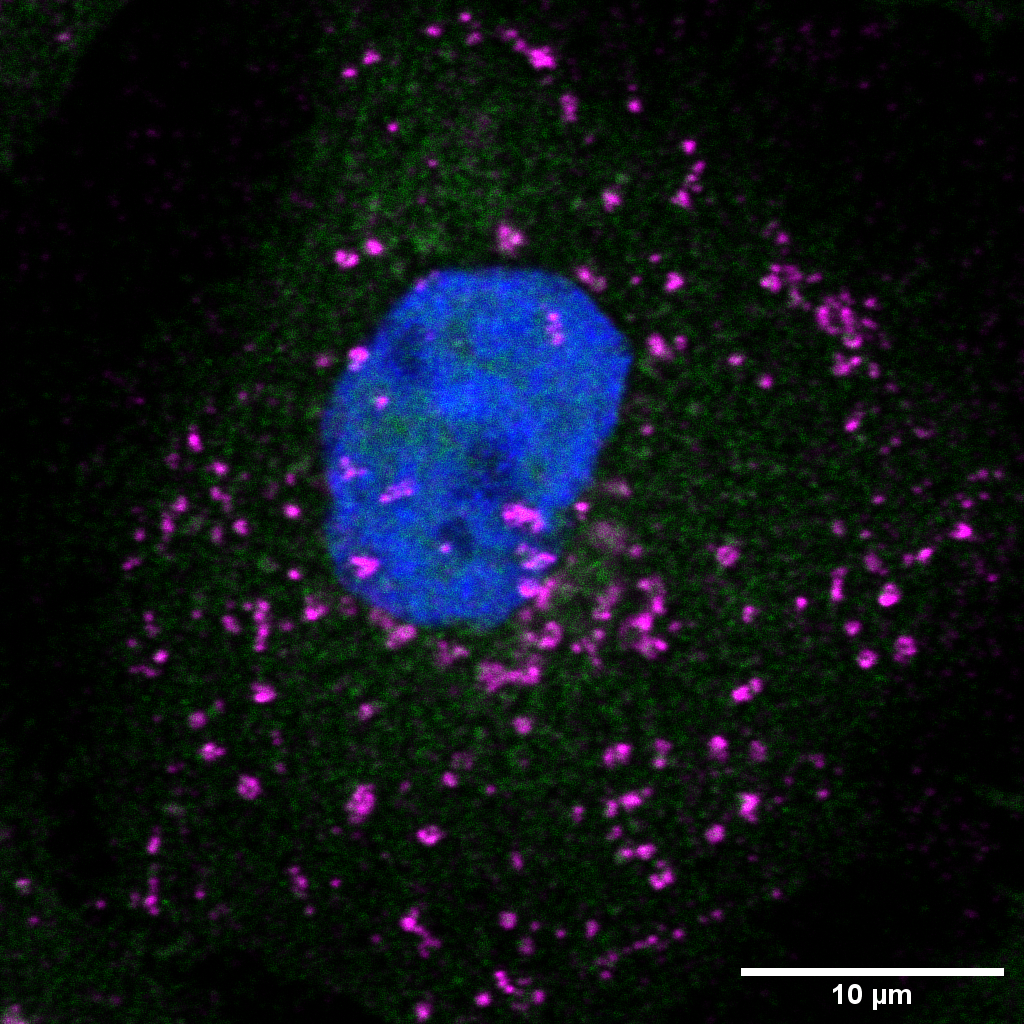

Supplement: Supplementary file 9 — Figure EV1-5 Source Data [file 44318_2025_672_MOESM9_ESM.zip › EV Source Data/EV5/EV5A/BAPTA_LLOMe_scale.tif]

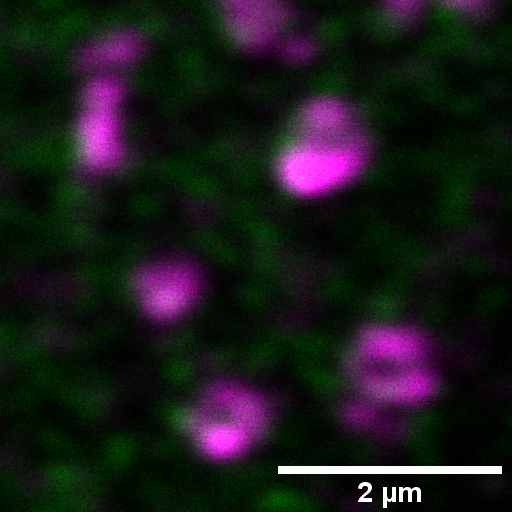

Supplement: Supplementary file 9 — Figure EV1-5 Source Data [file 44318_2025_672_MOESM9_ESM.zip › EV Source Data/EV5/EV5A/BAPTA_LLOMe_scale_zoom.tif]

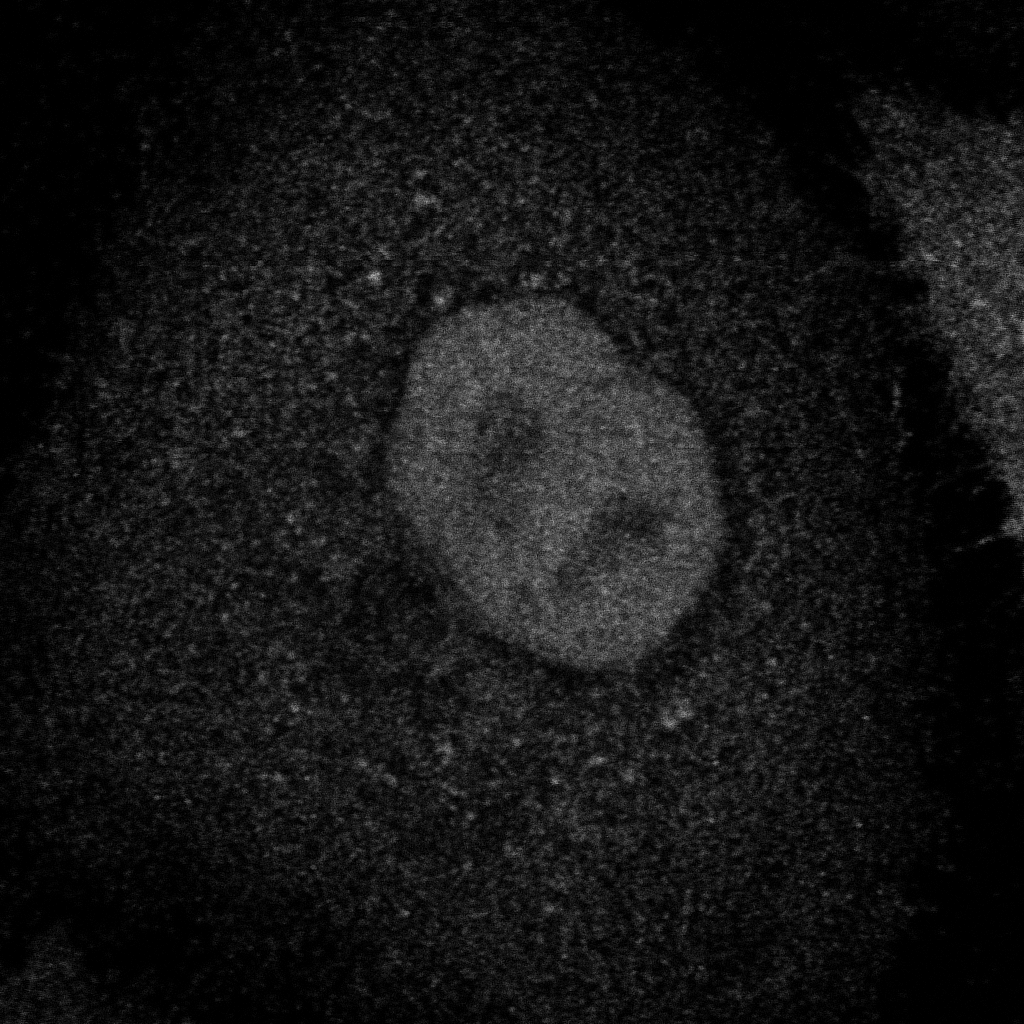

Supplement: Supplementary file 9 — Figure EV1-5 Source Data [file 44318_2025_672_MOESM9_ESM.zip › EV Source Data/EV5/EV5A/BAPTA_VEH_ALG2.tif]

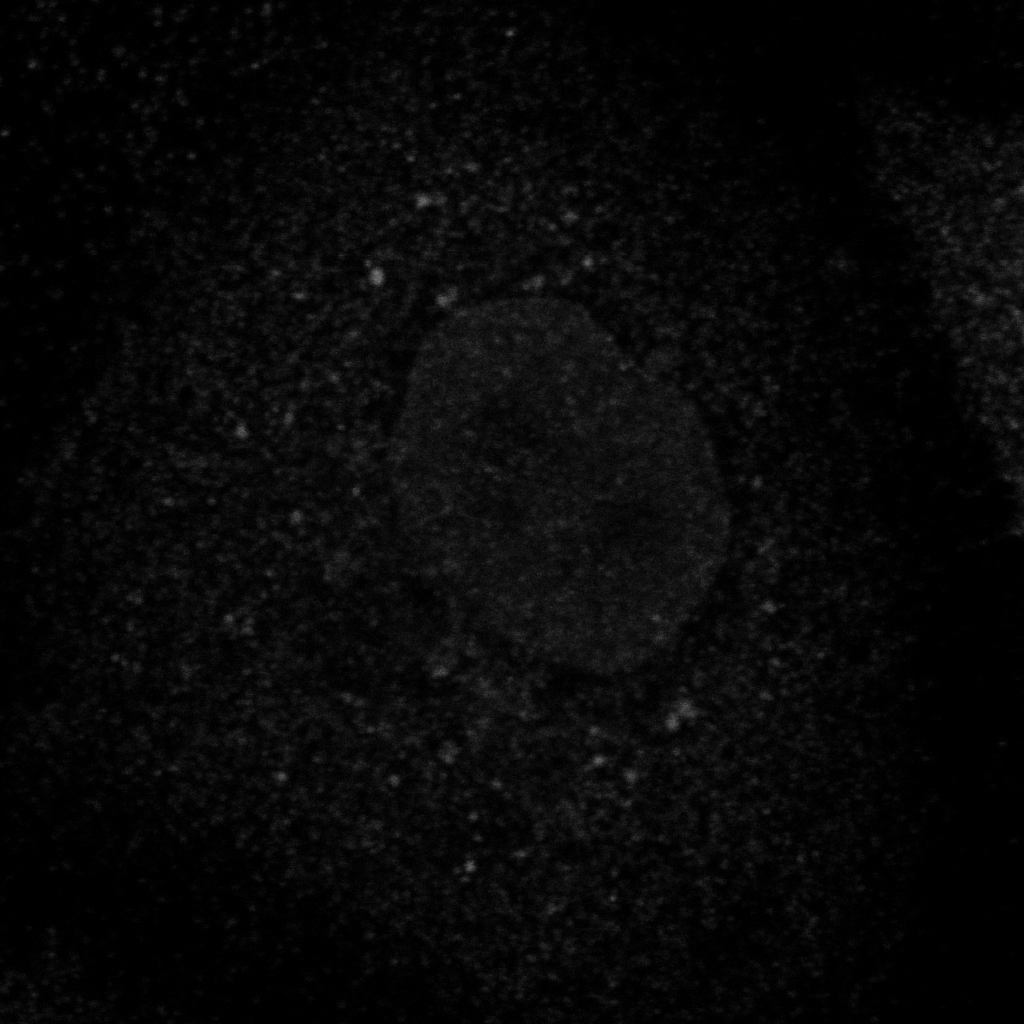

Supplement: Supplementary file 9 — Figure EV1-5 Source Data [file 44318_2025_672_MOESM9_ESM.zip › EV Source Data/EV5/EV5A/BAPTA_VEH_ALIX.tif]

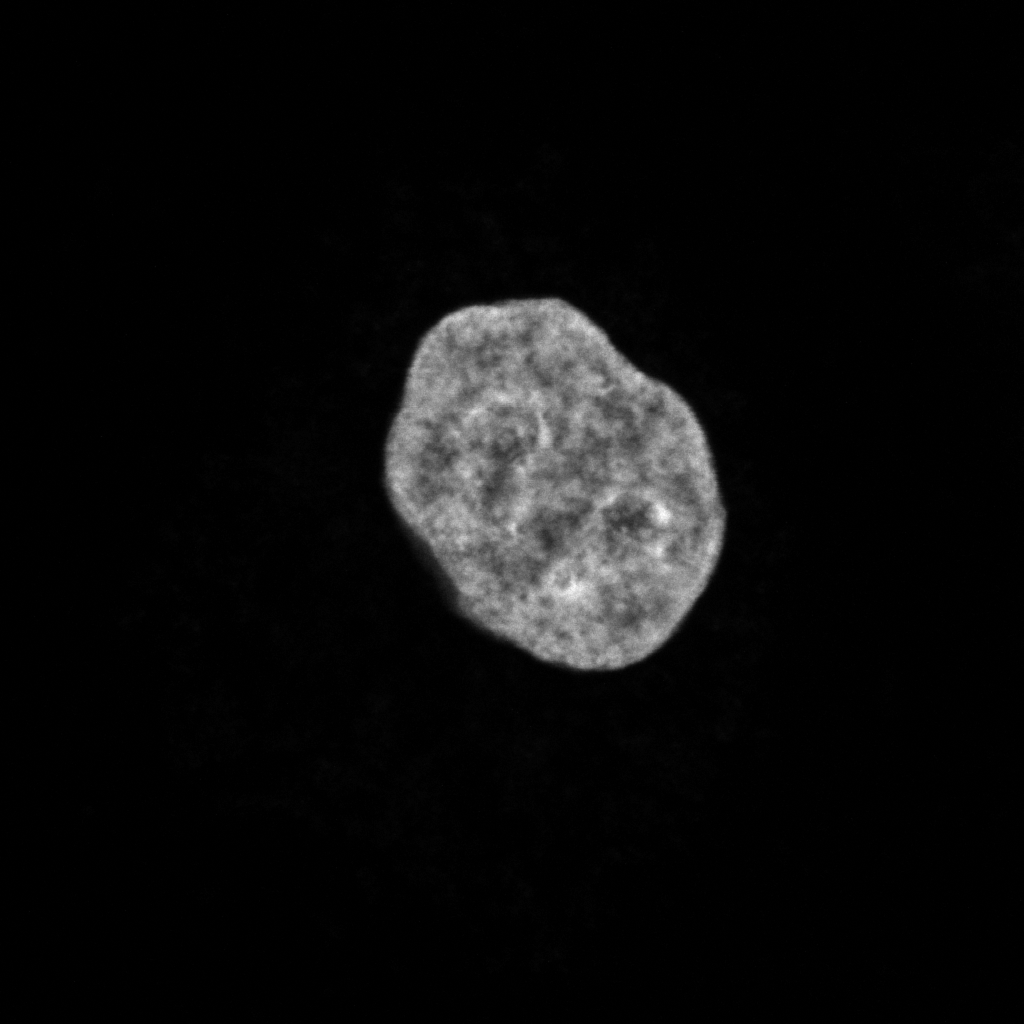

Supplement: Supplementary file 9 — Figure EV1-5 Source Data [file 44318_2025_672_MOESM9_ESM.zip › EV Source Data/EV5/EV5A/BAPTA_VEH_DAPI.tif]

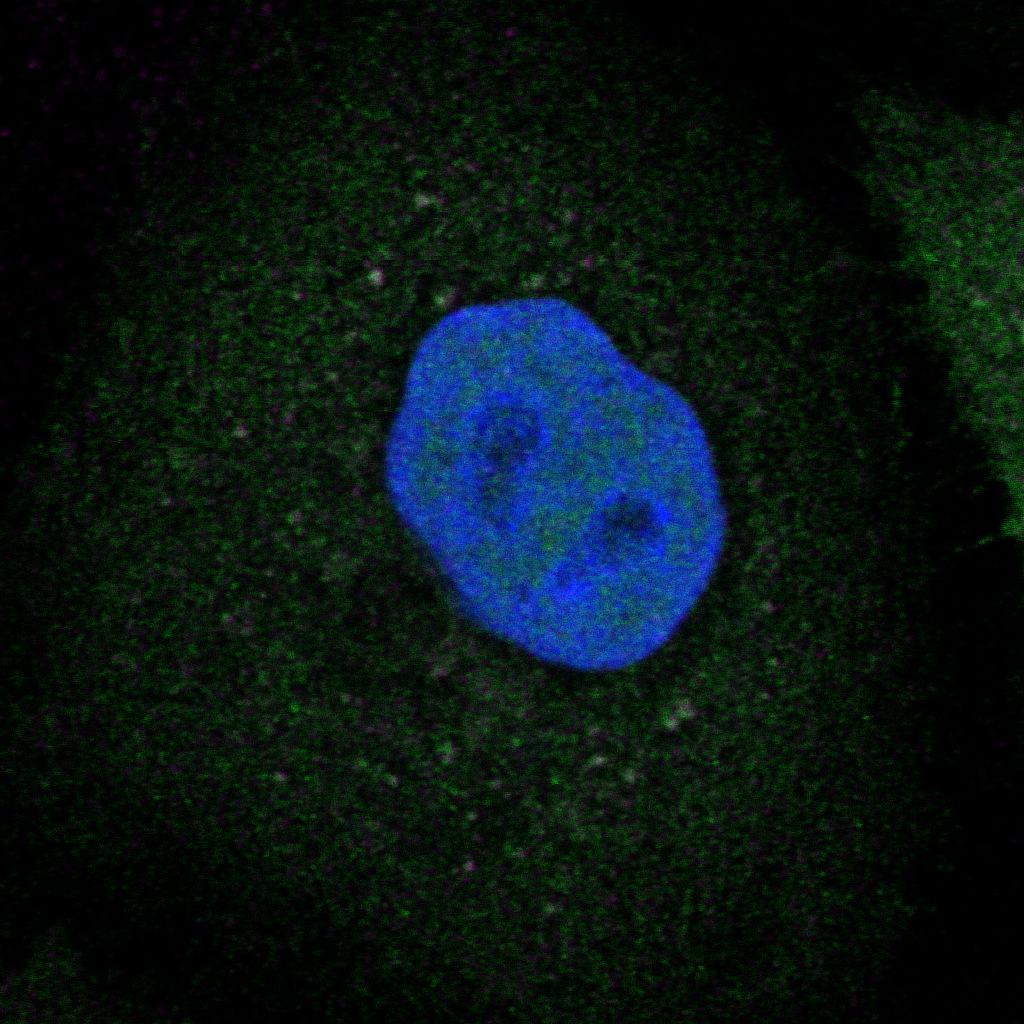

Supplement: Supplementary file 9 — Figure EV1-5 Source Data [file 44318_2025_672_MOESM9_ESM.zip › EV Source Data/EV5/EV5A/BAPTA_VEH_merge.tif]

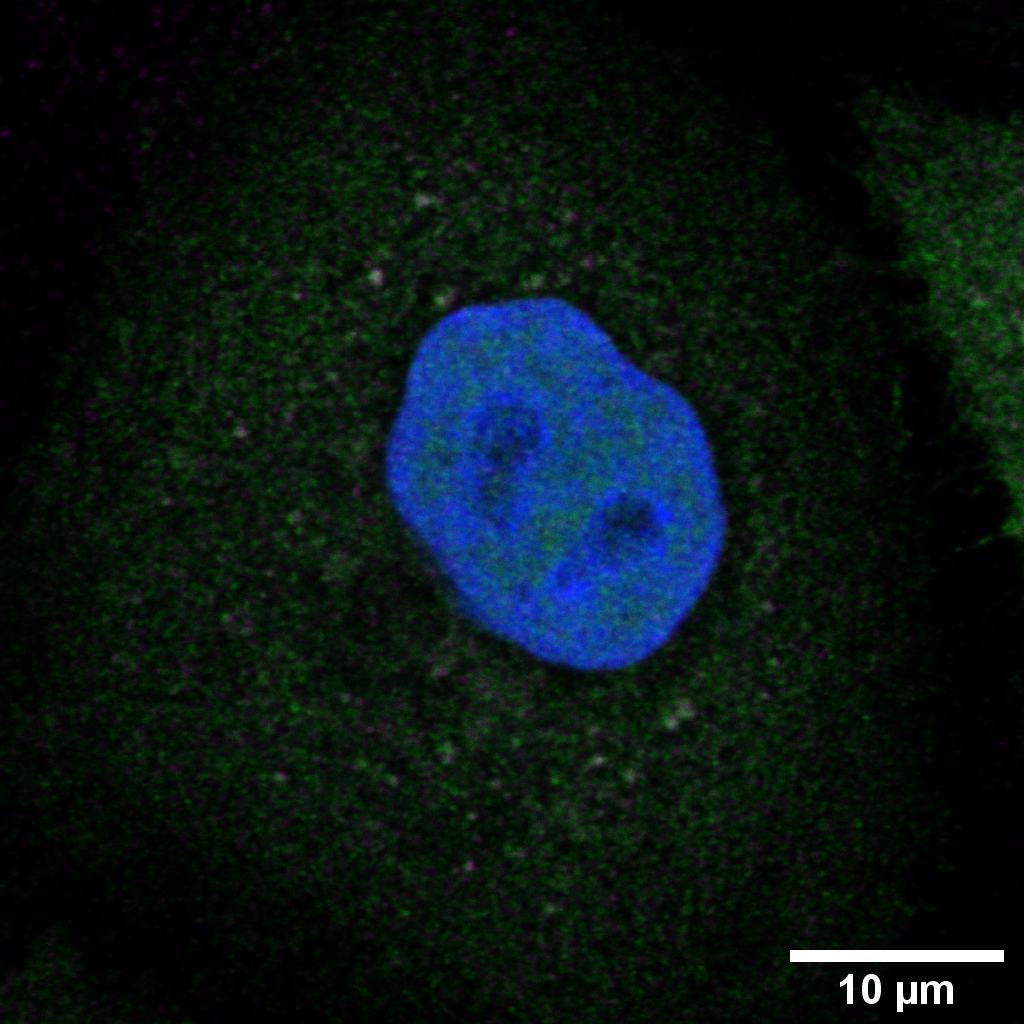

Supplement: Supplementary file 9 — Figure EV1-5 Source Data [file 44318_2025_672_MOESM9_ESM.zip › EV Source Data/EV5/EV5A/BAPTA_VEH_scale.tif]

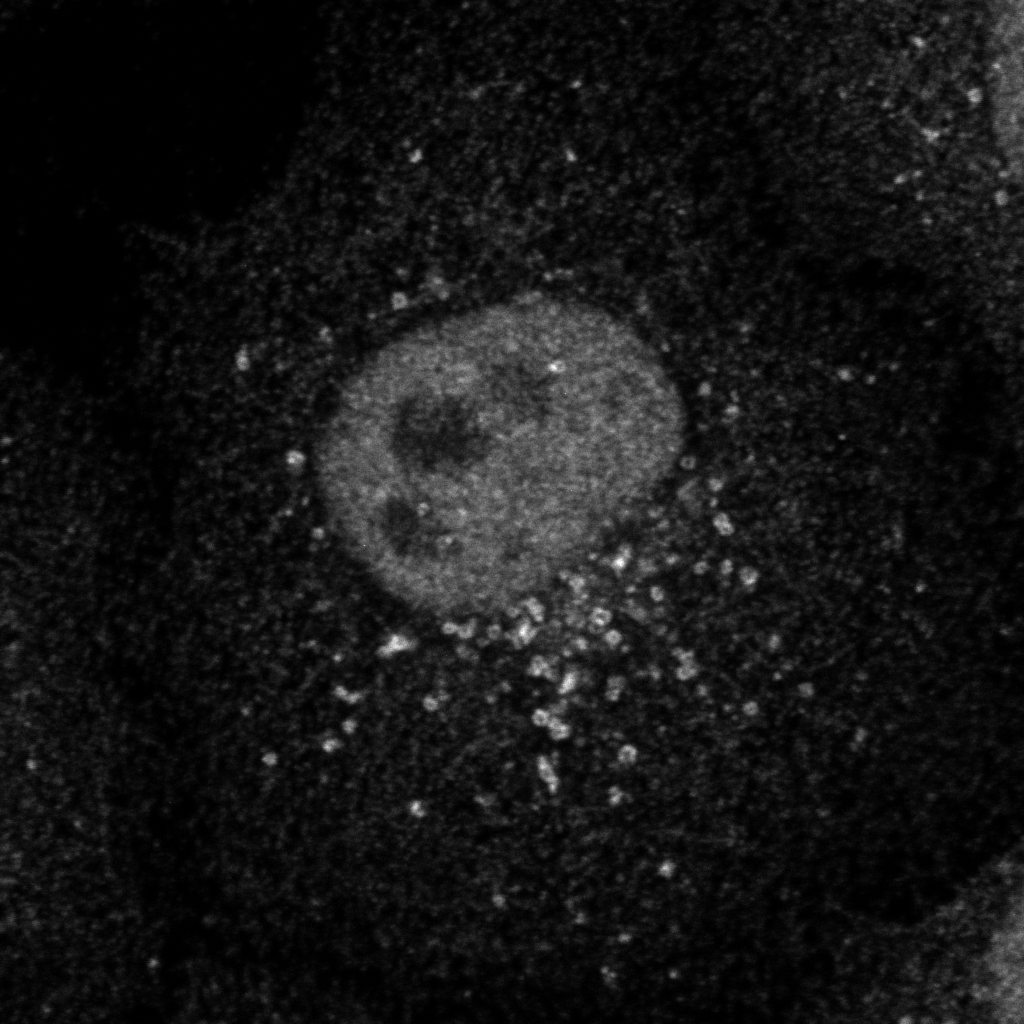

Supplement: Supplementary file 9 — Figure EV1-5 Source Data [file 44318_2025_672_MOESM9_ESM.zip › EV Source Data/EV5/EV5A/LLOMe_ALG2.tif]

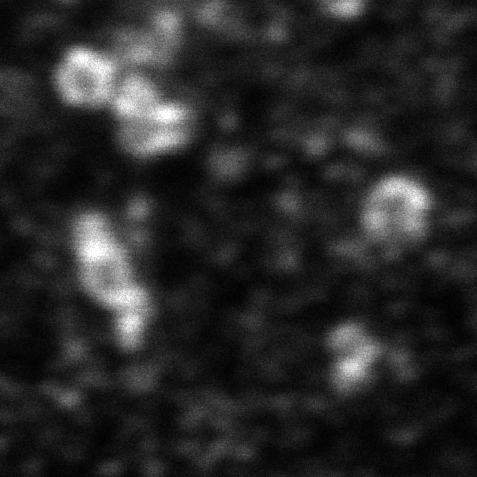

Supplement: Supplementary file 9 — Figure EV1-5 Source Data [file 44318_2025_672_MOESM9_ESM.zip › EV Source Data/EV5/EV5A/LLOMe_ALG2_zoom.tif]
