# Supplementary figures and images for: The ATG8 E3-like ligases sense lysosomal damage and initiate ESCRT-mediated membrane repair (part 6 of 7)
Source: EMBO J. 2026 Jan 3;45(3):930–52. doi: 10.1038/s44318-025-00672-1 (PMC12865045; doi:10.1038/s44318-025-00672-1)

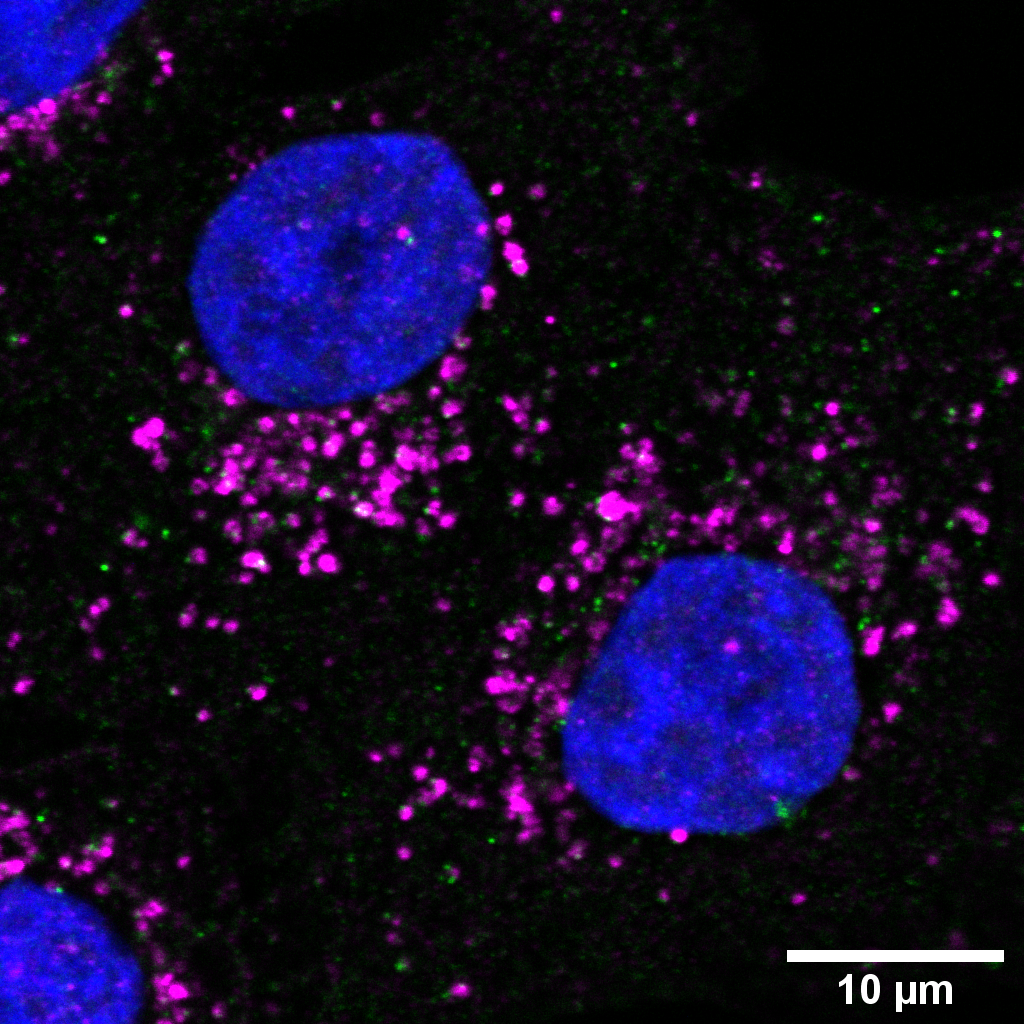

Supplement: Supplementary file 9 — Figure EV1-5 Source Data [file 44318_2025_672_MOESM9_ESM.zip › EV Source Data/EV1/EV1B/DKO3_scale.tif]

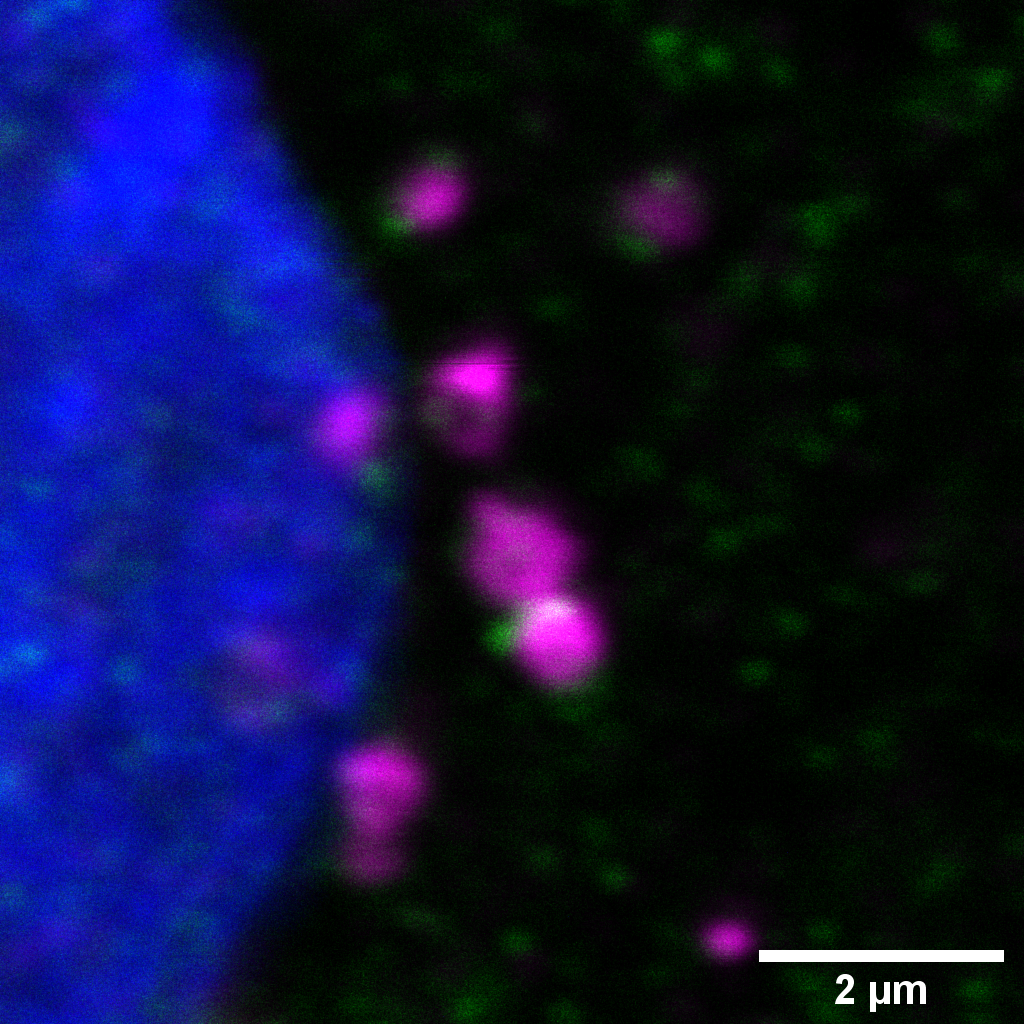

Supplement: Supplementary file 9 — Figure EV1-5 Source Data [file 44318_2025_672_MOESM9_ESM.zip › EV Source Data/EV1/EV1B/DKO3_scale_zoom.tif]

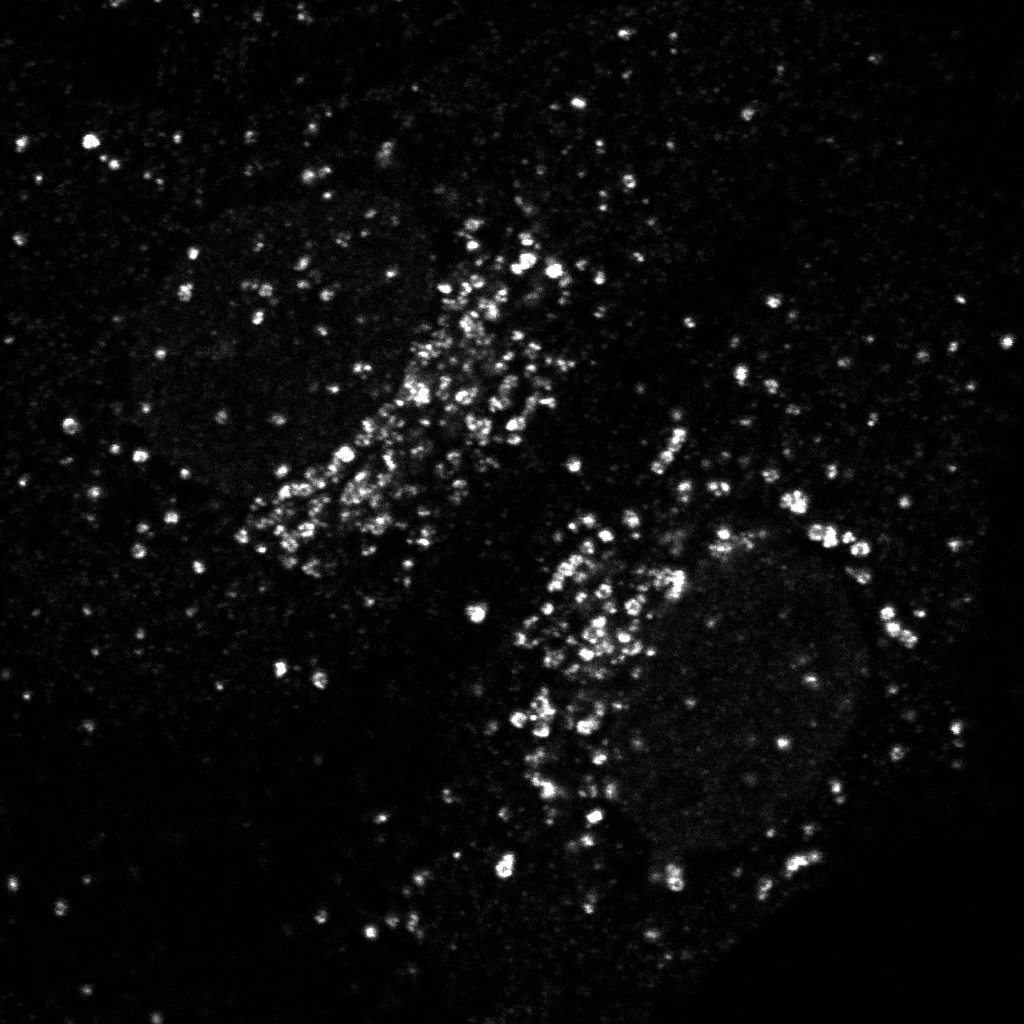

Supplement: Supplementary file 9 — Figure EV1-5 Source Data [file 44318_2025_672_MOESM9_ESM.zip › EV Source Data/EV1/EV1B/WT_ALIX.tif]

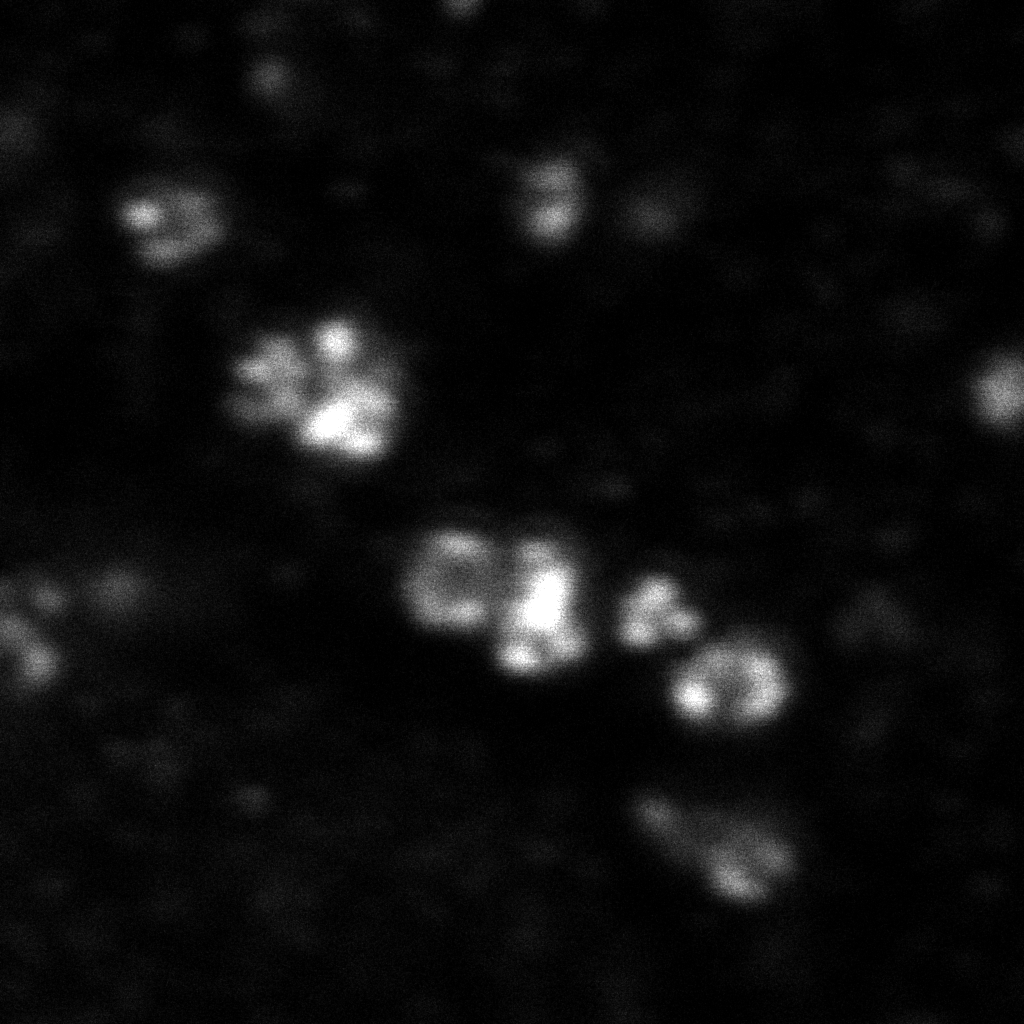

Supplement: Supplementary file 9 — Figure EV1-5 Source Data [file 44318_2025_672_MOESM9_ESM.zip › EV Source Data/EV1/EV1B/WT_ALIX_zoom.tif]

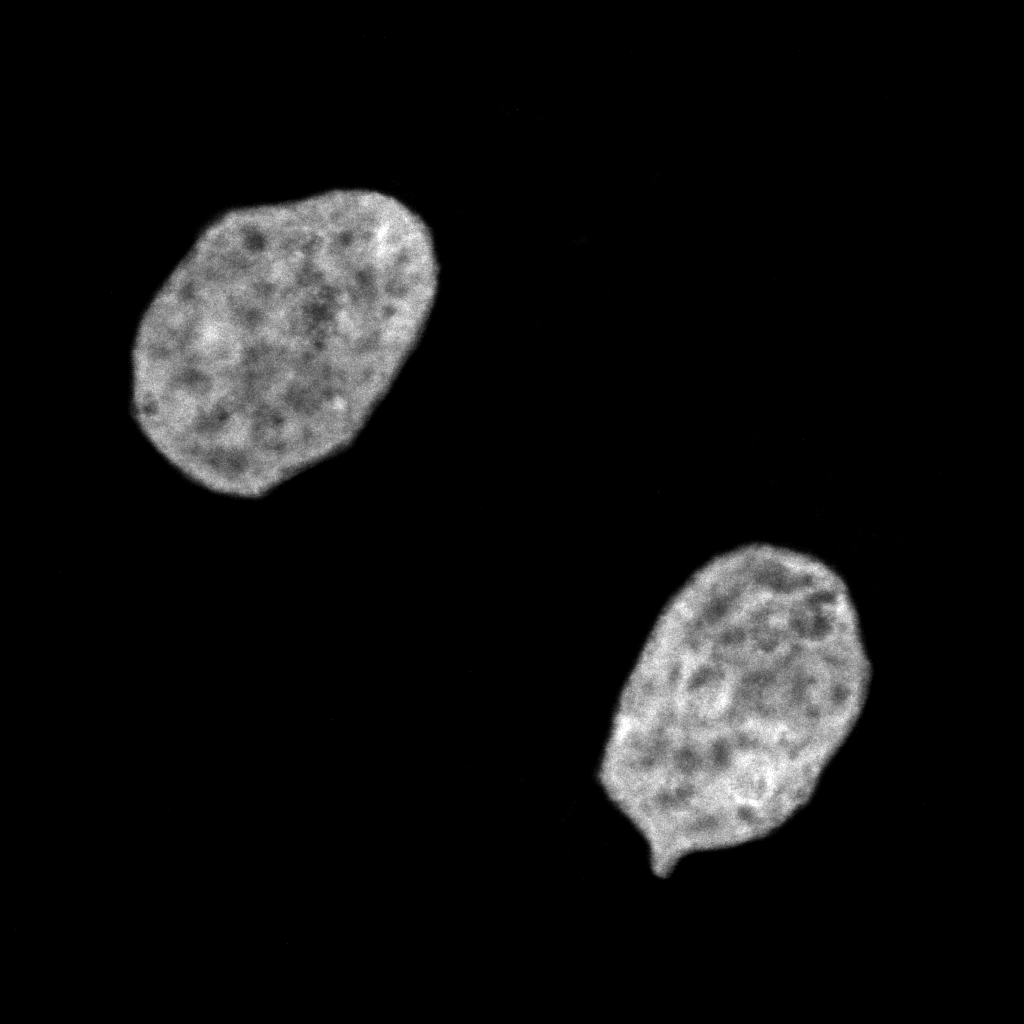

Supplement: Supplementary file 9 — Figure EV1-5 Source Data [file 44318_2025_672_MOESM9_ESM.zip › EV Source Data/EV1/EV1B/WT_DAPI.tif]

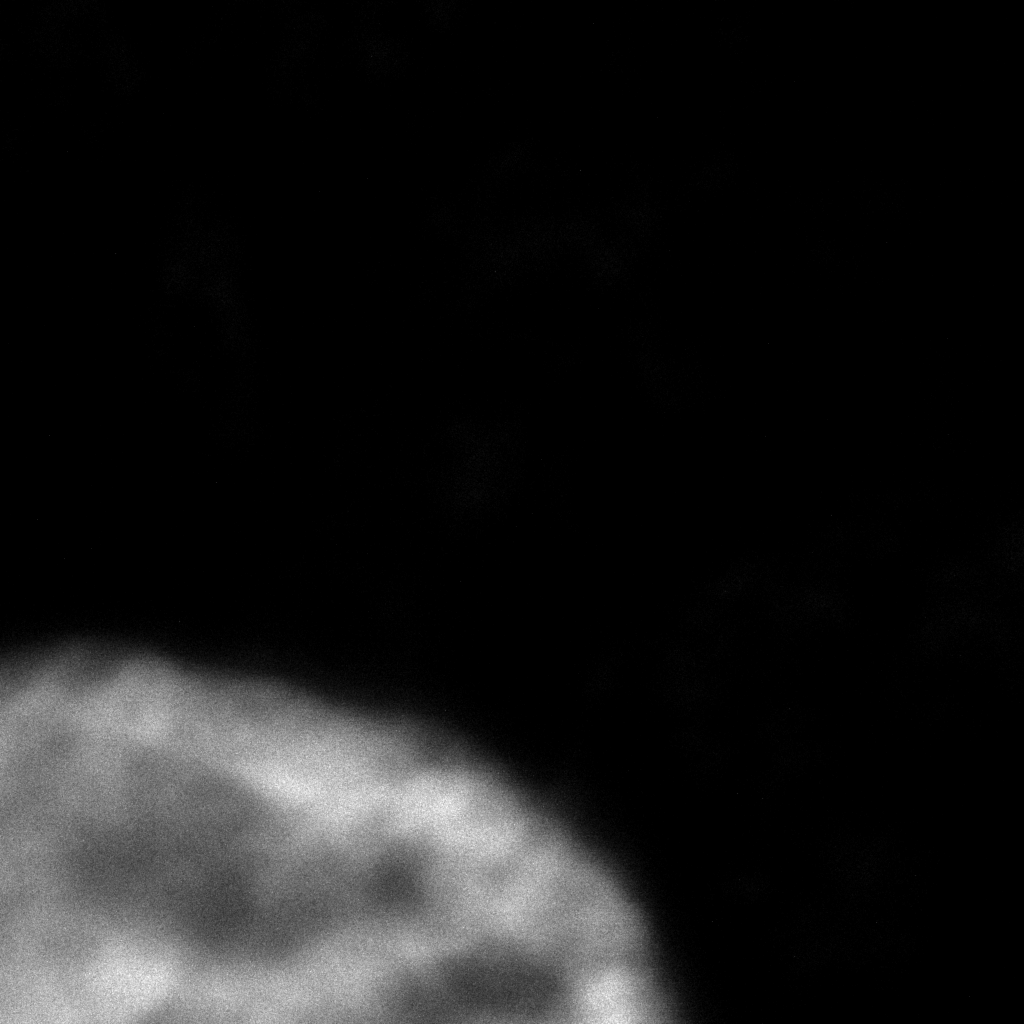

Supplement: Supplementary file 9 — Figure EV1-5 Source Data [file 44318_2025_672_MOESM9_ESM.zip › EV Source Data/EV1/EV1B/WT_DAPI_zoom.tif]

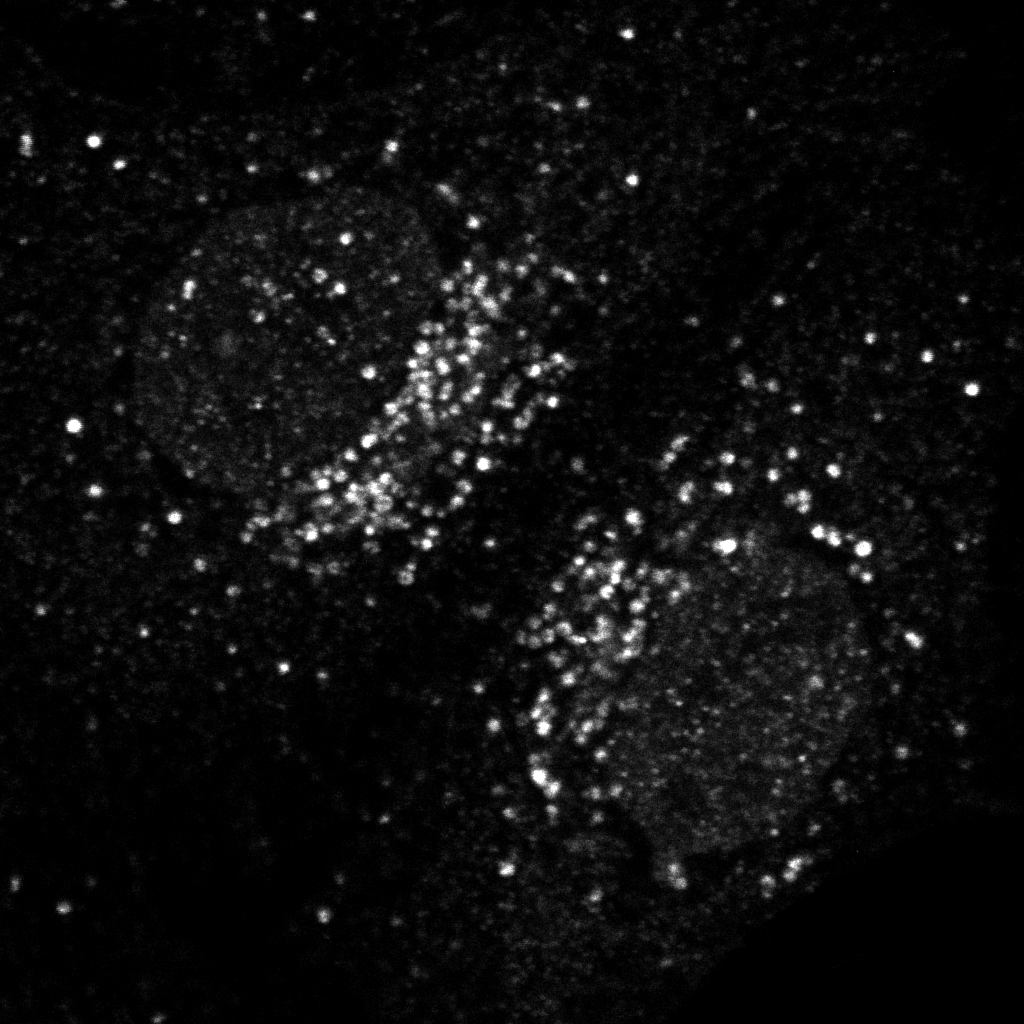

Supplement: Supplementary file 9 — Figure EV1-5 Source Data [file 44318_2025_672_MOESM9_ESM.zip › EV Source Data/EV1/EV1B/WT_Gal3.tif]

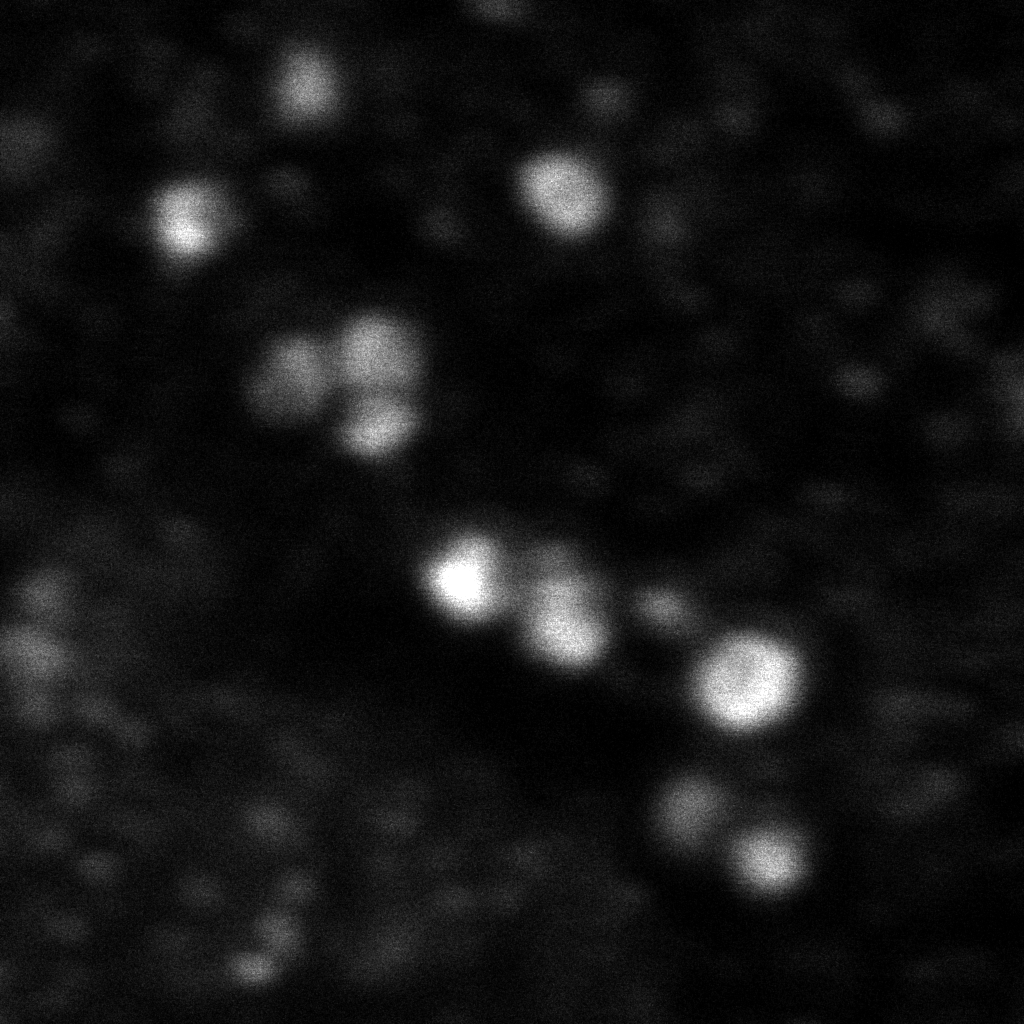

Supplement: Supplementary file 9 — Figure EV1-5 Source Data [file 44318_2025_672_MOESM9_ESM.zip › EV Source Data/EV1/EV1B/WT_Gal3_zoom.tif]

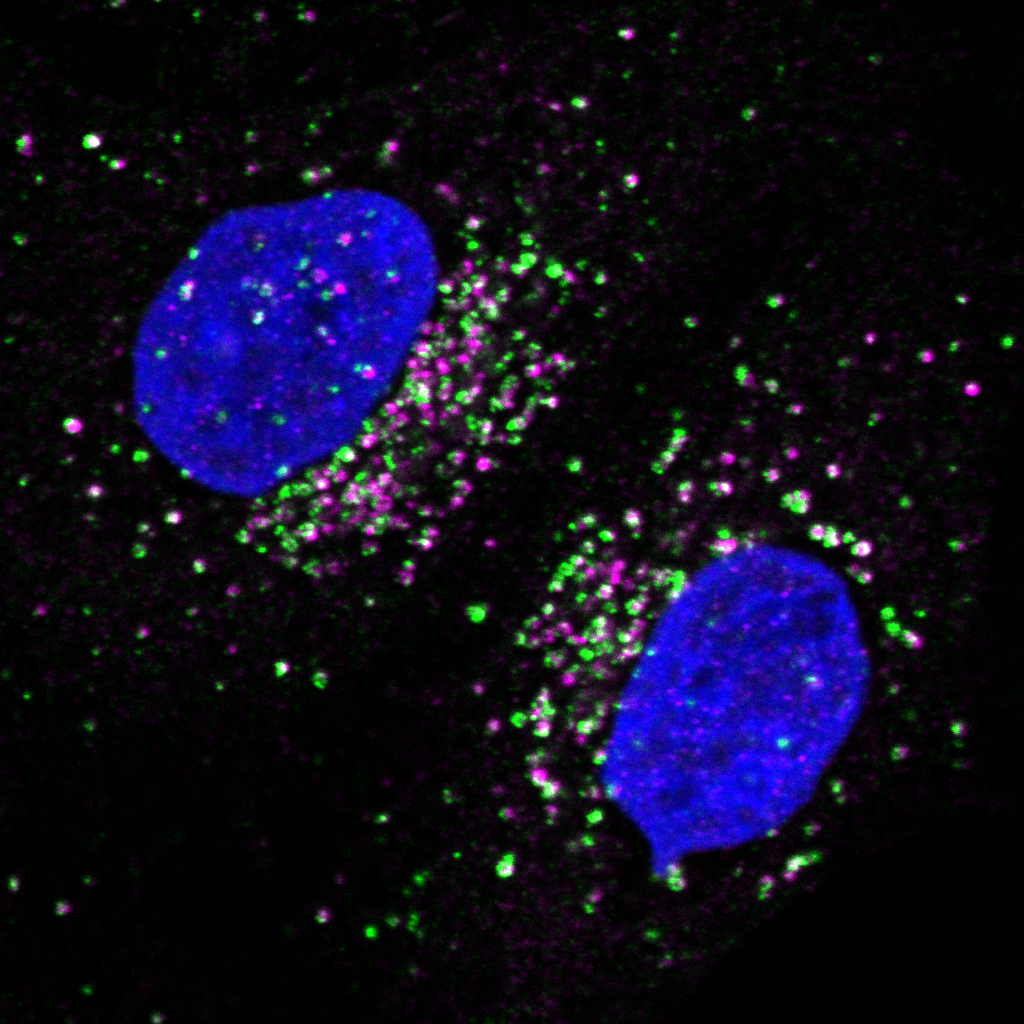

Supplement: Supplementary file 9 — Figure EV1-5 Source Data [file 44318_2025_672_MOESM9_ESM.zip › EV Source Data/EV1/EV1B/WT_merge.tif]

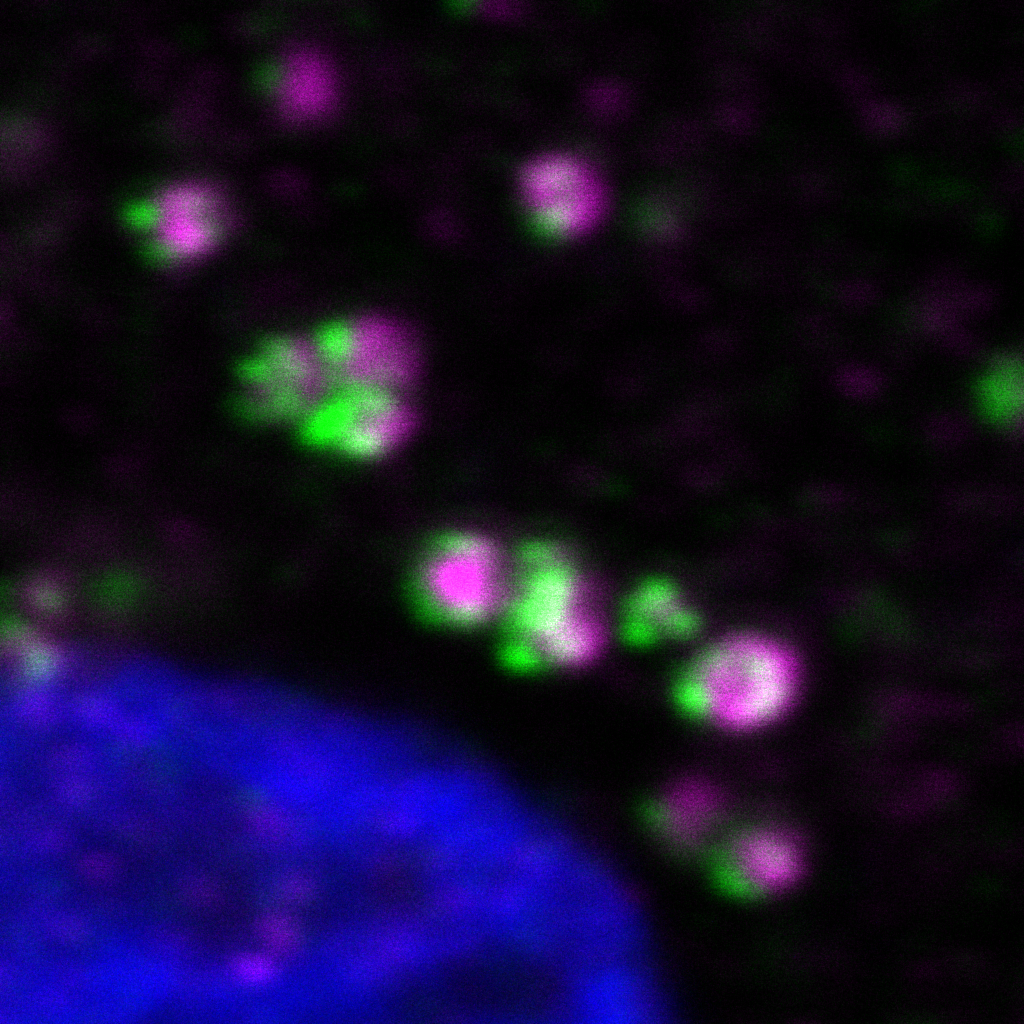

Supplement: Supplementary file 9 — Figure EV1-5 Source Data [file 44318_2025_672_MOESM9_ESM.zip › EV Source Data/EV1/EV1B/WT_merge_zoom.tif]

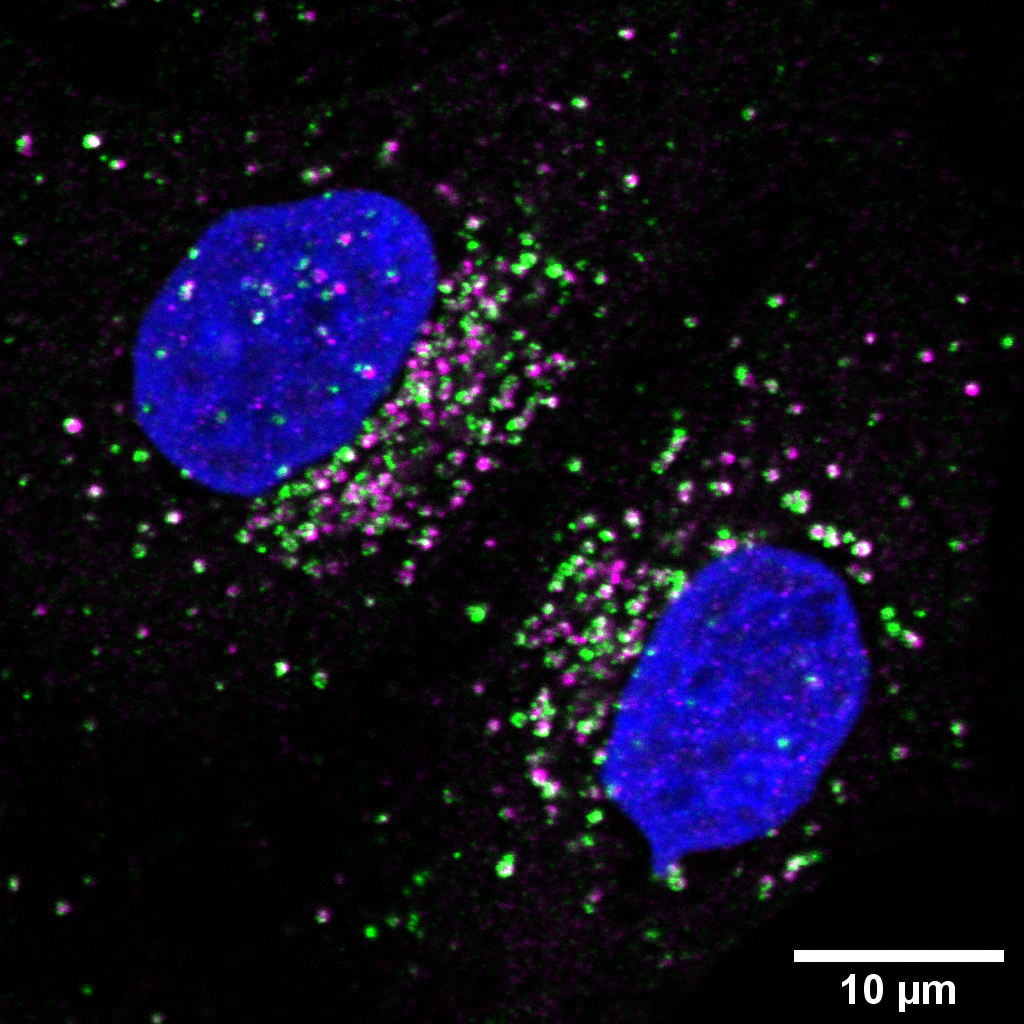

Supplement: Supplementary file 9 — Figure EV1-5 Source Data [file 44318_2025_672_MOESM9_ESM.zip › EV Source Data/EV1/EV1B/WT_scale.tif]

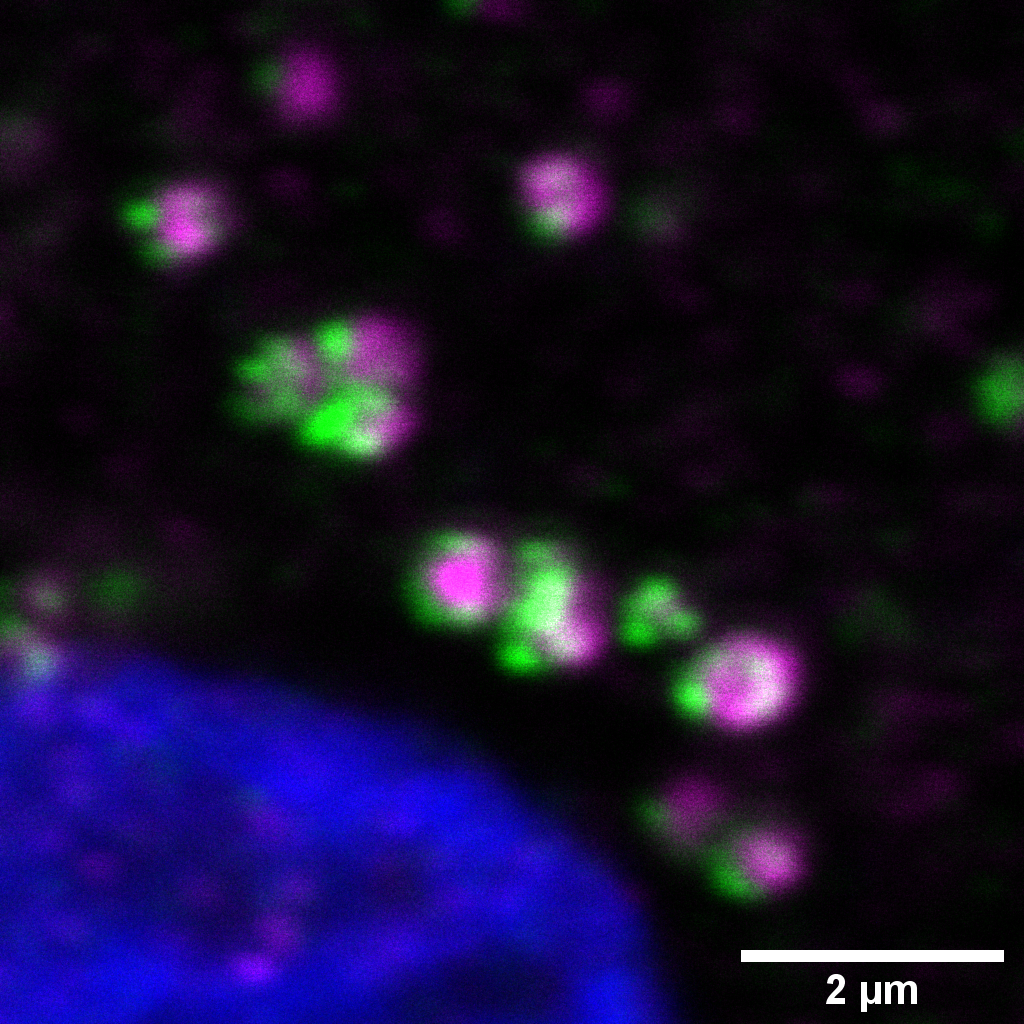

Supplement: Supplementary file 9 — Figure EV1-5 Source Data [file 44318_2025_672_MOESM9_ESM.zip › EV Source Data/EV1/EV1B/WT_scale_zoom.tif]

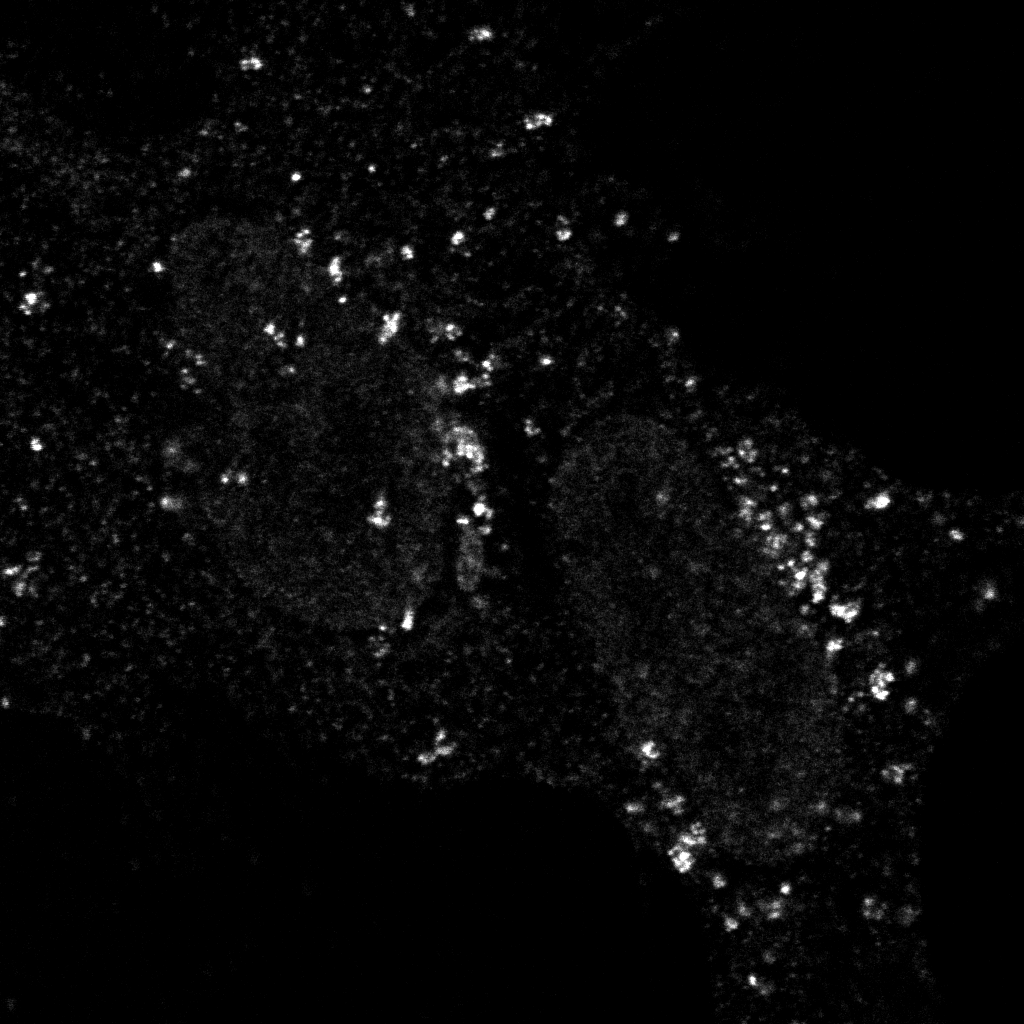

Supplement: Supplementary file 9 — Figure EV1-5 Source Data [file 44318_2025_672_MOESM9_ESM.zip › EV Source Data/EV1/EV1C/16KO_ALIX.tif]

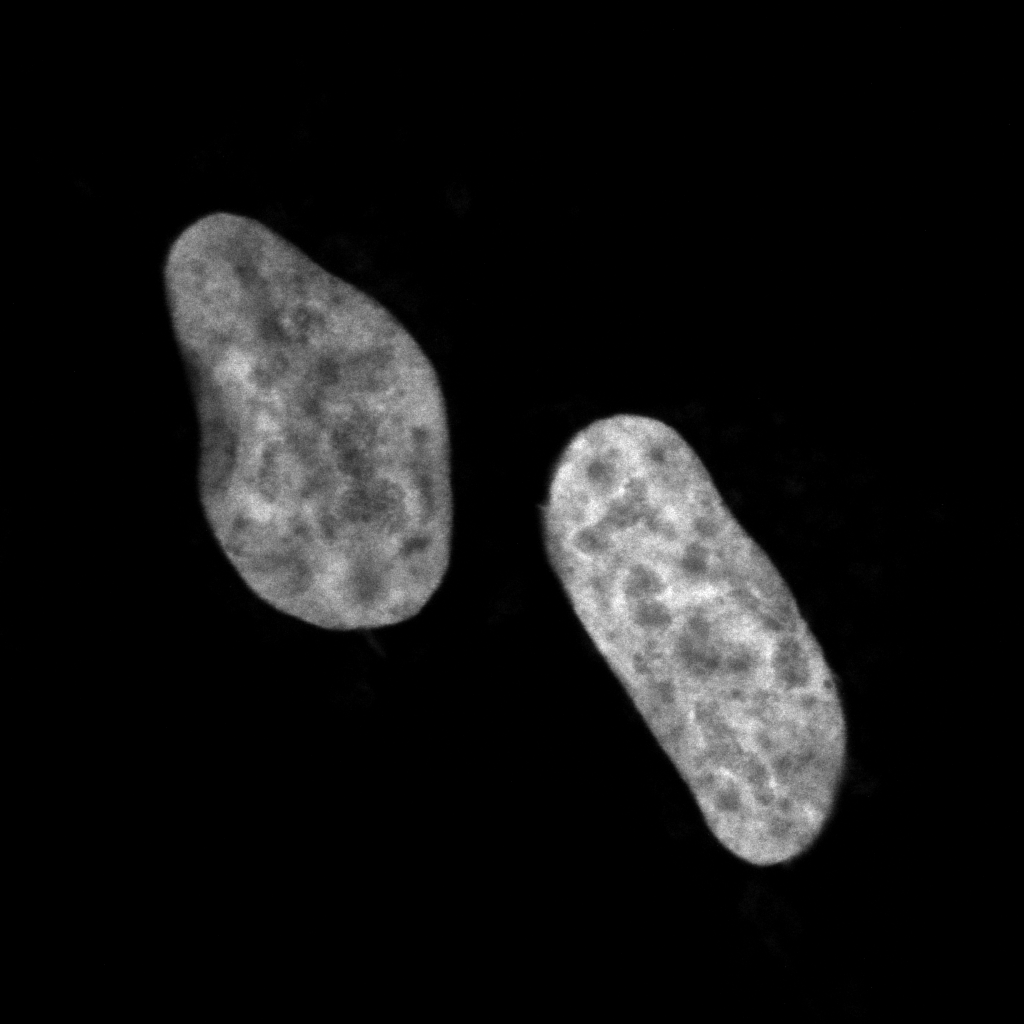

Supplement: Supplementary file 9 — Figure EV1-5 Source Data [file 44318_2025_672_MOESM9_ESM.zip › EV Source Data/EV1/EV1C/16KO_DAPI.tif]

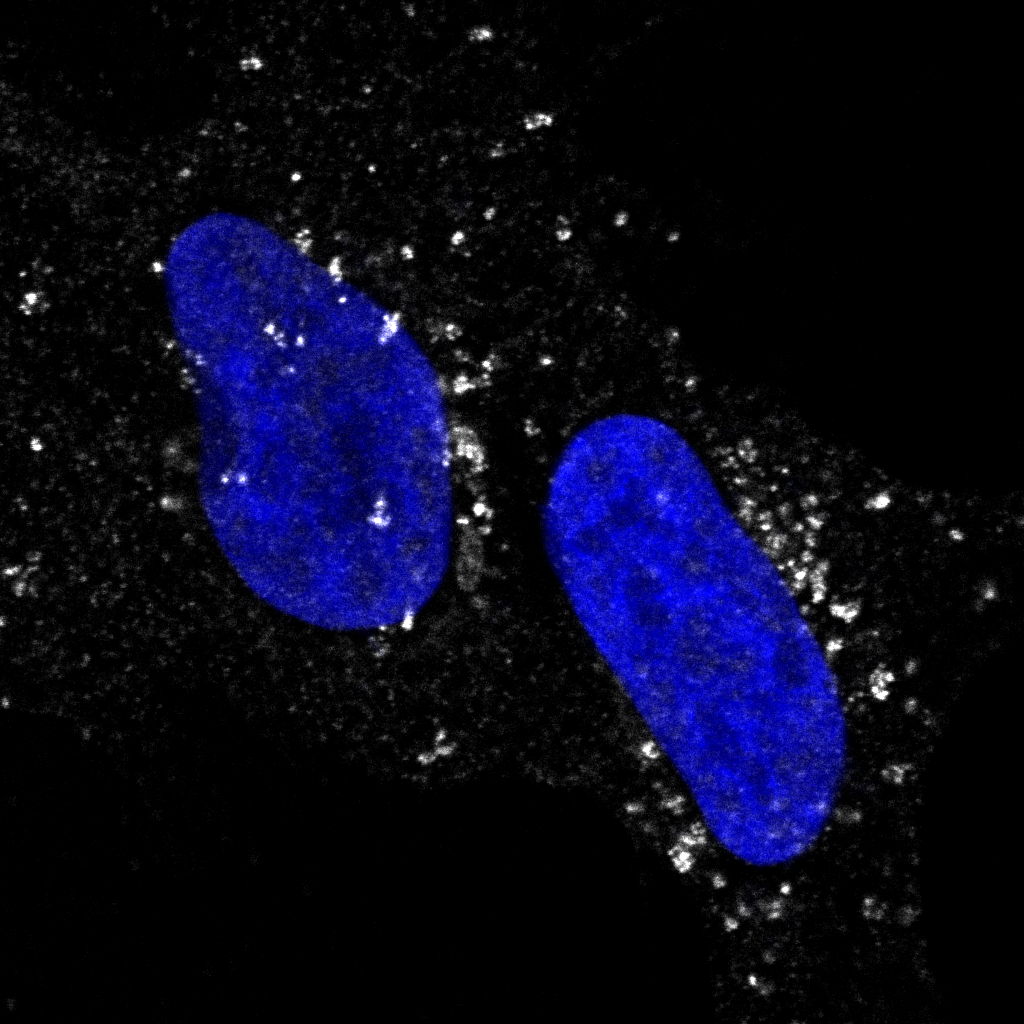

Supplement: Supplementary file 9 — Figure EV1-5 Source Data [file 44318_2025_672_MOESM9_ESM.zip › EV Source Data/EV1/EV1C/16KO_merge.tif]

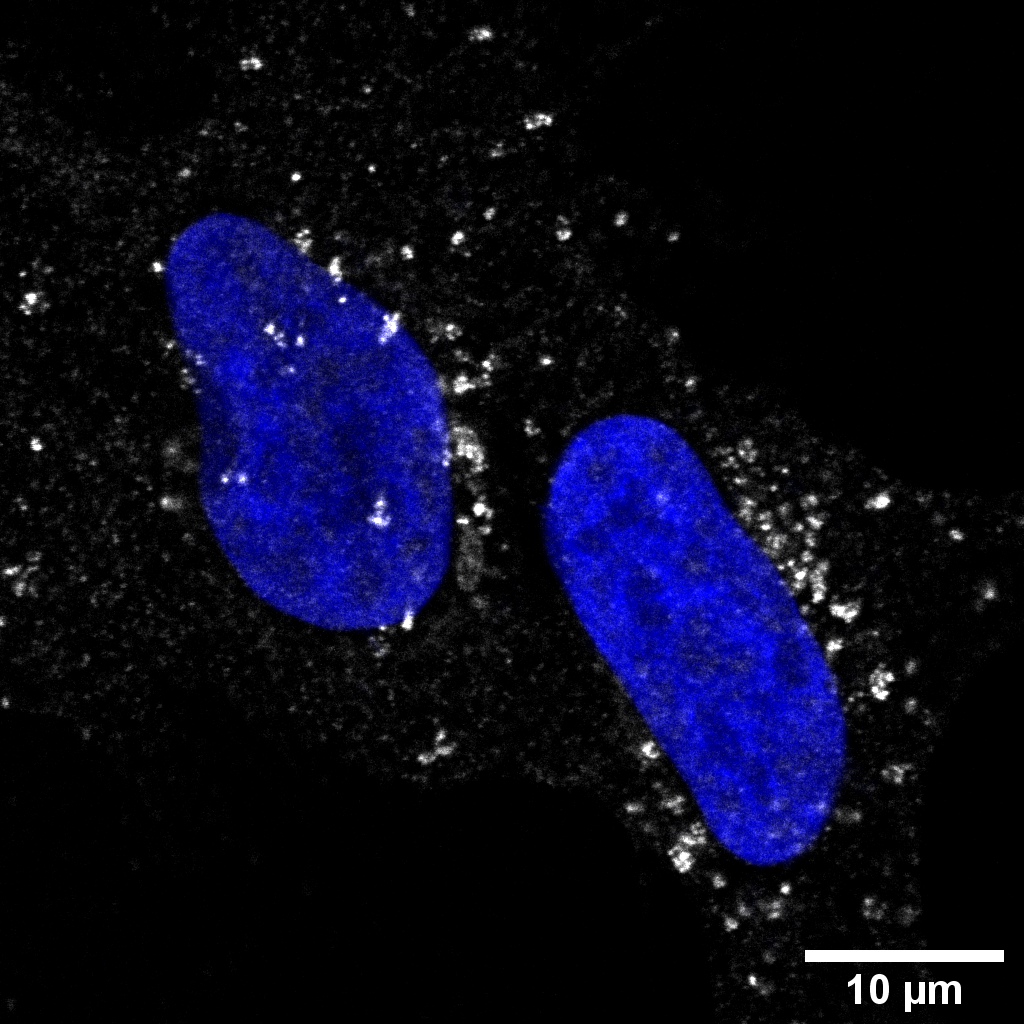

Supplement: Supplementary file 9 — Figure EV1-5 Source Data [file 44318_2025_672_MOESM9_ESM.zip › EV Source Data/EV1/EV1C/16KO_scale.tif]

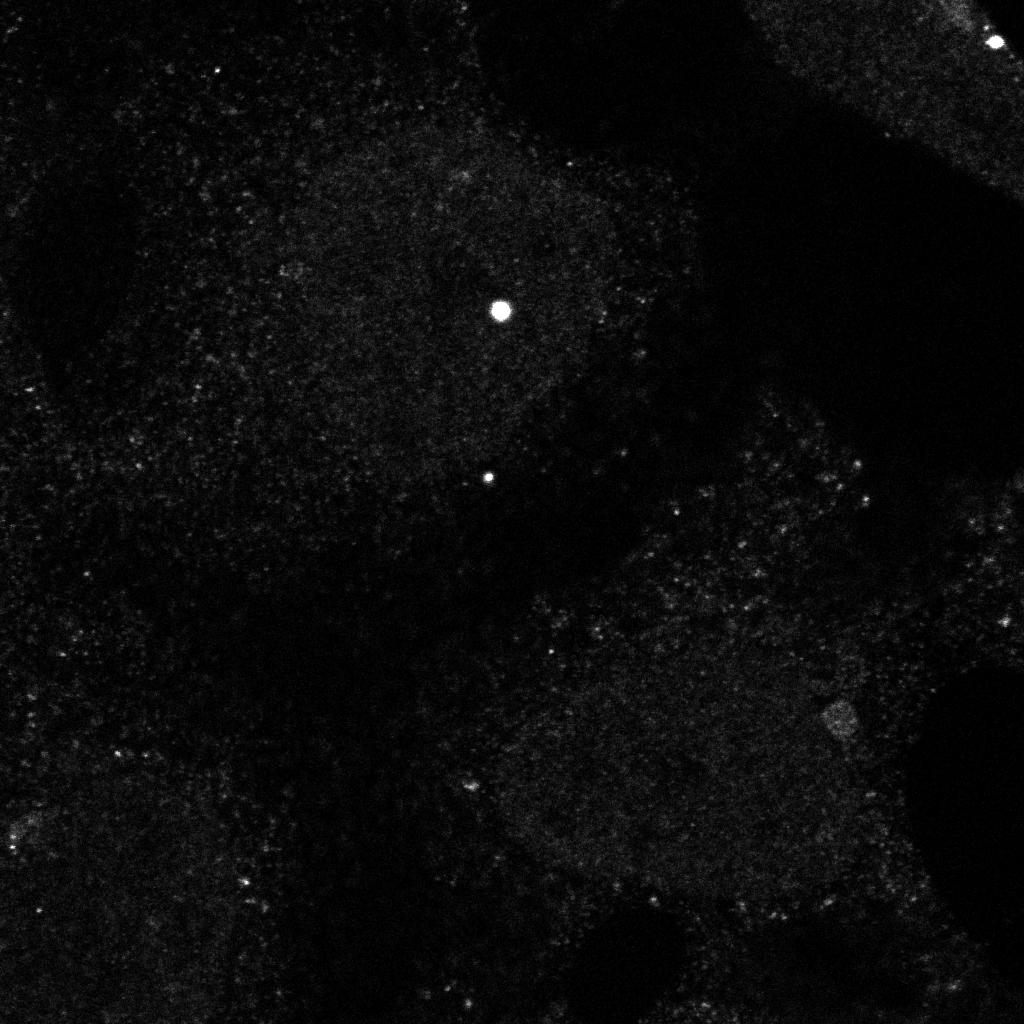

Supplement: Supplementary file 9 — Figure EV1-5 Source Data [file 44318_2025_672_MOESM9_ESM.zip › EV Source Data/EV1/EV1C/E3 DKO_ALIX.tif]

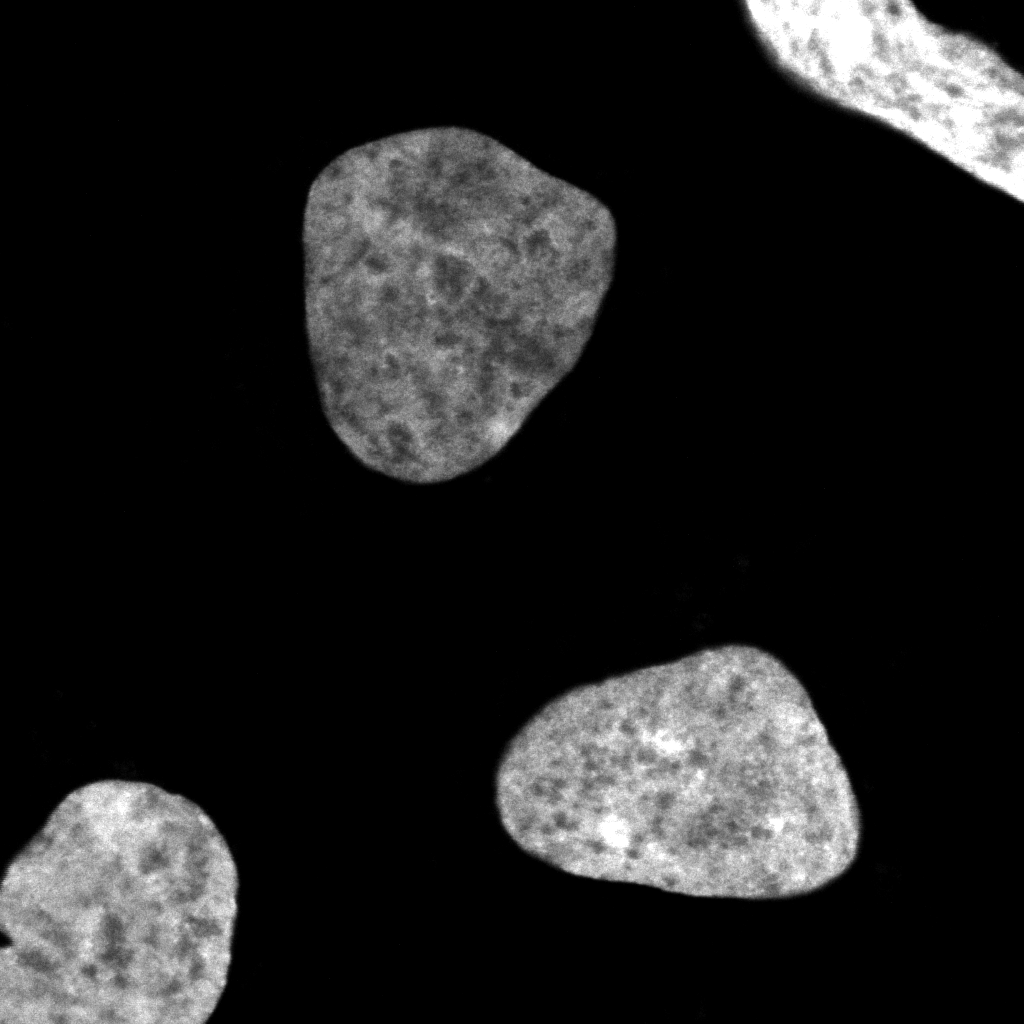

Supplement: Supplementary file 9 — Figure EV1-5 Source Data [file 44318_2025_672_MOESM9_ESM.zip › EV Source Data/EV1/EV1C/E3 DKO_DAPI.tif]

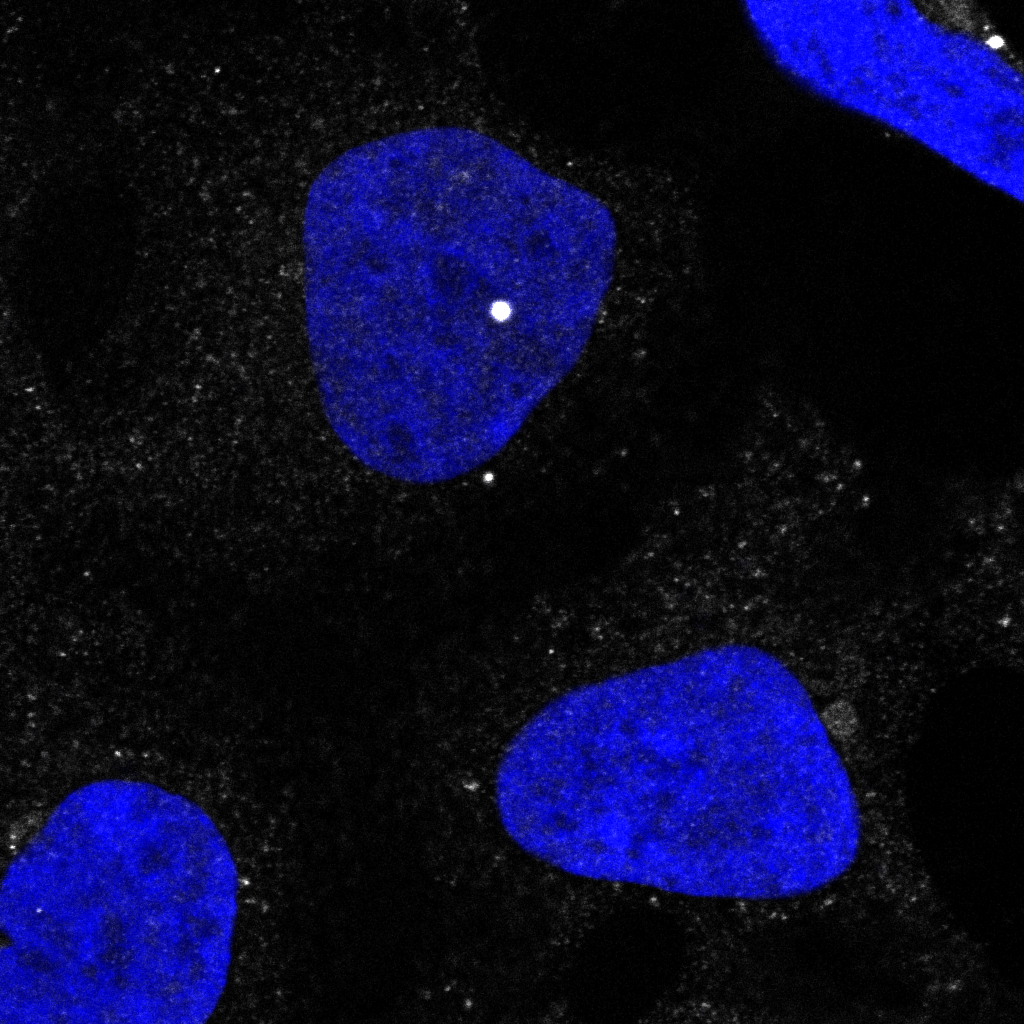

Supplement: Supplementary file 9 — Figure EV1-5 Source Data [file 44318_2025_672_MOESM9_ESM.zip › EV Source Data/EV1/EV1C/E3 DKO_merge.tif]

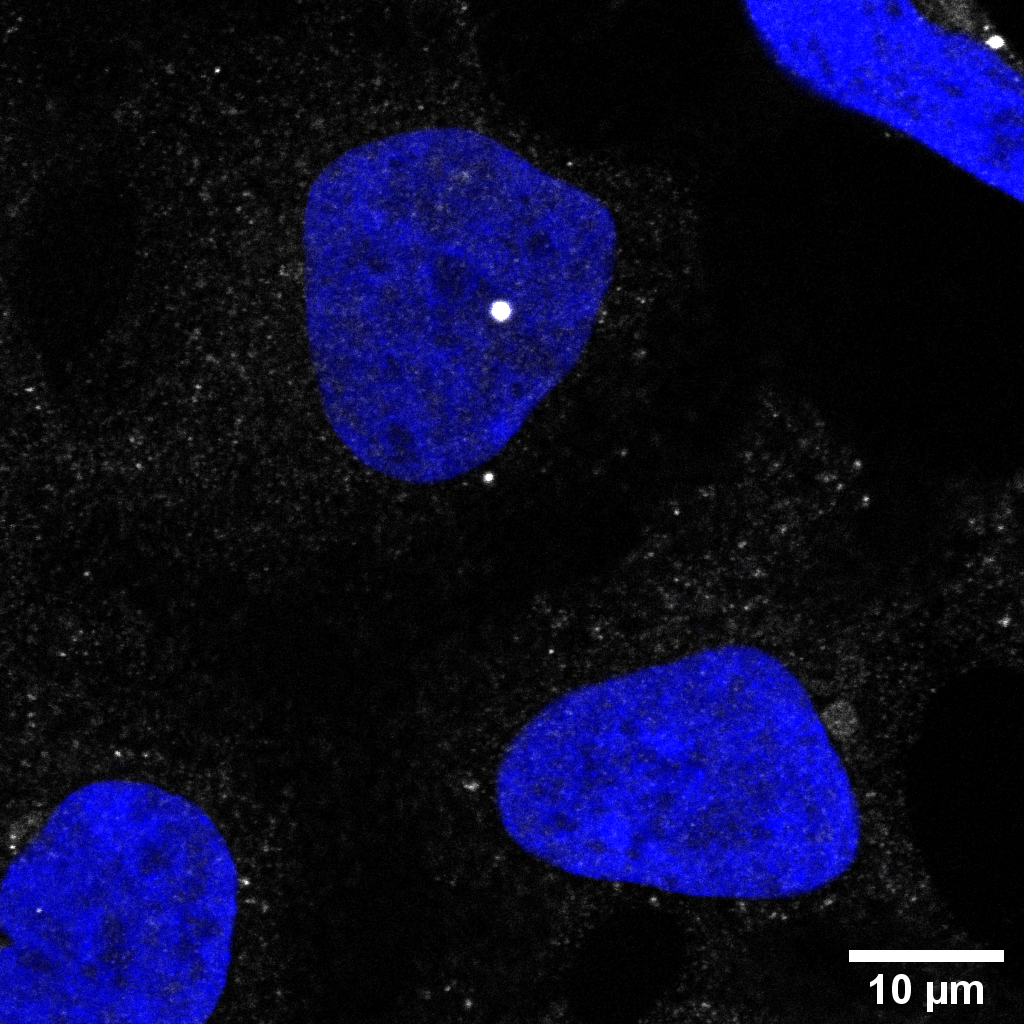

Supplement: Supplementary file 9 — Figure EV1-5 Source Data [file 44318_2025_672_MOESM9_ESM.zip › EV Source Data/EV1/EV1C/E3 DKO_scale.tif]

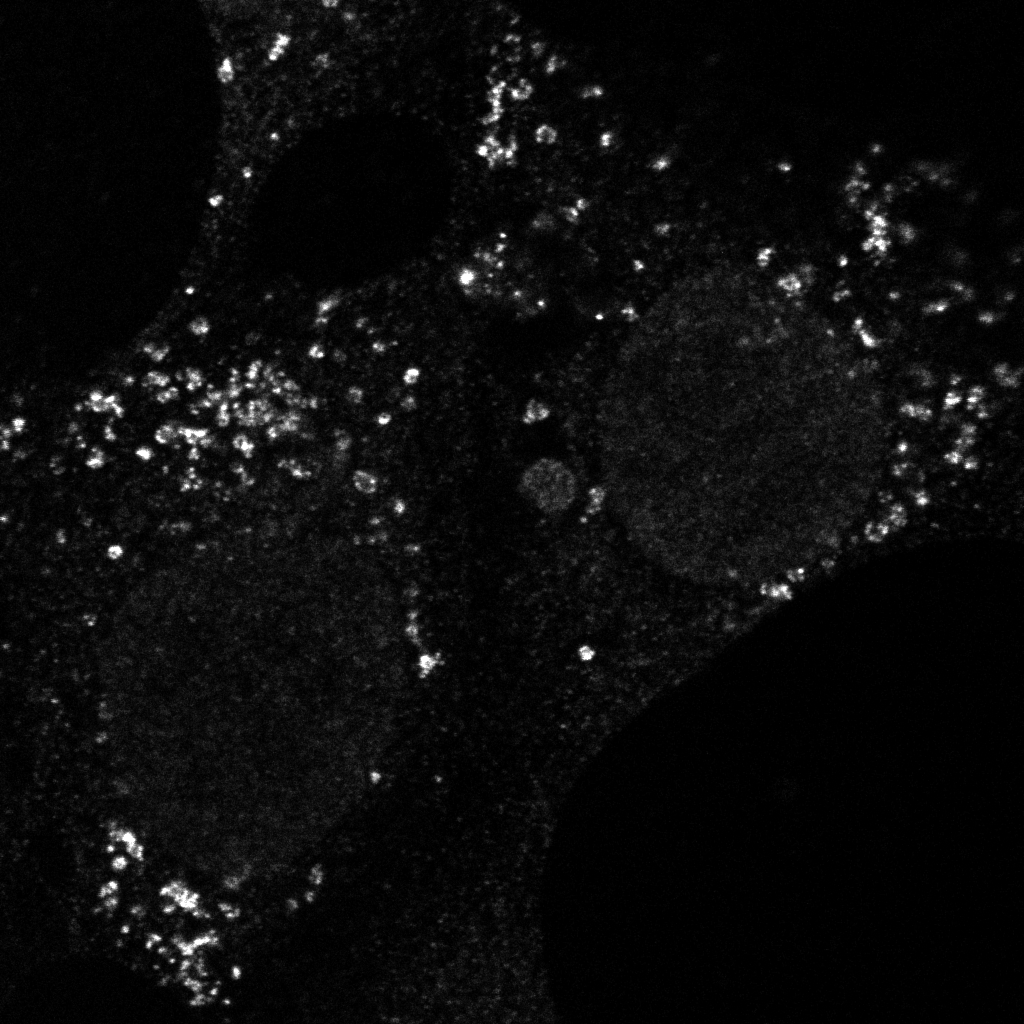

Supplement: Supplementary file 9 — Figure EV1-5 Source Data [file 44318_2025_672_MOESM9_ESM.zip › EV Source Data/EV1/EV1C/TECKO_ALIX.tif]

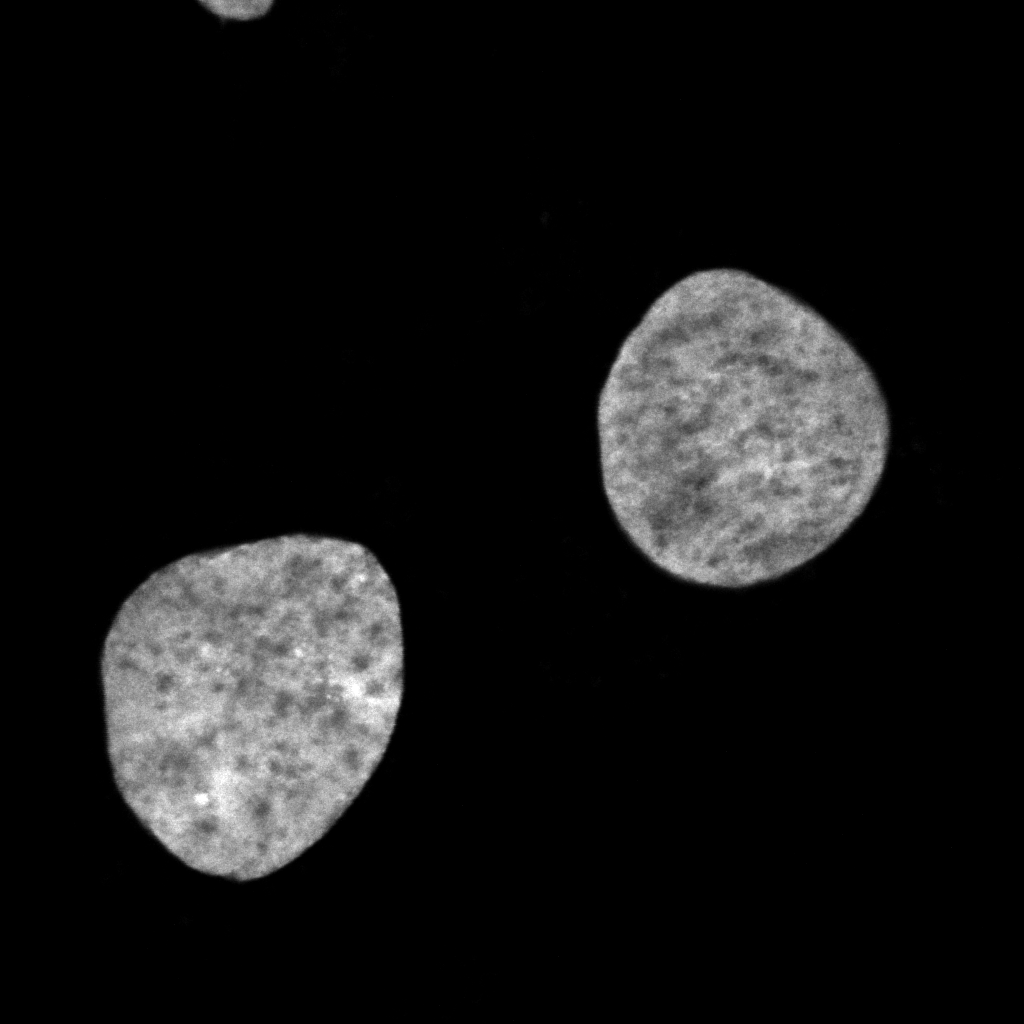

Supplement: Supplementary file 9 — Figure EV1-5 Source Data [file 44318_2025_672_MOESM9_ESM.zip › EV Source Data/EV1/EV1C/TECKO_DAPI.tif]

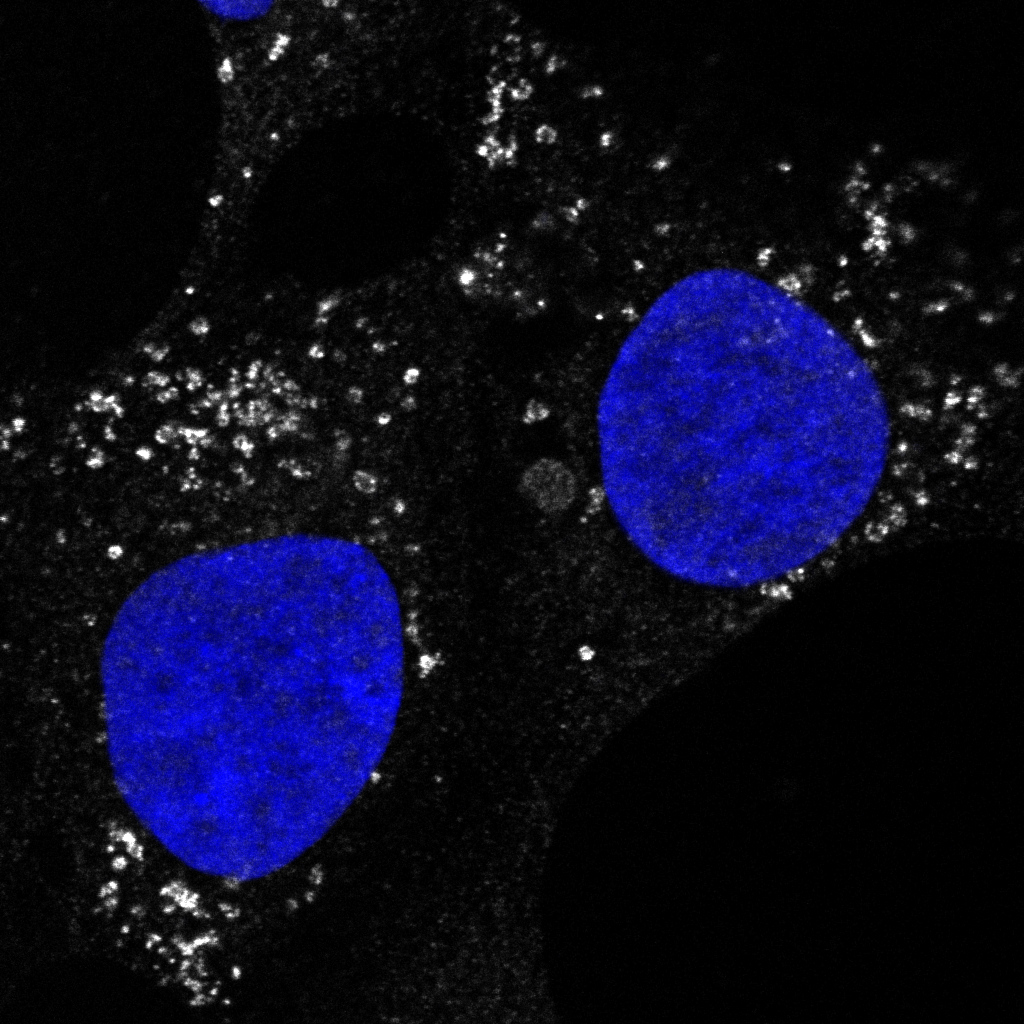

Supplement: Supplementary file 9 — Figure EV1-5 Source Data [file 44318_2025_672_MOESM9_ESM.zip › EV Source Data/EV1/EV1C/TECKO_merge.tif]

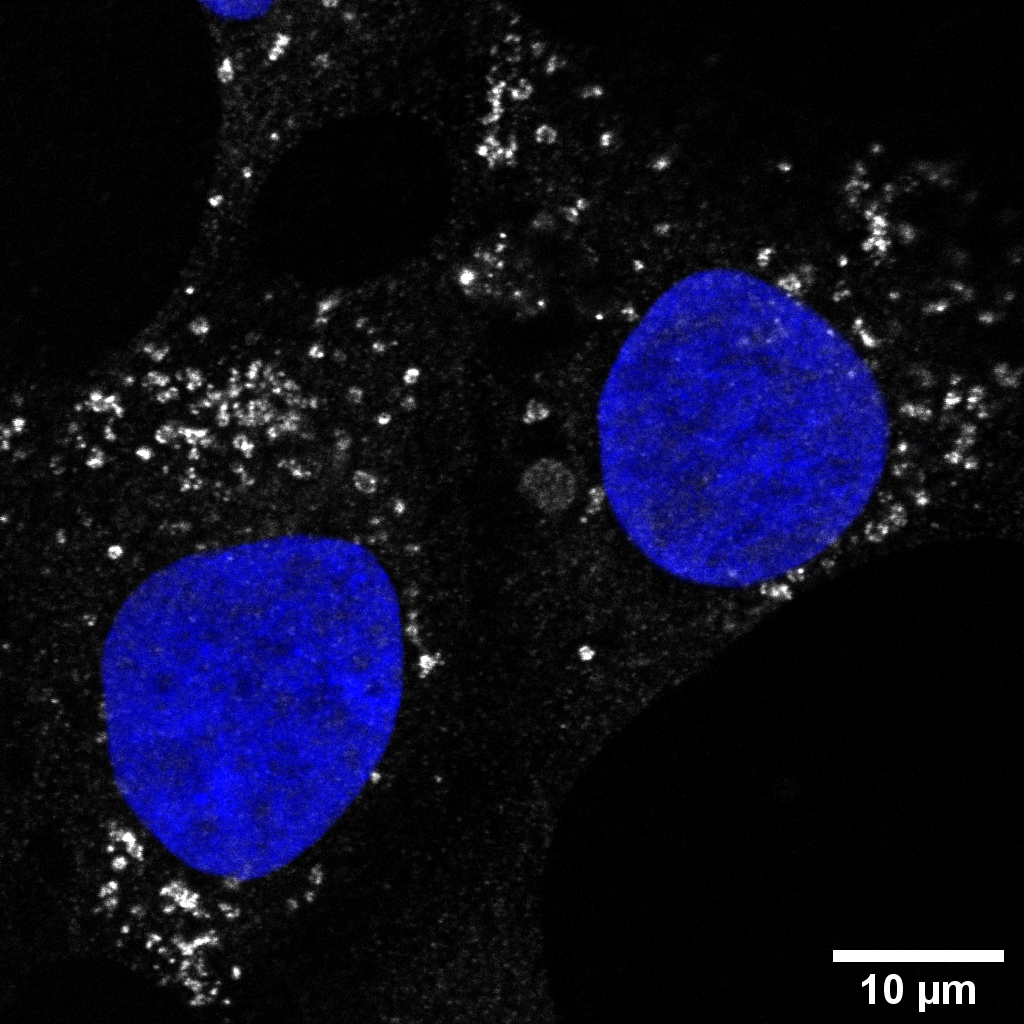

Supplement: Supplementary file 9 — Figure EV1-5 Source Data [file 44318_2025_672_MOESM9_ESM.zip › EV Source Data/EV1/EV1C/TECKO_scale.tif]

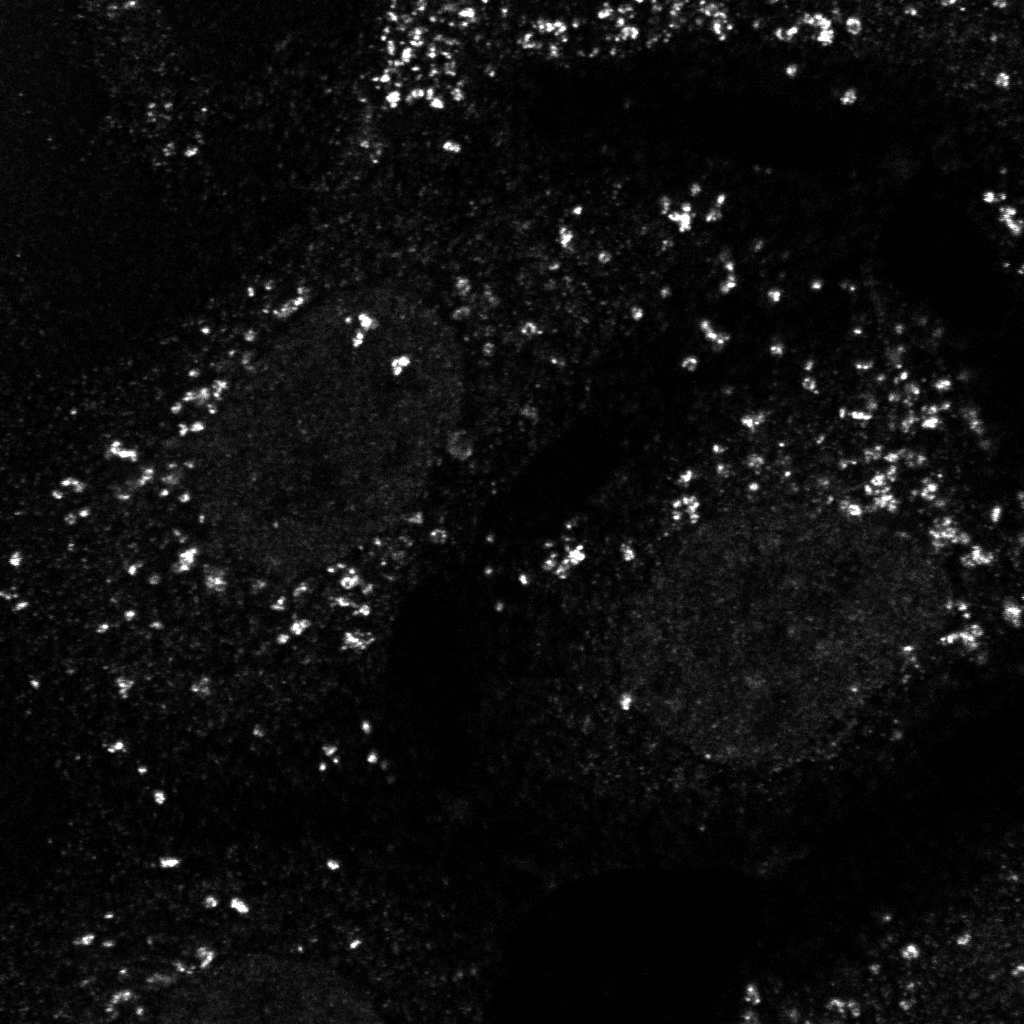

Supplement: Supplementary file 9 — Figure EV1-5 Source Data [file 44318_2025_672_MOESM9_ESM.zip › EV Source Data/EV1/EV1C/WT_ALIX.tif]

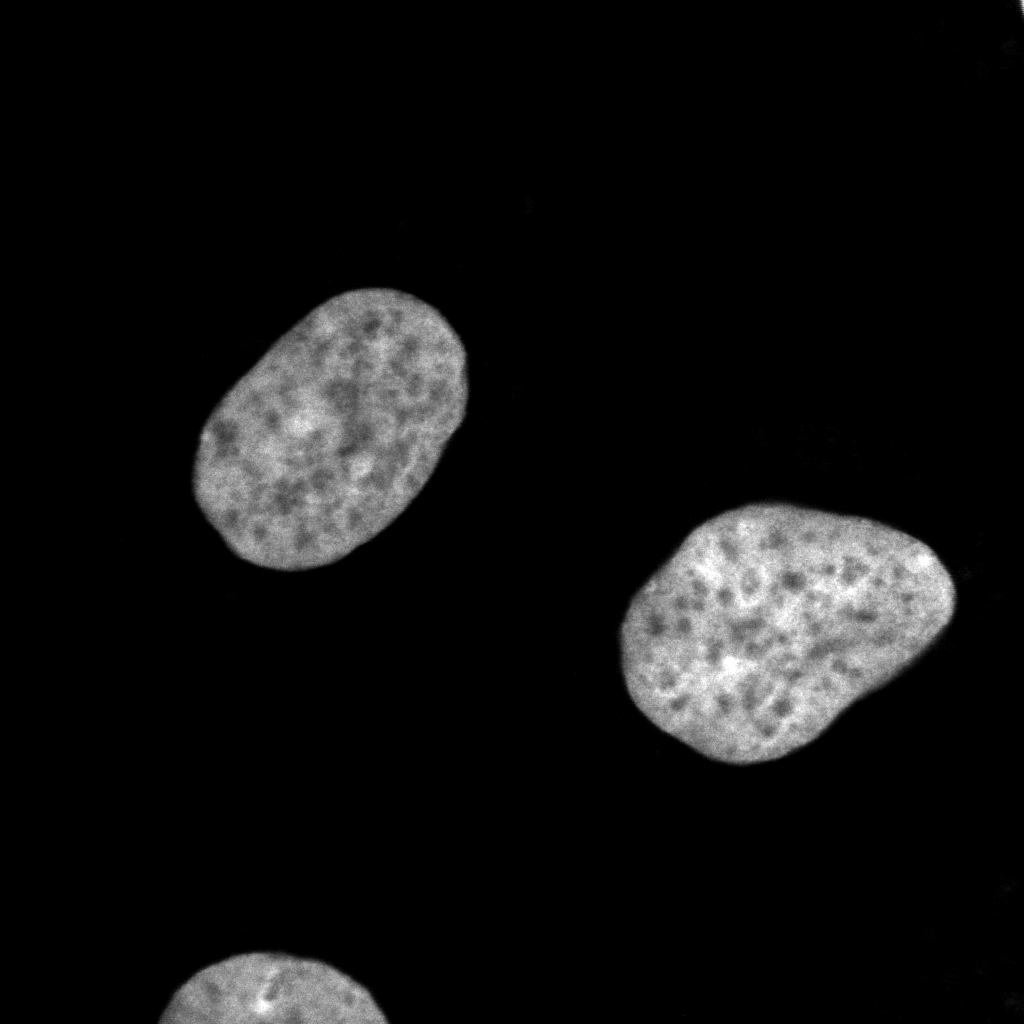

Supplement: Supplementary file 9 — Figure EV1-5 Source Data [file 44318_2025_672_MOESM9_ESM.zip › EV Source Data/EV1/EV1C/WT_DAPI.tif]

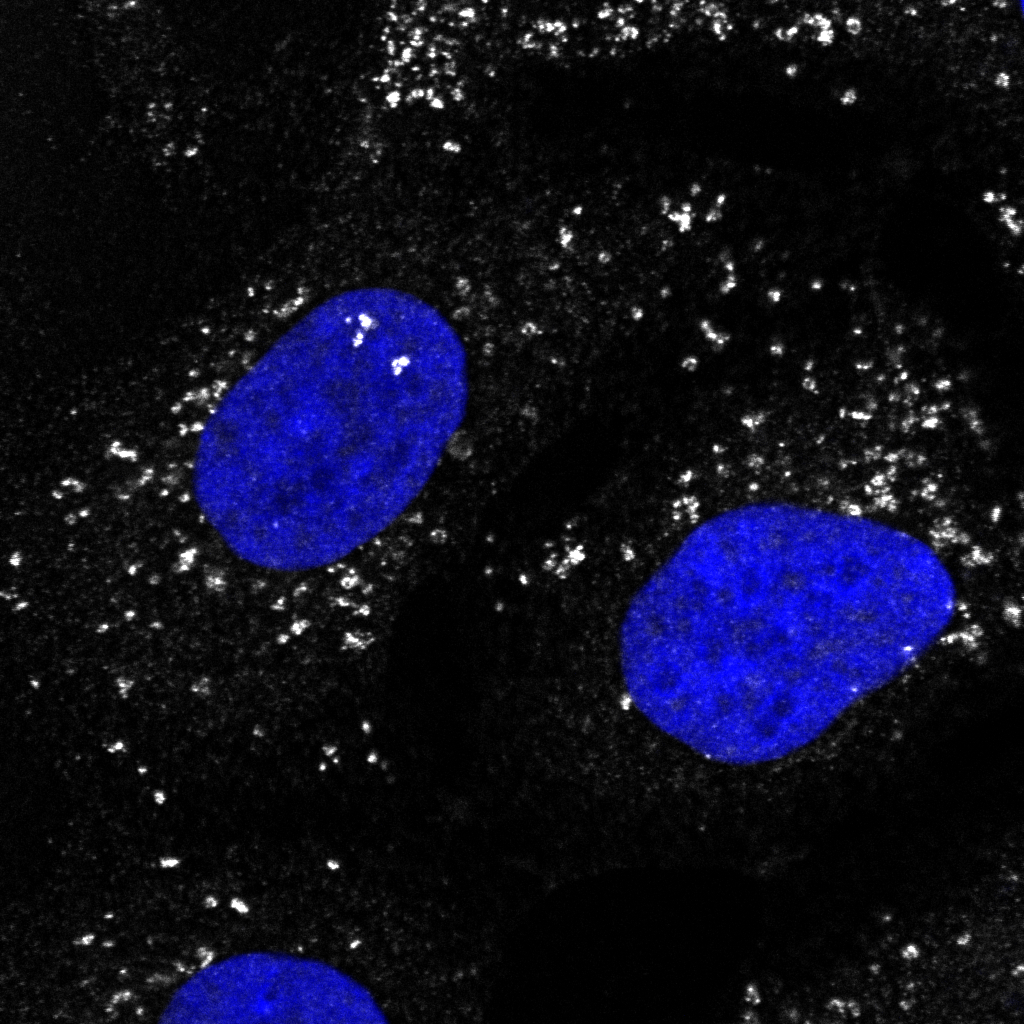

Supplement: Supplementary file 9 — Figure EV1-5 Source Data [file 44318_2025_672_MOESM9_ESM.zip › EV Source Data/EV1/EV1C/WT_merge.tif]

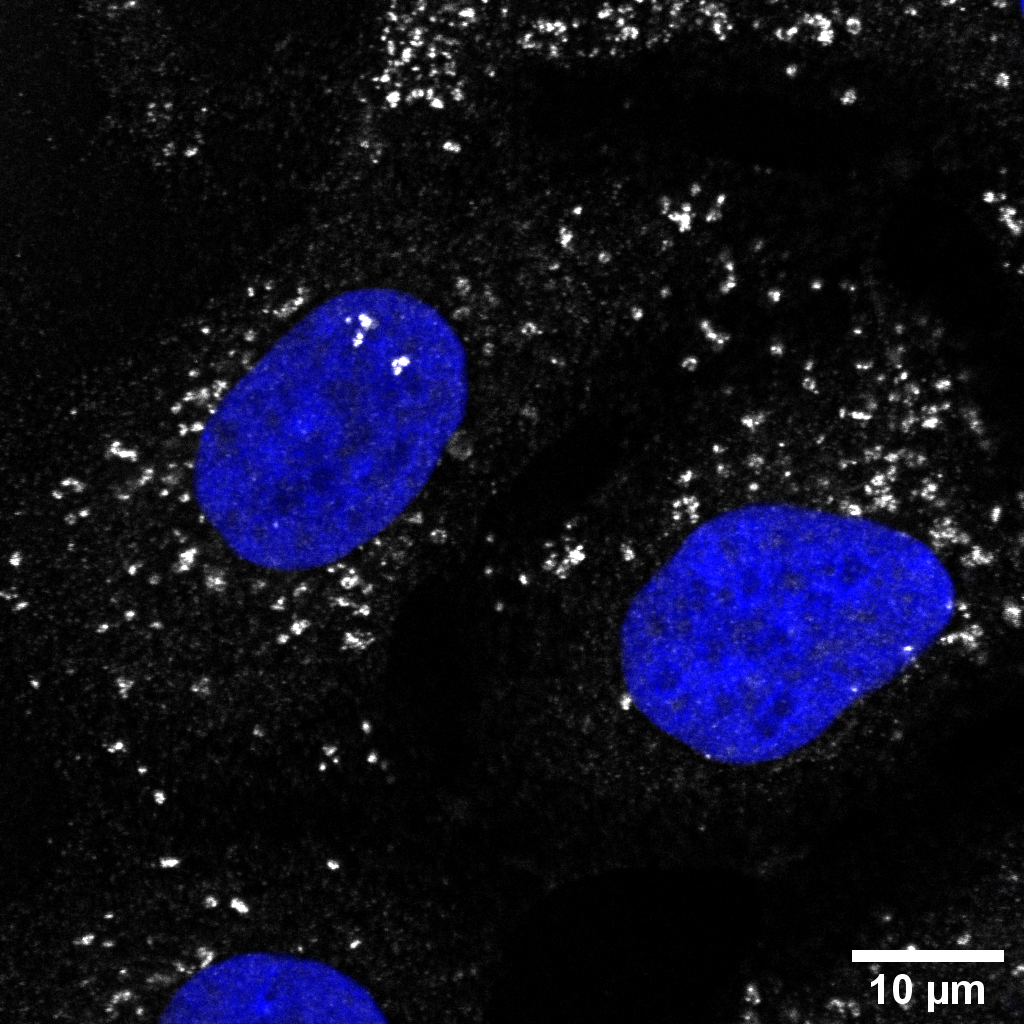

Supplement: Supplementary file 9 — Figure EV1-5 Source Data [file 44318_2025_672_MOESM9_ESM.zip › EV Source Data/EV1/EV1C/WT_scale.tif]

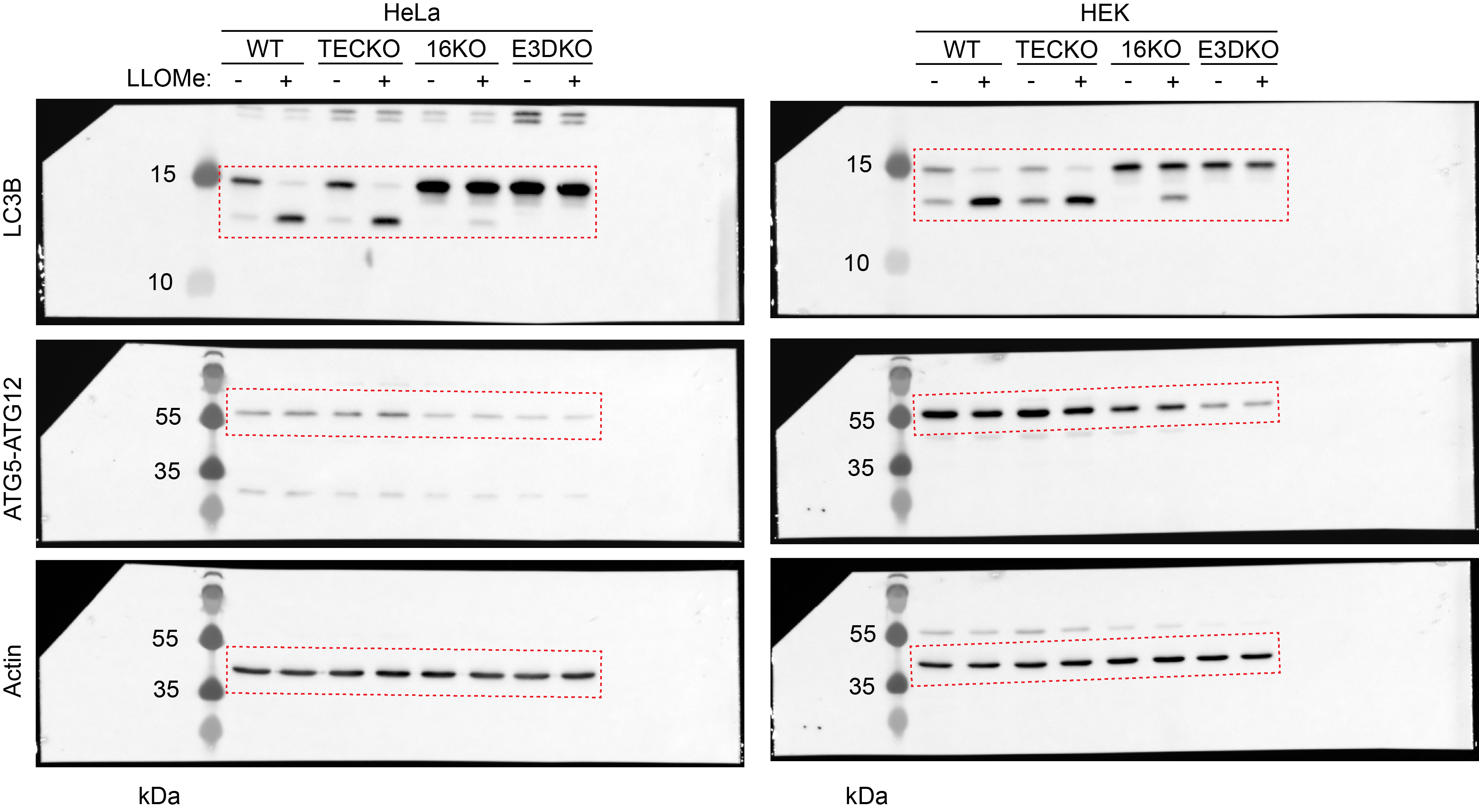

Supplement: Supplementary file 9 — Figure EV1-5 Source Data [file 44318_2025_672_MOESM9_ESM.zip › EV Source Data/EV1/EV1D/EV1D_uncropped blots.jpg]

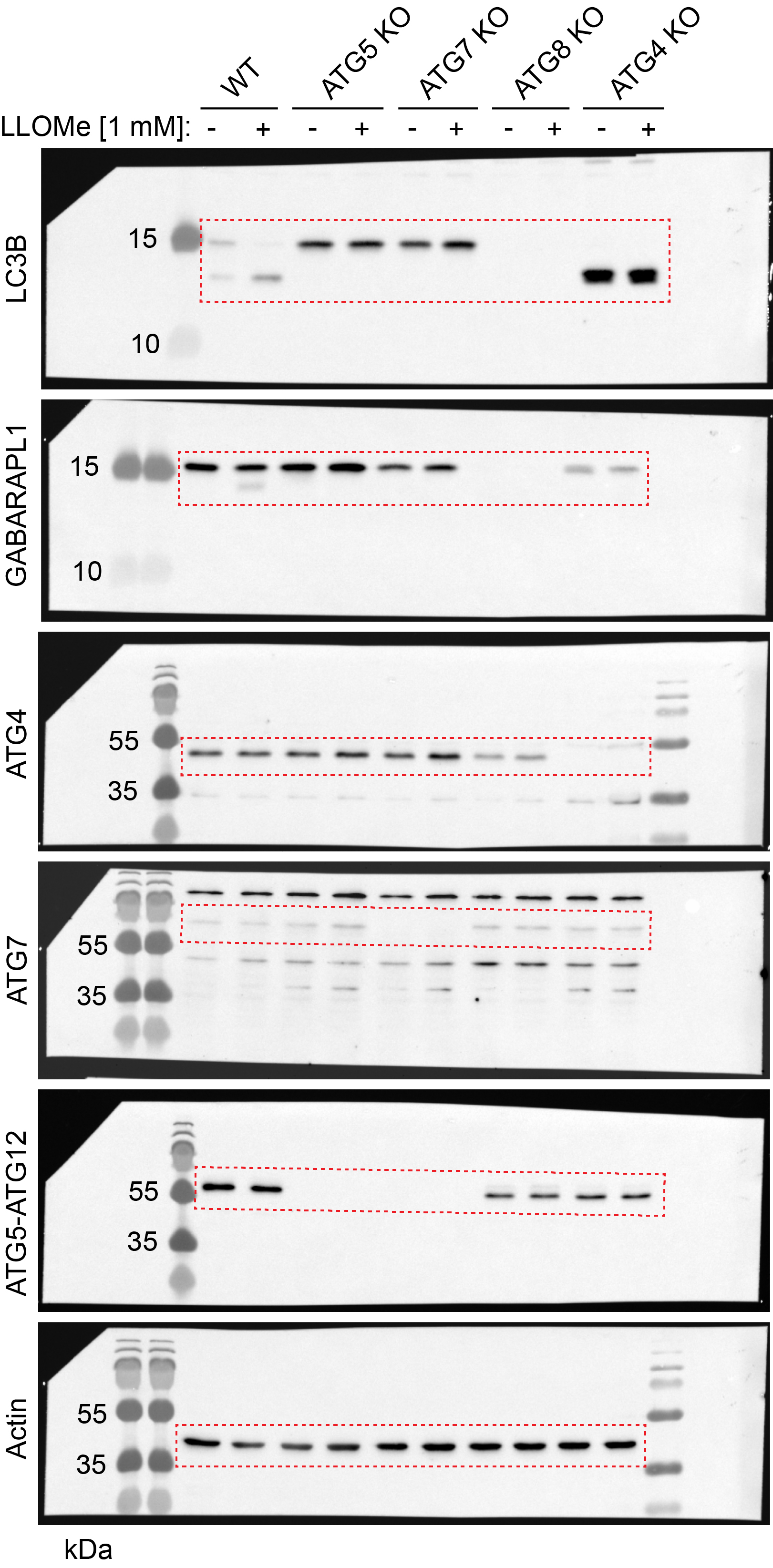

Supplement: Supplementary file 9 — Figure EV1-5 Source Data [file 44318_2025_672_MOESM9_ESM.zip › EV Source Data/EV2/EV2B/EV2B.jpg]

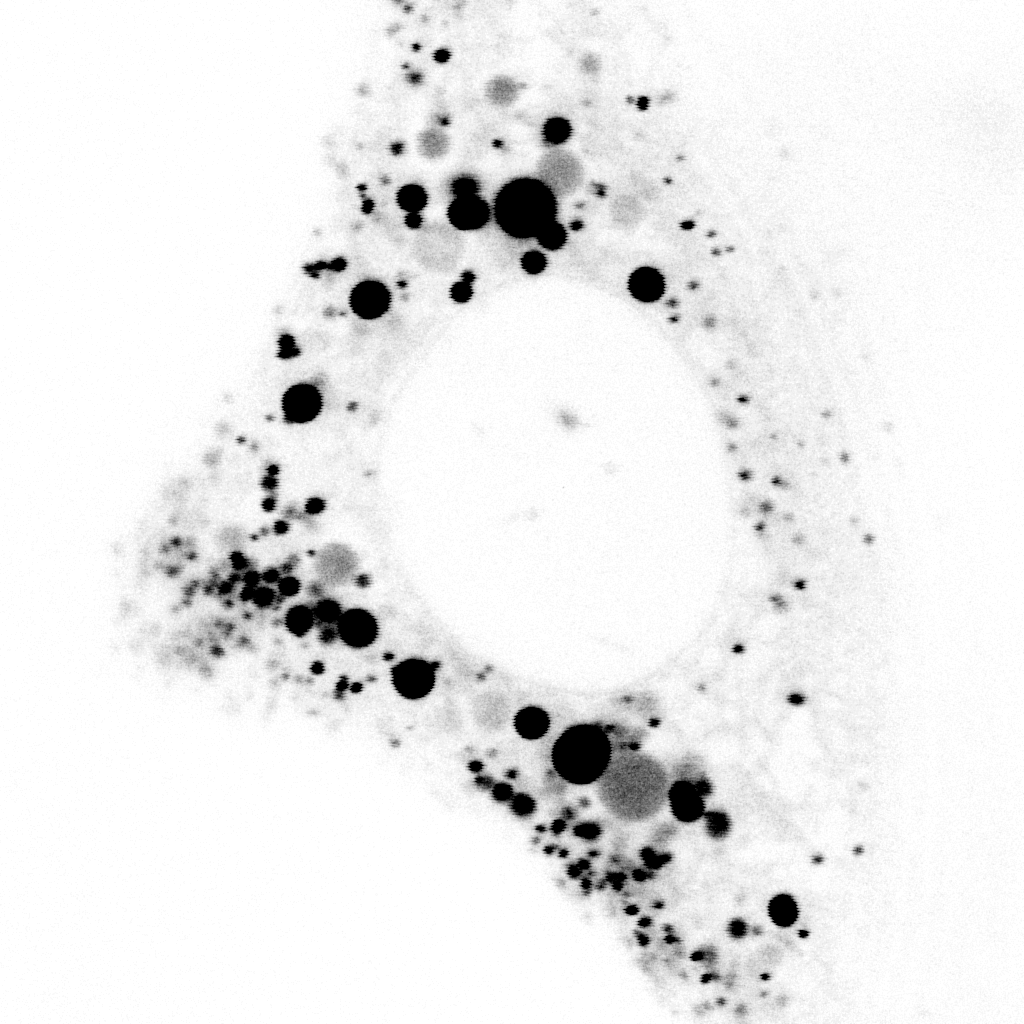

Supplement: Supplementary file 9 — Figure EV1-5 Source Data [file 44318_2025_672_MOESM9_ESM.zip › EV Source Data/EV3/EV3A/LT_inverted.tif]

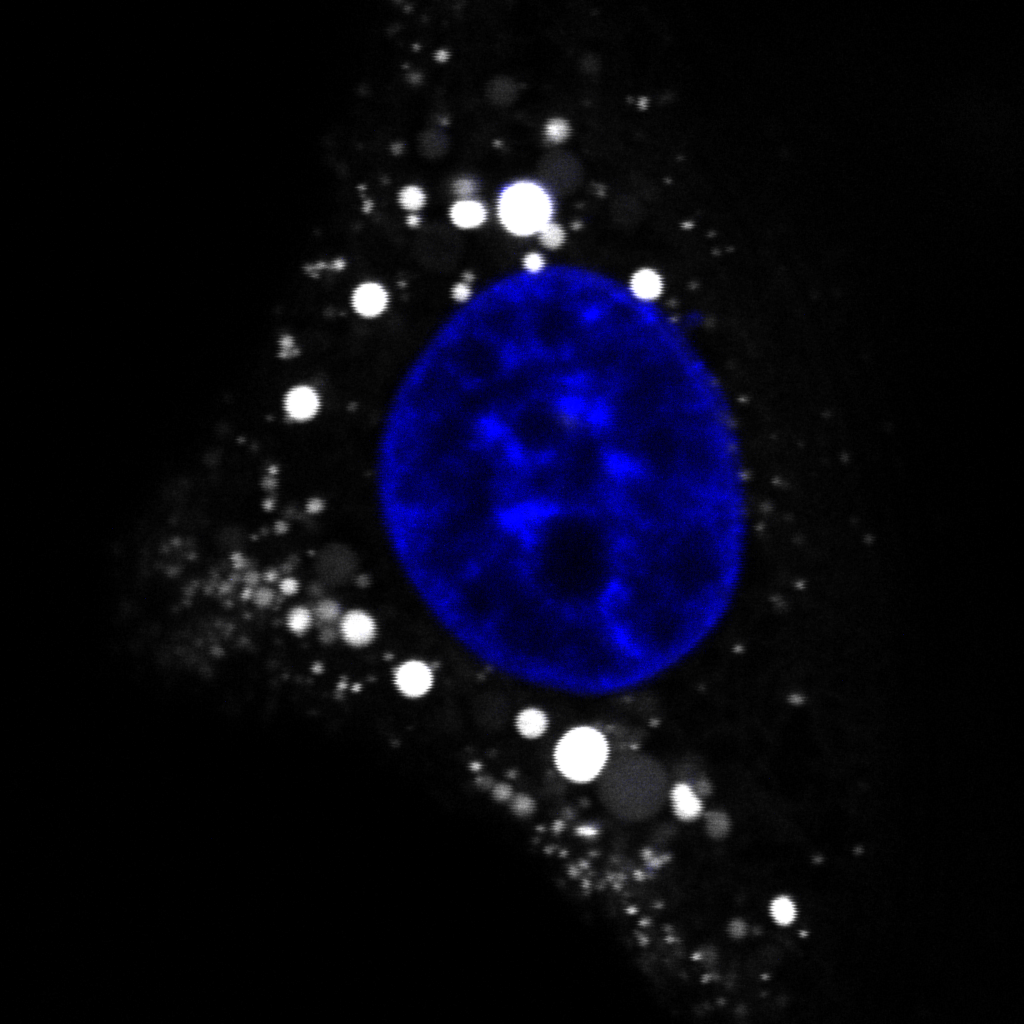

Supplement: Supplementary file 9 — Figure EV1-5 Source Data [file 44318_2025_672_MOESM9_ESM.zip › EV Source Data/EV3/EV3A/LT_merge.tif]

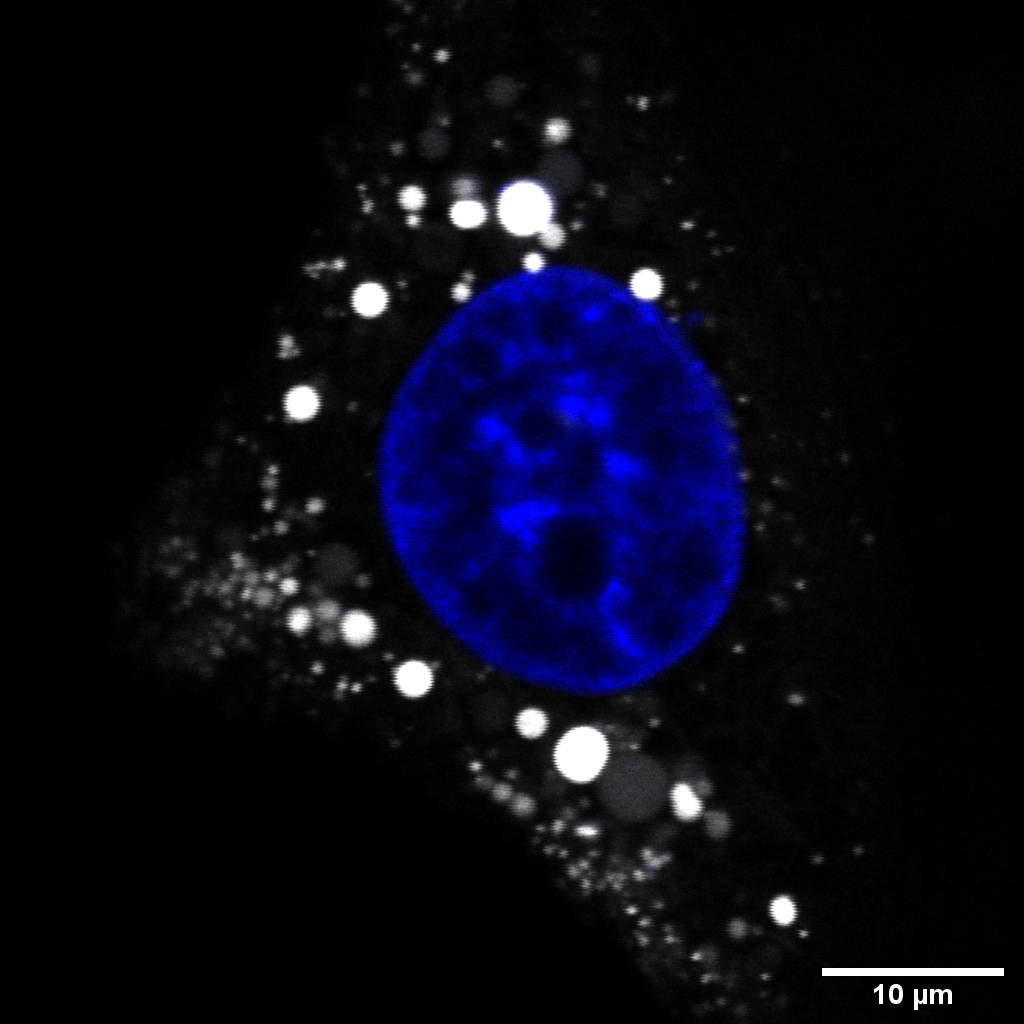

Supplement: Supplementary file 9 — Figure EV1-5 Source Data [file 44318_2025_672_MOESM9_ESM.zip › EV Source Data/EV3/EV3A/LT_scale.tif]

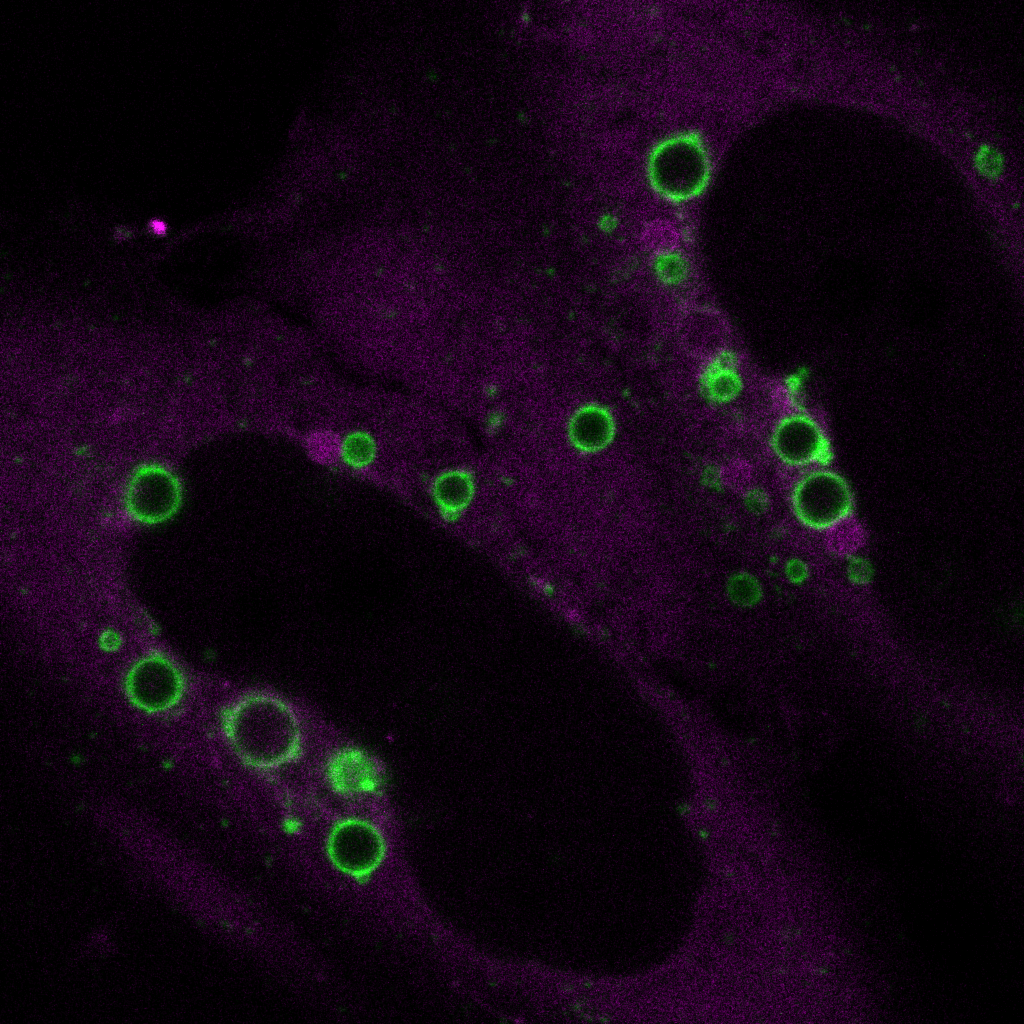

Supplement: Supplementary file 9 — Figure EV1-5 Source Data [file 44318_2025_672_MOESM9_ESM.zip › EV Source Data/EV3/EV3B/0min_merge.tif]

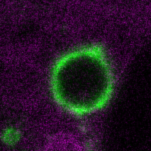

Supplement: Supplementary file 9 — Figure EV1-5 Source Data [file 44318_2025_672_MOESM9_ESM.zip › EV Source Data/EV3/EV3B/0min_merge_a.tif]

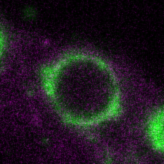

Supplement: Supplementary file 9 — Figure EV1-5 Source Data [file 44318_2025_672_MOESM9_ESM.zip › EV Source Data/EV3/EV3B/0min_merge_b.tif]

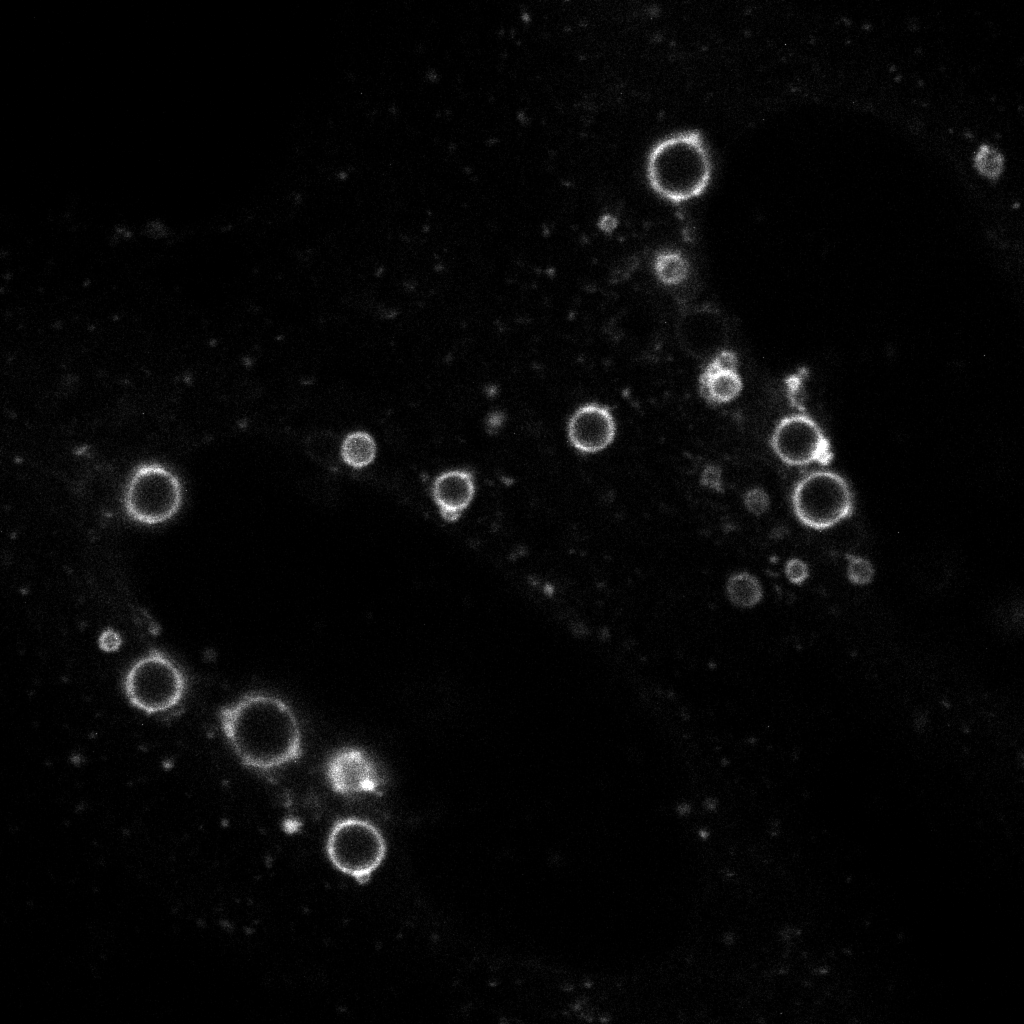

Supplement: Supplementary file 9 — Figure EV1-5 Source Data [file 44318_2025_672_MOESM9_ESM.zip › EV Source Data/EV3/EV3B/0min_Rab5.tif]

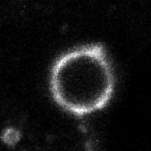

Supplement: Supplementary file 9 — Figure EV1-5 Source Data [file 44318_2025_672_MOESM9_ESM.zip › EV Source Data/EV3/EV3B/0min_Rab5_a.tif]

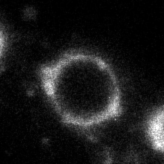

Supplement: Supplementary file 9 — Figure EV1-5 Source Data [file 44318_2025_672_MOESM9_ESM.zip › EV Source Data/EV3/EV3B/0min_Rab5_b.tif]

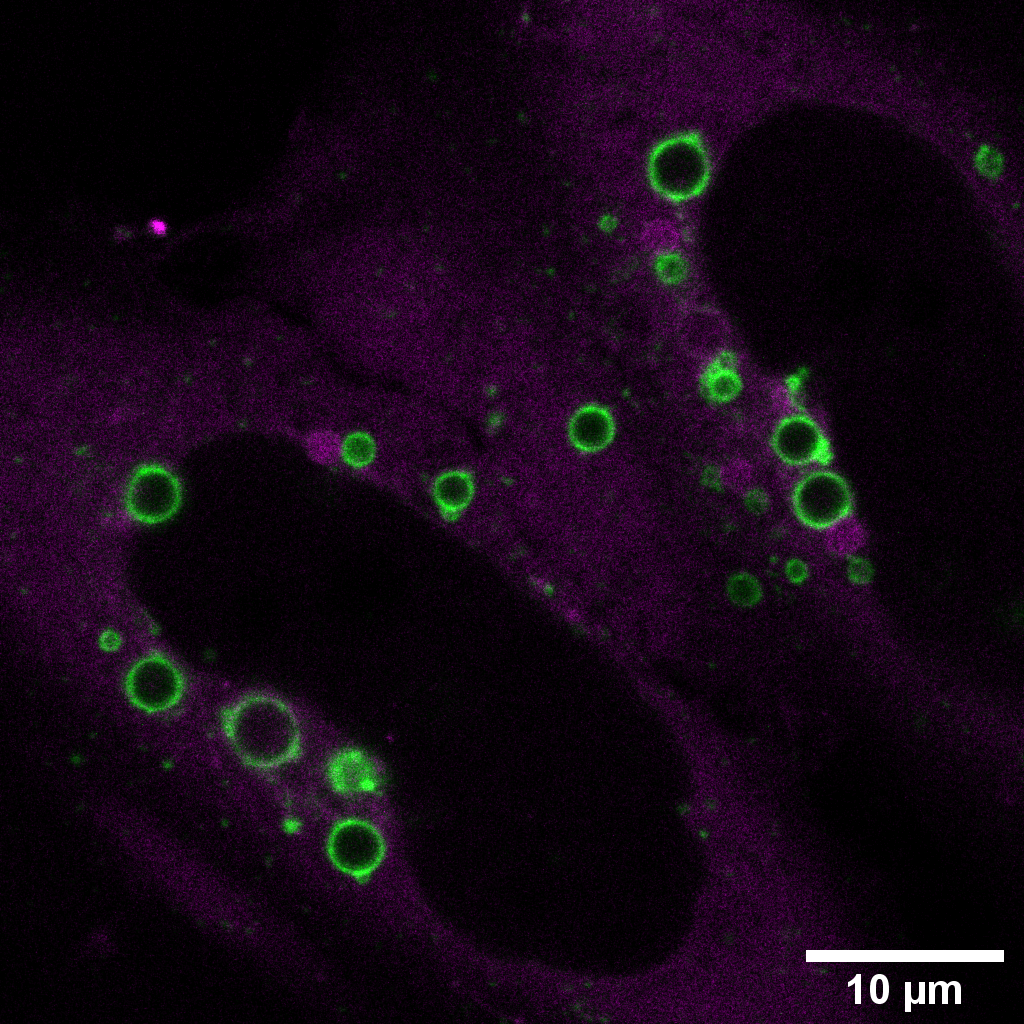

Supplement: Supplementary file 9 — Figure EV1-5 Source Data [file 44318_2025_672_MOESM9_ESM.zip › EV Source Data/EV3/EV3B/0min_scale.tif]

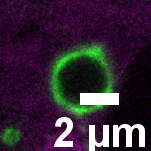

Supplement: Supplementary file 9 — Figure EV1-5 Source Data [file 44318_2025_672_MOESM9_ESM.zip › EV Source Data/EV3/EV3B/0min_scale_a.tif]

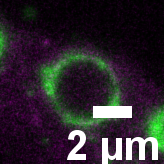

Supplement: Supplementary file 9 — Figure EV1-5 Source Data [file 44318_2025_672_MOESM9_ESM.zip › EV Source Data/EV3/EV3B/0min_scale_b.tif]

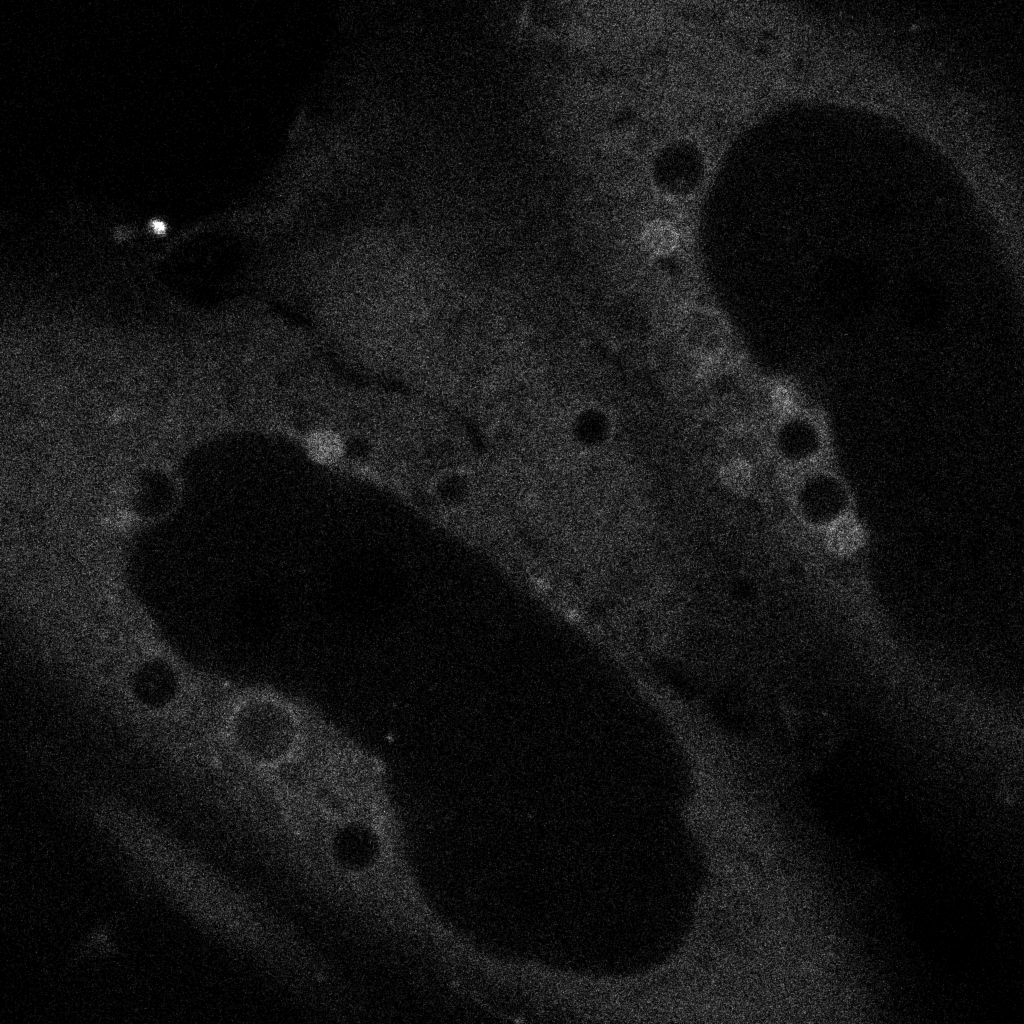

Supplement: Supplementary file 9 — Figure EV1-5 Source Data [file 44318_2025_672_MOESM9_ESM.zip › EV Source Data/EV3/EV3B/0min_TECPR1.tif]

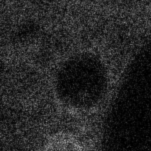

Supplement: Supplementary file 9 — Figure EV1-5 Source Data [file 44318_2025_672_MOESM9_ESM.zip › EV Source Data/EV3/EV3B/0min_TECPR1_a.tif]

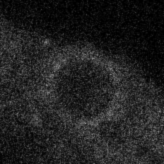

Supplement: Supplementary file 9 — Figure EV1-5 Source Data [file 44318_2025_672_MOESM9_ESM.zip › EV Source Data/EV3/EV3B/0min_TECPR1_b.tif]

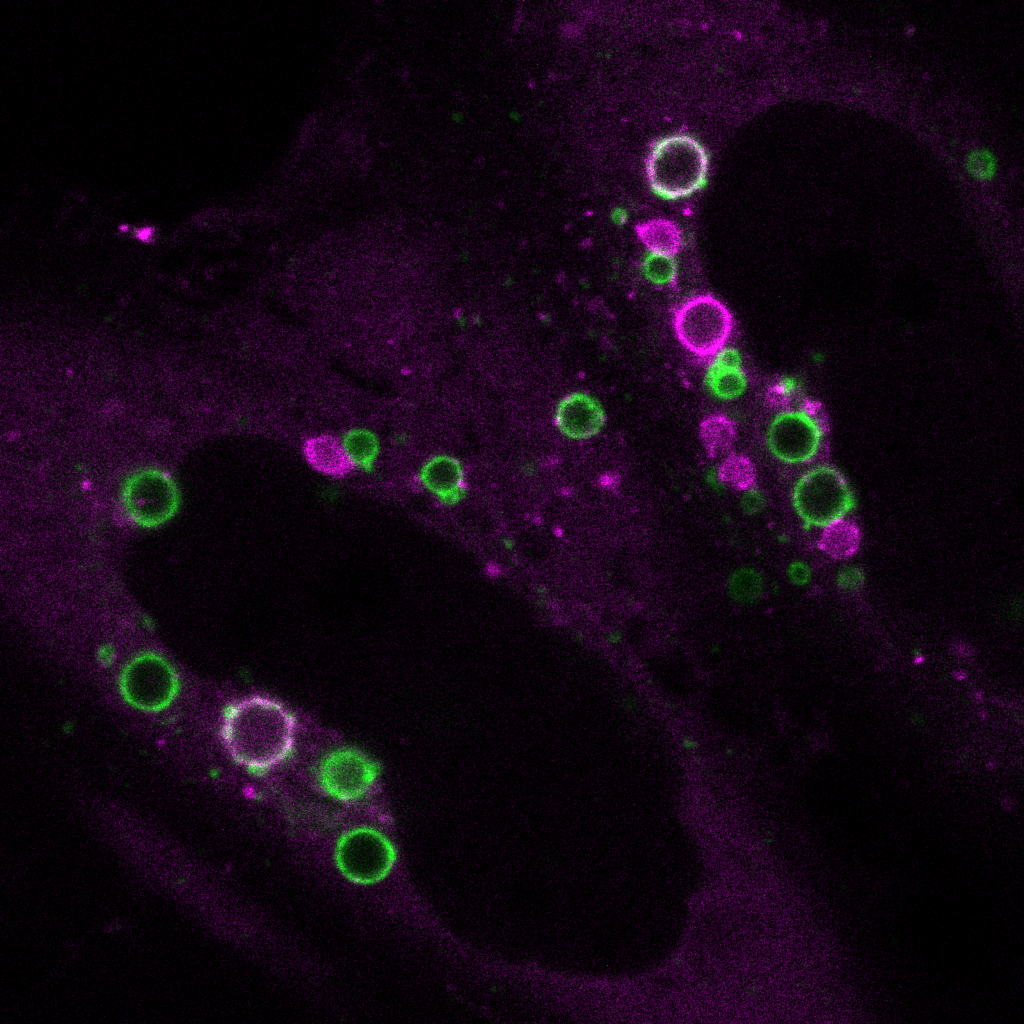

Supplement: Supplementary file 9 — Figure EV1-5 Source Data [file 44318_2025_672_MOESM9_ESM.zip › EV Source Data/EV3/EV3B/10min_merge.tif]

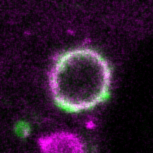

Supplement: Supplementary file 9 — Figure EV1-5 Source Data [file 44318_2025_672_MOESM9_ESM.zip › EV Source Data/EV3/EV3B/10min_merge_a.tif]

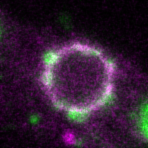

Supplement: Supplementary file 9 — Figure EV1-5 Source Data [file 44318_2025_672_MOESM9_ESM.zip › EV Source Data/EV3/EV3B/10min_merge_b.tif]

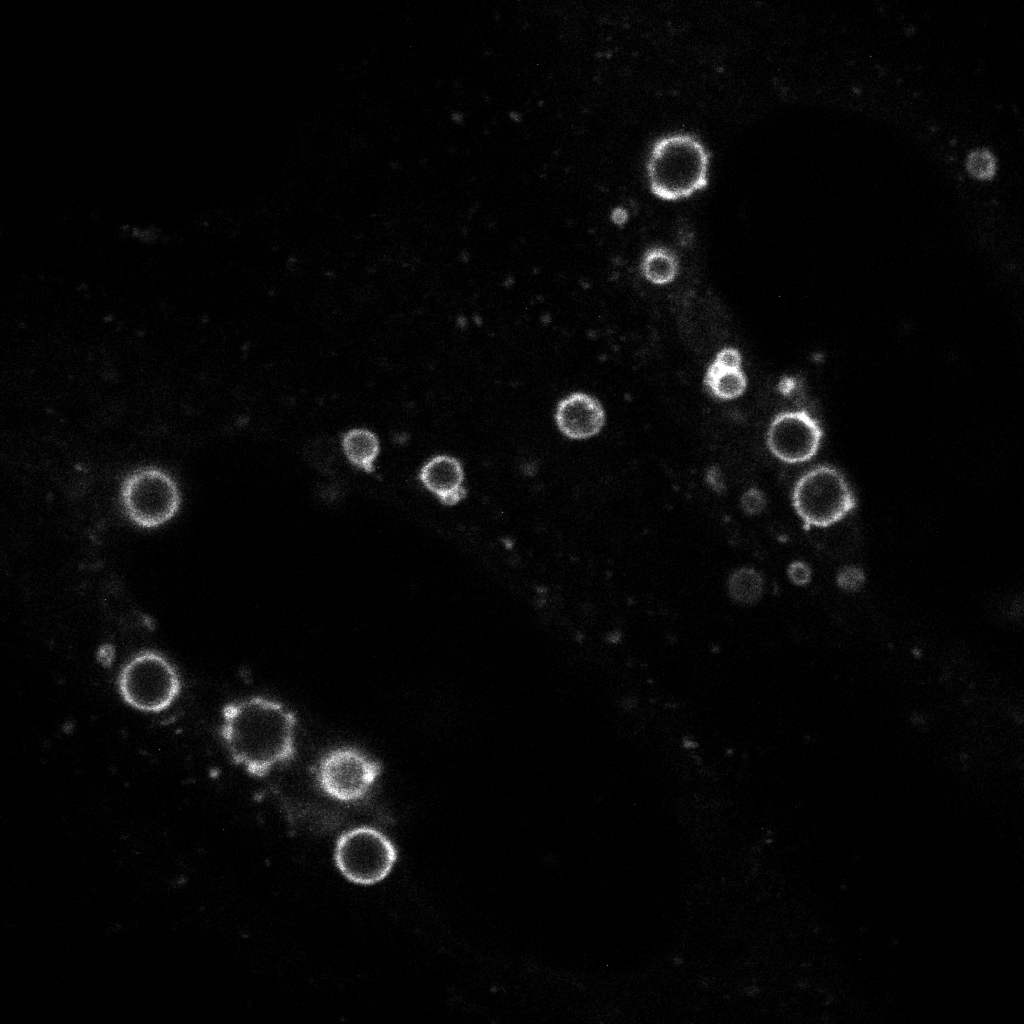

Supplement: Supplementary file 9 — Figure EV1-5 Source Data [file 44318_2025_672_MOESM9_ESM.zip › EV Source Data/EV3/EV3B/10min_Rab5.tif]

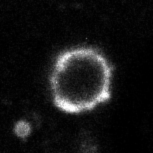

Supplement: Supplementary file 9 — Figure EV1-5 Source Data [file 44318_2025_672_MOESM9_ESM.zip › EV Source Data/EV3/EV3B/10min_Rab5_a.tif]

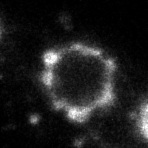

Supplement: Supplementary file 9 — Figure EV1-5 Source Data [file 44318_2025_672_MOESM9_ESM.zip › EV Source Data/EV3/EV3B/10min_Rab5_b.tif]

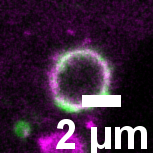

Supplement: Supplementary file 9 — Figure EV1-5 Source Data [file 44318_2025_672_MOESM9_ESM.zip › EV Source Data/EV3/EV3B/10min_scale_a.tif]

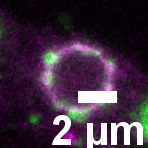

Supplement: Supplementary file 9 — Figure EV1-5 Source Data [file 44318_2025_672_MOESM9_ESM.zip › EV Source Data/EV3/EV3B/10min_scale_b.tif]

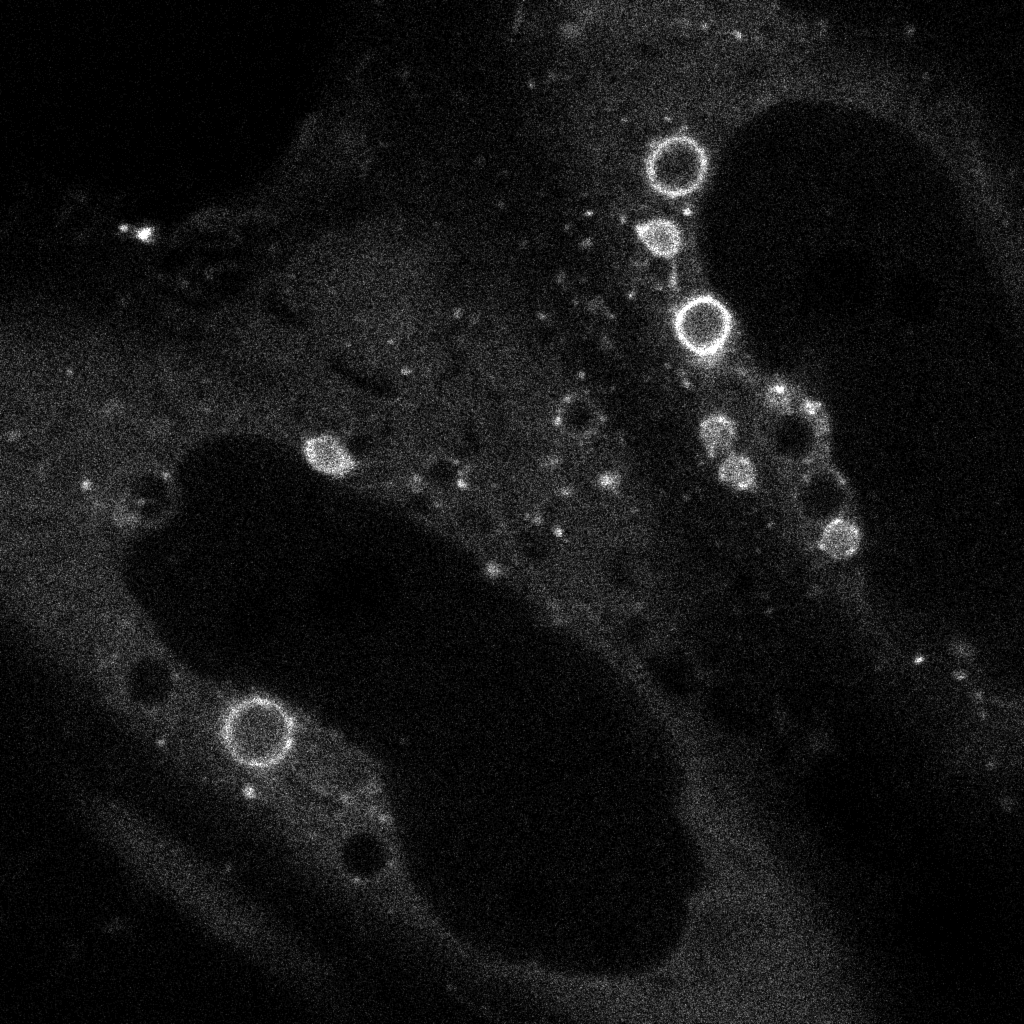

Supplement: Supplementary file 9 — Figure EV1-5 Source Data [file 44318_2025_672_MOESM9_ESM.zip › EV Source Data/EV3/EV3B/10min_TECPR1.tif]

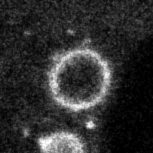

Supplement: Supplementary file 9 — Figure EV1-5 Source Data [file 44318_2025_672_MOESM9_ESM.zip › EV Source Data/EV3/EV3B/10min_TECPR1_a.tif]

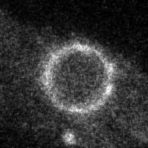

Supplement: Supplementary file 9 — Figure EV1-5 Source Data [file 44318_2025_672_MOESM9_ESM.zip › EV Source Data/EV3/EV3B/10min_TECPR1_b.tif]

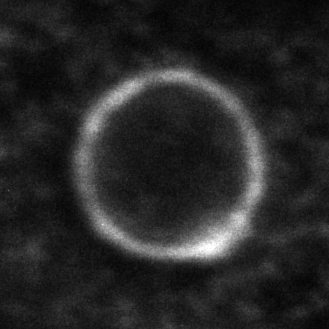

Supplement: Supplementary file 9 — Figure EV1-5 Source Data [file 44318_2025_672_MOESM9_ESM.zip › EV Source Data/EV3/EV3D/ALG2/ALG2.tif]

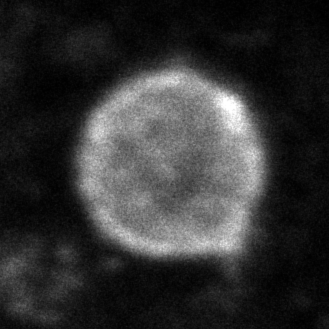

Supplement: Supplementary file 9 — Figure EV1-5 Source Data [file 44318_2025_672_MOESM9_ESM.zip › EV Source Data/EV3/EV3D/ALG2/LAMP.tif]

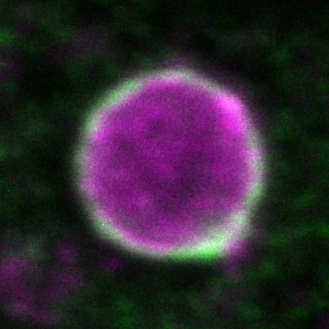

Supplement: Supplementary file 9 — Figure EV1-5 Source Data [file 44318_2025_672_MOESM9_ESM.zip › EV Source Data/EV3/EV3D/ALG2/Merge.tif]

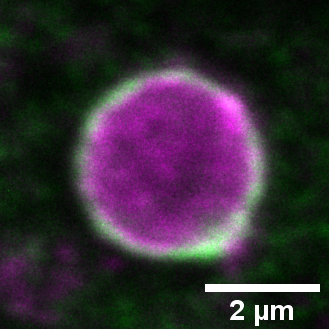

Supplement: Supplementary file 9 — Figure EV1-5 Source Data [file 44318_2025_672_MOESM9_ESM.zip › EV Source Data/EV3/EV3D/ALG2/Scale.tif]

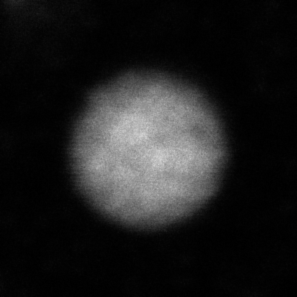

Supplement: Supplementary file 9 — Figure EV1-5 Source Data [file 44318_2025_672_MOESM9_ESM.zip › EV Source Data/EV3/EV3D/Gal3/Gal3.tif]

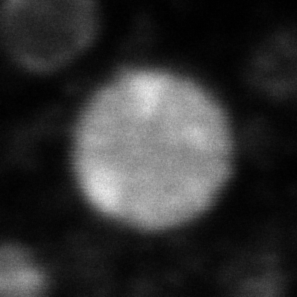

Supplement: Supplementary file 9 — Figure EV1-5 Source Data [file 44318_2025_672_MOESM9_ESM.zip › EV Source Data/EV3/EV3D/Gal3/LAMP.tif]

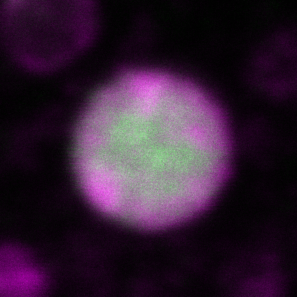

Supplement: Supplementary file 9 — Figure EV1-5 Source Data [file 44318_2025_672_MOESM9_ESM.zip › EV Source Data/EV3/EV3D/Gal3/merge.tif]

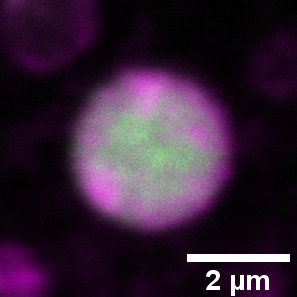

Supplement: Supplementary file 9 — Figure EV1-5 Source Data [file 44318_2025_672_MOESM9_ESM.zip › EV Source Data/EV3/EV3D/Gal3/scale.tif]

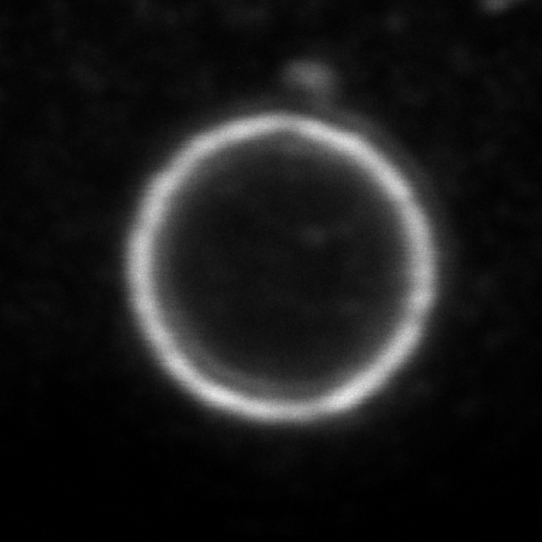

Supplement: Supplementary file 9 — Figure EV1-5 Source Data [file 44318_2025_672_MOESM9_ESM.zip › EV Source Data/EV3/EV3D/IST1/IST1.tif]

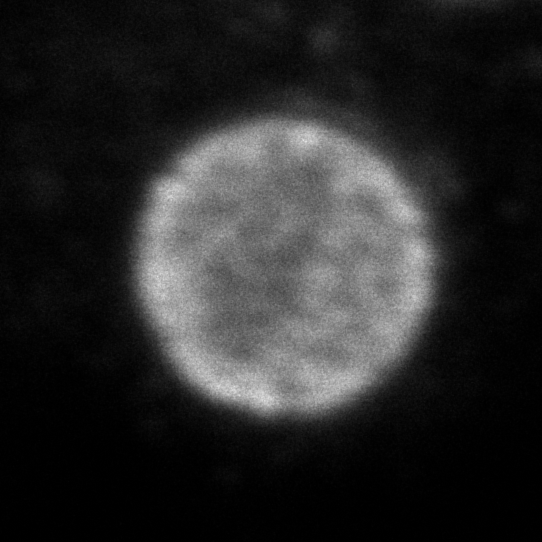

Supplement: Supplementary file 9 — Figure EV1-5 Source Data [file 44318_2025_672_MOESM9_ESM.zip › EV Source Data/EV3/EV3D/IST1/LAMP.tif]

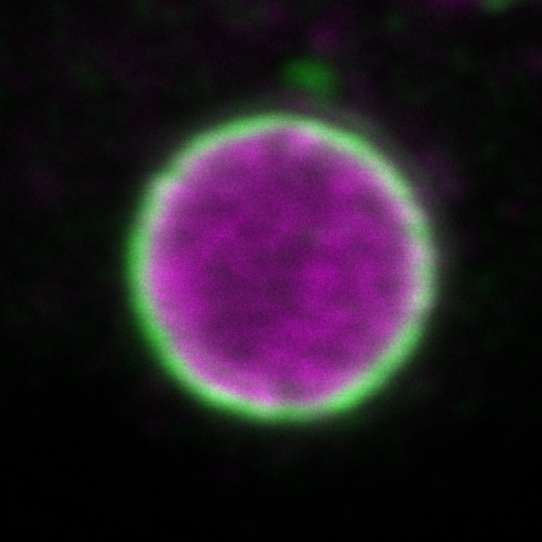

Supplement: Supplementary file 9 — Figure EV1-5 Source Data [file 44318_2025_672_MOESM9_ESM.zip › EV Source Data/EV3/EV3D/IST1/Merge.tif]

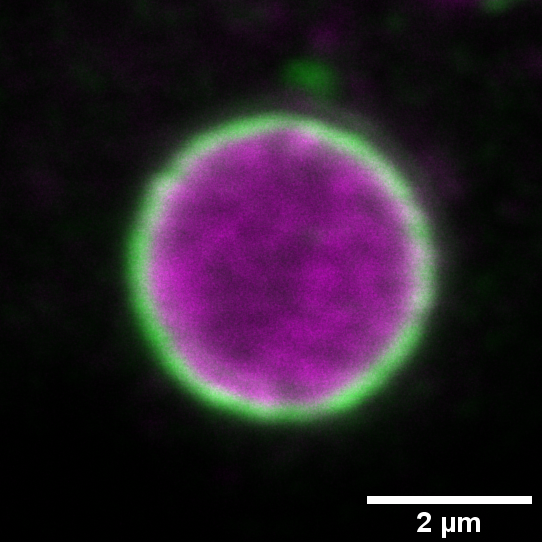

Supplement: Supplementary file 9 — Figure EV1-5 Source Data [file 44318_2025_672_MOESM9_ESM.zip › EV Source Data/EV3/EV3D/IST1/Scale.tif]

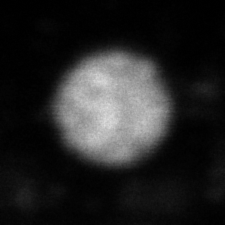

Supplement: Supplementary file 9 — Figure EV1-5 Source Data [file 44318_2025_672_MOESM9_ESM.zip › EV Source Data/EV3/EV3D/LC3/LAMP.tif]

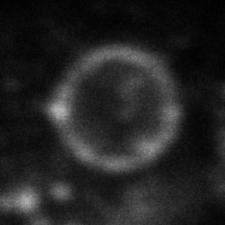

Supplement: Supplementary file 9 — Figure EV1-5 Source Data [file 44318_2025_672_MOESM9_ESM.zip › EV Source Data/EV3/EV3D/LC3/LC3.tif]

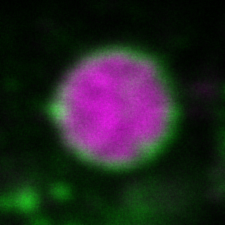

Supplement: Supplementary file 9 — Figure EV1-5 Source Data [file 44318_2025_672_MOESM9_ESM.zip › EV Source Data/EV3/EV3D/LC3/Merge.tif]

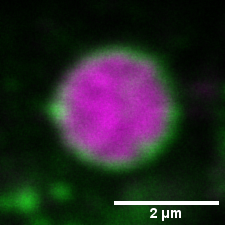

Supplement: Supplementary file 9 — Figure EV1-5 Source Data [file 44318_2025_672_MOESM9_ESM.zip › EV Source Data/EV3/EV3D/LC3/scale.tif]

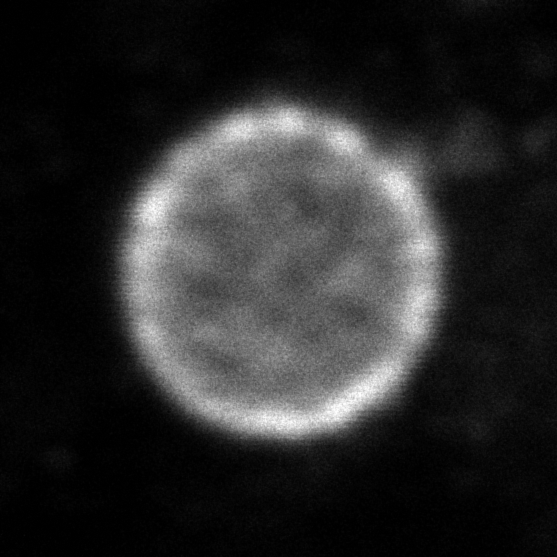

Supplement: Supplementary file 9 — Figure EV1-5 Source Data [file 44318_2025_672_MOESM9_ESM.zip › EV Source Data/EV3/EV3D/TECPR1/LAMP.tif]

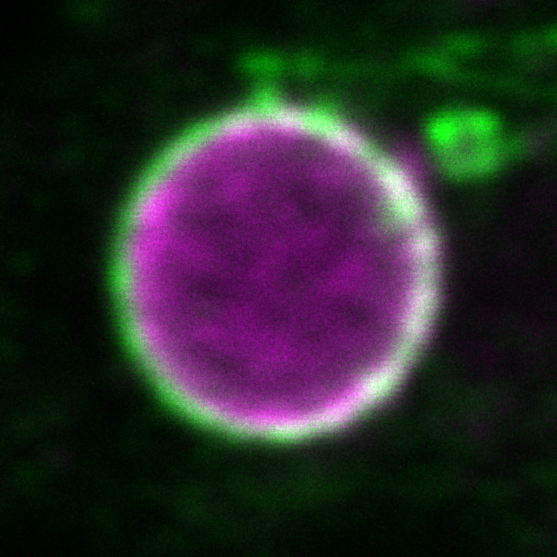

Supplement: Supplementary file 9 — Figure EV1-5 Source Data [file 44318_2025_672_MOESM9_ESM.zip › EV Source Data/EV3/EV3D/TECPR1/Merge.tif]

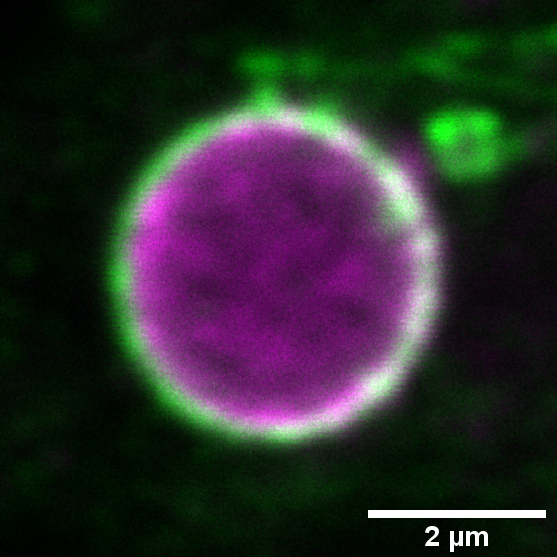

Supplement: Supplementary file 9 — Figure EV1-5 Source Data [file 44318_2025_672_MOESM9_ESM.zip › EV Source Data/EV3/EV3D/TECPR1/Scale.tif]

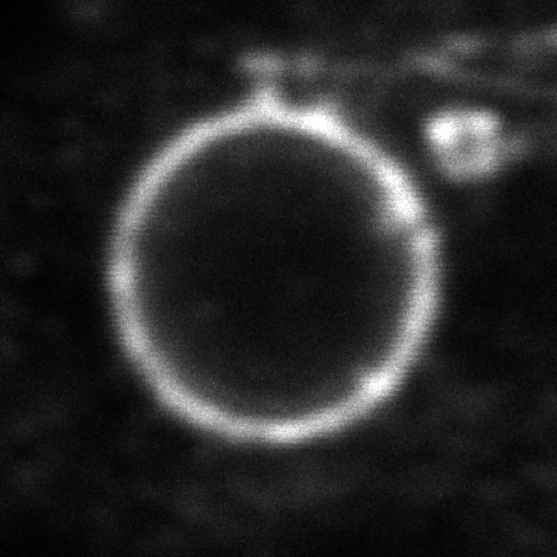

Supplement: Supplementary file 9 — Figure EV1-5 Source Data [file 44318_2025_672_MOESM9_ESM.zip › EV Source Data/EV3/EV3D/TECPR1/TECPR1.tif]

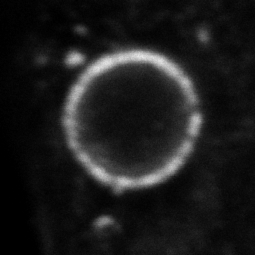

Supplement: Supplementary file 9 — Figure EV1-5 Source Data [file 44318_2025_672_MOESM9_ESM.zip › EV Source Data/EV3/EV3E/16KO_LLOMe_IST1.tif]

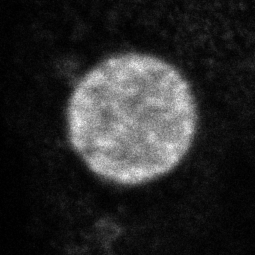

Supplement: Supplementary file 9 — Figure EV1-5 Source Data [file 44318_2025_672_MOESM9_ESM.zip › EV Source Data/EV3/EV3E/16KO_LLOMe_LAMP.tif]

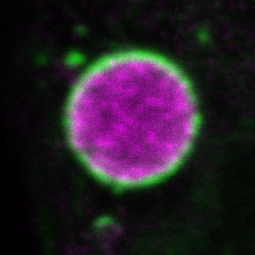

Supplement: Supplementary file 9 — Figure EV1-5 Source Data [file 44318_2025_672_MOESM9_ESM.zip › EV Source Data/EV3/EV3E/16KO_LLOMe_Merge.tif]

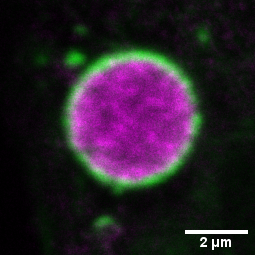

Supplement: Supplementary file 9 — Figure EV1-5 Source Data [file 44318_2025_672_MOESM9_ESM.zip › EV Source Data/EV3/EV3E/16KO_LLOMe_Scale.tif]

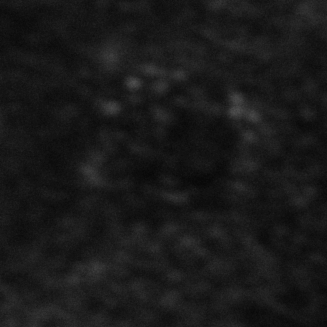

Supplement: Supplementary file 9 — Figure EV1-5 Source Data [file 44318_2025_672_MOESM9_ESM.zip › EV Source Data/EV3/EV3E/16KO_VEH_IST1.tif]

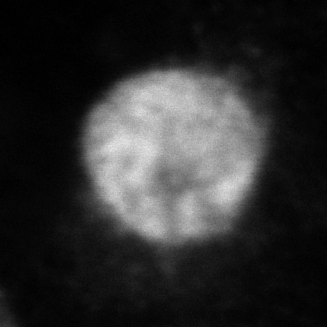

Supplement: Supplementary file 9 — Figure EV1-5 Source Data [file 44318_2025_672_MOESM9_ESM.zip › EV Source Data/EV3/EV3E/16KO_VEH_LAMP.tif]

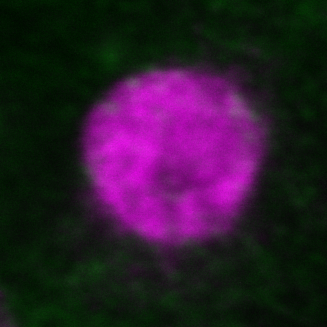

Supplement: Supplementary file 9 — Figure EV1-5 Source Data [file 44318_2025_672_MOESM9_ESM.zip › EV Source Data/EV3/EV3E/16KO_VEH_Merge.tif]

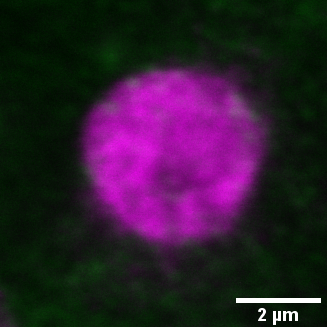

Supplement: Supplementary file 9 — Figure EV1-5 Source Data [file 44318_2025_672_MOESM9_ESM.zip › EV Source Data/EV3/EV3E/16KO_VEH_Scale.tif]

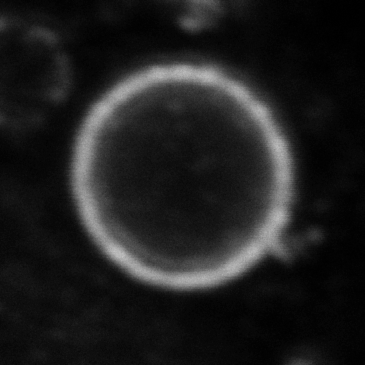

Supplement: Supplementary file 9 — Figure EV1-5 Source Data [file 44318_2025_672_MOESM9_ESM.zip › EV Source Data/EV3/EV3E/TECKO_LLOMe_IST1.tif]

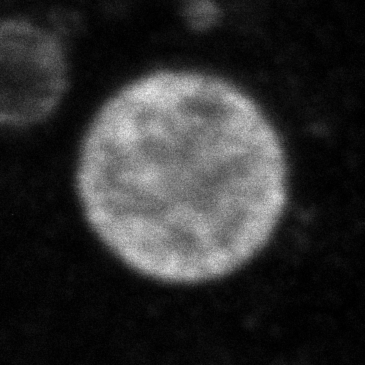

Supplement: Supplementary file 9 — Figure EV1-5 Source Data [file 44318_2025_672_MOESM9_ESM.zip › EV Source Data/EV3/EV3E/TECKO_LLOMe_LAMP.tif]

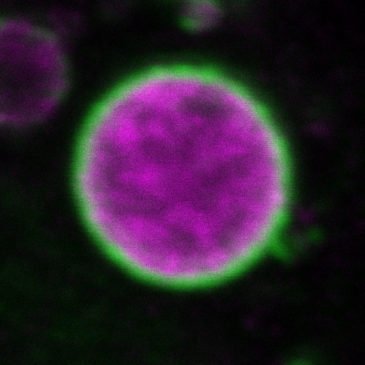

Supplement: Supplementary file 9 — Figure EV1-5 Source Data [file 44318_2025_672_MOESM9_ESM.zip › EV Source Data/EV3/EV3E/TECKO_LLOMe_Merge.tif]

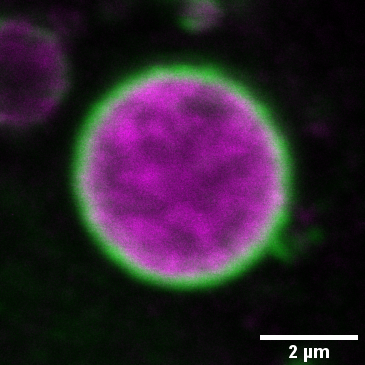

Supplement: Supplementary file 9 — Figure EV1-5 Source Data [file 44318_2025_672_MOESM9_ESM.zip › EV Source Data/EV3/EV3E/TECKO_LLOMe_Scale.tif]

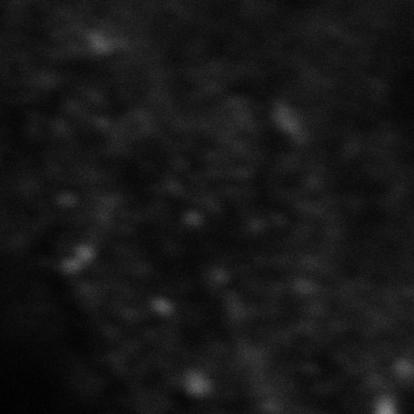

Supplement: Supplementary file 9 — Figure EV1-5 Source Data [file 44318_2025_672_MOESM9_ESM.zip › EV Source Data/EV3/EV3E/TECKO_VEH_IST1.tif]

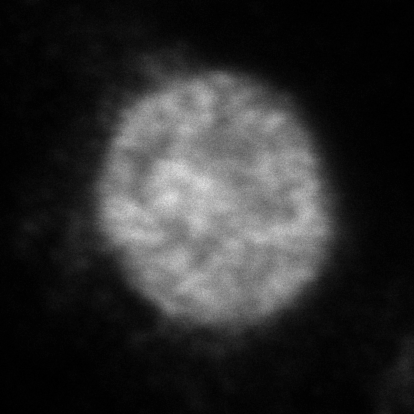

Supplement: Supplementary file 9 — Figure EV1-5 Source Data [file 44318_2025_672_MOESM9_ESM.zip › EV Source Data/EV3/EV3E/TECKO_VEH_LAMP.tif]

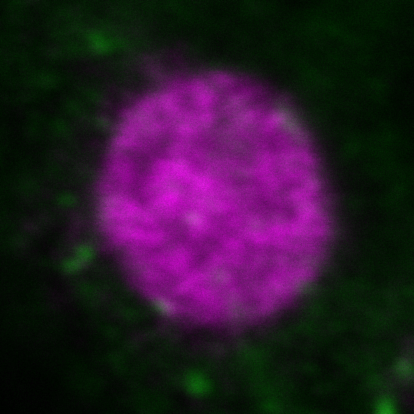

Supplement: Supplementary file 9 — Figure EV1-5 Source Data [file 44318_2025_672_MOESM9_ESM.zip › EV Source Data/EV3/EV3E/TECKO_VEH_Merge.tif]

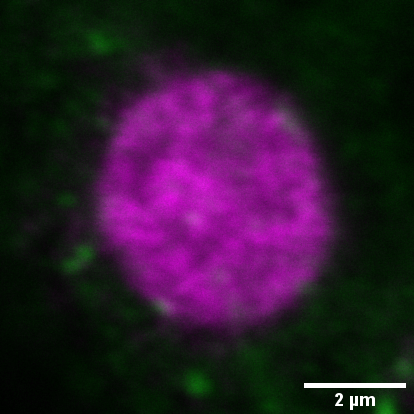

Supplement: Supplementary file 9 — Figure EV1-5 Source Data [file 44318_2025_672_MOESM9_ESM.zip › EV Source Data/EV3/EV3E/TECKO_VEH_Scale.tif]

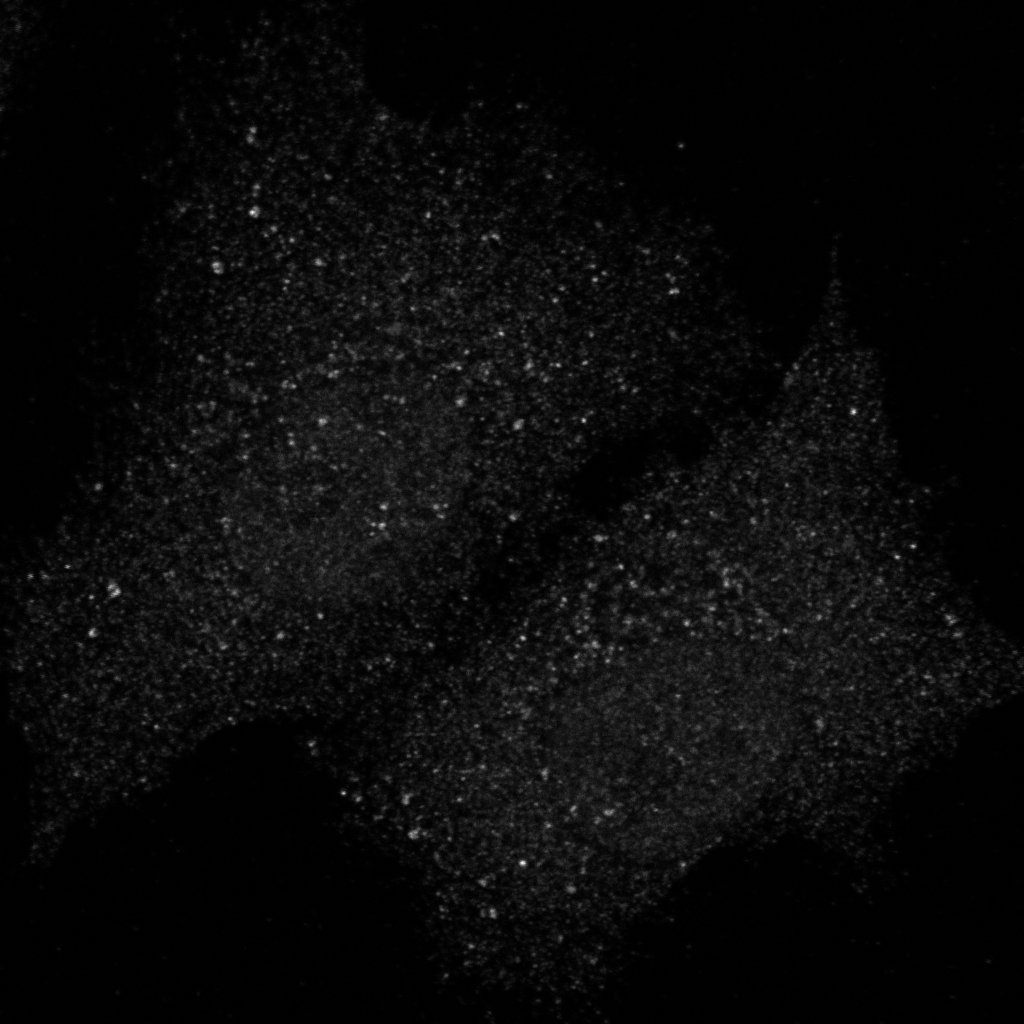

Supplement: Supplementary file 9 — Figure EV1-5 Source Data [file 44318_2025_672_MOESM9_ESM.zip › EV Source Data/EV4/EV4A/5KO_LLOMe_BAPTA_CHMP2A.tif]

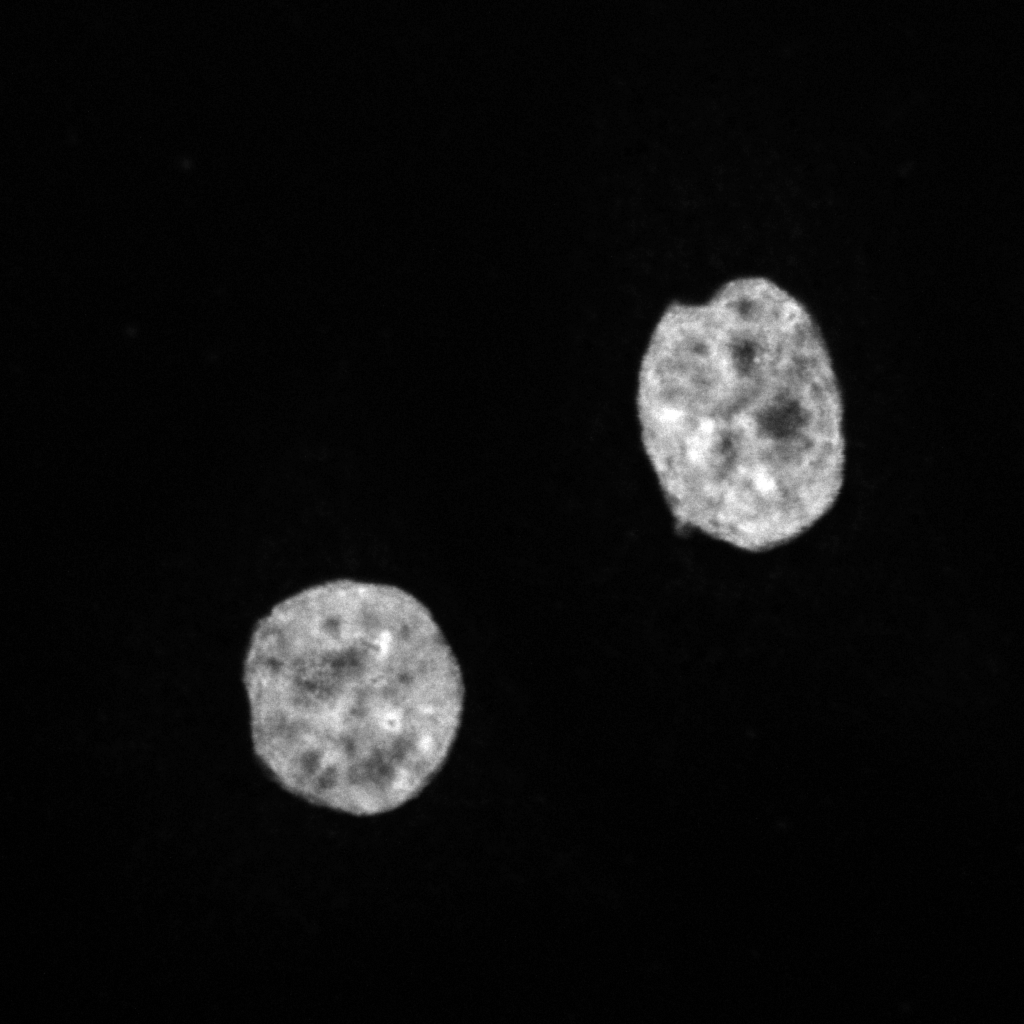

Supplement: Supplementary file 9 — Figure EV1-5 Source Data [file 44318_2025_672_MOESM9_ESM.zip › EV Source Data/EV4/EV4A/5KO_LLOMe_BAPTA_DAPI.tif]

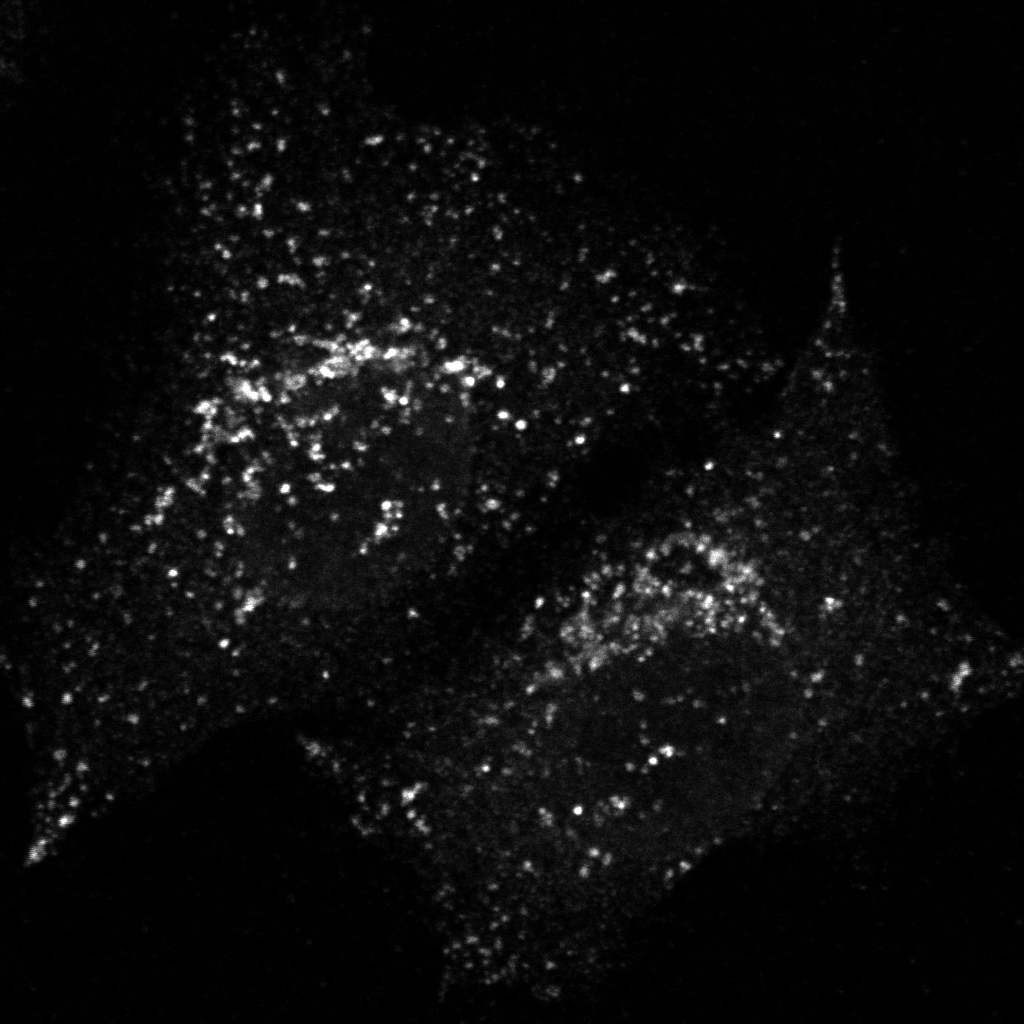

Supplement: Supplementary file 9 — Figure EV1-5 Source Data [file 44318_2025_672_MOESM9_ESM.zip › EV Source Data/EV4/EV4A/5KO_LLOMe_BAPTA_GAL3.tif]

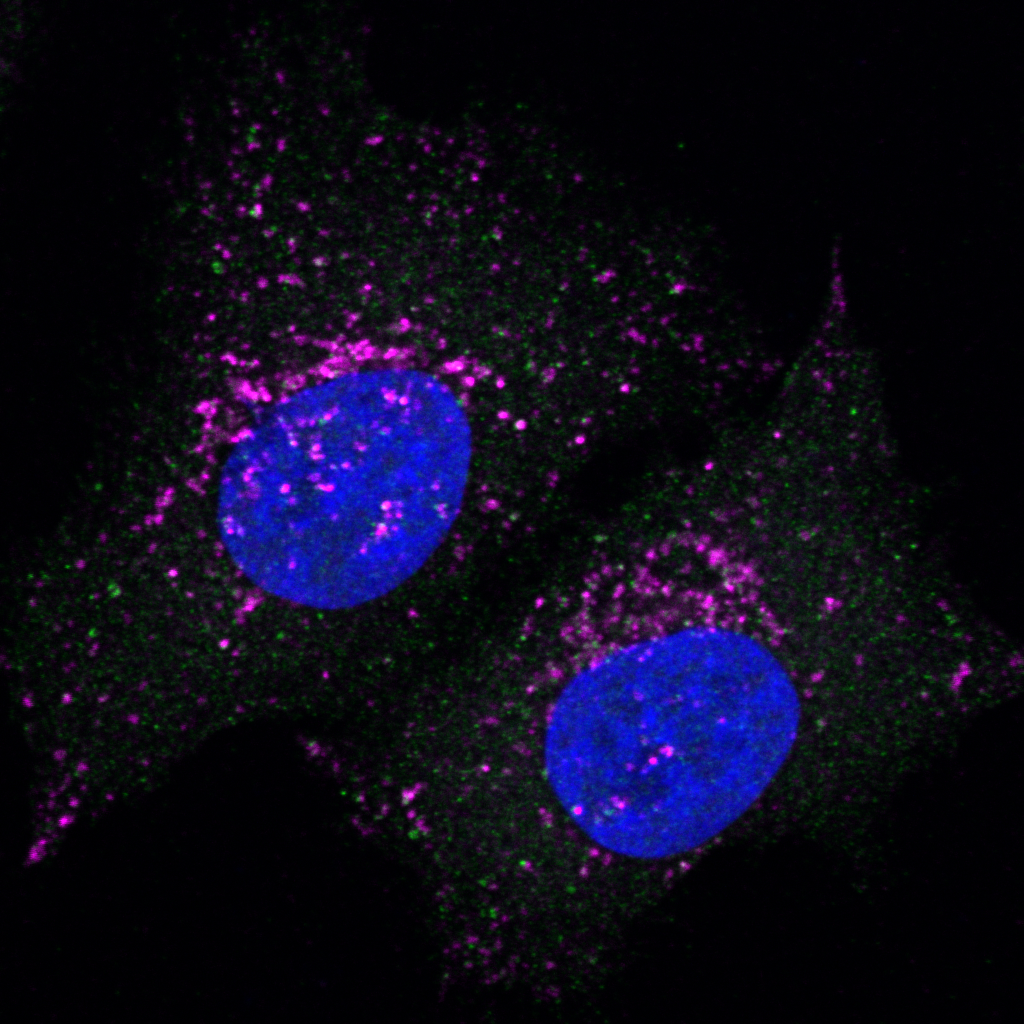

Supplement: Supplementary file 9 — Figure EV1-5 Source Data [file 44318_2025_672_MOESM9_ESM.zip › EV Source Data/EV4/EV4A/5KO_LLOMe_BAPTA_merge.tif]

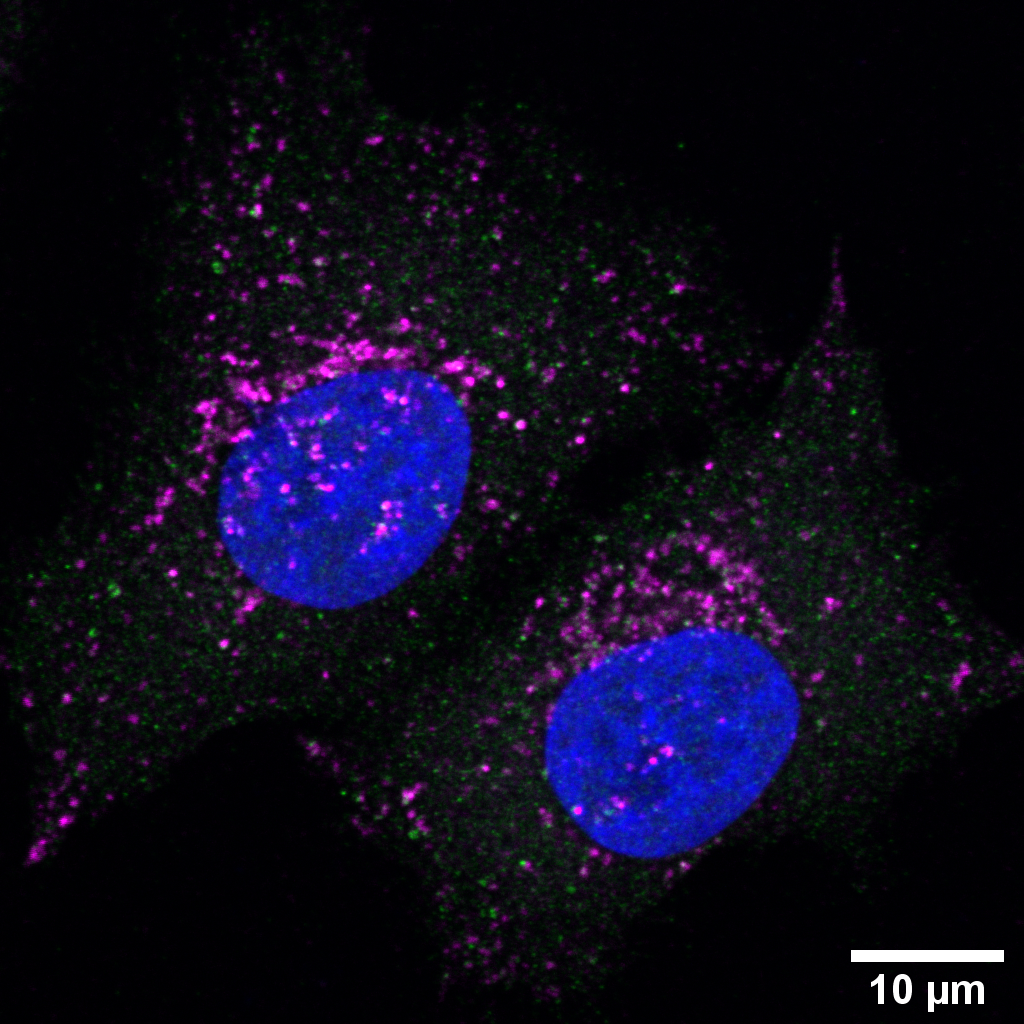

Supplement: Supplementary file 9 — Figure EV1-5 Source Data [file 44318_2025_672_MOESM9_ESM.zip › EV Source Data/EV4/EV4A/5KO_LLOMe_BAPTA_scale.tif]

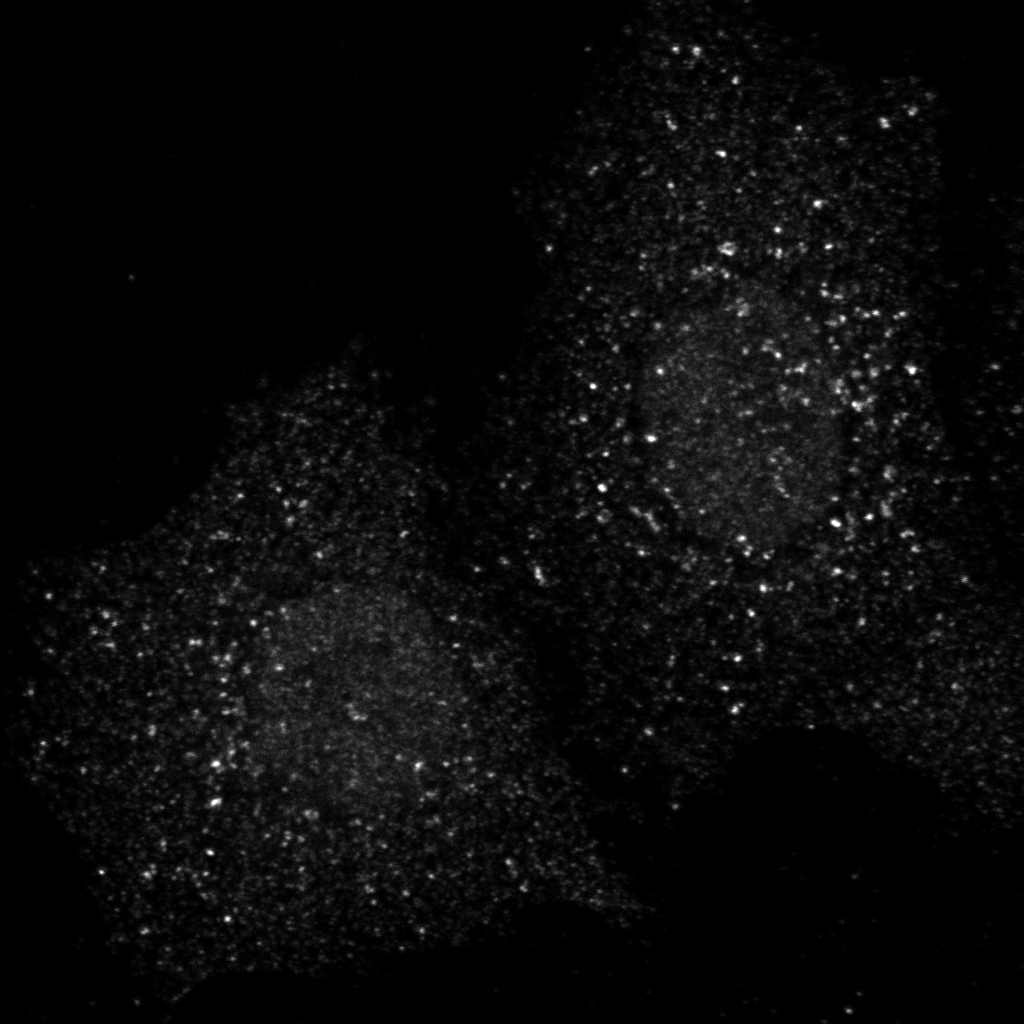

Supplement: Supplementary file 9 — Figure EV1-5 Source Data [file 44318_2025_672_MOESM9_ESM.zip › EV Source Data/EV4/EV4A/5KO_LLOMe_CHMP2A.tif]

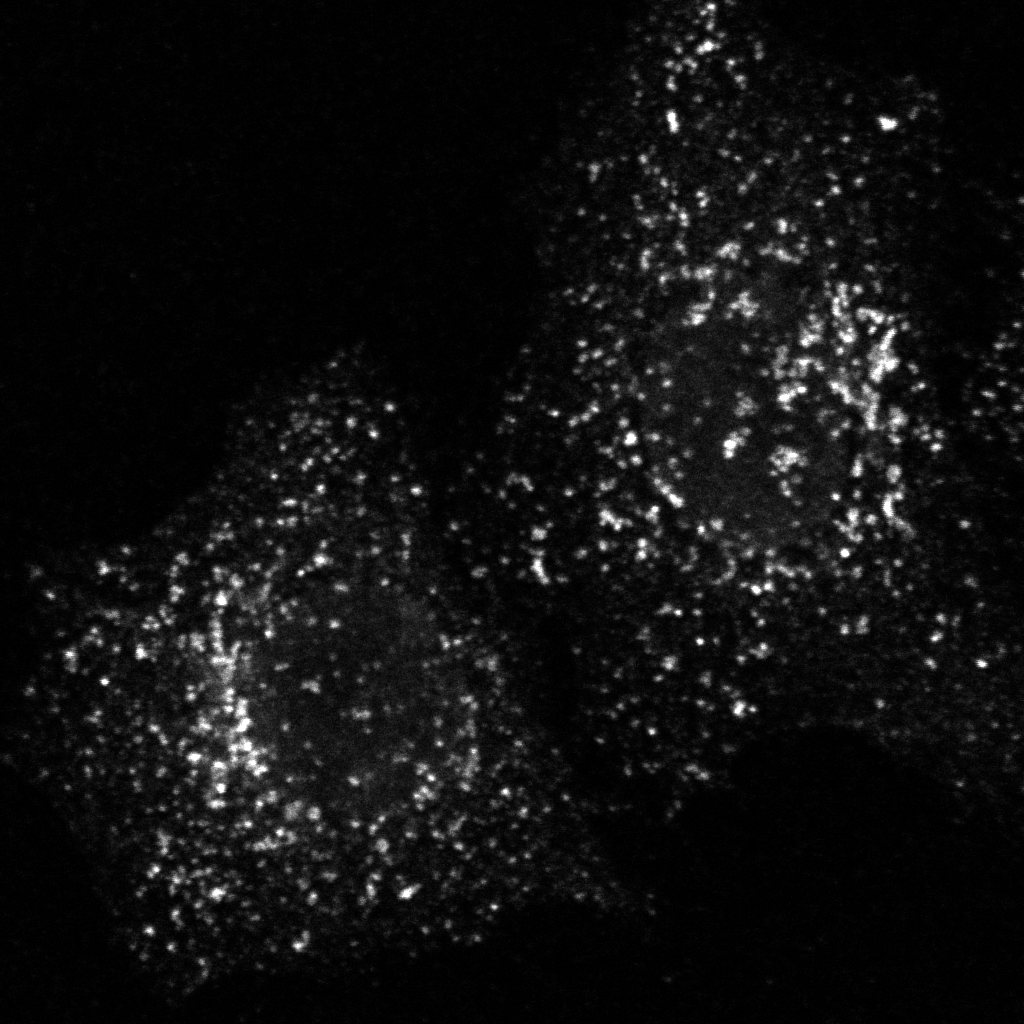

Supplement: Supplementary file 9 — Figure EV1-5 Source Data [file 44318_2025_672_MOESM9_ESM.zip › EV Source Data/EV4/EV4A/5KO_LLOMe_GAL3.tif]

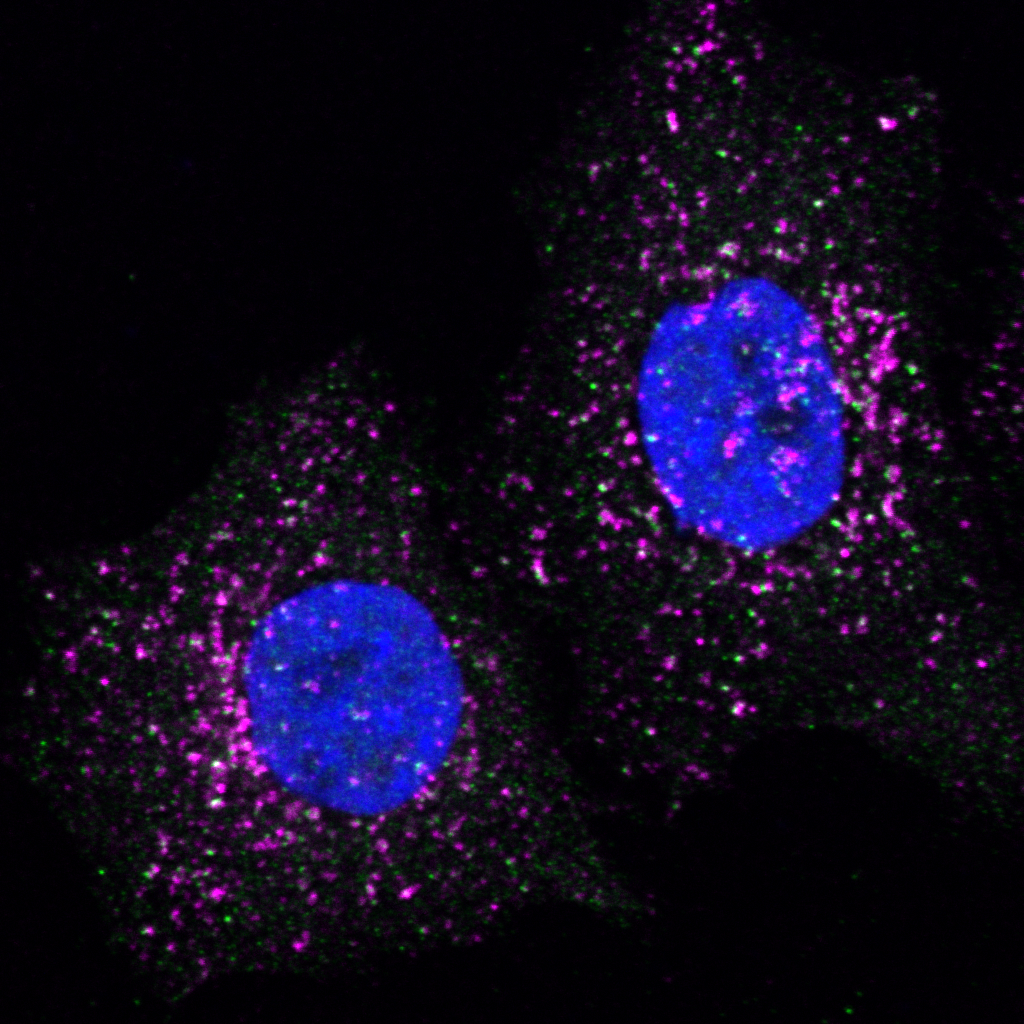

Supplement: Supplementary file 9 — Figure EV1-5 Source Data [file 44318_2025_672_MOESM9_ESM.zip › EV Source Data/EV4/EV4A/5KO_LLOMe_merge.tif]
