# Supplementary material for: The ATG8 E3-like ligases sense lysosomal damage and initiate ESCRT-mediated membrane repair
Source: EMBO J. 2026 Jan 3;45(3):930–52. doi: 10.1038/s44318-025-00672-1 (PMC12865045; doi:10.1038/s44318-025-00672-1)
Supplement: Supplementary file 10 — Expanded View Figures [file 44318_2025_672_MOESM10_ESM.pdf]

Expanded View Figures

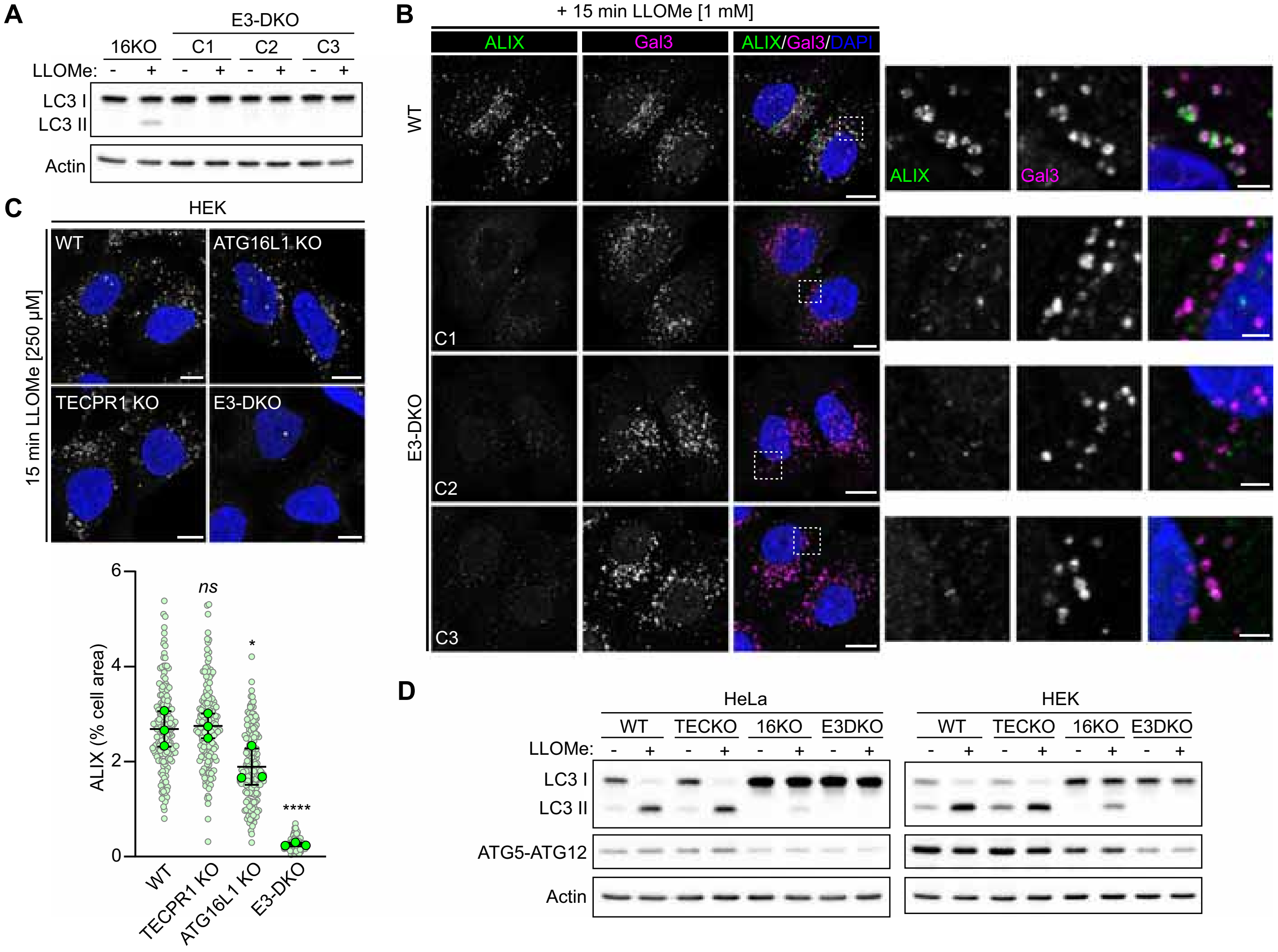

Figure EV1. Validation of ESCRT recruitment deficiency across multiple ATG16L1/TECPR1 DKO clones and cell lines.

(**A**) Western blot analysis of HeLa ATG16L1 KO and three ATG16L1/TECPR1 DKO clones treated with or without 1 mM LLOMe for 30 min. (**B**) Confocal images of cell lines from (**A**) treated with 1 mM LLOMe for 15 min and immunostained for ALIX and Gal3. Nuclei were stained with DAPI. Scale bars = 10 µm for whole image and 2 µm for insets. (**C**) Top: Confocal images of HEK WT, ATG16L1 KO, TECPR1 KO and ATG16L1/TECPR1 DKO cells treated with 1 mM LLOMe for 15 min and immunostained for ALIX. Scale bars = 10 µm. Bottom: Quantification of ALIX area. Small points represent individual cells from three independent experiments. Large points represent the means of individual experiments ($n = 60$ cells per experiment). Bars represent the mean ± SD from the three experiments. Significance was determined from biological replicates using a one-way ANOVA with Tukey's multiple comparisons tests. ns (not significant) represents $P > 0.05$, *$P = 0.0314$, ****$P < 0.0001$. (**D**) Western blot analysis of HeLa and HEK KO cell lines treated with or without 1 mM LLOMe for 30 min. Source data are available online for this figure.

© The Author(s)

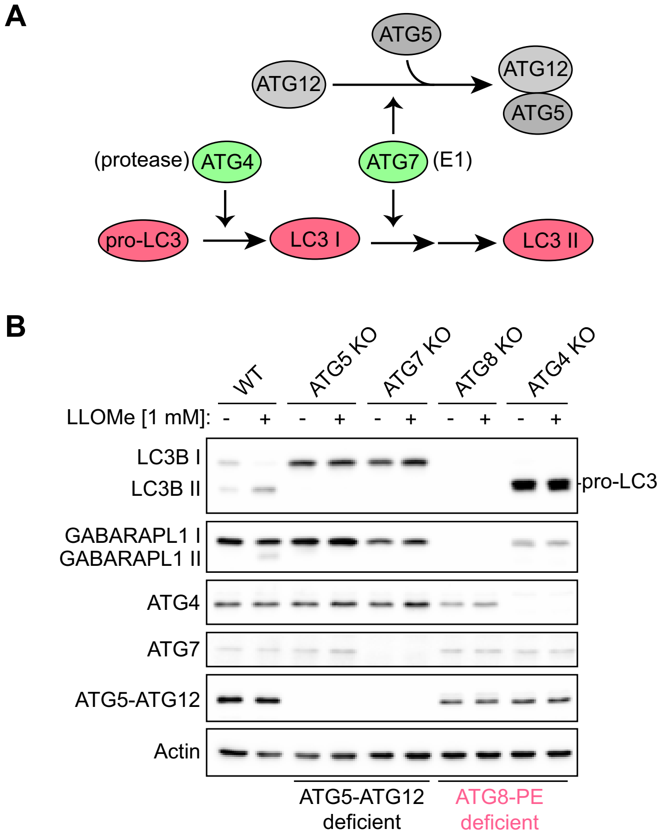

Figure EV2. Effect of ATG KO on ATG5-ATG12 conjugation and ATG8 lipidation.

(**A**) Simplified schematic of ATG8ylation pathway. (**B**) Western blot analysis of ATG KO cell lines. Source data are available online for this figure.

 © The Author(s)

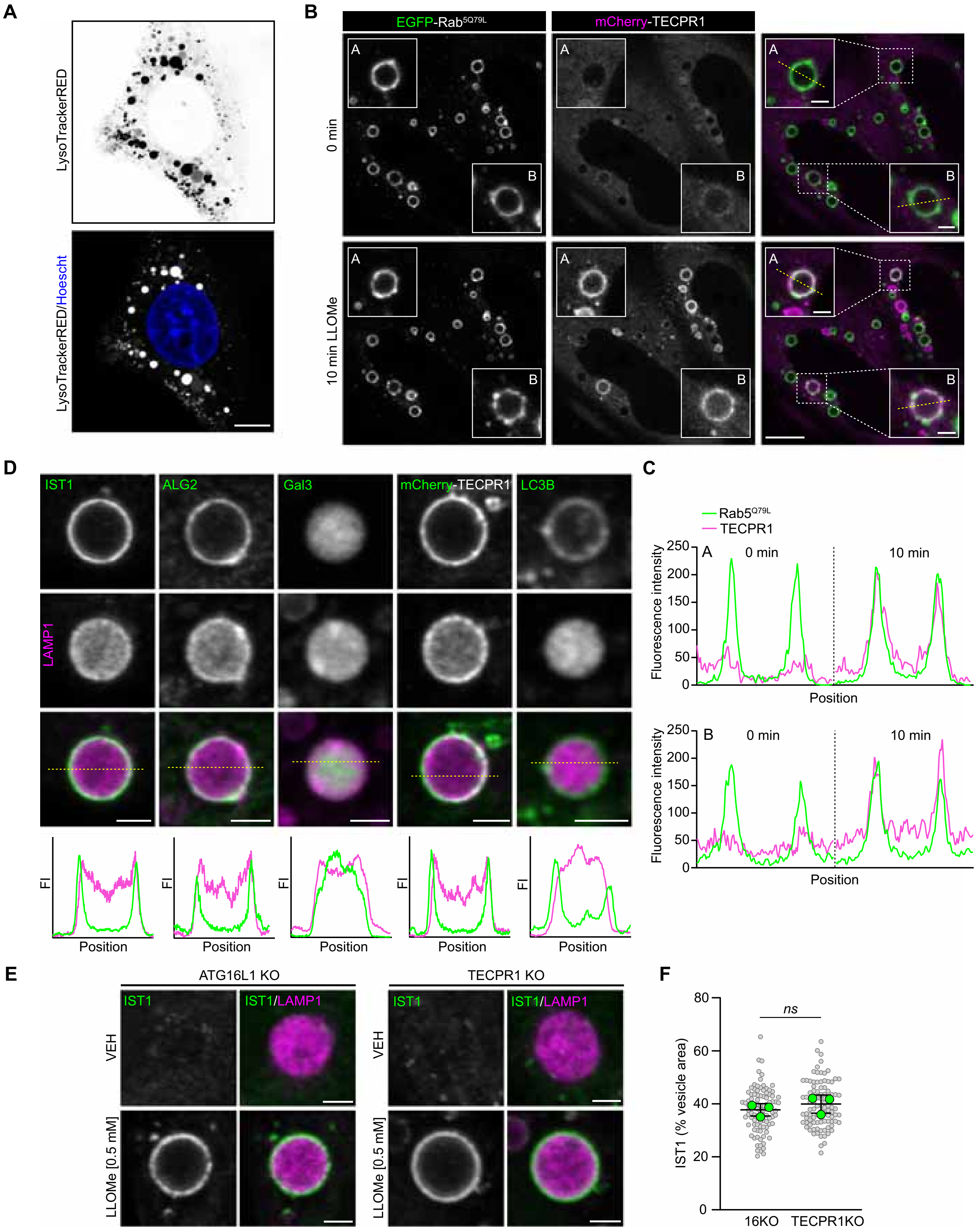
A
LysoTrackerRED
LysoTrackerRED/Hoescht
B
EGFP-Rab5Q79L
mCherry-TECPR1
0 min
10 min LLOMe
C
Rab5Q79L
TECPR1
Fluorescence intensity
0 min
10 min
Position
D
IST1
ALG2
Gal3
mCherry-TECPR1
LC3B
LAMP1
FI
Position
E
ATG16L1 KO
TECPR1 KO
IST1
IST1/LAMP1
VEH
LLOMe [0.5 mM]
F
ns
IST1 (% vesicle area)
16KO
TECPR1KO

Figure EV3. Rab5^{Q79L}-induced oversized vesicles can be used to model the LLOMe-induced membrane damage response.

(**A**) Confocal images of a HeLa cell stably expressing Rab5^{Q79L}, stained with LysoTrackerRED. Nuclei were stained with Hoechst. Scale bar = 10 µm. (**B**) Live cell imaging of HeLa cells stable expressing EGFP-Rab5^{Q79L}, co-transfected with mCherry-TECPR1, and treated with 0.5 mM LLOMe for 10 min. Scale bars = 10 µm for whole cell images and 2 µm for single vesicle insets. (**C**) Fluorescence intensity profiles of Rab5^{Q79L} and TECPR1 at oversized vesicles from (**A**), before and after LLOMe treatment (location marked by a yellow line on the inset image). (**D**) Confocal images of oversized vesicles from HeLa cells stably expressing Rab5^{Q79L} treated with 0.5 mM LLOMe for 10 min. Scale bars = 2 µm. Corresponding fluorescence intensity (FI) profiles are shown below (location marked by a yellow line on the merged image). (**E**) Confocal images of oversized vesicles from HeLa ATG16L1 KO and TECPR1 KO cells stably expressing EGFP-Rab5Q79L treated with 0.5 mM LLOMe for 10 min. Scale bars = 2 µm. (**F**) Quantification of IST1 lysosomal area from (**A**). Grey points represent individual vesicles from three independent experiments ($n \geq 30$ vesicles per experiment). Significance was determined from biological replicates using a Student's *t* test. ns (not significant) represents $P > 0.05$. Source data are available online for this figure.

 © The Author(s)

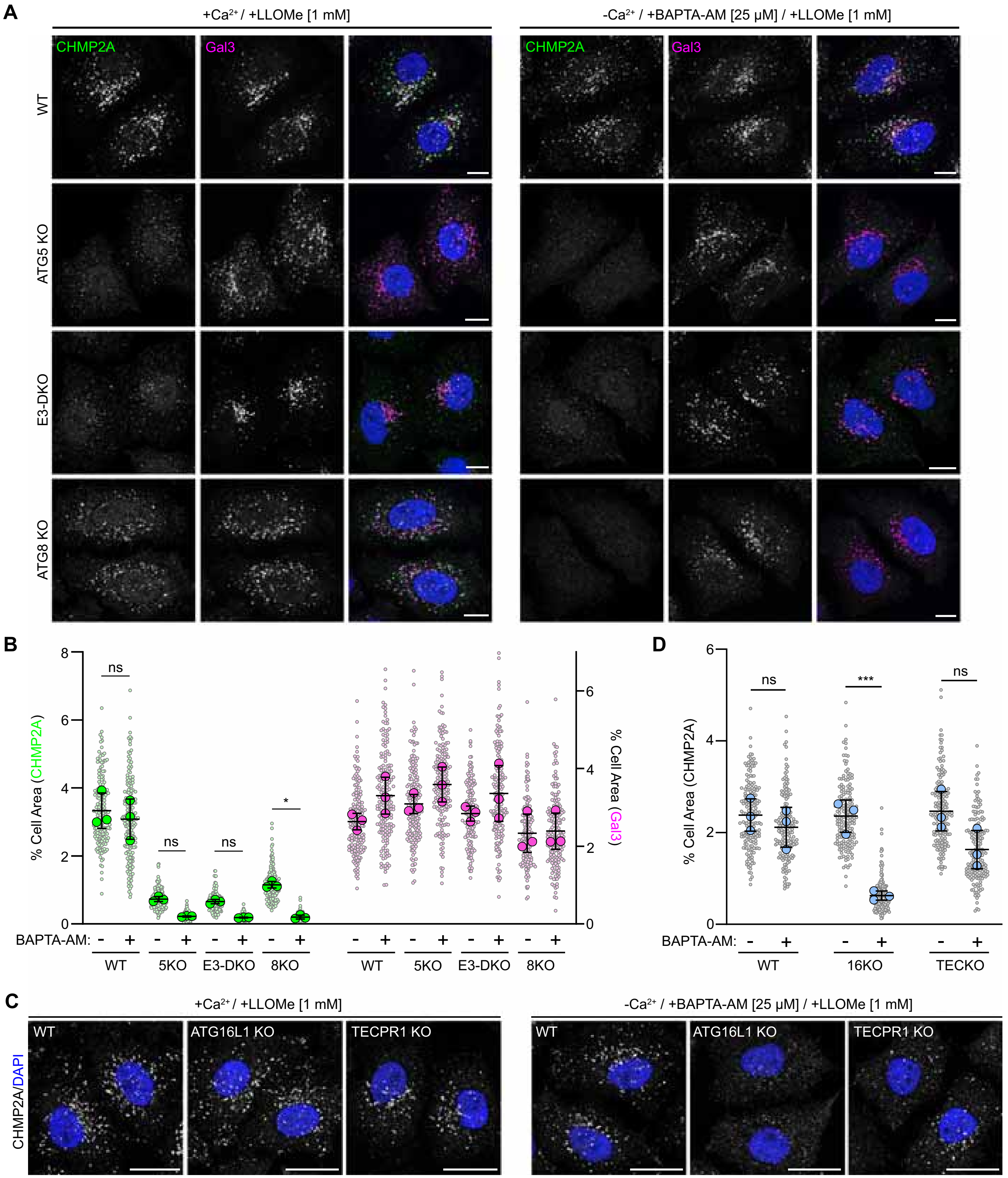
A
+Ca²⁺ / +LLOMe [1 mM]
-Ca²⁺ / +BAPTA-AM [25 µM] / +LLOMe [1 mM]
CHMP2A
Gal3
WT
ATG5 KO
E3-DKO
ATG8 KO
B
% Cell Area (CHMP2A)
% Cell Area (Gal3)
ns
*
BAPTA-AM:
WT
5KO
E3-DKO
8KO
D
% Cell Area (CHMP2A)
***
16KO
TECKO
C
WT
ATG16L1 KO
TECPR1 KO
CHMP2A/DAPI

Figure EV4. Calcium chelation inhibits ESCRT recruitment most profoundly in the absence of ATG16L1.

(**A**) Confocal images of HeLa WT, ATG5 KO, E3-DKO and ATG8 KO cells treated with or without BAPTA-AM followed by 1 mM LLOMe for 30 min and immunostained for CHMP2A and Gal3. Scale bars = 10 µm. (**B**) Quantification of CHMP2A/Gal3 cell area from (**A**). Small points represent individual cells from three independent experiments. Large points represent the means of individual experiments ($n = 60$ cells per experiment). Bars represent the mean ± SD from the three experiments. Significance was determined from biological replicates using a one-way ANOVA with Tukey's multiple comparisons tests. ns (not significant) represents $P > 0.05$, *$P = 0.0134$. (**C**) Confocal images of HeLa WT, ATG16L1 KO and TECPR1 KO cells treated with or without BAPTA-AM followed by 1 mM LLOMe for 30 min and immunostained for CHMP2A. Scale bars = 20 µm. (**D**) Quantification of CHMP2A cell area from (**C**). Small points represent individual cells from three independent experiments. Large points represent the means of individual experiments ($n = 60$ cells per experiment). Bars represent the mean ± SD from the three experiments. Significance was determined from biological replicates using a one-way ANOVA with Tukey's multiple comparisons tests. ns (not significant) represents $P > 0.05$, ***$P = 0.0009$. Source data are available online for this figure.

© The Author(s)

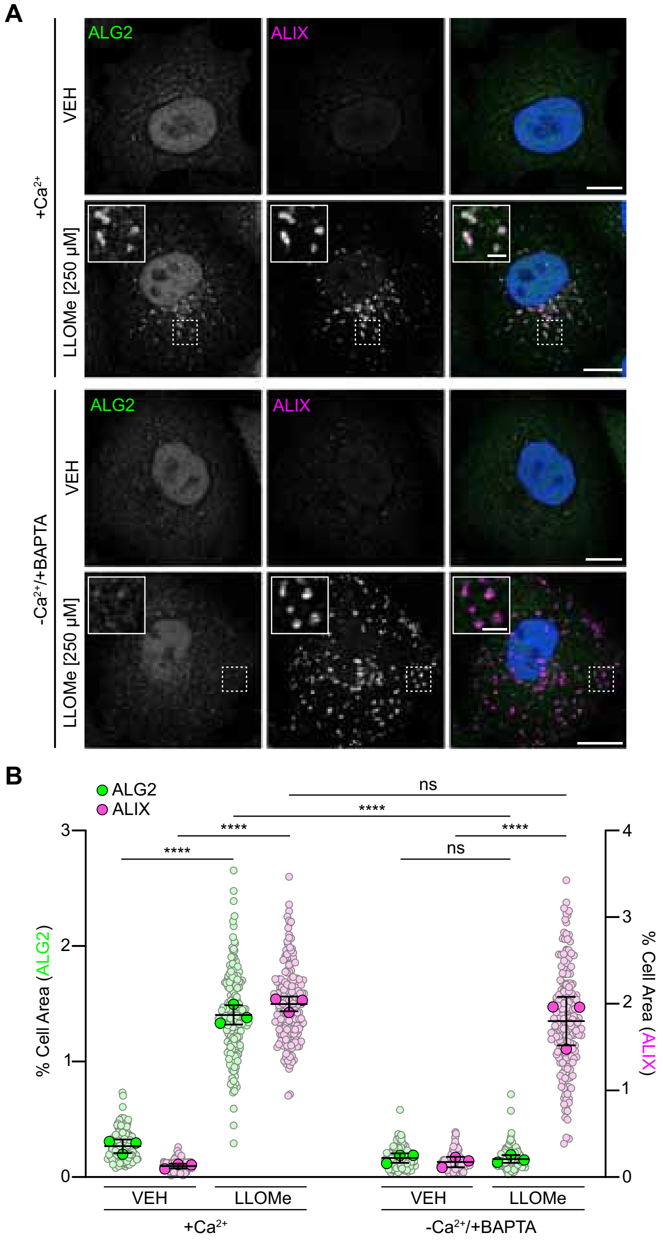

Figure EV5. Calcium chelation prevents ALG-2 recruitment to damaged lysosomes.

(**A**) Confocal images of HeLa WT cells treated with or without BAPTA-AM followed by 1 mM LLOMe for 30 min and immunostained for ALG-2 and ALIX. Scale bars = 10 µm for whole cell images and 2 µm for insets. (**B**) Quantification of ALG-2/ALIX cell area from (**A**). Small points represent individual cells from three independent experiments. Large points represent the means of individual experiments ($n = 60$ cells per experiment). Bars represent the mean ± SD from the three experiments. Significance was determined from biological replicates using a one-way ANOVA with Tukey's multiple comparisons tests. ns (not significant) (LLOMe ALIX, $P = 0.4283$; BAPTA VEH/LLOMe ALG2, $P = 0.9981$), ****$P < 0.0001$. Source data are available online for this figure.

© The Author(s)
